# C2-ketonylation of carbohydrates *via* excited-state palladium-catalyzed 1,2-spin-center shift†

**DOI:** 10.1039/d2sc01042a

**Published:** 2022-05-11

**Authors:** Gaoyuan Zhao, Upasana Mukherjee, Lin Zhou, Yue Wu, Wang Yao, Jaclyn N. Mauro, Peng Liu, Ming-Yu Ngai

**Affiliations:** Department of Chemistry, Institute of Chemical Biology and Drug Discovery, The State University of New York at Stony Brook Stony Brook New York 11794 USA ming-yu.ngai@stonybrook.edu; Department of Chemistry, Department of Chemical and Petroleum Engineering, University of Pittsburgh Pittsburgh Pennsylvania 15260 USA pengliu@pitt.edu

## Abstract

C2-ketonyl-2-deoxysugars, sugars with the C2-hydroxyl group replaced by a ketone side chain, are important carbohydrate mimetics in glycobiology and drug discovery studies; however, their preparation remains a vital challenge in organic synthesis. Here we report the first direct strategy to synthesize this class of glycomimetics from readily available 1-bromosugars and silyl enol ethers *via* an excited-state palladium-catalyzed 1,2-spin-center shift (SCS) process. This step-economic reaction features broad substrate scope, has a high functional group tolerance, and can be used in late-stage functionalization of natural product- and drug-glycoconjugates. Preliminary experimental and computational mechanistic studies suggested a non-chain radical mechanism involving photoexcited palladium species, a 1,2-SCS process, and a radical Mizoroki–Heck reaction.

## Abstract 2

The excited-state palladium-catalyzed 1,2-spin-center shift process streamlines the synthesis of C2-ketonyl sugars. This step-economic reaction has a broad scope and allows late-stage functionalization of natural product- and drug-glycoconjugates.

## Graphical abstract

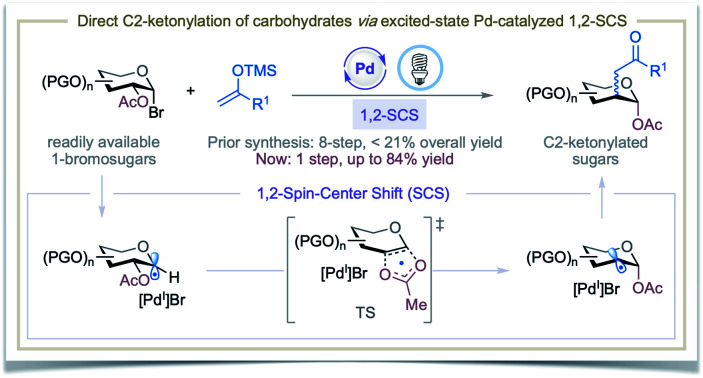

## Introduction

**Fig. 1:**
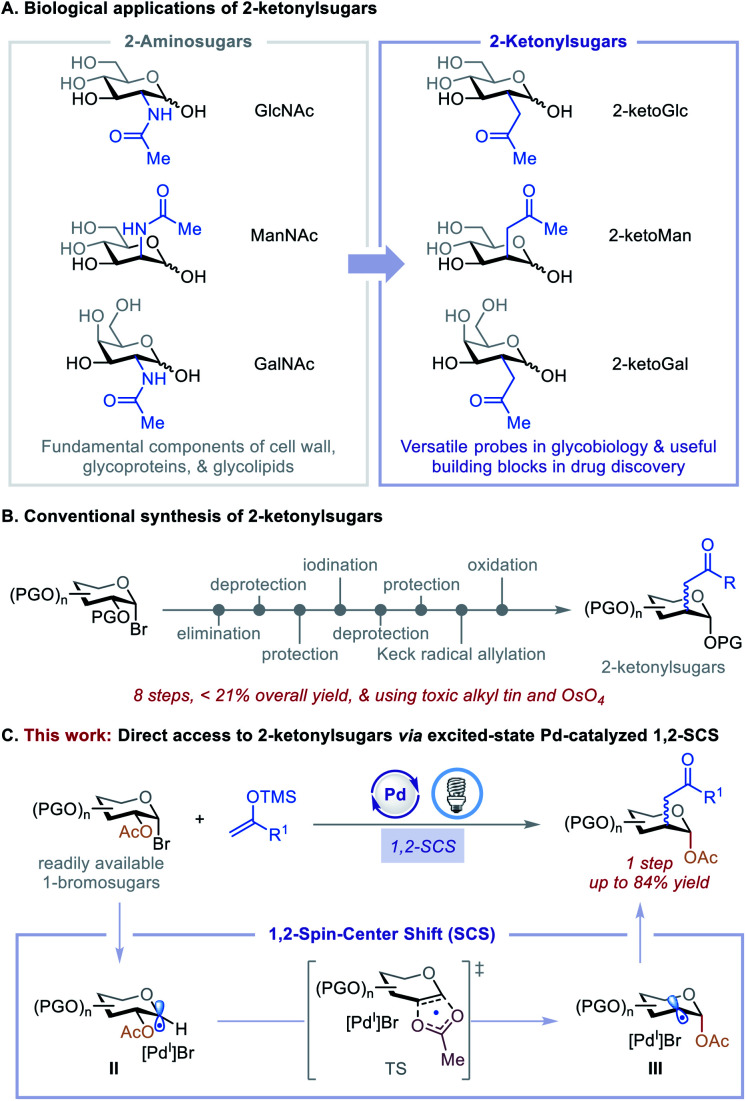
Applications and synthesis of C2-ketonylated carbohydrates.

Carbohydrates with substituents at C2 are ubiquitous in nature and feature prominently in bioactive agents and natural products, including many clinically significant antiviral, anticancer, and antibiotic drugs.^1^ For example, *N*-acetylglucosamine (GlcNAc), *N*-acetylmannosamine (ManNAc), and *N*-acetylgalactosamine (GalNAc) can be found in living organisms ranging from bacteria to vertebrates and are fundamental components of the cell wall, glycoproteins, and glycolipids (Fig. 1A).^1*a*^ Consequently, their C2-carbon isosteres, C2-ketonylsugars such as 2-ketoGlc, 2-ketoMan, and 2-ketoGal, have been synthesized for the development of antibiotics^2^ and to study cell surface recognition, metabolic pathways, and the mechanism of polysaccharide formation and protein post-translational modifications.^3^ However, the preparation of C2-ketonylsugars is labor-intensive and time-consuming. It requires an 8-step procedure with less than 21% overall yield, multiple protection/deprotection protocols, and the use of toxic alkyl tin and OsO_4_ reagents (Fig. 1B).^3*a*,*b*,4^ This synthetic strategy is substrate-specific, and it is difficult to produce other such C2-ketonylsugars. Thus, the development of a concise, general method to access a wide array of C2-ketonylsugars from readily accessible starting materials can have a significant impact on glycobiology, medicinal chemistry, and drug discovery.

To develop such a transformation, we were drawn to a 1,2-spin-center shift (SCS) process that involves a 1,2-radical translocation and a group elimination/migration.^5^ This process has been observed and studied in various biological and chemical applications.^6^ For example, during DNA biosynthesis, ribonucleotide reductase mediates the formation of deoxyribonucleoside diphosphates *via* a 1,2-SCS process.^6*c*^ In carbohydrate chemistry, a tin hydride-mediated 1,2-SCS of 1-bromosugars for the synthesis of 2-deoxysugars was developed by Giese *et al.*^7^ Inspired by these reports, we hypothesized that 1,2-SCS could serve as a reaction platform with which to develop a general, catalytic C2-functionalization of carbohydrates. The feasibility of this hypothesis was recently demonstrated by our preliminary studies using nickel catalysis^8,9^ and excited-state palladium catalysis.^10,11^ On the basis of these initial findings and the recent development of the excited-state Pd-catalyzed radical Mizoroki–Heck reactions,^10*i*,*j*,12^ we questioned whether we could merge these two reactivities to achieve a catalytic, one-step C2-ketonylation of 1-bromosugars using silyl enol ethers as coupling partners. The mechanism involves the generation of a 1-glycosyl radical (II) followed by a concerted β-C–O bond scission and acetoxyl migration leading to a transition state (TS), then forming a deoxypyranosan-2-yl radical (III) (Fig. 1C). A subsequent radical Mizoroki–Heck reaction and hydrolysis furnishes the desired C2-ketonylsugars. Realization of such a reaction would be novel and significant because it (i) greatly streamlines the synthesis of C2-ketonylsugars from an 8-step protocol to a single-step procedure, (ii) expands the reactivity profile of the excited-state Pd catalysis, and (iii) provides a new strategy for the preparation of useful glycomimetics to tackle fundamental questions in glycobiology and drug discovery.

## Results and discussion

According to the postulated mechanism in Fig. 1C, we commenced our study by investigating the reaction of acetyl-protected 1-glucosyl bromide 1a with acetophenone trimethylsilyl enol ether 2a under photoexcited Pd-catalyzed conditions (Table 1). To our delighted, in the presence of 5.00 mol% Pd(PPh_3_)_4_, 6.00 mol% Xantphos, and 1.50 equiv. KOAc in benzene (0.015 M) at 90 °C under the irradiation of 36 W blue LEDs for 24 h, the desired C2-ketonylsugar 3a was obtained in 85% yield with 4.5 : 1 axial : equatorial (ax : eq) selectivity together with a small amount of the C1-ketonylsugar byproduct (entry 1). Pd(PPh_3_)_4_ was critical for this reaction since no reaction occurred in its absence, and only 10% of the desired product was obtained by replacing Pd(PPh_3_)_4_ with Pd(OAc)_2_ (entries 2 & 3). Removal of Xantphos or replacing it with BINAP decreased the reaction yield (entries 4 & 5). Other photosensitizers, such as Ir(ppy)_3_, Ru(bpy)_3_(PF_6_)_2_, and eosin Y free acid, were either inefficient or failed to catalyze the desired reaction (entries 6–8). Other bases, such as Cs_2_CO_3_, diminished the reaction efficiency (entry 9). The use of 1,4-dioxane as a solvent formed hydro-debromination side products, lowering the product yield (entry 10). Higher reaction concentrations or lower temperatures favored the formation of the C1-ketonylsugar side product, decreasing the yield of the desired product 3a (entries 11 & 12). Control experiments confirmed that both oxygen-free conditions and visible light were essential for product formation (entries 13 & 14).

**Table 1:** Selected optimization experiments^a^

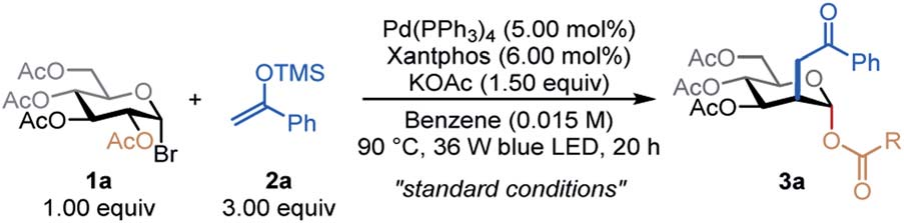			
Entry	Deviation from standard conditions	Yield (%)	ax/eq
1	None	85	4.5 : 1
2	Without Pd(PPh_3_)_4_	N.R.	—
3	Pd(OAc)_2_ instead of Pd(PPh_3_)_4_	10	3.2 : 1
4	Without xantphos	18	4.0 : 1
5	BINAP instead of xantphos	36	4.3 : 1
6	Ir(ppy)_3_ as photocatalyst	24	3.0 : 1
7	Ru(bpy)_3_(PF_6_)_2_ as photocatalyst	N.R.	—
8	Eosin Y free acid as photocatalyst	N.R.	—
9	Cs_2_CO_3_ as base	35	4.6 : 1
10	Dioxane as solvent	33	3.7 : 1
11	0.10 M	25	5.0 : 1
12	RT	39	5.1 : 1
13	Air	N.R.	—
14	Keep in dark	0	—

a See ESI for Experimental details. Reaction yields and axial to equatorial (ax/eq) ratios were determined by 1H-NMR using CH_2_Br_2_ as an internal standard. Ac, acetyl; BINAP, 2,2′-bis(diphenylphosphino)-1,1′-binaphthyl; LED, light-emitting diode; N.R., no reaction.

With optimized conditions in hand, we examined scope of the different silyl enol ethers in the reaction. As shown in Table 2, A, a diverse array of silyl enol ethers proved to be competent coupling partners for the C2-ketonylation protocol. Aryl silyl enol ethers with electron-neutral (2a), electron-withdrawing (2b–2e), or electron-donating (2f) substituents on the aryl ring reacted well, delivering the corresponding C2-ketonyl glucosides (3a–3f) in 61–84% yields and with 4.4 : 1 to 6.0 : 1 ax : eq selectivity. Aryl silyl enol ethers with multiple substituents (2g–2h) or extended conjugation (2i–2j) were compatible. The transformation is effective for compounds with medicinally relevant heterocyclic derivatives, such as pyridyl (2k) or thiophenyl (2l). Alkyl silyl enol ethers were also viable substrates, furnishing the desired products (2m–2o) with moderate to good yields. Silyl vinyl ether, a surrogate for acetaldehyde, couples with the C2-radical to give the C2-formylmethyl glucoside 3p in 81% yield and 5.1 : 1 ax : eq selectivity. When the silyl enol ether (2q) derived from *tert*-butyl acetate was employed as a substrate, the C2-carboxymethyl glucoside 3q was generated, presumably through the hydrolysis of the resulting silyl enol intermediate. Finally, the C2-acetamidated product 3r could also be obtained with this strategy. The absolute stereochemistry of the product was confirmed by a single-crystal analysis of 3o, as shown in Table 2.^13^

**Table 2:** Scope of excited-state palladium-catalyzed C2-ketonylation of carbohydrates^a^

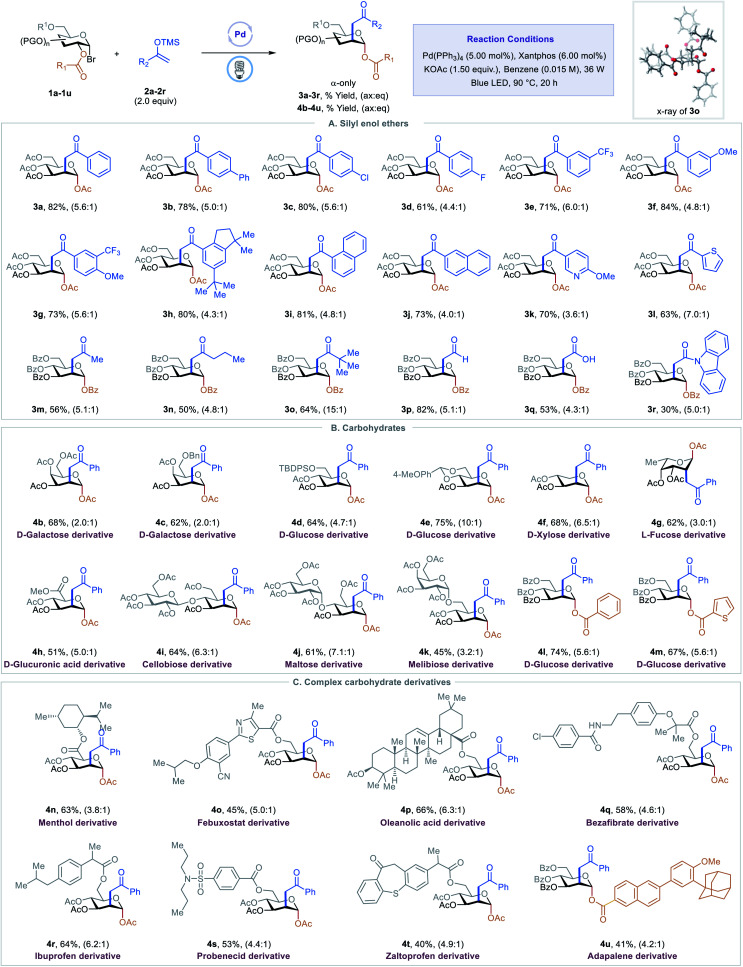

a See ESI for Experimental details. Isolated yield and ax : eq ratio are indicated below each entry.

Next, we evaluated the generality of this transformation with respect to 1-bromosugars (Table 2, B). A wide range of 1-bromosugars, including derivatives of d-galactose, d-glucose, d-xylose, and l-fucose (1b–1g), reacted with silyl enol ether 2a, affording the desired products (4b–4g) in 62–86% yields and with up to 10 : 1 ax : eq selectivity. It is noteworthy that peracetylated monosaccharide derivatives are particularly useful because they have been shown to passively diffuse through mammalian cell membranes and undergo subsequent deacetylation by cytosolic or ER esterases.^14^ Other protecting groups such as benzyl, *tert*-butyldiphenylsilyl, acetal, and benzoyl were well-tolerated as well. The d-glucuronic acid derivative (1h) was also a viable substrate, and disaccharide derivatives, such as cellobiose, maltose, and melibiose, proved to be compatible with the standard reaction conditions (4i–4k). In addition, C2-esters substituted with aryl or heteroaryl groups migrated smoothly, delivering the desired products (4l–4m) in good yields and with good selectivity.

Late-stage modification of complex molecules is often a key to identifying medicinal agents.^15^ To demonstrate the applicability of the excited-state Pd-catalyzed C2-ketonylation to late-stage syntheses, natural product- and drug-conjugated sugars were subjected to the standard reaction conditions (Table 2, C). For example, 1-bromosugar derivatives of l-menthol (decongestant and analgesic), febuxostat (an anti-hyperuricemic drug), oleanolic acid, bezafibrate (antilipemic agent), ibuprofen (non-steroidal anti-inflammatory drug, NSAID), probenecid (anti-gout), zaltoprofen (NSAID), and adapalene (antiacne agent) worked well under the standard conditions, affording the desired products 4n–4u in 40–66% yields and with up to 6.3 : 1 ax : eq selectivity.

The C2-ketonylsugar products are useful synthetic intermediates and can be converted into other novel glycomimetics (Table 3). For example, C2-ketonylated glycoside could be reduced to C2-hydroxyalkylated glycoside 5a and C2-alkylated glycoside 6a (Table 3, A and B). Cyclopropanated glycoside 7a, an important glycosylation donor,^16^ could be prepared from the C2-ketonylsugar (Table 3, C). Under Lewis acid activation conditions, 3a undergoes cyclization, and the resulting carbocation can be trapped with furan, affording perhydrofuro[2,3-*b*]pyran 8a in good yield (Table 3, D).^17^ C2-ketonylsugars can also serve as good glycosylation donors. For example, *N*/*S*/*O*-glycosylation of 3a proceeds smoothly, furnishing glycosyl azide 9a, thioglycoside 10a, *trans*-androsteronyl glycoside 12a, and disaccharide 14a in good yields and up to 20 : 1 α/β-selectivity (Table 3, E–H).

**Table 3:** Post-functionalization of C2-ketonylsugars^a^

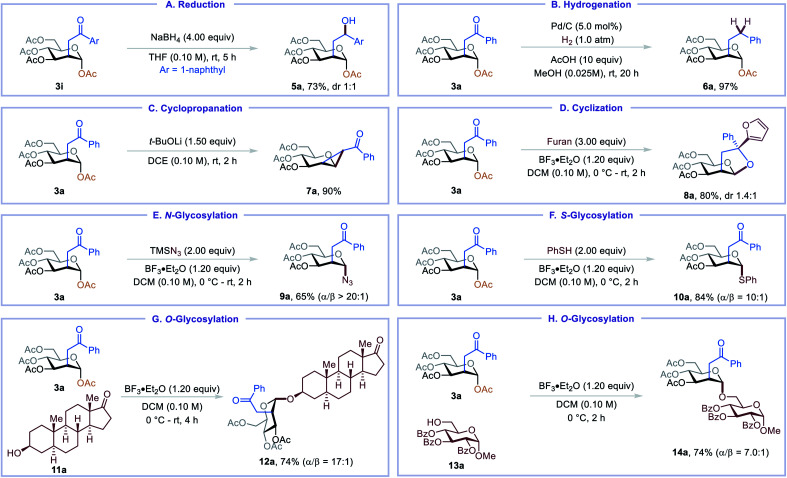

a See ESI for Experimental details. Isolated yield and diastereomeric ratio are indicated below each entry.

**Fig. 2:**
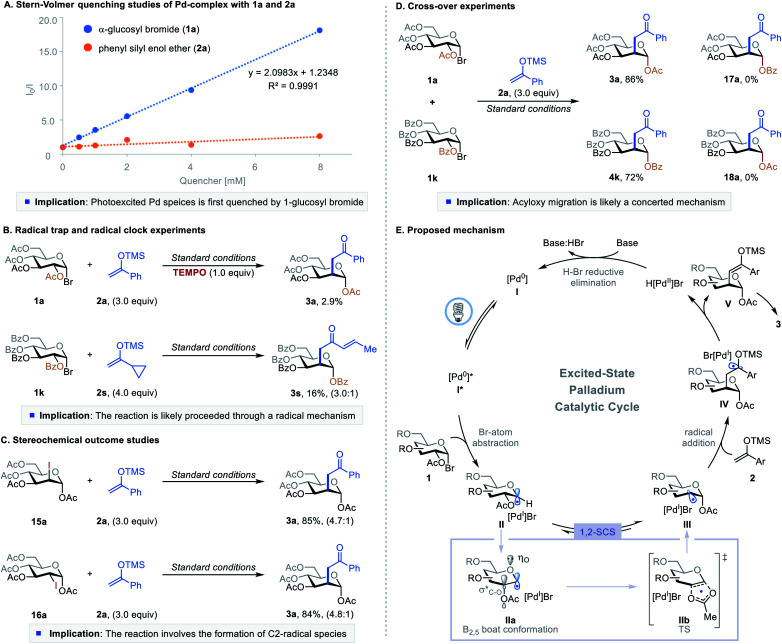
Mechanistic studies and proposed mechanism. See ESI† for Experimental details.

To better understand the mechanism of this excited-state Pd-catalyzed C2-ketonylation, we conducted a series of experimental and computational studies (Fig. 2). First, Stern–Volmer quenching studies showed that 1-glucosyl bromide 1a quenches the excited palladium species more efficiently than silyl enol ether 2a (Fig. 2A). The radical-trapping experiment using 2,2,6,6-tetramethylpiperidine 1-oxyl (TEMPO) as the scavenger significantly inhibited the reaction, and the radical clock reaction gave the desired product with a ring-opening (3s), implying the reaction proceeds through a radical pathway (Fig. 2B). However, radical chain propagation is unlikely as the quantum yield of the reaction was found to be 0.009 (Fig. S9 in the ESI†). When the 1,2-*trans*- and 1,2-*cis* 2-iodo-sugar (15a and 16a, respectively) were subjected to the reaction conditions, they both formed the desired product (3a) with similar yields and stereoselectivity as produced by the parent reaction (Fig. 2C*vs.*Table 1, entry 1) and without the formation of C1-ketonylated side products. These results suggest that the reaction proceeds through a common deoxypyranosan-2-yl radical intermediate (III), and the reverse acetoxy migration is slower than the addition of radical III to silyl enol ethers. Furthermore, crossover experiments using substrates 1a and 1k afforded only the non-crossover products 3a and 4k, suggesting that the 1,2-SCS probably takes place through an in-cage or a concerted mechanism (Fig. 2D). DFT calculations showed that the addition of C2-radical III to the silyl enol ether *via* transition state TS1-ax to form IV-ax is more favorable than the formation of the equatorial isomer IV-eq*via*TS1-eq (Fig. S11 in the ESI†).

Combining the insights gained from these experiments and published reports,^10–12^ a plausible mechanism is shown in Fig. 2E. The photoexcited species [Pd^0^]* abstracts a bromine atom from 1-bromosugar 1, forming a [Pd^I^]Br complex and 1-glycosyl radical intermediate II. This radical intermediate then undergoes a 1,2-SCS pathway through a conformational change (IIa) followed by concerted [2,3]-acyloxy migration (IIb) under the standard reaction conditions, generating deoxypyranosan-2-yl radical III. Although the C2-radical is more reactive than the C1-radical, the formation of an anomeric C–O bond lowers the molecular energy of III and drives the migration.^7*b*,9^ C2-radical species III adds to silyl enol ether 2, furnishing intermediate IV. Pd-catalyzed β-hydride elimination or palladoradical H-atom abstraction liberates H[Pd^II^]Br and silyl enol ether V, which upon hydrolysis affords the desired product 3. Meanwhile, base-assisted H–Br reductive elimination of H[Pd^II^]Br regenerates the ground state [Pd^0^] catalyst, closing the catalytic cycle.^18^

## Conclusions

In summary, we have developed a one-step synthesis of valuable C2-ketonylsugars from readily available 1-bromosugars and silyl enol ethers *via* a 1,2-SCS process catalyzed by excited-state palladium. The reaction features a broad substrate scope, tolerates a wide range of functional groups, and is amenable to late-stage modification of disaccharides, natural product- and drug-glycoconjugates. Preliminary experimental and computational mechanistic studies suggest a non-chain radical mechanism involving photoexcited Pd-complexes, a 1,2-SCS process, and a Mizoroki–Heck reaction. The catalytic 1,2-SCS process *via* a [2,3]-acyloxy migration could (i) offer a general catalytic strategy for the site-selective functionalization of carbohydrates to access a wide array of unexplored carbohydrate mimetics and (ii) guide the design and development of new transformations in organic synthesis.

## Data availability

All the data associated with this manuscript were provided in ESI.†

## Author contributions

G. Z., U. M., W. Y., and J. N. M performed the experiments, synthesized starting materials, developed substrate scope, and conducted detailed mechanistic studies. L. Z., Y. W., and P. L. designed and performed the DFT calculations. G. Z. and M.-Y. N. conceived the idea, designed the research, and wrote the manuscript. All the authors commented on the final draft of the manuscript and contributed to the analysis and interpretation of the data.

## Conflicts of interest

There are no conflicts to declare.

## Supplementary Material

SC-013-D2SC01042A-s001

SC-013-D2SC01042A-s002
