# Supplementary material for: C2-ketonylation of carbohydrates via excited-state palladium-catalyzed 1,2-spin-center shift
Source: Chem Sci. 2022 May 11;13(21):6276–82. doi: 10.1039/d2sc01042a (PMC9159084; doi:10.1039/d2sc01042a)
Supplement: SC-013-D2SC01042A-s001 [file SC-013-D2SC01042A-s001.pdf]

Electronic Supplementary Material (ESI) for Chemical Science.
This journal is © The Royal Society of Chemistry 2022

Electronic Supplementary Information

C2-Ketonylation of Carbohydrates via Excited-State Palladium-Catalyzed 1,2-Spin-Center Shift

Gaoyuan Zhao,[1] Upasana Mukherjee,[1] Lin Zhou,[3] Yue Wu,[3] Wang Yao,[1] Jaclyn N. Mauro,[1] Peng Liu,[*,3,4] and Ming-Yu Ngai[*,1,2]

pengliu@pitt.edu
ming-yu.ngai@stonybrook.edu

[1]Department of Chemistry, State University of New York, Stony Brook, New York, 11794, United States;
[2]Institute of Chemical Biology and Drug Discovery, State University of New York, Stony Brook, New York 11794, United States;
[3]Department of Chemistry, University of Pittsburgh, Pittsburgh, Pennsylvania 15260, United States;
[4]Department of Chemical and Petroleum Engineering, University of Pittsburgh, Pittsburgh, Pennsylvania 15261, United States.

Table of Contents

General Information 5

Photoredox Reaction Setup 6

Experimental Data 7

General Procedure A (for the synthesis of silyl enol ethers): 7
Trimethyl((1-phenylvinyl)oxy)silane (2a) 7
((1-([1,1'-biphenyl]-4-yl)vinyl)oxy)trimethylsilane (2b) 7
((1-(4-chlorophenyl)vinyl)oxy)trimethylsilane (2c) 8
((1-(4-fluorophenyl)vinyl)oxy)trimethylsilane (2d) 8
((1-(3-methoxyphenyl)vinyl)oxy)trimethylsilane (2f) 8
((1-(4-methoxy-3-(trifluoromethyl)phenyl)vinyl)oxy)trimethylsilane (2g) 9
((1-(6-(tert-butyl)-1,1-dimethyl-2,3-dihydro-1H-inden-4-yl)vinyl)oxy)trimethylsilane (2h) 9
((1-(naphthalen-2-yl)vinyl)oxy)trimethylsilane (2j) 10
2-methoxy-5-(1-((trimethylsilyl)oxy)vinyl)pyridine (2k) 10
((1-(thiophen-2-yl)vinyl)oxy)trimethylsilane (2l) 10
trimethyl(pent-1-en-2-yloxy)silane (2n) 11
((3,3-dimethylbut-1-en-2-yl)oxy)trimethylsilane (2o) 11
((1-(tert-butoxy)vinyl)oxy)trimethylsilane (2q) 11
9-(1-((trimethylsilyl)oxy)vinyl)-9H-carbazole (2r) 11
((1-cyclopropylvinyl)oxy)trimethylsilane (2s) 11
General Procedure B (for the synthesis of 1-bromosugars): 12
2,3,4,6-Tetra-*O*-acetyl-α-D-glucopyranosyl bromide (1a) 12
2,3,4,6-Tetra-*O*-acetyl-α-D-galactopyranosyl bromide (1b) 12
2,3,4-Tri-*O*-acetyl-6-*O*-benzyl-α-D-galactopyranosyl bromide (1c) 13
2,3,4-Tri-*O*-acetyl-6-*O*-[(1,1-dimethylethyl)diphenylsilyl]-α-D-glucopyranosyl bromide (1d) 13
2,3-Di-*O*-acetyl-4,6-*O*-[(*R*)-(4-methoxyphenyl)methylene]-α-D-glucopyranosyl bromide (1e) 14
2,3,4-Tri-*O*-acetyl-α-D-xylopyranosyl bromide (1f) 14
2,3,4-Tri-*O*-acetyl-α-L-fucopyranosyl bromide (1g) 14
(2R,3R,4S,5S,6S)-2-bromo-6-(methoxycarbonyl)tetrahydro-2H-pyran-3,4,5-triyl triacetate (1h) 15
2,3,6,2',3',4',6'-Hepta-*O*-acetyl-α-D-cellobiose bromide (1i) 15
2,3,6,2',3',4',6'-Hepta-*O*-acetyl-α-D-maltose bromide (1j) 15
2,3,4,2',3',4',6'-Hepta-*O*-acetyl-α-D-melibiosyl bromide (1k) 16
2,3,4,6-Tetra-*O*-benzoyl-α-D-glucopyranosyl bromide (1l) 16
3,4,6-Tri-*O*-Benzoyl-2-*O*-(thiophene-2-carbonyl)-α-D-glucopyranosyl bromide (1m) 17
(2R,3R,4S,5S,6S)-2-bromo-6-((((1S,2R,5S)-2-isopropyl-5-methylcyclohexyl)oxy)carbonyl)tetrahydro-2H-pyran-3,4,5-triyl triacetate (1n) 18
2,3,4-Tri-*O*-acetyl-6-*O*-[(2-(3-cyano-4-isobutoxyphenyl)-4-methylthiazole-5-carbonyl)]-α-D-glucopyranosyl bromide (1o) 18

2,3,4-Tri-*O*-acetyl-6-*O*-[((4aS,6aS,6bR,8aR,10S,12aR,12bR,14bS)-10-acetoxy-2,2,6a,6b,9,9,12a-heptamethyl-1,2,3,4,4a,5,6,6a,6b,7,8,8a,9,10,11,12,12a,12b,13,14b-icosahydropicene-4a-carbonyl)]-α-D-glucopyranosyl bromide (1p) 19
2,3,4-Tri-*O*-acetyl-6-*O*-[(2-(4-(2-(4-chlorobenzamido)ethyl)phenoxy)-2-methylpropanoyl)]-α-D-glucopyranosyl bromide (1q) 20
2,3,4-Tri-*O*-acetyl-6-*O*-[((2-(4-isobutylphenyl)propanoyl)]-α-D-glucopyranosyl bromide (1r) 20
2,3,4-Tri-*O*-acetyl-6-*O*-[(4-(*N,N*-dipropylsulfamoyl)benzoyl)]-α-D-glucopyranosyl bromide (1s) 21
2,3,4-Tri-*O*-acetyl-6-*O*-[((2-(10-oxo-10,11-dihydrodibenzo[*b,f*]thiepin-2-yl)propanoyl)]-α-D-glucopyranosyl bromide (1t) 21
3,4,6-Tri-*O*-benzoyl-6-*O*-[(6-(3-((3r,5r,7r)-adamantan-1-yl)-4-methoxyphenyl)-2-naphthoyl)]-α-D-glucopyranosyl bromide (1z) 22
General Procedure C (for the C2-ketonylation reaction): 23
(2R,3S,4R,5S,6R)-6-(acetoxymethyl)-3-(2-oxo-2-phenylethyl)tetrahydro-2H-pyran-2,4,5-triyl triacetate (3a) 24
(2R,3S,4R,5S,6R)-3-(2-([1,1'-biphenyl]-4-yl)-2-oxoethyl)-6-(acetoxymethyl)tetrahydro-2H-pyran-2,4,5-triyl triacetate (3b) 24
(2R,3S,4R,5S,6R)-6-(acetoxymethyl)-3-(2-(4-chlorophenyl)-2-oxoethyl)tetrahydro-2H-pyran-2,4,5-triyl triacetate (3c) 25
(2R,3S,4R,5S,6R)-6-(acetoxymethyl)-3-(2-(4-fluorophenyl)-2-oxoethyl)tetrahydro-2H-pyran-2,4,5-triyl triacetate (3d) 26
(2R,3S,4R,5S,6R)-6-(acetoxymethyl)-3-(2-oxo-2-(3-(trifluoromethyl)phenyl)ethyl)tetrahydro-2H-pyran-2,4,5-triyl triacetate (3e) 27
(2R,3S,4R,5S,6R)-6-(acetoxymethyl)-3-(2-(3-methoxyphenyl)-2-oxoethyl)tetrahydro-2H-pyran-2,4,5-triyl triacetate (3f) 27
(2R,3S,4R,5S,6R)-6-(acetoxymethyl)-3-(2-(4-methoxy-3-(trifluoromethyl)phenyl)-2-oxoethyl)tetrahydro-2H-pyran-2,4,5-triyl triacetate (3g) 28
(2R,3S,4R,5S,6R)-6-(acetoxymethyl)-3-(2-(6-(tert-butyl)-1,1-dimethyl-2,3-dihydro-1H-inden-4-yl)-2-oxoethyl)tetrahydro-2H-pyran-2,4,5-triyl triacetate (3h) 28
(2R,3S,4R,5S,6R)-6-(acetoxymethyl)-3-(2-(naphthalen-1-yl)-2-oxoethyl)tetrahydro-2H-pyran-2,4,5-triyl triacetate (3i) 29
(2R,3S,4R,5S,6R)-6-(acetoxymethyl)-3-(2-(naphthalen-2-yl)-2-oxoethyl)tetrahydro-2H-pyran-2,4,5-triyl triacetate (3j) 30
(2R,3S,4R,5S,6R)-6-(acetoxymethyl)-3-(2-(6-methoxypyridin-3-yl)-2-oxoethyl)tetrahydro-2H-pyran-2,4,5-triyl triacetate (3k) 31
(2R,3S,4R,5S,6R)-6-(acetoxymethyl)-3-(2-oxo-2-(thiophen-2-yl)ethyl)tetrahydro-2H-pyran-2,4,5-triyl triacetate (3l) 32
(2R,3S,4R,5S,6R)-6-((benzoyloxy)methyl)-3-(2-oxopropyl)tetrahydro-2H-pyran-2,4,5-triyl tribenzoate (3m) 32
(2R,3S,4R,5S,6R)-6-((benzoyloxy)methyl)-3-(2-oxopentyl)tetrahydro-2H-pyran-2,4,5-triyl tribenzoate (3n) 33

(2R,3S,4R,5S,6R)-6-((benzoyloxy)methyl)-3-(3,3-dimethyl-2-oxobutyl)tetrahydro-2H-pyran-2,4,5-triyl tribenzoate (3o) 34
(2R,3S,4R,5S,6R)-6-((benzoyloxy)methyl)-3-(2-oxoethyl)tetrahydro-2H-pyran-2,4,5-triyl tribenzoate (3p) 34
2-((2R,3S,4R,5S,6R)-2,4,5-tris(benzoyloxy)-6-((benzoyloxy)methyl)tetrahydro-2H-pyran-3-yl)acetic acid (3q) 35
(2R,3S,4R,5S,6R)-3-(2-(9H-carbazol-9-yl)-2-oxoethyl)-6-((benzoyloxy)methyl)tetrahydro-2H-pyran-2,4,5-triyl tribenzoate (3r) 36
(2R,3S,4R,5R,6R)-6-(acetoxymethyl)-3-(2-oxo-2-phenylethyl)tetrahydro-2H-pyran-2,4,5-triyl triacetate (4b) 36
(2R,3S,4R,5R,6R)-6-((benzyloxy)methyl)-3-(2-oxo-2-phenylethyl)tetrahydro-2H-pyran-2,4,5-triyl triacetate (4c) 37
(2R,3S,4R,5S,6R)-6-(((tert-butyldiphenylsilyl)oxy)methyl)-3-(2-oxo-2-phenylethyl)tetrahydro-2H-pyran-2,4,5-triyl triacetate (4d) 38
(2R,4aR,6R,7S,8R,8aS)-2-(4-methoxyphenyl)-7-(2-oxo-2-phenylethyl)hexahydropyrano[3,2-d][1,3]dioxine-6,8-diyl diacetate (4e) 39
(2R,3S,4R,5R)-3-(2-oxo-2-phenylethyl)tetrahydro-2H-pyran-2,4,5-triyl triacetate (4f) 39
(2S,3R,4S,5R,6S)-6-methyl-3-(2-oxo-2-phenylethyl)tetrahydro-2H-pyran-2,4,5-triyl triacetate (4g) 40
(2R,3S,4R,5S,6S)-6-(methoxycarbonyl)-3-(2-oxo-2-phenylethyl)tetrahydro-2H-pyran-2,4,5-triyl triacetate (4h) 41
(2R,3R,4S,5R,6S)-2-(acetoxymethyl)-6-(((2R,3S,4R,5S,6R)-4,6-diacetoxy-2-(acetoxymethyl)-5-(2-oxo-2-phenylethyl)tetrahydro-2H-pyran-3-yl)oxy)tetrahydro-2H-pyran-3,4,5-triyl triacetate (4i) 41
(2R,3R,4S,5R,6R)-2-(acetoxymethyl)-6-(((2R,3S,4R,5S,6R)-4,6-diacetoxy-2-(acetoxymethyl)-5-(2-oxo-2-phenylethyl)tetrahydro-2H-pyran-3-yl)oxy)tetrahydro-2H-pyran-3,4,5-triyl triacetate (4j) 42
(2R,3S,4S,5R,6S)-2-(acetoxymethyl)-6-(((2R,3S,4R,5S,6R)-3,4,6-triacetoxy-5-(2-oxo-2-phenylethyl)tetrahydro-2H-pyran-2-yl)methoxy)tetrahydro-2H-pyran-3,4,5-triyl triacetate (4k) 43
(2R,3S,4R,5S,6R)-6-((benzoyloxy)methyl)-3-(2-oxo-2-phenylethyl)tetrahydro-2H-pyran-2,4,5-triyl tribenzoate (4l) 43
(2R,3S,4R,5S,6R)-2-((benzoyloxy)methyl)-5-(2-oxo-2-phenylethyl)-6-((thiophene-2-carbonyl)oxy)tetrahydro-2H-pyran-3,4-diyl dibenzoate (4m) 44
(2R,3S,4R,5S,6S)-6-((((1R,2S,5R)-2-isopropyl-5-methylcyclohexyl)oxy)carbonyl)-3-(2-oxo-2-phenylethyl)tetrahydro-2H-pyran-2,4,5-triyl triacetate (4n) 45
(2R,3S,4R,5S,6R)-6-(((2-(3-cyano-4-isobutoxyphenyl)-4-methylthiazole-5-carbonyl)oxy)methyl)-3-(2-oxo-2-phenylethyl)tetrahydro-2H-pyran-2,4,5-triyl triacetate (4o) 45
(2R,3S,4R,5S,6R)-6-((((4aS,6aS,6bR,8aR,10S,12aR,12bR,14bS)-10-acetoxy-2,2,6a,6b,9,9,12a-heptamethyl-1,2,3,4,4a,5,6,6a,6b,7,8,8a,9,10,11,12,12a,12b,13,14b-icosahydropicene-4a-carbonyl)oxy)methyl)-3-(2-oxo-2-phenylethyl)tetrahydro-2H-pyran-2,4,5-triyl triacetate (4p) 46

(2R,3S,4R,5S,6R)-6-(((2-(4-(2-(4-chlorobenzamido)ethyl)phenoxy)-2-methylpropanoyl)oxy)methyl)-3-(2-oxo-2-phenylethyl)tetrahydro-2H-pyran-2,4,5-triyl triacetate (4q) 46
(2R,3S,4R,5S,6R)-6-(((2-(4-isobutylphenyl)propanoyl)oxy)methyl)-3-(2-oxo-2-phenylethyl)tetrahydro-2H-pyran-2,4,5-triyl triacetate (4r) 47
(2R,3S,4R,5S,6R)-6-(((4-(N,N-dipropylsulfamoyl)benzoyl)oxy)methyl)-3-(2-oxo-2-phenylethyl)tetrahydro-2H-pyran-2,4,5-triyl triacetate (4s) 48
(2R,3S,4R,5S,6R)-6-(((2-(10-oxo-10,11-dihydrodibenzo[b,f]thiepin-2-yl)propanoyl)oxy)methyl)-3-(2-oxo-2-phenylethyl)tetrahydro-2H-pyran-2,4,5-triyl triacetate (4t) 48
(2R,3S,4R,5S,6R)-6-((6-(3-((3r,5r,7r)-adamantan-1-yl)-4-methoxyphenyl)-2-naphthoyl)oxy)-2-((benzoyloxy)methyl)-5-(2-oxo-2-phenylethyl)tetrahydro-2H-pyran-3,4-diyl dibenzoate (4u) 49
Post-functionalizations 50
Reduction of C2-Ketonylsugar: 50
Hydrogenation of C2-Ketonylsugar: 51
Cyclopropanation of C2-Ketonylsugar: 51
Cyclization: 52
N-Glycosylation of C2-Ketonylsugar: 53
S-Glycosylation of C2-Ketonylsugar: 53
O-Glycosylation of C2-Ketonylsugar: 54
(2R,3S,4R,5S,6S)-2-(acetoxymethyl)-6-(((3S,5S,8R,9S,10S,13S,14S)-10,13-dimethyl-17-oxohexadecahydro-1H-cyclopenta[a]phenanthren-3-yl)oxy)-5-(2-oxo-2-phenylethyl)tetrahydro-2H-pyran-3,4-diyl diacetate (12a): 54
(2R,3R,4S,5R,6S)-2-((((2S,3S,4R,5S,6R)-4,5-diacetoxy-6-(acetoxymethyl)-3-(2-oxo-2-phenylethyl)tetrahydro-2H-pyran-2-yl)oxy)methyl)-6-methoxytetrahydro-2H-pyran-3,4,5-triyl tribenzoate (14a): 55

Mechanistic Studies 56

Radical Trapping Experiment 56
Radical-clock Experiment 56
Studies of stereochemical outcome using 2-iodo sugar 57
Cross-over experiment 57
Stern–Volmer Luminescence Quenching Experiments 58
Quantum Yield Experiment 60
X-Ray Crystal Structure 63
DFT Calculations 65

Spectroscopic Data 67

Cartesian Coordinates (Å) and Energies of the Optimized Structures 172

References 195

General Information

All air- and moisture-insensitive reactions were carried out under an ambient atmosphere, magnetically stirred, and monitored by thin-layer chromatography (TLC) using Agela Technologies TLC plates pre-coated with 250 μm thickness silica gel 60 F254 plates and visualized by fluorescence quenching under UV light. Flash column chromatography was performed on SiliaFlash® Silica Gel 40-63μm 60 Å particle size using a forced flow of eluent at 0.3–0.5 bar pressure.[1] Preparative TLC was performed on Uniplate® UV254 (20 x 20 cm) with 1000 μm thickness and visualized fluorescence quenching under UV light.

All air and moisture-sensitive manipulations were performed using oven-dried glassware, including standard Schlenk and glovebox techniques under an atmosphere of nitrogen. All reaction vials were capped using green caps with F-217 PTFE liners. Isopropyl acetate was distilled from calcium chloride $CaCl_2$. Diethyl ether and THF were distilled from deep purple sodium benzophenone ketyl. Acetonitrile was dried ver CaH_2 and distilled. Isopropyl acetate and acetonitrile were degassed *via* three freeze-pump-thaw cycles. All other chemicals were used as received.

All deuterated solvents were purchased from Cambridge Isotope Laboratories. NMR spectra were recorded on either a Bruker Ascend 700 spectrometer operating at 700 MHz for ^1H acquisitions and 175 MHz for ^{13}C acquisitions, a Bruker 500 Advance spectrometer operating at 500 MHz for ^1H acquisitions and 125 MHz for ^{13}C acquisitions. A Bruker 400 Nanobay spectrometer was operating at 400 MHz, 100 MHz, and 376 MHz for ^1H, ^{13}C, and ^{19}F acquisitions, respectively. Chemical shifts were referenced to the residual proton solvent peaks (^1H: $CDCl_3$, δ 7.26; CD_3CN, δ 1.94) and ^{13}C solvent signals ($CDCl_3$, δ 77.16; CD_3CN, δ 118.26).[2] Signals are listed in ppm, and multiplicity identified as s = singlet, br = broad, d = doublet, t = triplet, q = quartet, m = multiplet; coupling constants in Hz; integration.

UV-Vis Absorptions were measured on a Cary 100 UV-Vis spectrophotometer from Agilent Technologies. Emission intensities were recorded using a Perkin Elmer LS50B Luminescence spectrometer. High-resolution mass spectra were performed at Mass Spectrometry Services at Stony Brook University and were obtained using an Agilent LC-UV-TOF mass spectrometer. Optical rotations were measured on Anton Paar MCP 100Polarimeters. Melting points were measured on Thomas Hoover Uni-Melt Capillary Melting Point Apparatus. Concentration under reduced pressure was performed by rotary evaporation at 25–30 °C at the appropriate pressure. Purified compounds were further dried under a high vacuum (0.01–0.05 Torr). Yields refer to purified and spectroscopically pure compounds.

The blue light-emitting diodes used for the quantum yield measurements: 30 W Blue LEDs (LEDs, 30 W Royal Blue 455 nm, chip size = 45.0 x 45.0 mm) and the heat sink (diameter: 90.0 mm) were purchased from Babaoshop on eBay (https://www.ebay.com/usr/babaoshop).

Abbreviations: DCM = dichloromethane; THF = tetrahydrofuran; MsCl = methanesulfonyl chloride; DIAD = Diisopropyl azodicarboxylate; DMAP = 4-dimethylaminopyridine; DCC = *N,N'*-Dicyclohexylcarbodiim - ide; EDCI·HCl = *N*-(3-Dimethylaminopropyl)-*N'*-ethylcarbodiimide hydrochloride; DIPEA = *N,N*-Diisopropylethylamine.

Photoredox Reaction Setup

LED Light: 12 W and 24 W PAR38 Blue LED flood lamps from ABi® LED lighting company.

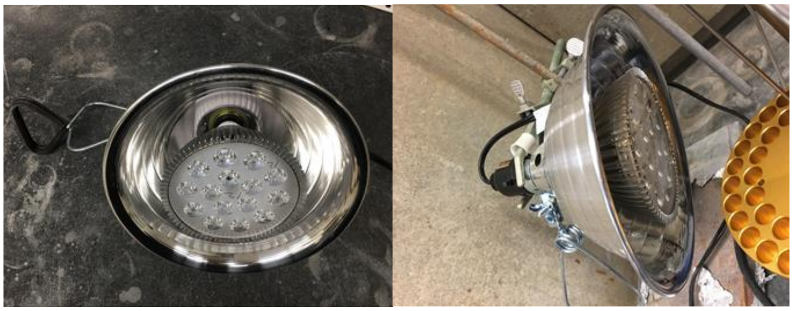

LED Light

The reaction set up: An oil bath was preheated to 90 °C, then the 150 mL pressure vessel was placed into it. A 12 W and 24 W PAR38 Blue LED flood lamps from ABi were placed at a 45° angle to face the pressure vessel (shown in the picture below). The distance between the blue LED lamps and the pressure vessel was 8.00 cm.

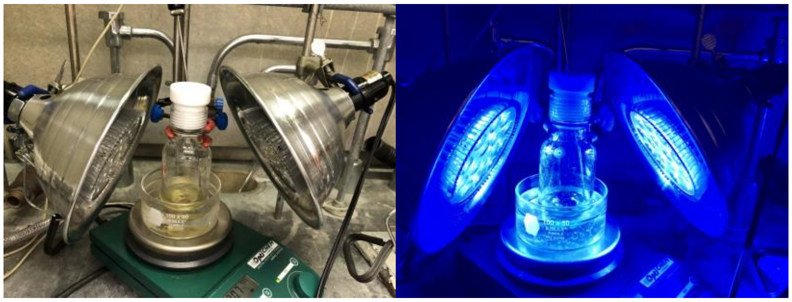

Reaction set up

Experimental Data

General Procedure A (for the synthesis of silyl enol ethers):

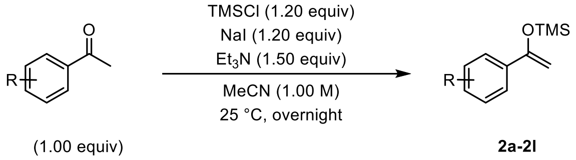

The silyl enol ethers were synthesized according to the literature procedure.[3] An oven-dried round-bottom flask containing acetophenone (1.00 equiv) and pre-dried sodium iodide (1.20 equiv) was evacuated and backfilled with nitrogen thrice. To it was added dry MeCN (1.00 M) and it was allowed to stir for 5 mins. Subsequently, triethyl amine (1.50 equiv) was added to this reaction mixture followed by dropwise addition of chlorotrimethylsilane (1.20 equiv). The resulting mixture was allowed to stir at room temperature for overnight. Upon completion, monitored by GC-MS, it was quenched with a mixture of hexanes (50 mL) and satd. NH_4Cl (50 mL) at 0 °C. The organic phase was collected, and the aqueous phase was extracted with hexanes twice (2 x 50 mL). The combined organic layers were dried over $MgSO_4$ and filtered. The filtrate was concentrated under reduced pressure and the residue was purified by short path distillation under reduced pressure.

Trimethyl((1-phenylvinyl)oxy)silane (2a)

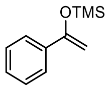

2a

The reaction was performed according to the General Procedure A using acetophenone (0.45 g, 3.75 mmol, 1.00 equiv) as the substrate. After work up, the reaction mixture was purified by short path distillation under reduced pressure to afford the title compound (0.685 g, 3.56 mmol, 95%) as a colorless liquid. **^{1}H NMR** (400 MHz, $CDCl_3$, 25 ºC, δ): 7.62 – 7.60 (m, 2H), 7.36 – 7.28 (m, 3H), 4.93 (d, J = 1.6 Hz, 1H), 4.45 (d, J = 1.6 Hz, 1H), 0.29 (s, 1H). **^{13}C NMR** (100 MHz, $CDCl_3$, 25 ºC, δ): 155.63, 137.49, 128.18, 128.04, 125.19, 91.05, 0.07. The spectroscopic data corresponds to previously reported data.[3]

((1-([1,1'-biphenyl]-4-yl)vinyl)oxy)trimethylsilane (2b)

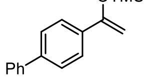

2b

The reaction was performed according to the General Procedure A using 1-([1,1'-biphenyl]-4-yl)ethan-1-one (0.735 g, 3.75 mmol, 1.00 equiv) as the substrate. After work up, the reaction mixture was purified by short path distillation under reduced pressure to afford the title compound (0.846 g, 3.15 mmol, 84%) as a colorless

liquid. **^{1}H NMR** (400 MHz, $CDCl_3$, 25 ºC, δ): 7.69 (d, *J* = 8.4 Hz, 2H), 7.63 (d, *J* = 7.4 Hz, 2H), 7.58 (d, *J* = 8.4 Hz, 2H), 7.46 (t, *J* = 7.6 Hz, 2H), 7.36 (t, *J* = 7.3 Hz, 1H), 4.99 (d, *J* = 1.6 Hz, 1H), 4.48 (d, *J* = 1.5 Hz, 1H), 0.32 (s, 9H). **^{13}C NMR** (100 MHz, $CDCl_3$, 25 ºC, δ): 155.34 (s), 140.93 (s), 140.69 (s), 136.46 (s), 128.76 (s), 127.33 (s), 126.99 (s), 126.77 (s), 125.62 (s), 91.12 (s), 0.11 (s). The spectroscopic data corresponds to previously reported data.[3]

((1-(4-chlorophenyl)vinyl)oxy)trimethylsilane (2c)

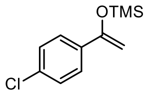

2c

The reaction was performed according to the General Procedure A using 1-(4-chlorophenyl)ethan-1-one (0.580 g, 3.75 mmol, 1.00 equiv) as the substrate. After work up, the reaction mixture was purified by short path distillation under reduced pressure to afford the title compound (0.748 g, 3.30 mmol, 88%) as a colorless liquid. **^{1}H NMR** (400 MHz, $CDCl_3$, 25 ºC, δ): 7.52 (d, *J* = 8.6 Hz, 2H), 7.29 (d, *J* = 8.6 Hz, 2H), 4.89 (d, *J* = 1.8 Hz, 1H), 4.44 (d, *J* = 1.8 Hz, 1H), 0.27 (s, 9H). **^{13}C NMR** (100 MHz, $CDCl_3$, 25 ºC, δ): 154.64 (s), 136.02 (s), 133.98 (s), 128.20 (s), 126.51 (s), 91.37 (s), 0.03 (s). The spectroscopic data corresponds to previously reported data.[3]

((1-(4-fluorophenyl)vinyl)oxy)trimethylsilane (2d)

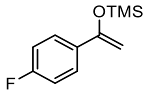

2d

The reaction was performed according to the General Procedure A using 1-(4-fluorophenyl)ethan-1-one (0.518 g, 3.75 mmol, 1.00 equiv) as the substrate. After work up, the reaction mixture was purified by short path distillation under reduced pressure to afford the title compound (0.592 g, 2.81 mmol, 75%) as a colorless liquid. **^{1}H NMR** (400 MHz, $CDCl_3$, 25 ºC, δ): 7.56 (dd, *J* = 8.8, 5.5 Hz, 2H), 7.00 (t, *J* = 8.7 Hz, 2H), 4.84 (d, *J* = 1.7 Hz, 1H), 4.40 (d, *J* = 1.6 Hz, 1H), 0.27 (s, 9H). **^{13}C NMR** (100 MHz, $CDCl_3$, 25 ºC, δ): 162.81 (d, *J* = 247.2 Hz), 154.79, 133.67 (d, *J* = 3.2 Hz), 126.97 (d, *J* = 8.2 Hz), 114.88 (d, *J* = 21.6 Hz), 90.63 (d, *J* = 1.5 Hz), 0.03. **^{19}F NMR** (375 MHz, $CDCl_3$, 25 ºC, δ): -114.05. The spectroscopic data corresponds to previously reported data.[3]

((1-(3-methoxyphenyl)vinyl)oxy)trimethylsilane (2f)

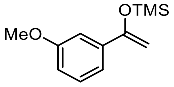

2f

The reaction was performed according to the General Procedure A using 1-(3-methoxyphenyl)ethan-1-one (0.562 g, 3.75 mmol, 1.00 equiv) as the substrate. After work up, the reaction mixture was purified by short path distillation under reduced pressure to afford the title compound (0.792 g, 2.81 mmol, 75%) as a colorless liquid. **^{1}H NMR** (700 MHz, $CDCl_3$, 25 ºC, δ): 7.25 (t, *J* = 7.9 Hz, 1H), 7.21 (d, *J* = 7.8 Hz, 1H), 7.16 (s, 1H), 6.86 (d, *J* = 8.1 Hz, 1H), 4.94 (s, 1H), 4.46 (s, 1H), 3.83 (s, 3H), 0.29 (s, 9H). **^{13}C NMR** (175 MHz, $CDCl_3$, 25 ºC, δ): 159.42 (s), 155.37 (s), 139.02 (s), 129.00 (s), 117.77 (s), 113.59 (s), 110.93 (s), 91.40 (s), 55.14 (s), 0.04 (s). The spectroscopic data corresponds to previously reported data.[3]

((1-(4-methoxy-3-(trifluoromethyl)phenyl)vinyl)oxy)trimethylsilane (2g)

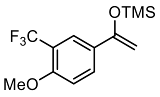

2g

The reaction was performed according to the General Procedure A using 1-(4-methoxy-3-(trifluoromethyl)phenyl)ethan-1-one (0.818 g, 3.75 mmol, 1.00 equiv) as the substrate. After work up, the reaction mixture was purified by short path distillation under reduced pressure to afford the title compound (0.653 g, 2.25 mmol, 60%) as a colorless liquid. **^{1}H NMR** (400 MHz, $CDCl_3$, 25 ºC, δ): 7.79 (d, *J* = 2.2 Hz, 1H), 7.71 (dd, *J* = 8.7, 2.2 Hz, 1H), 6.95 (d, *J* = 8.7 Hz, 1H), 4.84 (d, *J* = 2.0 Hz, 1H), 4.40 (d, *J* = 2.0 Hz, 1H), 3.91 (s, 3H), 0.27 (s, 9H). **^{13}C NMR** (125 MHz, $CDCl_3$, 25 ºC, δ): 157.35, 154.21, 129.89, 129.78, 124.15 (q, *J* = 5.4 Hz), 123.60 (q, *J* = 270.9 Hz), 118.31 (q, *J* = 30.6 Hz), 111.54, 90.30, 56.00, 0.01. **^{19}F NMR** (375 MHz, $CDCl_3$, 25 ºC, δ): –62.54. **HRMS** (ESI-TOF) *m/z* calcd for $C_{13}H_{17}F_3O_2Si$ [(M + H)$^+$], 291.1028, found, 291.1024.

((1-(6-(tert-butyl)-1,1-dimethyl-2,3-dihydro-1H-inden-4-yl)vinyl)oxy)trimethylsilane (2h)

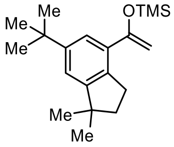

2h

The reaction was performed according to the General Procedure A using 1-(naphthalen-2-yl)ethan-1-one (0.638 g, 3.75 mmol, 1.00 equiv) as the substrate. After work up, the reaction mixture was purified by short path distillation under reduced pressure to afford the title compound (0.763 g, 3.15 mmol, 84%) as a colorless liquid. **^{1}H NMR** (400 MHz, $CDCl_3$, 25 ºC, δ): 7.36 (d, *J* = 1.6 Hz, 1H), 7.11 (d, *J* = 1.3 Hz, 1H), 4.58 (s, 1H), 4.52 (s, 1H), 2.95 (t, *J* = 7.1 Hz, 2H), 1.90 (t, *J* = 7.1 Hz, 2H), 1.33 (s, 9H), 1.26 (s, 6H), 0.25 (s, 9H).**^{13}C NMR** (100 MHz, $CDCl_3$, 25 ºC, δ): 156.86, 153.00, 149.41, 137.25, 134.09, 122.24, 118.76, 93.64, 43.70, 41.61, 34.64, 31.56, 30.31, 28.66, 0.12. **HRMS** (ESI-TOF) *m/z* calcd for $C_{20}H_{32}OSi$ [(M + H)$^+$], 317.2301, found, 317.2295.

((1-(naphthalen-2-yl)vinyl)oxy)trimethylsilane (2j)

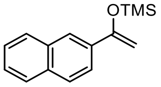

2j

The reaction was performed according to the General Procedure A using 1-(naphthalen-2-yl)ethan-1-one (0.638 g, 3.75 mmol, 1.00 equiv) as the substrate. After work up, the reaction mixture was purified by short path distillation under reduced pressure to afford the title compound (0.763 g, 3.15 mmol, 84%) as a colorless liquid. **^{1}H NMR** (400 MHz, $CDCl_3$, 25 ºC, δ): 8.08 (s, 1H), 7.89 – 7.78 (m, 3H), 7.72 (dd, *J* = 8.7, 1.4 Hz, 1H), 7.51 – 7.45 (m, 2H), 5.09 (d, *J* = 1.4 Hz, 1H), 4.57 (d, *J* = 1.3 Hz, 1H), 0.34 (s, 9H). **^{13}C NMR** (100 MHz, $CDCl_3$, 25 ºC, δ): 155.57, 134.74, 133.24, 133.16, 128.49, 127.61, 127.50, 126.10, 126.08, 124.24, 123.33, 91.91, 0.14. The spectroscopic data corresponds to previously reported data.[3]

2-methoxy-5-(1-((trimethylsilyl)oxy)vinyl)pyridine (2k)

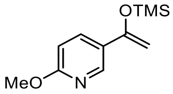

2k

The reaction was performed according to the General Procedure A using 1-(6-methoxypyridin-3-yl)ethan-1-one (0.567 g, 3.75 mmol, 1.00 equiv) as the substrate. After work up, the reaction mixture was purified by short path distillation under reduced pressure to afford the title compound (0.645 g, 2.88 mmol, 77%) as a colorless liquid. **^{1}H NMR** (400 MHz, $CDCl_3$, 25 ºC, δ): 8.40 (d, *J* = 2.2 Hz, 1H), 7.75 (dd, *J* = 8.7, 2.5 Hz, 1H), 6.69 (dd, *J* = 8.7, 0.5 Hz, 1H), 4.79 (d, *J* = 2.0 Hz, 1H), 4.37 (d, *J* = 1.9 Hz, 1H), 3.94 (s, 3H), 0.26 (s, 9H). **^{13}C NMR** (100 MHz, $CDCl_3$, 25 ºC, δ): 164.02, 153.45, 144.06, 135.72, 126.59, 110.14, 90.22, 53.49, 0.03. **HRMS** (ESI-TOF) *m/z* calcd for $C_{11}H_{17}NO_2Si$ [$(M + H)^+$], 224.1107, found, 224.1100.

((1-(thiophen-2-yl)vinyl)oxy)trimethylsilane (2l)

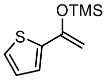

2l

The reaction was performed according to the General Procedure A using 1-(thiophen-2-yl)ethan-1-one (0.473 g, 3.75 mmol, 1.00 equiv) as the substrate. After work up, the reaction mixture was purified by short path distillation under reduced pressure to afford the title compound (0.632 g, 3.19 mmol, 85%) as a colorless liquid. **^{1}H NMR** (400 MHz, $CDCl_3$, 25 ºC, δ): 7.19–7.17 (m, 2H), 6.96 (dd, *J* = 5.0, 3.7 Hz, 1H), 4.81 (d, *J* = 1.9 Hz, 1H), 4.33 (d, *J* = 1.9 Hz, 1H), 0.28 (s, 9H). **^{13}C NMR** (100 MHz, $CDCl_3$, 25 ºC, δ): 150.89, 142.68, 127.24, 124.99, 123.85, 90.25, 0.02. The spectroscopic data corresponds to previously reported data.[4]

trimethyl(pent-1-en-2-yloxy)silane (2n)

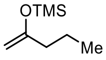

2n

The title compound was prepared according to the literature procedure. **^{1}H NMR** (500 MHz, $CDCl_3$, 25 ºC, δ): 4.04 (s, 2H), 2.01 – 1.95 (m, 2H), 1.52 – 1.42 (m, 2H), 0.91 (t, *J* = 7.4 Hz, 3H), 0.20 (s, 9H). **^{13}C NMR** (125 MHz, $CDCl_3$, 25 ºC, δ): 159.55, 90.08, 38.73, 20.17, 13.74, 0.29. The spectroscopic data corresponds to previously reported data.[5]

((3,3-dimethylbut-1-en-2-yl)oxy)trimethylsilane (2o)

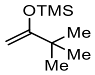

2o

The title compound was prepared according to the literature procedure.[3] **^{1}H NMR** (500 MHz, $CDCl_3$, 25 ºC, δ): 4.09 (d, *J* = 1.0 Hz, 1H), 3.93 (d, *J* = 0.7 Hz, 1H), 1.05 (s, 9H), 0.21 (s, 9H). **^{13}C NMR** (125 MHz, $CDCl_3$, 25 ºC, δ): 167.37, 85.94, 36.57, 28.23, 0.31. The spectroscopic data corresponds to previously reported data.[3]

((1-(tert-butoxy)vinyl)oxy)trimethylsilane (2q)

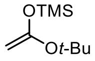

2q

The title compound was prepared according to the literature procedure.[6] **^{1}H NMR** (500 MHz, $CDCl_3$, 25 ºC, δ): 3.43 (d, *J* = 1.4 Hz, 1H), 3.41 (d, *J* = 1.4 Hz, 1H), 1.34 (s, 9H), 0.22 (s, 9H). **^{13}C NMR** (125 MHz, $CDCl_3$, 25 ºC, δ): 157.67, 78.21, 71.85, 28.65, 0.04. The spectroscopic data corresponds to previously reported data.[6]

9-(1-((trimethylsilyl)oxy)vinyl)-9H-carbazole (2r)

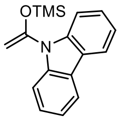

2r

The title compound was prepared according to the literature procedure.[7] **^{1}H NMR** (500 MHz, $CDCl_3$, 25 ºC, δ): 8.06 (d, *J* = 7.7 Hz, 2H), 7.69 (d, *J* = 8.3 Hz, 2H), 7.47 – 7.44 (m, 2H), 7.27 (d, *J* = 8.2 Hz, 2H), 4.68 (d, *J* = 1.6 Hz, 1H), 4.55 (d, *J* = 1.6 Hz, 1H), 0.15 (s, 9H). **^{13}C NMR** (125 MHz, $CDCl_3$, 25 ºC, δ): 146.74, 139.60, 126.09, 123.81, 120.33, 120.13, 111.73, 88.07, -0.25. The spectroscopic data corresponds to previously reported data.[7]

((1-cyclopropylvinyl)oxy)trimethylsilane (2s)

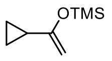

2s

The reaction was performed according to the General Procedure A using 1-cyclopropylethan-1-one (0.315 g, 3.75 mmol, 1.00 equiv) as the substrate. After work up, the reaction mixture was purified by short path distillation under reduced pressure to afford the title compound (0.468 g, 3.00 mmol, 80%) as a colorless liquid. ^{1}**H NMR** (400 MHz, $CDCl_3$, 25 ºC, δ): 4.13 (d, *J* = 0.9 Hz, 1H), 4.00 (d, *J* = 0.7 Hz, 1H), 1.40 (ddd, *J* = 10.1, 8.1, 5.1 Hz, 1H), 0.62 – 0.56 (m, 2H), 0.53 (ddd, *J* = 10.3, 5.1, 2.4 Hz, 2H), 0.19 (s, 9H). ^{13}**C NMR** (100 MHz, $CDCl_3$, 25 ºC, δ): 159.11, 87.62, 15.49, 4.45, 0.04. The spectroscopic data corresponds to previously reported data.[8]

General Procedure B (for the synthesis of 1-bromosugars):

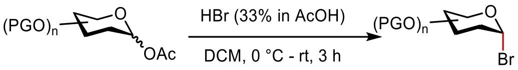

1a-1z

The C1 acetyl protected sugar (1.00 equiv) was dissolved in dry DCM (0.500 M) and cooled to 0 °C. HBr (33% Wt in AcOH, 2.00 equiv) was added, and the reaction mixture was slowly warmed to room temp over 10 min. After stirring at room temperature for 3 h, the reaction mixture was poured onto an ice/water mixture. The organic phase was collected, and the aqueous phase was extracted with DCM twice. The combined organic layers were washed with satd. $NaHCO_3$, brine, dried over Mg_2SO_4, and filtered. The filtrate was concentrated *in vacuo* and the residue was purified by flash column chromatography on silica gel to afford the desired compound.

2,3,4,6-Tetra-*O*-acetyl-α-D-glucopyranosyl bromide (1a)

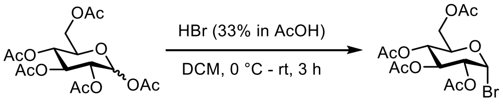

S1 **1a**

The reaction was performed according to the General Procedure B using **S1** (2.00 g, 5.13 mmol, 1.00 equiv) as the substrate. After work up, the reaction mixture was purified by flash column chromatography on silica gel, eluting with Hexanes: EtOAc [3:1 (v/v)] to afford the title compound (1.85 g, 4.50 mmol, 88%) as a white solid. $\mathbf{R}_f$ = 0.65 [Hexanes: EtOAc 2:1 (v/v)]. ^{1}**H NMR** (500 MHz, $CDCl_3$, 25 ºC, δ): 6.58 (d, *J* = 4.0 Hz, 1H), 5.52 (t, *J* = 9.7 Hz, 1H), 5.13 (t, *J* = 9.8 Hz, 1H), 4.81 (dd, *J* = 10.0, 4.0 Hz, 1H), 4.33 – 4.21 (m, 2H), 4.13 – 4.06 (m, 1H), 2.07 (s, 3H), 2.06 (s, 3H), 2.02 (s, 3H), 2.00 (s, 3H). ^{13}**C NMR** (125 MHz, $CDCl_3$, 25 ºC, δ): 170.52, 169.87, 169.82, 169.50, 86.66, 72.20, 70.64, 70.21, 67.21, 61.00, 20.72, 20.70, 20.67, 20.60. The spectroscopic data corresponds to previously reported data.[9]

2,3,4,6-Tetra-*O*-acetyl-α-D-galactopyranosyl bromide (1b)

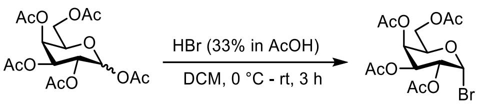

S2 1b

The reaction was performed according to the General Procedure B using **S2** (2.00 g, 5.13 mmol, 1.00 equiv) as the substrate. After work up, the reaction mixture was purified by flash column chromatography on silica gel, eluting with Hexanes: EtOAc [3:1 (v/v)] to afford the title compound (1.73 g, 4.21 mmol, 82%) as a white solid. **R$_f$** = 0.65 [Hexanes: EtOAc 2:1 (v/v)]. **^{1}H NMR** (500 MHz, $CDCl_3$, 25 ºC, δ): 6.69 (d, *J* = 3.9 Hz, 1H), 5.57 – 5.48 (m, 1H), 5.40 (dd, *J* = 10.6, 3.3 Hz, 1H), 5.04 (dd, *J* = 10.6, 4.0 Hz, 1H), 4.48 (t, *J* = 6.6 Hz, 1H), 4.18 (dd, *J* = 11.4, 6.4 Hz, 1H), 4.11 (dd, *J* = 11.4, 6.8 Hz, 1H), 2.15 (s, 3H), 2.11 (s, 3H), 2.06 (s, 3H), 2.01 (s, 3H). **^{13}C NMR** (125 MHz, $CDCl_3$, 25 ºC, δ): 170.45, 170.20, 170.02, 169.89, 88.25, 71.19, 68.12, 67.90, 67.11, 60.96, 20.88, 20.77, 20.72, 20.69. The spectroscopic data corresponds to previously reported data.[9]

2,3,4-Tri-*O*-acetyl-6-*O*-benzyl-α-D-galactopyranosyl bromide (1c)

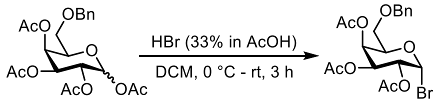

S3 1c

The reaction was performed according to the General Procedure B using **S3** (635 mg, 1.45 mmol, 1.00 equiv) as the substrate. After work up, the reaction mixture was purified by flash column chromatography on silica gel, eluting with Hexanes: EtOAc [4:1 (v/v)] to afford the title compound (315 mg, 0.78 mmol 47%) as a white solid. **R$_f$** = 0.50 [Hexanes: EtOAc 4:1 (v/v)]. **^{1}H NMR** (700 MHz, $CDCl_3$, 25 ºC, δ): 7.34 (t, *J* = 7.3 Hz, 2H), 7.28 (m, 3H), 6.70 (d, *J* = 3.9 Hz, 1H), 5.57 (d, *J* = 2.7 Hz, 1H), 5.40 (dd, *J* = 10.6, 2.7 Hz, 1H), 5.03 (dd, *J* = 10.6, 3.9 Hz, 1H), 4.55 (d, *J* = 12.0 Hz, 1H), 4.45 (t, *J* = 6.0 Hz, 1H), 4.42 (d, *J* = 12.0 Hz, 1H), 3.54 (dd, *J* = 9.8, 6.0 Hz, 1H), 3.48 (dd, *J* = 9.8, 6.0 Hz, 1H), 2.10 (s, 3H), 2.04 (s, 3H), 2.00 (s, 3H). **^{13}C NMR** (175 MHz, $CDCl_3$, 25 ºC, δ): 170.23, 169.95, 169.83, 137.37, 128.60, 128.60, 128.04, 128.04, 128.04, 88.77, 73.57, 72.17, 68.29, 68.08, 67.50, 66.84, 20.88, 20.73, 20.65. The spectroscopic data corresponds to previously reported data.[10]

2,3,4-Tri-*O*-acetyl-6-*O*-[(1,1-dimethylethyl)diphenylsilyl]-α-D-glucopyranosyl bromide (1d)

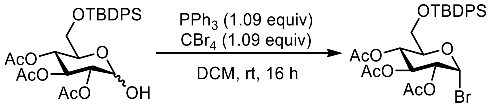

S4 1d

To a solution of the **S4** (571 mg, 1.05 mmol, 1.00 equiv) in dry DCM (2.00 mL) was added triphenylphosphine (301 mg, 1.15 mmol, 1.09 equiv) and tetrabromomethane (383 mg, 1.15 mmol, 1.09 equiv). The reaction mixture was stirred under nitrogen at room temperature for 16 h. Saturated NaHCO3 was added until the pH of the solution became neutral. The organic layer was collected, washed with brine, dried with solid anhydrous Mg_2SO_4 and filtered. The filtrate was concentrated *in vacuo* and the residue was purified by flash column chromatography on silica gel, eluting with Hexanes: EtOAc [8:1 (v/v)] to afford

the title compound (171 mg, 0.28 mmol, 27%) as a white foam. **R*f*** = 0.70 [Hexanes: EtOAc 4:1 (v/v)]. **^{1}H NMR** (700 MHz, $CDCl_3$, 25 ºC, δ): 7.63 (dd, *J* = 16.1, 7.0 Hz, 4H), 7.34-7.46 (m, 6H), 6.67 (d, *J* = 3.5 Hz, 1H), 5.54 (t, *J* = 9.8 Hz, 1H), 5.35 (t, *J* = 9.8 Hz, 1H), 4.82 (dd, *J* = 10.5, 4.2 Hz, 1H), 4.13 (d, *J* = 10.5 Hz, 1H), 3.76 (dd, *J* = 11.9, 1.4 Hz, 1H), 3.72 (dd, *J* = 11.9, 4.2 Hz, 1H), 2.11 (s, 3H), 2.04 (s, 3H), 1.93 (s, 3H), 1.05 (s, 9H). **^{13}C NMR** (175 MHz, $CDCl_3$, 25 ºC, δ): 170.28, 170.04, 169.39, 135.81, 135.81, 135.79, 135.79, 133.00, 132.92, 129.69, 129.93, 127.88, 127.88, 127.88, 127.88 87.68, 74.85, 70.97, 70.82, 67.40, 61.54, 26.85, 26.85, 26.85, 20.86, 20.86, 20.67, 19.35. The spectroscopic data corresponds to previously reported data.[11]

2,3-Di-*O*-acetyl-4,6-*O*-[(*R*)-(4-methoxyphenyl)methylene]-α-D-glucopyranosyl bromide (1e)

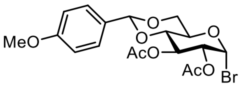

1e

The title compound was prepared according to the literature procedure.[12] **^{1}H NMR** (400 MHz, $CDCl_3$, 25 ºC, δ): 7.37 (d, *J* = 8.6 Hz, 2H), 6.88 (d, *J* = 8.6 Hz, 2H), 6.60 (d, *J* = 4.1 Hz, 1H), 5.65 (t, *J* = 9.8 Hz, 1H), 5.47 (s, 1H), 4.84 (dd, *J* = 9.7, 4.1 Hz, 1H), 4.32 (dd, *J* = 10.2, 4.9 Hz, 1H), 4.23 (td, *J* = 9.8, 5.0 Hz, 1H), 3.86 – 3.63 (m, 5H), 2.11 (s, 3H), 2.07 (s, 3H). **^{13}C NMR** (100 MHz, $CDCl_3$, 25 ºC, δ): 170.15, 169.61, 160.35, 129.12, 127.63, 113.75, 101.87, 87.11, 78.15, 71.52, 68.91, 67.94, 67.09, 55.41, 20.88, 20.81. The spectroscopic data corresponds to previously reported data.[12]

2,3,4-Tri-*O*-acetyl-α-D-xylopyranosyl bromide (1f)

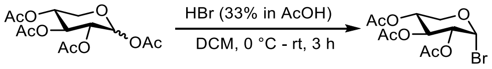

S5 **1f**

The reaction was performed according to the General Procedure B using **S5** (2.20 g, 6.90 mmol, 1.00 equiv) as the substrate. After work up, the reaction mixture was purified by flash column chromatography on silica gel, eluting with Hexanes: EtOAc [3:1 (v/v)] to afford the title compound (1.30 g, 3.85 mmol, 57%) as a white solid. **R*f*** = 0.50 [Hexanes: EtOAc 2:1 (v/v)]. **^{1}H NMR** (700 MHz, $CDCl_3$, 25 ºC, δ) 6.57 (d, *J* = 4.2 Hz, 1H), 5.55 (t, *J* = 9.8 Hz, 1H), 5.03 (td, *J* = 9.8, 6.3 Hz, 1H), 4.76 (dd, *J* = 9.8, 4.2 Hz, 1H), 4.04 (dd, *J* = 11.2, 6.3 Hz, 1H), 3.87 (t, *J* = 11.2 Hz, 1H), 2.09 (s, 3H), 2.05 (s, 3H), 2.05 (s, 3H). **^{13}C NMR** (175 MHz, $CDCl_3$, 25 ºC, δ): 169.98, 169.98, 169.89, 87.70, 70.98, 69.61, 68.20, 62.64, 20.81, 20.80, 20.77. The spectroscopic data corresponds to previously reported data.[13]

2,3,4-Tri-*O*-acetyl-α-L-fucopyranosyl bromide (1g)

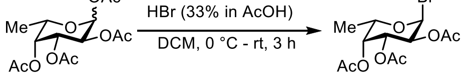

S6 **1g**

The reaction was performed according to the General Procedure B using **S6** (2.02 g, 6.00 mmol, 1.00 equiv) as the substrate. After work up, the reaction mixture was purified by flash column chromatography on silica

gel, eluting with Hexanes: EtOAc [5:1 (v/v)] to afford the title compound (1.66 g, 4.70 mmol, 78%) as a white solid. **R$_f$** = 0.25 [Hexanes: EtOAc 5:1 (v/v)]. **^{1}H NMR** (700 MHz, $CDCl_3$, 25 ºC, δ): 6.68 (d, *J* = 3.5 Hz, 1H), 5.39 (dd, *J* = 10.5, 3.5 Hz, 1H), 5.34 (d, *J* = 3.5 Hz, 1H), 5.01 (dd, *J* = 10.5, 3.5 Hz, 1H), 4.39 (q, *J* = 7.0 Hz, 1H), 2.16 (s, 3H), 2.09 (s, 3H), 1.99 (s, 3H), 1.20 (d, *J* = 7.0 Hz, 3H). **^{13}C NMR** (175 MHz, $CDCl_3$, 25 ºC, δ): 170.37, 170.24, 169.91, 89.40, 70.08, 69.91, 68.51, 67.95, 20.89, 20.73, 20.67, 15.56. The spectroscopic data corresponds to previously reported data.[14]

(2R,3R,4S,5S,6S)-2-bromo-6-(methoxycarbonyl)tetrahydro-2H-pyran-3,4,5-triyl triacetate (1h)

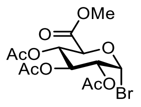

1h

The title compound was prepared according to the literature procedure.[15] **^{1}H NMR** (500 MHz, $CDCl_3$, 25 ºC, δ): 6.63 (d, *J* = 4.0 Hz, 1H), 5.60 (t, *J* = 9.7 Hz, 1H), 5.23 (t, *J* = 9.9 Hz, 1H), 4.84 (dd, *J* = 10.0, 4.1 Hz, 1H), 4.57 (d, *J* = 10.3 Hz, 1H), 3.75 (s, 3H), 2.09 (s, 3H), 2.04 (s, 3H), 2.04 (s, 3H). **^{13}C NMR** (125 MHz, $CDCl_3$, 25 ºC, δ): 169.78, 169.75, 169.57, 166.78, 85.48, 72.15, 70.43, 69.40, 68.60, 53.25, 20.72, 20.57. The spectroscopic data corresponds to previously reported data.[15]

2,3,6,2',3',4',6'-Hepta-*O*-acetyl-α-D-cellobiose bromide (1i)

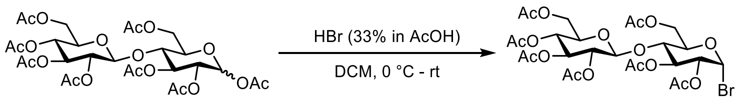

S7 **1i**

The reaction was performed according to the General Procedure B using **S7** (3.49 g, 5.14 mmol, 1.00 equiv) as the substrate. After work up, the reaction mixture was purified by flash column chromatography on silica gel, eluting with Hexanes: EtOAc [2.5:1 (v/v)] to afford the title compound (2.00 g, 2.86 mmol, 56%) as a white solid. **R$_f$** = 0.50 [Hexanes: EtOAc 3:1 (v/v)]. **^{1}H NMR** (500 MHz, $CDCl_3$, 25 ºC, δ): 6.53 (d, *J* = 4.0 Hz, 1H), 5.53 (t, *J* = 9.7 Hz, 1H), 5.15 (t, *J* = 9.3 Hz, 1H), 5.08 (t, *J* = 9.7 Hz, 1H), 4.98 – 4.87 (m, 1H), 4.77 (dd, *J* = 10.0, 4.1 Hz, 1H), 4.54 (t, *J* = 8.6 Hz, 2H), 4.37 (dd, *J* = 12.5, 4.4 Hz, 1H), 4.23 – 4.14 (m, 2H), 4.05 (dd, *J* = 12.5, 2.0 Hz, 1H), 3.84 (t, *J* = 9.7 Hz, 1H), 3.67 (ddd, *J* = 9.9, 4.2, 2.2 Hz, 1H), 2.14 (s, 3H), 2.09 (s, 6H), 2.04 (s, 6H), 2.01 (s, 3H), 1.99 (s, 3H). **^{13}C NMR** (125 MHz, $CDCl_3$, 25 ºC, δ): 170.64, 170.40, 170.24, 170.13, 169.43, 169.12, 100.72, 86.55, 75.38, 73.16, 73.09, 72.20, 71.75, 70.92, 69.57, 67.90, 61.74, 61.07, 20.97, 20.83, 20.74, 20.69. The spectroscopic data corresponds to previously reported data.[16]

2,3,6,2',3',4',6'-Hepta-*O*-acetyl-α-D-maltose bromide (1j)

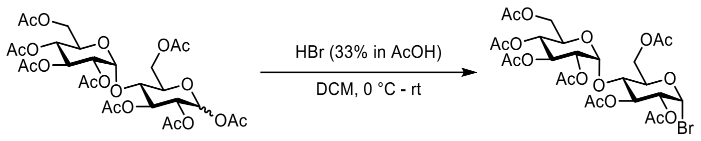

S8 1j

The reaction was performed according to the General Procedure B using **S8** (750 mg, 1.10 mmol, 1.00 equiv) as the substrate. After work up, the reaction mixture was purified by flash column chromatography on silica gel, eluting with Hexanes: EtOAc [2.5:1 (v/v)] to afford the title compound (420 mg, 0.600 mmol, 55%) as a white solid. $\mathbf{R}_f$ = 0.50 [Hexanes: EtOAc 3:1 (v/v)]. **^{1}H NMR** (500 MHz, $CDCl_3$, 25 ºC, δ): 6.45 (d, *J* = 4.0 Hz, 1H), 5.55 (t, *J* = 9.4 Hz, 1H), 5.36 (d, *J* = 4.0 Hz, 1H), 5.31 (t, *J* = 10.0 Hz, 1H), 5.01 (t, *J* = 9.9 Hz, 1H), 4.81 (dd, *J* = 10.5, 4.0 Hz, 1H), 4.66 (dd, *J* = 9.9, 4.0 Hz, 1H), 4.46 (dd, *J* = 13.7, 3.4 Hz, 1H), 4.28 – 4.13 (m, 3H), 4.03 – 3.96 (m, 2H), 3.92 – 3.84 (m, 1H), 2.09 (s, 3H), 2.04 (s, 3H), 2.02 (s, 3H), 2.01 (s, 3H), 1.98 (s, 3H), 1.97 (s, 3H), 1.95 (s, 3H). **^{13}C NMR** (125 MHz, $CDCl_3$, 25 ºC, δ): 171.07, 170.66, 170.45, 170.25, 169.83, 169.50, 169.41, 95.79, 86.14, 72.57, 72.34, 71.60, 71.01, 70.02, 69.24, 68.65, 67.92, 61.86, 61.35, 60.35, 21.03, 20.86, 20.76, 20.67, 20.63, 20.59. The spectroscopic data corresponds to previously reported data.[16b]

2,3,4,2',3',4',6'-Hepta-*O*-acetyl-α-D-melibiosyl bromide (1k)

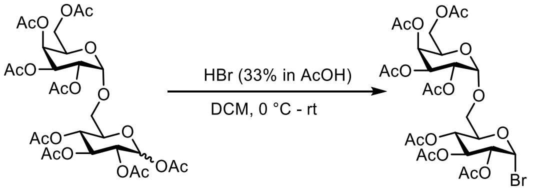

S9 1k

The reaction was performed according to the General Procedure B using **S9** (1.22 g, 1.80 mmol, 1.00 equiv) as the substrate. After work up, the reaction mixture was purified by flash column chromatography on silica gel, eluting with Hexanes: EtOAc [1.5:1 (v/v)] to afford the title compound (320 mg, 0.460 mmol, 25%) as a white solid. $\mathbf{R}_f$ = 0.70 [Hexanes: EtOAc 1:1 (v/v)]. **^{1}H NMR** (700 MHz, $CDCl_3$, 25 ºC, δ): 6.58 (d, *J* = 4.2 Hz, 1H), 5.55 (t, *J* = 9.8 Hz, 1H), 5.46 (d, *J* = 3.5 Hz, 1H), 5.32 (dd, *J* = 10.5, 3.5 Hz, 1H), 5.15-5.18 (m, 2H), 5.08 (dd, *J* = 10.5, 3.5 Hz, 1H), 4.78 (dd, *J* = 9.8, 4.2 Hz, 1H), 4.23 (ddd, *J* = 10.5, 4.2, 2.1 Hz, 1H), 4.16 (t, *J* = 7.0 Hz, 1H), 4.06 (qd, *J* = 11.2, 7.0 Hz, 1H), 3.76 (dd, *J* = 11.9, 4.2 Hz, 1H), 3.62 (dd, *J* = 11.9, 2.1 Hz, 1H), 2.13 (s, 3H), 2.11 (s, 3H), 2.09 (s, 3H), 2.06 (s, 3H), 2.04 (s, 3H), 2.03 (s, 3H), 1.98 (s, 3H). **^{13}C NMR** (175 MHz, $CDCl_3$, 25 ºC, δ): 170.67, 170.50, 170.30, 170.02, 169.98, 169.93, 169.48, 96.37, 86.64, 73.02, 70.72, 70.33, 68.16, 68.06, 67.73, 67.55, 66.56, 65.51, 61.72, 20.93, 20.85, 20.79, 20.79, 20.76, 20.76, 20.72. The spectroscopic data corresponds to previously reported data.[17]

2,3,4,6-Tetra-*O*-benzoyl-α-D-glucopyranosyl bromide (1l)

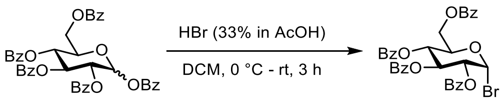

The reaction was performed according to the General Procedure B using **S10** (2.00 g, 2.86 mmol, 1.00 equiv) as the substrate. After work up, the reaction mixture was purified by flash column chromatography on silica gel, eluting with Hexanes: EtOAc [3:1 (v/v)] to afford the title compound (1.51 g, 2.29 mmol, 80%) as a white solid. **R$_f$** = 0.68 [Hexanes: EtOAc 2:1 (v/v)]. **^{1}H NMR** (500 MHz, $CDCl_3$, 25 °C, δ): 8.07 (d, *J* = 7.3 Hz, 2H), 8.01 (d, *J* = 7.3 Hz, 2H), 7.96 (d, *J* = 7.3 Hz, 2H), 7.88 (d, *J* = 7.3 Hz, 2H), 7.60 – 7.50 (m, 3H), 7.47 – 7.35 (m, 7H), 7.31 (t, *J* = 7.8 Hz, 2H), 6.87 (d, *J* = 4.0 Hz, 1H), 6.27 (t, *J* = 9.8 Hz, 1H), 5.83 (t, *J* = 10.0 Hz, 1H), 5.34 (dd, *J* = 10.0, 4.0 Hz, 1H), 4.80 – 4.71 (m, 1H), 4.68 (dd, *J* = 12.5, 2.6 Hz, 1H), 4.52 (dd, *J* = 12.5, 4.5 Hz, 1H). **^{13}C NMR** (125 MHz, $CDCl_3$, 25 °C, δ): 166.16, 165.70, 165.44, 165.23, 133.94, 133.78, 133.49, 133.40, 130.22, 130.07, 129.97, 129.88, 129.59, 128.94, 128.70, 128.66, 128.63, 128.60, 128.50, 87.01, 72.85, 71.61, 70.76, 68.13, 62.08. The spectroscopic data corresponds to previously reported data.[17]

3,4,6-Tri-*O*-Benzoyl-2-*O*-(thiophene-2-carbonyl)-α-D-glucopyranosyl bromide (1m)

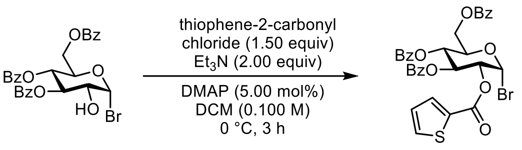

The compound **S11** was prepared according to the literature procedure.[18] To a solution of **S11** (278 mg, 0.500 mmol, 1.00 equiv) in dry DCM (3.00 mL) was added Et_3N (101 mg, 1.00 mmol, 2.00 equiv), Thiophene-2-carbonyl chloride (110 mg, 0.75 mmol, 1.50 equiv) and DMAP (3.05 mg, 0.0250 mmol, 5.00 mol%) at 0 °C. After the reaction mixture was stirred at 0 °C for 3 h, it was quenched with saturated $NaHCO_3$ solution (6.00 mL) and extracted with DCM (2 × 50 mL). The combined organic layers were washed with brine, dried with anhydrous Mg_2SO_4 and filtered. The filtrate was concentrated *in vacuo.* The residue was purified by flash column chromatography on silica gel, eluting with Hexanes: EtOAc [3:1 (v/v)] to afford the title compound (219 mg, 0.329 mmol, 66%) as a white solid. **R$_f$** = 0.60 [Hexanes: EtOAc 2:1 (v/v)]. **^{1}H NMR** (500 MHz, $CDCl_3$, 25 °C, δ): 8.06 (d, *J* = 7.2 Hz, 2H), 7.97 – 7.92 (m, 2H), 7.89 (d, *J* = 7.2 Hz, 2H), 7.80 (dd, *J* = 3.8, 1.2 Hz, 1H), 7.61 – 7.55 (m, 2H), 7.52 (t, *J* = 7.5 Hz, 1H), 7.46 (dd, *J* = 14.4, 6.3 Hz, 3H), 7.37 (t, *J* = 7.8 Hz, 2H), 7.32 (t, *J* = 7.8 Hz, 2H), 7.10 – 7.00 (m, 1H), 6.85 (d, *J* = 4.0 Hz, 1H), 6.22 (t, *J* = 9.8 Hz, 1H), 5.80 (t, *J* = 10.0 Hz, 1H), 5.26 (dd, *J* = 9.9, 4.1 Hz, 1H), 4.75 – 4.68 (m, 1H), 4.66 (dd, *J* = 12.5, 2.5 Hz, 1H), 4.50 (dd, *J* = 12.5, 4.5 Hz, 1H). **^{13}C NMR** (175 MHz, $CDCl_3$, 25 °C, δ): 166.17, 165.60, 165.22, 160.95, 135.13, 134.24, 133.80, 133.49, 133.42, 131.81, 130.09, 129.98, 129.91, 129.58, 128.97, 128.65, 128.61, 128.53, 128.21, 86.83, 72.83, 71.70, 70.66, 68.10, 62.06. **HRMS** (ESI-TOF) *m/z* calcd for $C_{32}H_{26}BrO_9S$ [$(M + H)^+$], 665.0475, found, 665.0477.

(2R,3R,4S,5S,6S)-2-bromo-6-((((1S,2R,5S)-2-isopropyl-5-methylcyclohexyl)oxy)carbonyl)tetrahydro-2H-pyran-3,4,5-triyl triacetate (1n)

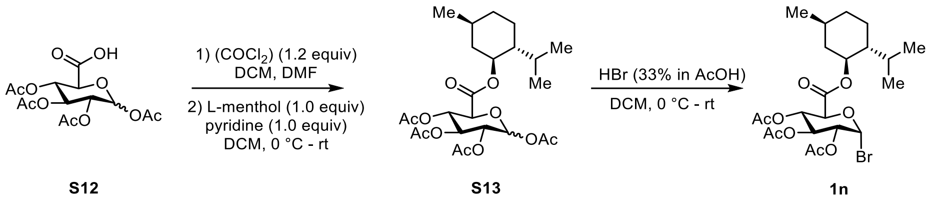

To a solution of **S12** in DCM was added 2 drops of DMF and the solution was cooled to 0 °C. To that solution, oxalyl chloride was added dropwise. The reaction was allowed to run at room temperature for 3-4 h. Then the excess oxalyl chloride was removed under reduced pressure. In another round bottom flask, L-menthol was dissolved in DCM and to it triethylamine was added. It was cooled to 0 °C and the solution of the acid chloride in DCM was added dropwise to it. The reaction was allowed to run overnight after which it was quenched with satd. $NaHCO_3$. The organic layers were collected and washed with brine. The combined organic layers were dried over Na_2SO_4, filtered, and concentrated *in vacuo*. The residue was purified by silica gel column chromatography Hexanes: EtOAc [4:1 (v/v)] to give **S13** (470 mg, 0.94 mmol, 50% yield) as a colorless solid.

1n was synthesized according to the General Procedure B using **S13** (440 mg, 0.700 mmol, 1.00 equiv) as the substrate. After work up, the reaction mixture was purified by flash column chromatography on silica gel, eluting with Hexanes: EtOAc [4:1 (v/v)] to afford the title compound (55.0 mg, 0.110 mmol, 10%) as a white foam. $\mathbf{R}_f$ = 0.42 [Hexanes: EtOAc 4:1 (v/v)]. **^{1}H NMR** (400 MHz, $CDCl_3$, 25 °C, δ): 6.64 (d, *J* = 4.0 Hz, 1H), 5.57 (t, *J* = 9.7 Hz, 1H), 5.29 (dd, *J* = 10.3, 9.5 Hz, 1H), 4.87 (dd, *J* = 10.0, 4.0 Hz, 1H), 4.77 (td, *J* = 10.9, 4.4 Hz, 1H), 4.58 (d, *J* = 10.4 Hz, 1H), 2.10 (s, 3H), 2.06 – 2.03 (m, 6H), 1.98 – 1.92 (m, 1H), 1.81 (dtd, *J* = 13.9, 7.0, 2.7 Hz, 1H), 1.69 (dd, *J* = 14.2, 2.5 Hz, 2H), 1.53 – 1.34 (m, 2H), 1.10 – 0.98 (m, 1H), 0.96 – 0.80 (m, 8H), 0.74 (d, *J* = 6.9 Hz, 3H). **^{13}C NMR** (100 MHz, $CDCl_3$, 25 °C, δ): 169.95, 169.84, 169.18, 165.96, 85.75, 76.73, 72.52, 70.40, 70.03, 68.59, 47.03, 40.55, 34.18, 31.50, 26.17, 23.35, 22.07, 20.90, 20.76, 20.70, 16.22. **HRMS** (ESI-TOF) *m/z* calcd for $C_{22}H_{34}BrO_9$ [(M + H)$^+$], 521.1381, found, 521.1382.

2,3,4-Tri-*O*-acetyl-6-*O*-[(2-(3-cyano-4-isobutoxyphenyl)-4-methylthiazole-5-carbonyl)]-α-D-glucopyranosyl bromide (1o)

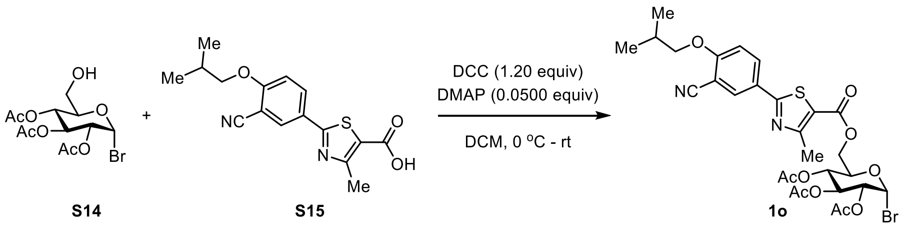

A suspension of febuxostat **S15** (190 mg, 0.600 mmol, 1.20 equiv) and DMAP (3.00 mg, 0.0250 mmol, 5.00 mol%) in DCM (3.00 mL) was added a solution of DCC (124 mg, 0.600 mmol, 1.20 equiv) in DCM (1.00 mL) at 0 °C. After stirring for 10 min at 0 °C, **S14** (185 mg, 0.50 mmol, 1.00 equiv) was added. The reaction mixture was stirred at room temperature for 12 h, quenched with saturated $NaHCO_3$ solution (6.00 mL), and

extracted with DCM (2×30 mL). The organic layer was collected, washed with brine, dried with anhydrous Mg_2SO_4, and filtered. The filtrate was concentrated *in vacuo.* The residue was purified by flash column chromatography on silica gel, eluting with Hexanes: EtOAc [2:1 (v/v)] to afford the title compound (214 mg, 0.320 mmol, 64%) as a white solid. **R*f*** = 0.30 [Hexanes: EtOAc 2:1 (v/v)]. **^{1}H NMR** (500 MHz, $CDCl_3$, 25 ºC, δ): 8.20 (d, *J* = 2.2 Hz, 1H), 8.11 (dd, *J* = 8.8, 2.3 Hz, 1H), 7.00 (d, *J* = 8.9 Hz, 1H), 6.63 (d, *J* = 4.0 Hz, 1H), 5.59 (t, *J* = 9.7 Hz, 1H), 5.20 (t, *J* = 9.8 Hz, 1H), 4.85 (dd, *J* = 10.0, 4.1 Hz, 1H), 4.44 (d, *J* = 4.0 Hz, 2H), 4.40 (dd, *J* = 10.3, 2.7 Hz, 1H), 3.90 (d, *J* = 6.5 Hz, 2H), 2.75 (s, 3H), 2.26 – 2.13 (m, 1H), 2.10 (s, 3H), 2.07 (s, 3H), 2.04 (s, 3H), 1.08 (d, *J* = 6.7 Hz, 6H). **^{13}C NMR** (125 MHz, $CDCl_3$, 25 ºC, δ): 169.96, 169.94, 169.54, 168.01, 162.71, 162.32, 161.45, 132.83, 132.33, 126.00, 120.80, 115.48, 112.71, 103.13, 86.57, 75.81, 72.23, 70.75, 70.23, 67.46, 61.72, 28.28, 20.77, 20.75, 20.70, 19.17, 17.71. **HRMS** (ESI-TOF) *m/z* calcd for $C_{28}H_{32}BrN_2O_{10}S$ [(M + H)$^+$], 667.0956, found, 667.0966.

2,3,4-Tri-*O*-acetyl-6-*O*-[((4aS,6aS,6bR,8aR,10S,12aR,12bR,14bS)-10-acetoxy-2,2,6a,6b,9,9,12a-heptamethyl-1,2,3,4,4a,5,6,6a,6b,7,8,8a,9,10,11,12,12a,12b,13,14b-icosahydropicene-4a-carbonyl)]-α-D-glucopyranosyl bromide (1p)

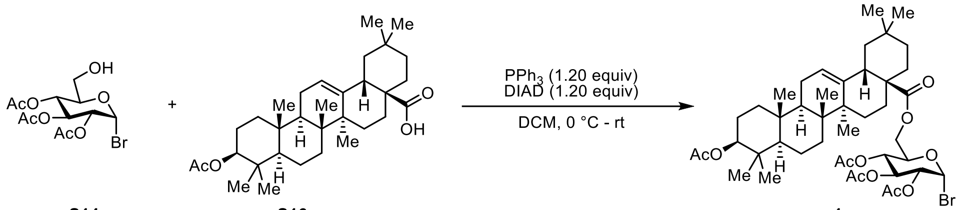

S14 **S16** **1p**

To a solution of **S14** (147 mg, 0.400 mmol, 1.00 equiv) and **S16** (239 mg, 0.480 mmol, 1.20 equiv) in dry DCM (8.00 ml, M = 0.0500) was added triphenylphosphine (126 mg, 0.480 mmol, 1.20 equiv) at 0 ºC. DIAD (94.5 ul, 0.480 mmol, 1.20 equiv) was then added dropwise to the resulting mixture. After the reaction mixture was stirred at room temperature for 12 h, the solvent was removed under vacuum. The residue was purified by flash column chromatography on silica gel, eluting with Hexanes: EtOAc [9:1 (v/v)] to afford the title compound as an off-white solid (129 mg, 0.152 mmol, 38% yield). **R*f*** = 0.45 [Hexanes: EtOAc 2:1 (v/v)]. **^{1}H NMR** (700 MHz, $CDCl_3$, 25 ℃, δ): 6.59 (d, *J* = 3.5 Hz, 1H), 5.54 (t, *J* = 9.8 Hz, 1H), 5.29 (t, *J* = 3.5 Hz, 1H), 5.12 (t, *J* = 9.8 Hz, 1H), 4.77 (dd, *J* = 9.8, 4.2 Hz, 1H), 4.47-4.50 (m, 1H), 4.30 (dd, *J* = 12.6, 2.1 Hz, 1H), 4.27 (ddd, *J* = 10.5, 4.2, 1.4 Hz, 1H), 4.04 (dd, *J* = 12.6, 4.9 Hz, 1H), 2.83 (dd, *J* = 14.0, 4.2 Hz, 1H), 2.10 (s, 3H), 2.05 (s, 3H), 2.04 (s, 3H), 2.03 (s, 3H), 1.99 (td, *J* = 14.0, 4.2 Hz, 1H), 1.82-1.92 (m, 2H), 1.50-1.71 (m, 11H), 1.45 (td, *J* = 12.6, 4.2 Hz, 1H), 1.37 (td, *J* = 12.6, 2.8 Hz, 1H), 1.33 (td, *J* = 14.0, 4.2 Hz, 1H), 1.23-1.30 (m, 1H), 1.94-1.21 (m, 1H), 1.14-1.17 (m, 1H), 1.12 (s, 3H), 1.09 (dt, *J* = 14.0, 2.8 Hz, 1H), 1.01-1.06 (m, 1H), 0.93 (s, 3H), 0.92 (s, 3H), 0.89 (s, 3H), 0.86 (s, 3H), 0.85 (s, 3H), 0.83 (d, *J* = 11.2 Hz, 1H), 0.71 (s, 3H). **^{13}C NMR** (175 MHz, $CDCl_3$, 25 ℃, δ): 177.15, 171.18, 170.05, 169.96, 169.41, 143.54, 122.70, 86.67, 81.07, 72.54, 70.82, 70.36, 67.63, 60.84, 55.43, 47.68, 47.06, 45.96, 41.81, 41.36, 39.41, 38.24, 37.82, 37.06, 33.98, 33.20, 32.79, 32.33, 30.80, 28.18, 27.75, 25.91, 23.69, 23.66, 23.54, 23.21, 21.47, 20.81, 20.79, 20.72, 18.36, 17.06, 16.82, 15.53. **HRMS** (ESI-TOF) *m/z* calcd for $C_{44}H_{66}BrO_{11}$ [(M + H)$^+$], 849.3783, found, 849.3776.

2,3,4-Tri-*O*-acetyl-6-*O*-[(2-(4-(2-(4-chlorobenzamido)ethyl)phenoxy)-2-methylpropanoyl)]-α-D-glucopyranosyl bromide (1q)

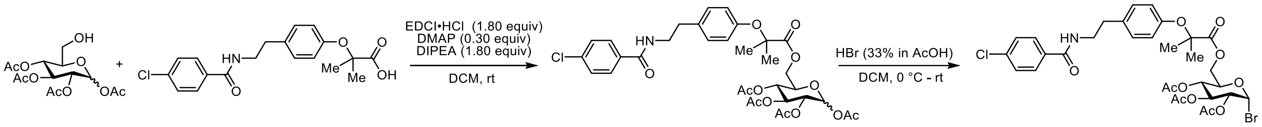

S17 S18 S19 1q

To a solution of compound **S17** (268 mg, 0.770 mmol, 1.00 equiv) in dry DCM (3.85 mL, 0.20 M) were added Bezafibrate **S18** (306 mg, 0.850 mmol, 1.10 equiv), DMAP (28.2 mg, 0.230 mmol, 0.300 equiv), EDCI·HCl (266 mg, 1.39 mmol, 1.80 equiv) and DIPEA (0.24 mL, 1.39 mmol, 1.80 equiv). After stirring at room temperature for 12 h, the reaction mixture was diluted with DCM and washed with saturated $NaHCO_3$ and brine successively. The organic phase was dried over Na_2SO_4, filtered, and concentrated *in vacuo*. The residue was purified by silica gel column chromatograph Hexanes: EtOAc [1:1 (v/v)] to give **S19** (470 mg, 0.680 mmol, 88% yield) as a colorless oil.

1q was synthesized according to the General Procedure B using **S19** (464 mg, 0.670 mmol, 1.00 equiv) as the substrate. After work up, the reaction mixture was purified by flash column chromatography on silica gel, eluting with Hexanes: EtOAc [2:1 (v/v)] to afford the title compound (331 mg, 0.460 mmol, 69%) as a white foam. **R*f*** = 0.30 [Hexanes: EtOAc 2:1 (v/v)]. **^{1}H NMR** (700 MHz, $CDCl_3$, 25 ºC, δ): 7.62 (d, *J* = 8.4 Hz, 1H), 7.37 (d, *J* = 8.4 Hz, 1H), 7.10 (d, *J* = 8.4 Hz, 1H), 6.82 (d, *J* = 8.4 Hz, 1H), 6.52 (d, *J* = 4.2 Hz, 1H), 6.16 (t, *J* = 5.6 Hz, 1H), 5.52 (t, *J* = 9.8 Hz, 1H), 5.07 (t, *J* = 9.8 Hz, 1H), 4.70 (dd, J = 10.5, 4.2 Hz, 1H), 4.19-4.37 (m, 3H), 3.55-3.73 (m, 2H), 2.86 (t, *J* = 7.0 Hz, 1H), 2.08 (s, 3H), 2.04 (s, 3H), 2.01 (s, 3H), 1.61 (s, 3H), 1.59 (s, 3H). **^{13}C NMR** (175 MHz, $CDCl_3$, 25 ºC, δ): 173.87, 169.98, 169.95, 169.52, 166.48, 154.12, 137.73, 133.16, 132.75, 129.68, 129.68, 128.93, 128.93, 128.41, 128.41, 119.83, 119.83, 86.45, 79.28, 72.16, 70.66, 70.14, 67.52, 61.92, 41.33, 34.84, 25.85, 25.26, 20.78, 20.73, 20.69. **HRMS** (ESI-TOF) *m/z* calcd for $C_{31}H_{36}BrClNO_{11}$ [(M + H)$^+$], 712.1155, found, 712.1158.

2,3,4-Tri-*O*-acetyl-6-*O*-[((2-(4-isobutylphenyl)propanoyl)]-α-D-glucopyranosyl bromide (1r)

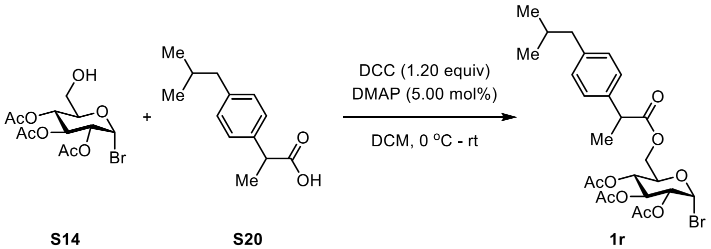

S14 S20 1r

The reaction was performed according to the same procedure as synthesizing **1p**. Ibuprofen **S20** (115 mg, 0.60 mmol, 1.50 equiv) was used as the coupling partner. After work up, the reaction mixture was purified by flash column chromatography on silica gel, eluting with Hexanes: EtOAc [3:1 (v/v)] to afford the title compound (196 mg, 0.352 mmol, 70%) as a white solid. **R*f*** = 0.60 [Hexanes: EtOAc 2:1 (v/v)]. **^{1}H NMR** (500 MHz, $CDCl_3$, 25 ºC, δ): 7.21 (d, *J* = 8.4 Hz, 3H), 7.10 (d, *J* = 8.4 Hz, 3H), 6.55 (d, *J* = 4.1 Hz, 0.5H), 6.51 (d, *J* = 4.1 Hz, 1H), 5.50 (td, *J* = 9.7, 2.2 Hz, 1.5H), 5.06 – 4.99 (m, 1.5H), 4.70 (dd, *J* = 9.8, 4.1 Hz,

0.5H), 4.67 (dd, *J* = 9.8, 4.1 Hz, 1H), 4.23 (ddd, *J* = 11.9, 10.7, 5.6 Hz, 4.5H), 3.73 (q, *J* = 7.1 Hz, 1.5H), 2.43 (d, *J* = 7.2 Hz, 3.5H), 2.09 (s, 1.5H), 2.08 (s, 3H), 2.03 (s, 3H), 2.02 (s, 4.5H), 1.99 (s, 1.5H), 1.86 (dt, *J* = 13.5, 6.8 Hz, 1.5H), 1.50 (d, *J* = 7.2 Hz, 1.5H), 1.49 (d, *J* = 7.2 Hz, 3H), 0.89 (d, *J* = 6.6 Hz, 9.5H). ^{13}**C NMR** (125 MHz, $CDCl_3$, 25 ºC, δ): 174.29, 169.95, 169.82, 169.49, 140.78, 140.76, 137.37, 137.13, 129.48, 129.44, 127.41, 127.37, 86.63, 86.58, 72.43, 72.34, 70.67, 70.26, 67.55, 67.26, 61.33, 60.98, 45.10, 44.95, 30.25, 30.23, 22.50, 20.73, 20.71, 20.65, 20.60, 18.42, 18.22. **HRMS** (ESI-TOF) *m/z* calcd for $C_{25}H_{34}BrO_9$ $[(M + H)^+]$, 557.1381, found, 557.1388.

2,3,4-Tri-*O*-acetyl-6-*O*-[(4-(*N*,*N*-dipropylsulfamoyl)benzoyl)]-α-D-glucopyranosyl bromide (1s)

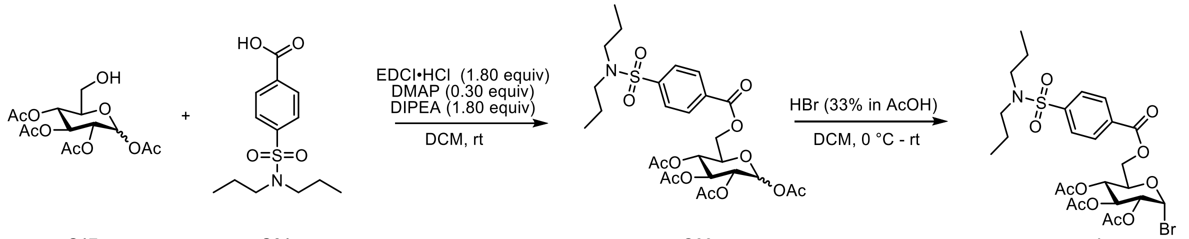

To a solution of compound **S17** (294 mg, 0.800 mmol, 1.00 equiv) in dry DCM (4.00 mL, 0.20 M) were added Probenecid **S21** (274 mg, 0.960 mmol, 1.20 equiv), DMAP (29.3 mg, 0.240 mmol, 0.300 equiv), EDCI·HCl (276 mg, 1.44 mmol, 1.80 equiv) and DIPEA (0.25 mL, 1.44 mmol, 1.80 equiv). After stirring at room temperature for 12 h, the reaction mixture was diluted with DCM and washed with saturated $NaHCO_3$ and brine successively. The organic phase was dried over Na_2SO_4, filtered, and concentrated *in vacuo*. The residue was purified by silica gel column chromatograph Hexanes: EtOAc [3:1 (v/v)] to give **S22** (209 mg, 0.340 mmol, 46.4% yield) as a colorless oil.

1s was synthesized according to the General Procedure B using **S22** (209 mg, 0.340 mmol, 46% yield) as the substrate. After work up, the reaction mixture was purified by flash column chromatography on silica gel, eluting with Hexanes: EtOAc [4:1 (v/v)] to afford the title compound (90.0 mg, 0.140 mmol, 40%) as a white foam. $\mathbf{R}_f$ = 0.50 [Hexanes: EtOAc 2:1 (v/v)]. ^{1}**H NMR** (700 MHz, $CDCl_3$, 25 ºC, δ): 8.16 (d, *J* = 8.4 Hz, 1H), 7.90 (d, *J* = 8.4 Hz, 1H), 6.62 (d, *J* = 4.2 Hz, 1H), 5.60 (t, *J* = 9.8 Hz, 1H), 5.26 (t, *J* = 9.8 Hz, 1H), 4.85 (dd, *J* = 9.8, 4.2 Hz, 1H), 4.55 (d, *J* = 12.6, 2.1 Hz, 1H), 4.42-4.49 (m, 2H), 3.05-3.14 (m, 4H), 2.11 (s, 2H), 2.07 (s, 1H), 2.04 (s, 2H), 1.50-1.58 (m, 4H), 0.88 (t, *J* = 7.0 Hz, 6H).^{13}**C NMR** (175 MHz, $CDCl_3$, 25 ºC, δ): 169.98, 169.96, 169.62, 164.85, 144.72, 132.78, 130.58, 130.58, 127.29, 127.29, 86.54, 72.22, 70.72, 70.25, 67.45, 62.07, 50.22, 50.22, 22.19, 22.19, 20.80, 20.76, 20.73, 11.31, 11.31.**HRMS** (ESI-TOF) *m/z* calcd for $C_{25}H_{35}BrNO_{11}S$ $[(M + H)^+]$, 636.1109, found, 636.1113.

2,3,4-Tri-*O*-acetyl-6-*O*-[((2-(10-oxo-10,11-dihydrodibenzo[*b,f*]thiepin-2-yl)propanoyl)]-α-D-glucopyranosyl bromide (1t)

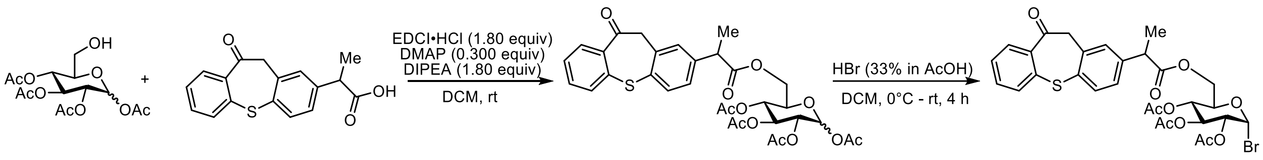

S17 S23 S24 **1t** (1:1.15)

To a solution of compound **S17** (452 mg, 1.30 mmol, 1.00 equiv) in dry DCM (6.5 mL, 0.20 M) were added Zaltoprofen **S23** (237 mg, 1.43 mmol, 1.10 equiv), DMAP (47.6 mg, 0.390 mmol, 0.300 equiv), EDCI·HCl (448 mg, 2.34 mmol, 1.80 equiv) and DIPEA (0.480 mL, 2.34 mmol, 1.80 equiv). After stirring at room temperature for overnight, the reaction mixture was diluted with DCM and washed with saturated $NaHCO_3$ and brine successively. The organic phase was dried over Na_2SO_4, filtered, and concentrated *in vacuo*. The residue was purified by silica gel column chromatography Hexanes: EtOAc [2:1 (v/v)] to give **S24** (700 mg, 1.11 mmol, 86% yield) as a colorless oil.

1t was synthesized according to the General Procedure B using **S24** (440 mg, 0.700 mmol, 1.00 equiv) as the substrate. After work up, the reaction mixture was purified by flash column chromatography on silica gel, eluting with Hexanes: EtOAc [3:1 (v/v)] to afford the title compound (289 mg, 0.450 mmol, 64%) as a white foam. $\mathbf{R_f}$ = 0.25 [Hexanes: EtOAc 3:1 (v/v)]. **^{1}H NMR** (700 MHz, $CDCl_3$, 25 °C, δ): 8.20 (dd, *J* = 3.5, 1.4 Hz, 1H), 8.19 (dd, J = 3.5, 1.4 Hz, 1.15H), 7.58- 7.63 (m, 4.12H), 7.39-7.44 (m, 4.30H), 7.29-7.33 (m, 2.15H), 7.16 (t, *J* = 2.1 Hz, 1.15H), 7.15 (dt, *J* = 2.1 Hz, 1H), 6.55 (d, *J* = 4.2 Hz, 1H), 6.52 (d, *J* = 4.2 Hz, 1.15H), 5.49 (t, *J* = 9.8 Hz, 2.15H), 5.02 (t, *J* = 9.8 Hz, 1.15H), 4.97 (t, *J* = 9.8 Hz, 1H), 4.70 (dd, *J* = 9.8, 4.2 Hz, 1H), 4.65 (dd, *J* = 9.8, 4.2 Hz, 1.15H), 4.38 (s, 4.30H), 4.16-4.30 (m, 6.45H), 3.77 (qd, *J* = 7.0, 2.1 Hz, 2.15H), 2.10 (s, 3H), 2.09 (s, 3.45H), 2.04 (s, 3.45H), 2.03 (s, 3.45H), 2.03 (s, 3H), 1.99 (s, 3H), 1.51 (d, *J* = 7.0 Hz, 3H), 1.49 (d, *J* = 7.0 Hz, 3.45H). **^{13}C NMR** (175 MHz, $CDCl_3$, 25 °C, δ): 191.50, 191.48, 173.48, 173.41, 169.99, 169.96, 169.87, 169.85, 169.54, 169.37, 142.30, 142.08, 140.31, 140.29, 138.15, 138.10, 136.33, 136.30, 133.58, 133.55, 132.63, 132.60, 131.70, 131.67, 131.65, 131.62, 131.00, 131.00, 128.98, 128.66, 126.97, 126.96, 126.73, 126.52, 86.55, 86.51, 72.32, 72.27, 70.68, 70.67, 70.23, 70.22, 67.42, 67.19, 61.54, 61.27, 51.20, 51.19, 45.16, 45.03, 20.79, 20.79, 20.79, 20.79, 20.70, 20.61, 18.26, 18.15. **HRMS** (ESI-TOF) *m/z* calcd for $C_{29}H_{30}BrO_{10}S$ [(M + H)$^+$], 649.0738, found, 649.0738.

3,4,6-Tri-*O*-benzoyl-6-*O*-[(6-(3-((3r,5r,7r)-adamantan-1-yl)-4-methoxyphenyl)-2-naphthoyl)]-α-D-glucopyranosyl bromide (1z)

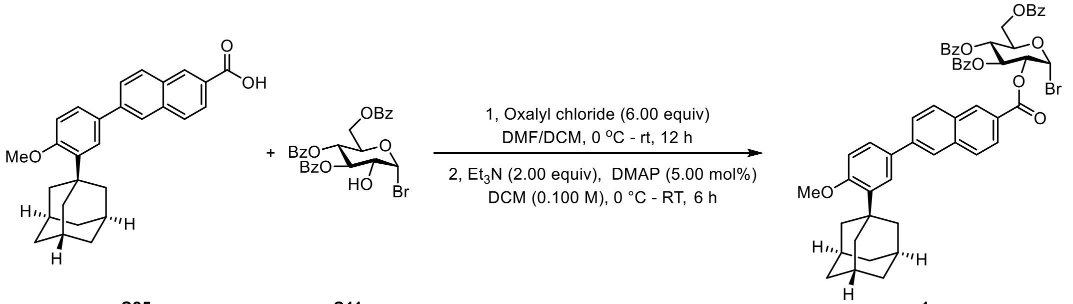

S25 S11 1z

To a solution of Adapalene **S25** (248 mg, 0.6 mmol, 1.00 equiv) in dry DCM (6 mL, 0.10 M) were added DMF (60.0 uL) and oxalyl chloride (457 mg, 6.60 mmol, 6.00 equiv) at 0 ºC. After stirring at room temperature for 12 h, the solvent was removed under vacuum and the residue was directly used for next step. To a solution of crude mixture obtained above in dry DCM (6.00 mL) were added Et_3N (101 mg, 1.00 mmol, 2.00 equiv), **S11** (278 mg, 0.500 mmol, 1.00 equiv) and DMAP (3.05 mg, 0.0250 mmol, 5.00 mol%) at 0 ºC. The reaction mixture stirred at room temperature for 6 h, then quenched with saturated $NaHCO_3$ solution (6.00 mL) and extracted with DCM (2×30 mL). The organic layer was collected, washed with brine, dried with anhydrous Mg_2SO_4 and filtered. The filtrate was concentrated *in vacuo.* The residue was purified by flash column chromatography on silica gel, eluting with Hexanes: EtOAc [3:1 (v/v)] to afford the title compound **1z** (237 mg, 0.250 mmol, 50%) as a white solid. $\mathbf{R_f}$ = 0.70 [Hexanes: EtOAc 2:1 (v/v)]. **^{1}H NMR** (500 MHz, $CDCl_3$, 25 ºC, δ): 8.57 (s, 1H), 8.09 (d, *J* = 7.3 Hz, 2H), 8.01 – 7.95 (m, 5H), 7.88 (dd, *J* = 15.3, 8.1 Hz, 3H), 7.79 (dd, *J* = 8.7, 1.4 Hz, 1H), 7.62 – 7.56 (m, 2H), 7.55 – 7.51 (m, 2H), 7.43 (ddd, *J* = 24.4, 15.6, 7.7 Hz, 5H), 7.29 (t, *J* = 7.8 Hz, 2H), 6.99 (d, *J* = 8.5 Hz, 1H), 6.93 (d, *J* = 4.0 Hz, 1H), 6.33 (t, *J* = 9.8 Hz, 1H), 5.85 (dd, *J* = 16.7, 6.7 Hz, 1H), 5.39 (dd, *J* = 10.0, 4.1 Hz, 1H), 4.83 – 4.75 (m, 1H), 4.69 (dd, *J* = 12.5, 2.4 Hz, 1H), 4.53 (dt, *J* = 12.3, 4.4 Hz, 1H), 3.90 (s, 3H), 2.18 (s, 6H), 2.10 (s, 3H), 1.80 (s, 6H). **^{13}C NMR** (125 MHz, $CDCl_3$, 25 ºC, δ): 166.18, 165.80, 165.69, 165.25, 159.14, 141.97, 139.16, 136.46, 133.79, 133.48, 133.40, 132.50, 132.01, 131.24, 130.09, 129.98, 129.88, 129.61, 128.96, 128.70, 128.65, 128.61, 128.58, 128.51, 126.71, 126.09, 125.88, 125.61, 125.18, 124.77, 112.24, 87.14, 72.88, 71.74, 70.88, 68.17, 62.12, 55.30, 40.72, 37.34, 37.25, 29.23. **HRMS** (ESI-TOF) *m/z* calcd for $C_{55}H_{49}BrO_{10}Na$ [(M + Na)$^+$], 971.2401, found, 971.2402.

General Procedure C (for the C2-ketonylation reaction):

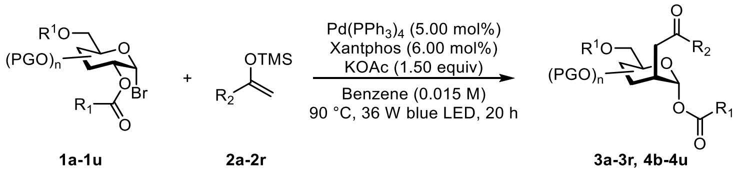

In a glovebox, to an oven-dried 150 mL pressure vessel was added $Pd(PPh_3)_4$ (11.55 mg, 10.0 µmol, 5.00 mol%), Xantphos (6.94 mg, 12.0 µmol, 6.00 mol%), bromo-sugar (0.200 mmol, 1.00 equiv), KOAc (29.45 mg, 0.300 mmol, 1.5 equiv), silyl enol ether (**1a-1l**, 0.400 mmol, 2.00 equiv; **1m-1r**, 0.800 mmol, 4.00 equiv) and benzene (13.33 mL, 0.015 M). The pressure vessel was equipped with a stir bar, capped and taken out of the glovebox. After the reaction mixture was stirred at 90 °C, irradiated with 36 W Blue LEDs for 20 h (as the setup described above). The reaction mixture was then concentrated *in vacuo* and residue was purified by flash column chromatography on silica gel to afford the desired product. Given that many products are new and have not been characterized, if the diastereomers could not be separated by the flash column chromatography, we purified them through HPLC system [Lux® 5*µ*m i-Amylose-1 column eluting with isopropanol:hexane (v/v) at the flow rate of 1.0 ml/min] to obtain pure NMR spectra.

(2R,3S,4R,5S,6R)-6-(acetoxymethyl)-3-(2-oxo-2-phenylethyl)tetrahydro-2H-pyran-2,4,5-triyl triacetate (3a)

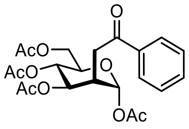

3a

Prepared according to the General Procedure C, the title compound was obtained as a white solid (73.8 mg, 0.164 mmol, 82% yield, axial: equatorial = 5.6:1). $\mathbf{R}_{f}$ = 0.30 [Hexanes: EtOAc 3:1 (v/v)]. The analytic amount of axial and equatorial diastereomers were separated by the analytical HPLC column.

Data for axial product (**3a-*ax***): t_R = 7.9 min, 10% (v/v) isopropanol in hexane at the flow rate of 1.0 ml/min. ^{1}**H NMR** (700 MHz, $CDCl_3$, 25 ºC, δ): 8.05 – 7.95 (m, 2H), 7.61 (t, *J* = 7.4 Hz, 1H), 7.50 (t, *J* = 7.8 Hz, 2H), 6.09 (s, 1H, *H1*), 5.57 (dd, *J* = 9.9, 4.8 Hz, 1H, *H3*), 5.23 (t, *J* = 10.0 Hz, 1H, *H4*), 4.24 (dd, *J* = 12.3, 4.6 Hz, 1H, *H6*), 4.10 (dd, *J* = 12.3, 2.4 Hz, 1H, *H7*), 4.05 (ddd, *J* = 10.0, 4.5, 2.4 Hz, 1H, *H5*), 3.47 – 3.32 (m, 1H, *H8*), 3.26 – 3.03 (m, 2H, *H2*, *H9*), 2.18 (s, 3H), 2.10 (s, 3H), 2.06 (s, 3H), 1.96 (s, 3H). ^{13}**C NMR** (175 MHz, $CDCl_3$, 25 ºC, δ): 197.01, 170.75, 170.02, 169.87, 168.83, 136.57, 133.73, 128.91, 128.21, 93.35 (*C1*), 70.18 (*C5*), 69.44 (*C3*), 66.39 (*C4*), 62.44 (*C6*), 37.68 (*C2*), 34.34, 21.16, 20.93, 20.90, 20.86. **HRMS** (ESI-TOF) *m/z* calcd for $C_{22}H_{30}NO_{10}$ [$(M + NH_4)^+$], 268.1864, found, 268.1862.

Data for equatorial product(**3a-*eq***): t_R = 8.7 min, 10% (v/v) isopropanol in hexane at the flow rate of 1.0 ml/min. ^{1}**H NMR** (700 MHz, $CDCl_3$, 25 ºC, δ): 7.90 (dd, *J* = 8.3, 1.1 Hz, 2H), 7.62 – 7.54 (m, 1H), 7.47 (t, *J* = 7.8 Hz, 2H), 6.34 (d, *J* = 3.1 Hz, 1H, *H1*), 5.34 (dd, *J* = 11.2, 9.3 Hz, 1H, *H3*), 5.15 – 5.10 (m, 1H, *H4*), 4.30 (dd, *J* = 12.4, 4.1 Hz, 1H, *H6*), 4.10 – 4,06 (m, 2H, *H5*, *H7*), 3.07 – 2.93 (m, 2H, *H2*, *H8*), 2.93 – 2.86 (m, 1H, *H9*), 2.11 (s, 3H), 2.09 (s,3H), 2.03 (s, 3H), 1.93 (s, 3H). ^{13}**C NMR** (175 MHz, $CDCl_3$, 25 ºC, δ): 196.80, 171.00, 170.91, 169.76, 168.96, 136.38, 133.72, 128.93, 128.20, 92.30 (*C1*), 71.66 (*C3*), 69.92 (*C5*), 69.19 (*C4*), 61.99 (*C6*), 39.57 (*C2*), 35.79, 29.86, 21.02, 20.90, 20.82, 20.80. **HRMS** (ESI-TOF) *m/z* calcd for $C_{22}H_{27}O_{10}$ [$(M + H)^+$], 451.1599, found, 451.1597.

(2R,3S,4R,5S,6R)-3-(2-([1,1'-biphenyl]-4-yl)-2-oxoethyl)-6-(acetoxymethyl)tetrahydro-2H-pyran-2,4,5-triyl triacetate (3b)

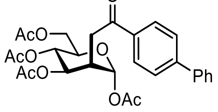

3b

Prepared according to the General Procedure C, the title compound was obtained as a white solid (82.1 mg, 0.156 mmol, 78% yield, axial: equatorial = 5.0:1). $\mathbf{R}_{f}$ = 0.30 [Hexanes: EtOAc 3:1 (v/v)]. The analytic amount of axial and equatorial diastereomers were separated by the analytical HPLC column.

Data for axial product (**3b-*ax***): t_R = 12.1 min, 10% (v/v) isopropanol in hexane at the flow rate of 1.0 ml/min. **^{1}H NMR** (700 MHz, $CDCl_3$, 25 °C, δ): 8.07 (d, *J* = 8.3 Hz, 2H), 7.72 (d, *J* = 8.3 Hz, 2H), 7.65 (d, *J* = 7.9 Hz, 2H), 7.49 (t, *J* = 7.6 Hz, 2H), 7.42 (dd, *J* = 10.6, 4.1 Hz, 1H), 6.11 (s, 1H, *H1*), 5.58 (dd, *J* = 9.8, 4.8 Hz, 1H, *H3*), 5.25 (t, *J* = 10.0 Hz, 1H, *H4*), 4.25 (dd, *J* = 12.3, 4.6 Hz, 1H, *H6*), 4.11 (dd, *J* = 12.3, 2.3 Hz, 1H, *H7*), 4.07 (ddd, *J* = 9.9, 4.4, 2.4 Hz, 1H, *H5*), 3.43 (dd, *J* = 21.3, 7.7 Hz, 1H), 3.26 – 3.12 (m, 2H, *H2*), 2.19 (s, 3H), 2.11 (s, 3H), 2.07 (s, 3H), 1.98 (s, 3H). **^{13}C NMR** (175 MHz, $CDCl_3$, 25 °C, δ): 196.59, 170.76, 170.05, 169.89, 168.84, 146.42, 139.84, 135.26, 129.15, 128.83, 128.53, 127.53, 127.45, 93.37, 70.20, 69.47, 66.43, 62.46, 37.74, 34.37, 21.18, 20.97, 20.92, 20.87. **HRMS** (ESI-TOF) *m/z* calcd for $C_{28}H_{31}O_{10}$ [$(M + H)^+$], 527.1912, found, 527.1912.

Data for equatorial product(**3b-*eq***): t_R = 13.9 min, 10% (v/v) isopropanol in hexane at the flow rate of 1.0 ml/min. **^{1}H NMR** (700 MHz, $CDCl_3$, 25 °C, δ): 7.97 (d, *J* = 8.4 Hz, 2H), 7.69 (d, *J* = 8.4 Hz, 2H), 7.62 (dd, *J* = 8.2, 1.0 Hz, 2H), 7.48 (t, *J* = 7.6 Hz, 2H), 7.41 (dd, *J* = 9.9, 4.8 Hz, 1H), 6.36 (d, *J* = 3.1 Hz, 1H, *H1*), 5.35 (dd, *J* = 11.2, 9.3 Hz, 1H, *H3*), 5.14 (t, *J* = 9.6 Hz, 1H, *H4*), 4.31 (dd, *J* = 12.9, 4.6 Hz, 1H, *H6*), 4.10 – 4.04 (m, 2H, , *H5, H7*), 3.05 – 2.97 (m, 2H, , *H2*), 2.93 (dd, *J* = 12.5, 5.6 Hz, 1H), 2.13 (s, 3H), 2.10 (s, 3H), 2.04 (s, 3H), 1.95 (s, 3H). **^{13}C NMR** (175 MHz, $CDCl_3$, 25 °C, δ): 196.37, 171.03, 170.93, 169.77, 168.99, 146.42, 139.79, 135.05, 129.16, 128.81, 128.55, 127.54, 127.42, 92.34, 71.68, 69.93, 69.20, 62.00, 39.59, 35.83, 21.04, 20.90, 20.85, 20.80. **HRMS** (ESI-TOF) *m/z* calcd for $C_{28}H_{31}O_{10}$ [$(M + H)^+$], 527.1912, found, 527.1914.

(2R,3S,4R,5S,6R)-6-(acetoxymethyl)-3-(2-(4-chlorophenyl)-2-oxoethyl)tetrahydro-2H-pyran-2,4,5-triyl triacetate (3c)

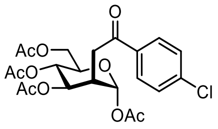

3c

Prepared according to the General Procedure C, the title compound was obtained as a white solid (77.5 mg, 0.160 mmol, 80% yield, axial: equatorial = 5.6:1). **R*f*** = 0.28 [Hexanes: EtOAc 3:1 (v/v)]. The analytic amount of axial and equatorial diastereomers were separated by the analytical HPLC column.

Data for axial product (**3c-*ax***): t_R = 8.5 min, 10% (v/v) isopropanol in hexane at the flow rate of 1.0 ml/min. **^{1}H NMR** (700 MHz, $CDCl_3$, 25 °C, δ): 7.98 – 7.89 (m, 2H), 7.51 – 7.46 (m, 2H), 6.07 (s, 1H, *H1*), 5.58 – 5.51 (m, 1H, *H3*), 5.21 (t, *J* = 10.0 Hz, 1H, *H4*), 4.24 (dd, *J* = 12.3, 4.7 Hz, 1H, *H6*), 4.09 (dd, *J* = 12.3, 2.4 Hz, 1H, *H7*), 4.05 (ddd, *J* = 10.1, 4.6, 2.4 Hz, 1H, *H5*), 3.46 – 3.28 (m, 1H), 3.21 – 3.04 (m, 2H, *H2*), 2.18 (s, 3H), 2.09 (s, 3H), 2.06 (s, 3H), 1.96 (s, 3H). **^{13}C NMR** (175 MHz, $CDCl_3$, 25 °C, δ): 195.83, 170.70, 170.03, 169.80, 168.80, 140.26, 134.85, 129.63, 129.25, 93.21, 70.17, 69.38, 66.34, 62.41, 37.71, 34.32,

21.14, 20.92, 20.89, 20.84. **HRMS** (ESI-TOF) *m/z* calcd for $C_{22}H_{29}ClNO_{10}$ [$(M + NH_4)^+$], 502.1475, found, 502.1474.

Data for equatorial product(**3c-*eq***): t_R = 10.3 min, 10% (v/v) isopropanol in hexane at the flow rate of 1.0 ml/min. ^{1}**H NMR** (700 MHz, $CDCl_3$, 25 ºC, δ): 7.86 – 7.79 (m, 2H), 7.48 – 7.41 (m, 2H), 6.33 (d, *J* = 3.1 Hz, 1H, *H1*), 5.32 (dd, *J* = 11.4, 9.4 Hz, 1H, *H3*), 5.13 (t, *J* = 9.8 Hz, 1H, *H4*), 4.30 (dd, *J* = 12.4, 4.1 Hz, 1H, *H6*), 4.06 (ddd, *J* = 10.0, 6.1, 2.3 Hz, 2H, *H5, H7*), 3.02 – 2.88 (m, 2H, *H2*), 2.85 (dd, *J* = 17.4, 8.4 Hz, 1H), 2.11 (s, 3H), 2.09 (s, 3H), 2.03 (s, 3H), 1.94 (s, 3H). ^{13}**C NMR** (175 MHz, $CDCl_3$, 25 ºC, δ): 195.54, 170.99, 170.90, 169.74, 168.97, 140.26, 134.66, 129.60, 129.26, 92.23, 71.59, 69.95, 69.12, 61.96, 39.47, 35.75, 21.03, 20.89, 20.84, 20.79. **HRMS** (ESI-TOF) *m/z* calcd for $C_{22}H_{29}ClNO_{10}$ [$(M + NH_4)^+$], 502.1475, found, 502.1473.

(2R,3S,4R,5S,6R)-6-(acetoxymethyl)-3-(2-(4-fluorophenyl)-2-oxoethyl)tetrahydro-2H-pyran-2,4,5-triyl triacetate (3d)

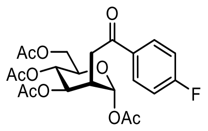

3d

Prepared according to the General Procedure C, the title compound was obtained as a white solid (57.1 mg, 0.121 mmol, 61% yield, axial: equatorial = 4.4:1). $\mathbf{R}_f$ = 0.25 [Hexanes: EtOAc 3:1 (v/v)]. The analytic amount of axial and equatorial diastereomers were separated by the analytical HPLC column.

Data for axial product (**3d-*ax***): t_R = 8.5 min, 10% (v/v) isopropanol in hexane at the flow rate of 1.0 ml/min. ^{1}**H NMR** (500 MHz, $CDCl_3$, 25 ºC, δ): 8.03 (dd, *J* = 8.7, 5.4 Hz, 2H), 7.17 (t, *J* = 8.5 Hz, 2H), 6.08 (s, 1H, *H1*), 5.62 – 5.51 (m, 1H, *H3*), 5.21 (t, *J* = 10.0 Hz, 1H, *H4*), 4.24 (dd, *J* = 12.3, 4.7 Hz, 1H, *H6*), 4.16 – 3.91 (m, 2H, *H5*, *H7*), 3.36 (q, *J* = 8.0 Hz, 1H), 3.18 – 3.05 (m, 2H, *H2*), 2.18 (s, 3H), 2.10 (s, 3H), 2.06 (s, 3H), 1.96 (s, 3H). ^{13}**C NMR** (125 MHz, $CDCl_3$, 25 ºC, δ): 195.41, 170.70, 170.03, 169.81, 168.80, 167.18, 165.14, 133.03, 133.01, 130.94, 130.86, 116.16, 115.98, 93.26, 70.19, 69.41, 66.39, 62.44, 37.74, 34.25, 21.15, 20.92, 20.89, 20.84. **HRMS** (ESI-TOF) *m/z* calcd for $C_{22}H_{29}FNO_{10}$ [$(M + NH_4)^+$], 486.1770, found, 486.1770.

Data for equatorial product(**3d-*eq***): t_R = 9.4 min, 10% (v/v) isopropanol in hexane at the flow rate of 1.0 ml/min. ^{1}**H NMR** (500 MHz, $CDCl_3$, 25 ºC, δ): 7.93 (dd, *J* = 8.7, 5.4 Hz, 2H), 7.14 (t, *J* = 8.5 Hz, 2H), 6.33 (d, *J* = 3.0 Hz, 1H, *H1*), 5.37 – 5.28 (m, 1H, *H3*), 5.13 (t, *J* = 9.7 Hz, 1H, *H4*), 4.30 (dd, *J* = 12.5, 4.2 Hz, 1H, *H6*), 4.12 – 4.01 (m, 2H, *H5, H7*), 2.96 (ddd, *J* = 14.1, 9.4, 4.1 Hz, 2H, *H2*, *H8*), 2.85 (dd, *J* = 17.9, 8.9 Hz, 1H, *H9*), 2.12 (s, 3H), 2.09 (s, 3H), 2.03 (s, 3H), 1.94 (s, 3H). ^{13}**C NMR** (125 MHz, $CDCl_3$, 25 ºC, δ): 195.15, 170.99, 170.89, 169.74, 168.96, 167.16, 165.09, 132.85, 130.91, 130.84, 116.16, 115.99, 92.27,

71.63, 69.96, 69.16, 61.99, 39.53, 35.69, 21.02, 20.89, 20.83, 20.78. **HRMS** (ESI-TOF) *m/z* calcd for $C_{22}H_{26}FO_{10}$ [(M + H)$^+$], 469.1505, found, 569.1505.

(2R,3S,4R,5S,6R)-6-(acetoxymethyl)-3-(2-oxo-2-(3-(trifluoromethyl)phenyl)ethyl)tetrahydro-2H-pyran-2,4,5-triyl triacetate (3e)

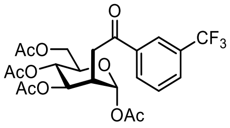

3e

Prepared according to the General Procedure C, the title compound was obtained as a white solid (73.6 mg, 0.142 mmol, 71% yield, axial: equatorial = 6.0:1). $\mathbf{R_f}$ = 0.30 [Hexanes: EtOAc 3:1 (v/v)]. The analytic amount of axial diastereomer was separated by the analytical HPLC column.

Data for axial product (**3e-*ax***): t_R = 5.9 min, 10% (v/v) isopropanol in hexane at the flow rate of 1.0 ml/min. **^{1}H NMR** (500 MHz, $CDCl_3$, 25 ºC, δ): 8.23 (s, 1H), 8.18 (d, *J* = 7.9 Hz, 1H), 7.87 (d, *J* = 7.8 Hz, 1H), 7.66 (t, *J* = 7.8 Hz, 1H), 6.08 (s, 1H, *H1*), 5.57 (dd, *J* = 9.9, 5.0 Hz, 1H, *H3*), 5.24 (t, *J* = 9.9 Hz, 1H, *H4*), 4.25 (dd, *J* = 12.3, 4.4 Hz, 1H, *H6*), 4.15 – 4.01 (m, 2H, *H7, H5*), 3.39 (t, *J* = 11.4 Hz, 1H), 3.25 – 3.12 (m, 2H, *H2*), 2.18 (s, 3H), 2.10 (s, 3H), 2.06 (s, 3H), 1.97 (s, 3H). **^{13}C NMR** (125 MHz, $CDCl_3$, 25 ºC, δ): 195.76, 170.70, 170.01, 169.81, 168.76, 137.04, 131.75, 131.49, 131.37, 130.16, 129.66, 125.08, 124.80, 122.64, 93.13, 93.11, 70.21, 69.35, 66.25, 62.33, 37.69, 34.55, 21.14, 21.12, 20.91, 20.82. **HRMS** (ESI-TOF) *m/z* calcd for $C_{23}H_{29}F_3NO_{10}$ [(M + NH_4)$^+$], 536.1738, found, 536.1735.

(2R,3S,4R,5S,6R)-6-(acetoxymethyl)-3-(2-(3-methoxyphenyl)-2-oxoethyl)tetrahydro-2H-pyran-2,4,5-triyl triacetate (3f)

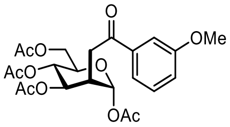

3f

Prepared according to the General Procedure C, the title compound was obtained as a white solid (80.7 mg, 0.168 mmol, 84% yield, axial: equatorial = 4.8:1). $\mathbf{R_f}$ = 0.25 [Hexanes: EtOAc 3:1 (v/v)]. The analytic amount of axial and equatorial diastereomers were separated by the analytical HPLC column.

Data for axial product (**3f-*ax***): t_R = 8.5 min, 10% (v/v) isopropanol in hexane at the flow rate of 1.0 ml/min. **^{1}H NMR** (700 MHz, $CDCl_3$, 25 ºC, δ): 7.60 – 7.55 (m, 1H), 7.50 (dd, *J* = 2.4, 1.7 Hz, 1H), 7.41 (t, *J* = 7.9 Hz, 1H), 7.17 – 7.13 (m, 1H), 6.09 (s, 1H, *H1*), 5.56 (dd, *J* = 9.9, 4.8 Hz, 1H, *H3*), 5.22 (t, *J* = 10.0 Hz, 1H, *H4*), 4.24 (dd, *J* = 12.3, 4.6 Hz, 1H, *H6*), 4.10 (dd, *J* = 12.3, 2.4 Hz, 1H, *H7*), 4.05 (ddd, *J* = 10.0, 4.5, 2.4 Hz, 1H, *H5*), 3.87 (s, 3H), 3.38 (dd, *J* = 21.3, 7.5 Hz, 1H), 3.19 – 3.06 (m, 2H, *H2*), 2.18 (s, 3H), 2.10 (s,

3H), 2.06 (s, 3H), 1.97 (s, 3H). **^{13}C NMR** (175 MHz, $CDCl_3$, 25 ºC, δ): 196.89, 170.76, 170.00, 169.89, 168.83, 160.07, 137.94, 129.90, 120.81, 120.09, 112.57, 93.32, 70.19, 69.44, 66.38, 62.43, 55.67, 37.75, 34.44, 21.16, 20.95, 20.90, 20.86. **HRMS** (ESI-TOF) *m/z* calcd for $C_{23}H_{32}NO_{11}$ [$(M + NH_4)^+$], 498.1970, found, 498.1967.

Data for equatorial product(**3f-*eq***): t_R = 9.9 min, 10% (v/v) isopropanol in hexane at the flow rate of 1.0 ml/min. **^{1}H NMR** (700 MHz, $CDCl_3$, 25 ºC, δ): 7.44 (dd, *J* = 10.0, 5.1 Hz, 2H), 7.37 (t, *J* = 7.9 Hz, 1H), 7.12 (dd, *J* = 8.1, 2.5 Hz, 1H), 6.33 (d, *J* = 3.1 Hz, 1H, *H1*), 5.33 (dd, *J* = 10.9, 9.4 Hz, 1H, *H3*), 5.19 – 5.09 (m, 1H, *H4*), 4.30 (dd, *J* = 12.4, 4.1 Hz, 1H, *H6*), 4.06 (ddd, *J* = 10.0, 5.7, 2.3 Hz, 2H, *H5*, *H7*), 3.86 (s, 3H), 2.96 (ddd, *J* = 12.9, 9.0, 4.7 Hz, 2H, *H2*), 2.87 (dd, *J* = 18.3, 9.4 Hz, 1H), 2.12 (s, 3H), 2.09 (s, 3H), 2.03 (s, 3H), 1.94 (s, 3H). **^{13}C NMR** (175 MHz, $CDCl_3$, 25 ºC, δ): 196.66, 181.32, 170.97, 170.91, 169.77, 168.97, 160.09, 137.73, 129.89, 120.76, 120.10, 112.54, 92.25, 71.62, 69.90, 69.21, 61.99, 55.66, 39.61, 35.88, 21.03, 20.90, 20.83, 20.80. **HRMS** (ESI-TOF) *m/z* calcd for $C_{23}H_{29}O_{11}$ [$(M + H)^+$], 481.1704, found, 481.1702.

(2R,3S,4R,5S,6R)-6-(acetoxymethyl)-3-(2-(4-methoxy-3-(trifluoromethyl)phenyl)-2-oxoethyl)tetrahydro-2H-pyran-2,4,5-triyl triacetate (3g)

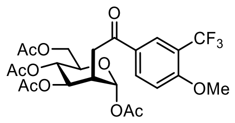

3g

Prepared according to the General Procedure C, the title compound was obtained as a white solid (80.0 mg, 0.145 mmol, 73% yield, axial: equatorial = 5.6:1). **R*f*** = 0.30 [Hexanes: EtOAc 3:1 (v/v)]. The analytic amount of axial diastereomer was separated by the analytical HPLC column.

Data for axial product (**3g-*ax***): t_R = 11.3 min, 10% (v/v) isopropanol in hexane at the flow rate of 1.0 ml/min. **^{1}H NMR** (500 MHz, $CDCl_3$, 25 ºC, δ): 8.21 (s, 1H), 8.18 (d, *J* = 8.7 Hz, 1H), 7.09 (d, *J* = 8.7 Hz, 1H), 6.06 (s, 1H, *H1*), 5.56 (dd, *J* = 9.9, 5.0 Hz, 1H, *H3*), 5.23 (t, *J* = 10.0 Hz, 1H, *H4*), 4.25 (dd, *J* = 12.3, 4.4 Hz, 1H, *H6*), 4.10 (dd, *J* = 12.3, 2.2 Hz, 1H, *H7*), 4.07 – 4.03 (m, 1H, *H5*), 4.00 (s, 3H), 3.41 – 3.27 (m, 1H), 3.19 – 3.07 (m, 2H, *H2*), 2.18 (s, 3H), 2.10 (s, 3H), 2.06 (s, 3H), 1.97 (s, 3H). **^{13}C NMR** (125 MHz, $CDCl_3$, 25 ºC, δ): 194.63, 170.73, 170.02, 169.84, 168.80, 161.54, 133.96, 128.87, 127.85, 119.07, 111.93, 93.24, 70.20, 69.37, 66.35, 62.36, 56.54, 56.48, 37.75, 33.99, 21.13, 20.93, 20.84. **HRMS** (ESI-TOF) *m/z* calcd for $C_{24}H_{28}F_3O_{11}$ [$(M + H)^+$], 549.1578, found, 549.1574.

(2R,3S,4R,5S,6R)-6-(acetoxymethyl)-3-(2-(6-(tert-butyl)-1,1-dimethyl-2,3-dihydro-1H-inden-4-yl)-2-oxoethyl)tetrahydro-2H-pyran-2,4,5-triyl triacetate (3h)

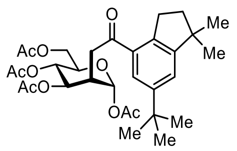

3h

Prepared according to the General Procedure C, the title compound was obtained as a white solid (91.8 mg, 0.159 mmol, 80% yield, axial: equatorial = 4.3:1). $\mathbf{R}_f$ = 0.35 [Hexanes: EtOAc 3:1 (v/v)]. The analytic amount of axial and equatorial diastereomers were separated by the analytical HPLC column.

Data for axial product (**3h-*ax***): t_R = 3.3 min, 10% (v/v) isopropanol in hexane at the flow rate of 1.0 ml/min. **^{1}H NMR** (500 MHz, $CDCl_3$, 25 ºC, δ): 7.70 (d, *J* = 1.7 Hz, 1H), 7.37 (d, *J* = 1.5 Hz, 1H), 6.06 (s, 1H, *H1*), 5.57 (dd, *J* = 9.9, 5.0 Hz, 1H, *H3*), 5.25 (t, *J* = 10.0 Hz, 1H, *H4*), 4.25 (dd, *J* = 12.3, 4.2 Hz, 1H, *H6*), 4.09 (dd, *J* = 12.3, 2.3 Hz, 1H, *H7*), 4.04 (ddd, *J* = 10.0, 4.0, 2.4 Hz, 1H, *H5*), 3.30 (q, *J* = 7.8 Hz, 1H), 3.23 – 3.08 (m, 4H, *H2*), 2.17 (s, 3H), 2.08 (s, 3H), 2.05 (s, 3H), 1.97 (s, 3H), 1.93 (td, *J* = 7.4, 2.0 Hz, 2H), 1.38 (s, 9H), 1.26 (d, *J* = 1.2 Hz, 6H). **^{13}C NMR** (125 MHz, $CDCl_3$, 25 ºC, δ): 198.99, 170.70, 169.95, 169.91, 168.87, 154.77, 150.31, 141.30, 133.22, 123.85, 123.83, 93.53, 70.20, 69.47, 66.30, 62.32, 43.58, 41.51, 37.73, 35.94, 34.92, 31.64, 31.02, 28.88, 21.14, 20.99, 20.84, 20.79. **HRMS** (ESI-TOF) *m/z* calcd for $C_{31}H_{43}O_{10}$ [(M + H)$^+$], 575.2851, found, 575.2846.

Data for equatorial product(**3h-*eq***): t_R = 3.8 min, 10% (v/v) isopropanol in hexane at the flow rate of 1.0 ml/min. **^{1}H NMR** (500 MHz, $CDCl_3$, 25 ºC, δ): 7.54 (d, *J* = 1.6 Hz, 1H), 7.35 (d, *J* = 1.4 Hz, 1H), 6.32 (d, *J* = 3.0 Hz, 1H, *H1*), 5.33 (dd, *J* = 11.1, 9.4 Hz, 1H, *H3*), 5.11 (t, *J* = 9.8 Hz, 1H, *H4*), 4.29 (dd, *J* = 12.5, 4.2 Hz, 1H, *H6*), 4.11 – 4.01 (m, 2H, *H5, H7*), 3.21 – 3.05 (m, 2H, *H2*), 3.00 – 2.89 (m, 2H), 2.84 (dd, *J* = 17.8, 9.5 Hz, 1H), 2.11 (s, 3H), 2.09 (s, 3H), 2.03 (s, 3H), 1.94 (s, 3H), 1.91 (dt, *J* = 8.1, 5.1 Hz, 2H), 1.35 (s, 9H), 1.25 (s, 6H). **^{13}C NMR** (125 MHz, $CDCl_3$, 25 ºC, δ): 199.02, 170.94, 170.91, 169.79, 168.96, 154.91, 150.25, 141.55, 128.80, 123.89, 123.73, 92.29, 71.71, 69.86, 69.34, 62.03, 43.59, 41.50, 39.74, 37.25, 34.89, 31.62, 30.90, 28.88, 28.85, 21.01, 20.88, 20.84, 20.81. **HRMS** (ESI-TOF) *m/z* calcd for $C_{31}H_{46}NO_{10}$ [(M + NH_4)$^+$], 592.3116, found, 592.3110.

(2R,3S,4R,5S,6R)-6-(acetoxymethyl)-3-(2-(naphthalen-1-yl)-2-oxoethyl)tetrahydro-2H-pyran-2,4,5-triyl triacetate (3i)

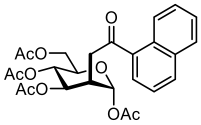

3i

Prepared according to the General Procedure C, the title compound was obtained as a white solid (81.0 mg, 0.162 mmol, 81% yield, axial: equatorial = 4.8:1). $\mathbf{R}_f$ = 0.35 [Hexanes: EtOAc 3:1 (v/v)]. The analytic amount of axial and equatorial diastereomers were separated by the analytical HPLC column.

Data for axial product (**3i-*ax***): t_R = 9.1 min, 10% (v/v) isopropanol in hexane at the flow rate of 1.0 ml/min. **^{1}H NMR** (700 MHz, $CDCl_3$, 25 ºC, δ): 8.61 (d, *J* = 8.6 Hz, 1H), 8.04 (d, *J* = 8.2 Hz, 1H), 7.97 (dd, *J* = 7.1, 0.7 Hz, 1H), 7.90 (d, *J* = 8.1 Hz, 1H), 7.61 (ddd, *J* = 8.4, 6.8, 1.3 Hz, 1H), 7.55 (t, *J* = 7.7 Hz, 2H), 6.19 (s, 1H, *H1*), 5.61 (dd, *J* = 9.9, 5.1 Hz, 1H, *H3*), 5.25 (t, *J* = 10.0 Hz, 1H, *H4*), 4.25 (dd, *J* = 12.3, 4.6 Hz, 1H, *H6*), 4.11 (dd, *J* = 12.3, 2.4 Hz, 1H, *H7*), 4.08 (ddd, *J* = 10.0, 4.5, 2.4 Hz, 1H, *H5*), 3.59 – 3.46 (m, 1H), 3.34 – 3.18 (m, 2H, *H2*), 2.20 (s, 3H), 2.07 (s, 3H), 2.07 (s, 3H), 1.98 (s, 3H). **^{13}C NMR** (175 MHz, $CDCl_3$, 25 ºC, δ): 200.87, 170.76, 170.02, 169.92, 168.89, 135.25, 134.12, 133.47, 130.22, 128.69, 128.39, 128.12, 126.80, 125.77, 124.51, 93.51, 70.26, 69.56, 66.38, 62.41, 38.00, 37.81, 21.19, 21.01, 20.89, 20.86. **HRMS** (ESI-TOF) *m/z* calcd for $C_{26}H_{32}NO_{10}$ [$(M + NH_4)^+$], 518.2021, found, 518.2016.

Data for equatorial product(**3i-*eq***): t_R = 10.3 min, 10% (v/v) isopropanol in hexane at the flow rate of 1.0 ml/min. **^{1}H NMR** (500 MHz, $CDCl_3$, 25 ºC, δ): 8.59 (d, *J* = 8.6 Hz, 1H), 8.01 (d, *J* = 8.2 Hz, 1H), 7.88 (d, *J* = 8.1 Hz, 1H), 7.78 (d, *J* = 6.5 Hz, 1H), 7.61 (dd, *J* = 11.3, 4.1 Hz, 1H), 7.55 (t, *J* = 7.0 Hz, 1H), 7.48 (dd, *J* = 13.7, 6.2 Hz, 1H), 6.42 (d, *J* = 3.2 Hz, 1H, *H1*), 5.35 (dd, *J* = 11.1, 9.4 Hz, 1H, *H3*), 5.16 (t, *J* = 9.6 Hz, 1H, *H4*), 4.31 (dd, *J* = 12.9, 4.5 Hz, 1H, *H6*), 4.08 (d, *J* = 10.7 Hz, 2H, *H7*, *H5*), 3.12 (dd, *J* = 16.7, 4.3 Hz, 1H), 3.05 (ddd, *J* = 15.4, 7.6, 3.6 Hz, 1H, *H2*), 2.97 (dd, *J* = 16.7, 7.8 Hz, 1H), 2.11 (s, 3H), 2.10 (s, 3H), 2.03 (s, 3H), 1.95 (s, 3H). **^{13}C NMR** (125 MHz, $CDCl_3$, 25 ºC, δ): 200.50, 171.02, 170.91, 169.76, 168.99, 134.93, 134.15, 133.52, 130.22, 128.64, 128.49, 127.96, 126.87, 125.82, 124.37, 92.33, 71.75, 69.99, 69.24, 62.00, 39.75, 39.20, 21.02, 20.91, 20.80. **HRMS** (ESI-TOF) *m/z* calcd for $C_{26}H_{29}O_{10}$ [$(M + H)^+$], 501.1755, found, 501.1751.

(2R,3S,4R,5S,6R)-6-(acetoxymethyl)-3-(2-(naphthalen-2-yl)-2-oxoethyl)tetrahydro-2H-pyran-2,4,5-triyl triacetate (3j)

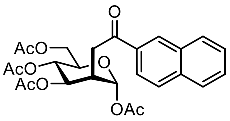

3j

Prepared according to the General Procedure C, the title compound was obtained as a white solid (73.0 mg, 0.146 mmol, 73% yield, axial: equatorial = 4.0:1). **R*f*** = 0.35 [Hexanes: EtOAc 3:1 (v/v)]. The analytic amount of axial and equatorial diastereomers were separated by the analytical HPLC column.

Data for axial product (**3j-*ax***): t_R = 6.6 min, 20% (v/v) isopropanol in hexane at the flow rate of 1.0 ml/min. **^{1}H NMR** (500 MHz, $CDCl_3$, 25 ºC, δ): 8.53 (s, 1H), 8.11 – 7.99 (m, 2H), 7.91 (dd, *J* = 13.6, 8.4 Hz, 2H), 7.69 – 7.57 (m, 2H), 6.15 (s, 1H, *H1*), 5.60 (dd, *J* = 9.9, 5.4 Hz, 1H, *H3*), 5.30 (t, *J* = 10.0 Hz, 1H, *H4*), 4.27 (dd, *J* = 12.3, 4.7 Hz, 1H, *H6*), 4.13 (dd, *J* = 12.2, 2.4 Hz, 1H, *H7*), 4.08 (ddd, *J* = 10.1, 4.5, 2.3 Hz, 1H, *H5*), 3.54 (dd, *J* = 17.6, 3.9 Hz, 1H), 3.29 (dd, *J* = 17.6, 8.9 Hz, 1H), 3.21 (dt, *J* = 9.0, 3.9 Hz, 1H, *H2*), 2.19 (s,

3H), 2.12 (s, 3H), 2.08 (s, 3H), 1.97 (s, 3H). **^{13}C NMR** (125 MHz, $CDCl_3$, 25 ºC, δ): 196.97, 170.75, 170.10, 169.88, 168.83, 135.93, 133.93, 132.63, 130.04, 129.77, 128.92, 128.82, 127.99, 127.17, 123.78, 93.39, 70.22, 69.52, 66.52, 62.51, 37.86, 34.40, 21.17, 20.96, 20.92, 20.88. **HRMS** (ESI-TOF) *m/z* calcd for $C_{26}H_{29}O_{10}$ [$(M + H)^+$], 501.1755, found, 501.1753.

Data for equatorial product(**3j-*eq***): t_R = 8.3 min, 20% (v/v) isopropanol in hexane at the flow rate of 1.0 ml/min. **^{1}H NMR** (500 MHz, $CDCl_3$, 25 ºC, δ): 8.39 (s, 1H), 7.98 (dd, *J* = 8.5, 1.7 Hz, 1H), 7.93 – 7.86 (m, 2H), 7.62 (dd, *J* = 10.9, 4.1 Hz, 1H), 7.60 – 7.55 (m, 1H), 6.39 (d, *J* = 2.8 Hz, 1H, *H1*), 5.39 (dd, *J* = 10.8, 9.4 Hz, 1H, *H3*), 5.15 (t, *J* = 9.7 Hz, 1H, *H4*), 4.31 (dd, *J* = 12.9, 4.6 Hz, 1H, *H6*), 4.08 (dd, *J* = 10.2, 2.6 Hz, 2H, *H7*, *H5*), 3.06 (ddt, *J* = 16.1, 13.3, 9.9 Hz, 3H, *H2*), 2.12 (s, 2H), 2.10 (s, 2H), 2.04 (s, 2H), 1.94 (s, 2H). **^{13}C NMR** (125 MHz, $CDCl_3$, 25 ºC, δ): 196.72, 170.92, 169.76, 168.97, 135.89, 133.76, 132.57, 129.92, 129.74, 128.94, 128.87, 127.98, 127.16, 123.78, 92.35, 71.72, 69.94, 69.25, 62.02, 39.66, 35.85, 21.04, 20.90, 20.86, 20.80. **HRMS** (ESI-TOF) *m/z* calcd for $C_{26}H_{29}O_{10}$ [$(M + H)^+$], 501.1755, found, 501.1753.

(2R,3S,4R,5S,6R)-6-(acetoxymethyl)-3-(2-(6-methoxypyridin-3-yl)-2-oxoethyl)tetrahydro-2H-pyran-2,4,5-triyl triacetate (3k)

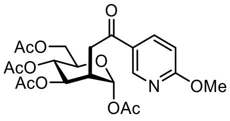

3k

Prepared according to the General Procedure C, the title compound was obtained as a white solid (67.4 mg, 0.140 mmol, 70% yield, axial: equatorial = 3.6:1). **R_f** = 0.25 [Hexanes: EtOAc 3:1 (v/v)]. The analytic amount of axial and equatorial diastereomers were separated by the analytical HPLC column.

Data for axial product (**3k-*ax***): t_R = 9.9 min, 10% (v/v) isopropanol in hexane at the flow rate of 1.0 ml/min. **^{1}H NMR** (500 MHz, $CDCl_3$, 25 ºC, δ): 8.84 (d, *J* = 2.1 Hz, 1H), 8.14 (dd, *J* = 8.7, 2.5 Hz, 1H), 6.81 (d, *J* = 8.7 Hz, 1H), 6.08 (s, 1H, *H1*), 5.55 (dd, *J* = 9.9, 5.3 Hz, 1H, *H3*), 5.21 (t, *J* = 10.0 Hz, 1H, *H4*), 4.24 (dd, *J* = 12.3, 4.5 Hz, 1H, *H6*), 4.09 (dd, *J* = 12.3, 2.3 Hz, 1H, *H7*), 4.05 (ddd, *J* = 10.3, 4.4, 2.5 Hz, 1H, *H5*), 4.02 (s, 3H), 3.32 (dd, *J* = 17.0, 3.5 Hz, 1H), 3.18 – 3.03 (m, 2H, *H2*), 2.17 (s, 3H), 2.11 (s, 3H), 2.05 (s, 3H), 1.96 (s, 3H). **^{13}C NMR** (125 MHz, $CDCl_3$, 25 ºC, δ): 194.77, 170.74, 169.95, 169.82, 168.79, 167.20, 149.24, 138.17, 126.41, 111.44, 93.28, 70.21, 69.41, 66.28, 62.36, 54.32, 37.61, 34.13, 21.14, 20.94, 20.91, 20.83. **HRMS** (ESI-TOF) *m/z* calcd for $C_{22}H_{28}NO_{11}$ [$(M + H)^+$], 482.1657, found, 482.1659.

Data for equatorial product(**3k-*eq***): t_R = 11.2 min, 10% (v/v) isopropanol in hexane at the flow rate of 1.0 ml/min. **^{1}H NMR** (500 MHz, $CDCl_3$, 25 ºC, δ): 8.71 (d, *J* = 2.1 Hz, 1H), 8.09 (dd, *J* = 8.8, 2.5 Hz, 1H), 6.79 (d, *J* = 8.8 Hz, 1H), 6.32 (d, *J* = 3.3 Hz, 1H, *H1*), 5.32 (dd, *J* = 11.2, 9.4 Hz, 1H, *H3*), 5.13 (t, *J* = 9.7 Hz, 1H, *H4*), 4.30 (dd, *J* = 12.6, 4.3 Hz, 1H, *H6*), 4.11 – 4.03 (m, 2H, *H7*, *H5*), 4.00 (s, 3H), 3.01 – 2.92 (m, 1H),

2.91 – 2.79 (m, 2H, *H2*), 2.13 (s, 3H), 2.09 (s, 3H), 2.03 (s, 3H), 1.96 (s, 3H). **^{13}C NMR** (125 MHz, $CDCl_3$, 25 ºC, δ): 194.46, 170.92, 170.89, 169.74, 168.98, 167.18, 149.14, 138.21, 126.26, 111.57, 92.22, 71.58, 69.95, 69.15, 61.99, 54.31, 39.43, 35.62, 21.03, 20.89, 20.84, 20.79. **HRMS** (ESI-TOF) *m/z* calcd for $C_{22}H_{28}NO_{11}$ [$(M + H)^+$], 482.1657, found, 482.1659.

(2R,3S,4R,5S,6R)-6-(acetoxymethyl)-3-(2-oxo-2-(thiophen-2-yl)ethyl)tetrahydro-2H-pyran-2,4,5-triyl triacetate (3l)

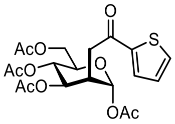

3l

Prepared according to the General Procedure C, the title compound was obtained as a white solid (57.5 mg, 0.126 mmol, 63% yield, axial: equatorial = 7.0:1). **R_f** = 0.45 [Hexanes: EtOAc 2:1 (v/v)].

^{1}H NMR (700 MHz, $CDCl_3$, 25 ºC, δ): 7.79 (d, *J* = 3.6 Hz, 1H), 7.68 (d, *J* = 4.8 Hz, 1H), 7.17 (t, *J* = 4.3 Hz, 1H), 6.09 (s, 1H, *H1*), 5.54 (dd, *J* = 9.9, 5.4 Hz, 1H, *H3*), 5.22 (t, *J* = 10.0 Hz, 1H, *H4*), 4.93 (s, 2H), 4.24 (dd, *J* = 12.3, 4.5 Hz, 1H, *H6*), 4.10 (d, *J* = 12.3 Hz, 1H, *H7*), 4.04 (dd, *J* = 6.1, 3.9 Hz, 1H, *H5*), 3.33 (dd, *J* = 16.8, 4.3 Hz, 1H), 3.12 (dd, *J* = 11.0, 6.7 Hz, 1H, *H2*), 3.07 (dd, *J* = 16.8, 8.4 Hz, 1H), 2.17 (s, 3H), 2.11 (s, 3H), 2.05 (s, 2H), 1.95 (s, 3H). **^{13}C NMR** (175 MHz, $CDCl_3$, 25 ºC, δ): 189.83, 170.73, 170.00, 169.85, 168.78, 143.70, 134.40, 132.36, 128.46, 93.30, 70.21, 69.36, 66.24, 62.40, 37.90, 35.17, 21.13, 20.90, 20.88, 20.84. **HRMS** (ESI-TOF) *m/z* calcd for $C_{20}H_{25}O_{10}S$ [$(M + H)^+$], 457.1163, found, 457.1158.

(2R,3S,4R,5S,6R)-6-((benzoyloxy)methyl)-3-(2-oxopropyl)tetrahydro-2H-pyran-2,4,5-triyl tribenzoate (3m)

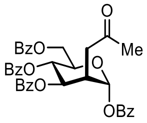

3m

Prepared according to the General Procedure C, the title compound was obtained as a white solid (71.0 mg, 0.112 mmol, 56% yield, axial: equatorial = 5.1:1). **R_f** = 0.40 [Hexanes: EtOAc 2:1 (v/v)]. The analytic amount of axial and equatorial diastereomers were separated by the analytical HPLC column.

Data for axial product (**3m-*ax***): t_R = 6.5 min, 20% (v/v) isopropanol in hexane at the flow rate of 1.0 ml/min.

^{1}H NMR (500 MHz, $CDCl_3$, 25 ºC, δ): 8.20 – 8.17 (m, 2H), 8.02 – 7.98 (m, 2H), 7.94 (dd, *J* = 12.8, 4.8 Hz, 4H), 7.65 (t, *J* = 7.4 Hz, 1H), 7.52 (dt, *J* = 8.8, 7.6 Hz, 5H), 7.42 – 7.34 (m, 6H), 6.35 (d, *J* = 1.6 Hz, 1H, *H1*), 6.02 (dd, *J* = 9.7, 5.2 Hz, 1H, *H3*), 5.79 (t, *J* = 9.8 Hz, 1H, *H4*), 4.58 (dd, *J* = 12.6, 3.2 Hz, 1H, *H6*), 4.50 – 4.40 (m, 2H, *H5, H7*), 3.39 (ddd, *J* = 9.2, 5.8, 1.8 Hz, 1H, *H2*), 3.04 (dd, *J* = 18.3, 4.2 Hz, 1H), 2.89

(dd, *J* = 18.3, 9.2 Hz, 1H), 2.18 (s, 3H). **^{13}C NMR** (125 MHz, $CDCl_3$, 25 ºC, δ): 205.06, 133.90, 133.68, 133.60, 133.24, 130.20, 129.95, 129.80, 129.34, 129.22, 128.94, 128.85, 128.67, 128.59, 128.51, 93.94, 70.81, 70.63, 66.94, 63.10, 39.37, 37.73, 30.41. **HRMS** (ESI-TOF) *m/z* calcd for $C_{37}H_{36}NO_{10}$ [$(M + NH_4)^+$], 654.2334, found, 654.2327.

Data for equatorial product(**3m-*eq***): t_R = 8.3 min, 20% (v/v) isopropanol in hexane at the flow rate of 1.0 ml/min. **^{1}H NMR** (700 MHz, $CDCl_3$, 25 ºC, δ): 8.16 (d, *J* = 7.2 Hz, 2H), 8.00 (d, *J* = 7.2 Hz, 2H), 7.92 (d, *J* = 7.3 Hz, 2H), 7.88 (d, *J* = 7.4 Hz, 2H), 7.68 (t, *J* = 7.4 Hz, 1H), 7.58 – 7.50 (m, 4H), 7.47 (t, *J* = 7.4 Hz, 1H), 7.38 (dt, *J* = 15.3, 7.8 Hz, 4H), 7.32 (t, *J* = 7.8 Hz, 2H), 6.63 (d, *J* = 3.1 Hz, 1H, *H1*), 5.85 (dd, *J* = 11.2, 9.7 Hz, 1H, *H3*), 5.74 (t, *J* = 9.8 Hz, 1H, *H4*), 4.55 (dd, *J* = 12.2, 2.6 Hz, 1H, *H6*), 4.51 – 4.46 (m, 1H, *H5*), 4.43 (dd, *J* = 12.2, 4.3 Hz, 1H, *H7*), 3.19 (ddd, *J* = 11.5, 8.1, 4.8 Hz, 1H, *H2*), 2.62 (qd, *J* = 18.4, 6.5 Hz, 2H), 2.03 (s, 3H). **^{13}C NMR** (175 MHz, $CDCl_3$, 25 ºC, δ): 205.33, 166.61, 166.28, 165.43, 164.69, 134.11, 133.72, 133.50, 133.19, 130.12, 129.96, 129.91, 129.75, 129.00, 128.84, 128.65, 128.51, 128.48, 93.11, 72.00, 70.82, 70.09, 62.90, 40.79, 39.92, 30.39. **HRMS** (ESI-TOF) *m/z* calcd for $C_{37}H_{36}NO_{10}$ [$(M + NH_4)^+$], 654.2334, found, 654.2327.

(2R,3S,4R,5S,6R)-6-((benzoyloxy)methyl)-3-(2-oxopentyl)tetrahydro-2H-pyran-2,4,5-triyl tribenzoate (3n)

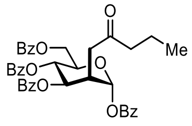

3n

Prepared according to the General Procedure C, the title compound was obtained as a white solid (67.0 mg, 0.101 mmol, 50% yield, axial: equatorial = 4.8:1). **R_f** = 0.4 [Hexanes: EtOAc 2:1 (v/v)]. The analytic amount of axial and equatorial diastereomers were separated by the analytical HPLC column.

Data for axial product (**3n-*ax***): t_R = 11.5 min, 10% (v/v) isopropanol in hexane at the flow rate of 1.0 ml/min. mp = 175-176°C. $[\alpha]_D^{25}$ = +77.7 (*c* = 0.97, MeCN). **^{1}H NMR** (700 MHz, $CDCl_3$, 25 ºC, δ): 8.19 (d, *J* = 7.0 Hz, 2H), 8.01 (d, *J* = 7.0 Hz, 2H), 7.96 (d, *J* = 7.2 Hz, 2H), 7.93 (d, *J* = 7.1 Hz, 2H), 7.65 (t, *J* = 7.1 Hz, 1H), 7.56 – 7.49 (m, 5H), 7.38 (dt, *J* = 14.3, 7.6 Hz, 6H), 6.34 (s, 1H, *H1*), 6.06 – 5.97 (m, 1H, *H3*), 5.80 (t, *J* = 9.4 Hz, 1H, *H4*), 4.58 (d, *J* = 11.3 Hz, 1H, *H6*), 4.44 (t, *J* = 10.1 Hz, 2H, *H7*, *H5*), 3.41 (s, 1H, *H2*), 3.00 (d, *J* = 18.1 Hz, 1H), 2.83 (dd, *J* = 18.0, 9.0 Hz, 1H), 2.41 (t, *J* = 7.0 Hz, 2H), 1.56 – 1.41 (m, 2H), 0.85 (t, *J* = 6.8 Hz, 3H). **^{13}C NMR** (175 MHz, $CDCl_3$, 25 ºC, δ): 207.44, 166.13, 165.73, 165.55, 164.48, 133.90, 133.69, 133.57, 133.25, 130.21, 129.96, 129.81, 129.79, 129.35, 129.26, 128.95, 128.85, 128.65, 128.60, 128.52, 94.09, 70.82, 70.68, 66.89, 63.06, 45.31, 38.43, 37.70, 17.23, 13.78. **HRMS** (ESI-TOF) *m/z* calcd for $C_{39}H_{40}NO_{10}$ [$(M + NH_4)^+$], 682.2647, found, 682.2633.

Data for equatorial product(**3n-*eq***): t_R = 9.8 min, 10% (v/v) isopropanol in hexane at the flow rate of 1.0 ml/min. **^{1}H NMR** (700 MHz, $CDCl_3$, 25 ºC, δ): 8.16 (d, *J* = 6.9 Hz, 2H), 7.99 (d, *J* = 7.0 Hz, 2H), 7.92 (d, *J* = 7.2 Hz, 2H), 7.88 (d, *J* = 7.1 Hz, 2H), 7.67 (d, *J* = 6.6 Hz, 1H), 7.58 – 7.49 (m, 4H), 7.49 – 7.45 (m, 1H), 7.42 – 7.34 (m, 4H), 7.32 (t, *J* = 7.2 Hz, 2H), 6.61 (s, 1H, *H1*), 6.02 – 5.80 (m, 1H, *H3*), 5.74 (t, *J* = 9.8 Hz, 1H, *H4*), 4.54 (d, *J* = 12.3 Hz, 1H, *H6*), 4.47 (d, *J* = 9.6 Hz, 1H, *H5*), 4.43 (d, *J* = 12.2 Hz, 1H, *H7*), 3.22 (s, 1H, *H2*), 2.57 (q, *J* = 18.4 Hz, 2H), 2.35 – 2.14 (m, 2H), 1.49 – 1.32 (m, 2H), 0.73 (t, *J* = 7.1 Hz, 3H). **^{13}C NMR** (175 MHz, $CDCl_3$, 25 ºC, δ): 207.61, 166.60, 166.28, 165.43, 164.66, 134.08, 133.70, 133.49, 133.18, 130.11, 129.98, 129.91, 128.99, 128.62, 128.50, 128.48, 93.14, 72.12, 70.80, 70.13, 62.92, 45.18, 39.99, 39.84, 17.08, 13.60. **HRMS** (ESI-TOF) *m/z* calcd for $C_{39}H_{40}NO_{10}$ [$(M + NH_4)^+$], 682.2647, found, 682.2638.

(2R,3S,4R,5S,6R)-6-((benzoyloxy)methyl)-3-(3,3-dimethyl-2-oxobutyl)tetrahydro-2H-pyran-2,4,5-triyl tribenzoate (3o)

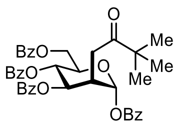

3o

Prepared according to the General Procedure C, the title compound was obtained as a white solid (86.5 mg, 0.127 mmol, 64% yield, axial: equatorial = 15:1). **R_f** = 0.50 [Hexanes: EtOAc 2:1 (v/v)]. The analytic amount of axial diastereomer was separated by the analytical HPLC column.

Data for axial product (**3o-*ax***): t_R = 10.4 min, 5% (v/v) isopropanol in hexane at the flow rate of 1.0 ml/min. mp = 115-116°C. $[\alpha]_D^{25}$ = +61.7 (*c* = 6.55, MeCN). **^{1}H NMR** (500 MHz, $CDCl_3$, 25 ºC, δ): 8.22 – 8.17 (m, 2H), 8.05 – 8.01 (m, 2H), 7.98 – 7.94 (m, 2H), 7.94 – 7.91 (m, 2H), 7.65 (t, *J* = 7.4 Hz, 1H), 7.57 – 7.49 (m, 5H), 7.43 – 7.35 (m, 6H), 6.29 (d, *J* = 1.7 Hz, 1H, *H1*), 6.04 (dd, *J* = 9.8, 5.2 Hz, 1H, *H3*), 5.86 (t, *J* = 9.8 Hz, 1H, *H4*), 4.64 – 4.55 (m, 1H, *H6*), 4.47 – 4.36 (m, 2H, *H7*, *H5*), 3.39 (ddd, *J* = 9.1, 5.8, 1.8 Hz, 1H, *H2*), 3.07 (dd, *J* = 18.2, 4.1 Hz, 1H), 2.93 (dd, *J* = 18.2, 9.1 Hz, 1H), 1.11 (s, 9H). **^{13}C NMR** (125 MHz, $CDCl_3$, 25 ºC, δ): 212.55, 166.05, 165.74, 165.58, 164.47, 133.87, 133.68, 133.51, 133.25, 130.19, 129.95, 129.90, 129.86, 129.77, 129.76, 129.39, 129.33, 128.96, 128.84, 128.62, 128.60, 128.53, 94.15, 70.88, 70.73, 66.78, 62.80, 44.39, 37.86, 32.44, 26.44. **HRMS** (ESI-TOF) *m/z* calcd for $C_{40}H_{42}NO_{10}$ [$(M + NH_4)^+$], 696.2803, found, 696.2794.

(2R,3S,4R,5S,6R)-6-((benzoyloxy)methyl)-3-(2-oxoethyl)tetrahydro-2H-pyran-2,4,5-triyl tribenzoate (3p)

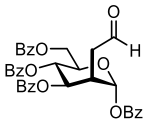

3p

Prepared according to the General Procedure C, the title compound was obtained as a white solid (101.5 mg, 0.163 mmol, 82% yield, axial: equatorial = 5.1:1). $\mathbf{R_f}$ = 0.40 [Hexanes: EtOAc 2:1 (v/v)].

^{1}H NMR (700 MHz, $CDCl_3$, 25 ºC, δ): 9.70 (s, 1H), 8.20 (d, *J* = 7.4 Hz, 2H), 8.00 (d, *J* = 7.4 Hz, 2H), 7.96 (d, *J* = 7.4 Hz, 2H), 7.90 (d, *J* = 7.5 Hz, 2H), 7.72 (dd, *J* = 10.7, 4.0 Hz, 1H), 7.61 (dt, *J* = 15.3, 7.5 Hz, 3H), 7.56 (t, *J* = 7.3 Hz, 2H), 7.48 (dd, *J* = 13.2, 5.5 Hz, 2H), 7.43 – 7.37 (m, 4H), 6.37 (s, 1H, *H1*), 6.03 (dd, *J* = 9.6, 5.3 Hz, 1H, *H3*), 5.82 (t, *J* = 9.9 Hz, 1H, *H4*), 4.58 (d, *J* = 9.9 Hz, 1H, *H6*), 4.51 – 4.45 (m, 2H, *H7*, *H5*), 3.38 – 3.37 (m, 1H, *H2*), 3.17 (dd, *J* = 18.5, 4.9 Hz, 1H), 2.92 (dd, *J* = 18.5, 8.0 Hz, 1H). **^{13}C NMR** (175 MHz, $CDCl_3$, 25 ºC, δ): 200.94, 166.60, 166.31, 166.04, 165.21, 134.83, 134.57, 134.48, 134.24, 130.73, 130.42, 130.35, 130.33, 130.29, 130.19, 130.08, 129.77, 129.57, 129.53, 129.49, 129.48, 129.25, 94.84, 71.59, 71.54, 67.47, 63.45, 40.60, 37.18. **HRMS** (ESI-TOF) *m/z* calcd for $C_{36}H_{34}NO_{10}$ [(M + $NH_4)^+$], 640.2177, found, 640.2167.

2-((2R,3S,4R,5S,6R)-2,4,5-tris(benzoyloxy)-6-((benzoyloxy)methyl)tetrahydro-2H-pyran-3-yl)acetic acid (3q)

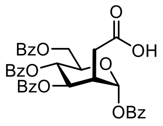

3q

Prepared according to the General Procedure C, the title compound was obtained as a white solid (68.0 mg, 0.106 mmol, 53% yield, axial: equatorial = 4.3:1). $\mathbf{R_f}$ = 10 [Hexanes: EtOAc 2:1 (v/v)]. The analytic amount of axial and equatorial diastereomers were separated by the analytical HPLC column.

Data for axial product (**3q-*ax***): t_R = 6.5 min, 20% (v/v) isopropanol in hexane at the flow rate of 1.0 ml/min. **^{1}H NMR** (500 MHz, $CDCl_3$, 25 ºC, δ): 8.19 – 8.16 (m, 2H), 8.03 – 7.98 (m, 2H), 7.94 (d, *J* = 7.3 Hz, 4H), 7.65 (t, *J* = 7.4 Hz, 1H), 7.58 – 7.51 (m, 3H), 7.50 (t, *J* = 7.4 Hz, 1H), 7.47 – 7.38 (m, 3H), 7.34 (dd, *J* = 13.7, 7.5 Hz, 4H), 6.43 (d, *J* = 1.0 Hz, 1H, *H1*), 6.02 (dd, *J* = 9.9, 5.3 Hz, 1H, *H3*), 5.84 (t, *J* = 9.8 Hz, 1H, *H4*), 4.68 – 4.53 (m, 1H, *H6*), 4.52 – 4.36 (m, 2H, *H7*, *H5*), 3.26 – 3.16 (m, 1H, *H2*), 2.89 (dd, *J* = 17.2, 5.9 Hz, 1H), 2.69 (dd, *J* = 17.3, 8.3 Hz, 1H). **^{13}C NMR** (125 MHz, $CDCl_3$, 25 ºC, δ): 176.16, 166.21, 165.52, 165.51, 164.40, 133.99, 133.66, 133.58, 133.29, 130.20, 129.95, 129.87, 129.81, 129.72, 129.21, 129.01, 128.89, 128.69, 128.58, 93.94, 70.98, 70.53, 66.30, 62.90, 39.12, 30.79. **HRMS** (ESI-TOF) *m/z* calcd for $C_{36}H_{34}NO_{11}$ [(M + $NH_4)^+$], 656.2126, found, 656.2118.

Data for equatorial product(**3q-*eq***): t_R = 8.3 min, 20% (v/v) isopropanol in hexane at the flow rate of 1.0 ml/min. **^{1}H NMR** (700 MHz, $CDCl_3$, 25 ºC, δ): 8.15 (d, *J* = 7.4 Hz, 2H), 7.98 (d, *J* = 7.3 Hz, 2H), 7.93 (d, *J* = 8.4 Hz, 2H), 7.88 (d, *J* = 7.4 Hz, 2H), 7.66 (t, *J* = 7.3 Hz, 1H), 7.54 (dt, *J* = 15.5, 7.6 Hz, 3H), 7.47 (dd, *J* = 15.2, 7.6 Hz, 2H), 7.43 – 7.30 (m, 6H), 6.68 (d, *J* = 2.7 Hz, 1H, *H1*), 5.92 – 5.87 (m, 1H, *H3*), 5.71 (t, *J* = 9.8 Hz, 1H, *H4*), 4.54 (d, *J* = 12.1 Hz, 1H, *H6*), 4.49 – 4.36 (m, 2H, *H7*, *H5*), 3.01 (d, *J* = 6.3 Hz, 1H, *H2*), 2.55 – 2.46 (m, 2H). **^{13}C NMR** (175 MHz, $CDCl_3$, 25 ºC, δ): 174.48, 166.32, 166.24, 165.46, 164.68, 134.15, 133.64, 133.54, 133.19, 130.21, 130.15, 129.95, 129.93, 129.89, 129.82, 129.74, 129.03, 128.99, 128.93, 128.89, 128.68, 128.60, 128.52, 128.48, 92.70, 71.83, 70.76, 70.06, 62.86, 41.25, 31.91. **HRMS** (ESI-TOF) *m/z* calcd for $C_{36}H_{34}NO_{11}$ [$(M + NH_4)^+$], 656.2126, found, 656.2117.

(2R,3S,4R,5S,6R)-3-(2-(9H-carbazol-9-yl)-2-oxoethyl)-6-((benzoyloxy)methyl)tetrahydro-2H-pyran-2,4,5-triyl tribenzoate (3r)

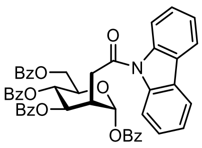

3r

Prepared according to the General Procedure C, the title compound was obtained as a white solid (48.0 mg, 0.060 mmol, 30% yield, axial: equatorial = 5.0:1). **R*f*** = 0.60 [Hexanes: EtOAc 3:1 (v/v)]. The analytic amount of axial diastereomer was separated by the analytical HPLC column.

Data for axial product (**3r-*ax***): t_R = 12.9 min, 20% (v/v) isopropanol in hexane at the flow rate of 1.0 ml/min. **^{1}H NMR** (700 MHz, $CDCl_3$, 25 ºC, δ): 8.24 (d, *J* = 7.1 Hz, 2H), 8.13 (d, *J* = 7.7 Hz, 2H), 8.01 (dd, *J* = 6.5, 2.3 Hz, 2H), 7.97 (dd, *J* = 8.1, 7.3 Hz, 4H), 7.88 (d, *J* = 7.3 Hz, 2H), 7.68 (t, *J* = 7.4 Hz, 1H), 7.57 (t, *J* = 7.7 Hz, 2H), 7.52 (dd, *J* = 12.9, 7.3 Hz, 2H), 7.45 – 7.35 (m, 7H), 7.29 (t, *J* = 7.8 Hz, 2H), 7.27 – 7.23 (m, 2H), 6.70 (s, 1H, *H1*), 6.23 (dd, *J* = 9.9, 5.3 Hz, 1H, *H3*), 5.99 (t, *J* = 9.9 Hz, 1H, *H4*), 4.59 (dd, *J* = 12.2, 2.5 Hz, 1H, *H5*), 4.56 – 4.53 (m, 1H, *H5*), 4.50 (dd, *J* = 12.2, 3.9 Hz, 1H, *H7*), 3.84 – 3.76 (m, 2H, *H2*), 3.57 (dd, *J* = 16.4, 8.5 Hz, 1H). **^{13}C NMR** (175 MHz, $CDCl_3$, 25 ºC, δ): 170.24, 166.19, 165.71, 165.62, 164.48, 138.46, 134.00, 133.74, 133.56, 133.18, 130.28, 129.97, 129.74, 129.30, 129.08, 128.91, 128.64, 128.62, 128.60, 127.81, 126.74, 124.07, 120.08, 116.48, 94.20, 70.85, 70.58, 66.72, 62.78, 39.02, 35.47. **HRMS** (ESI-TOF) *m/z* calcd for $C_{48}H_{41}N_2O_{10}$ [$(M + NH_4)^+$], 805.2756, found, 805.2741.

(2R,3S,4R,5R,6R)-6-(acetoxymethyl)-3-(2-oxo-2-phenylethyl)tetrahydro-2H-pyran-2,4,5-triyl triacetate (4b)

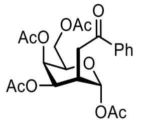

4b

Prepared according to the General Procedure C, the title compound was obtained as a white solid (61.0 mg, 0.136 mmol, 68% yield, axial: equatorial = 2.0:1). **R*f*** = 0.30 [Hexanes: EtOAc 3:1 (v/v)]. The analytic amount of axial and equatorial diastereomers were separated by the analytical HPLC column.

Data for axial product (**4b-*ax***): t_R = 9.1 min, 10% (v/v) isopropanol in hexane at the flow rate of 1.0 ml/min. **^{1}H NMR** (500 MHz, $CDCl_3$, 25 ºC, δ): 8.07 – 7.91 (m, 2H), 7.60 (t, *J* = 7.4 Hz, 1H), 7.50 (t, *J* = 7.7 Hz, 2H), 6.12 (s, 1H, *H1*), 5.51 (dd, *J* = 5.7, 3.5 Hz, 1H, *H3*), 5.40 (s, 1H, *H4*), 4.31 (td, *J* = 6.7, 1.4 Hz, 1H, *H5*), 4.18 (dd, *J* = 11.2, 6.8 Hz, 1H, *H6*), 4.09 (dd, *J* = 11.2, 6.8 Hz, 1H, *H7*), 3.40 (qd, *J* = 18.3, 6.6 Hz, 2H), 2.94 (dt, *J* = 9.6, 4.9 Hz, 1H, *H2*), 2.21 (s, 3H), 2.16 (s, 3H), 2.03 (s, 3H), 1.97 (s, 3H). **^{13}C NMR** (125 MHz, $CDCl_3$, 25 ºC, δ): 197.80, 170.63, 169.83, 169.73, 168.83, 136.84, 133.60, 128.90, 128.05, 94.02, 68.36, 66.54, 66.05, 61.62, 35.57, 35.40, 21.16, 21.03, 20.88, 20.82. **HRMS** (ESI-TOF) *m/z* calcd for $C_{22}H_{30}NO_{10}$ [$(M + NH_4)^+$], 468.1864, found, 468.1858.

Data for equatorial product(**4b-*eq***): t_R = 12.1 min, 10% (v/v) isopropanol in hexane at the flow rate of 1.0 ml/min. **^{1}H NMR** (500 MHz, $CDCl_3$, 25 ºC, δ): 7.96 – 7.86 (m, 2H), 7.58 (t, *J* = 7.4 Hz, 1H), 7.47 (t, *J* = 7.7 Hz, 2H), 6.39 (d, *J* = 3.3 Hz, 1H, *H1*), 5.38 (d, *J* = 1.6 Hz, 1H, *H4*), 5.19 (dd, *J* = 12.1, 3.0 Hz, 1H, *H3*), 4.27 (t, *J* = 6.8 Hz, 1H, *H5*), 4.11 (m, 2H, *H6*, *H7*), 3.20 (ddd, *J* = 12.1, 8.3, 4.4 Hz, 1H, *H2*), 3.03 (dd, *J* = 17.3, 4.7 Hz, 1H), 2.81 (dd, *J* = 17.3, 8.5 Hz, 1H), 2.15 (s, 3H), 2.09 (s, 3H), 2.03 (s, 3H), 1.88 (s, 3H). **^{13}C NMR** (125 MHz, $CDCl_3$, 25 ºC, δ): 197.28, 170.63, 170.56, 169.10, 136.67, 133.63, 128.90, 128.14, 92.94, 69.35, 68.61, 66.19, 61.79, 35.33, 34.40, 21.02, 20.86, 20.85, 20.76. **HRMS** (ESI-TOF) *m/z* calcd for $C_{22}H_{30}NO_{10}$ [$(M + NH_4)^+$], 468.1864, found, 468.1858.

(2R,3S,4R,5R,6R)-6-((benzyloxy)methyl)-3-(2-oxo-2-phenylethyl)tetrahydro-2H-pyran-2,4,5-triyl triacetate (4c)

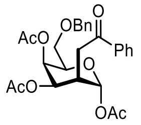

4c

Prepared according to the General Procedure C, the title compound was obtained as a white solid (61.5 mg, 0.123 mmol, 62% yield, axial: equatorial = 2.0:1). **R*f*** = 0.35 [Hexanes: EtOAc 3:1 (v/v)]. The analytic amount of axial and equatorial diastereomers were separated by the analytical HPLC column.

Data for axial product (**4c-*ax***): t_R = 7.6 min, 10% (v/v) isopropanol in hexane at the flow rate of 1.0 ml/min. **^{1}H NMR** (500 MHz, $CDCl_3$, 25 ºC, δ): 7.95 (d, *J* = 7.5 Hz, 2H), 7.59 (t, *J* = 7.4 Hz, 1H), 7.48 (t, *J* = 7.7 Hz,

2H), 7.38 – 7.31 (m, 2H), 7.31 – 7.24 (m, 3H), 6.10 (s, 1H, *H1*), 5.50 (dd, *J* = 10.5, 4.8 Hz, 2H, *H3*, *H4*), 4.55 (d, *J* = 11.9 Hz, 1H, *H6*), 4.40 (d, *J* = 11.9 Hz, 1H, *H7*), 4.26 (t, *J* = 6.1 Hz, 1H, *H5*), 3.55 – 3.46 (m, 2H), 3.42 (dd, *J* = 18.3, 3.8 Hz, 1H), 3.34 (dd, *J* = 18.3, 9.3 Hz, 1H), 2.95 – 2.86 (m, 1H, *H2*), 2.15 (s, 3H), 2.10 (s, 3H), 1.96 (s, 3H). **^{13}C NMR** (175 MHz, $CDCl_3$, 25 ºC, δ): 198.01, 169.82, 169.80, 169.08, 137.81, 137.00, 133.62, 128.96, 128.70, 128.22, 128.15, 128.08, 94.24, 73.80, 69.78, 67.84, 67.24, 66.35, 35.81, 35.62, 21.32, 21.12, 21.01. **HRMS** (ESI-TOF) *m/z* calcd for $C_{27}H_{34}NO_9$ [$(M + NH_4)^+$], 516.2228, found, 516.2224.

Data for equatorial product(**4c-*eq***): t_R = 7.0 min, 10% (v/v) isopropanol in hexane at the flow rate of 1.0 ml/min. **^{1}H NMR** (700 MHz, $CDCl_3$, 25 ºC, δ): 7.90 (d, *J* = 7.5 Hz, 2H), 7.57 (t, *J* = 7.0 Hz, 1H), 7.46 (t, *J* = 7.3 Hz, 2H), 7.33 (t, *J* = 6.9 Hz, 2H), 7.30 – 7.23 (m, 3H), 6.36 (s, 1H, *H1*), 5.47 (s, 1H, *H4*), 5.19 (d, *J* = 12.1 Hz, 1H, *H3*), 4.55 (d, *J* = 11.9 Hz, 1H, *H6*), 4.40 (d, *J* = 11.9 Hz, 1H, *H7*), 4.22 (s, 1H, *H5*), 3.53 – 3.48 (m, 1H, *H2*), 3.48 – 3.38 (m, 1H, *H2*), 3.17 (s, 1H), 3.01 (d, *J* = 17.4 Hz, 1H), 2.80 (dd, *J* = 17.3, 8.3 Hz, 1H), 2.07 (s, 3H), 2.04 (s, 3H), 1.86 (s, 3H). **^{13}C NMR** (175 MHz, $CDCl_3$, 25 ºC, δ): 197.45, 178.07, 170.60, 170.55, 170.49, 170.39, 169.27, 156.70, 137.67, 137.16, 136.70, 134.75, 133.58, 131.85, 128.88, 128.76, 128.60, 128.57, 128.14, 128.09, 127.98, 93.03, 73.70, 69.95, 69.57, 67.73, 66.71, 35.40, 34.53, 21.08, 20.85, 20.79. **HRMS** (ESI-TOF) *m/z* calcd for $C_{27}H_{31}O_9$ [$(M + H)^+$], 499.1963, found, 499.1960.

(2R,3S,4R,5S,6R)-6-(((tert-butyldiphenylsilyl)oxy)methyl)-3-(2-oxo-2-phenylethyl)tetrahydro-2H-pyran-2,4,5-triyl triacetate (4d)

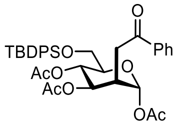

4d

Prepared according to the General Procedure C, the title compound was obtained as a white solid (82.0 mg, 0.127 mmol, 64% yield, axial: equatorial = 4.7:1). **R*f*** = 0.50 [Hexanes: EtOAc 3:1 (v/v)]. The analytic amount of axial and equatorial diastereomers were separated by the analytical HPLC column.

Data for axial product (**4d-*ax***): t_R = 5.3 min, 20% (v/v) isopropanol in hexane at the flow rate of 1.0 ml/min. **^{1}H NMR** (500 MHz, $CDCl_3$, 25 ºC, δ): 7.98 (d, *J* = 7.3 Hz, 2H), 7.70 – 7.66 (m, 2H), 7.65 – 7.62 (m, 2H), 7.58 (t, *J* = 7.4 Hz, 1H), 7.45 (t, *J* = 7.7 Hz, 2H), 7.43 – 7.32 (m, 6H), 6.14 (d, *J* = 0.9 Hz, 1H, *H1*), 5.63 – 5.42 (m, 2H, *H3*, *H4*), 3.86 (d, *J* = 8.8 Hz, 1H, *H5*), 3.76 (dd, *J* = 11.5, 1.7 Hz, 1H, *H6*), 3.68 (dd, *J* = 11.6, 3.3 Hz, 1H, *H7*), 3.44 (dd, *J* = 17.9, 4.1 Hz, 1H), 3.22 (dd, *J* = 17.9, 8.7 Hz, 1H), 3.16 – 3.10 (m, 1H, *H2*), 2.14 (s, 3H), 1.95 (s, 3H), 1.94 (s, 3H) , 1.08 (s, 9H). **^{13}C NMR** (125 MHz, $CDCl_3$, 25 ºC, δ): 197.31, 170.17, 169.77, 168.91, 136.65, 135.85, 135.81, 133.59, 133.15, 133.03, 129.89, 129.84, 128.82, 128.29, 127.86,

127.82, 93.48, 72.83, 69.98, 66.14, 62.02, 37.89, 34.48, 26.92, 21.19, 21.00, 20.82, 19.40. **HRMS** (ESI-TOF) *m/z* calcd for $C_{36}H_{46}NO_9Si$ [$(M + NH_4)^+$], 664.2936, found, 664.2929.

Data for equatorial product(**4d-*eq***): t_R = 4.8 min, 20% (v/v) isopropanol in hexane at the flow rate of 1.0 ml/min. **^{1}H NMR** (500 MHz, $CDCl_3$, 25 ºC, δ): 7.91 (d, *J* = 7.6 Hz, 2H), 7.66 (d, *J* = 6.3 Hz, 2H), 7.63 (d, *J* = 6.6 Hz, 2H), 7.58 (dd, *J* = 9.2, 5.5 Hz, 1H), 7.47 (t, *J* = 7.7 Hz, 2H), 7.44 – 7.33 (m, 6H), 6.39 (d, *J* = 2.9 Hz, 1H, *H1*), 5.32 (dd, *J* = 13.3, 7.0 Hz, 1H, *H3*), 5.23 (t, *J* = 9.7 Hz, 1H, *H4*), 3.91 (dt, *J* = 10.0, 3.1 Hz, 1H, *H6*), 3.73 – 3.66 (m, 2H, *H5*, *H7*), 3.02 – 2.91 (m, 2H, *H2*), 2.88 (td, *J* = 9.5, 2.7 Hz, 1H), 2.07 (s, 3H), 1.92 (s, 3H), 1.90 (s, 3H), 1.04 (s, 9H). **^{13}C NMR** (125 MHz, $CDCl_3$, 25 ºC, δ): 197.03, 171.21, 169.61, 169.02, 136.52, 135.86, 135.84, 133.64, 133.36, 133.25, 129.83, 129.81, 128.91, 128.27, 128.21, 127.82, 92.40, 72.67, 72.26, 69.54, 62.56, 39.70, 35.87, 26.86, 21.03, 20.87, 20.77, 19.36. **HRMS** (ESI-TOF) *m/z* calcd for $C_{36}H_{43}O_9Si$ [$(M + H)^+$], 647.2671, found, 647.2659.

(2R,4aR,6R,7S,8R,8aS)-2-(4-methoxyphenyl)-7-(2-oxo-2-phenylethyl)hexahydropyrano[3,2-d][1,3]dioxine-6,8-diyl diacetate (4e)

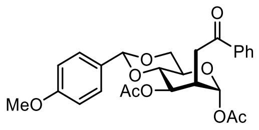

4e

Prepared according to the General Procedure C, the title compound was obtained as a white solid (73 mg, 0.15 mmol, 75% yield, axial: equatorial = 10:1). **R_f** = 0.45 [Hexanes: EtOAc 3:1 (v/v)]. The analytic amount of axial diastereomer was separated by the analytical HPLC column.

Data for axial product (**4e-*ax***): t_R = 19.5 min, 10% (v/v) isopropanol in hexane at the flow rate of 1.0 ml/min. **^{1}H NMR** (700 MHz, $CDCl_3$, 25 ºC, δ): 8.00 (d, *J* = 7.4 Hz, 2H), 7.61 (t, *J* = 7.4 Hz, 1H), 7.51 (t, *J* = 7.7 Hz, 2H), 7.39 (d, *J* = 8.7 Hz, 2H), 6.89 (d, *J* = 8.7 Hz, 2H), 6.03 (s, 1H, *H1*), 5.64 (dd, *J* = 10.4, 5.7 Hz, 1H), 5.54 (s, 1H), 4.27 (dd, *J* = 10.4, 4.8 Hz, 1H), 4.02 (td, *J* = 9.8, 4.7 Hz, 1H), 3.88 (t, *J* = 10.0 Hz, 1H), 3.81 (s, 3H), 3.79–3.76 (m, 1H), 3.46 (dd, *J* = 17.8, 4.1 Hz, 1H), 3.28–3.25 (m, 1H, *H2*), 3.14 (dd, *J* = 17.8, 8.7 Hz, 1H), 2.20 (s, 3H), 1.98 (s, 3H). **^{13}C NMR** (175 MHz, $CDCl_3$, 25 ºC, δ): 196.74, 169.57, 169.02, 160.18, 136.45, 133.55, 129.45, 128.77, 128.05, 127.48, 113.62, 101.93, 94.00, 68.66, 68.15, 65.72, 55.29, 37.81, 34.77, 21.07, 20.94. **HRMS** (ESI-TOF) *m/z* calcd for $C_{26}H_{28}O_9$ [$(M + H)^+$], 485.1812, found, 485.1807.

(2R,3S,4R,5R)-3-(2-oxo-2-phenylethyl)tetrahydro-2H-pyran-2,4,5-triyl triacetate (4f)

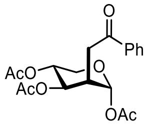

4f

Prepared according to the General Procedure C, the title compound was obtained as a white solid (51.5 mg, 0.136 mmol, 68% yield, axial: equatorial = 6.5:1). **R*f*** = 0.26 [Hexanes: EtOAc 3:1 (v/v)]. The analytic amount of axial and equatorial diastereomers were separated by the analytical HPLC column.

Data for axial product (**4f-*ax***): t_R = 12.6 min, 5% (v/v) isopropanol in hexane at the flow rate of 1.0 ml/min. **^{1}H NMR** (700 MHz, $CDCl_3$, 25 ºC, δ): 7.95-7.97 (m, 2H), 7.58-7.61 (m, 1H), 7.47-7.50 (m, 2H), 5.94 (d, *J* = 3.5 Hz, 1H, *H1*), 5.46 (dd, *J* = 4.2, 7.7 Hz, 1H, *H3*), 5.01 (dt, *J* = 4.9, 7.7 Hz, 1H, *H4*), 3.98 (dd, *J* = 4.9, 11.9 Hz, 1H), 3.79 (dd, *J* = 7.7, 11.9 Hz, 1H), 3.20 (dd, *J* = 5.6, 16.8 Hz, 1H), 3.11-3.15 (m, 1H, *H2*), 3.09 (dd, *J* = 7.0, 16.8 Hz, 1H), 2.11 (s, 3H), 2.10 (s, 3H), 2.01 (s, 3H). **^{13}C NMR** (175 MHz, $CDCl_3$, 25 ºC, δ): 197.00, 170.23, 169.67, 169.35, 136.58, 133.64, 128.87, 128.87, 128.16, 128.16, 93.32, 69.28, 66.73, 62.45, 36.90, 34.36, 29.97, 21.05, 21.02, 20.93. **HRMS** (ESI-TOF) *m/z* calcd for $C_{19}H_{26}NO_8$ [$(M + NH_4)^+$], 396.1653, found, 396.1652.

Data for equatorial product(**4f-*eq***): t_R = 11.5 min, 5% (v/v) isopropanol in hexane at the flow rate of 1.0 ml/min. **^{1}H NMR** (700 MHz, $CDCl_3$, 25 ºC, 7.90-7.92 (m, 2H), 7.57-7.60 (m, 2H), 7.45-7.48 (m, 1H), 6.25 (d, *J* = 2.8 Hz, 1H, *H1*), 5.32 (dd, *J* = 9.8, 10.5 Hz, 1H, *H3*), 5.02 (ddd, *J* = 5.6, 9.1, 10.5 Hz, 1H, *H4*), 3.95 (dd, *J* = 5.6, 11.2 Hz, 1H), 3.69 (t, *J* = 10.5 Hz, 1H), 2.98-3.03 (m, 1H), 2.89-2.97 (m, 2H), 2.10 (s, 3H), 2.03 (s, 3H), 1.96 (s, 3H). **^{13}C NMR** (175 MHz, $CDCl_3$, 25 ºC, δ): 197.01, 170.89, 170.01, 169.22, 136.44, 133.66, 128.88, 128.88, 128.17, 128.17, 92.44, 71.07, 69.76, 61.18, 39.37, 35.68, 20.98, 20.88, 20.88. **HRMS** (ESI-TOF) *m/z* calcd for $C_{19}H_{26}NO_8$ [$(M + NH_4)^+$], 396.1653, found, 396.1652.

(2S,3R,4S,5R,6S)-6-methyl-3-(2-oxo-2-phenylethyl)tetrahydro-2H-pyran-2,4,5-triyl triacetate (4g)

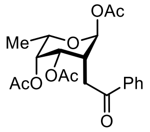

4g

Prepared according to the General Procedure C, the title compound was obtained as a white solid (48.5 mg, 0.123 mmol, 62% yield, axial: equatorial = 3.0:1). **R*f*** = 0.3 [Hexanes: EtOAc 3:1 (v/v)]. The analytic amount of axial and equatorial diastereomers were separated by the analytical HPLC column.

Data for axial product (**4g-*ax***): **^{1}H NMR** (500 MHz, $CDCl_3$, 25 ºC, δ): 7.97 (d, *J* = 7.4 Hz, 2H), 7.59 (t, *J* = 7.4 Hz, 1H), 7.49 (t, *J* = 7.7 Hz, 2H), 6.09 (s, 1H, *H1*), 5.51 (dd, *J* = 5.8, 3.5 Hz, 1H, *H3*), 5.25 (s, 1H, *H4*), 4.22 – 4.12 (m, 1H, *H5*), 3.46 (dd, *J* = 18.3, 3.9 Hz, 1H), 3.36 (dd, *J* = 18.3, 9.2 Hz, 1H), 2.91 (dt, *J* = 9.4, 4.8 Hz, 1H, *H2*), 2.23 (s, 3H), 2.14 (s, 3H), 1.96 (s, 3H), 1.17 (d, *J* = 6.5 Hz, 2H). **^{13}C NMR** (125 MHz,

$CDCl_3$, 25 ºC, δ): 197.97, 170.19, 169.79, 169.07, 136.94, 133.49, 128.85, 128.06, 94.32, 69.77, 66.84, 66.62, 35.77, 35.02, 21.23, 21.04, 20.92, 16.34. **HRMS** (ESI-TOF) *m/z* calcd for $C_{20}H_{28}NO_8$ [$(M + NH_4)^+$], 410.1809, found, 410.1804.

Data for equatorial product(**4g-*eq***): **^{1}H NMR** (500 MHz, $CDCl_3$, 25 ºC, δ): 7.90 (d, *J* = 7.4 Hz, 2H), 7.57 (d, *J* = 9.6 Hz, 1H), 7.48 – 7.44 (m, 2H), 6.34 (d, *J* = 3.4 Hz, 1H, *H1*), 5.22 (s, 1H, *H4*), 5.19 (dd, *J* = 12.0, 3.0 Hz, 1H, *H3*), 4.24 – 4.14 (m, 1H, *H5*), 3.17 (ddd, *J* = 11.9, 8.3, 4.7 Hz, 1H, *H2*), 3.02 (dd, *J* = 17.1, 4.9 Hz, 1H), 2.80 (dd, *J* = 17.2, 8.3 Hz, 1H), 2.17 (s, 3H), 2.07 (s, 3H), 1.86 (s, 3H), 1.16 (d, *J* = 6.3 Hz, 3H). **^{13}C NMR** (125 MHz, $CDCl_3$, 25 ºC, δ): 197.46, 170.93, 170.68, 169.33, 136.76, 133.53, 128.86, 128.13, 93.21, 69.97, 69.47, 67.07, 35.47, 34.18, 21.07, 20.87, 20.78, 16.50. **HRMS** (ESI-TOF) *m/z* calcd for $C_{20}H_{28}NO_8$ [$(M + NH_4)^+$], 410.1809, found, 410.1804.

(2R,3S,4R,5S,6S)-6-(methoxycarbonyl)-3-(2-oxo-2-phenylethyl)tetrahydro-2H-pyran-2,4,5-triyl triacetate (4h)

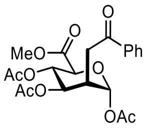

4h

Prepared according to the General Procedure C, the title compound was obtained as a white solid (44.5 mg, 0.102 mmol, 51% yield, axial: equatorial = 5.0:1). **R_f** = 0.30 [Hexanes: EtOAc 2:1 (v/v)]. The analytic amount of axial diastereomer was separated by the analytical HPLC column.

Data for axial product (**4h-*ax***): t_R = 5.5 min, 20% (v/v) isopropanol in hexane at the flow rate of 1.0 ml/min. **^{1}H NMR** (500 MHz, $CDCl_3$, 25 ºC, δ): 8.06 – 7.89 (m, 2H), 7.60 (t, *J* = 7.4 Hz, 1H), 7.50 (t, *J* = 7.7 Hz, 2H), 6.23 (d, *J* = 2.8 Hz, 1H, *H1*), 5.54 (dd, *J* = 8.6, 4.5 Hz, 1H, *H3*), 5.32 (t, *J* = 8.5 Hz, 1H, *H4*), 4.43 (d, *J* = 8.3 Hz, 1H, *H5*), 3.77 (s, 3H), 3.31 (dd, *J* = 21.0, 8.4 Hz, 1H), 3.21 – 3.11 (m, 2H, *H2*), 2.16 (s, 3H), 2.09 (s, 3H), 1.97 (s, 3H). **^{13}C NMR** (125 MHz, $CDCl_3$, 25 ºC, δ): 196.82, 169.95, 169.58, 168.72, 168.16, 136.48, 133.75, 128.92, 128.27, 92.64, 71.83, 68.91, 67.13, 52.99, 36.77, 34.39, 21.09, 20.86. **HRMS** (ESI-TOF) *m/z* calcd for $C_{21}H_{28}NO_{10}$ [$(M + NH_4)^+$], 454.1708, found, 454.1699.

(2R,3R,4S,5R,6S)-2-(acetoxymethyl)-6-(((2R,3S,4R,5S,6R)-4,6-diacetoxy-2-(acetoxymethyl)-5-(2-oxo-2-phenylethyl)tetrahydro-2H-pyran-3-yl)oxy)tetrahydro-2H-pyran-3,4,5-triyl triacetate (4i)

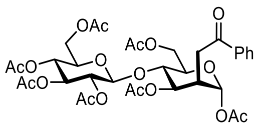

4i

Prepared according to the General Procedure C, the title compound was obtained as a white solid (94.5 mg, 0.128 mmol, 64% yield, axial: equatorial = 6.3:1). **R*f*** = 0.20 [Hexanes: EtOAc 2:1 (v/v)]. The analytic amount of axial diastereomer was separated by the analytical HPLC column.

Data for axial product (**4i-*ax***): t_R = 5.3 min, 20% (v/v) isopropanol in hexane at the flow rate of 1.0 ml/min. **^{1}H NMR** (500 MHz, $CDCl_3$, 25 ºC, δ): 7.95 (d, *J* = 7.5 Hz, 2H), 7.60 (t, *J* = 7.4 Hz, 1H), 7.49 (t, *J* = 7.7 Hz, 2H), 6.03 (d, *J* = 3.3 Hz, 1H, *H1*), 5.57 (dd, *J* = 6.9, 4.5 Hz, 1H), 5.15 (dt, *J* = 31.0, 9.5 Hz, 2H), 5.01 – 4.90 (m, 1H), 4.63 (d, *J* = 8.0 Hz, 1H), 4.32 (dd, *J* = 12.4, 4.0 Hz, 2H), 4.15 (dd, *J* = 12.0, 5.2 Hz, 1H), 4.07 (dd, *J* = 12.3, 2.0 Hz, 1H), 3.96 (dd, *J* = 9.6, 3.3 Hz, 1H), 3.76 (dd, *J* = 9.5, 7.2 Hz, 1H), 3.74 – 3.69 (m, 1H), 3.24 (dd, *J* = 16.6, 5.1 Hz, 1H), 3.13 – 3.00 (m, 2H), 2.14 (s, 3H), 2.11 (s, 3H), 2.05 (s, 3H), 2.03 (s, 3H), 2.02 (s, 3H), 2.00 (s, 3H), 1.99 (s, 3H). **^{13}C NMR** (125 MHz, $CDCl_3$, 25 ºC, δ): 196.71, 170.72, 170.57, 170.40, 169.69, 169.47, 169.38, 136.63, 133.70, 128.93, 128.13, 101.33, 93.42, 76.04, 73.07, 72.03, 71.73, 70.70, 69.97, 68.07, 62.74, 61.89, 36.73, 35.05, 21.25, 20.97, 20.93, 20.78, 20.73. **HRMS** (ESI-TOF) *m/z* calcd for $C_{34}H_{46}NO_{18}$ [(M + NH_4)$^+$], 766.2709, found, 756.2695.

(2R,3R,4S,5R,6R)-2-(acetoxymethyl)-6-(((2R,3S,4R,5S,6R)-4,6-diacetoxy-2-(acetoxymethyl)-5-(2-oxo-2-phenylethyl)tetrahydro-2H-pyran-3-yl)oxy)tetrahydro-2H-pyran-3,4,5-triyl triacetate (4j)

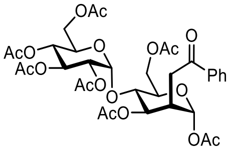

4j

Prepared according to the General Procedure C, the title compound was obtained as a white solid (90.0 mg, 0.122 mmol, 61% yield, axial: equatorial = 7.1:1). **R*f*** = 0.2 [Hexanes: EtOAc 2:1 (v/v)]. The analytic amount of axial diastereomer was separated by the analytical HPLC column.

Data for axial product (**4j-*ax***): t_R = 7.3 min, 20% (v/v) isopropanol in hexane at the flow rate of 1.0 ml/min. **^{1}H NMR** (500 MHz, $CDCl_3$, 25 ºC, δ): 7.93 (d, *J* = 7.5 Hz, 2H), 7.60 (t, *J* = 7.4 Hz, 1H), 7.49 (t, *J* = 7.7 Hz, 2H), 6.04 (d, *J* = 2.9 Hz, 1H, *H1*), 5.51 (d, *J* = 4.0 Hz, 1H), 5.44 – 5.35 (m, 2H), 5.08 (t, *J* = 9.9 Hz, 1H), 4.91 (dd, *J* = 10.5, 4.0 Hz, 1H), 4.34 (dd, *J* = 12.2, 2.1 Hz, 1H), 4.29 – 4.19 (m, 2H), 4.14 – 4.04 (m, 2H), 4.03 – 3.95 (m, 2H), 3.21 (dd, *J* = 17.5, 6.1 Hz, 1H), 3.14 (dt, *J* = 9.2, 6.2 Hz, 1H), 3.03 (dd, *J* = 17.5, 6.4 Hz, 1H), 2.20 (s, 3H), 2.14 (s, 3H), 2.10 (s, 3H), 2.09 (s, 3H), 2.03 (s, 3H), 2.01 (s, 3H), 1.95 (s, 3H). **^{13}C NMR** (125 MHz, $CDCl_3$, 25 ºC, δ): 196.49, 170.70, 170.65, 170.61, 170.21, 169.79, 169.58, 169.35, 136.45, 133.78, 128.96, 128.08, 95.80, 93.31, 72.66, 71.16, 70.35, 70.04, 69.66, 68.65, 68.14, 63.48, 61.51, 36.40, 34.88, 21.28, 21.15, 20.93, 20.84, 20.78, 20.74, 20.69. **HRMS** (ESI-TOF) *m/z* calcd for $C_{34}H_{46}NO_{18}$ [(M + NH_4)$^+$], 766.2709, found, 756.2703.

(2R,3S,4S,5R,6S)-2-(acetoxymethyl)-6-(((2R,3S,4R,5S,6R)-3,4,6-triacetoxy-5-(2-oxo-2-phenylethyl)tetrahydro-2H-pyran-2-yl)methoxy)tetrahydro-2H-pyran-3,4,5-triyl triacetate (4k)

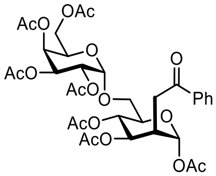

4k

Prepared according to the General Procedure C, the title compound was obtained as a white solid (66.5 mg, 0.090 mmol, 45% yield, axial: equatorial = 3.2:1). $\mathbf{R_f}$ = 0.30 [Hexanes: EtOAc 2:1 (v/v)].

Data for axial product (**4k-*ax***): $[\alpha]_D^{25}$ = -76.7 (*c* = 7.95, MeCN). **^{1}H NMR** (700 MHz, $CDCl_3$, 25 ºC, δ): 8.02 (d, *J* = 7.5 Hz, 2H), 7.58 (t, *J* = 7.4 Hz, 1H), 7.47 (t, *J* = 7.7 Hz, 2H), 6.07 (d, *J* = 1.0 Hz, 1H, *H1*), 5.57 (dd, *J* = 9.8, 5.3 Hz, 1H), 5.48 (q, *J* = 3.5 Hz, 2H), 5.34 (t, *J* = 9.9 Hz, 1H), 5.14 – 5.06 (m, 2H), 4.27 (t, *J* = 6.5 Hz, 1H), 4.16 – 4.09 (m, 1H), 4.06 – 3.98 (m, 1H), 3.98 – 3.93 (m, 1H), 3.74 (dd, *J* = 10.9, 4.0 Hz, 1H), 3.52 (dd, *J* = 10.9, 2.4 Hz, 1H), 3.43 (dd, *J* = 18.0, 3.5 Hz, 1H), 3.18 (dd, *J* = 18.0, 9.3 Hz, 1H), 3.11 (dd, *J* = 5.3, 3.9 Hz, 1H), 2.19 (s, 3H), 2.14 (s, 3H), 2.06 (s, 3H), 2.03 (s, 3H), 2.01 (s, 3H), 1.99 (s, 3H), 1.80 (s, 3H). **^{13}C NMR** (175 MHz, $CDCl_3$, 25 ºC, δ): 197.19, 170.61, 170.48, 170.36, 169.98, 169.94, 169.92, 169.03, 136.51, 133.66, 128.84, 128.37, 96.07, 93.21, 70.71, 69.50, 68.40, 67.46, 66.49, 66.45, 66.40, 61.71, 37.53, 34.09, 21.15, 20.99, 20.89, 20.87, 20.84, 20.82, 20.62. **HRMS** (ESI-TOF) *m/z* calcd for $C_{34}H_{46}NO_{18}$ $[(M + NH_4)^+$], 766.2709, found, 756.2704.

(2R,3S,4R,5S,6R)-6-((benzoyloxy)methyl)-3-(2-oxo-2-phenylethyl)tetrahydro-2H-pyran-2,4,5-triyl tribenzoate (4l)

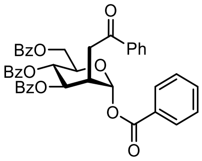

4l

Prepared according to the General Procedure C, the title compound was obtained as a white solid (103.0 mg, 0.147 mmol, 74% yield, axial: equatorial = 5.6:1). $\mathbf{R_f}$ = 0.45 [Hexanes: EtOAc 3:1 (v/v)]. The analytic amount of axial and equatorial diastereomers were separated by the analytical HPLC column.

Data for axial product (**4l-*ax***): t_R = 7.3 min, 20% (v/v) isopropanol in hexane at the flow rate of 1.0 ml/min. **^{1}H NMR** (500 MHz, $CDCl_3$, 25 ºC, δ): 8.23 – 8.19 (m, 2H), 8.06 – 8.02 (m, 2H), 7.99 – 7.94 (m, 2H), 7.92 (d, *J* = 7.3 Hz, 2H), 7.88 (d, *J* = 7.3 Hz, 2H), 7.66 (t, *J* = 7.4 Hz, 1H), 7.61 – 7.35 (m, 12H), 7.32 (t, *J* = 7.8 Hz, 2H), 6.46 (s, 1H, *H1*), 6.14 (dd, *J* = 9.9, 5.2 Hz, 1H, *H3*), 5.95 (t, *J* = 9.8 Hz, 1H, *H4*), 4.61 (d, *J* = 9.7 Hz, 1H, *H6*), 4.53 – 4.40 (m, 2H, *H5*, *H7*), 3.66 – 3.60 (m, 1H, *H2*), 3.57 (dd, *J* = 17.8, 4.0 Hz, 1H), 3.44

(dd, J = 17.8, 9.2 Hz, 1H). ^{13}**C NMR** (125 MHz, $CDCl_3$, 25 ºC, δ): 196.68, 166.10, 165.76, 165.59, 164.48, 136.58, 133.91, 133.69, 133.67, 133.49, 133.25, 130.24, 129.98, 129.92, 129.83, 129.40, 129.20, 128.97, 128.87, 128.85, 128.61, 128.57, 128.24, 94.12, 70.96, 70.63, 66.86, 62.92, 38.20, 34.38. **HRMS** (ESI-TOF) *m/z* calcd for $C_{42}H_{38}NO_{10}$ [(M + NH_4)$^+$], 716.2490, found, 716.2479.

Data for equatorial product(**4l-*eq***): t_R = 9.4 min, 20% (v/v) isopropanol in hexane at the flow rate of 1.0 ml/min. ^{1}**H NMR** (500 MHz, $CDCl_3$, 25 ºC, δ): 8.15 (d, J = 7.2 Hz, 2H), 8.00 (d, J = 7.2 Hz, 2H), 7.89 (d, J = 7.3 Hz, 2H), 7.86 (d, J = 7.3 Hz, 2H), 7.78 (d, J = 7.4 Hz, 2H), 7.66 (t, J = 7.4 Hz, 1H), 7.54 (dd, J = 15.5, 7.7 Hz, 3H), 7.46 (dt, J = 19.4, 7.4 Hz, 3H), 7.39 (t, J = 7.8 Hz, 3H), 7.37 – 7.29 (m, 5H), 6.74 (d, J = 3.2 Hz, 1H, *H1*), 6.03 – 5.90 (m, 1H, *H3*), 5.78 (t, J = 9.8 Hz, 1H, *H4*), 4.56 (dd, J = 11.9, 2.7 Hz, 1H, *H6*), 4.53 – 4.49 (m, 1H, *H5*), 4.45 (dd, J = 11.9, 4.2 Hz, 1H, *H7*), 3.44 – 3.32 (m, 1H, *H2*), 3.26 – 3.08 (m, 2H). ^{13}**C NMR** (125 MHz, $CDCl_3$, 25 ºC, δ): 196.98, 166.62, 166.29, 165.46, 164.54, 134.02, 133.48, 133.17, 130.11, 129.92, 128.96, 128.68, 128.50, 128.48, 128.21, 93.17, 72.15, 70.77, 70.29, 62.98, 40.53, 35.82. **HRMS** (ESI-TOF) *m/z* calcd for $C_{42}H_{38}NO_{10}$ [(M + NH_4)$^+$], 716.2490, found, 716.2483.

(2R,3S,4R,5S,6R)-2-((benzoyloxy)methyl)-5-(2-oxo-2-phenylethyl)-6-((thiophene-2-carbonyl)oxy)tetrahydro-2H-pyran-3,4-diyl dibenzoate (4m)

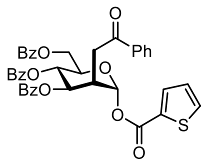

4m

Prepared according to the General Procedure C, the title compound was obtained as a white solid (94.0 mg, 0.133 mmol, 67% yield, axial: equatorial = 5.6:1). $\mathbf{R_f}$ = 0.45 [Hexanes: EtOAc 3:1 (v/v)]. The analytic amount of axial diastereomer was separated by the analytical HPLC column.

Data for axial product (**4m-*ax***): t_R = 8.9 min, 20% (v/v) isopropanol in hexane at the flow rate of 1.0 ml/min. ^{1}**H NMR** (500 MHz, $CDCl_3$, 25 ºC, δ): 8.05 (d, J = 7.2 Hz, 2H), 8.01 (dd, J = 3.7, 1.1 Hz, 1H), 7.97 (d, J = 7.2 Hz, 2H), 7.91 (d, J = 7.3 Hz, 2H), 7.87 (d, J = 7.2 Hz, 2H), 7.69 (dd, J = 5.0, 1.1 Hz, 1H), 7.57 (td, J = 7.4, 3.7 Hz, 2H), 7.52 (t, J = 7.4 Hz, 1H), 7.48 (t, J = 7.4 Hz, 1H), 7.45 – 7.35 (m, 6H), 7.32 (t, J = 7.8 Hz, 2H), 7.20 (dd, J = 4.9, 3.9 Hz, 1H), 6.41 (s, 1H, *H1*), 6.11 (dd, J = 9.9, 5.0 Hz, 1H, *H3*), 5.93 (t, J = 9.8 Hz, 1H, *H4*), 4.61 (dd, J = 11.6, 2.1 Hz, 1H, *H6*), 4.48 (ddd, J = 16.0, 9.1, 4.1 Hz, 2H, *H5*, *H7*), 3.64 – 3.51 (m, 2H, *H2*), 3.41 (dd, J = 17.6, 8.9 Hz, 1H). ^{13}**C NMR** (175 MHz, $CDCl_3$, 25 ºC, δ): 196.62, 166.10, 165.75, 165.51, 159.93, 136.57, 134.78, 133.79, 133.69, 133.47, 133.25, 132.79, 129.99, 129.92, 129.83, 129.21, 128.98, 128.85, 128.61, 128.58, 128.32, 128.23, 94.20, 71.02, 70.51, 66.81, 62.90, 38.16, 34.32. **HRMS** (ESI-TOF) *m/z* calcd for $C_{40}H_{36}NO_{10}S$ [(M + NH_4)$^+$], 722.2054, found, 722.2052.

(2R,3S,4R,5S,6S)-6-((((1R,2S,5R)-2-isopropyl-5-methylcyclohexyl)oxy)carbonyl)-3-(2-oxo-2-phenylethyl)tetrahydro-2H-pyran-2,4,5-triyl triacetate (4n)

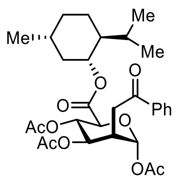

4n

Prepared according to the General Procedure C, the title compound was obtained as a white solid (70.0 mg, 0.125 mmol, 63% yield, axial: equatorial = 3.8:1). **R*f*** = 0.25 [Hexanes: EtOAc 2:1 (v/v)].

Data for axial product (**4n-*ax***): mp = 85-86°C. $[\alpha]_D^{25}$ = +61.2 (*c* = 5.85, MeCN). **^{1}H NMR** (500 MHz, $CDCl_3$, 25 ºC, δ): 7.98 (d, *J* = 7.6 Hz, 2H), 7.59 (t, *J* = 7.3 Hz, 1H), 7.49 (t, *J* = 7.6 Hz, 2H), 6.24 (s, 1H, *H1*), 5.52 (dd, *J* = 8.5, 4.2 Hz, 1H), 5.35 (t, *J* = 8.6 Hz, 1H), 4.75 (td, *J* = 10.8, 4.2 Hz, 1H), 4.40 (d, *J* = 8.6 Hz, 1H), 3.32 (dd, *J* = 20.9, 8.0 Hz, 1H), 3.21 – 3.10 (m, 2H), 2.16 (s, 3H), 2.07 (s, 3H), 1.99 (d, *J* = 12.4 Hz, 1H), 1.96 (s, 3H), 1.88 (dd, *J* = 15.7, 10.2 Hz, 1H), 1.68 (d, *J* = 11.9 Hz, 2H), 1.44 – 1.38 (m, 1H), 1.06 (dd, *J* = 23.2, 12.5 Hz, 1H), 0.99 – 0.83 (m, 9H), 0.77 (d, *J* = 6.9 Hz, 3H). **^{13}C NMR** (125 MHz, $CDCl_3$, 25 ºC, δ): 196.86, 169.73, 169.53, 168.58, 167.36, 136.52, 133.69, 128.88, 128.26, 92.72, 76.46, 71.99, 69.25, 67.13, 46.98, 40.62, 37.01, 34.37, 34.19, 31.46, 26.35, 23.51, 22.09, 21.12, 20.89, 16.47. **HRMS** (ESI-TOF) *m/z* calcd for $C_{30}H_{44}NO_{10}$ [$(M + NH_4)^+$], 578.2960, found, 578.2950.

(2R,3S,4R,5S,6R)-6-(((2-(3-cyano-4-isobutoxyphenyl)-4-methylthiazole-5-carbonyl)oxy)methyl)-3-(2-oxo-2-phenylethyl)tetrahydro-2H-pyran-2,4,5-triyl triacetate (4o)

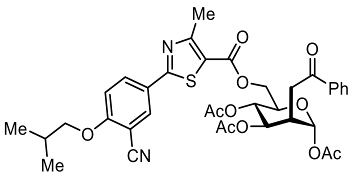

4o

Prepared according to the General Procedure C, the reaction run at 0.1 mmol scale. The title compound was obtained as a white solid (32 mg, 0.045 mmol, 45% yield, axial: equatorial = 5:1). **R*f*** = 0.4 [Hexanes: EtOAc 2:1 (v/v)]. The analytic amount of axial diastereomer was separated by the analytical HPLC column.

Data for axial product (**4o-*ax***): t_R = 42.0 min, 10% (v/v) isopropanol in hexane at the flow rate of 1.0 ml/min. **^{1}H NMR** (700 MHz, $CDCl_3$, 25 ºC, δ): 8.16 (s,1H), 8.07 (d, *J* = 8.6 Hz, 1H), 7.98 (d, *J* = 7.4 Hz, 2H), 7.57 (t, *J* = 7.4 Hz 1H), 7.42 (t, *J* = 7.4 Hz, 2H), 6.99 (d, *J* = 8.7 Hz, 1H), 6.12 (s, 1H, *H1*), 5.61 (d, *J* = 9.9 Hz, 1H), 5.34 (t, *J* = 10.2 Hz, 1H), 4.43–4.39 (m, 2H), 4.14 (d, *J* = 10.2 Hz, 1H), 3.91 (d, *J* = 5.7 Hz, 2H), 3.43 (dd, *J* = 21.2, 8.0 Hz, 1H), 3.21–3.17 (m, 2H), 2.78 (s, 3H), 2.25–2.21 (m, 1H), 2.19 (s, 3H), 2.08 (s, 3H),

1.97 (s, 3H), 1.10 (d, *J* = 6.2 Hz, 6H). **^{13}C NMR** (175 MHz, $CDCl_3$, 25 ºC, δ): 196.75, 169.83, 169.74, 168.68, 167.61, 162.60, 161.88, 161.38, 136.41, 133.65, 132.70, 132.09, 128.75, 127.99, 125.88, 121.07, 115.31, 112.60, 103.02, 93.15, 75.71, 70.09, 69.23, 66.07, 62.60, 37.56, 34.23, 28.15, 21.01, 20.80, 20.72, 19.05, 17.59. **HRMS** (ESI-TOF) *m/z* calcd for $C_{36}H_{38}N_2O_{11}S$ [$(M + H)^+$], 707.2275, found, 707.2268.

(2R,3S,4R,5S,6R)-6-((((4aS,6aS,6bR,8aR,10S,12aR,12bR,14bS)-10-acetoxy-2,2,6a,6b,9,9,12a-heptamethyl-1,2,3,4,4a,5,6,6a,6b,7,8,8a,9,10,11,12,12a,12b,13,14b-icosahydropicene-4a-carbonyl)oxy)methyl)-3-(2-oxo-2-phenylethyl)tetrahydro-2H-pyran-2,4,5-triyl triacetate (4p)

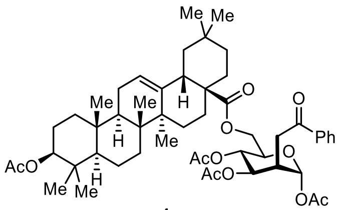

4p

Prepared according to the General Procedure C, the reaction run at 0.1 mmol scale. The title compound was obtained as a white solid (47 mg, 0.053 mmol, 53% yield, axial: equatorial = 6.3:1). **R_f** = 0.6 [Hexanes: EtOAc 2:1 (v/v)]. The analytic amount of axial diastereomer was separated by the analytical HPLC column. Data for axial product (**4p-*ax***): t_R = 14.9 min, 5% (v/v) isopropanol in hexane at the flow rate of 1.0 ml/min. **^{1}H NMR** (700 MHz, $CDCl_3$, 25 ºC, δ): 7.98 (d, *J* = 7.4 Hz, 2H), 7.61 (t, *J* = 7.4 Hz, 1H), 7.50 (t, *J* = 7.7 Hz, 2H), 6.09 (s, 1H, *H1*), 5.56 (dd, *J* = 9.9, 4.9 Hz, 1H), 5.30–5.26 (m, 2H), 4.48–4.45 (m, 1H), 4.26 (d, *J* = 10.8 Hz, 1H), 4.03–3.97 (m, 2H), 3.45–3.42 (m, 1H), 3.15–3.07 (m, 2H), 2.85 (dd, *J* = 13.6, 3.9 Hz, 1H), 2.16 (s, 3H), 2.05 (s, 3H), 2.04 (s, 3H), 1.95 (s, 3H), 1.92–1.11 (m, 21H), 1.04 (s, 3H), 0.92 (s, 3H), 0.91 (s, 3H), 0.89 (s, 3H), 0.84 (s, 3H), 0.83 (s, 3H), 0.80–0.75 (m, 1H), 0.66 (s, 3H). **^{13}C NMR** (175 MHz, $CDCl_3$, 25 ºC, δ): 196.79, 176.94, 169.78 168.53, 136.46, 133.59, 128.69, 128.04, 122.64, 93.07, 80.90, 70.23, 69.45, 66.06, 61.46, 55.25, 47.48, 46.82, 45.76, 41.55, 41.23, 39.19, 38.08, 37.65, 36.86, 34.65, 34.20, 33.86, 33.07, 32.60, 32.30, 31.57, 30.68, 27.97, 27.58, 25.76, 25.26, 23.57, 23.49, 23.35, 23.16, 21.30, 20.99, 20.79, 20.68, 18.12, 16.75, 16.63, 15.30, 14.10. **HRMS** (ESI-TOF) *m/z* calcd for $C_{52}H_{72}O_{12}$ [$(M + NH_4)^+$], 903.5368, found, 903.5363.

(2R,3S,4R,5S,6R)-6-(((2-(4-(2-(4-chlorobenzamido)ethyl)phenoxy)-2-methylpropanoyl)oxy)methyl)-3-(2-oxo-2-phenylethyl)tetrahydro-2H-pyran-2,4,5-triyl triacetate (4q)

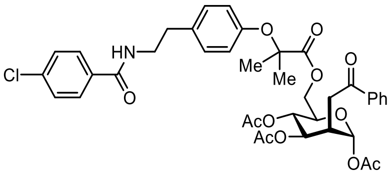

4q

Prepared according to the General Procedure C, the title compound was obtained as a white solid (88.0 mg, 0.117 mmol, 58% yield, axial: equatorial = 4.6:1). **R*f*** = 0.20 [Hexanes: EtOAc 2:1 (v/v)]. The analytic amount of axial diastereomer was separated by the analytical HPLC column.

Data for axial product (**4q-*ax***): t_R = 13.5 min, 10% (v/v) isopropanol in hexane at the flow rate of 1.0 ml/min. **^{1}H NMR** (500 MHz, $CDCl_3$, 25 ºC, δ): 7.92 (d, *J* = 7.5 Hz, 2H), 7.62 (d, *J* = 8.5 Hz, 2H), 7.60 – 7.55 (m, 1H), 7.45 (t, *J* = 7.7 Hz, 2H), 7.36 (d, *J* = 8.4 Hz, 2H), 6.99 (d, *J* = 8.4 Hz, 2H), 6.77 (d, *J* = 8.4 Hz, 2H), 6.23 (s, 1H), 6.01 (s, 1H, *H1*), 5.55 (dd, *J* = 9.8, 5.2 Hz, 1H), 5.29 (t, *J* = 9.9 Hz, 1H), 4.33 – 4.21 (m, 2H), 4.05 (dd, *J* = 10.0, 2.9 Hz, 1H), 3.73 – 3.52 (m, 2H), 3.34 (dd, *J* = 17.6, 3.7 Hz, 1H), 3.12 – 3.04 (m, 1H), 3.00 (dd, *J* = 17.6, 8.8 Hz, 1H), 2.87 (t, *J* = 6.2 Hz, 1H), 2.79 (t, *J* = 6.7 Hz, 1H), 2.13 (s, 3H), 2.05 (s, 3H), 1.94 (s, 3H), 1.63 (s, 3H), 1.57 (s, 3H). **^{13}C NMR** (125 MHz, $CDCl_3$, 25 ºC, δ): 197.08, 174.01, 169.93, 169.87, 168.87, 166.55, 154.05, 137.67, 136.42, 133.74, 133.21, 133.02, 129.65, 128.93, 128.88, 128.86, 128.48, 128.25, 120.10, 93.16, 79.36, 70.15, 69.37, 66.23, 62.91, 41.38, 37.64, 34.86, 34.05, 26.18, 24.78, 21.10, 20.92, 20.85, 20.78. **HRMS** (ESI-TOF) *m/z* calcd for $C_{39}H_{43}ClNO_{12}$ [(M + H)$^+$], 752.2468, found, 752.2456.

(2R,3S,4R,5S,6R)-6-(((2-(4-isobutylphenyl)propanoyl)oxy)methyl)-3-(2-oxo-2-phenylethyl)tetrahydro-2H-pyran-2,4,5-triyl triacetate (4r)

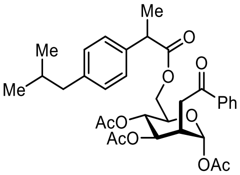

4r

Prepared according to the General Procedure C, the title compound was obtained as a white solid (76.0 mg, 0.127 mmol, 64% yield, axial: equatorial = 6.2:1). **R*f*** = 0.35 [Hexanes: EtOAc 2:1 (v/v)]. The analytic amount of axial diastereomer was separated by the analytical HPLC column.

Data for axial product (**4r-*ax***): t_R = 7.4 min, 10% (v/v) isopropanol in hexane at the flow rate of 1.0 ml/min. **^{1}H NMR** (500 MHz, $CDCl_3$, 25 ºC, δ): 7.97 (t, *J* = 8.2 Hz, 2H), 7.61 (dd, *J* = 12.1, 6.8 Hz, 1H), 7.50 (dd, *J* = 13.7, 7.2 Hz, 2H), 7.19 (t, *J* = 8.3 Hz, 2H), 7.05 (dd, *J* = 18.4, 7.8 Hz, 3H), 6.06 (s, 0.5H, *H1*), 6.04 (s, 0.5H, *H1*), 5.54 (dd, *J* = 9.6, 4.8 Hz, 1H), 5.22 (t, *J* = 10.0 Hz, 0.5H), 5.16 (t, *J* = 9.9 Hz, 0.5H), 4.25 – 4.10 (m, 2H), 4.08 – 3.96 (m, 1H), 3.74 (q, *J* = 6.9 Hz, 1H), 3.40 – 3.29 (m, 1H), 3.15 – 3.02 (m, 2H), 2.39 (dd, *J*

= 12.6, 7.2 Hz, 2H), 2.15 (s, 1.5H), 2.13 (s, 1.5H), 2.04 (s, 1.5H), 2.02 (s, 1.5H), 1.95 (s, 1.5H), 1.94 (s, 1.5H),, 1.86 – 1.76 (m, 1H), 1.54 – 1.48 (m, 3H), 0.86 (dd, *J* = 6.2, 3.0 Hz, 6H). ^{13}**C NMR** (125 MHz, $CDCl_3$, 25 ºC, δ): 197.10, 197.06, 174.41, 174.27, 169.96, 169.87, 169.84, 168.78, 168.75, 140.73, 137.46, 137.34, 136.59, 133.70, 129.47, 128.88, 128.23, 127.34, 127.31, 93.26, 93.23, 70.30, 70.26, 69.48, 66.49, 66.32, 62.72, 62.54, 45.34, 45.13, 45.10, 37.74, 34.29, 34.26, 30.27, 30.25, 22.50, 21.10, 21.07, 20.91, 20.89, 20.82, 20.80, 18.72, 18.65. **HRMS** (ESI-TOF) *m/z* calcd for $C_{33}H_{41}O_{10}$ [$(M + H)^+$], 597.2694, found, 597.2685.

(2R,3S,4R,5S,6R)-6-(((4-(N,N-dipropylsulfamoyl)benzoyl)oxy)methyl)-3-(2-oxo-2-phenylethyl)tetrahydro-2H-pyran-2,4,5-triyl triacetate (4s)

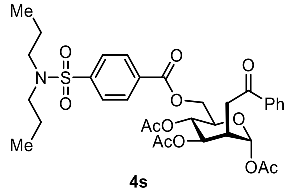

4s

Prepared according to the General Procedure C, the title compound was obtained as a white solid (71.5 mg, 0.105 mmol, 53% yield, axial: equatorial = 4.4:1). $\mathbf{R}_f$ = 0.15 [Hexanes: EtOAc 3:1 (v/v)]. The analytic amount of axial diastereomer was separated by the analytical HPLC column.

Data for axial product (**4s-*ax***): t_R = 14.9 min, 10% (v/v) isopropanol in hexane at the flow rate of 1.0 ml/min. ^{1}**H NMR** (700 MHz, $CDCl_3$, 25 ºC, δ): 8.15-8.19 (m, 2H), 7.93-7.96 (m, 2H), 7.87-7.90 (m, 2H), 7.60-7.63 (m, 1H), 7.48-7.51 (m, 2H), 6.11 (d, *J* = 1.4 Hz, 1H, *H1*), 5.53-5.68 (m, 1H), 5.36 (t, *J* = 9.8 Hz, 1H), 4.49 (dd, *J* = 2.1, 11.9 Hz, 1H), 4.41 (dd, *J* = 4.2, 11.9 Hz, 1H), 4.17 (ddd, *J* = 2.1, 4.2, 9.8 Hz, 1H), 3.38-3.44 (m, 1H), 3.15-3.21 (m, 2H), 3.10 (ddd, *J* = 0.7, 7.0, 9.1 Hz, 4H), 2.19 (s, 3H), 2.07 (s, 3H), 1.98 (s, 3H), 1.51-1.59 (m, 4H), 0.87 (t, *J* = 7.7 Hz, 6H). ^{13}**C NMR** (175 MHz, $CDCl_3$, 25 ºC, δ): 196.73, 170.04, 169.84, 168.85, 164.81, 144.71, 136.45, 133.89, 133.16, 130.38, 130.38, 128.97, 128.97, 128.07, 128.07, 127.27, 127.27, 93.26, 70.21, 69.28, 66.31, 63.19, 50.12, 50.12, 37.56, 34.31, 22.12, 22.12, 21.13, 20.92, 20.84, 11.28, 11.28. **HRMS** (ESI-TOF) *m/z* calcd for $C_{33}H_{45}N_2O_{12}S$ [$(M + NH_4)^+$], 693.2688, found, 693.2682.

(2R,3S,4R,5S,6R)-6-(((2-(10-oxo-10,11-dihydrodibenzo[b,f]thiepin-2-yl)propanoyl)oxy)methyl)-3-(2-oxo-2-phenylethyl)tetrahydro-2H-pyran-2,4,5-triyl triacetate (4t)

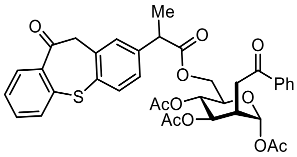

4t

Prepared according to the General Procedure C, the reaction run at 0.1 mmol scale. The title compound was obtained as a white solid (28 mg, 0.040 mmol, 40% yield, axial: equatorial = 4.9:1). **R*f*** = 0.5 [Hexanes: EtOAc 2:1 (v/v)]. The analytic amount of axial diastereomer was separated by the analytical HPLC column. Data for axial product (**4t-*ax***): t_R = 21.5 min, 10% (v/v) isopropanol in hexane at the flow rate of 1.0 ml/min. **^{1}H NMR** (700 MHz, $CDCl_3$, 25 ºC, δ): 8.15 (dd, *J* = 7.9, 1.3 Hz, 1H), 7.97–7.94 (m, 2H), 7.62–7.56 (m, 2H), 7.56 (dd, *J* = 17.3, 8.0 Hz, 1H), 7.49 (dd, *J* = 17.1, 8.0 Hz, 2H), 7.42–7.39 (m, 2H), 7.30–7.28 (m, 1H), 7.16–7.13 (m, 1H), 6.07 (d, *J* = 1.7 Hz, 1H, *H1*), 5.54 (ddd, *J* = 9.7, 5.3, 1.7 Hz, 1H), 5.20 (t, *J* = 10.0 Hz, 0.5H), 5.13 (t, *J* = 10.0 Hz, 0.5H), 4.35 (s, 1H), 4.33 (d, *J* = 4.4 Hz, 1H), 4.24 (dd, *J* = 12.3, 5.1 Hz, 0.6H), 4.19–4.15 (m, 1H), 4.09 (dd, *J* = 12.3, 2.1 Hz, 0.5H), 4.05–4.00 (m, 1H), 3.77 (ddd, *J* = 14.3, 11.5, 7.2 Hz, 1H), 3.36 (dd, *J* = 17.6, 4.1 Hz, 0.7H), 3.31 (dd, *J* = 17.2, 3.6 Hz, 0.5H), 3.14–3.11 (m, 1H), 3.07 (ddd, *J* = 17.6, 8.8, 5.9 Hz, 1H), 2.14 (s, 1.4H), 2.12 (s, 1.4H), 2.04 (s, 1.6H), 2.02 (s, 1.4H), 1.96 (s, 1.4H), 1.95 (s, 1.5H), 1.51 (dd, *J* = 7.2, 2.6 Hz, 3H). **^{13}C NMR** (175 MHz, $CDCl_3$, 25 ºC, δ): 196.86, 191.31, 191.28, 173.41, 173.37, 169.87, 169.73, 169.70, 142.26, 138.00, 136.41, 133.59, 133.58, 133.36, 133.33, 132.48, 132.47, 131.50, 131.49, 131.46, 130.83, 128.76, 128.59, 128.06, 126.83, 126.82, 126.55, 126.39, 93.12, 93.06, 70.01, 69.26, 66.27, 66.12, 62.83, 62.65, 50.95, 45.11, 45.08, 37.59, 37.54, 34.17, 34.11, 20.95, 20.92, 20.78, 20.76, 20.68, 20.64, 18.57, 18.28. **HRMS** (ESI-TOF) *m/z* calcd for $C_{37}H_{36}O_{11}S$ [$(M + NH_4)^+$], 706.2322, found, 706.2317.

(2R,3S,4R,5S,6R)-6-((6-(3-((3r,5r,7r)-adamantan-1-yl)-4-methoxyphenyl)-2-naphthoyl)oxy)-2-((benzoyloxy)methyl)-5-(2-oxo-2-phenylethyl)tetrahydro-2H-pyran-3,4-diyl dibenzoate (4u)

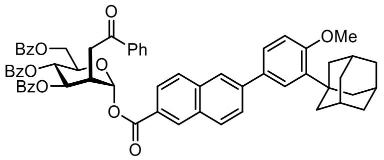

4u

Prepared according to the General Procedure C, the title compound was obtained as a white solid (89.0 mg, 0.090 mmol, 45% yield, axial: equatorial = 4.2:1). **R*f*** = 0.40 [Hexanes: EtOAc 2:1 (v/v)].

Data for axial product (**4u-*ax***): mp = 253-256°C. $[\alpha]_D^{25}$ = +23.8 (*c* = 2.87, MeCN). **^{1}H NMR** (500 MHz, $CDCl_3$, 25 ºC, δ): 8.77 (s, 1H), 8.21 (d, *J* = 8.6 Hz, 1H), 8.11 (d, *J* = 8.5 Hz, 1H), 8.08 – 7.97 (m, 6H), 7.94 (d, *J* = 7.5 Hz, 2H), 7.90 (d, *J* = 7.5 Hz, 2H), 7.86 (d, *J* = 8.6 Hz, 1H), 7.64 (s, 1H), 7.61 – 7.30 (m, 13H), 7.03 (d, *J* = 8.4 Hz, 1H), 6.53 (s, 1H, *H1*), 6.22 (dd, *J* = 9.7, 5.2 Hz, 1H), 5.98 (t, *J* = 9.8 Hz, 1H), 4.63 (dd, *J* = 12.0, 2.2 Hz, 1H), 4.59 – 4.53 (m, 1H), 4.49 (dd, *J* = 12.1, 4.1 Hz, 1H), 3.92 (s, 3H), 3.75 – 3.64 (m, 1H), 3.60 (dd, *J* = 17.9, 4.0 Hz, 1H), 3.47 (dd, *J* = 17.9, 9.1 Hz, 1H), 2.21 (s, 6H), 2.12 (s, 3H), 1.82 (s, 6H). **^{13}C**

NMR (125 MHz, $CDCl_3$, 25 ºC, δ): 196.73, 166.11, 165.79, 165.61, 164.80, 159.17, 142.00, 139.21, 136.61, 136.51, 133.68, 133.49, 133.23, 132.60, 131.82, 131.38, 130.14, 130.01, 129.92, 129.83, 129.25, 129.01, 128.86, 128.77, 128.62, 128.59, 128.56, 128.26, 126.85, 126.16, 126.07, 125.97, 125.78, 124.94, 112.29, 94.26, 70.96, 70.74, 67.00, 62.98, 55.34, 40.76, 38.22, 37.39, 37.28, 34.49, 29.26. **HRMS** (ESI-TOF) *m/z* calcd for $C_{63}H_{57}O_{11}$ [$(M + H)^+$], 989.3895, found, 989.3878.

Data for equatorial product(**4u-*eq***): **^{1}H NMR** (700 MHz, $CDCl_3$, 25 ºC, δ): 8.70 (s, 1H), 8.12 (dd, *J* = 17.6, 8.5 Hz, 2H), 8.07 (s, 1H), 8.00 (d, *J* = 7.3 Hz, 3H), 7.91 (d, *J* = 7.5 Hz, 1H), 7.90 – 7.85 (m, 3H), 7.80 (d, *J* = 7.6 Hz, 2H), 7.64 (d, *J* = 1.8 Hz, 1H), 7.59 (dd, *J* = 8.3, 1.9 Hz, 1H), 7.52 (dd, *J* = 13.1, 5.7 Hz, 1H), 7.48 (t, *J* = 6.0 Hz, 2H), 7.44 (t, *J* = 7.3 Hz, 1H), 7.38 (t, *J* = 7.7 Hz, 2H), 7.35 – 7.28 (m, 7H), 7.02 (d, *J* = 8.4 Hz, 1H), 6.81 (d, *J* = 3.1 Hz, 1H, *H1*), 6.11 – 5.97 (m, 1H), 5.81 (t, *J* = 9.8 Hz, 1H), 4.64 – 4.54 (m, 2H), 4.48 (dd, *J* = 12.5, 4.5 Hz, 1H), 3.92 (s, 3H), 3.44 (dt, *J* = 10.5, 6.5 Hz, 1H), 3.22 (d, *J* = 6.4 Hz, 2H), 2.21 (s, 6H), 2.12 (s, 3H), 1.82 (s, 6H). **^{13}C NMR** (175 MHz, $CDCl_3$, 25 ºC, δ): 197.07, 166.65, 166.31, 165.50, 164.87, 159.21, 142.10, 139.24, 136.52, 136.35, 133.58, 133.51, 133.16, 132.47, 131.65, 131.34, 130.09, 129.96, 129.91, 129.78, 129.07, 128.87, 128.68, 128.52, 128.47, 128.22, 126.94, 126.13, 125.97, 125.86, 125.60, 124.91, 112.28, 93.23, 72.26, 70.77, 70.37, 63.00, 55.34, 40.75, 40.57, 37.38, 37.27, 35.90, 29.25. **HRMS** (ESI-TOF) *m/z* calcd for $C_{63}H_{56}NaO_{11}$ [$(M + Na)^+$], 1011.3715, found, 1011.3694.

Post-functionalizations

Reduction of C2-Ketonylsugar:

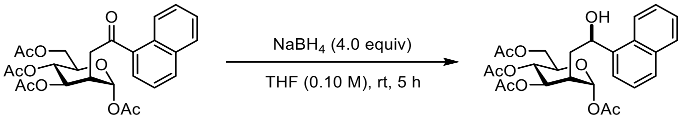

3i **5a**, 73%, dr 1:1

The reduction was done following the literature procedure.[19] An oven-dried round bottom flask containing a magnetic stir bar was charged with (2R,3S,4R,5S,6R)-6-(acetoxymethyl)-3-(2-(naphthalen-1-yl)-2-oxoethyl)tetrahydro-2H-pyran-2,4,5-triyl triacetate (**3i**) (50.0 mg, 0.10 mmol, 1.00 equiv) and THF (1.00 mL, 0.100 M). The solution was cooled to 0 °C and sodium borohydride (0.40 mmol, 4.00 equiv) was added. The reaction was allowed to warm up to room temperature and the progress was monitored by UPLC-MS. Upon completion, the reaction was quenched by adding water and extracted with ethyl acetate. The organic layer was washed with brine (1 x 50 mL), dried over $MgSO_4$ and concentrated under reduced pressure. The crude product was purified by flash column chromatography on silica gel eluting with Hexanes: EtOAc [2:1 (v/v)] to afford the title compound (36.6 mg, 0.073 mmol, 73%) as a colorless liquid. **R_f** = 0.5 [Hexanes: EtOAc 2:1 (v/v)]. **^{1}H NMR** (700 MHz, $CDCl_3$, 25 ºC, δ): 8.09 (d, *J* = 8.2 Hz, 1H), 8.00 (d, *J* = 8.4 Hz, 1H), 7.89 (t, *J* = 8.8 Hz, 2H), 7.83 – 7.77 (m, 2H), 7.70 (d, *J* = 7.0 Hz, 1H), 7.66 (d, *J* = 6.9 Hz, 1H), 7.57–7.46

(m, 6H), 6.47 (s, 1H), 6.40 (s, 1H), 5.78–5.68 (m, 1H), 5.57–5.51 (m, 1H), 5.41 (dd, *J* = 9.7, 5.3 Hz, 1H), 5.32 (dd, *J* = 9.9, 5.5 Hz, 1H), 5.14 (t, *J* = 9.9 Hz, 1H), 5.08 (t, *J* = 10.0 Hz, 1H), 4.18 (dd, *J* = 12.3, 4.4 Hz, 1H), 4.15 (dd, *J* = 12.3, 4.3 Hz, 1H), 4.11 (dd, *J* = 12.2, 2.1 Hz, 1H), 4.07 (dd, *J* = 12.3, 2.1 Hz, 1H), 4.05–3.99 (m, 2H), 2.71 (s, 1H), 2.63 (s, 1H), 2.39 (d, *J* = 14.8 Hz, 1H), 2.26–2.21 (m, 1H), 2.19 (s, 3H), 2.15 (s, 3H), 2.11–2.01 (m, 7H), 2.00 (s, 3H), 1.99 (s, 3H), 1.97 (s, 3H), 1.96 (s, 3H), 1.63 (s, 3H). **^{13}C NMR** (175 MHz, $CDCl_3$, 25 ºC, δ): 170.72, 170.71, 169.93, 169.80, 169.61, 169.59, 169.25, 169.06, 139.62, 139.21, 133.89, 133.77, 129.94, 129.69, 129.13, 129.03, 128.41, 128.20, 126.35, 126.22, 125.70, 125.67, 125.48, 122.91, 122.82, 122.75, 122.60, 94.46, 93.70, 70.58, 70.42, 70.36, 69.90, 68.33, 66.02, 65.88, 62.19, 62.17, 40.75, 38.07, 33.72, 32.35, 25.35, 21.16, 21.10, 20.78, 20.69, 20.66, 20.64, 20.34. **HRMS** (ESI-TOF) *m/z* calcd for $C_{26}H_{30}O_{10}$ [$(M + NH_4)^+$], 520.2183, found, 520.2176.

Hydrogenation of C2-Ketonylsugar:

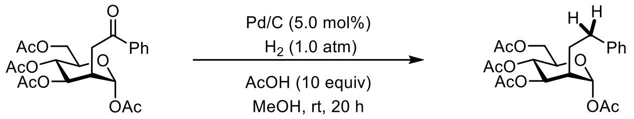

3a **6a**, 97%

To a solution of compound **3a** (45.0 mg, 0.100 mmol) in MeOH (4.00 mL, 0.025 M) were added AcOH (57.1 uL, 1.00 mmol, 10.0 equiv) and 10 wt. % Pd/C (5.3 mg, 5.0 mol%) under nitrogen gas atmosphere. Then, exchange the reaction flask with H_2 (1 atm) for 6 times. After stirring at room temperature for 12 h, the reaction mixture was filtered through celite, and the filtrate was concentrated *in vacuo*. The residue was purified by silica gel column chromatograph, eluting with Hexanes: EtOAc [2.0:1 (v/v)] to give **6a** (42.3 mg, 0.097 mmol, 97% yield) as a white solid. $\mathbf{R}_f$ = 0.45 [Hexanes: EtOAc 2:1 (v/v)]. **^{1}H NMR** (500 MHz, $CDCl_3$, 25 ºC, δ): 7.29 (dd, *J* = 10.4, 4.5 Hz, 2H), 7.22 – 7.15 (m, 3H), 6.18 (d, *J* = 1.3 Hz, 1H), 5.34 (dd, *J* = 9.7, 5.2 Hz, 1H), 5.16 (t, *J* = 9.8 Hz, 1H), 4.19 (dd, *J* = 12.3, 4.6 Hz, 1H), 4.10 (dd, *J* = 12.3, 2.3 Hz, 1H), 4.01 (ddd, *J* = 9.9, 4.4, 2.4 Hz, 1H), 2.86 – 2.78 (m, 1H), 2.56 (dt, *J* = 13.9, 8.3 Hz, 1H), 2.25 – 2.19 (m, 1H), 2.14 (s, 3H), 2.07 (s, 3H), 2.05 – 2.01 (m, 4H), 1.99 (s, 3H), 1.86 – 1.77 (m, 1H). **^{13}C NMR** (125 MHz, $CDCl_3$, 25 ºC, δ): 170.79, 170.08, 169.75, 169.19, 140.98, 128.65, 128.50, 126.35, 93.25, 70.63, 70.43, 66.15, 62.39, 41.38, 33.72, 26.79, 21.21, 20.95, 20.84, 20.80. **HRMS** (ESI-TOF) *m/z* calcd for $C_{22}H_{29}O_9$ [$(M + H)^+$], 437.1806, found, 437.1800.

Cyclopropanation of C2-Ketonylsugar:

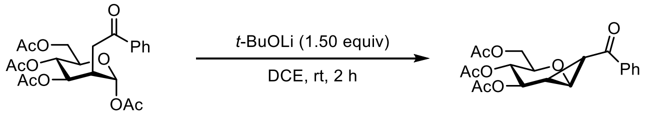

3a **7a**, 90%

To a solution of compound **3a** (45.0 mg, 0.100 mmol) in DCE (1.00 mL, 0.100 M) were added *t*-BuOLi (12.0 mg, 0.150 mmol, 1.50 equiv) under nitrogen gas atmosphere. The reaction mixture was stirred at room temperature for 1 h, the reaction mixture was concentrated *in vacuo*. The residue was purified by silica gel column chromatograph, eluting with Hexanes: EtOAc [2.0:1 (v/v)] to give **7a** (35.1 mg, 0.090 mmol, 90% yield) as a white solid. $\mathbf{R}_f$ = 0.35 [Hexanes: EtOAc 2:1 (v/v)]. **^{1}H NMR** (500 MHz, $CDCl_3$, 25 ºC, δ): 8.02 (d, *J* = 7.5 Hz, 2H), 7.61 (t, *J* = 7.3 Hz, 1H), 7.51 (t, *J* = 7.6 Hz, 2H), 5.54 (t, *J* = 7.6 Hz, 1H), 5.04 – 4.92 (m, 1H), 4.34 – 4.23 (m, 2H), 4.08 (d, *J* = 11.4 Hz, 1H), 3.77 (dd, *J* = 10.2, 2.8 Hz, 1H), 3.12 (d, *J* = 4.8 Hz, 1H), 2.56 (dd, *J* = 13.0, 6.6 Hz, 1H), 2.09 (s, 3H), 2.06 (s, 3H), 2.02 (s, 3H). **^{13}C NMR** (125 MHz, $CDCl_3$, 25 ºC, δ): 195.66, 170.78, 170.55, 169.96, 136.98, 133.57, 128.85, 128.38, 75.23, 70.44, 69.28, 64.15, 61.96, 31.60, 28.84, 21.06, 20.90. **HRMS** (ESI-TOF) *m/z* calcd for $C_{20}H_{23}O_8$ $[(M + H)^+]$, 391.1387, found, 391.1382.

Cyclization:

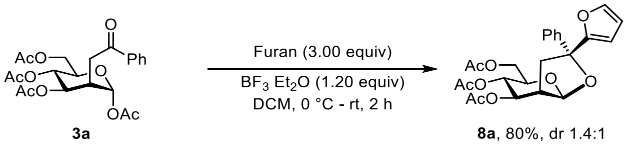

To a solution of compound **3a** (45.0 mg, 0.100 mmol) in DCM (1.00 mL, 0.100 M) were added furan (22 uL, 0.300 mmol, 3.0 equiv) and $BF_3{\cdot}Et_2O$ (14.8 uL, 0.120 mmol, 1.20 equiv) under nitrogen gas atmosphere at 0 °C. Then, the reaction was stirred at room temperature for 2 h. Saturated $NaHCO_3$ solution (4 mL) was added, the reaction mixture was extracted with DCM (10 mL) for 3 times. The combined organic layers were washed with satd. $NaHCO_3$, brine, dried over Mg_2SO_4, and filtered. The filtrate was concentrated *in vacuo* and the residue was purified by silica gel column chromatograph, eluting with Hexanes: EtOAc [2.0:1 (v/v)] to give **8a** (36.5 mg, 0.0797 mmol, 90% yield, dr = 1.4:1) as a white solid. $\mathbf{R}_f$ = 0.40 [Hexanes: EtOAc 2:1 (v/v)]. Data for product (**8a**): **^{1}H NMR** (700 MHz, $CDCl_3$, 25 ºC, δ): 7.46 (d, *J* = 7.9 Hz, 2H), 7.34 (dd, *J* = 20.1, 12.6 Hz, 3H), 7.28 (d, *J* = 7.2 Hz, 1H), 6.31 – 6.24 (m, 1H), 6.01 (d, *J* = 3.1 Hz, 1H), 5.45 (d, *J* = 3.1 Hz, 1H), 5.31 (dd, *J* = 9.3, 6.6 Hz, 1H), 5.14 (t, *J* = 9.7 Hz, 1H), 4.32 – 4.17 (m, 1H), 4.05 (dd, *J* = 12.0, 4.3 Hz, 1H), 3.61 (d, *J* = 9.9 Hz, 1H), 3.28 (tdd, *J* = 10.2, 6.9, 3.4 Hz, 1H), 2.78 (dd, *J* = 12.6, 7.3 Hz, 1H), 2.55 (t, *J* = 12.8 Hz, 1H), 2.06 (s, 3H), 2.03 (s, 3H), 1.91 (s, 3H). **^{13}C NMR** (125 MHz, $CDCl_3$, 25 ºC, δ): 170.84, 170.41, 170.04, 156.94, 144.11, 142.68, 128.09, 127.56, 125.97, 110.24, 107.76, 101.40, 86.76, 71.79, 71.14, 66.51, 62.15, 45.09, 37.27, 21.01, 20.91, 20.77. **HRMS** (ESI-TOF) *m/z* calcd for $C_{24}H_{27}O_9$ $[(M + H)^+]$, 459.1650, found, 459.1651.

Data for product (**8a-*epimer***): **^{1}H NMR** (700 MHz, $CDCl_3$, 25 ºC, δ): 7.40 (d, *J* = 7.6 Hz, 2H), 7.35 (dd, *J* = 16.7, 9.3 Hz, 3H), 7.29 (t, *J* = 7.2 Hz, 1H), 6.28 (dd, *J* = 3.2, 1.7 Hz, 1H), 6.10 (d, *J* = 3.2 Hz, 1H), 5.44 (d,

J = 3.4 Hz, 1H), 5.28 (d, *J* = 6.3 Hz, 2H), 4.23 – 4.13 (m, 2H), 3.65 (dd, *J* = 4.8, 3.1 Hz, 1H), 3.13 (t, *J* = 12.7 Hz, 1H), 2.86 – 2.74 (m, 1H), 2.38 (dd, *J* = 12.3, 7.1 Hz, 1H), 2.08 (s, 2H), 2.05 (s, 2H), 2.00 (s, 2H). **^{13}C NMR** (175 MHz, $CDCl_3$, 25 ºC, δ): 170.95, 170.25, 156.27, 143.04, 142.97, 128.23, 127.76, 125.97, 125.89, 110.29, 108.69, 101.10, 86.01, 71.48, 71.22, 66.80, 62.71, 43.36, 36.35, 20.96, 20.93. **HRMS** (ESI-TOF) *m/z* calcd for $C_{24}H_{27}O_9$ [(M + H)$^+$], 459.1650, found, 459.1652.

N-Glycosylation of C2-Ketonylsugar:

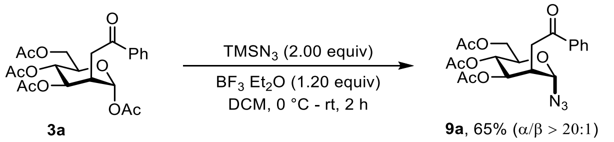

To a solution of compound **3a** (45.0 mg, 0.100 mmol) in DCM (1.00 mL, 0.100 M) were added $TMSN_3$ (26.3 uL, 0.200 mmol, 2.00 equiv) and $BF_3{\cdot}Et_2O$ (14.8 uL, 0.120 mmol, 1.20 equiv) at 0 °C. The reaction was stirred at room temperature for 2 h. Saturated $NaHCO_3$ solution (4 mL) was added, the reaction mixture was extracted with DCM (10 mL) for 3 times. The combined organic layers were washed with satd. $NaHCO_3$, brine, dried over Mg_2SO_4, and filtered. The filtrate was concentrated *in vacuo* and the residue was purified by silica gel column chromatograph, eluting with Hexanes: EtOAc [2.0:1 (v/v)] to give **9a** (28.1 mg, 0.0649 mmol, 65% yield, α/β > 20:1) as a white solid. **R*f*** = 0.45 [Hexanes: EtOAc 2:1 (v/v)]. **^{1}H NMR** (500 MHz, $CDCl_3$, 25 ºC, δ): 8.03 – 7.96 (m, 2H), 7.61 (t, *J* = 7.4 Hz, 1H), 7.51 (t, *J* = 7.7 Hz, 2H), 5.45 (dd, *J* = 9.9, 5.3 Hz, 1H), 5.41 (d, *J* = 1.0 Hz, 1H), 5.15 (t, *J* = 9.7 Hz, 1H), 4.34 – 4.23 (m, 1H), 4.20 – 4.11 (m, 2H), 3.35 (dd, *J* = 18.1, 3.3 Hz, 1H), 3.16 (dd, *J* = 18.1, 10.0 Hz, 1H), 3.00 (ddd, *J* = 6.8, 4.2, 1.7 Hz, 1H), 2.11 (s, 3H), 2.06 (s, 3H), 1.98 (s, 3H). **^{13}C NMR** (125 MHz, $CDCl_3$, 25 ºC, δ): 197.39, 170.72, 170.13, 169.61, 136.51, 133.84, 128.94, 128.19, 89.67, 70.39, 69.18, 66.41, 62.43, 38.39, 34.16, 20.92, 20.88, 20.86. **HRMS** (ESI-TOF) *m/z* calcd for $C_{20}H_{27}N_4O_8$ [(M + NH_4)$^+$], 451.1823, found, 451.1818.

S-Glycosylation of C2-Ketonylsugar:

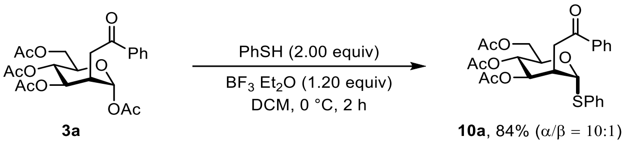

To a solution of compound **3a** (45.0 mg, 0.100 mmol) in DCM (1.00 mL, 0.100 M) were added PhSH (20.0 uL, 0.200 mmol, 2.00 equiv) and $BF_3{\cdot}Et_2O$ (14.8 uL, 0.120 mmol, 1.20 equiv) at 0 °C. The reaction was stirred at 0 °C for 2 h. Saturated $NaHCO_3$ solution (4 mL) was added, the reaction mixture was extracted with DCM (10 mL) for 3 times. The combined organic layers were washed with satd. $NaHCO_3$, brine, dried over Mg_2SO_4, and filtered. The filtrate was concentrated *in vacuo* and the residue was purified by silica gel

column chromatograph, eluting with Hexanes: EtOAc [2.0:1 (v/v)] to give **10a** (42.0 mg, 0.0840 mmol, 84% yield, α/β = 10:1) as a white solid. **R$_f$** = 0.45 [Hexanes: EtOAc 2:1 (v/v)]. Data for the α anomer: **^{1}H NMR** (500 MHz, $CDCl_3$, 25 ºC, δ): 8.01 – 7.95 (m, 2H), 7.60 (t, *J* = 7.4 Hz, 1H), 7.49 (dd, *J* = 11.1, 4.3 Hz, 4H), 7.32 – 7.23 (m, 3H), 5.54 (dd, *J* = 9.6, 4.6 Hz, 1H), 5.51 (s, 1H), 5.18 (t, *J* = 9.8 Hz, 1H), 4.60 (ddd, *J* = 9.8, 5.8, 2.2 Hz, 1H), 4.26 (dd, *J* = 12.2, 5.8 Hz, 1H), 4.10 (dd, *J* = 12.2, 2.3 Hz, 1H), 3.42 (dt, *J* = 11.9, 7.4 Hz, 1H), 3.33 (td, *J* = 8.9, 4.6 Hz, 2H), 2.09 (s, 3H), 2.04 (s, 3H), 1.99 (s, 3H). **^{13}C NMR** (125 MHz, $CDCl_3$, 25 ºC, δ): 197.49, 170.68, 170.24, 169.65, 136.66, 133.90, 133.69, 131.84, 129.15, 128.88, 128.19, 127.73, 87.06, 70.28, 69.12, 67.31, 62.80, 39.74, 35.90, 20.94, 20.91, 20.86. **HRMS** (ESI-TOF) *m/z* calcd for $C_{26}H_{32}NO_8S$ [(M + NH_4)$^+$], 518.1843, found, 518.1835.

O-Glycosylation of C2-Ketonylsugar:

(2R,3S,4R,5S,6S)-2-(acetoxymethyl)-6-(((3S,5S,8R,9S,10S,13S,14S)-10,13-dimethyl-17-oxohexadecahydro-1H-cyclopenta[a]phenanthren-3-yl)oxy)-5-(2-oxo-2-phenylethyl)tetrahydro-2H-pyran-3,4-diyl diacetate (12a):

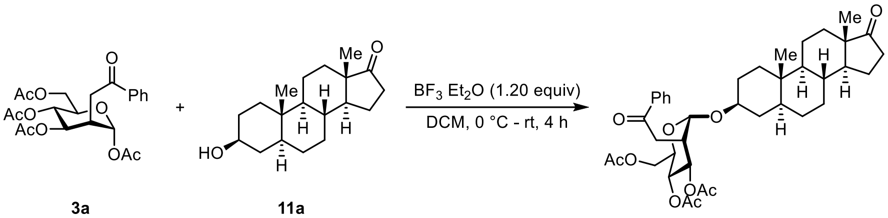

3a 11a 12a, 74% (α/β = 17:1)

To a solution of compound **3a** (45.0 mg, 0.100 mmol) in DCM (1.00 mL, 0.100 M) were added **11a** (58.1 mg, 0.200 mmol, 2.00 equiv) and $BF_3 \cdot Et_2O$ (14.8 uL, 0.120 mmol, 1.20 equiv) at 0 °C. The reaction was stirred at room temperature for 4 h. Saturated $NaHCO_3$ solution (4 mL) was added, the reaction mixture was extracted with DCM (10 mL) for 3 times. The combined organic layers were washed with satd. $NaHCO_3$ (5 mL), brine (5 mL), dried over Mg_2SO_4, and filtered. The filtrate was concentrated *in vacuo* and the residue was purified by silica gel column chromatograph, eluting with Hexanes: EtOAc [2.5:1 (v/v)] to give **12a** (50.3 mg, 0.0740 mmol, 74% yield, α/β = 17:1) as a white solid. **R$_f$** = 0.55 [Hexanes: EtOAc 2:1 (v/v)]. Data for the α anomer: **^{1}H NMR** (500 MHz, $CDCl_3$, 25 ºC, δ): 7.99 (d, *J* = 7.4 Hz, 2H), 7.59 (t, *J* = 7.3 Hz, 1H), 7.48 (t, *J* = 7.7 Hz, 2H), 5.57 (dd, *J* = 9.9, 5.5 Hz, 1H), 5.12 (t, *J* = 10.0 Hz, 1H), 4.21 (dd, *J* = 12.2, 5.2 Hz, 1H), 4.11 (dd, *J* = 12.5, 4.9 Hz, 2H), 3.56 – 3.47 (m, 1H), 3.34 (dd, *J* = 17.8, 3.2 Hz, 1H), 3.11 (dd, *J* = 17.9, 9.8 Hz, 1H), 3.03 (dd, *J* = 9.0, 4.4 Hz, 1H), 2.42 (dd, *J* = 19.3, 8.8 Hz, 1H), 2.09 (s, 3H), 2.04 (s, 3H), 1.94 (s, 3H), 1.93 – 1.88 (m, 2H), 1.82 – 1.70 (m, 3H), 1.67 – 1.39 (m, 5H), 1.36 – 1.21 (m, 5H), 1.20 (d, *J* = 6.1 Hz, 3H), 1.13 – 1.05 (m, 1H), 0.99 – 0.87 (m, 2H), 0.85 (d, *J* = 3.7 Hz, 6H), 0.67 (td, *J* = 11.8, 3.7 Hz, 1H). **^{13}C NMR** (100 MHz, $CDCl_3$, 25 ºC, δ): 221.41, 198.07, 170.77, 170.26, 169.82, 136.83, 133.55, 128.83,

128.16, 98.42, 77.59, 70.04, 68.14, 67.29, 64.55, 62.99, 54.55, 51.53, 47.93, 45.17, 39.47, 36.91, 35.98, 35.91, 35.90, 35.17, 34.50, 31.67, 31.04, 28.65, 27.77, 25.49, 21.90, 21.00, 20.93, 20.91, 20.60, 13.94, 12.42. **HRMS** (ESI-TOF) *m/z* calcd for $C_{39}H_{56}NO_{10}$ $[(M + H)^+]$, 698.3899, found, 698.3885.

(2R,3R,4S,5R,6S)-2-((((2S,3S,4R,5S,6R)-4,5-diacetoxy-6-(acetoxymethyl)-3-(2-oxo-2-phenylethyl)tetrahydro-2H-pyran-2-yl)oxy)methyl)-6-methoxytetrahydro-2H-pyran-3,4,5-triyl tribenzoate (14a):

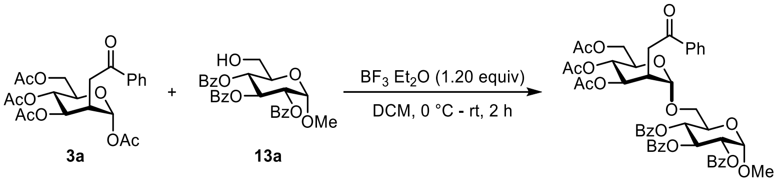

14a, 74% (α/β = 7.0:1)

To a solution of compound **3a** (45.0 mg, 0.100 mmol) in DCM (1.00 mL, 0.100 M) were added **13a** (101.3 mg, 0.200 mmol, 2.00 equiv) and $BF_3{\cdot}Et_2O$ (14.8 uL, 0.120 mmol, 1.20 equiv) at 0 °C. The reaction was stirred at at room temperature for 4 h. Saturated $NaHCO_3$ solution (4 mL) was added, the reaction mixture was extracted with DCM (10 mL) for 3 times. The combined organic layers were washed with satd. $NaHCO_3$, brine, dried over Mg_2SO_4, and filtered. The filtrate was concentrated *in vacuo* and the residue was purified by silica gel column chromatograph, eluting with Hexanes: EtOAc [2.0:1 (v/v)] to give **14a** (66.3 mg, 0.0740 mmol, 74% yield, α/β = 7.0:1) as a white solid. $\mathbf{R_f}$ = 0.55 [Hexanes: EtOAc 2:1 (v/v)]. Data for the α anomer: **^{1}H NMR** (700 MHz, $CDCl_3$, 25 ºC, δ): 8.10 (d, *J* = 7.3 Hz, 2H), 8.05 (d, *J* = 7.4 Hz, 2H), 7.96 (d, *J* = 7.4 Hz, 2H), 7.64 (d, *J* = 7.4 Hz, 2H), 7.58 (t, *J* = 7.4 Hz, 1H), 7.54 – 7.33 (m, 9H), 7.31 (t, *J* = 7.6 Hz, 2H), 6.18 – 6.11 (m, 1H), 5.34 (dd, *J* = 6.7, 4.4 Hz, 1H), 5.14 (ddd, *J* = 12.1, 7.8, 3.8 Hz, 3H), 4.97 – 4.91 (m, 1H), 4.80 (d, *J* = 11.8 Hz, 1H), 4.59 (dd, *J* = 11.9, 3.6 Hz, 1H), 4.25 – 4.19 (m, 3H), 4.13 – 4.09 (m, 1H), 4.02 (dd, *J* = 12.2, 2.1 Hz, 1H), 3.44 (s, 3H), 2.93 – 2.86 (m, 1H), 2.85 (d, *J* = 6.4 Hz, 2H), 2.06 (s, 3H), 2.04 (s, 3H), 1.72 (s, 3H). **^{13}C NMR** (175 MHz, $CDCl_3$, 25 ºC, δ): 196.60, 170.70, 170.01, 169.30, 166.36, 166.10, 165.60, 136.50, 133.48, 133.39, 133.29, 130.10, 129.99, 129.88, 129.85, 129.70, 129.12, 128.68, 128.58, 128.53, 128.49, 128.00, 100.65, 96.88, 74.84, 72.83, 72.39, 69.87, 69.56, 68.49, 67.52, 64.59, 63.64, 62.64, 55.58, 37.78, 34.35, 25.51, 20.93, 20.86, 20.59. **HRMS** (ESI-TOF) *m/z* calcd for $C_{48}H_{52}NO_{17}$ $[(M + NH_4)^+]$, 914.3230, found, 914.3224.

Mechanistic Studies

Radical Trapping Experiment

The procedure is based on General Procedure C: In a glovebox, to an oven-dried 4 mL screw cap vial was added $Pd(PPh_3)_4$ (1.16 mg, 1.00 µmol, 5.00 mol%), Xantphos (0.69 mg, 1.20 µmol, 6.00 mol%), 1-bromo-sugar **1a** (8.22 mg, 0.0200 mmol, 1.00 equiv), KOAc (2.94 mg, 0.0300 mmol, 1.50 equiv), TEMPO (3.13 mg, 0.020 mmol, 1.00 equiv), silyl enol ether (11.54 mg, 0.060 mmol, 3.00 equiv) and benzene (1.33 mL, 0.015 M). The vial equipped with a magnetic stir bar and capped with septum cap. Next, the vial was taken out of the glovebox and sealed with black tape. The reaction mixture was stirred at 90 °C, irradiated with 36 W Blue LEDs for 20 h. The yield was determined based on crude ^{1}H-NMR spectrum with dibromomethane as an internal standard.

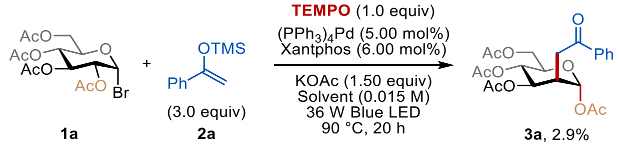

Fig. S1. Radical trapping experiment with TEMPO.

Results and Conclusion: The yield of the desired product decreased significantly in the presence of a radical scavenger, 2,2,6,6-tetramethylpiperidine 1-oxyl radical (TEMPO, 1.00 equiv), which indicated that the reaction likely proceeds through a radical mechanism.

Radical-clock Experiment

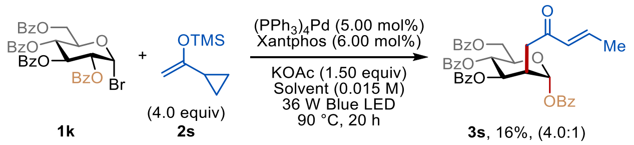

Fig. S2. Radical-clock experiment.

According to the General Procedure C, the title compound was obtained as a white solid (21.0 mg, 0.032 mmol, 16% yield, axial: equatorial = 3.0:1). $\mathbf{R_f}$ = 0.45 [Hexanes: EtOAc 2:1 (v/v)].

Data for axial product (**3s-*ax***): **^{1}H NMR** (700 MHz, $CDCl_3$, 25 °C, δ): 8.19 (d, *J* = 7.1 Hz, 2H), 8.01 (d, *J* = 7.1 Hz, 2H), 7.96 (d, *J* = 7.2 Hz, 2H), 7.93 – 7.91 (m, 2H), 7.68 – 7.63 (m, 1H), 7.58 – 7.46 (m, 5H), 7.42 – 7.30 (m, 6H), 6.84 (dt, *J* = 22.6, 6.8 Hz, 1H), 6.35 (d, *J* = 1.4 Hz, 1H), 6.12 (dd, *J* = 15.8, 1.6 Hz, 1H), 6.05 (dd, *J* = 9.8, 5.3 Hz, 1H), 5.84 (t, *J* = 9.8 Hz, 1H), 4.59 (d, *J* = 9.6 Hz, 2H), 4.48 – 4.36 (m, 1H), 3.45 (dd, *J* = 5.4, 3.6 Hz, 1H), 3.10 (dd, *J* = 17.6, 4.1 Hz, 1H), 2.99 (dd, *J* = 17.6, 9.3 Hz, 1H), 2.70 (ddd, *J* = 25.2, 17.4, 6.4 Hz, 1H), 1.87 (dd, *J* = 6.8, 1.5 Hz, 3H). **^{13}C NMR** (175 MHz, $CDCl_3$, 25 °C, δ): 196.46, 166.11, 165.74, 165.56, 164.47, 144.00, 133.88, 133.68, 133.53, 133.23, 131.75, 130.21, 129.97, 129.91, 129.85, 129.82, 128.97, 128.85, 128.60, 128.52, 94.07, 70.86, 70.66, 66.92, 63.04, 37.97, 35.64, 18.47. **HRMS** (ESI-TOF) *m/z* calcd for $C_{39}H_{38}NO_{10}$ [$(M + Na)^+$], 680.2490, found, 680.2480.

Results and Conclusion: The formation of ring-opening product suggested that radical intermediates are involved.

Studies of stereochemical outcome using 2-iodo sugar

The procedure is based on General Procedure C: In a glovebox, to an oven-dried 4 mL screw cap vial was added $Pd(PPh_3)_4$ (1.16 mg, 1.00 μmol, 5.00 mol%), Xantphos (0.69 mg, 1.20 μmol, 6.00 mol%), 2-Iodo-sugar **15a** or **16a** (9.16 mg, 0.0200 mmol, 0.100 equiv), KOAc (2.94 mg, 0.0300 mmol, 1.50 equiv), silyl enol ether (11.54 mg, 0.060 mmol, 3.00 equiv) and benzene (1.33 mL, 0.015 M). The vial equipped with a magnetic stir bar and capped with septum cap. Next, the vial was taken out of the glovebox and sealed with black tape. The reaction mixture was stirred at 90 °C, irradiated with 36 W Blue LEDs for 20 h. The yield and selectivity were determined based on crude ^1H-NMR spectrum with dibromomethane as an internal standard.

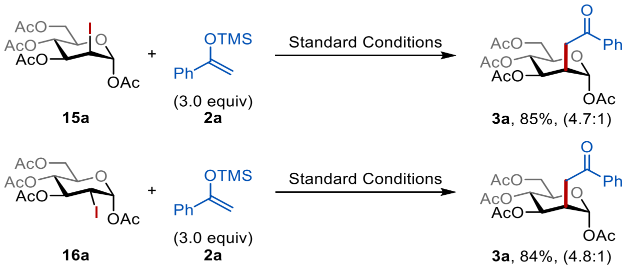

Fig. S3. Studies of stereochemical outcome using 2-iodo sugar.

Results and Conclusion: The reaction involves the formation of a common C2-radical species.

Cross-over experiment

The procedure is based on General Procedure C: In a glovebox, to an oven-dried 4 mL screw cap vial was added $Pd(PPh_3)_4$ (1.16 mg, 1.00 μmol, 5.00 mol%), Xantphos (0.69 mg, 1.20 μmol, 6.00 mol%), 1-bromo-sugar **1a** (4.11 mg, 0.0100 mmol, 0.50 equiv), **1k** (6.55 mg, 0.0100 mmol, 0.50 equiv), KOAc (2.94 mg, 0.0300 mmol, 1.50 equiv), silyl enol ether (11.54 mg, 0.060 mmol, 3.00 equiv) and benzene (1.33 mL, 0.015 M). The vial equipped with a magnetic stir bar and capped with septum cap. Next, the vial was taken out of the glovebox and sealed with black tape. The reaction mixture was stirred at 90 °C, irradiated with 36 W Blue LEDs for 20 h. At the end of the reaction, **17a** and **18a** were not detected on both LC-MS spectrum and crude ^1H-NMR spectrum. The yield and selectivity of **3a** and **4a** were determined based on crude ^1H-NMR spectrum with dibromomethane as an internal standard.

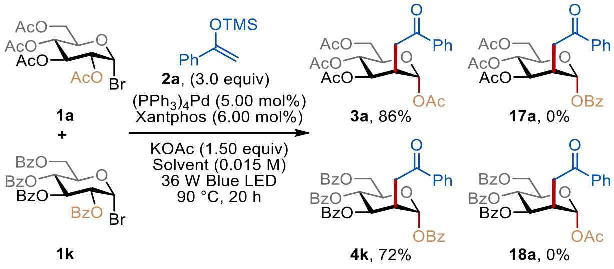

Fig. S4. Cross-over experiments.

Results and Conclusion: No cross-over products (**17a** and **18a**) were observed, the acyloxyl migration is likely proceeded through a in-cage or a concerted mechanism.

Stern–Volmer Luminescence Quenching Experiments

All quenching data was recorded in the dark at 23 ˚C. A 1.00 cm screw-top quartz cuvette was charged with $Pd(PPh_3)_4$ (0.400 μM) and varying concentration of quencher in degassed benzene. In a fluorometer, excitation was performed at 375 nm and emission was detected at 580 nm. After the acquisition, the data was plotted according to the Stern-Volmer equation shown below.

$$\mathbf{I_o/I = 1 + K_{SV}[Q]}$$

$$\mathbf{K_{SV}} = \boldsymbol{k_q}\tau_o$$

I_o is the luminescence intensity in the absence of quencher, I is the intensity in the presence of quencher, K_{SV} is the Stern−Volmer constant, k_q is the quenching rate, τ_o is the life-time of the photoredox catalyst and [Q] is the concentration of quencher.

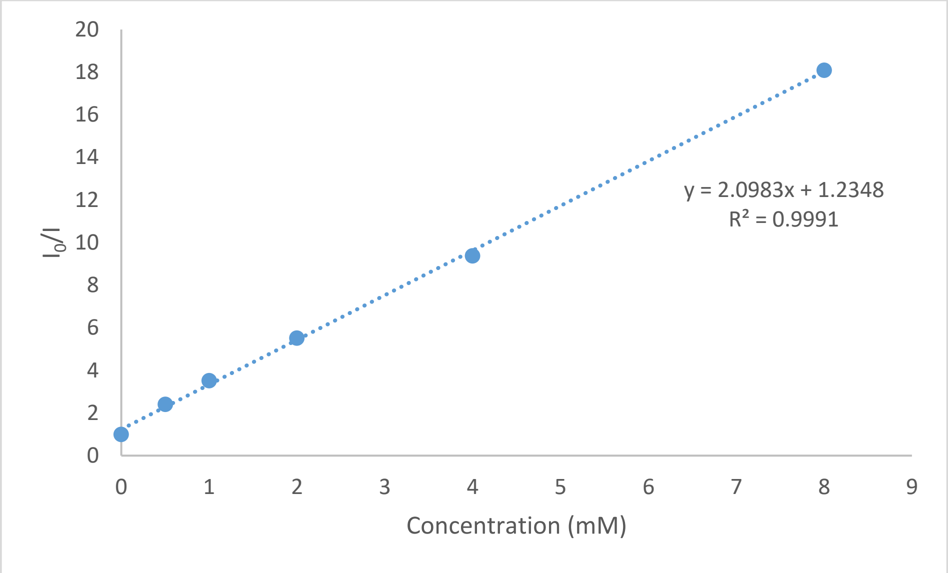

Fig. S5. Stern-Volmer plot for the emission quenching of $Pd(PPh_3)_4$ by various concentrations of 1-bromosugar **1a** (Benzene was used as the solvent).

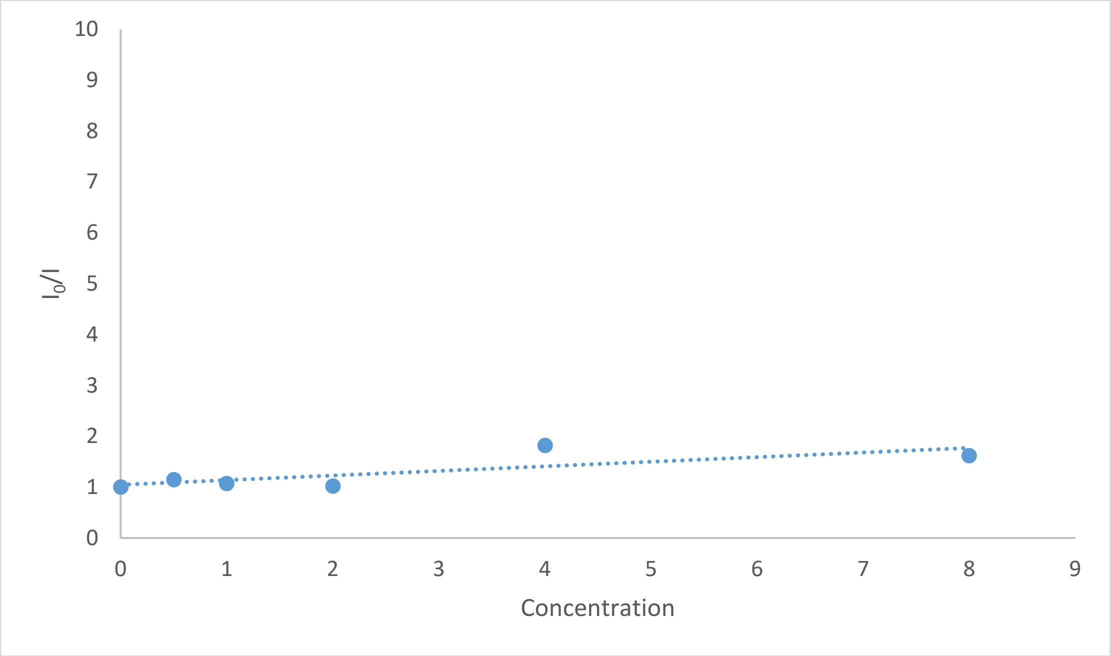

Fig. S6. Stern-Volmer plot for the emission quenching of $Pd(PPh_3)_4$ by various concentrations of phenyl silyl enol ether **2a** (Benzene was used as the solvent).

Results and Conclusion: Stern-Volmer Luminescence Quenching indicated that 1-bromosugar quenches the excited state of $Pd(PPh_3)_4$, more efficiently than silyl enol ether **2a**. Stern-Volmer quenching of $Pd(PPh_3)_4$ in the presence of Xantphos ligand generated a weak emission, where ligand exchange or dissociation is predicted to cause decreased emission measurements.[20]

Quantum Yield Experiment

The following quantum yield calculation was adapted from the procedure reported by Yoon et al.[21]

Determination of the Light Intensity at 450 nm:

The fraction of light absorbed (*f*) by ferrioxalate solution was calculated as shown in Fig. S7, where the absorbance of the ferrioxalate solution at 450 nm was measured to be 1.70593 (A), based on the equation ($f = 1\text{-}10^{-A}$), indicating $f = 0.98032$.

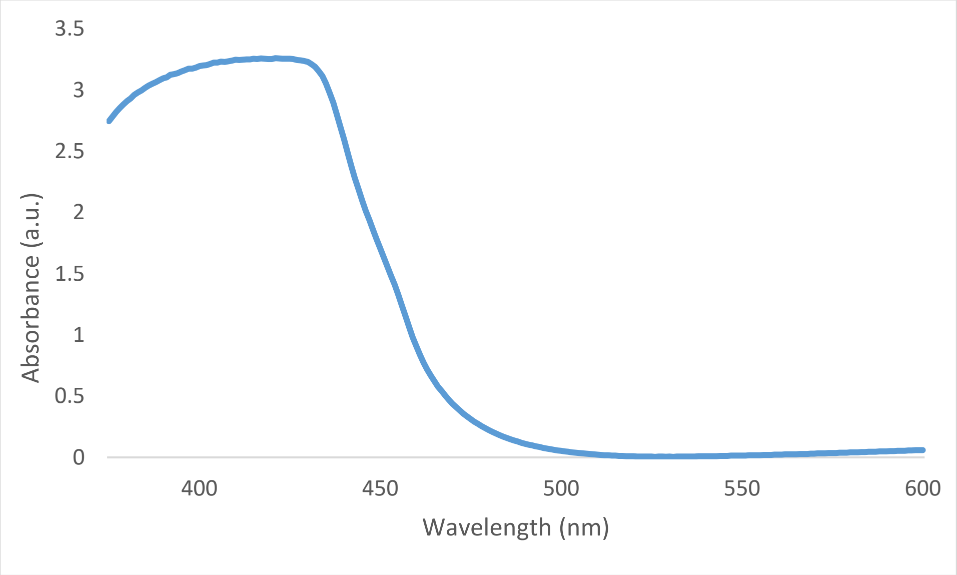

Fig. S7. Absorbance of the ferrioxalate solution at 450 nm (A = 1.70593).

The photon flux of the 30 W Blue LEDs (λ_{max} = 450 nm) was determined by standard ferrioxalate actinometry.[22] A 0.150 M solution of ferrioxalate was prepared by dissolving 2.21 g of potassium ferrioxalate hydrate ($K_3[Fe(C_2O_4)_3] \cdot 3\ H_2O$) in 30.0 mL of 0.05 M H_2SO_4 (aq). Next, a buffered solution of phenanthroline was prepared by dissolving 50.0 mg of phenanthroline and 11.25 g of sodium acetate in 50.0 mL of 0.500 M H_2SO_4. Both solutions were stored in an amber vial in the dark. To determine the photon flux of the 30 W Blue LEDs, 2.00 mL of the ferrioxalate solution was placed in a cuvette and the cuvette was positioned with half of the solution submerged in an oil bath. The cuvette was irradiated for 5.00 seconds at λ = 450 nm. After irradiation, 0.500 mL of the phenanthroline solution was added to the cuvette. The solution was then rested for 1 h in the dark to allow the ferrous ions to completely coordinate to the phenanthroline. A non-irradiated sample was also prepared and developed in the dark *(note: after developing the non/irradiated samples they were diluted with a dilution factor of 4 to prevent deviation from the Beer-Lambert law at high concentrations A = >2. Thus, to obtain the actual mol of* Fe^{2+} *they were multiplied by four. The values of the optical difference are the average of three trials*).

1 Ferrioxalate Actinometry

$$\text{mol of Fe}^{2+} = 4 \times \left[\frac{V \times \Delta A_{510}}{l \times \varepsilon_{510}} \right]$$

$$\text{mol of Fe}^{2+} = 4 \times \left[\frac{0.00250 \times 0.5437}{1 \times 11{,}100} \right] \text{mol}$$

$$= 4.90 \times 10^{-7} \text{ mol}$$

V= 0.00250L *(total volume)*
ΔA_{510} = 0.5437 *(difference in absorption at 510 nm)*
l = 1.00 cm *(path length)*
ε_{510} = 11,100 L $mol^{-1}cm^{-1}$ *(molar absorptivity at 510 nm)*

2 Determination of photon flux of 30W Blue Led

$$\text{photon flux} = \left[\frac{\text{mol of Fe}^{2+}}{\phi \times t \times f} \right]$$

$$\text{photon flux} = \left[\frac{4.90 \times 10^{-7}}{1.01 \times 5.00 \times 0.98032} \right] \text{einstein s}^{-1}$$

$$= 9.894 \times 10^{-8} \text{ einstein s}^{-1}$$

ϕ = 1.01 *(quantum yield of ferrioxalate actiometer)*
t = 5.00s *(time)*
f = 0.98032 *(Fraction of light absorbed)*

Fig. S8. Determination of the light intensity (photon flux) at 450 nm via ferrioxalate actinometry (ε = 11,100 L $mol^{-1}cm^{-1}$).[22]

Afterward, the absorbance of both solutions was measured at 510 nm and with mol of Fe^{2+} known. Next the photon flux was determined to be 9.89×10^{-8} einstein s^{-1}. We can obtain the quantum yield of our reaction provided if it is irradiated using the same geometry (*note: although Φ = 1.01 at 436 nm was used for the calculation of the photon flux it is known that the ferrioxalate system varied little with the wavelength as the Φ remained between 0.9 and 1.1 at wavelength between 400–480 nm*).[16a]

Determination of Quantum Yield:

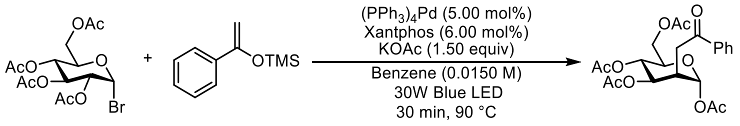

1a **2a**
ϕ = 0.009

To determine the quantum yield, in a glovebox, the cuvette was charged with **3b** (12.3 mg, 0.0300 mmol, 1.00 equiv), $Pd(PPh_3)_4$ (1.73 mg, 1.50 μmol, 5.00 mol%), Xantphos (1.04 mg, 1.8 μmol, 6.00 mol%), KOAc (4.42 mg, 0.0450 mmol, 1.5 equiv) and benzene (2.00 mL, 0.015 M). Afterward the cuvette was capped with a PTFE stopper and taken out of the glovebox. The cuvette was placed with half of the solvent in an oil bath at 90 ˚C. The reaction mixture was irradiated (λ_{max} = 450 nm) for 1800 s (30 min) with the same 30 W Blue LEDs. To determine the yield of the product, the solvent is removed under vacuum, an internal standard, dibromomethane (CH_2Br_2) (5.22 mg, 0.0300 mmol) was added to the cuvette, followed by 500 μL $CDCl_3$. The reaction was repeated three times with yield to be: 6.9%, 5.1%, 3.6%. The quantum yield was determined using the equation shown below.

1 Quantum Yield

$$\phi = \left[\frac{\text{mol x yield \%}}{\text{photon flux x t x f}} \right]$$

$$\phi = \left[\frac{0.00003 \times 5.2\%}{9.89 \times 10^{-8} \times 1800 \times 1} \right]$$

$$= 0.009$$

ϕ = quantum yield of the reaction
t = 1800s (time)
f = (Fraction of light absorbed)
yield% = averaged yield of three trials

Fig. S9. Quantum yield calculation.

Results and Conclusion: Quantum yield experiments suggest that an extended radical chain propagation is an unlikely mechanism under our reaction conditions.

X-Ray Crystal Structure

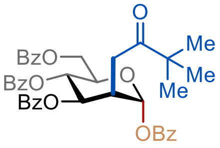

3o

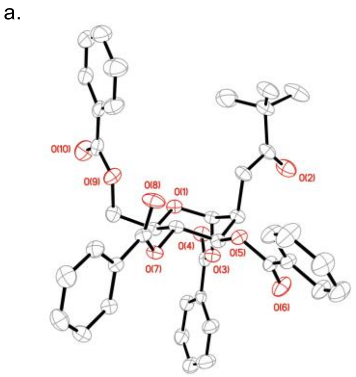

Fig. S10. (a) View of **3o** in the heteroatom labeling scheme. Displacement ellipsoids are scaled to the 50% probability level. The H atoms were omitted for clarity. (b) The 3D representation of **3o** was prepared from the CIF file using CYLview.[23]

Table S1. Crystal data and structure refinement for **3o**

Bond precision:	C-C = 0.0053 A		Wavelength=0.71073
Cell:	a=13.5907(8)	b=15.0346(9)	c=18.9632(12)
	alpha=97.644(2)	beta=101.994(2)	gamma=104.537(2)
Temperature:	213 K		

	Calculated	Reported
Volume	3598.0(4)	3598.0(4)
Space group	P1	P1
Hall group	P1	P1
Moiety formula	$C_{40}H_{38}O_{10}$	$C_{40}H_{38}O_{10}$
Sum formula	$C_{40}H_{38}O_{10}$	$C_{40}H_{38}O_{10}$
Mr	678.70	678.70
Dx, g cm^{-3}	1.253	1.253
Z	4	4
Mu (mm-1)	0.090	0.090
F000	1432.0	1432.0
F000’	1432.79	
h, k, lmax	18, 20, 25	18, 20, 25
Nref	35804[17902]	35804
Tmin, Tmax	0.985, 0.989	0.719, 0.746
Tmin’	0.982	

Correction method= # Reported T Limits: Tmin=0.719 Tmax=0.746

AbsCorr = MULTI-SCAN

Data completeness= 1.99/1.00 Theta(max)= 28.310

R(reflections)= 0.0481(20893) wR2(reflections)= 0.1022(35654)

S = 1.008 Npar= 1801

DFT Calculations

Computational Details: All density functional theory (DFT) calculations were carried out using Gaussian 16.[24] Geometries of intermediates and transition states were optimized using the dispersion-corrected B3LYP-D3 functional,[25] using Grimme's DFT-D3 dispersion correction,[26] with a mixed basis set of SDD for Pd and 6-31G(d) for other atoms in the gas phase. Vibrational frequency calculations were performed for all the stationary points to confirm if each optimized structure is a local minimum or a transition state structure. The M06 functional[27] with a mixed basis set of SDD for Pd and 6-311+G(d,p) for other atoms was used in single-point energy calculations. Solvation energy corrections were calculated in benzene solvent with the SMD continuum solvation model[28] based on the gas-phase optimized geometries. Thermal corrections to the Gibbs free energies and enthalpies were calculated using GoodVibes[29] with Truhlar's quasi-harmonic oscillator approximation[30] at 363.15 K. Because the default method in Gaussian 16 and GoodVibes to calculate translational entropies uses a gas-phase formula, *i.e.* the Sackur–Tetrode equation, the actual translation entropies in solution are significantly lower. Therefore, using the default gas-phase formula to calculate translation entropies would lead to error up to a few kilocalories per mole for Gibbs free energies of bimolecular reactions. To address this issue, we used the "free-volume model" originally developed by Whitesides et al.[31] to compute the translation entropies in solution. These calculations use the volume of benzene from DFT calculations using Gaussian 16 ($V_{benzene}$= 109.569 Å^3/mol) and the molar concentration of liquid benzene solvent ([X] = 11.3 mol/L) to obtain the free volume in benzene solution ($V_{free}^{benzene}$, eq. S1).

$$V_{free}^{benzene} = C_{free}\left(\sqrt[3]{\left(\frac{10^{27}}{[X]N_0}\right)} - \sqrt[3]{V_{benzene}}\right)^3 = 0.952\ \text{Å}^3/_{mol} \qquad (S1)$$

Here, N_0 is Avogado's number and C_{free} is equal to 8 due to the system being treated as 3D cubic array. The $V_{free}^{benzene}$ term is then implemented in eq. S2 with the molecular weight of benzene (M = 78.1 g/mol) and the reaction temperature to yield the corrected translational entropy in solution ($S_{trans}^{benzene}$).

$$\begin{aligned} S_{trans}^{benzene} &= 11.1 + 12.5\ \ln(T) + 12.5\ \ln(M) + 8.3\ln\left(V_{free}^{benzene}\right) \\ &= 136.4\,J\,mol^{-1}K^{-1}\ \text{(at 298.15K) and } 138.9\,J\,mol^{-1}K^{-1}\ \text{(at 363.15 K)} \end{aligned} \qquad (S2)$$

The $S_{trans}^{benzene}$ value is used in place of the original translational entropy value to determine the new adjusted Gibbs free energies. This free-volume model correction leads to corrections of −1.9 kcal/mol and −2.3 kcal/mol to the computed Gibbs free energies at 298.15 K and 363.15 K, respectively. While the translational entropy corrections do not affect the relative Gibbs free energies in unimolecular steps, such as the 1,2-radical migration, these corrections become evident when there is change in molarity from the energy zero in the reaction energy profile (e.g. in bimolecular steps such as glycosyl radical addition).

Conformational sampling of carbohydrate structures and transition states was carried out using the iterative metadynamic sampling and genetic crossover (iMTD-GC) method implemented in the CREST program[32] with the GFN2-xTB method.[33] Default settings in CREST were used in the conformational sampling and the forming/breaking bonds in the transition state structures were constrained to the

corresponding distances obtained from the DFT-optimized TS structures in the CREST conformational sampling. Low-energy conformers from CREST/xTB were then re-optimized using DFT at the M06/SDD-6-311+G(d,p)/SMD//B3LYP-D3/SDD-6-31G(d) level of theory. Only the lowest energy conformer from the DFT calculations were reported in this manuscript. DFT calculations were performed using a simplified model of the glucosyl radical (**II**), where OMe groups were used in place of the OAc groups at the C3, 4, and 6 positions of the pyranose ring. Images of 3D molecular structures were generated using CYLview 2.0.[23]

Results: DFT calculations showed that the addition of C2-radical **III** to the silyl enol ether *via* transition state **TS1-ax** to form **IV-ax** is 3.4 kcal/mol more favorable than the formation of the equatorial isomer **IV-eq** *via* **TS1-eq** (Fig. S11). Subsequent silyl enol ether formation from radical intermediate **IV-ax** could proceed through at least two different reaction pathways: (P1) the recombination of radical **IV-ax** with $[Pd^I]Br$ to form Pd^{II} intermediate **IV'-ax** followed by β-hydride elimination; (P2) a palladoradical β-H-atom abstraction. Preliminary DFT calculations showed that the formation of **IV'-ax** is endergonic by 9.3 kcal/mol, but the subsequent β-hydride elimination (**TS2**) requires one of the P-arms of the Xantphos ligand to be dissociated to free up an open-coordination site, leading to a relatively higher barrier ($\Delta G^{\ddagger}$ = 28.2 kcal/mol with respect to **IV-ax**). Consequently, intermediate **IV'-ax** is prone to undergo a reverse reaction to form radical **IV-ax** and $[Pd^I]Br$, especially under the photoexcitation conditions. Interestingly, DFT data suggests that the palladoradical β-H-atom abstraction is more favorable than β-hydride elimination pathway by $\Delta\Delta G^{\ddagger}$ = –4.3 kcal/mol (**TS3** vs. **TS2**). Further examination will be necessary to distinguish these two reaction pathways and is the subject of future studies.

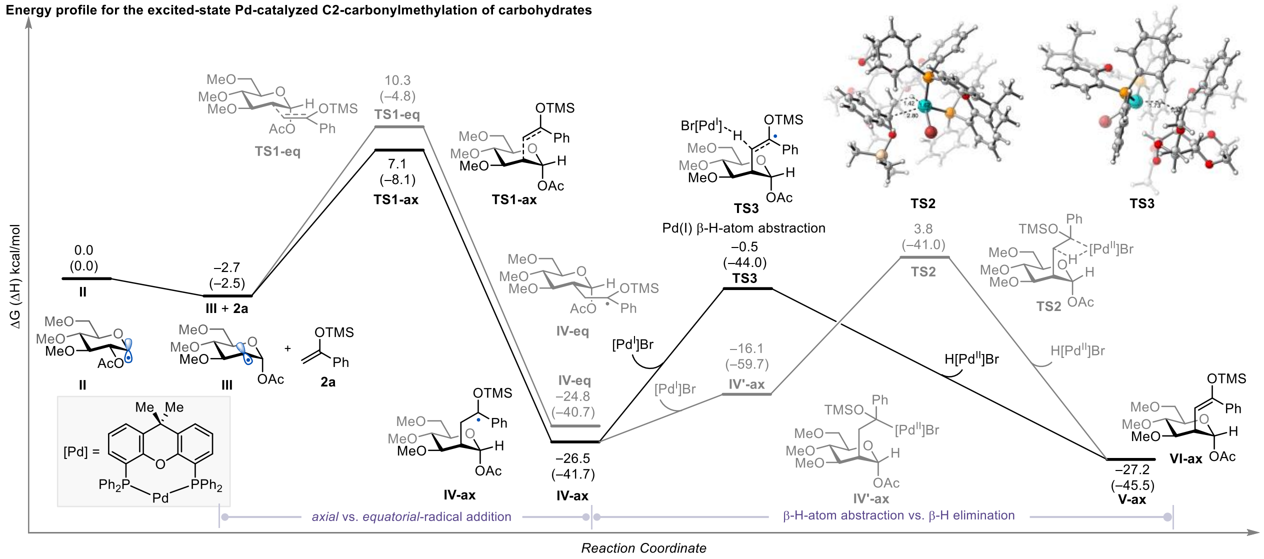

Fig. S11. Computed reaction energy profile of C2-ketonization at 90 °C. Calculations were performed at the M06/SDD–6-311+G(d,p)/(SMD, benzene)//B3LYP-D3/SDD–6-31G(d) level of theory. using a simplified model of the glucosyl radical (**II**), where the OMe groups were used in place of the OAc groups at the C3, C4, and C6 of the pyranose ring. Translational entropies in benzene solution were calculated using free-volume model.

Spectroscopic Data

^{1}H NMR (400 MHz, $CDCl_3$, 25 °C) of (2b)

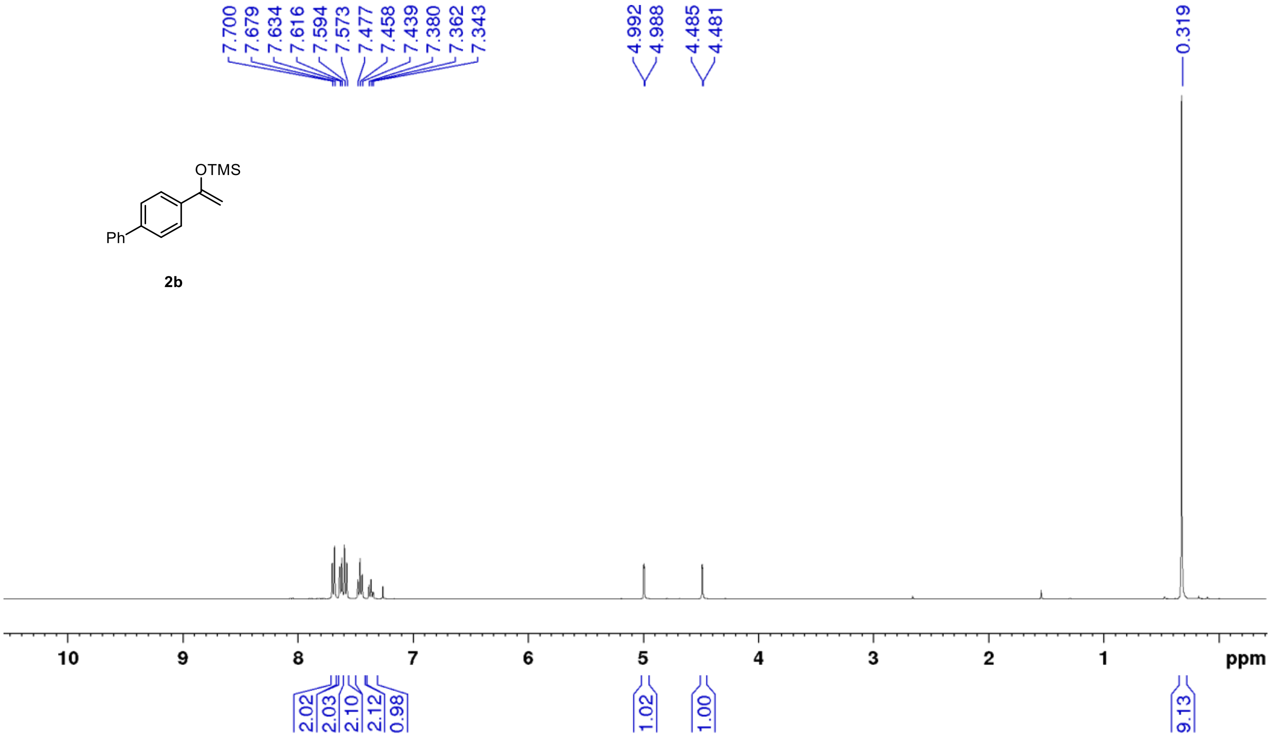

^{13}C NMR (100 MHz, $CDCl_3$, 25 °C) of (2b)

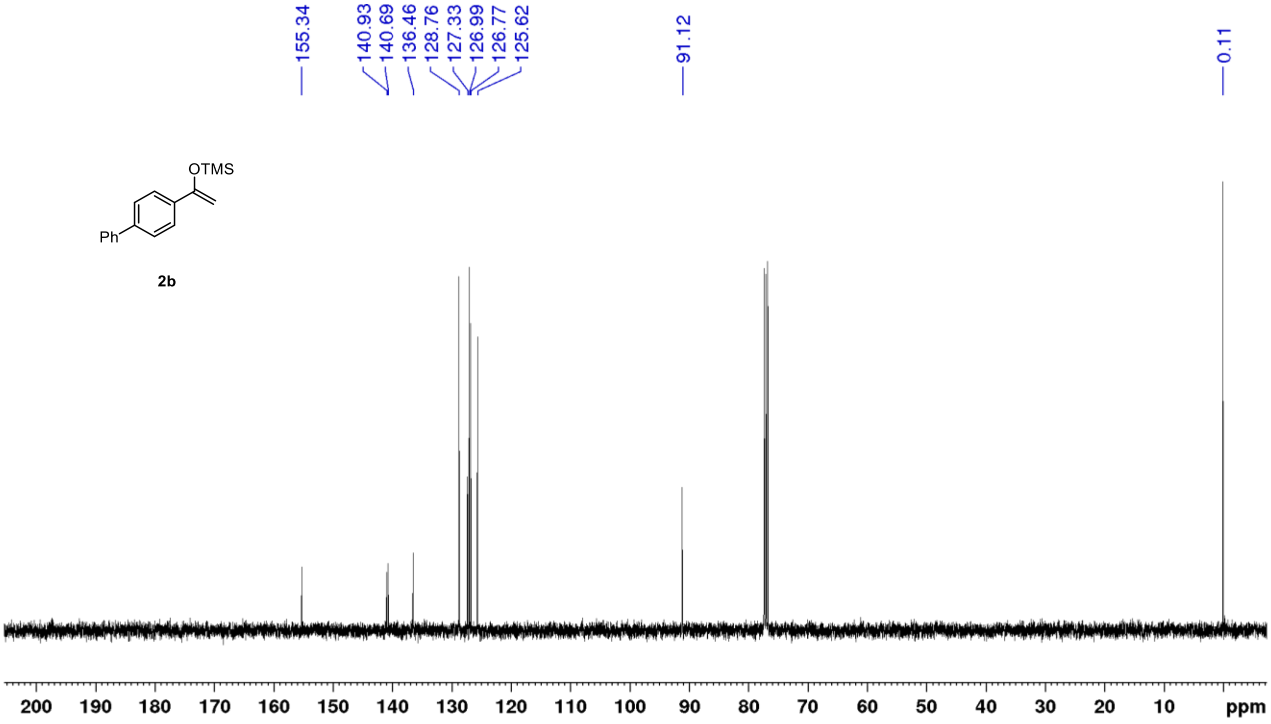

^{1}H NMR (400 MHz, $CDCl_3$, 25 ºC) of (2c)

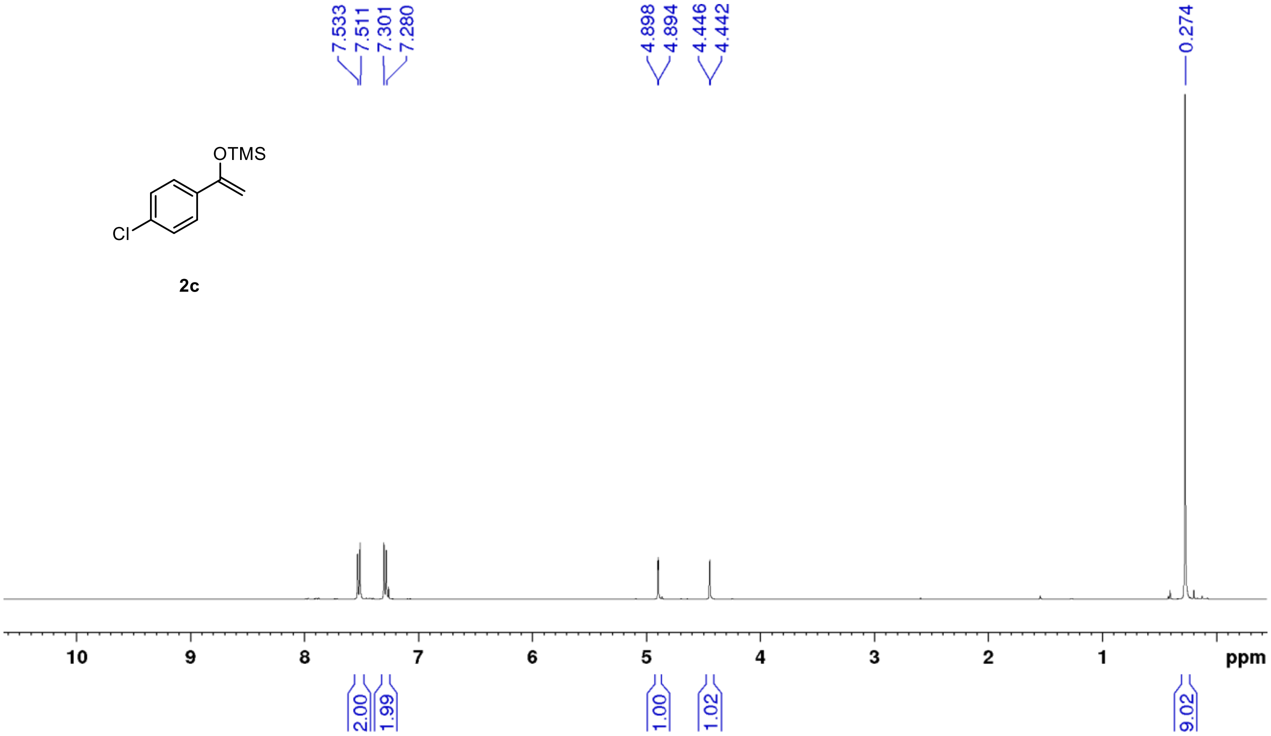

^{13}C NMR (100 MHz, $CDCl_3$, 25 ºC) of (2c)

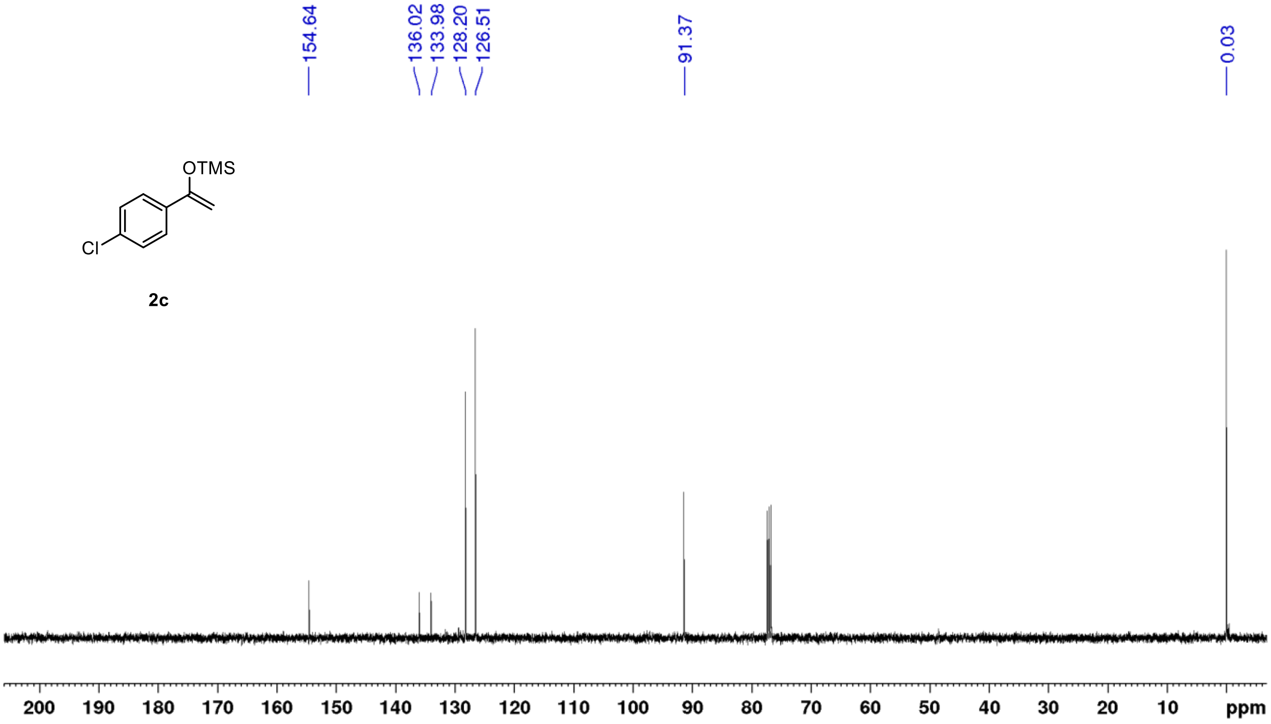

^{1}H NMR (400 MHz, $CDCl_3$, 25 ºC) of (2d)

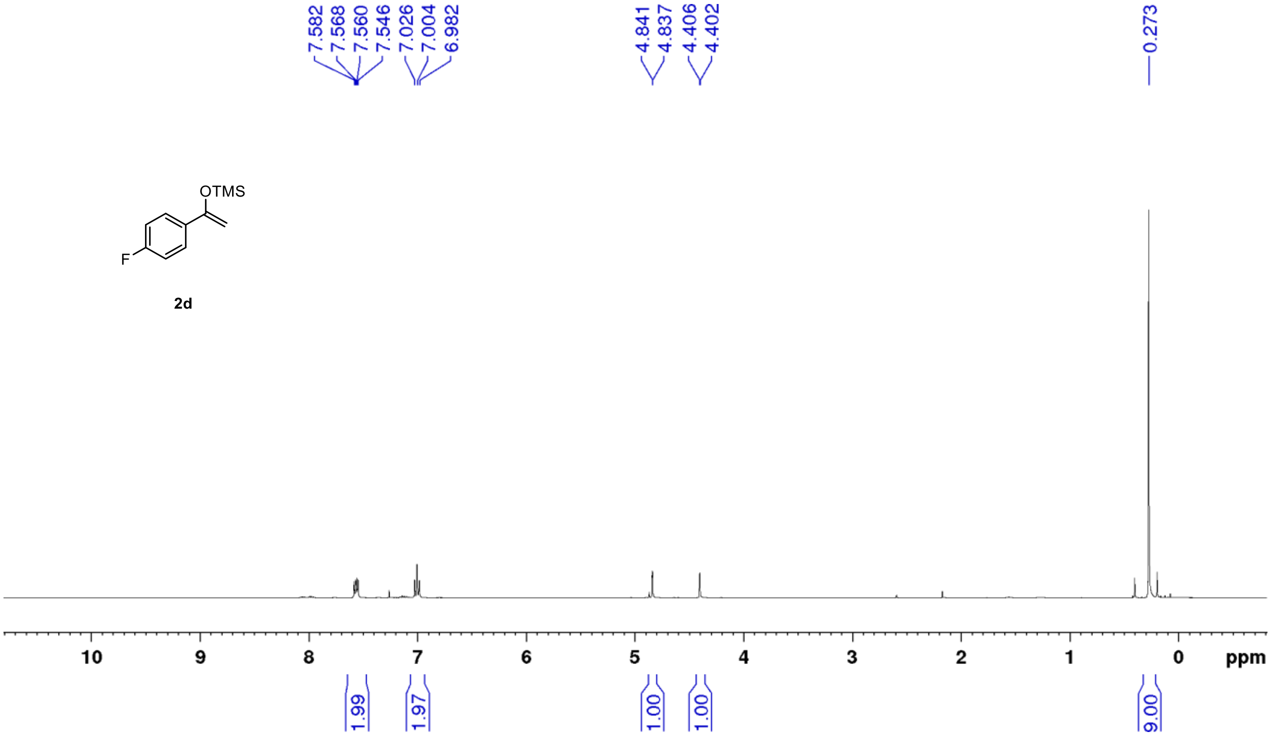

^{13}C NMR (100 MHz, $CDCl_3$, 25 ºC) of (2d)

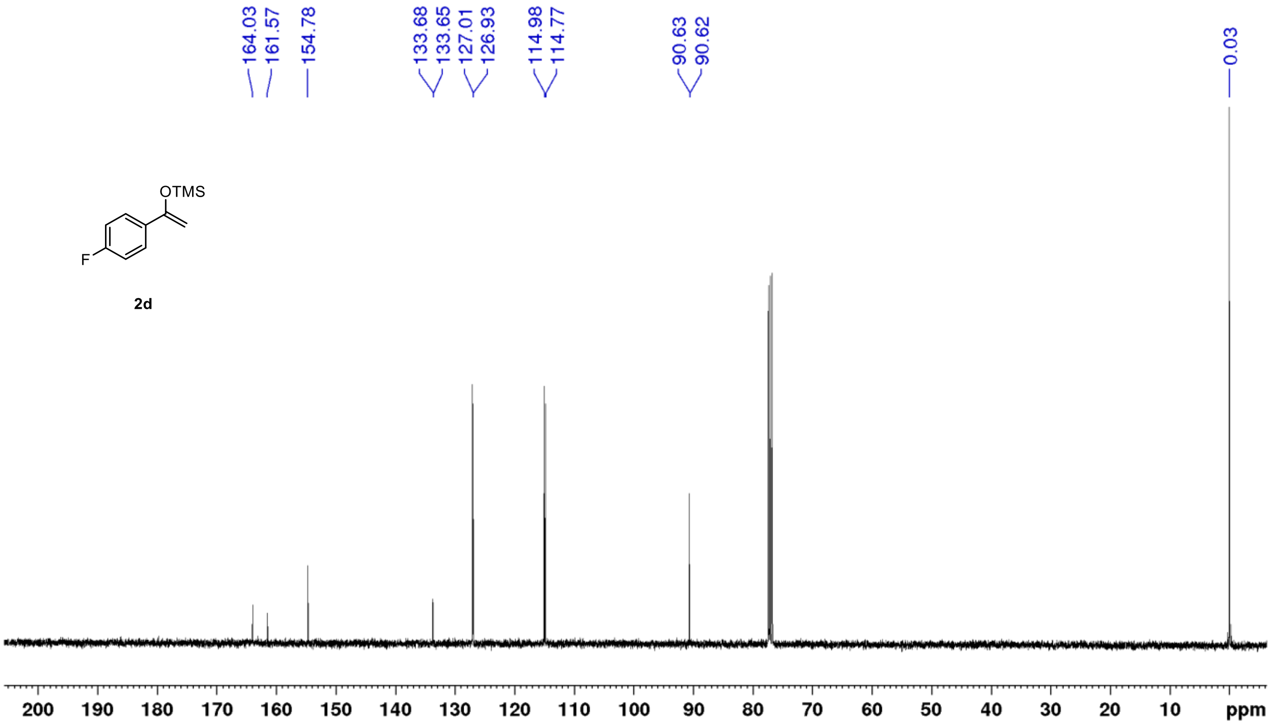

^{19}F NMR (375 MHz, CDCl$_3$, 25 ºC) of (2d)

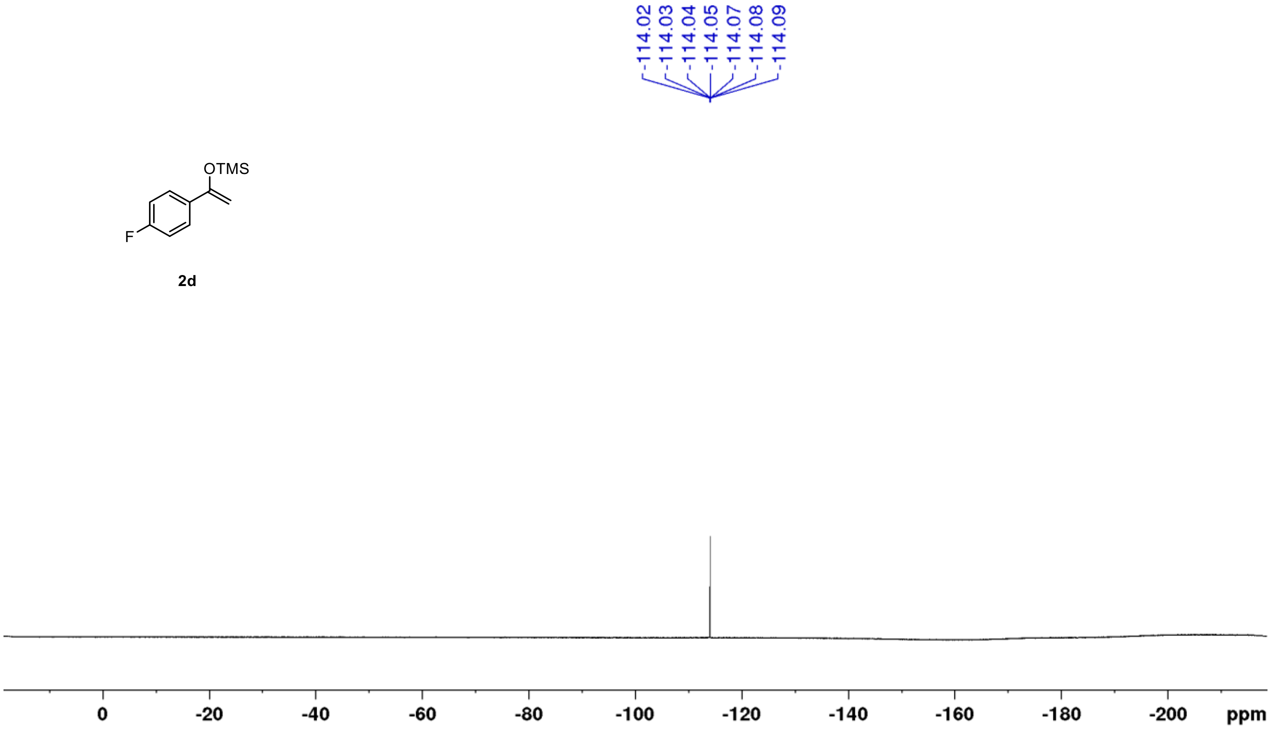

^{1}H NMR (500 MHz, CDCl$_3$, 25 ºC) of (2f)

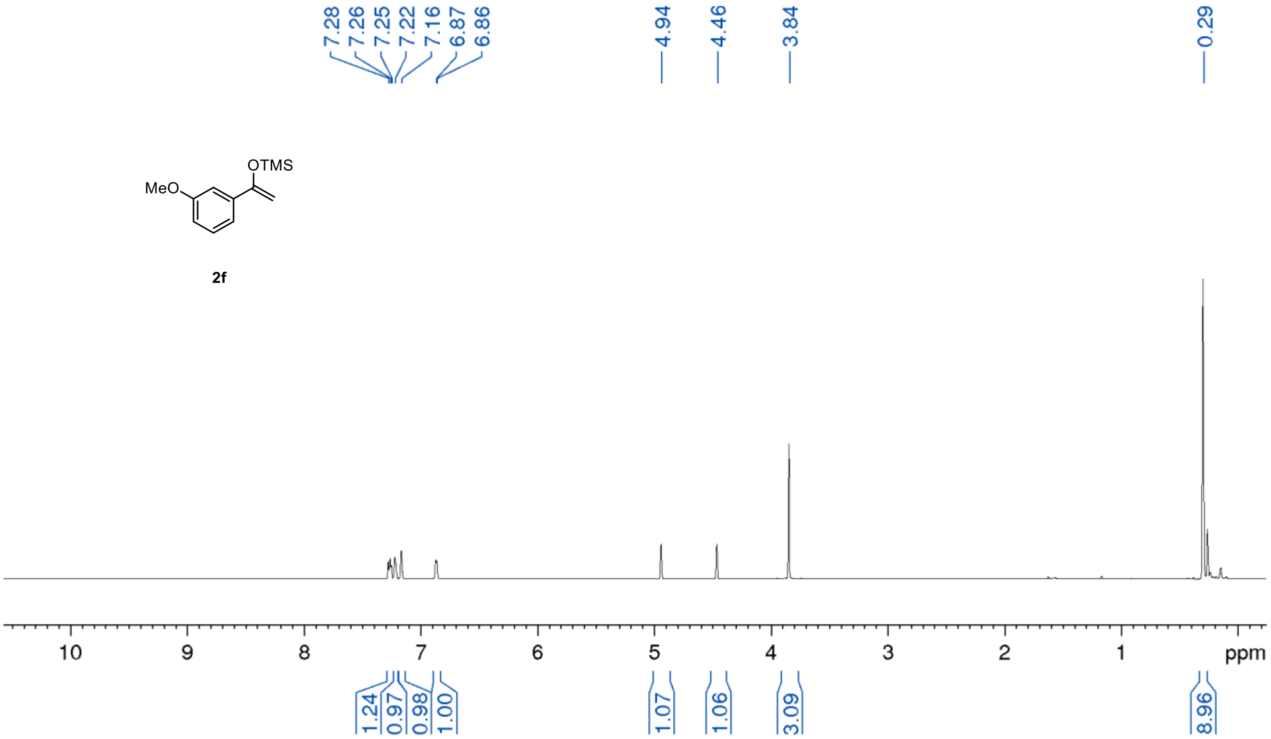

^{13}C NMR (125 MHz, CDCl$_3$, 25 ºC) of (2f)

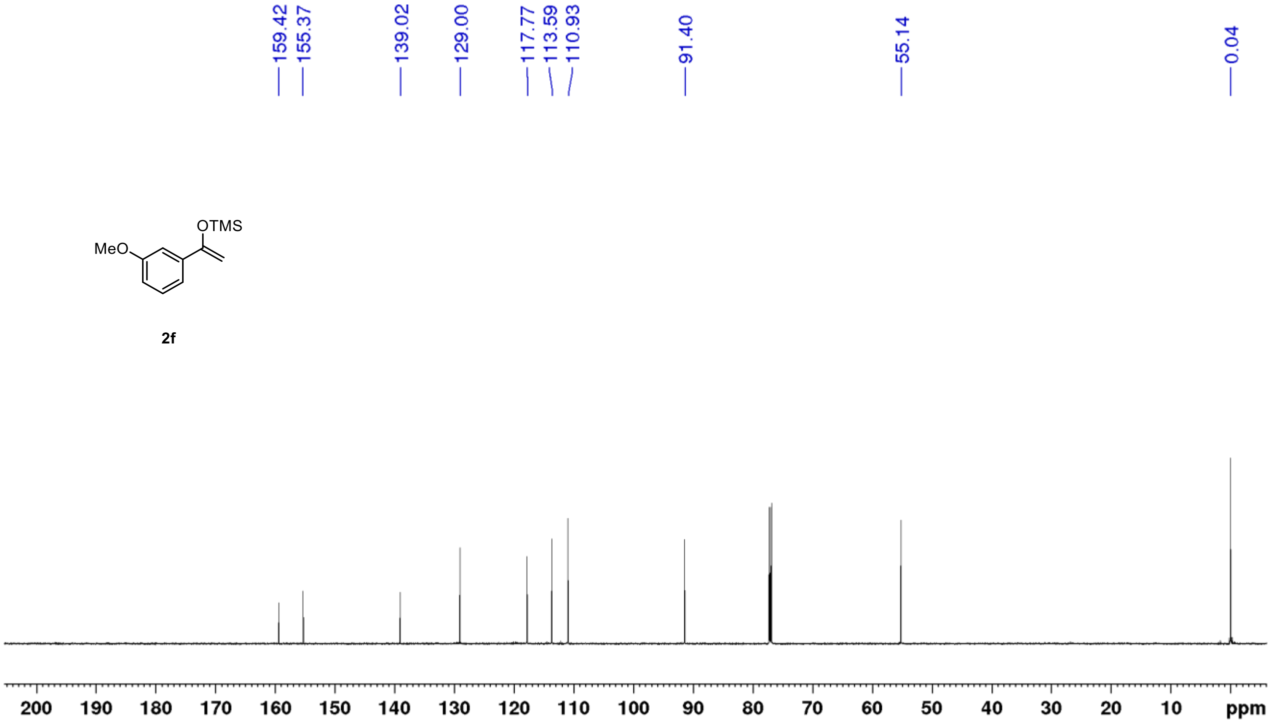

^{1}H NMR (400 MHz, CDCl$_3$, 25 ºC) of (2g)

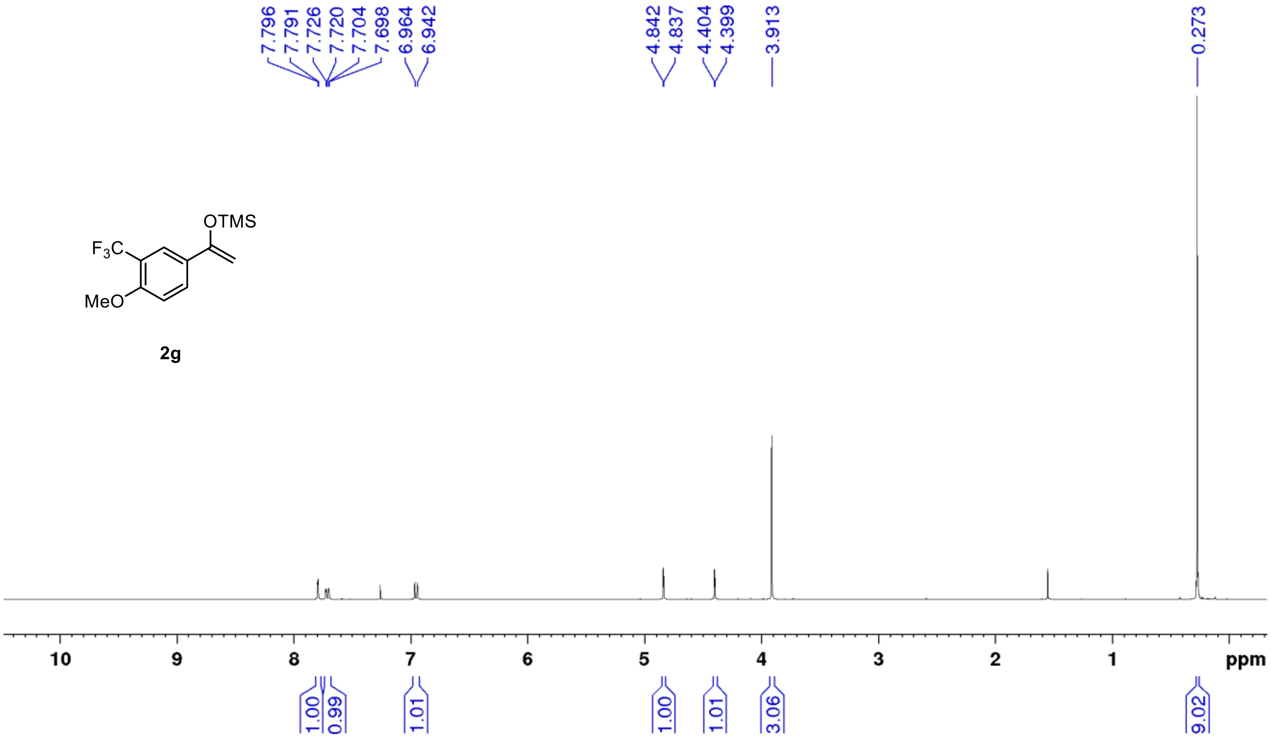

^{13}C NMR (125 MHz, $CDCl_3$, 25 ºC) of (2g)

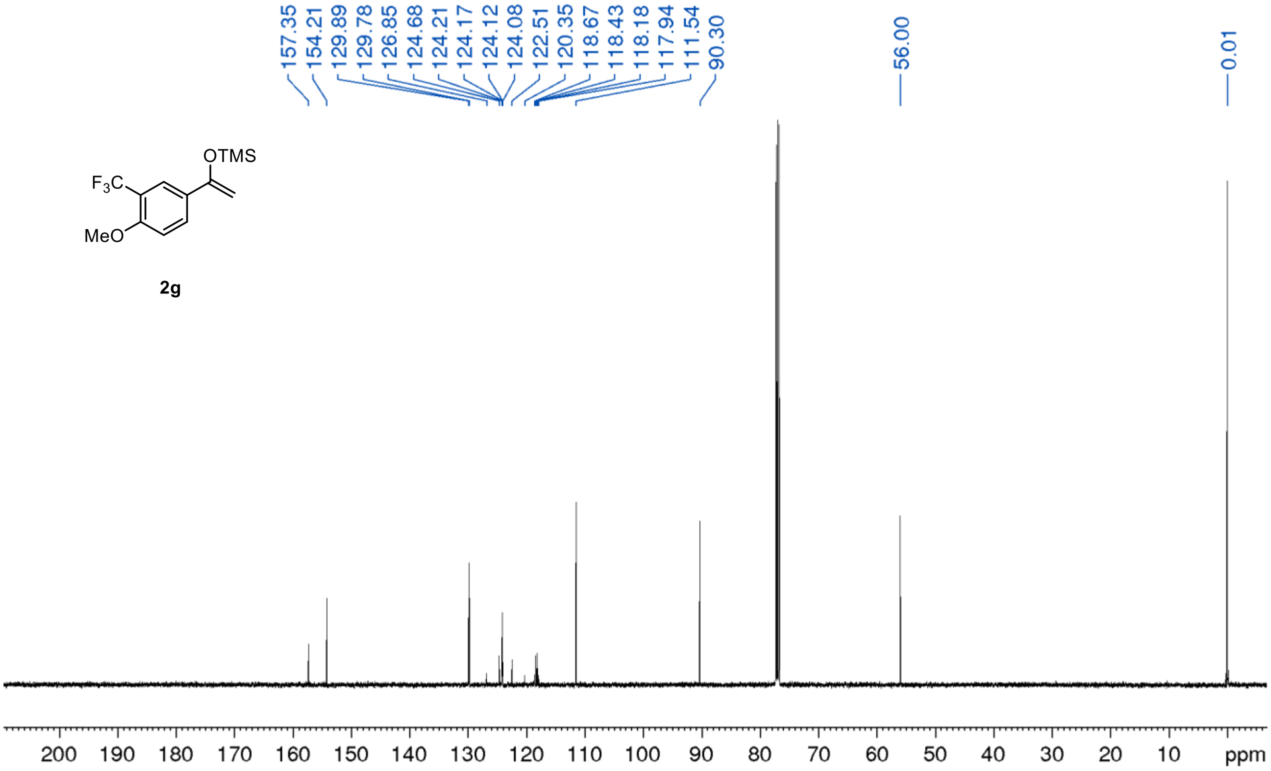

^{19}F NMR (375 MHz, $CDCl_3$, 25 ºC) of (2g)

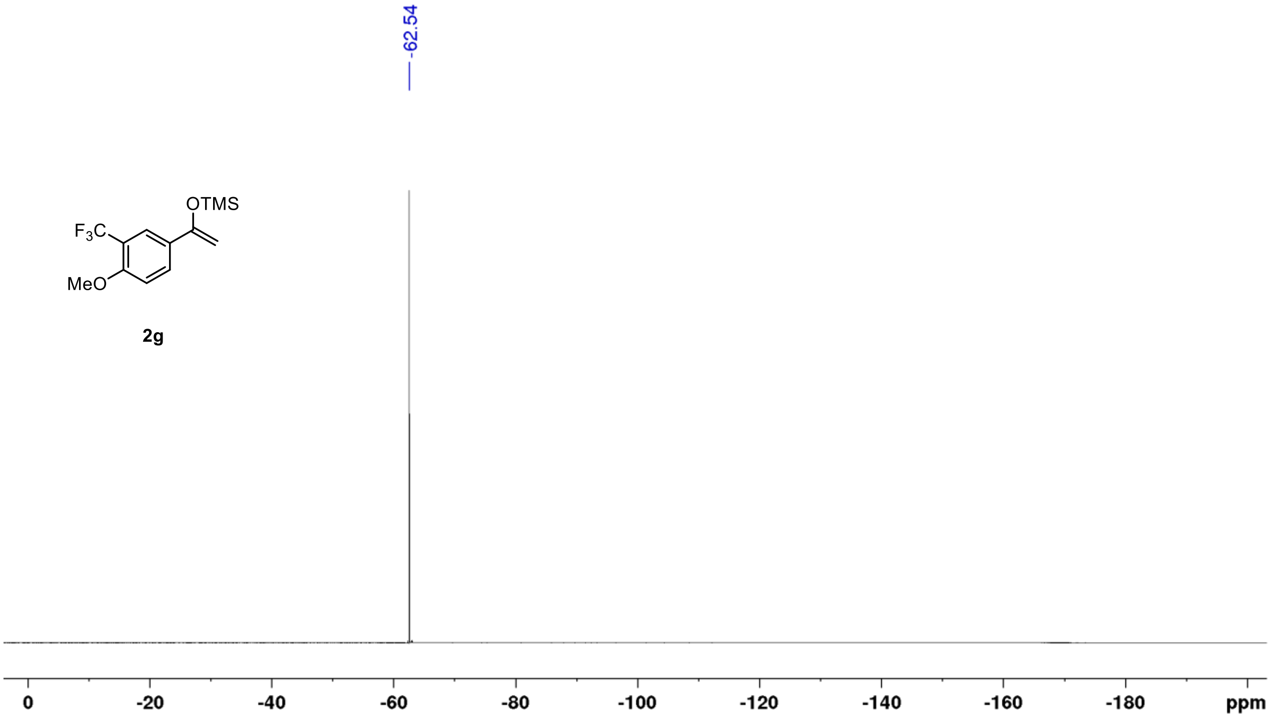

^{1}H NMR (400 MHz, $CDCl_3$, 25 °C) of (2h)

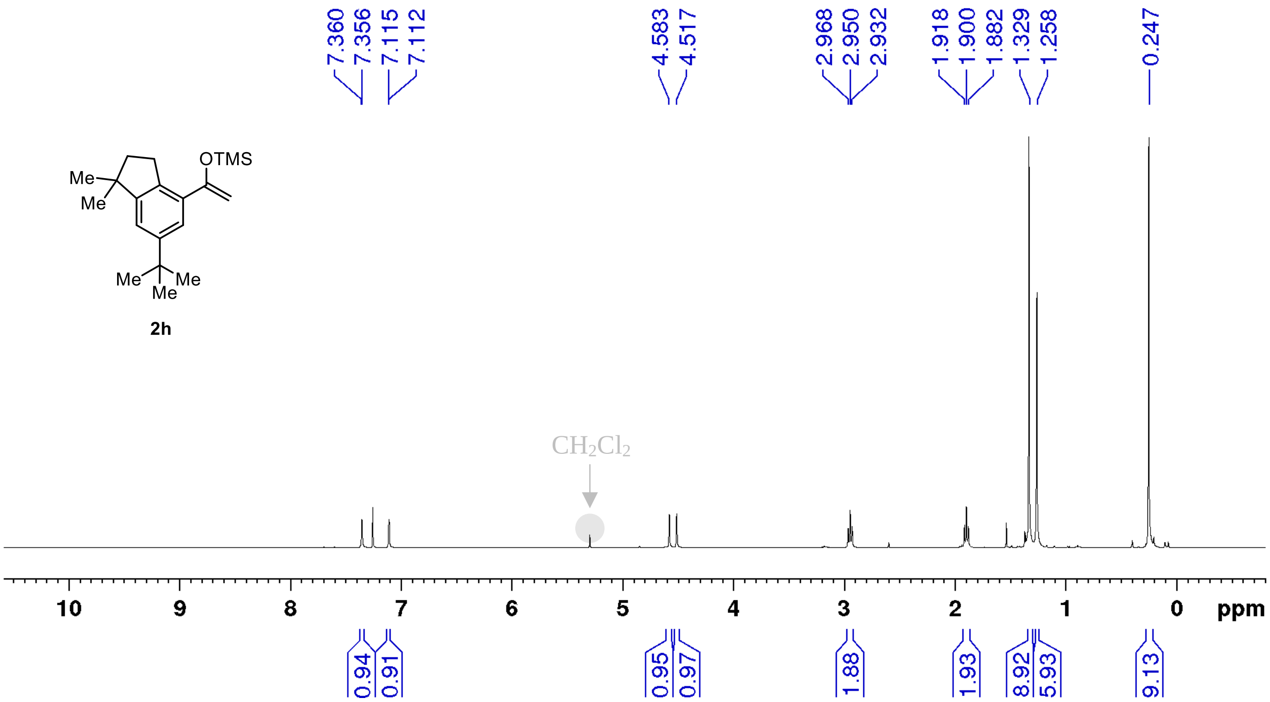

^{13}C NMR (100 MHz, $CDCl_3$, 25 °C) of (2h)

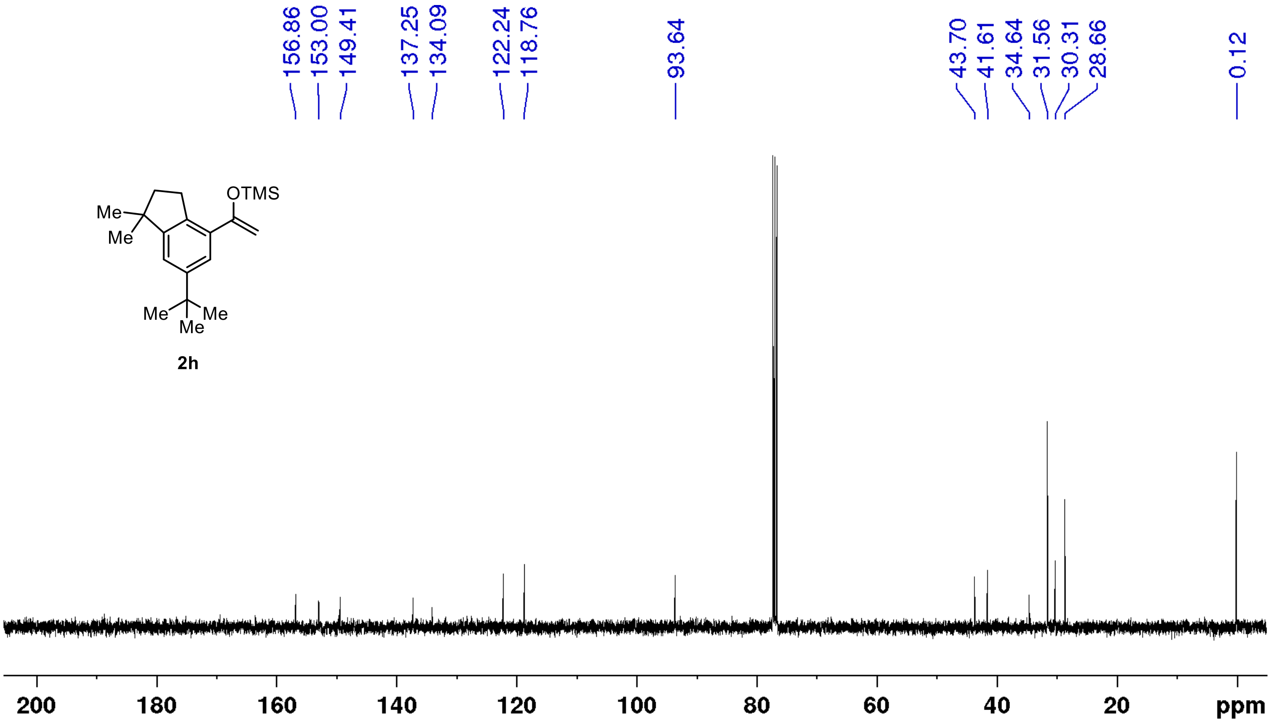

^{1}H NMR (400 MHz, $CDCl_3$, 25 ºC) of (2j)

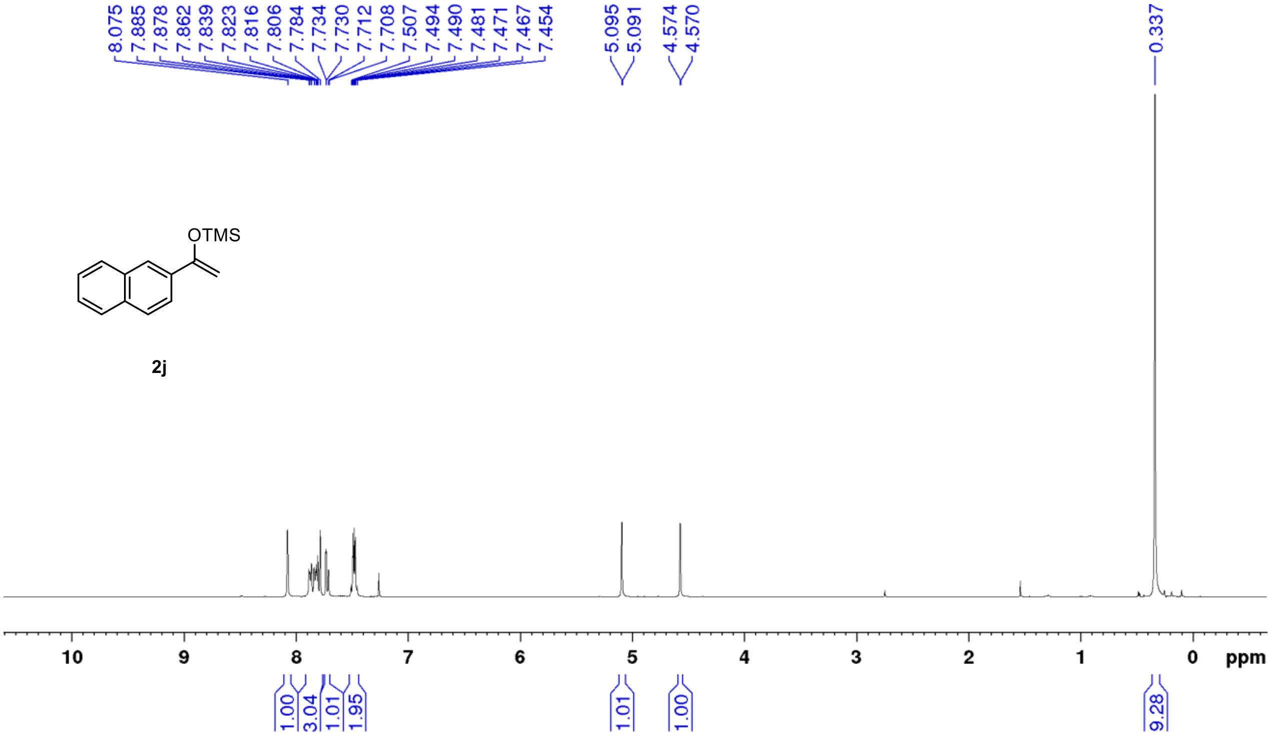

9

^{13}C NMR (100 MHz, $CDCl_3$, 25 ºC) of (2j)

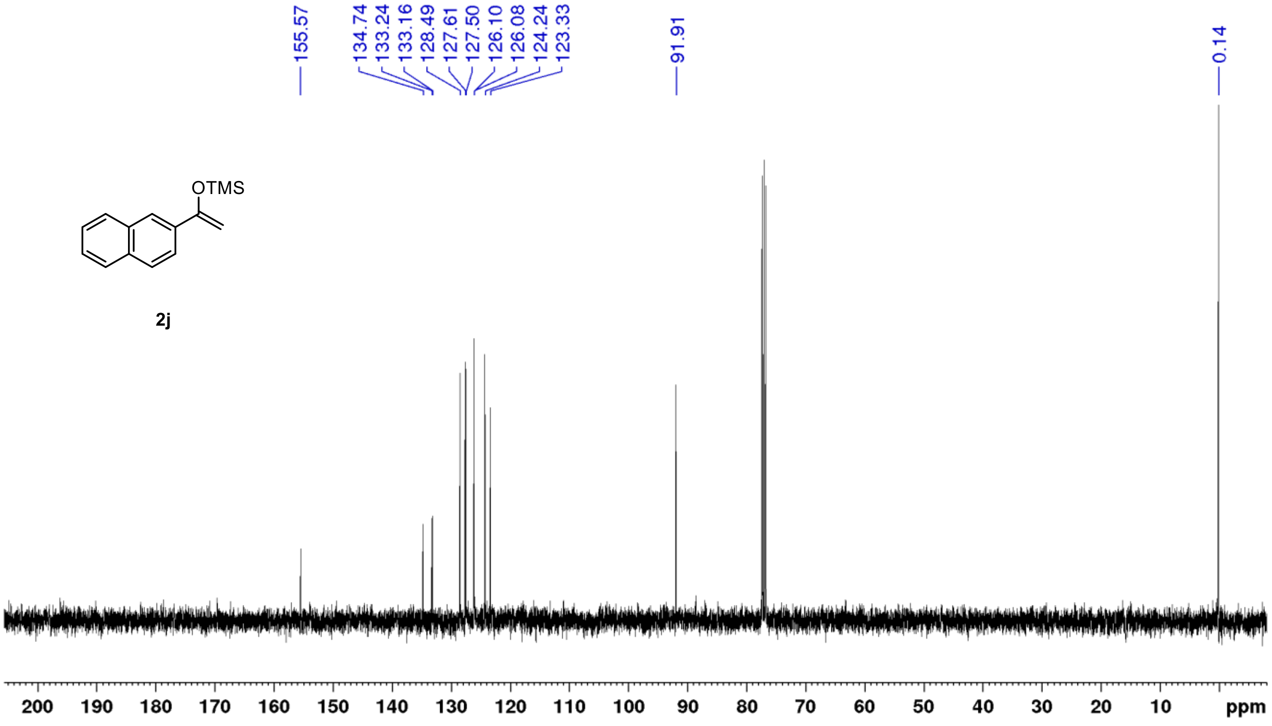

^{1}H NMR (400 MHz, $CDCl_3$, 25 °C) of (2k)

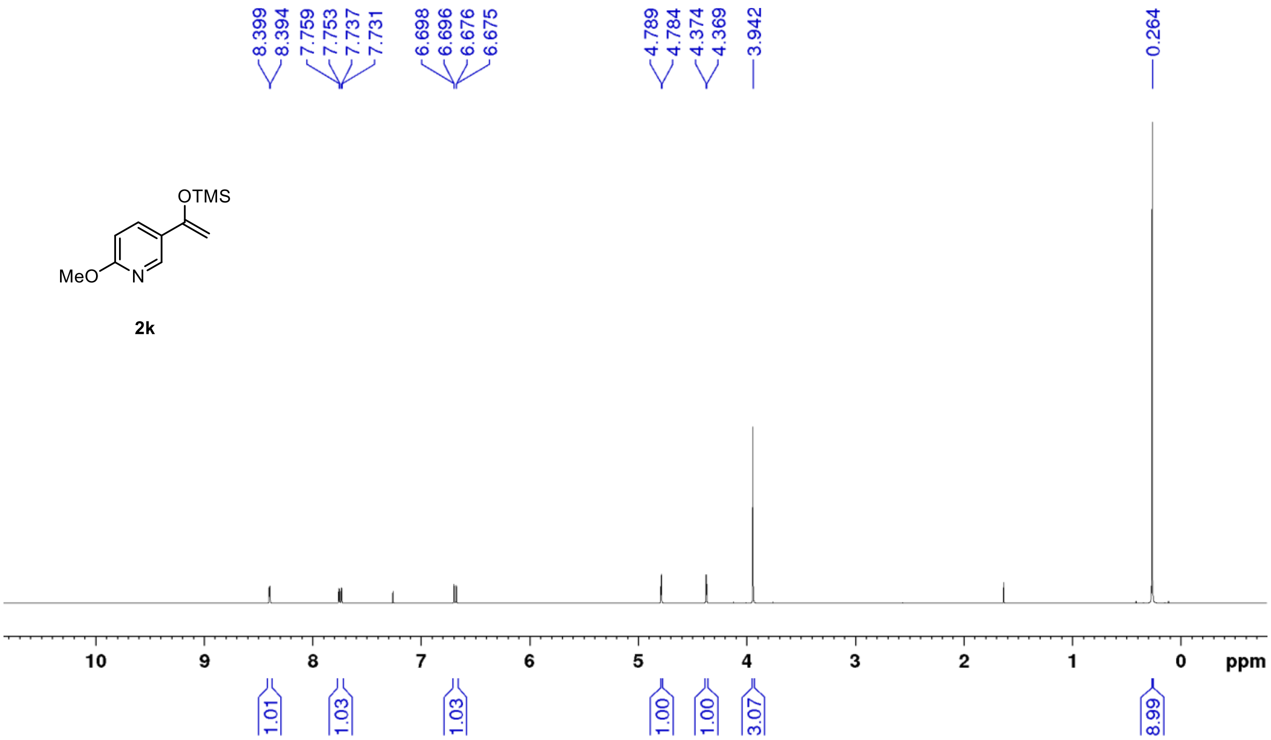

^{13}C NMR (100 MHz, $CDCl_3$, 25 °C) of (2k)

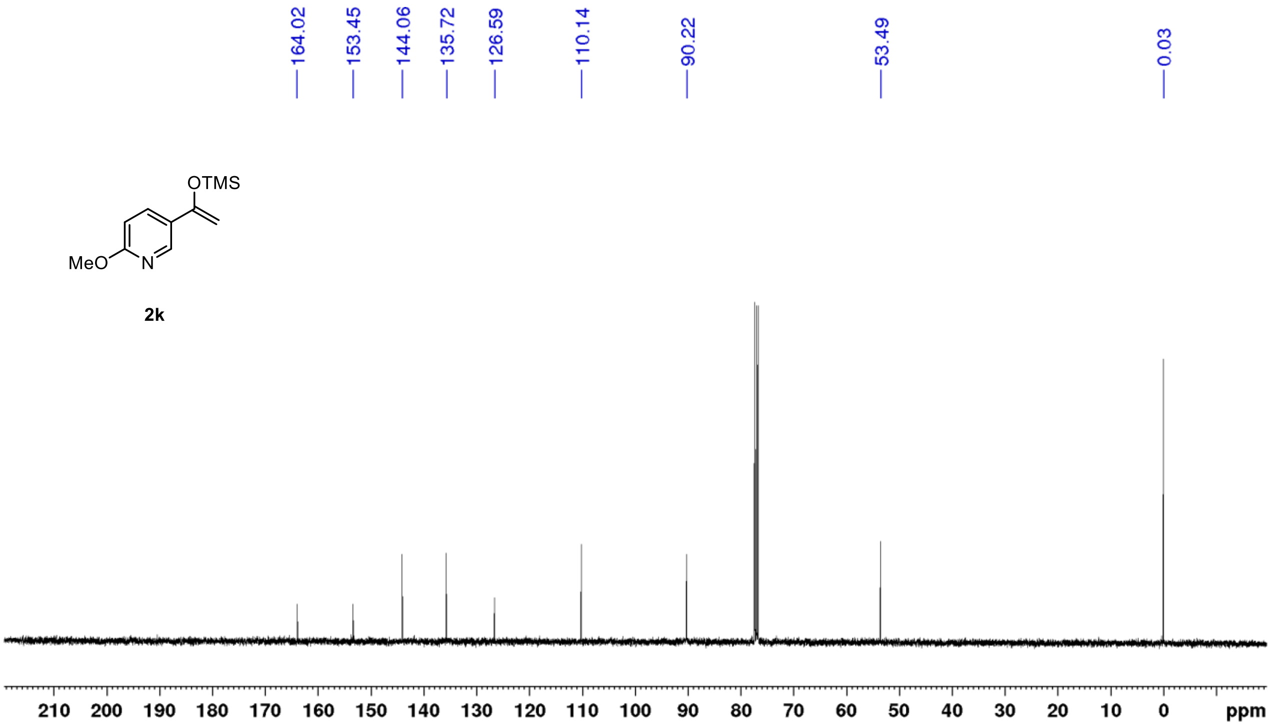

^{1}H NMR (400 MHz, $CDCl_3$, 25 °C) of (2l)

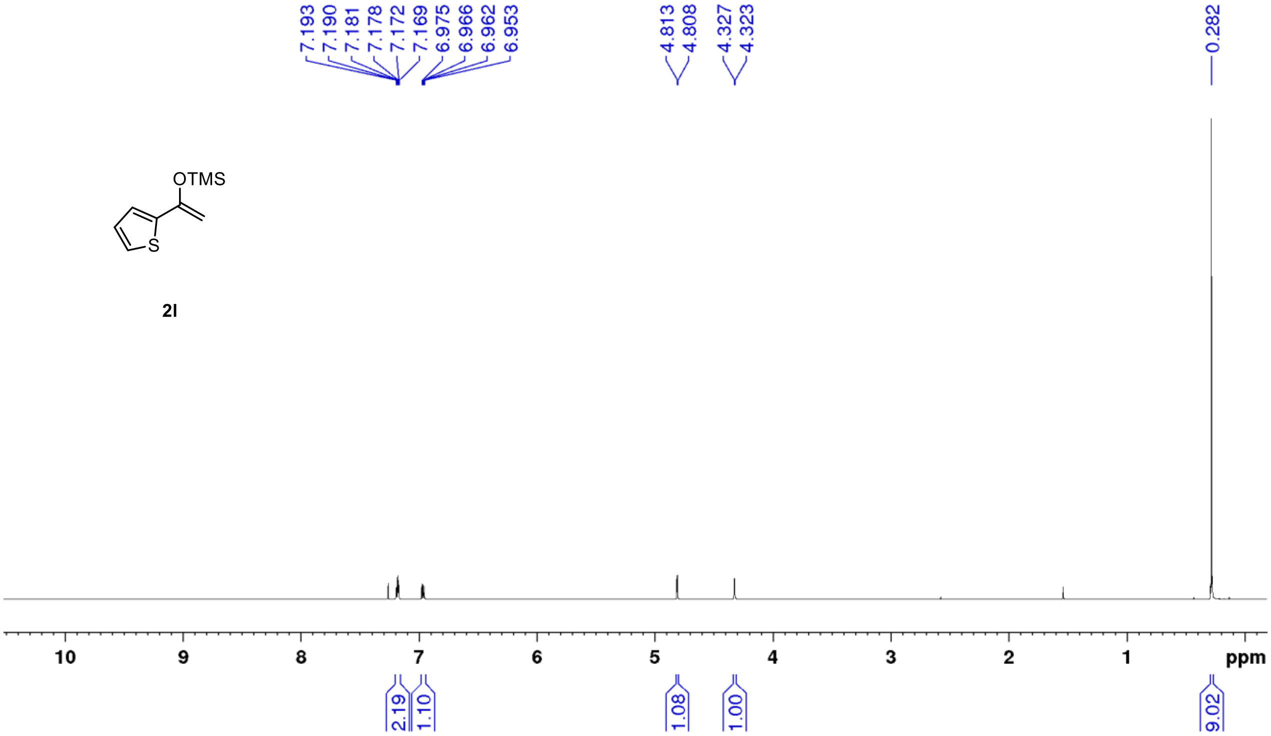

^{13}C NMR (100 MHz, $CDCl_3$, 25 °C) of (2l)

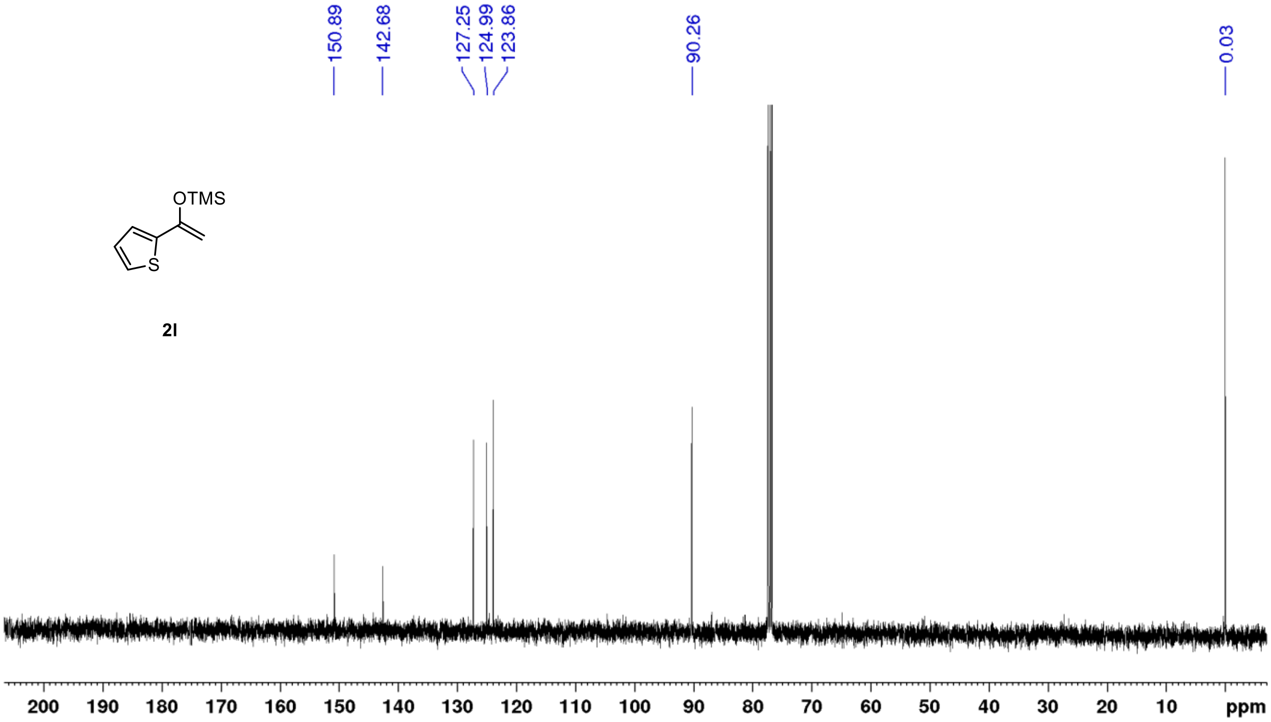

^{1}H NMR (500 MHz, $CDCl_3$, 25 °C) of (2n)

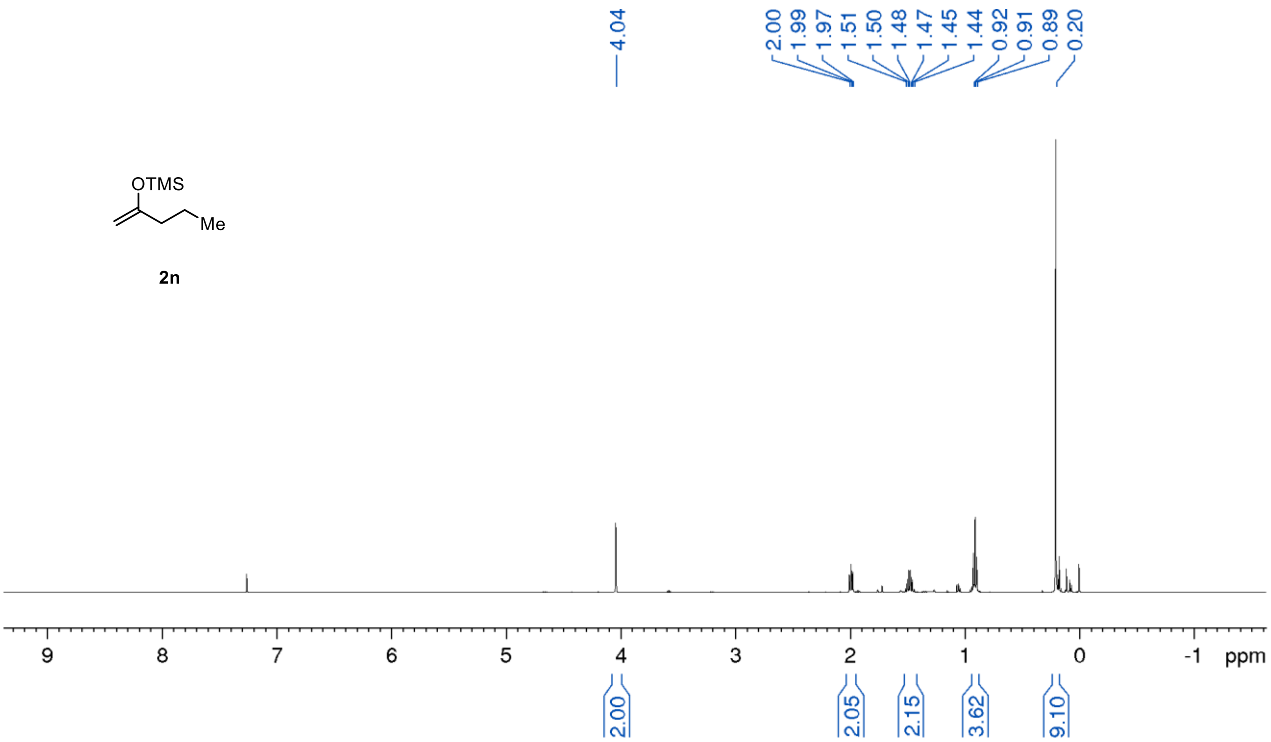

^{13}C NMR (125 MHz, $CDCl_3$, 25 °C) of (2n)

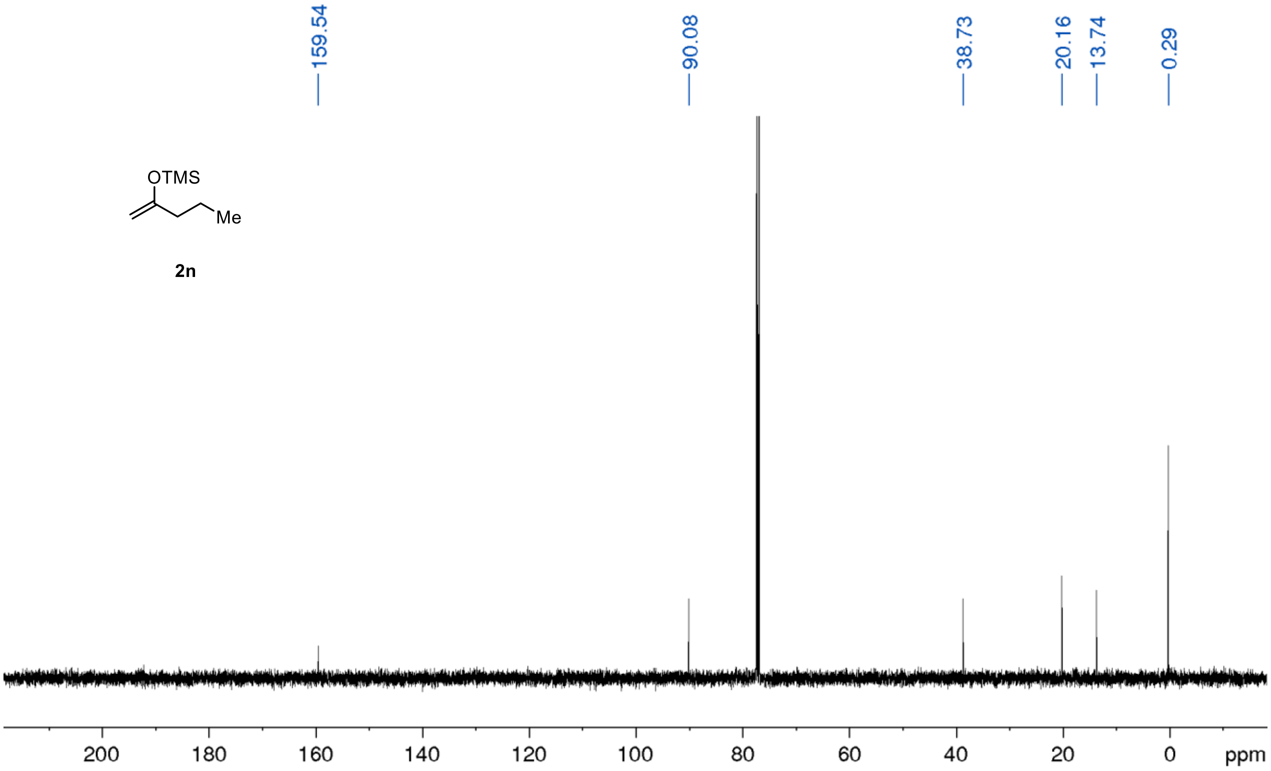

^{1}H NMR (500 MHz, CDCl$_3$, 25 °C) of (2o)

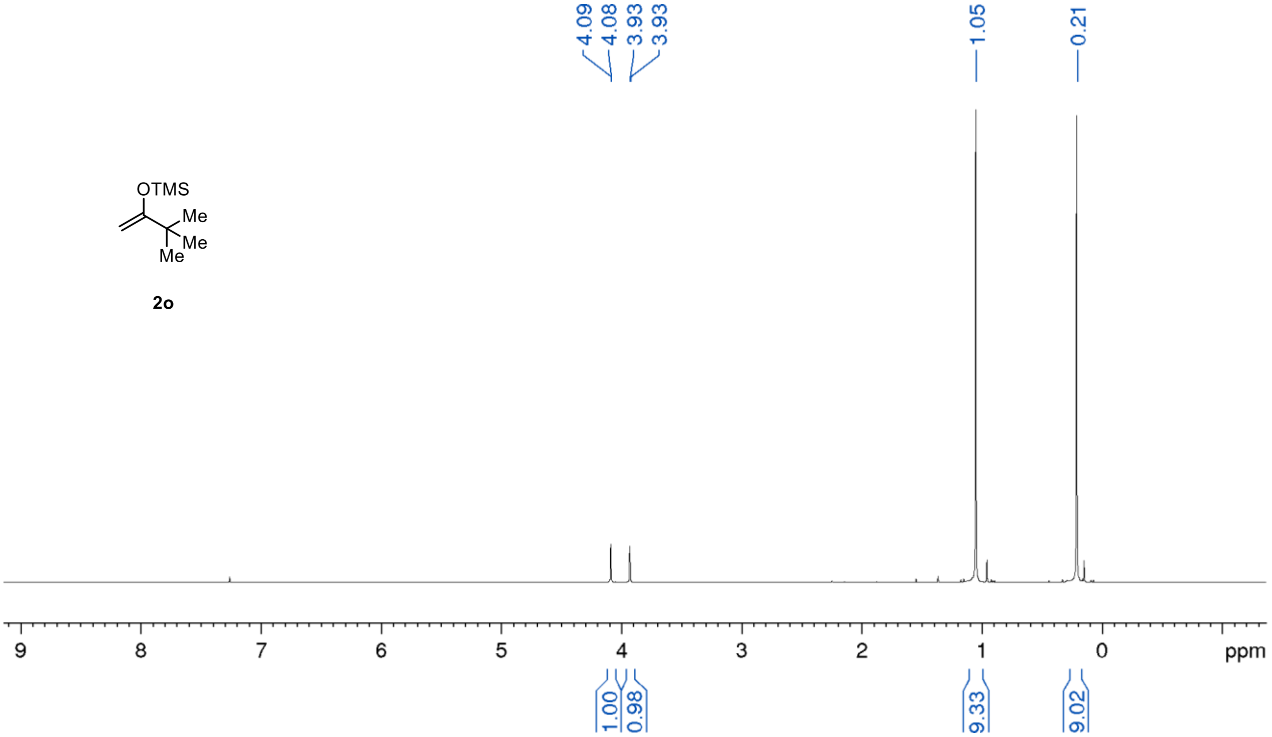

^{13}C NMR (125 MHz, CDCl$_3$, 25 °C) of (2o)

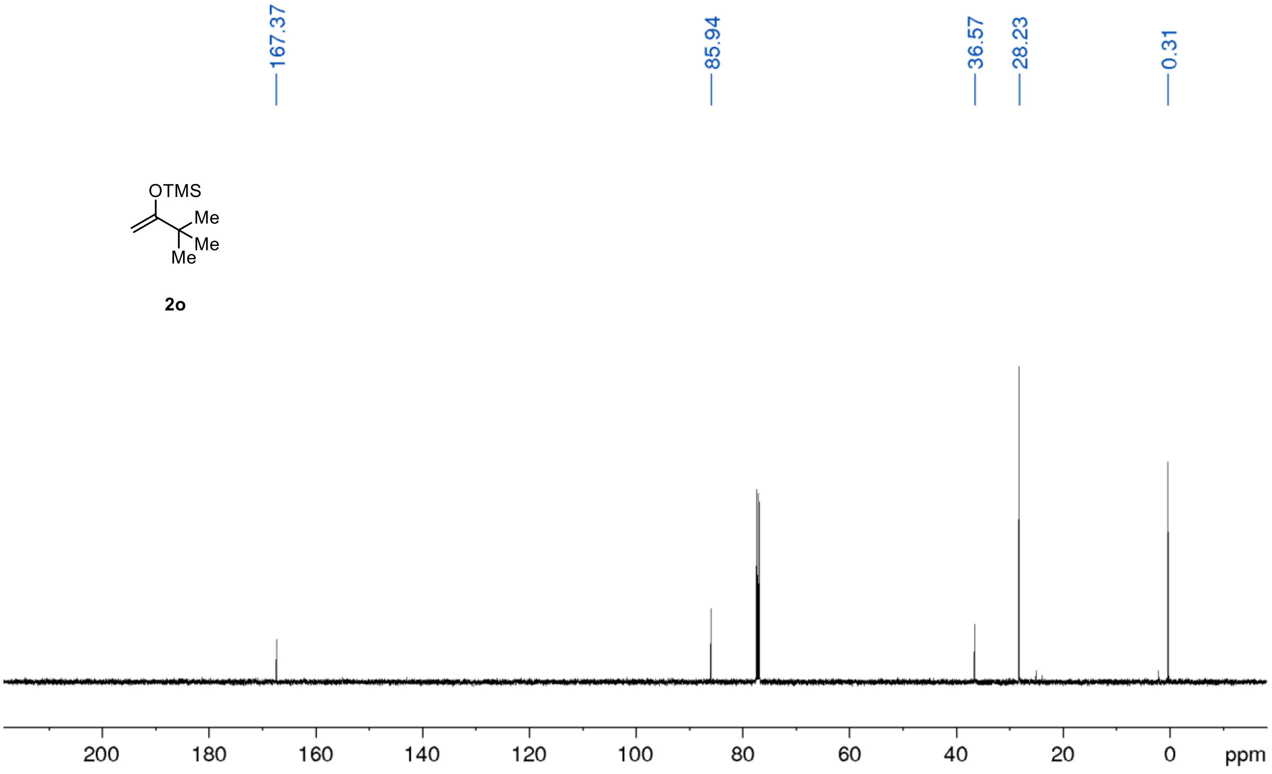

^{1}H NMR (400 MHz, $CDCl_3$, 25 °C) of (2q)

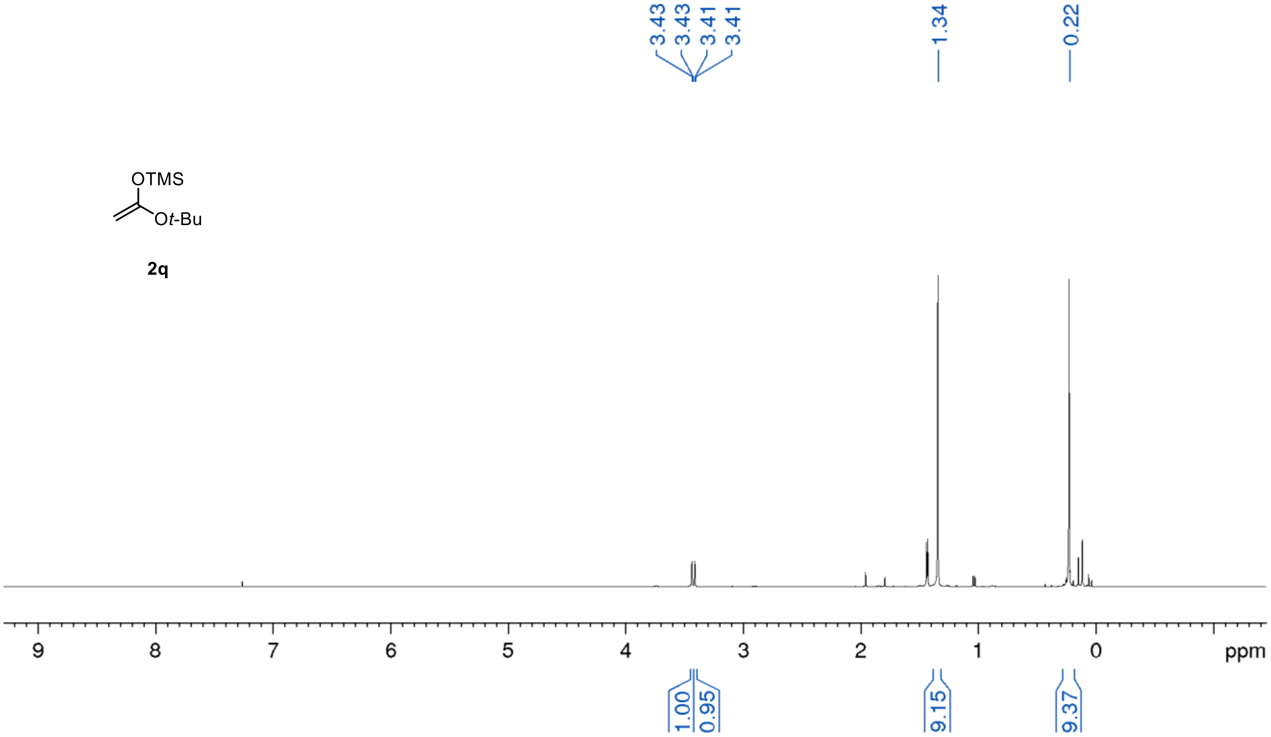

^{13}C NMR (100 MHz, $CDCl_3$, 25 °C) of (2q)

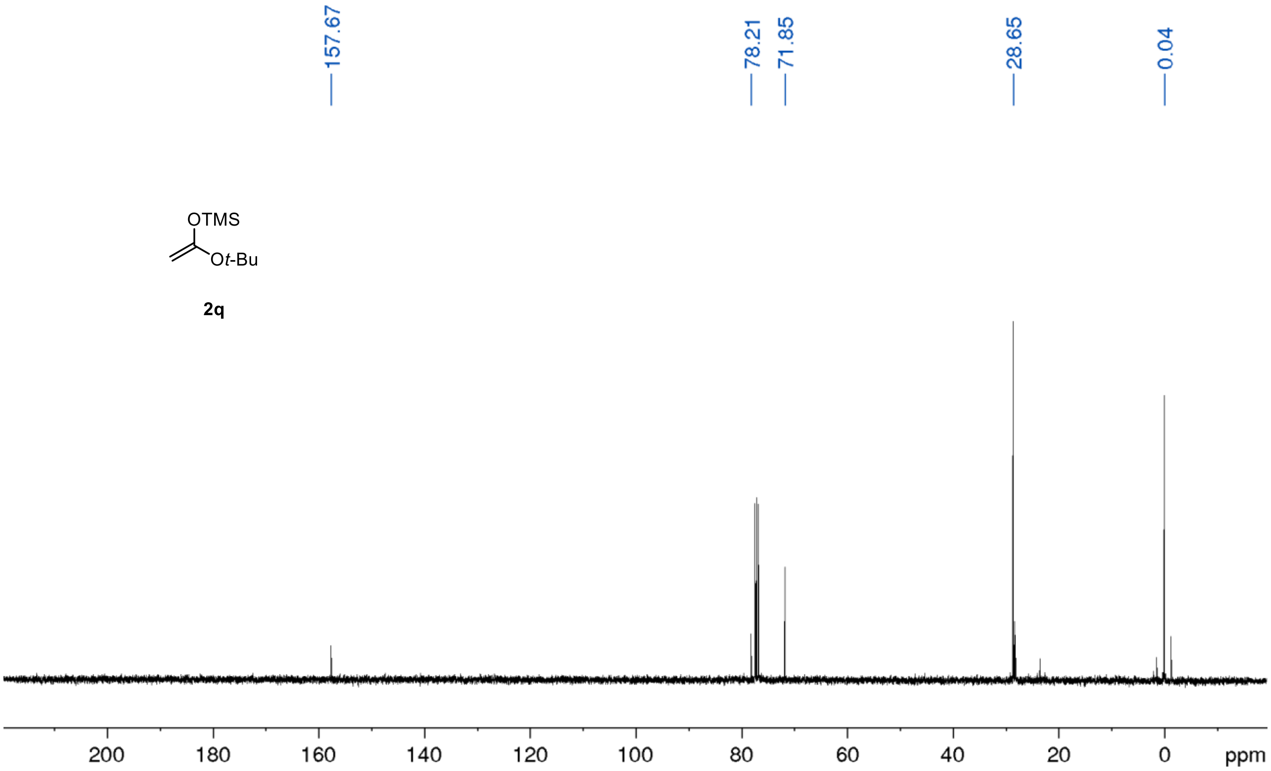

^{1}H NMR (400 MHz, $CDCl_3$, 25 ºC) of (2s)

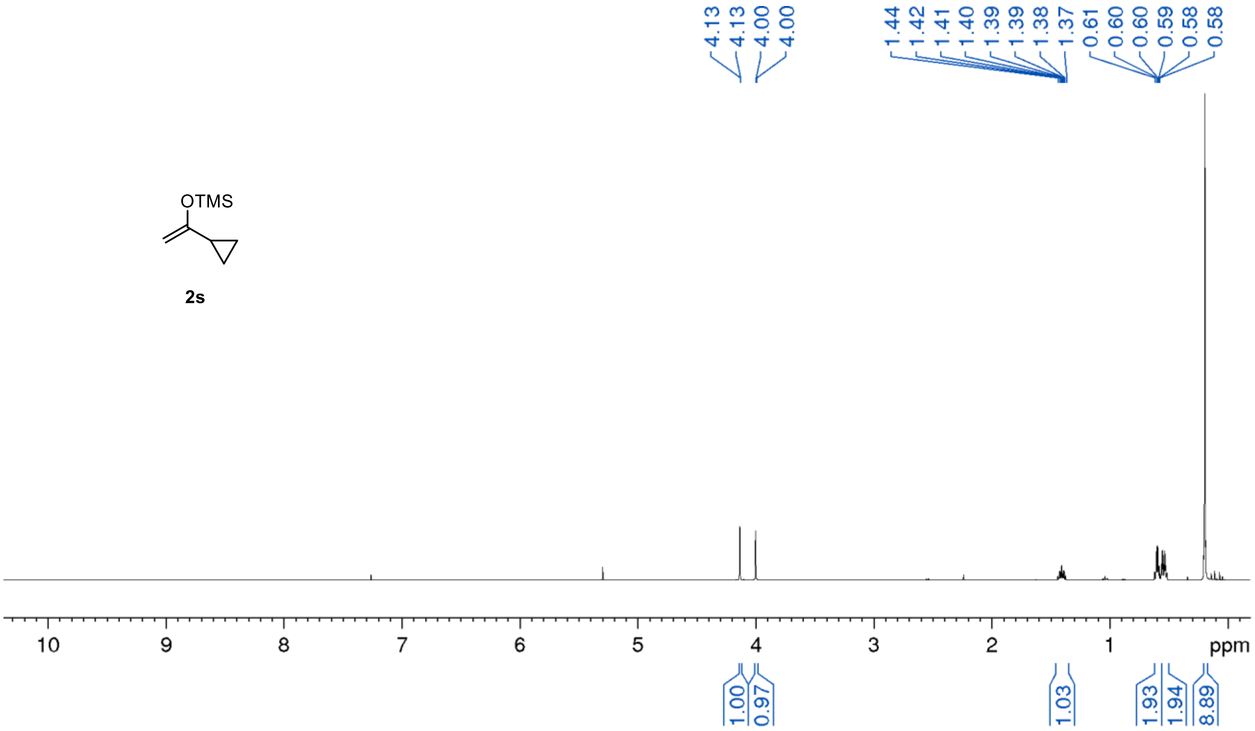

^{13}C NMR (100 MHz, $CDCl_3$, 25 ºC) of (2s)

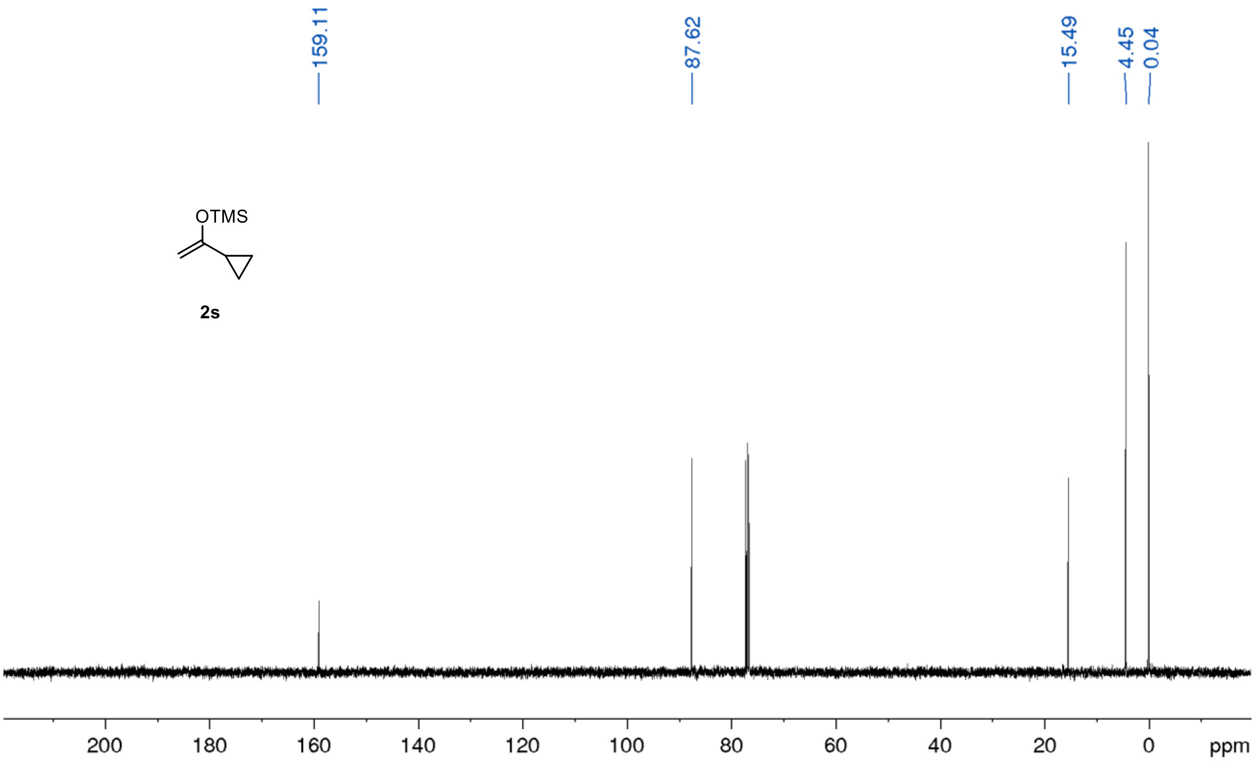

^{1}H NMR (500 MHz, $CDCl_3$, 25 ºC) of (1a)

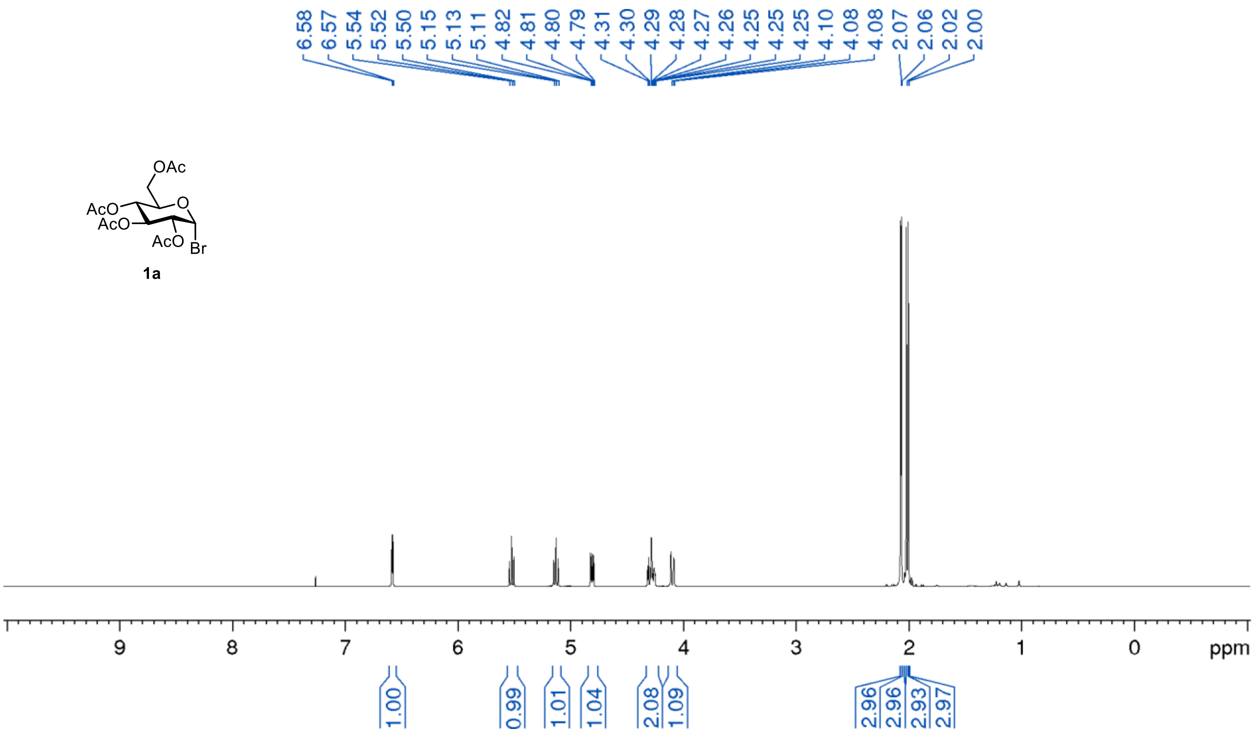

^{13}C NMR (125 MHz, $CDCl_3$, 25 ºC) of (1a)

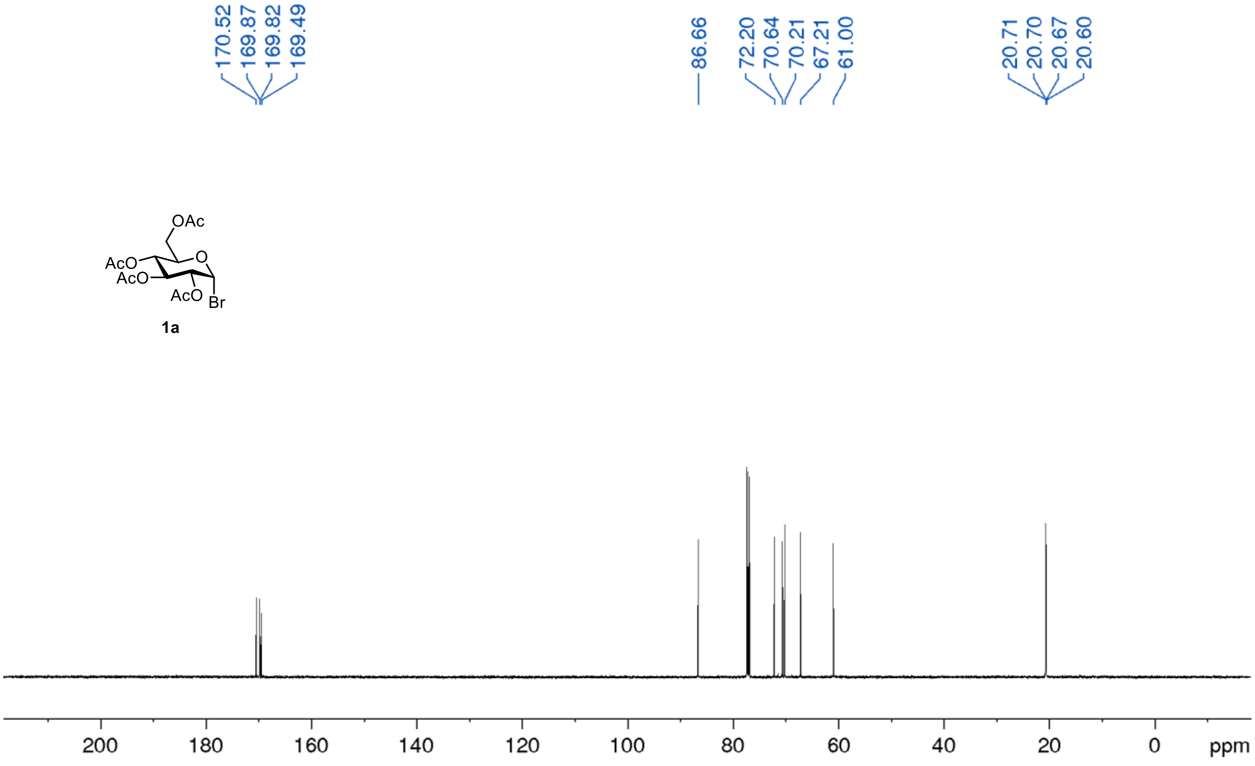

^{1}H NMR (500 MHz, $CDCl_3$, 25 ºC) of (1b)

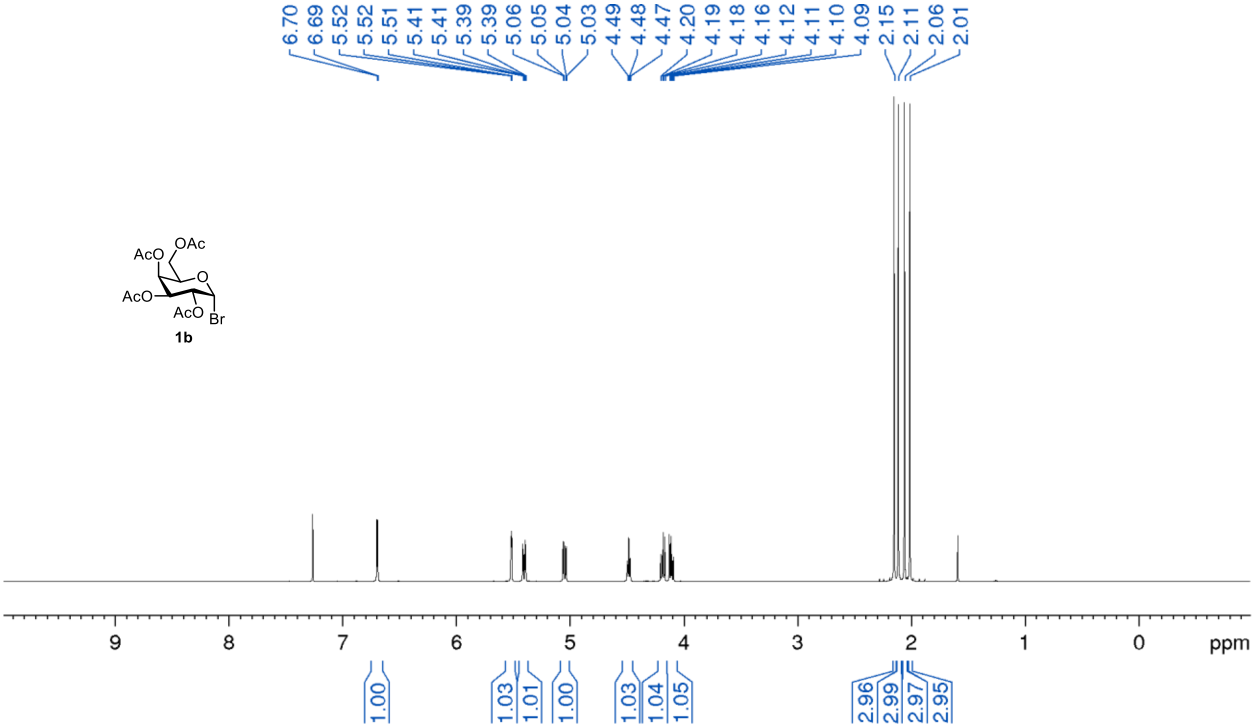

^{13}C NMR (125 MHz, $CDCl_3$, 25 ºC) of (1b)

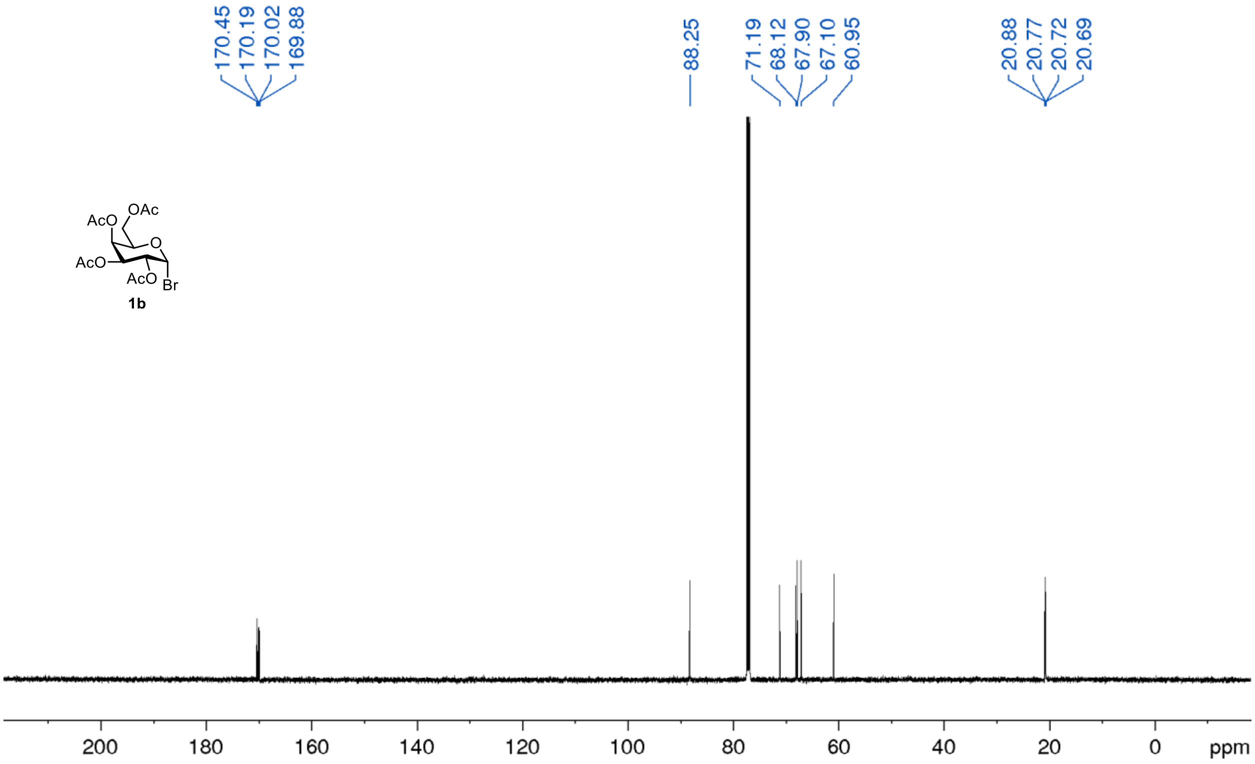

^{1}H NMR (700 MHz, $CDCl_3$, 25 ºC) of (1c)

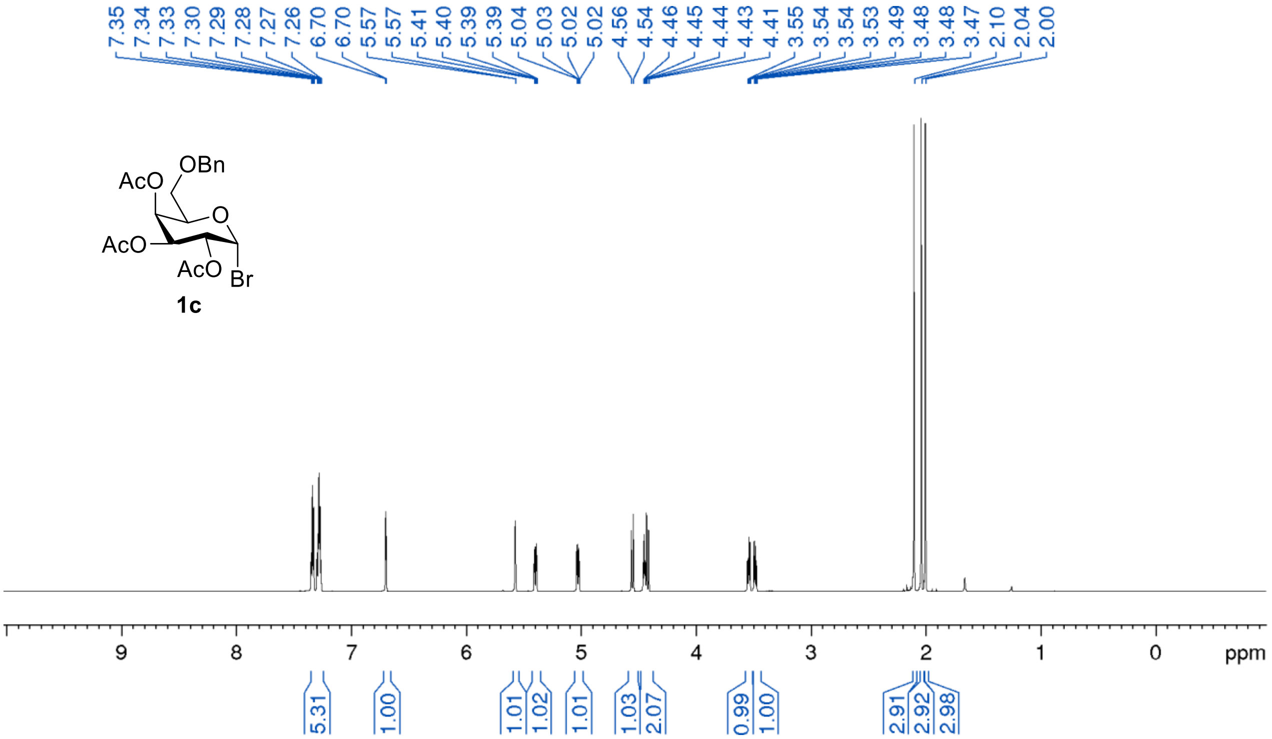

^{13}C NMR (175 MHz, $CDCl_3$, 25 ºC) of (1c)

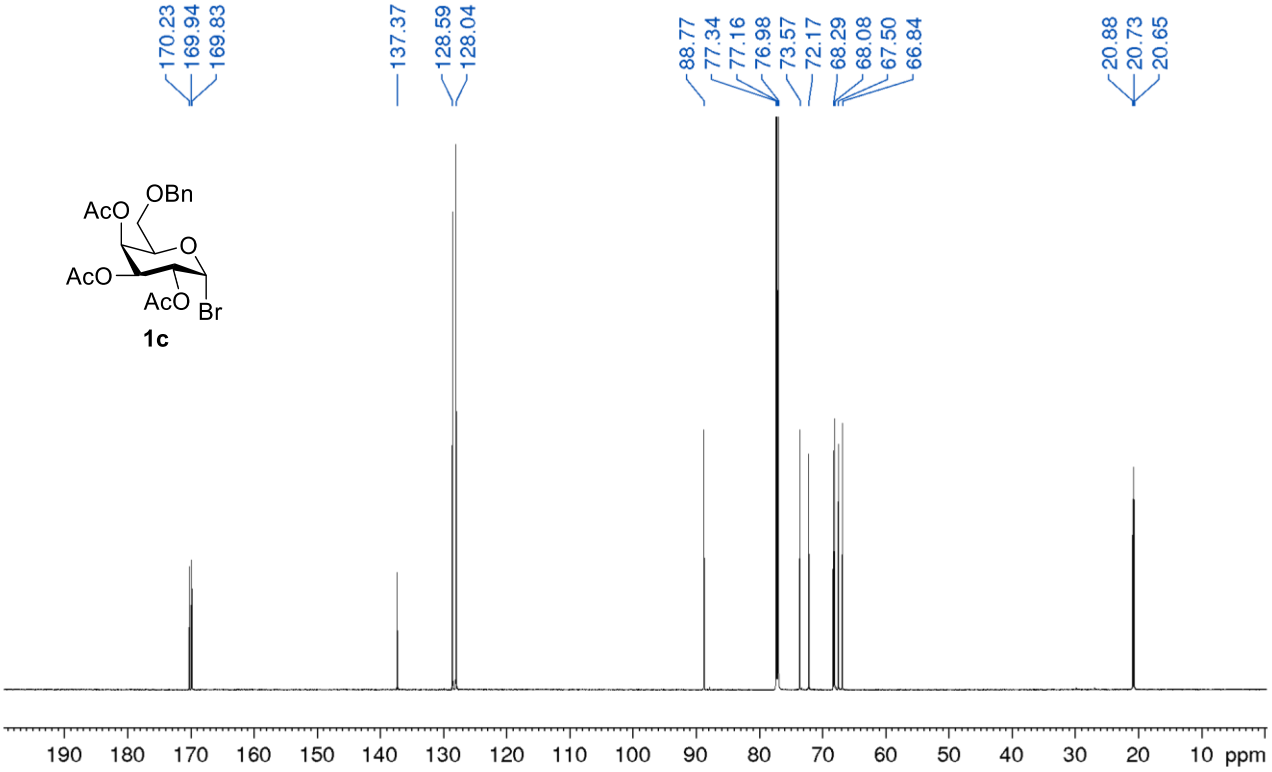

^{1}H NMR (700 MHz, $CDCl_3$, 25 °C) of (1d)

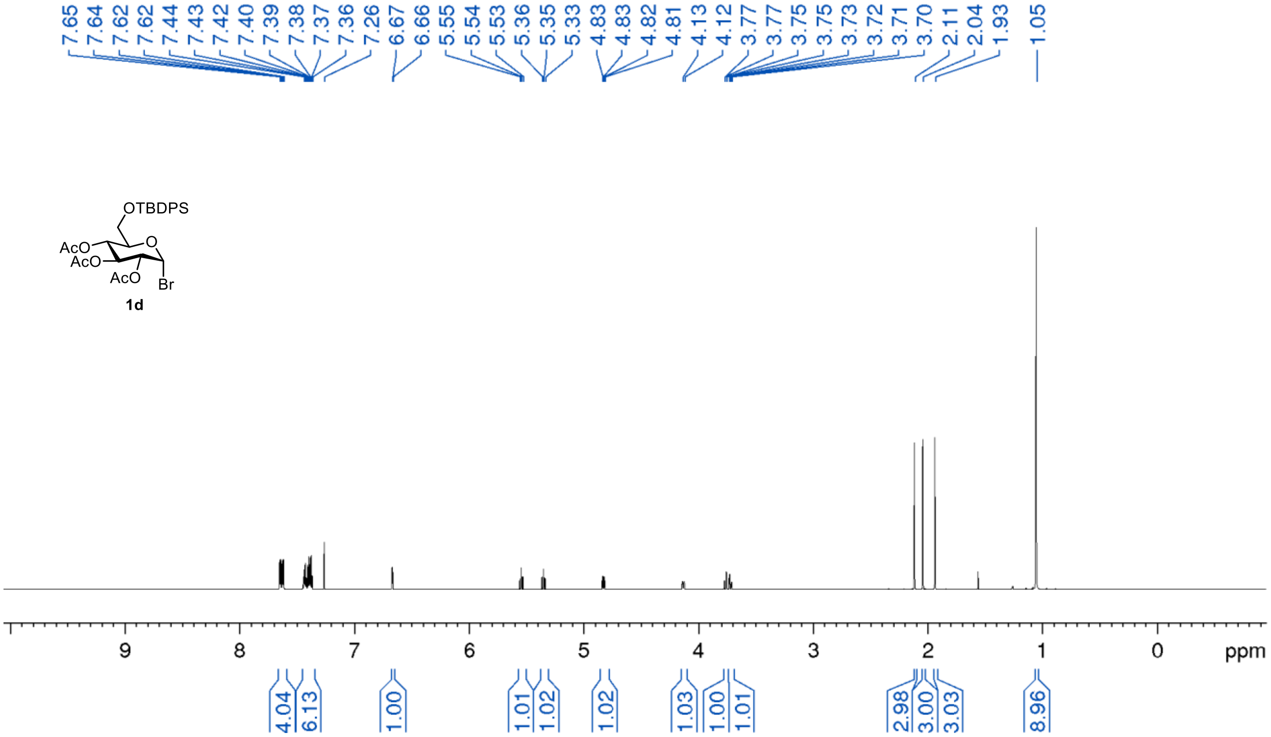

^{13}C NMR (175 MHz, $CDCl_3$, 25 °C) of (1d)

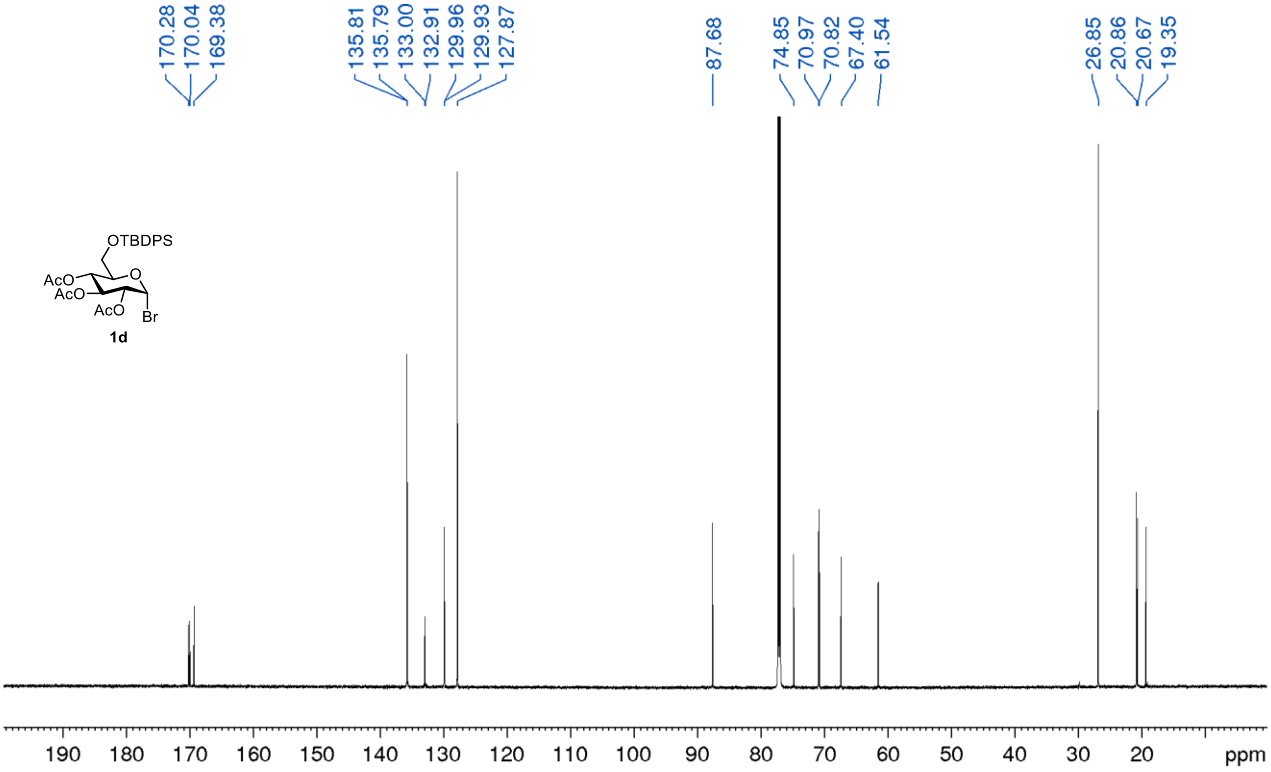

^{1}H NMR (400 MHz, $CDCl_3$, 25 ºC) of (1e)

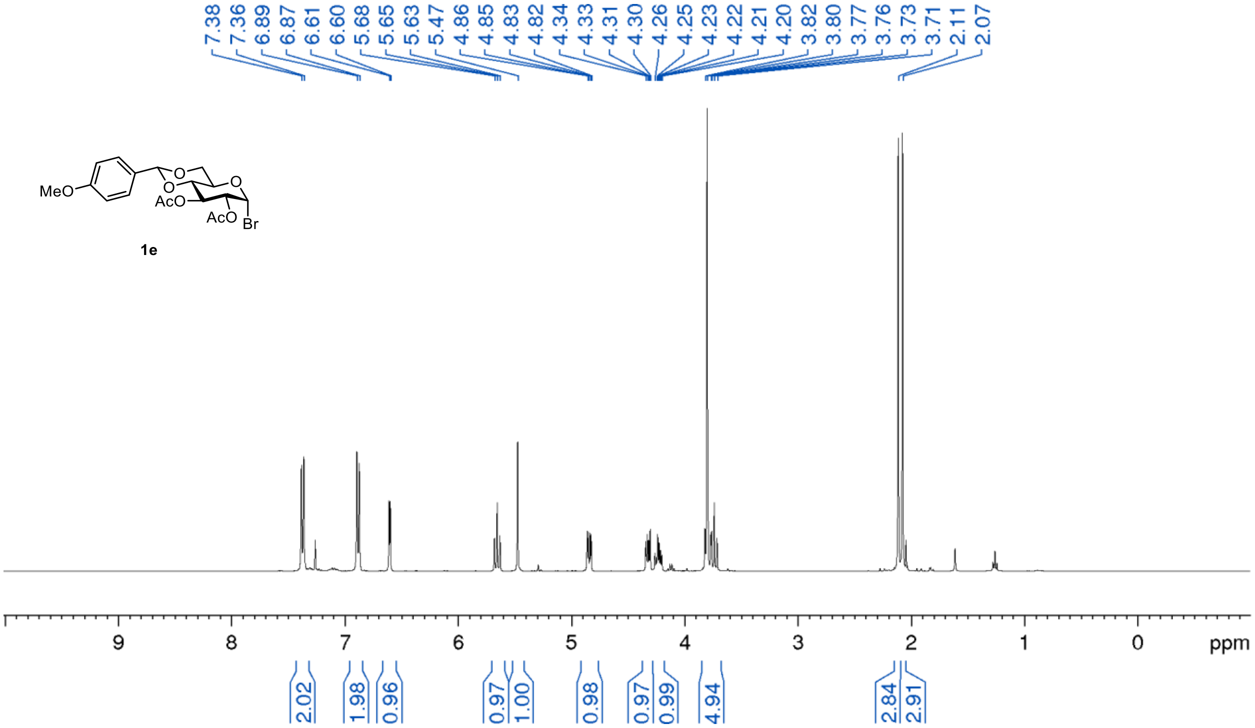

^{13}C NMR (100 MHz, $CDCl_3$, 25 ºC) of (1e)

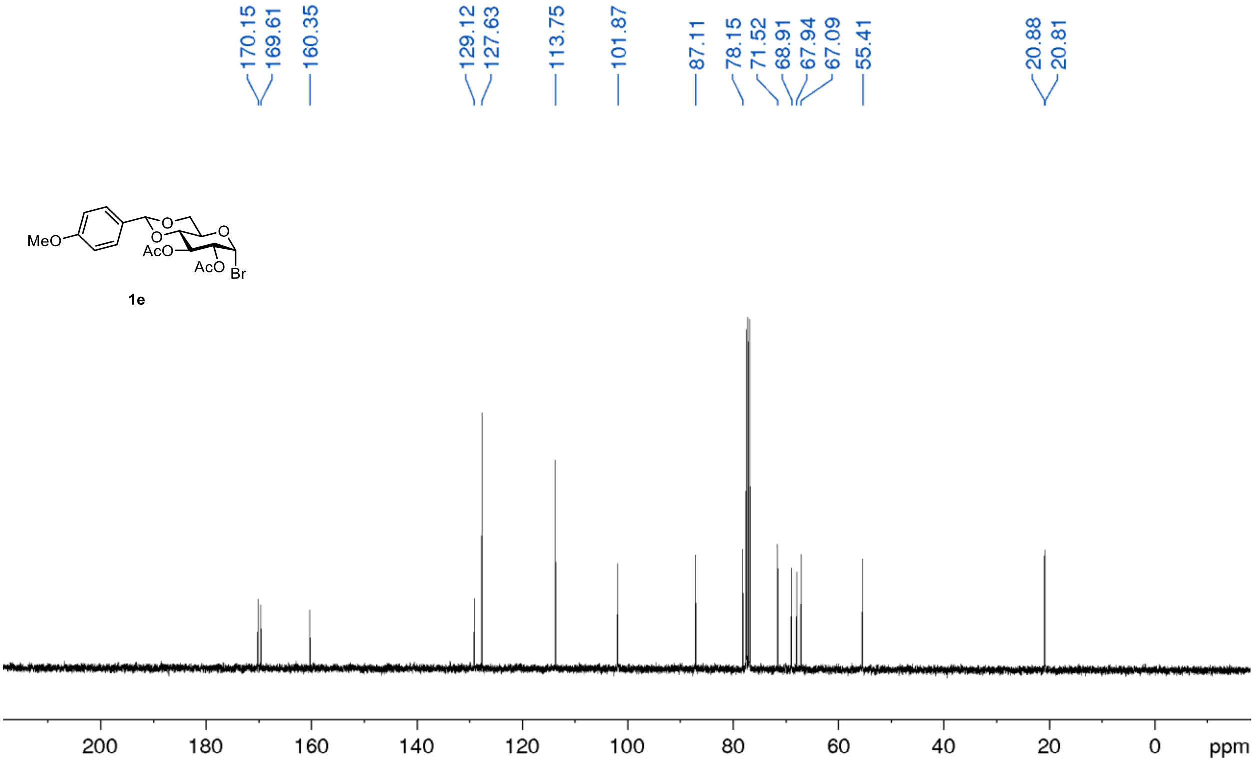

^{1}H NMR (700 MHz, $CDCl_3$, 25 ºC) of (1f)

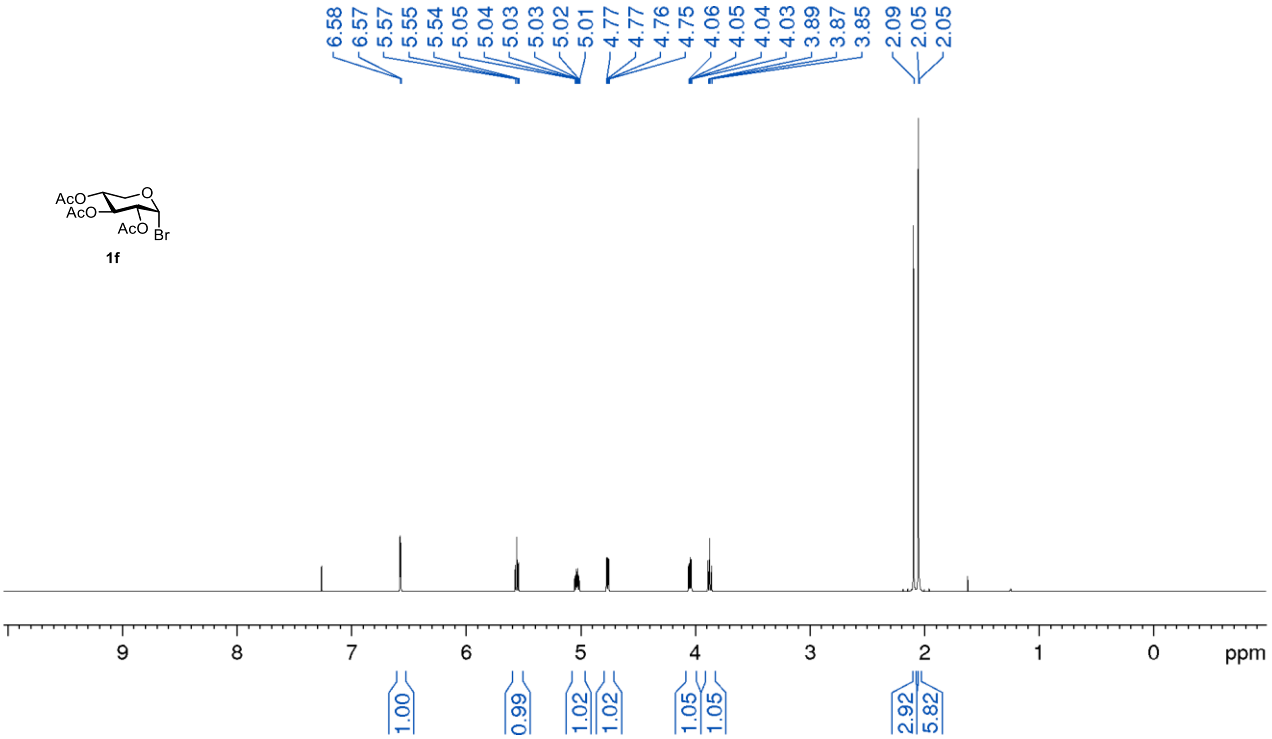

^{13}C NMR (175 MHz, $CDCl_3$, 25 ºC) of (1f)

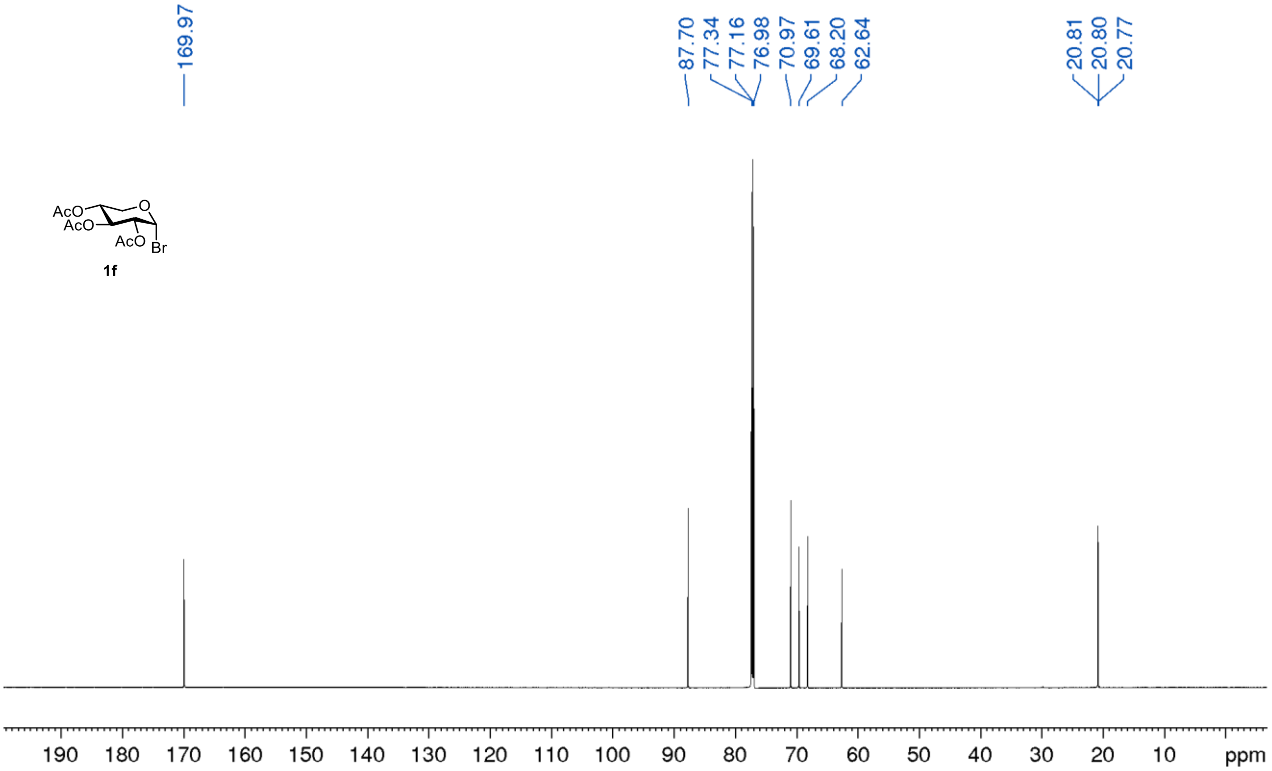

^{1}H NMR (700 MHz, $CDCl_3$, 25 ºC) of (1g)

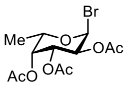

1g

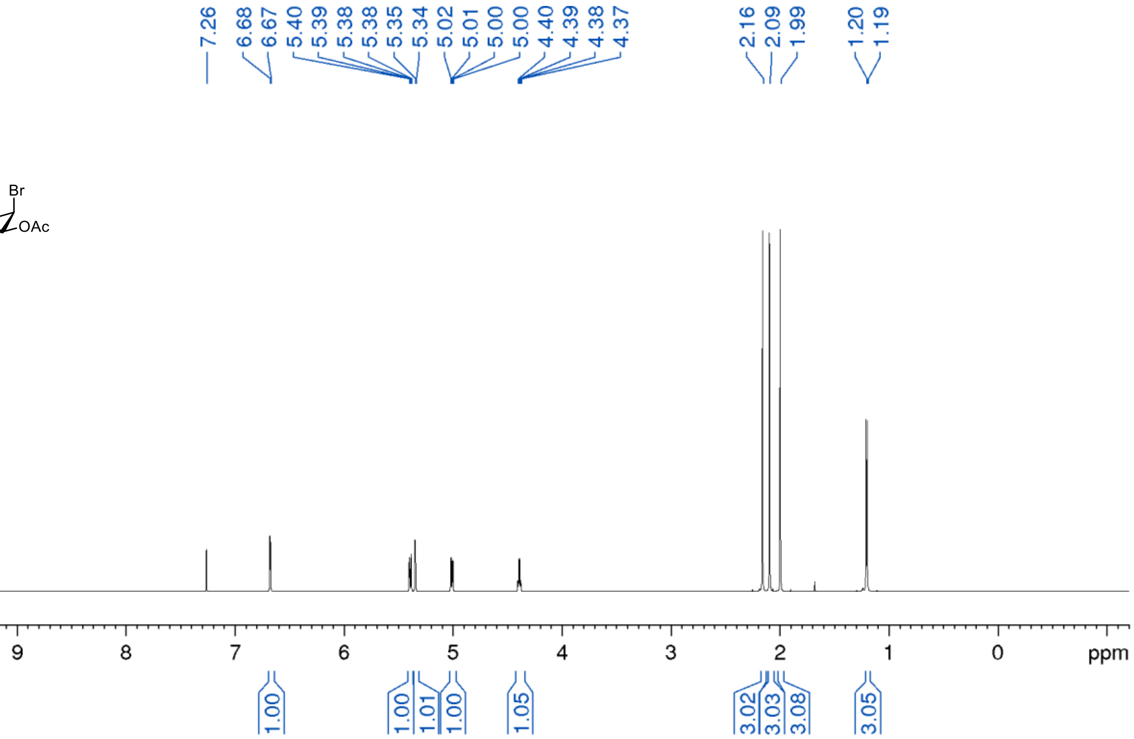

^{13}C NMR (175 MHz, $CDCl_3$, 25 ºC) of (1g)

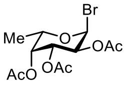

1g

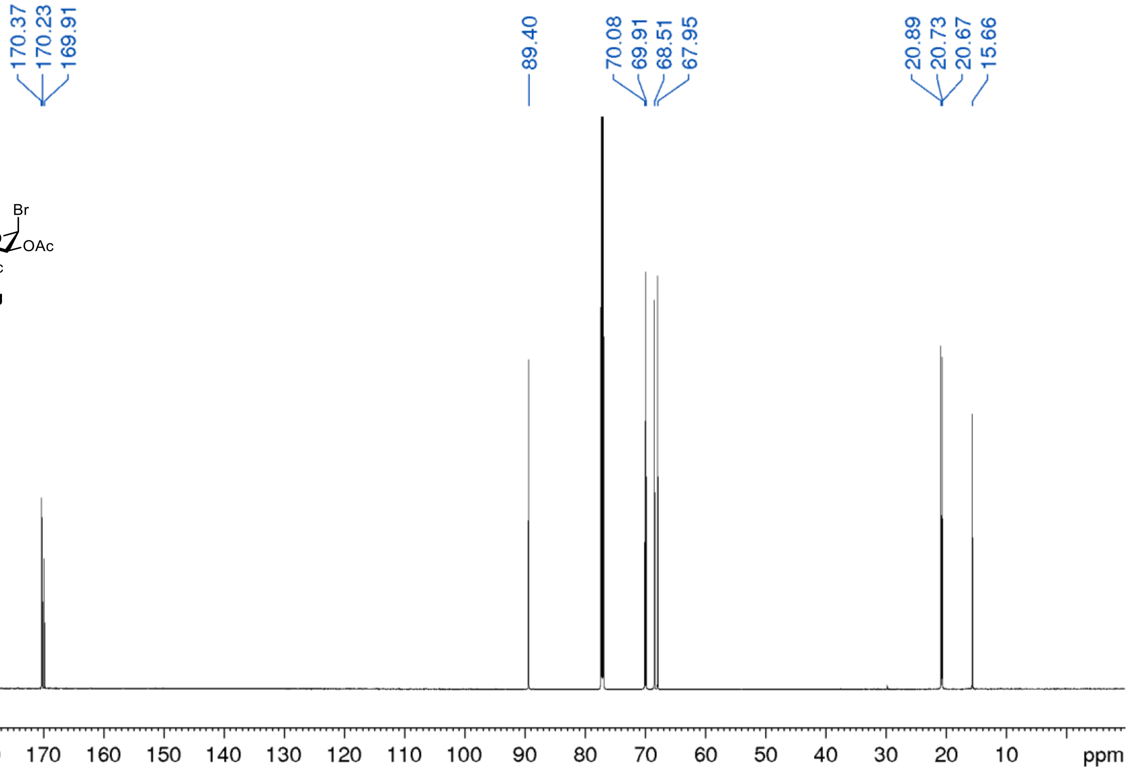

^{1}H NMR (500 MHz, $CDCl_3$, 25 ºC) of (1h)

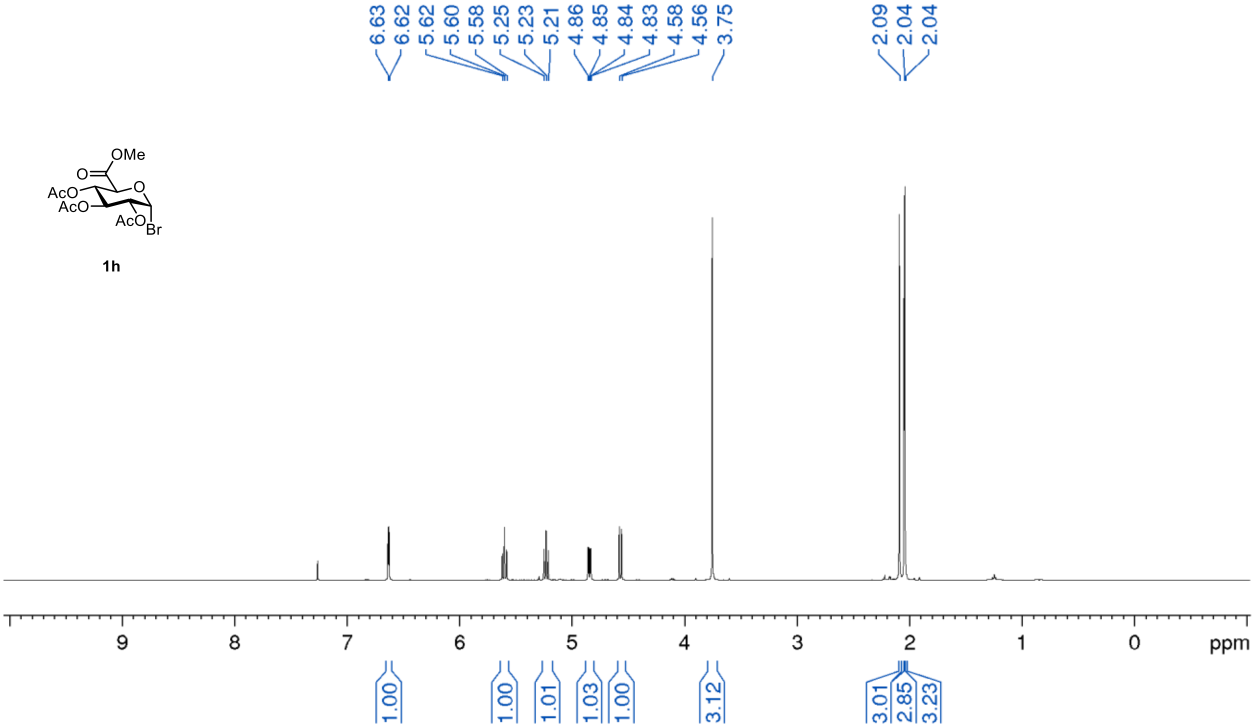

^{13}C NMR (125 MHz, $CDCl_3$, 25 ºC) of (1h)

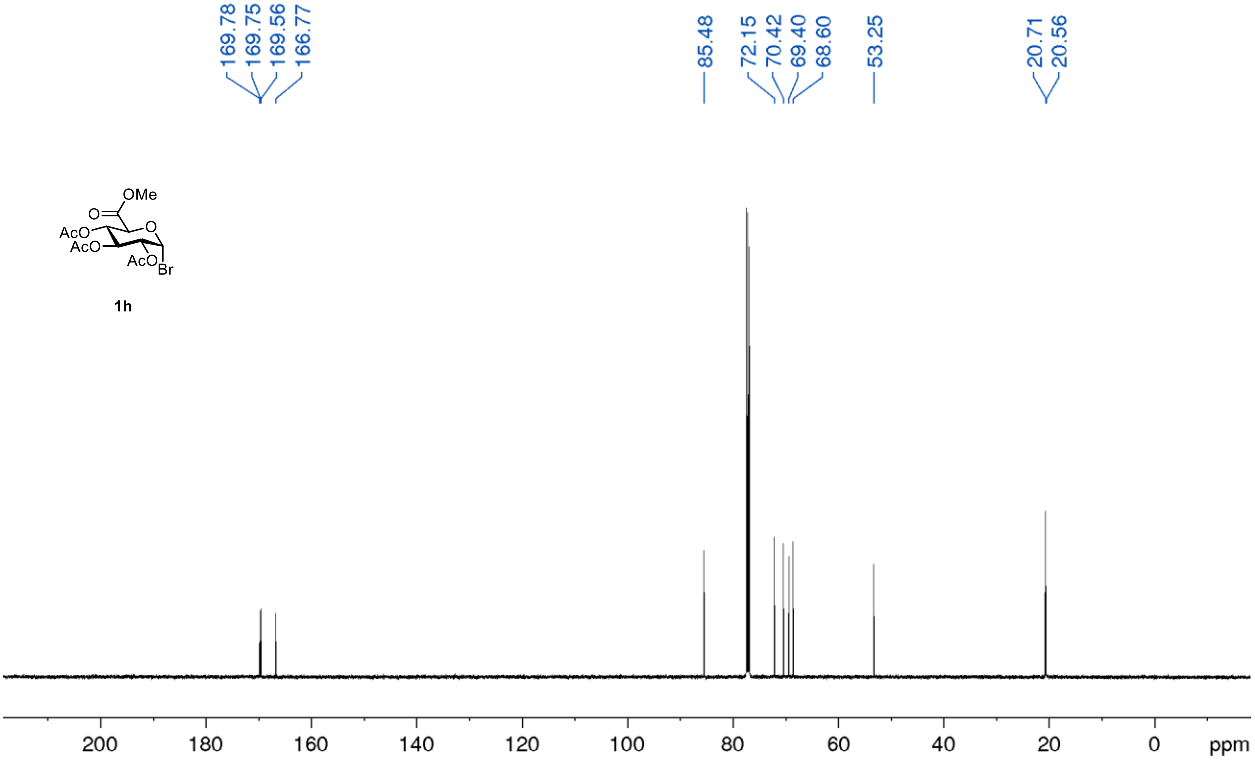

^{1}H NMR (500 MHz, $CDCl_3$, 25 ºC) of (1i)

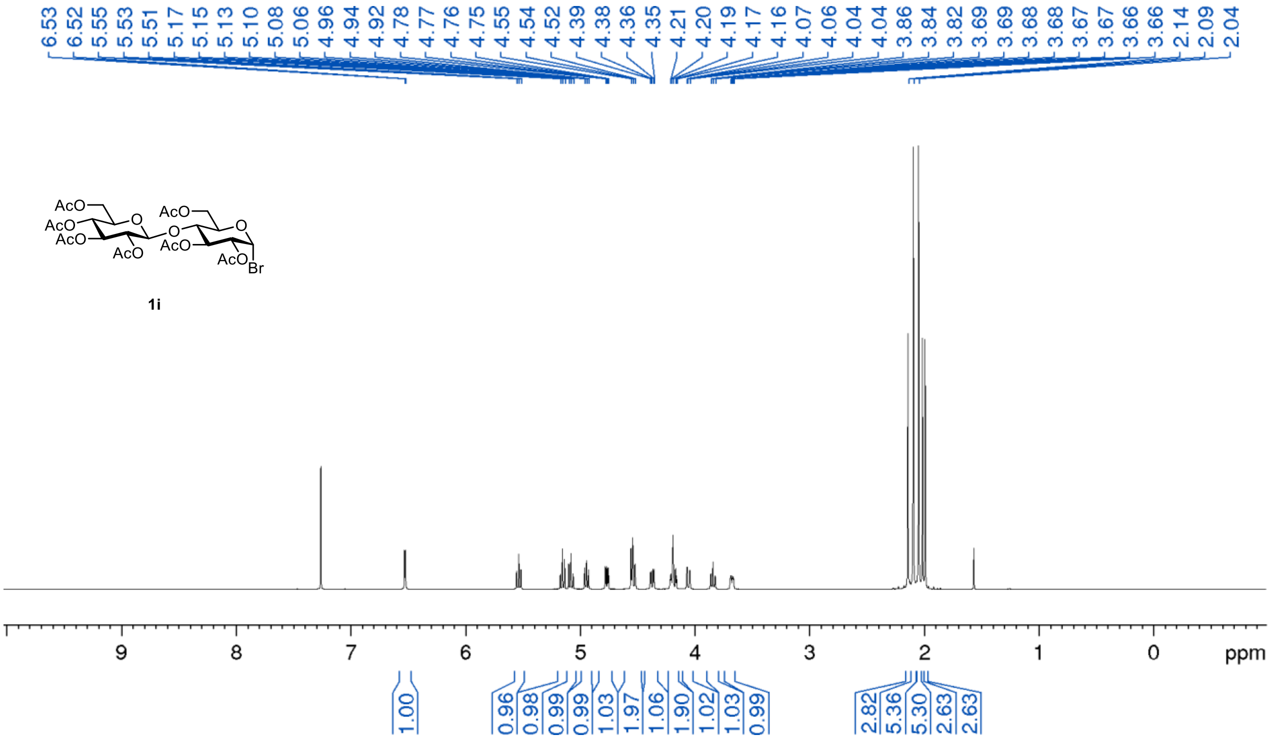

^{13}C NMR (125 MHz, $CDCl_3$, 25 ºC) of (1i)

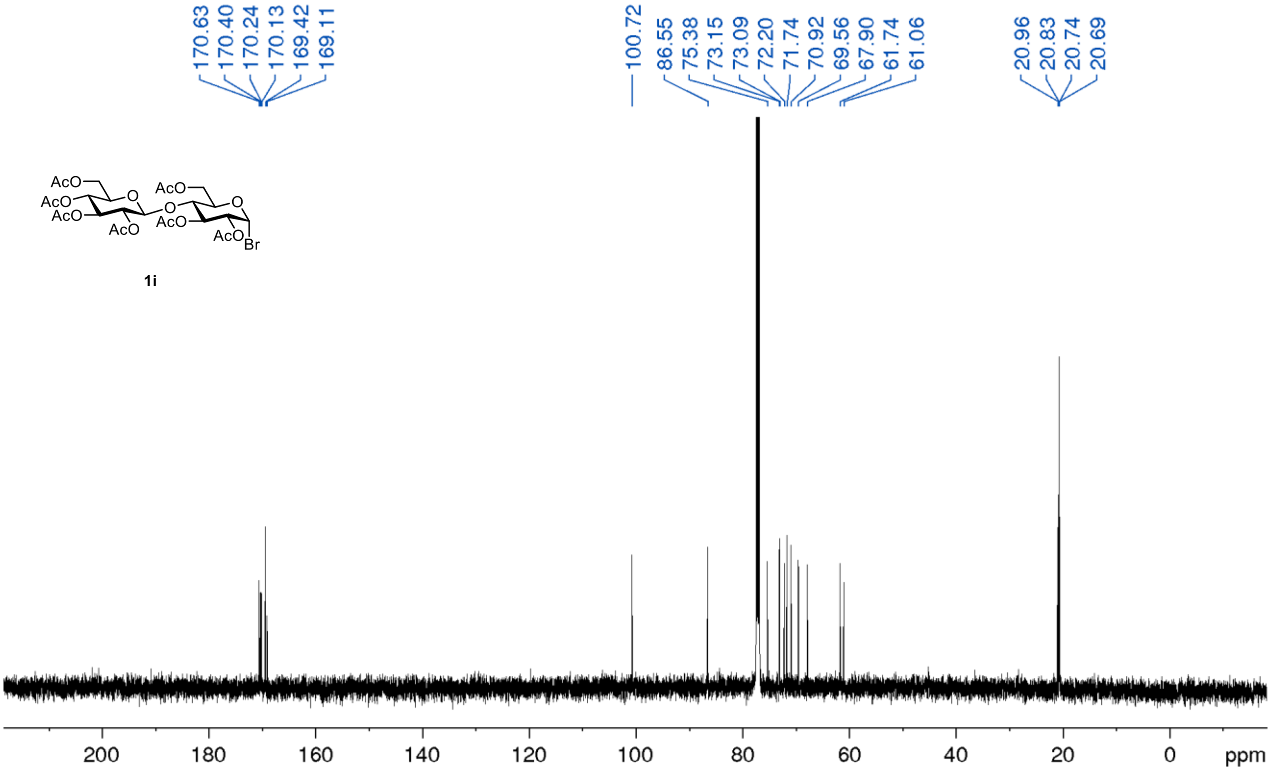

^{1}H NMR (500 MHz, $CDCl_3$, 25 ºC) of (1j)

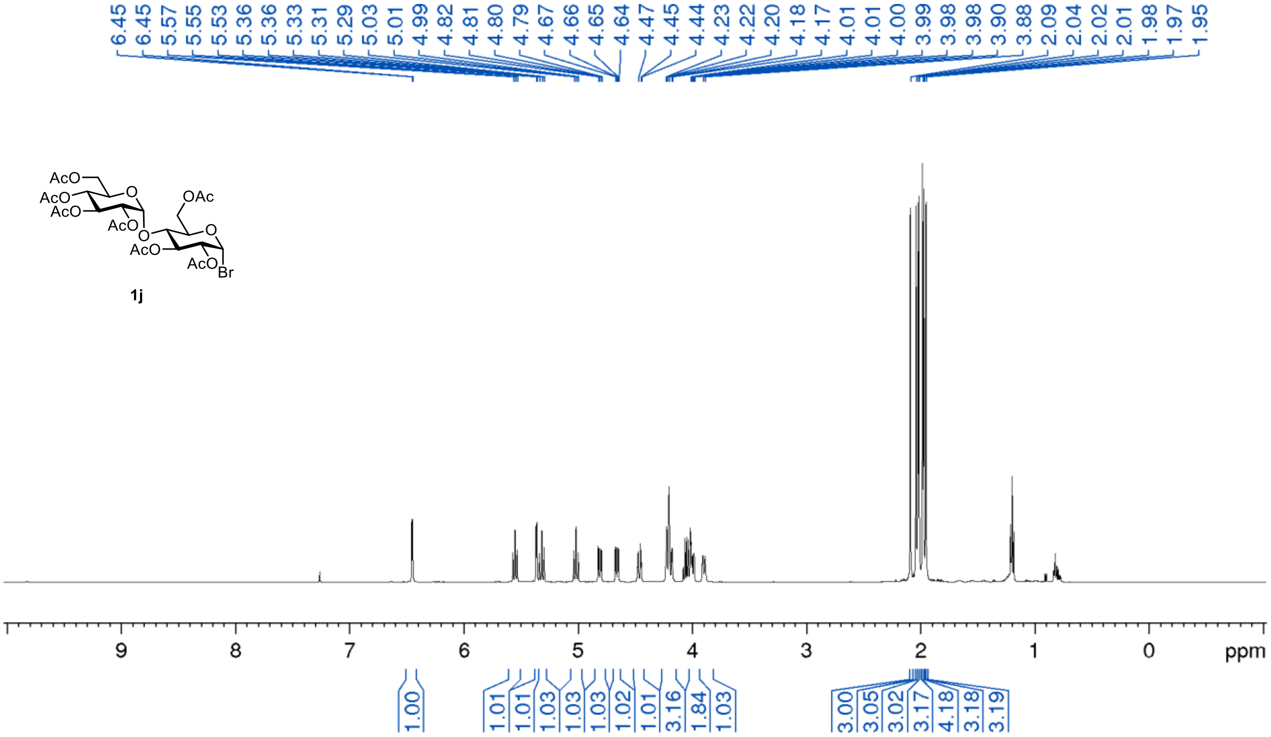

1j

^{13}C NMR (125 MHz, $CDCl_3$, 25 ºC) of (1j)

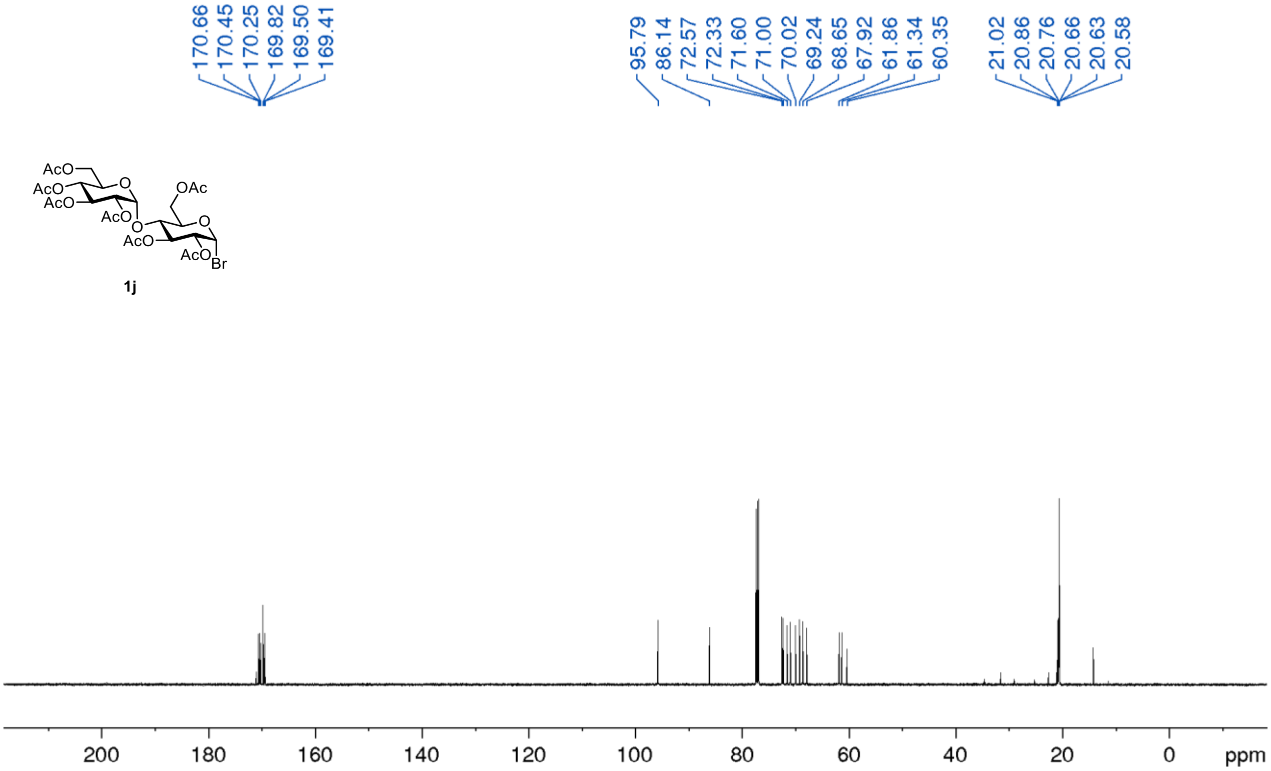

1j

^{1}H NMR (700 MHz, $CDCl_3$, 25 °C) of (1k)

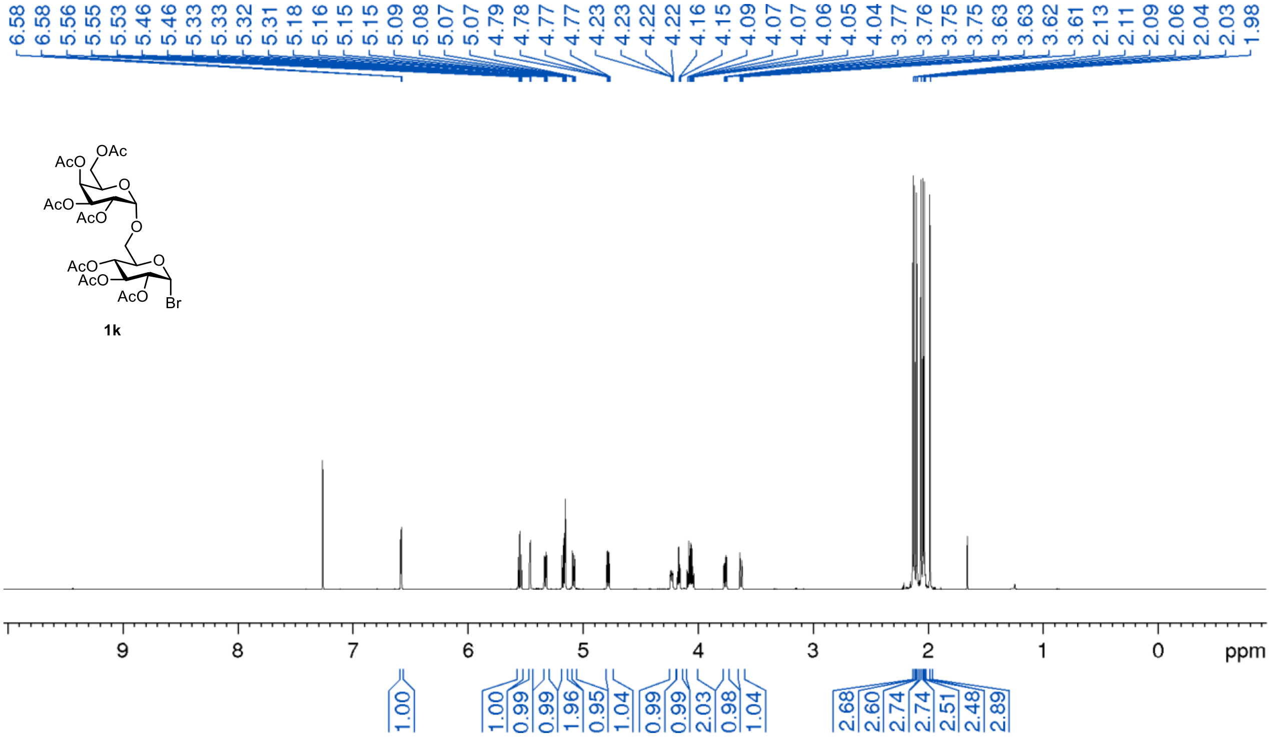

^{13}C NMR (175 MHz, $CDCl_3$, 25 °C) of (1k)

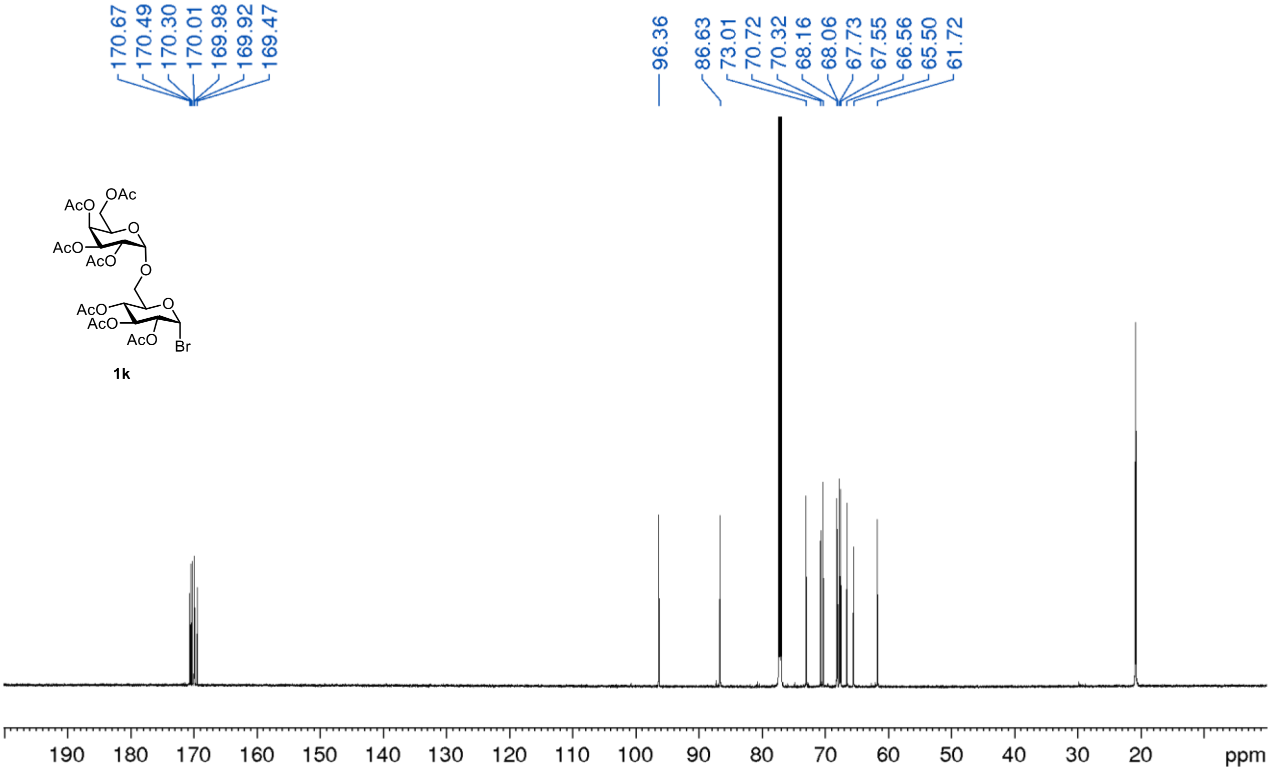

^{1}H NMR (500 MHz, $CDCl_3$, 25 °C) of (1l)

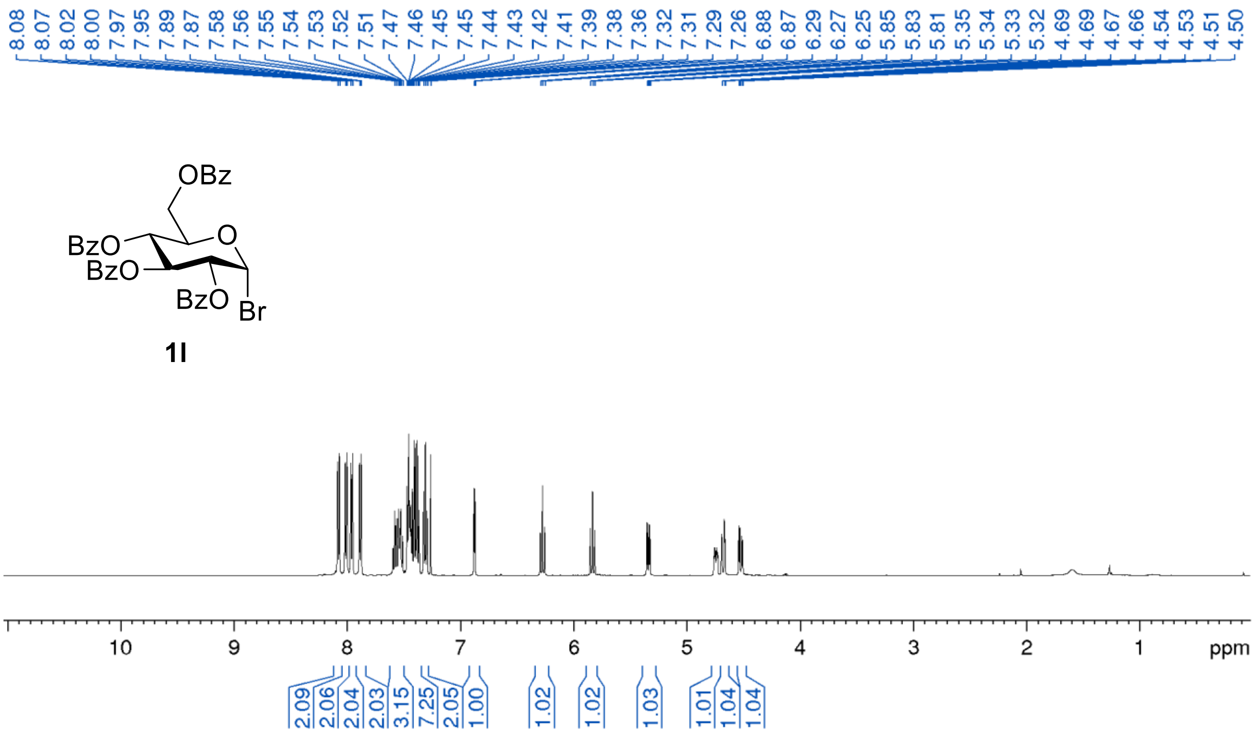

^{13}C NMR (125 MHz, $CDCl_3$, 25 °C) of (1l)

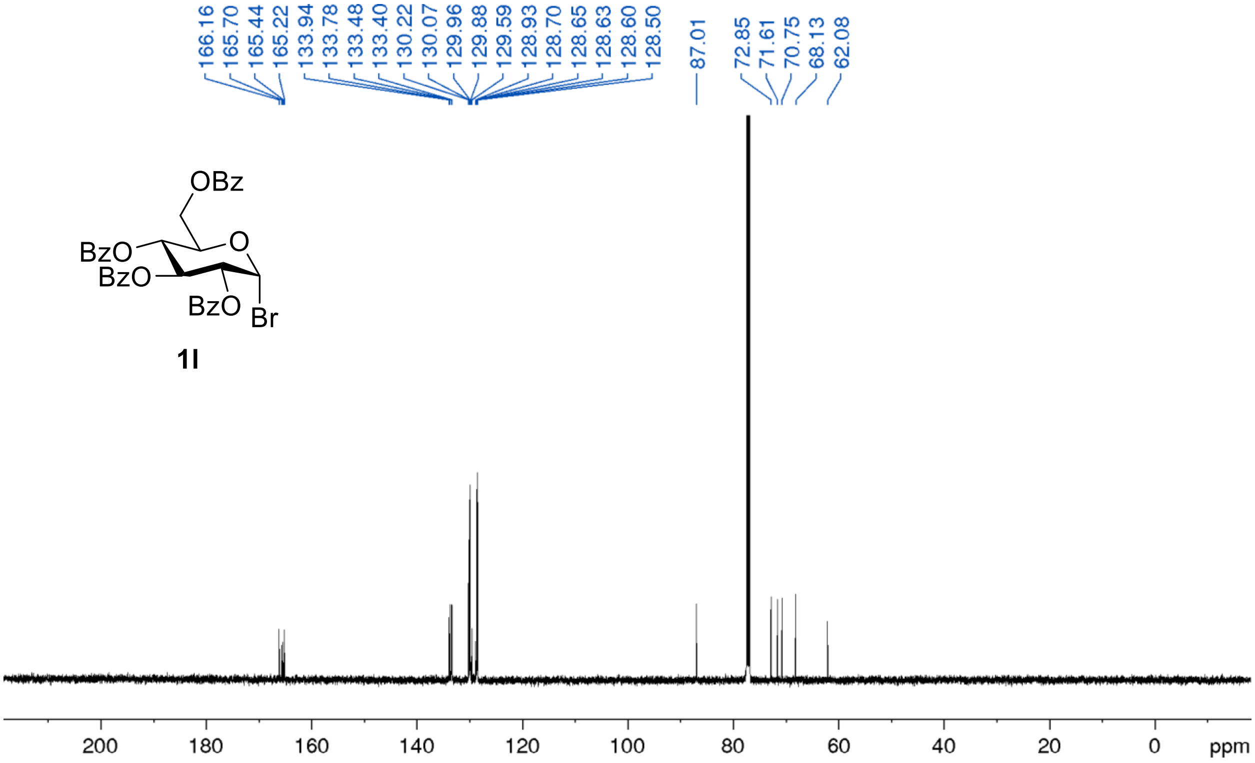

^{1}H NMR (500 MHz, $CDCl_3$, 25 °C) of (1m)

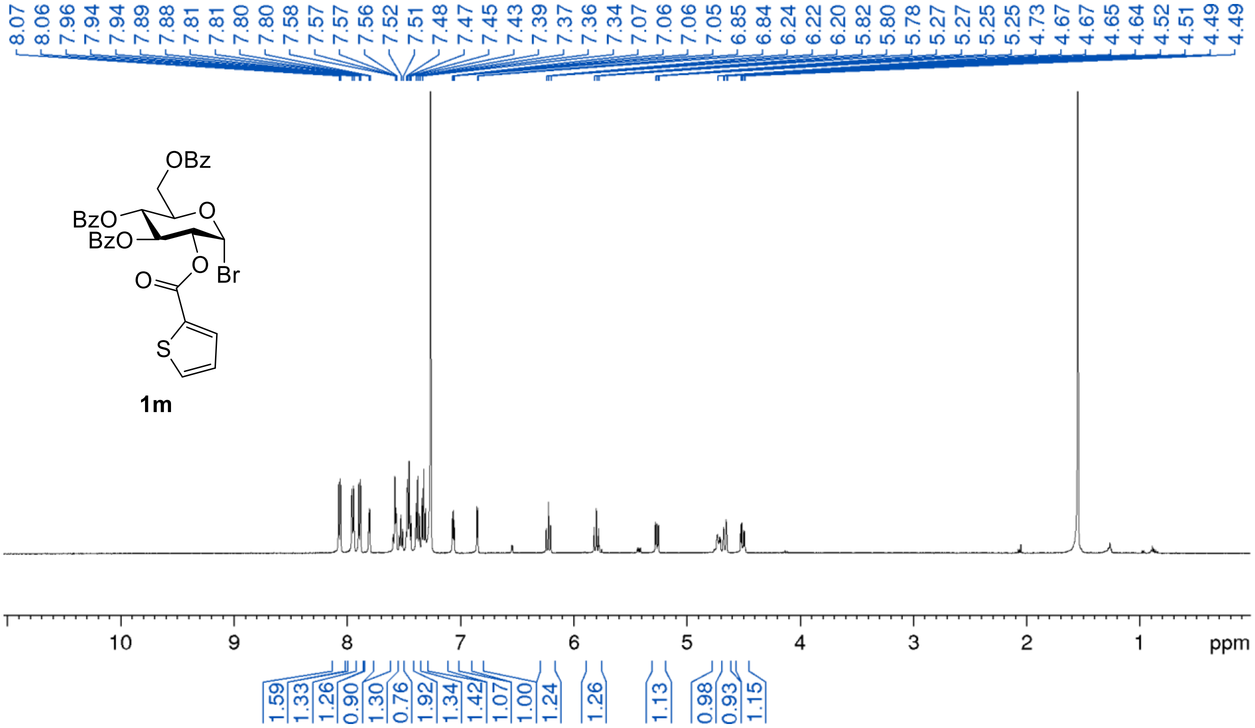

^{13}C NMR (175 MHz, $CDCl_3$, 25 °C) of (1m)

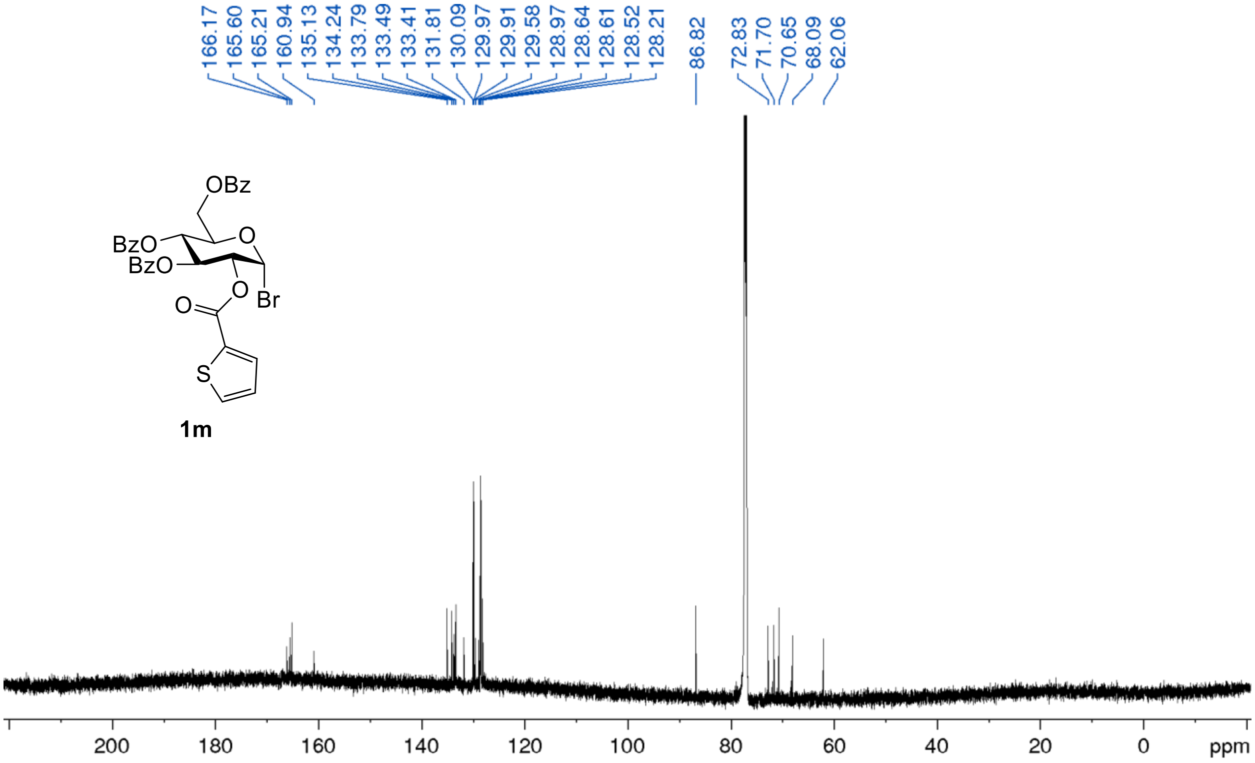

^{1}H NMR (400 MHz, $CDCl_3$, 25 ºC) of (1n)

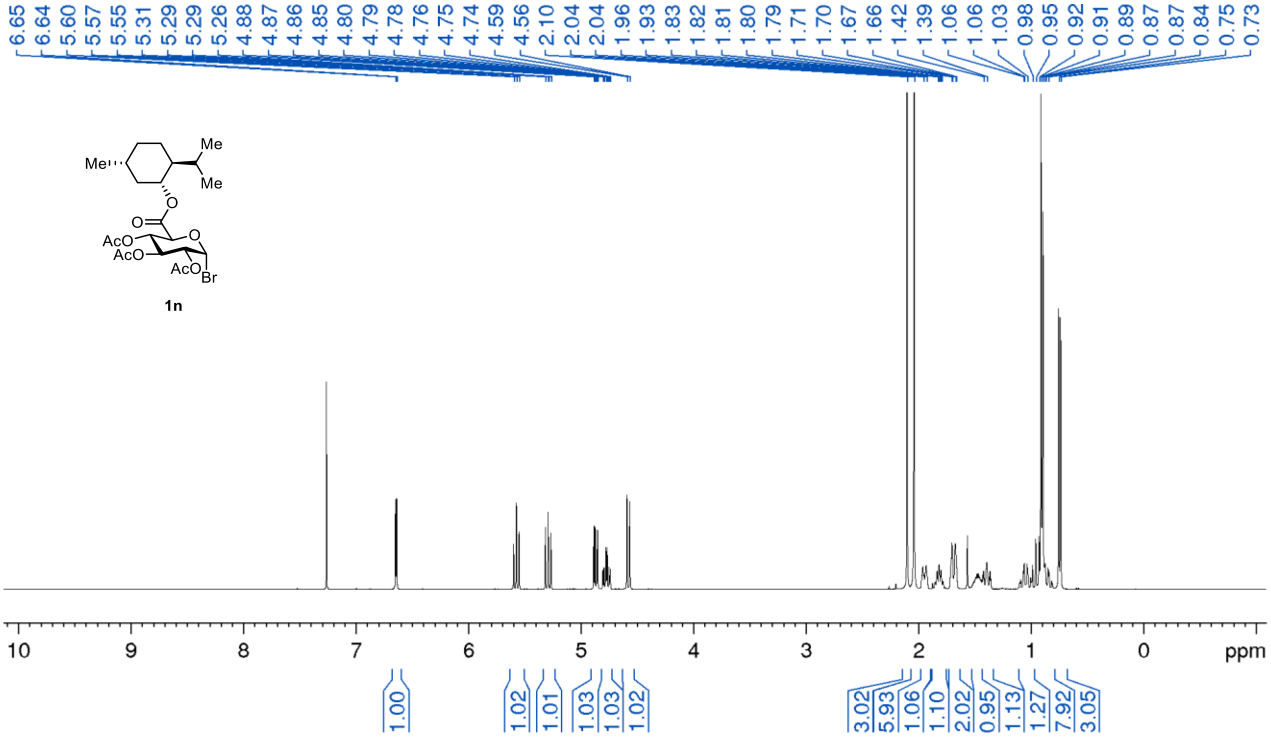

^{13}C NMR (100 MHz, $CDCl_3$, 25 ºC) of (1n)

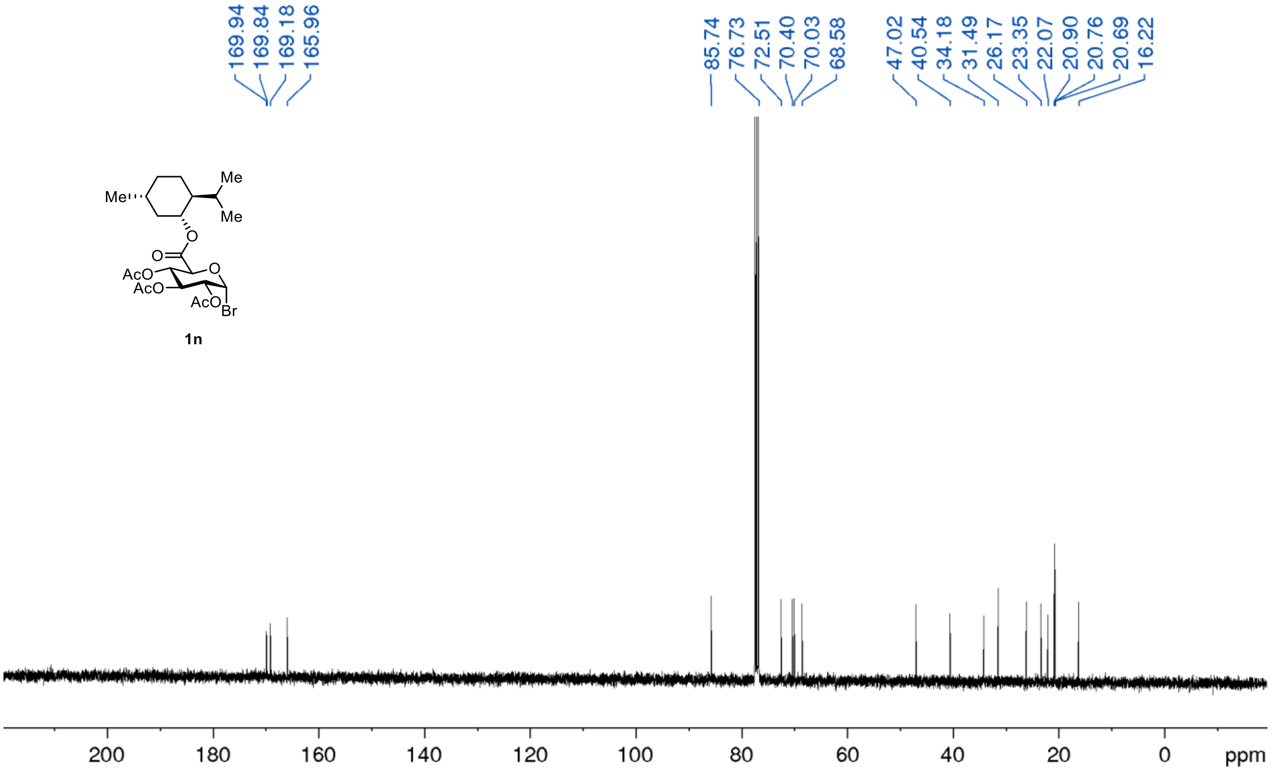

^{1}H NMR (500 MHz, $CDCl_3$, 25 °C) of (1o)

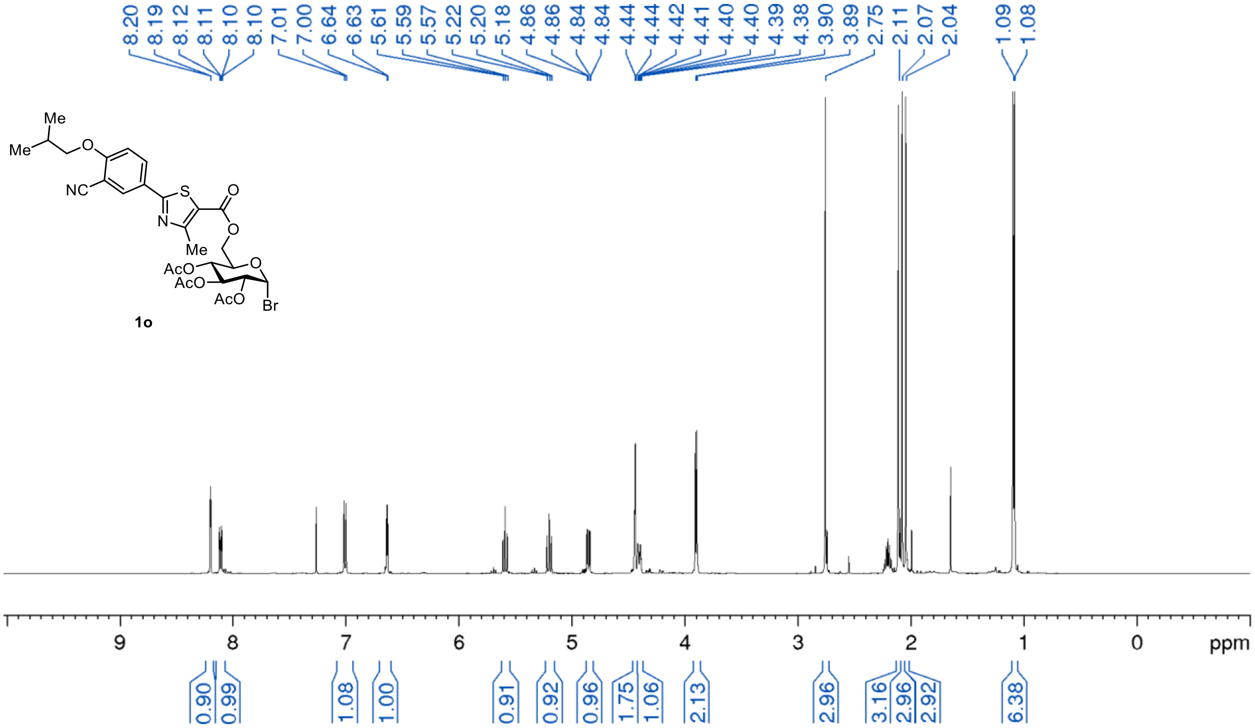

^{13}C NMR (125 MHz, $CDCl_3$, 25 °C) of (1o)

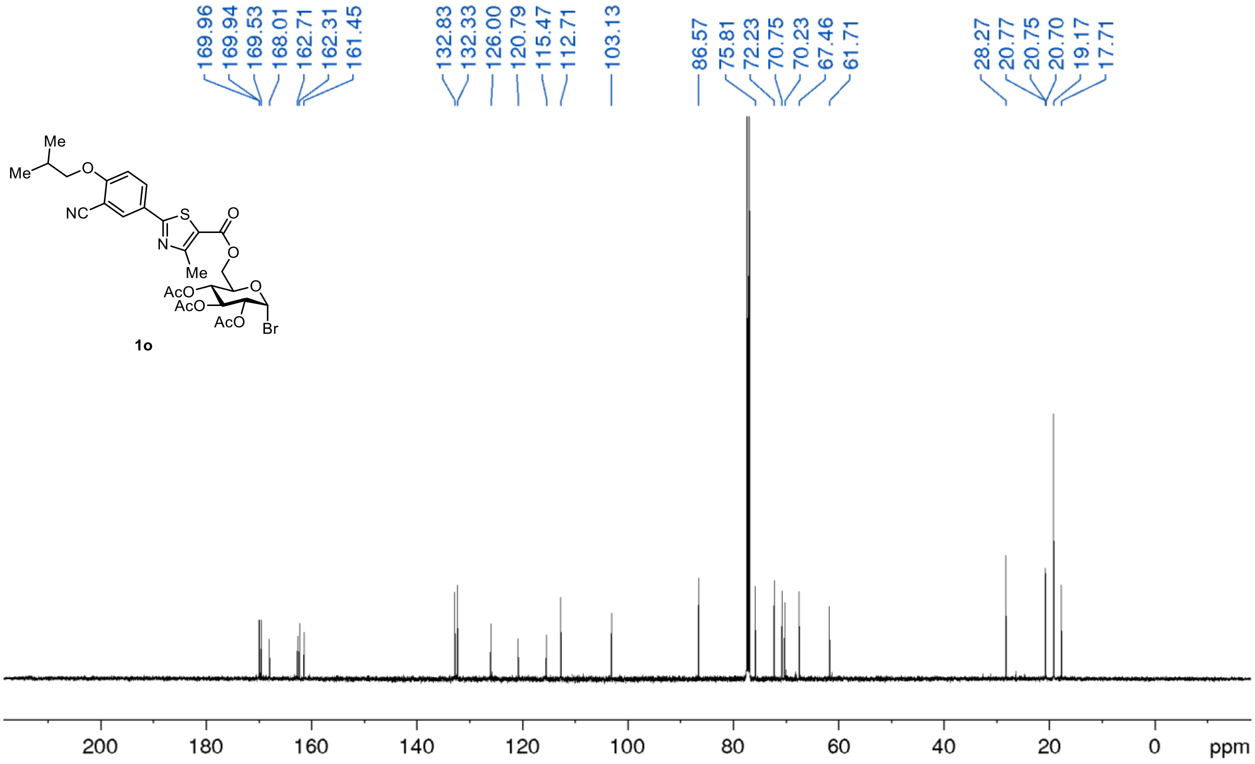

^{1}H NMR (700 MHz, $CDCl_3$, 25 °C) of (1p)

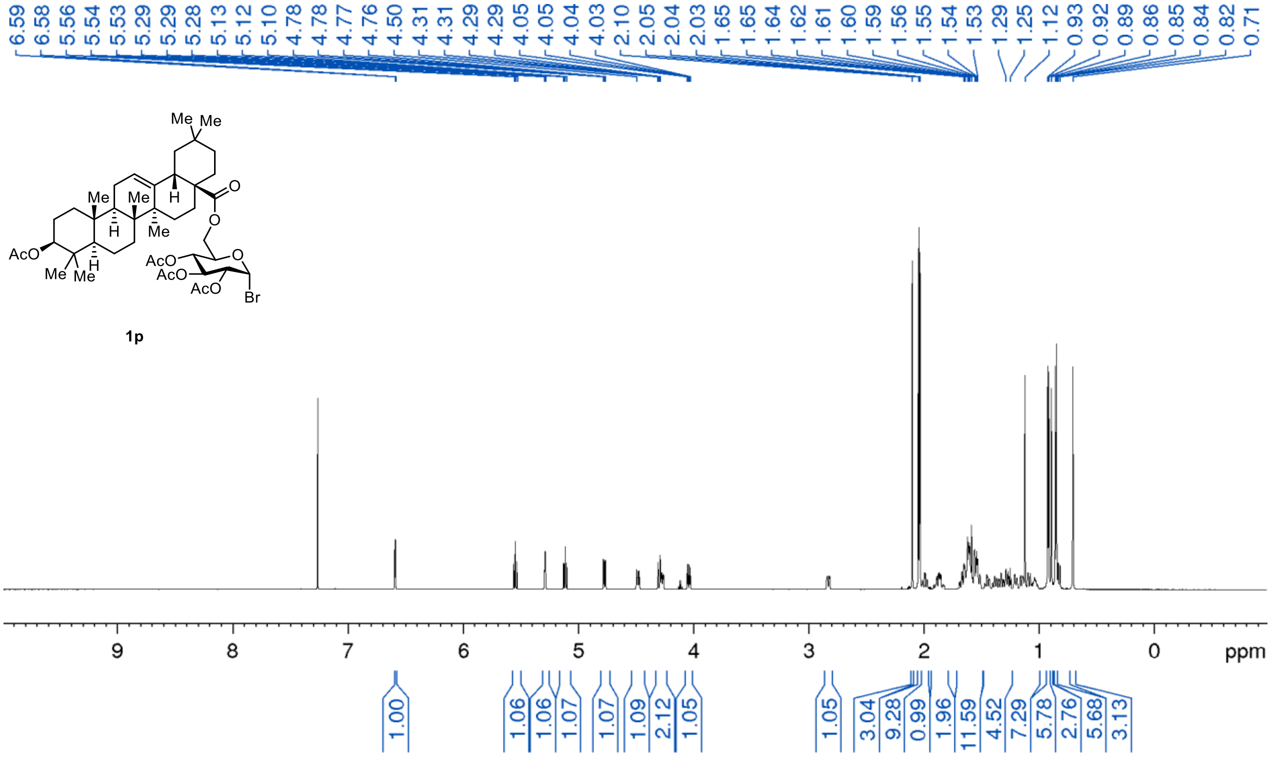

^{13}C NMR (175 MHz, $CDCl_3$, 25 °C) of (1p)

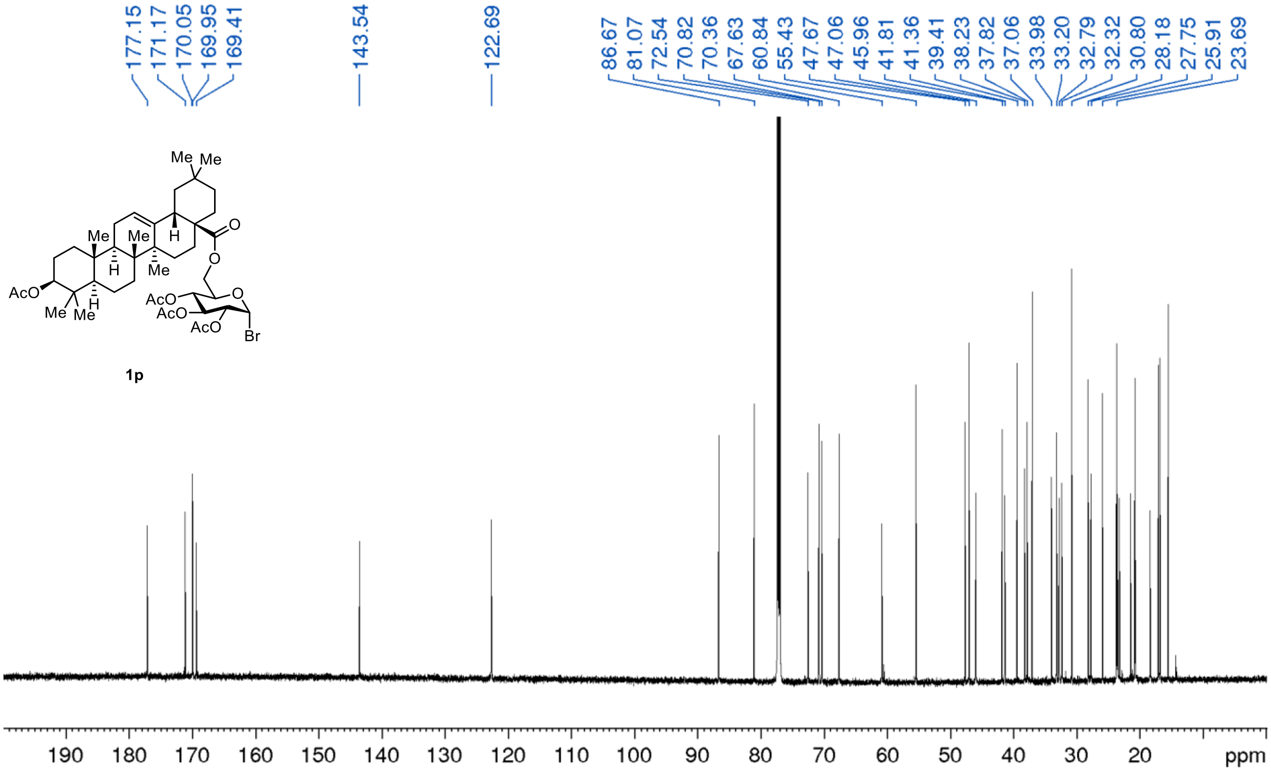

^{1}H NMR (700 MHz, CDCl$_3$, 25 °C) of (1q)

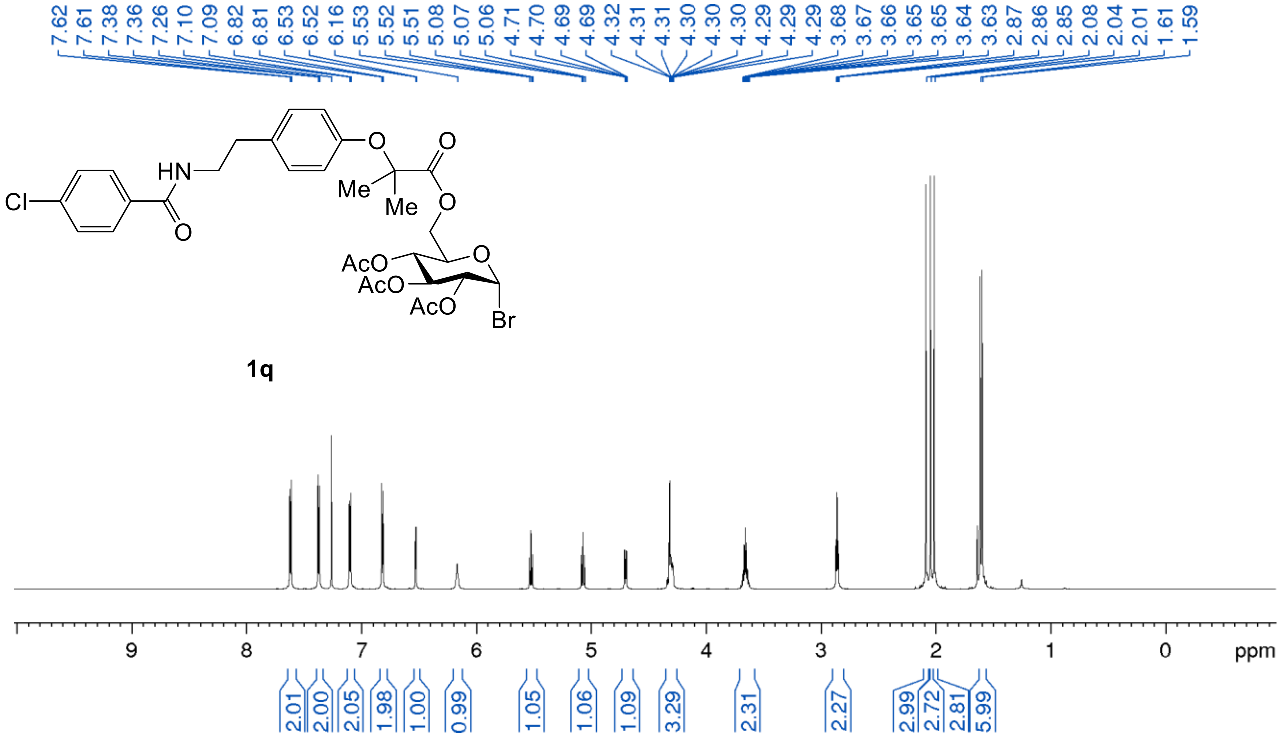

^{13}C NMR (175 MHz, CDCl$_3$, 25 °C) of (1q)

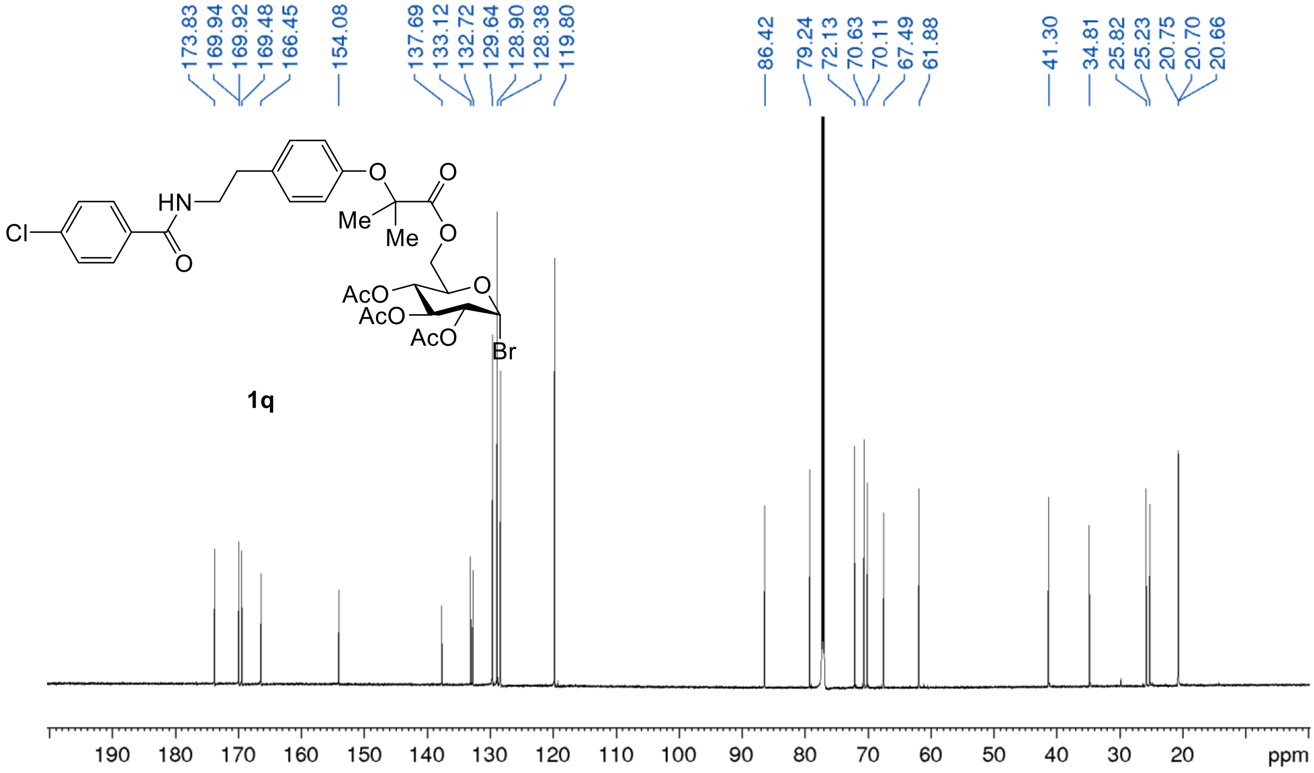

^{1}H NMR (500 MHz, $CDCl_3$, 25 °C) of (1r)

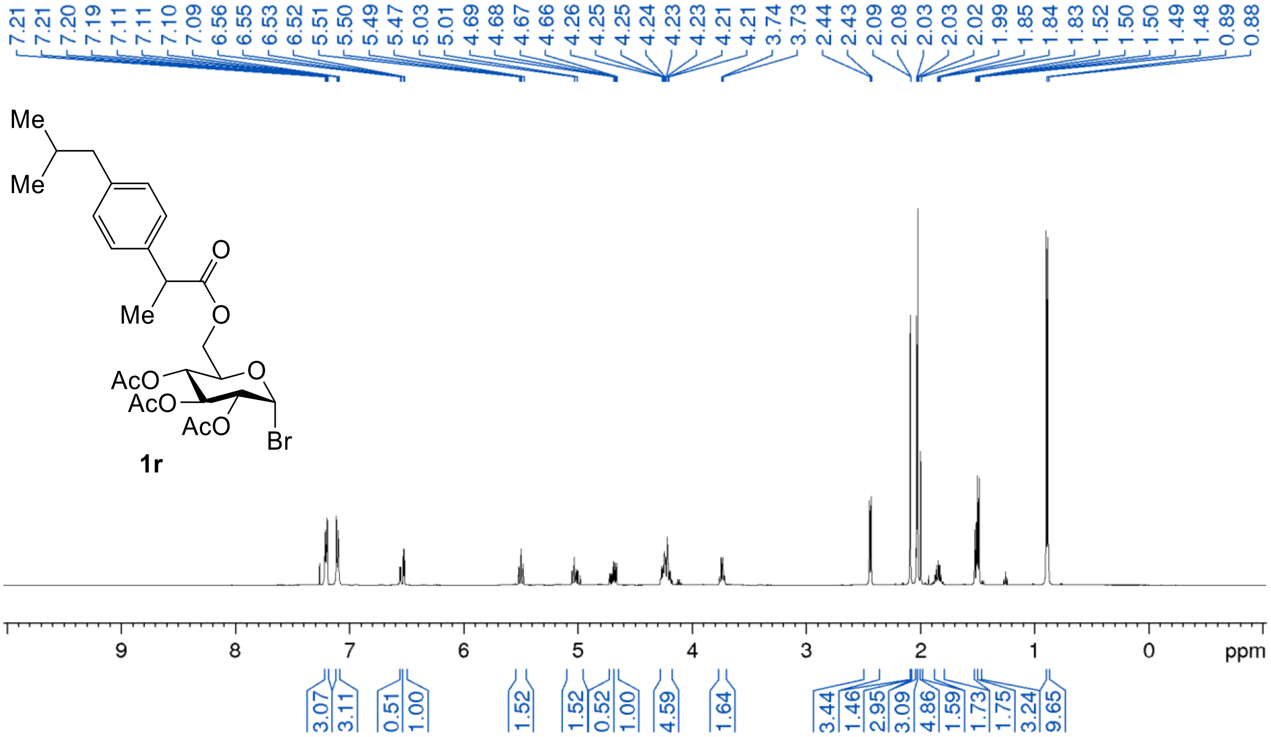

^{13}C NMR (125 MHz, $CDCl_3$, 25 °C) of (1r)

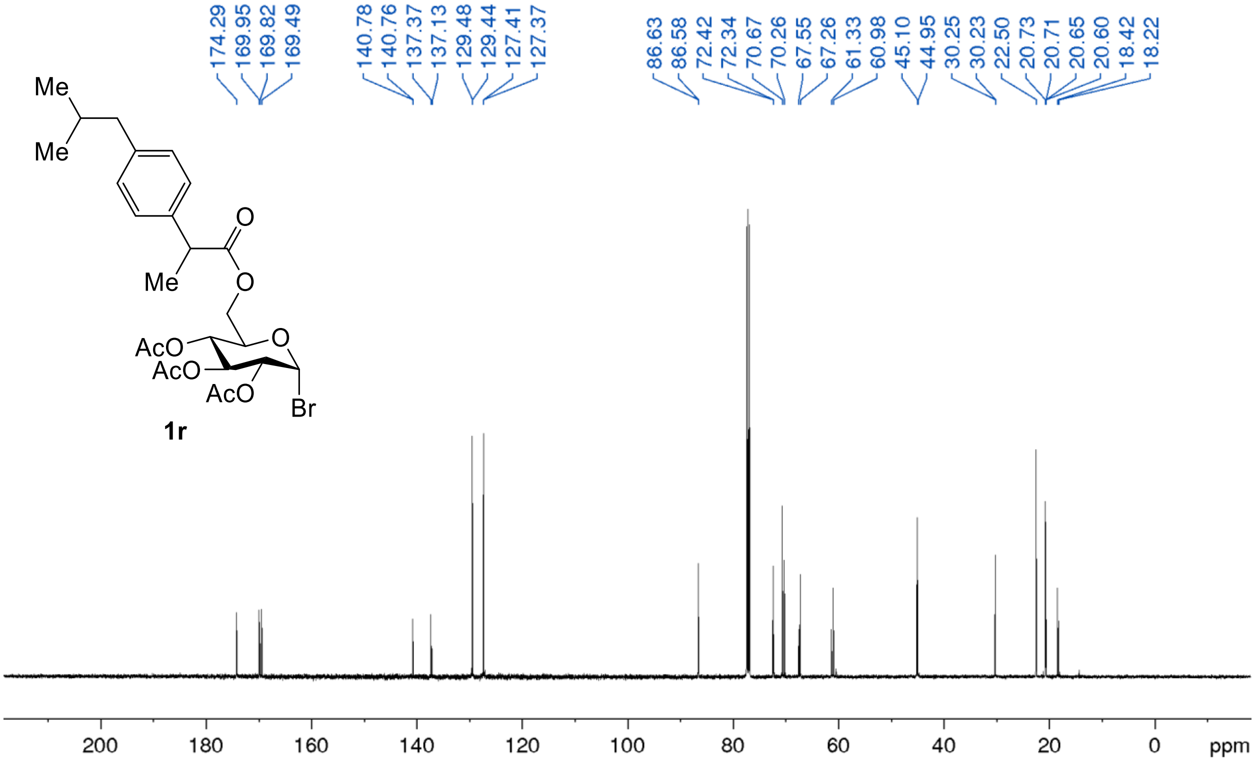

^{1}H NMR (700 MHz, $CDCl_3$, 25 °C) of (1s)

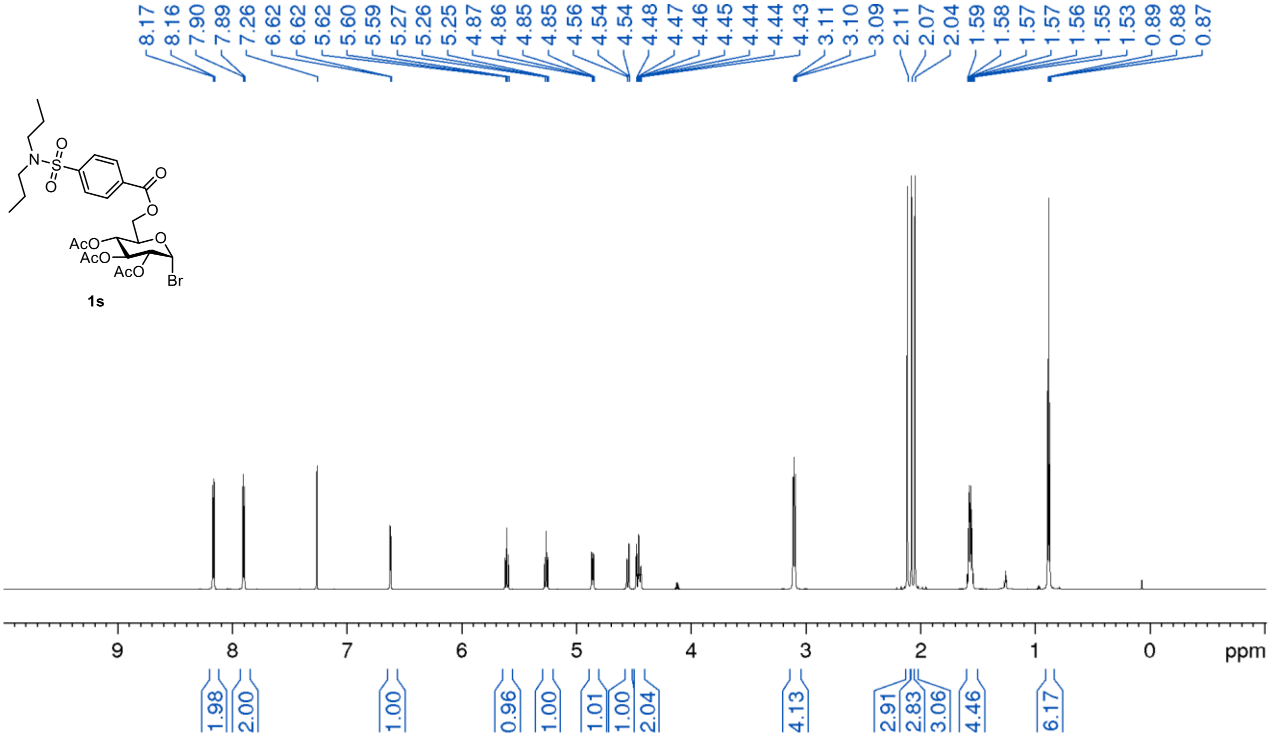

^{13}C NMR (175 MHz, $CDCl_3$, 25 °C) of (1s)

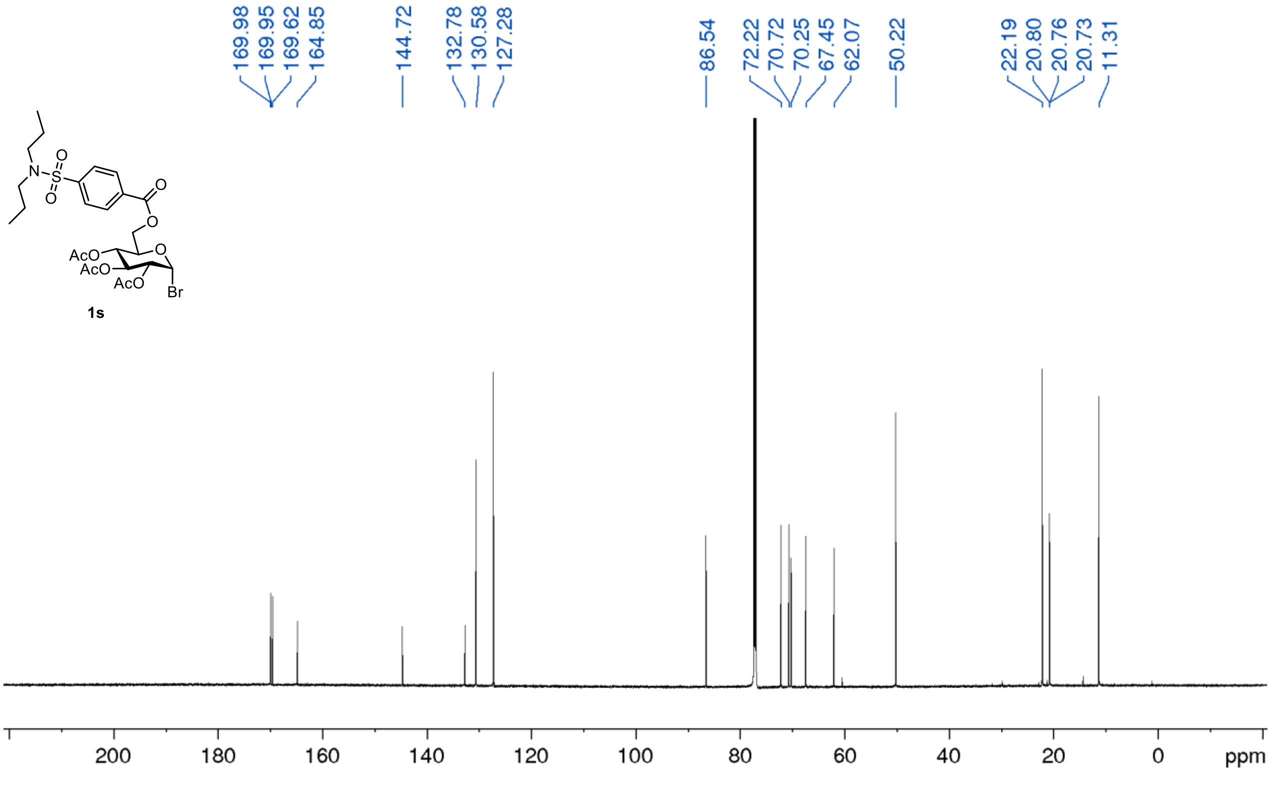

^{1}H NMR (700 MHz, $CDCl_3$, 25 °C) of (1t)

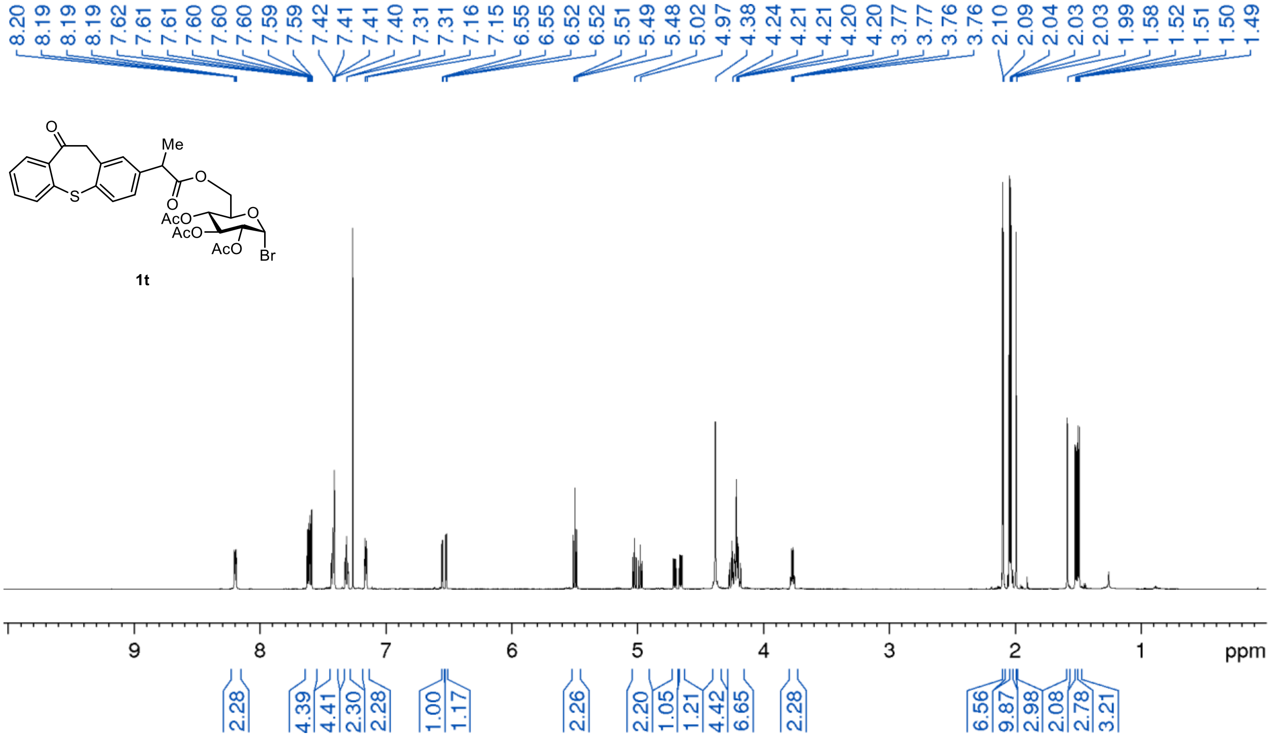

^{13}C NMR (175 MHz, $CDCl_3$, 25 °C) of (1t)

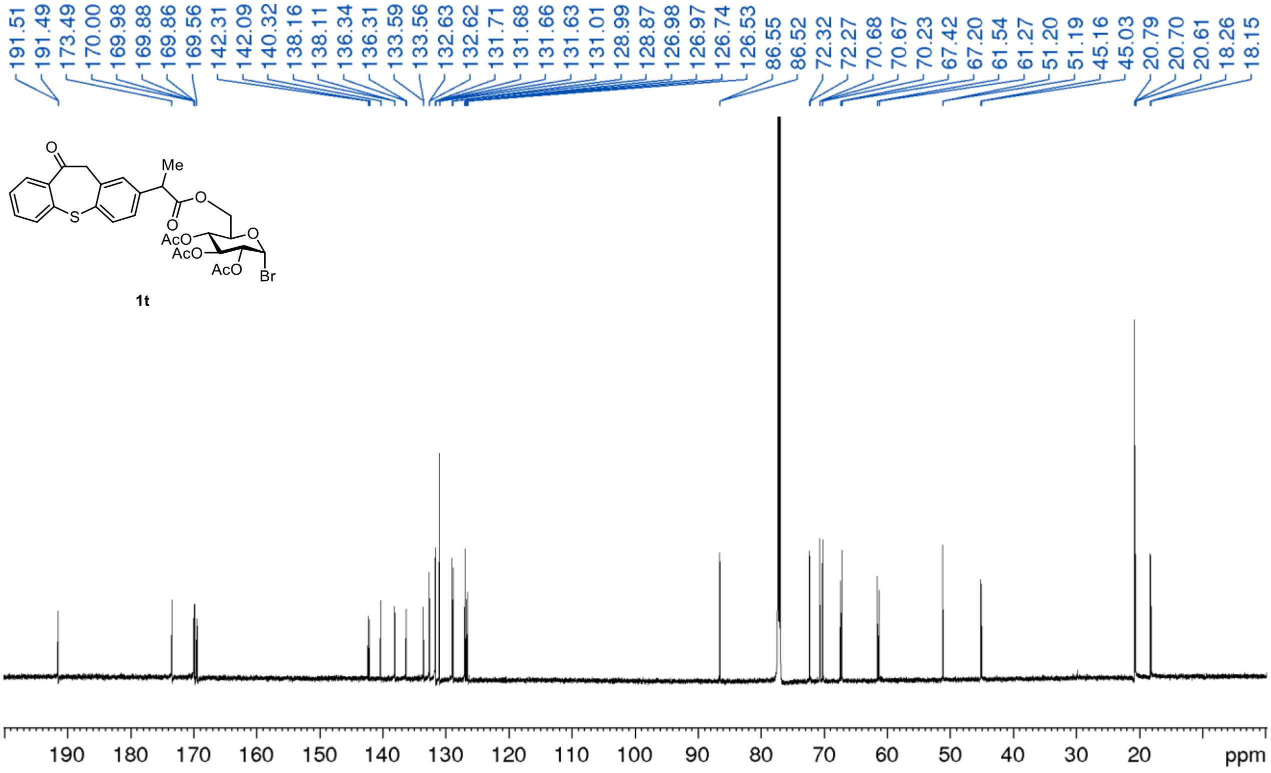

^{1}H NMR (500 MHz, $CDCl_3$, 25 °C) of (1u)

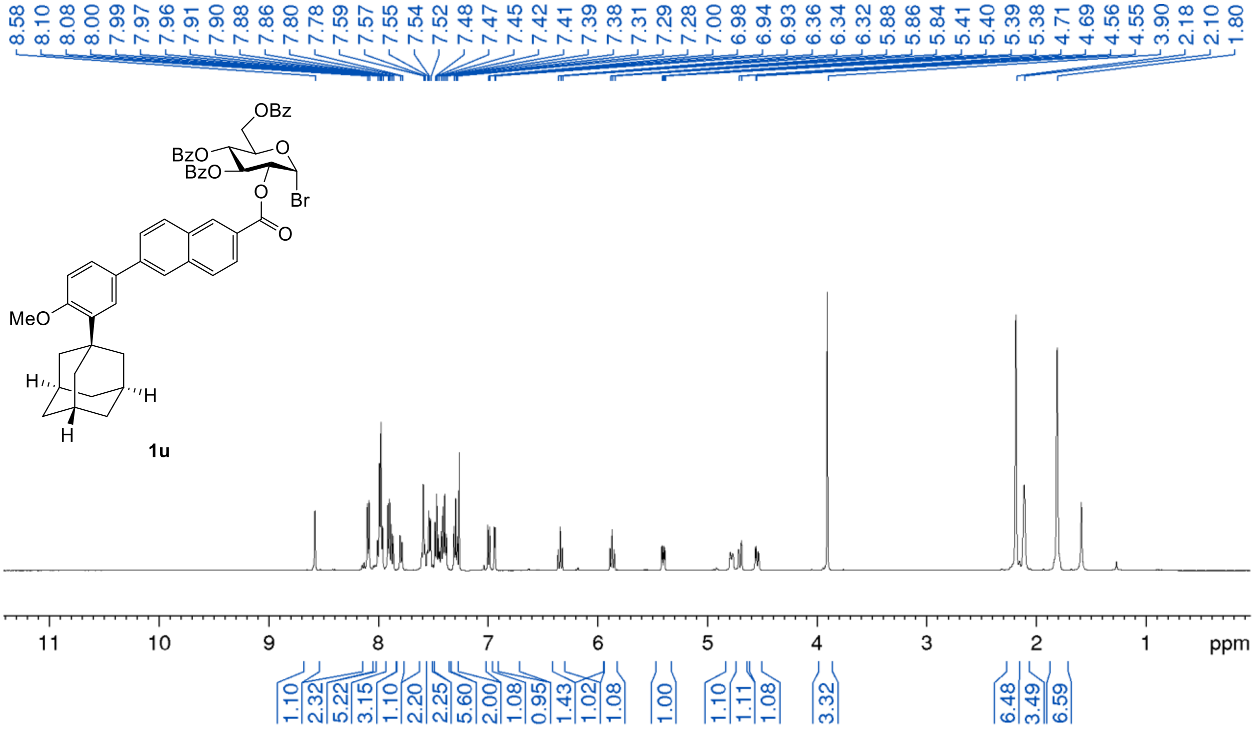

^{13}C NMR (125 MHz, $CDCl_3$, 25 °C) of (1u)

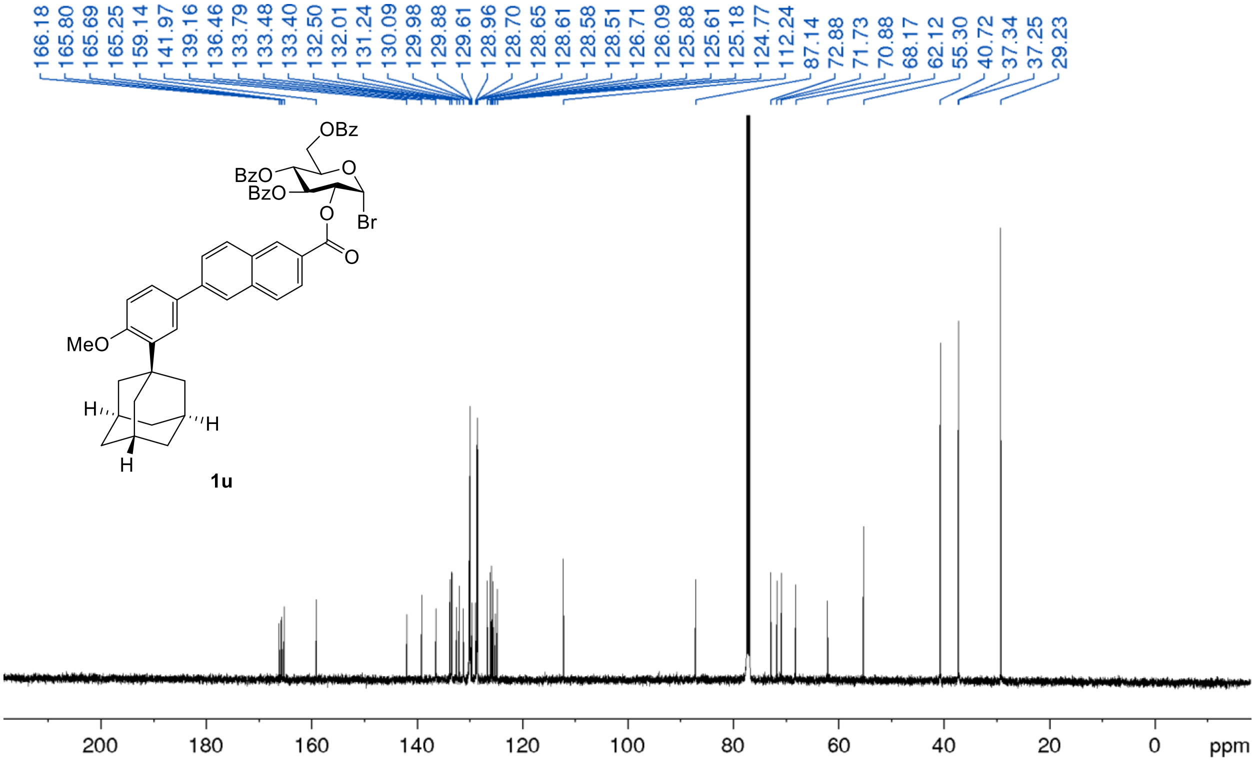

^{1}H NMR (700 MHz, $CDCl_3$, 25 °C) of (3a-*ax*)

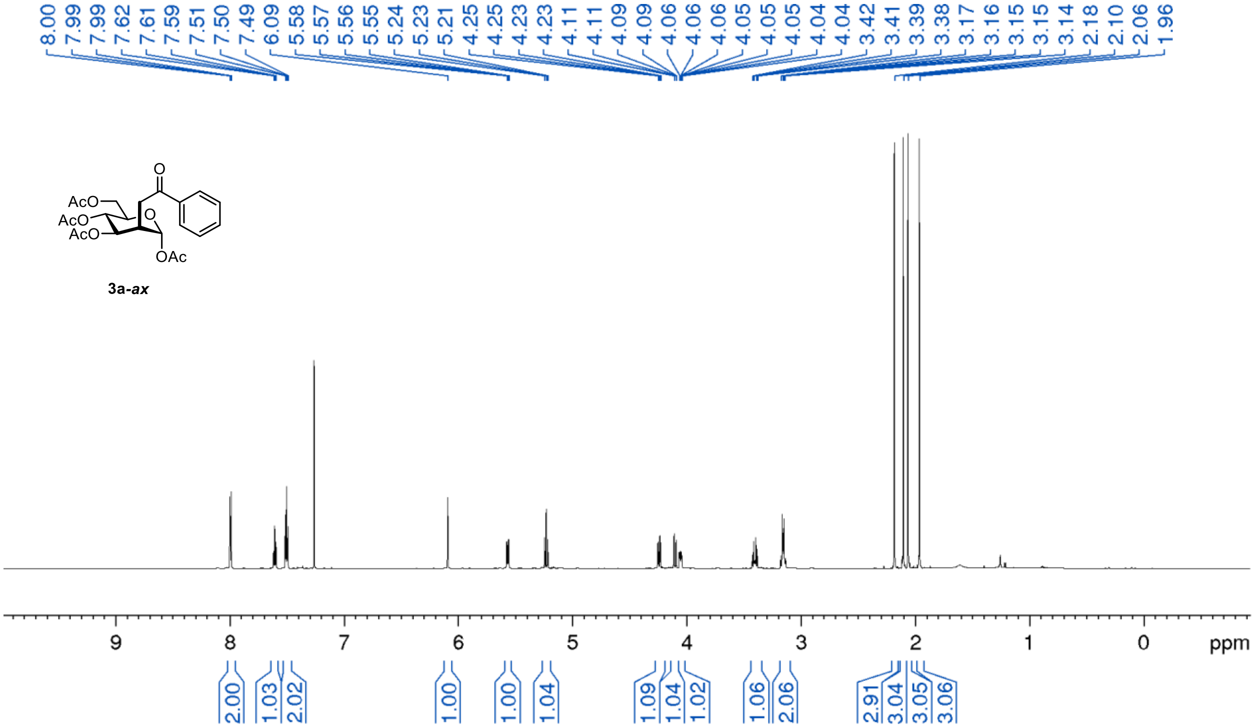

^{13}C NMR (175 MHz, $CDCl_3$, 25 °C) of (3a-*ax*)

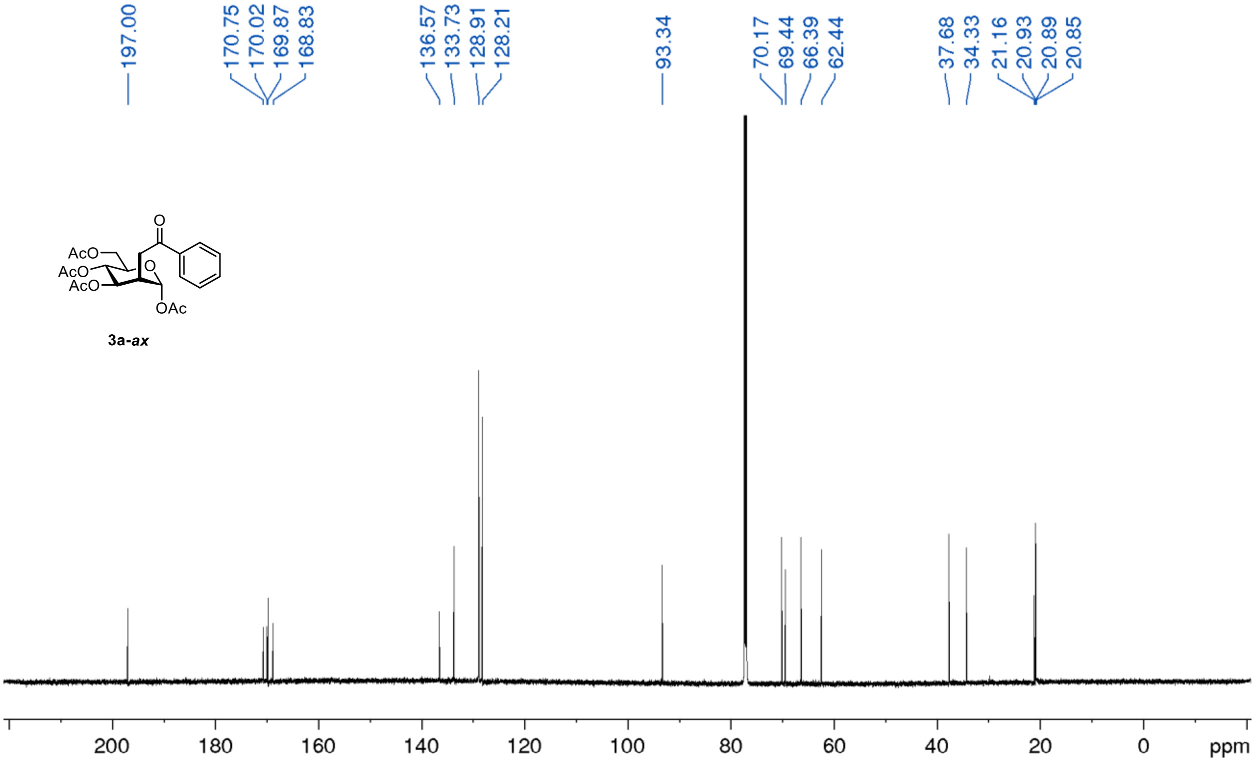

^{1}H NMR (700 MHz, CDCl$_3$, 25 ºC) of (3a-*eq*)

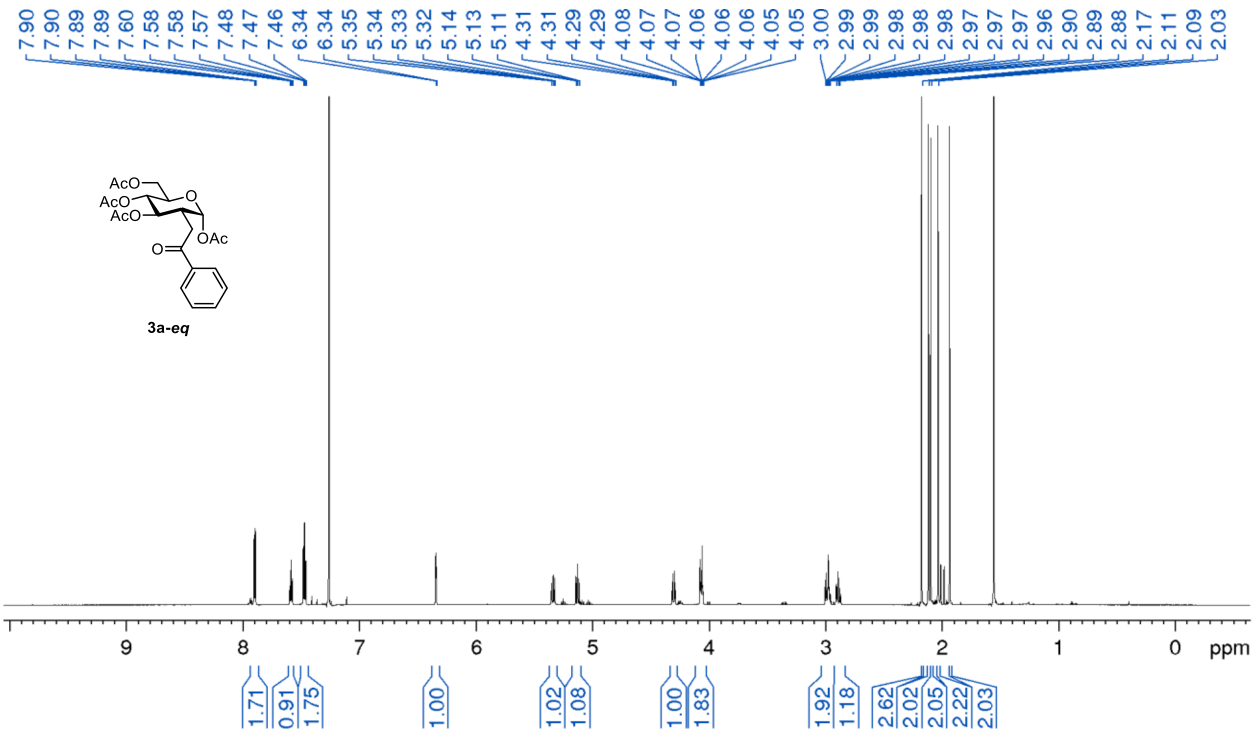

^{13}C NMR (175 MHz, CDCl$_3$, 25 ºC) of (3a-*eq*)

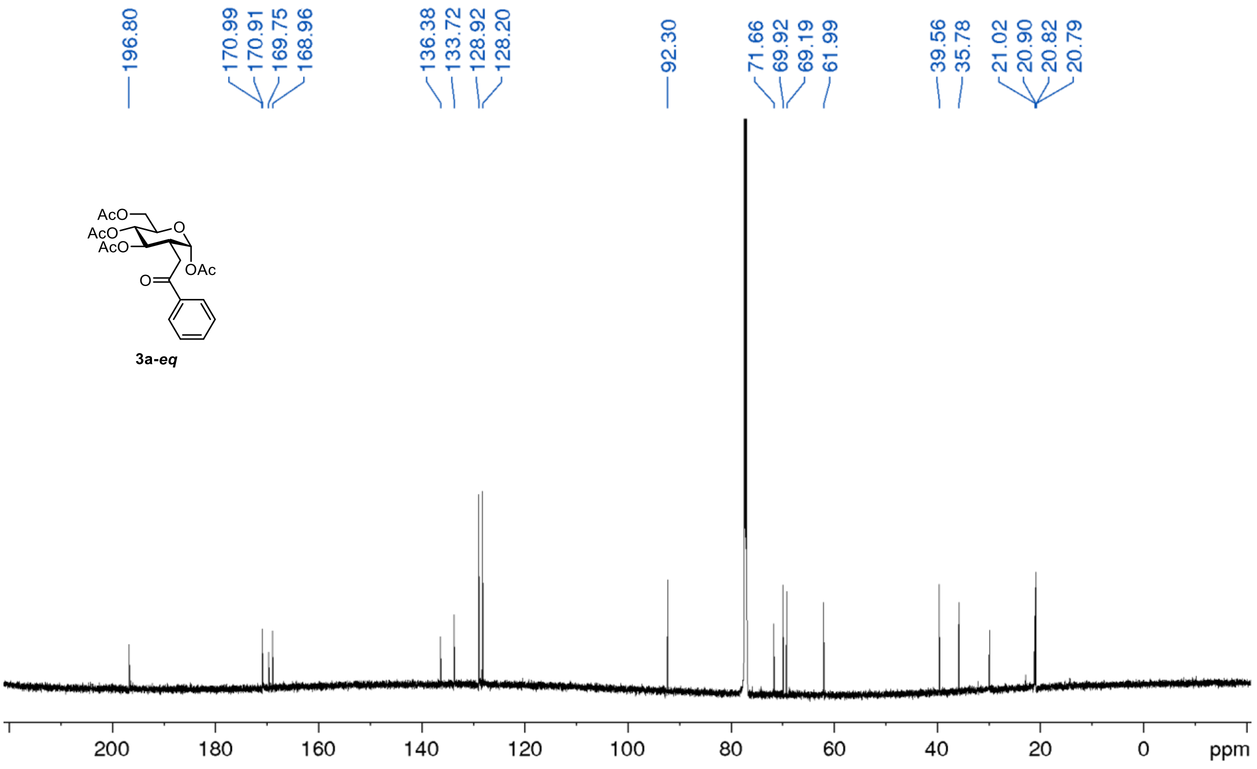

^{1}H NMR (700 MHz, CDCl$_3$, 25 ºC) of (3b-*ax*)

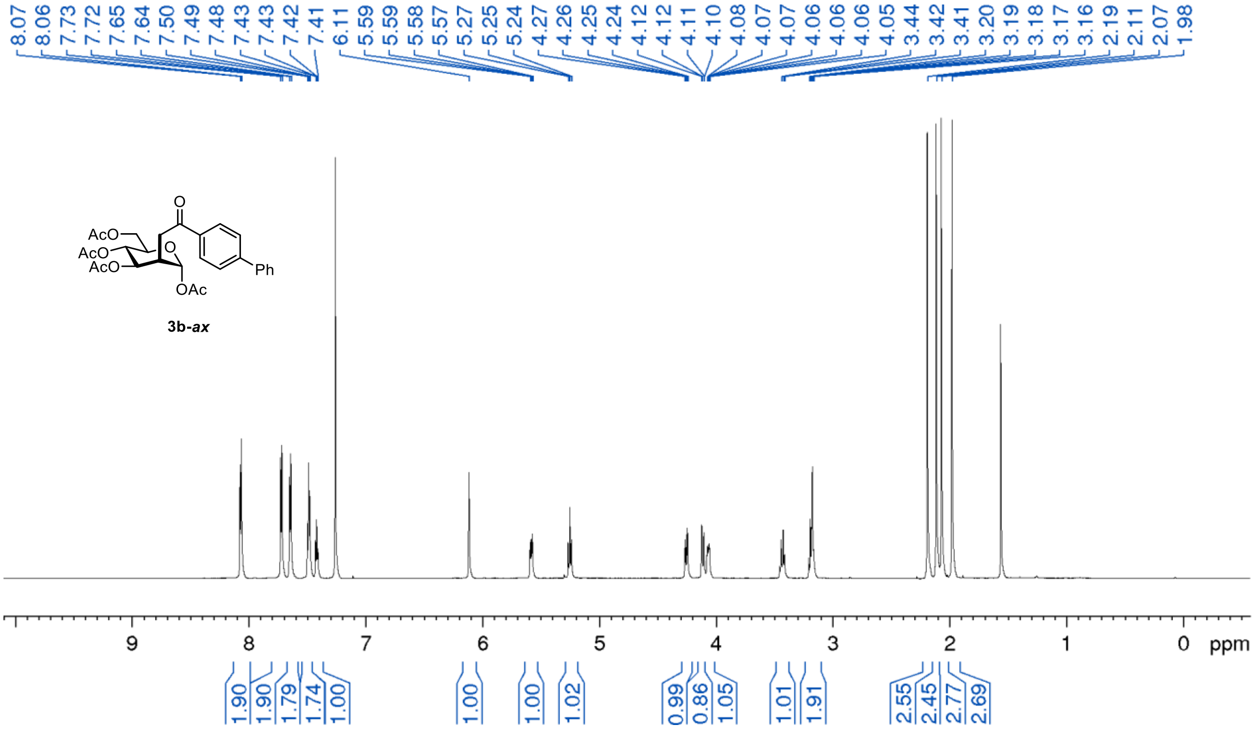

^{13}C NMR (175 MHz, CDCl$_3$, 25 ºC) of (3b-*ax*)

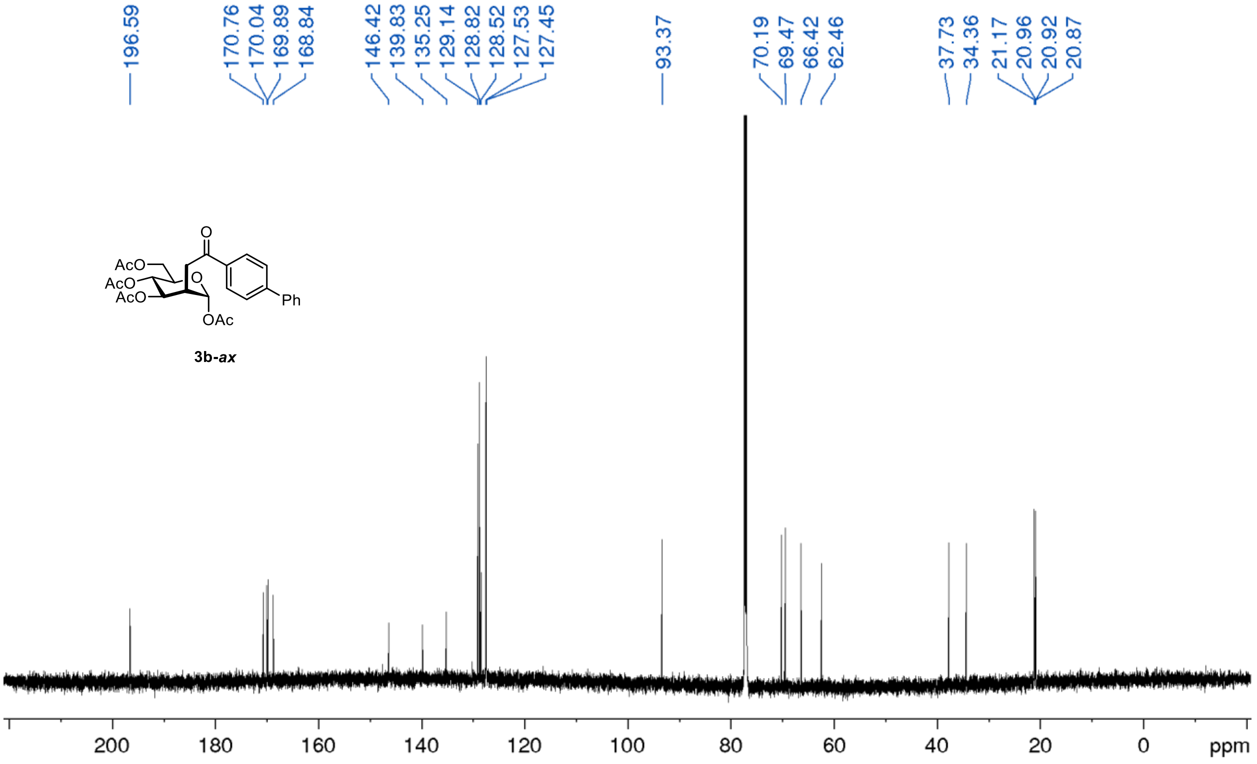

^{1}H NMR (700 MHz, $CDCl_3$, 25 °C) of (3b-*eq*)

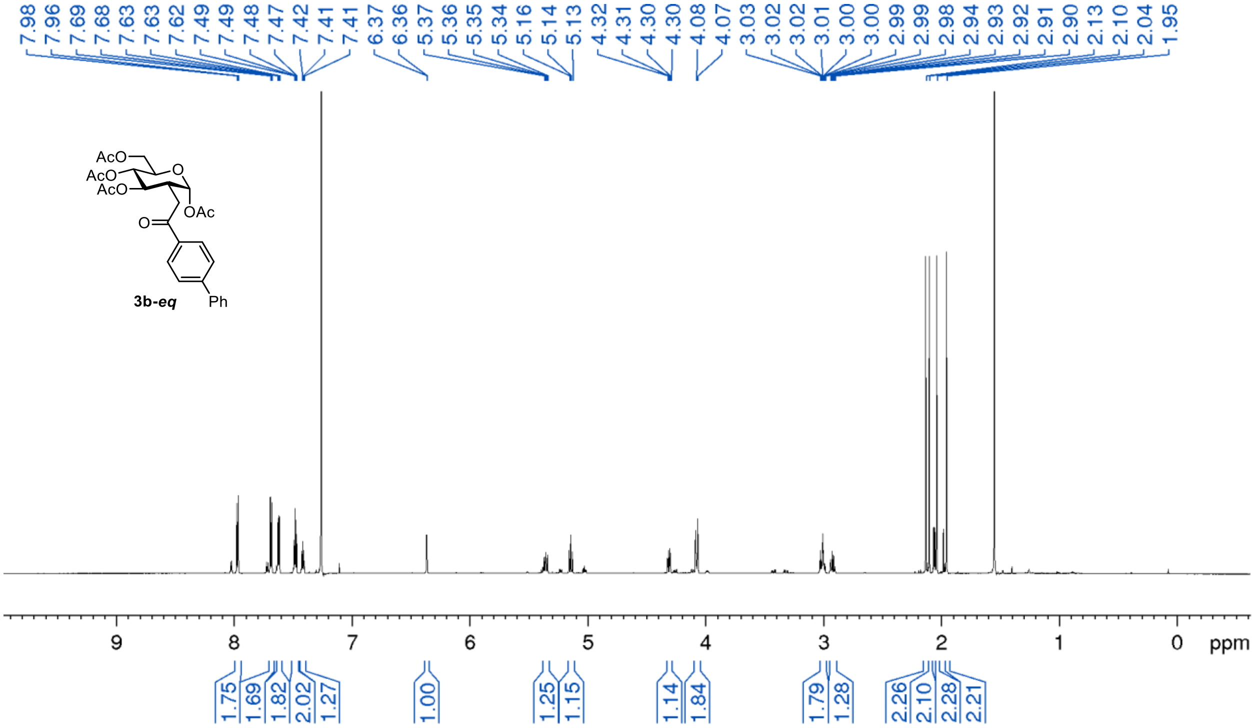

^{13}C NMR (175 MHz, $CDCl_3$, 25 °C) of (3b-*eq*)

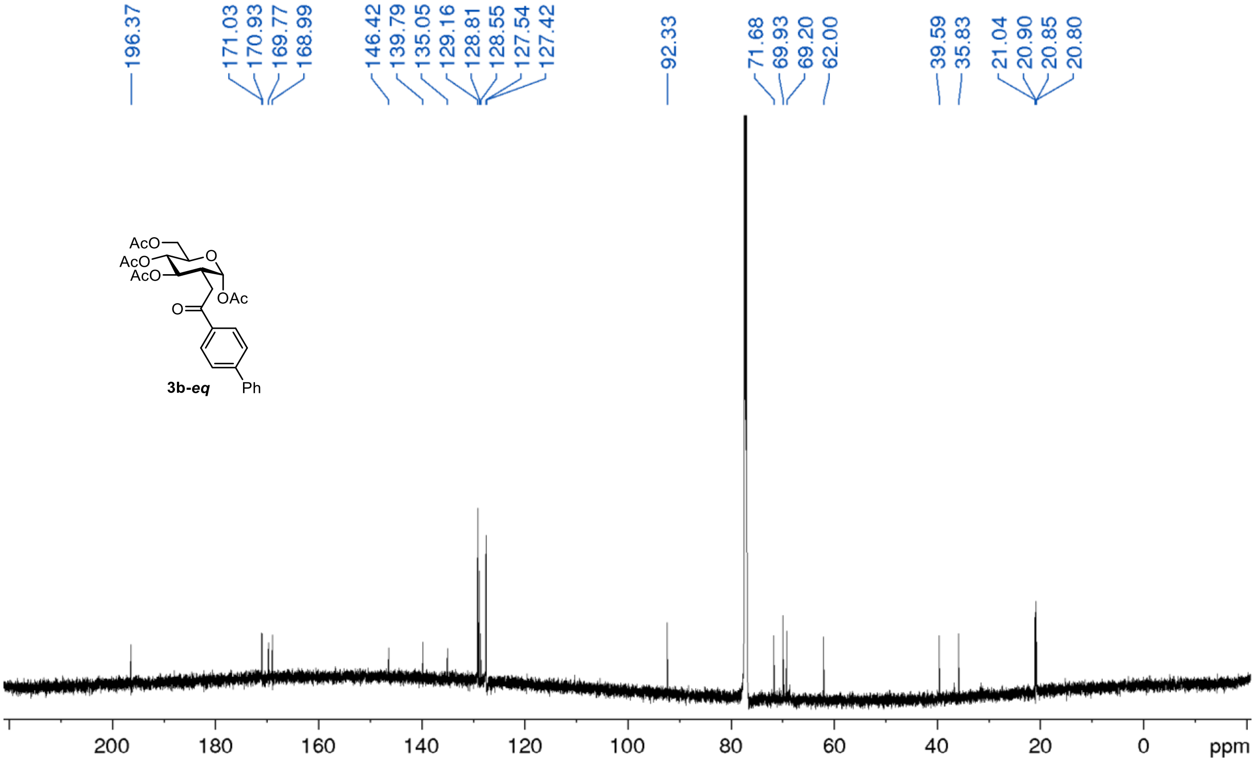

^{1}H NMR (700 MHz, $CDCl_3$, 25 °C) of (3c-*ax*)

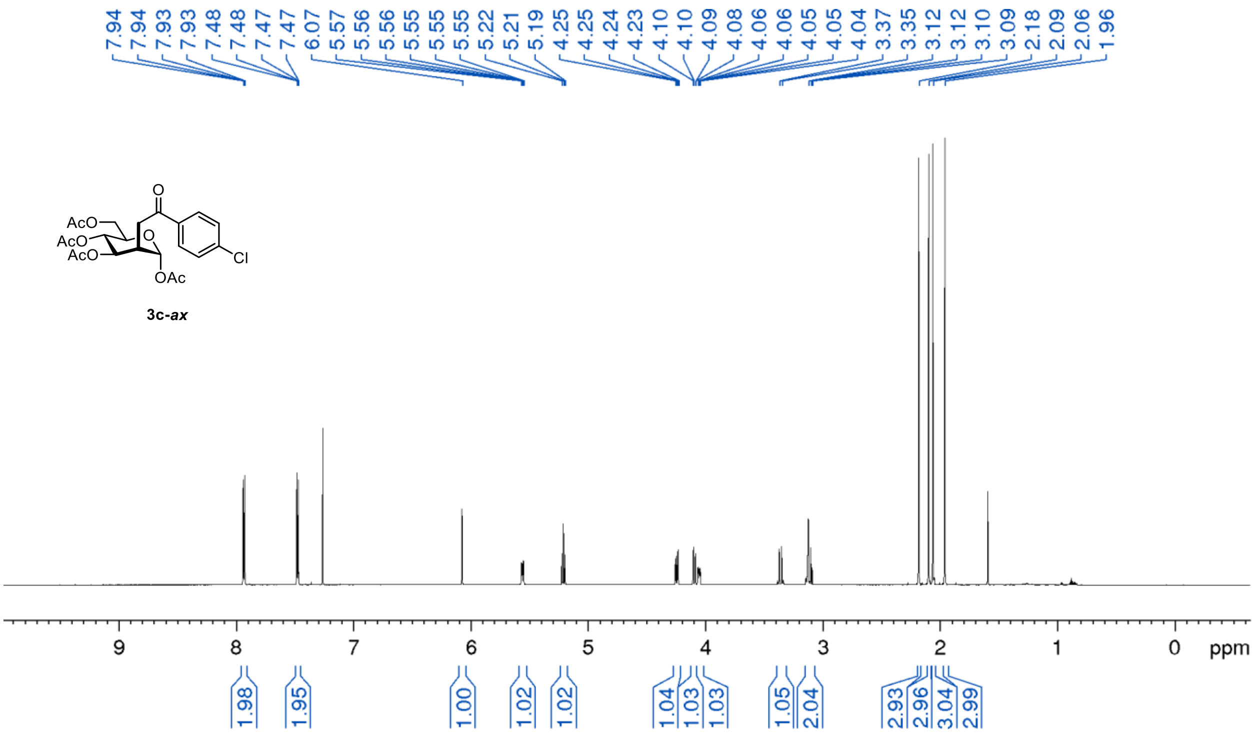

^{13}C NMR (175 MHz, $CDCl_3$, 25 °C) of (3c-*ax*)

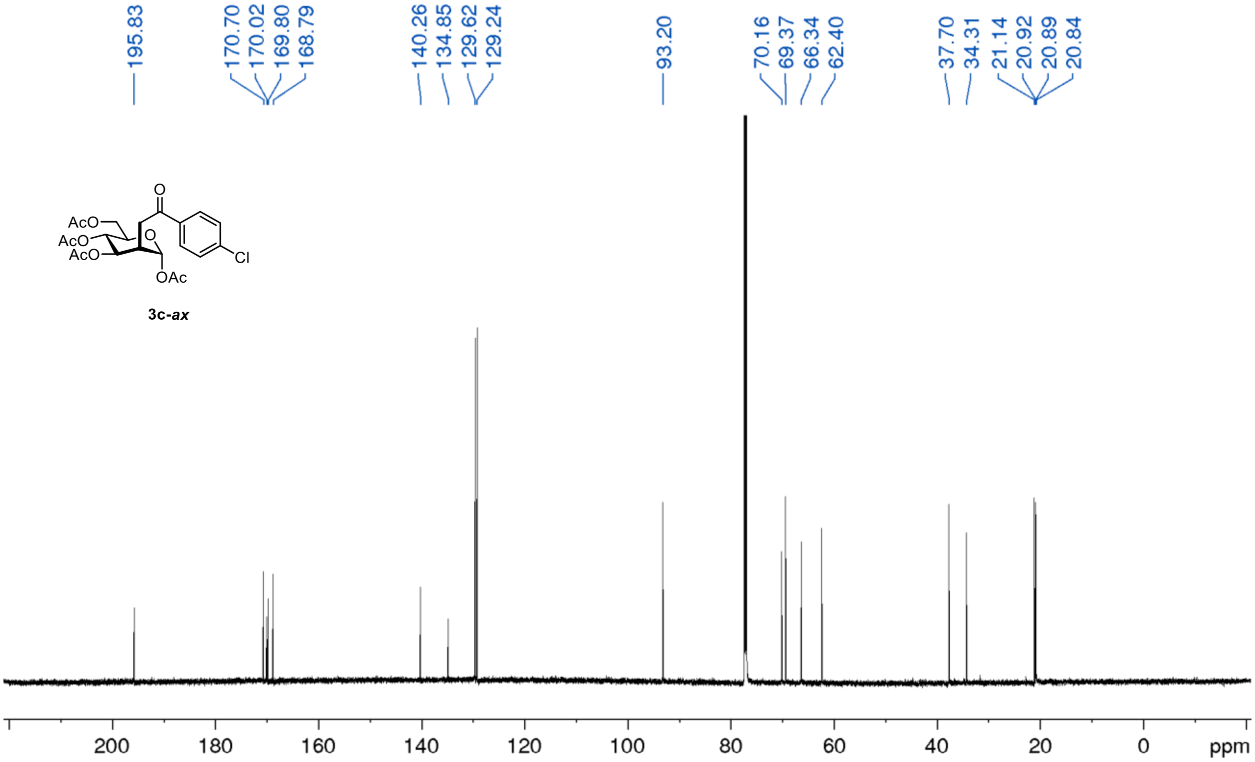

^{1}H NMR (700 MHz, CDCl$_3$, 25 °C) of (3c-*eq*)

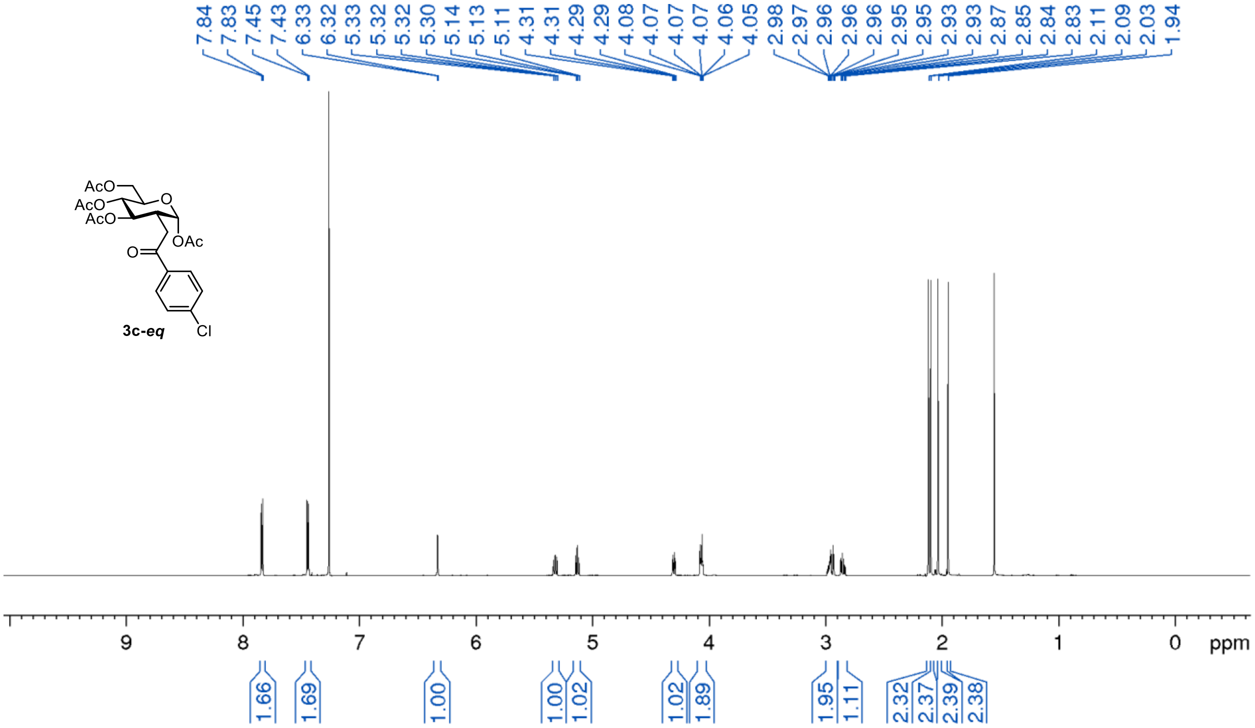

^{13}C NMR (175 MHz, CDCl$_3$, 25 °C) of (3c-*eq*)

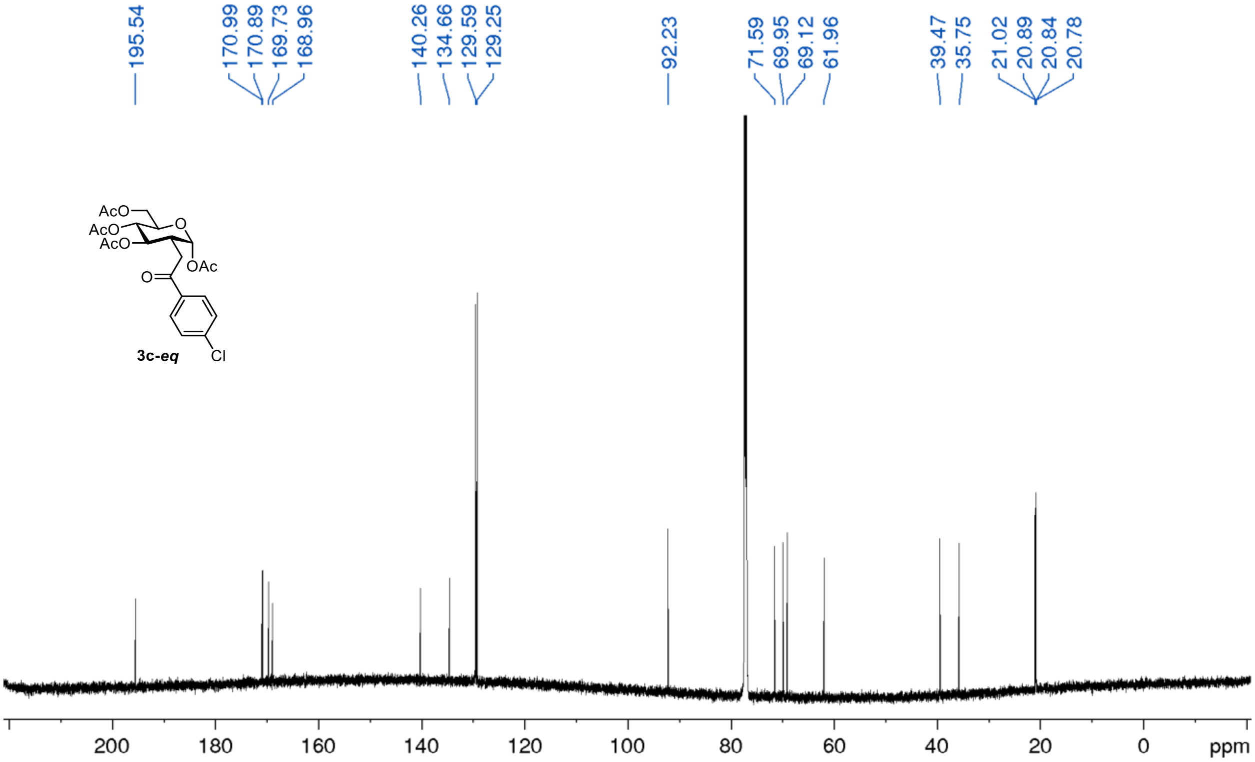

^{1}H NMR (500 MHz, $CDCl_3$, 25 °C) of (3d-*ax*)

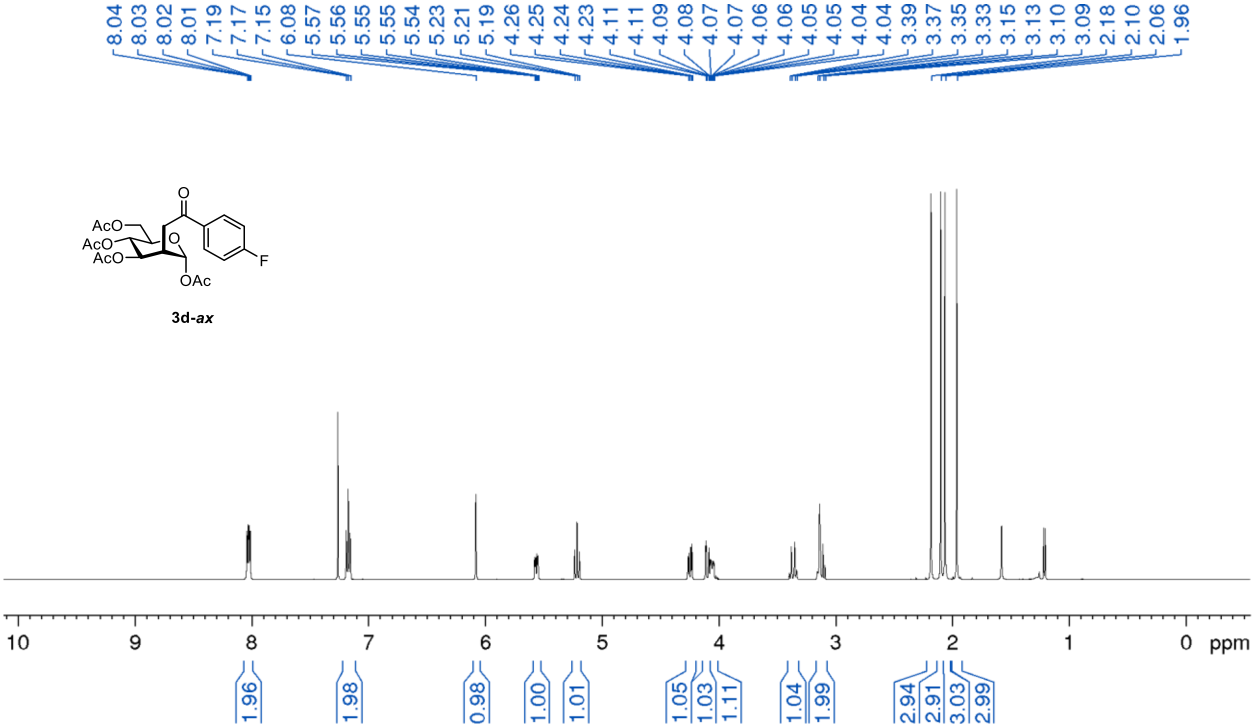

^{13}C NMR (125 MHz, $CDCl_3$, 25 °C) of (3d-*ax*)

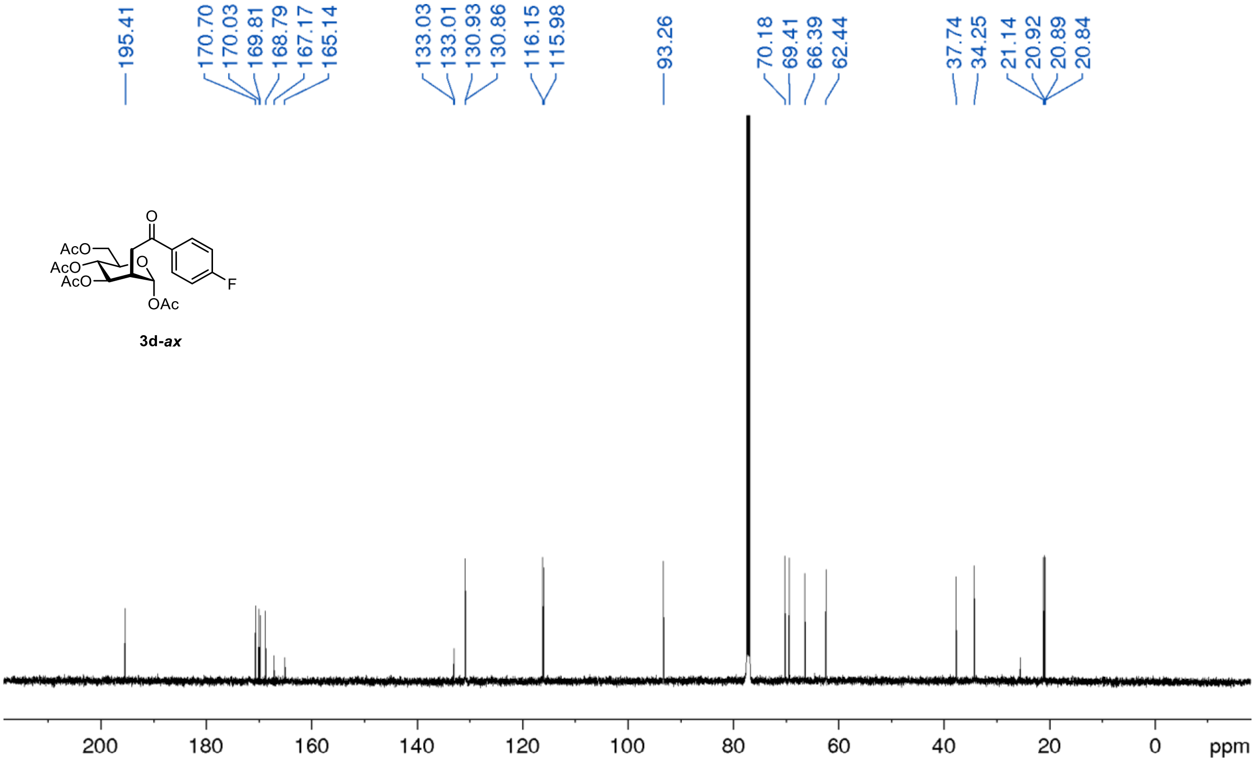

^{19}F NMR (700 MHz, CDCl$_3$, 25 ºC) of (3d-*ax*)

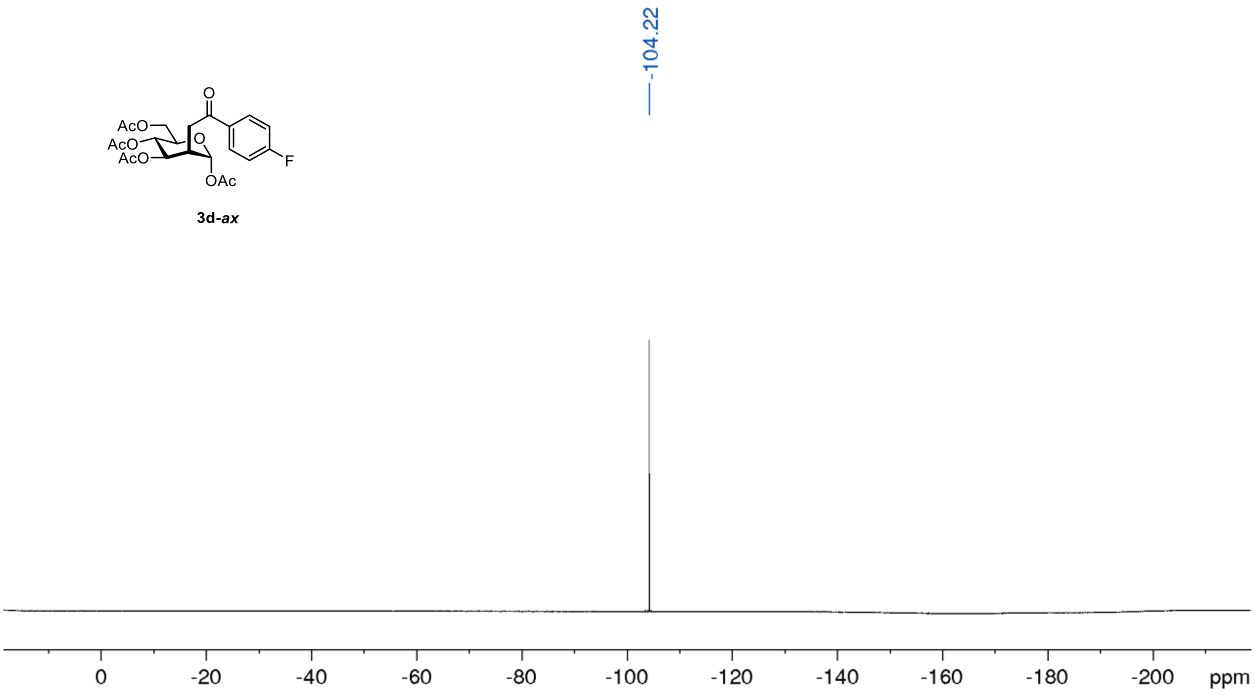

^{1}H NMR (500 MHz, CDCl$_3$, 25 ºC) of (3d-*eq*)

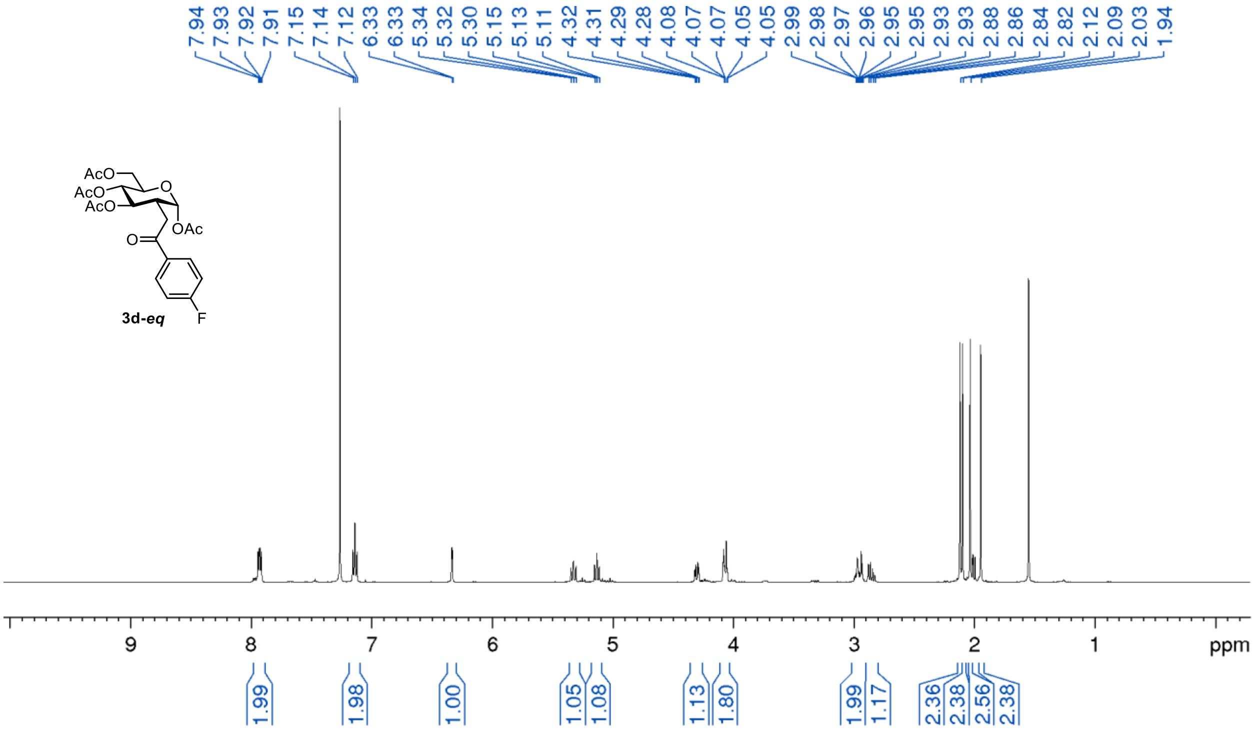

^{13}C NMR (125 MHz, $CDCl_3$, 25 °C) of (3d-*eq*)

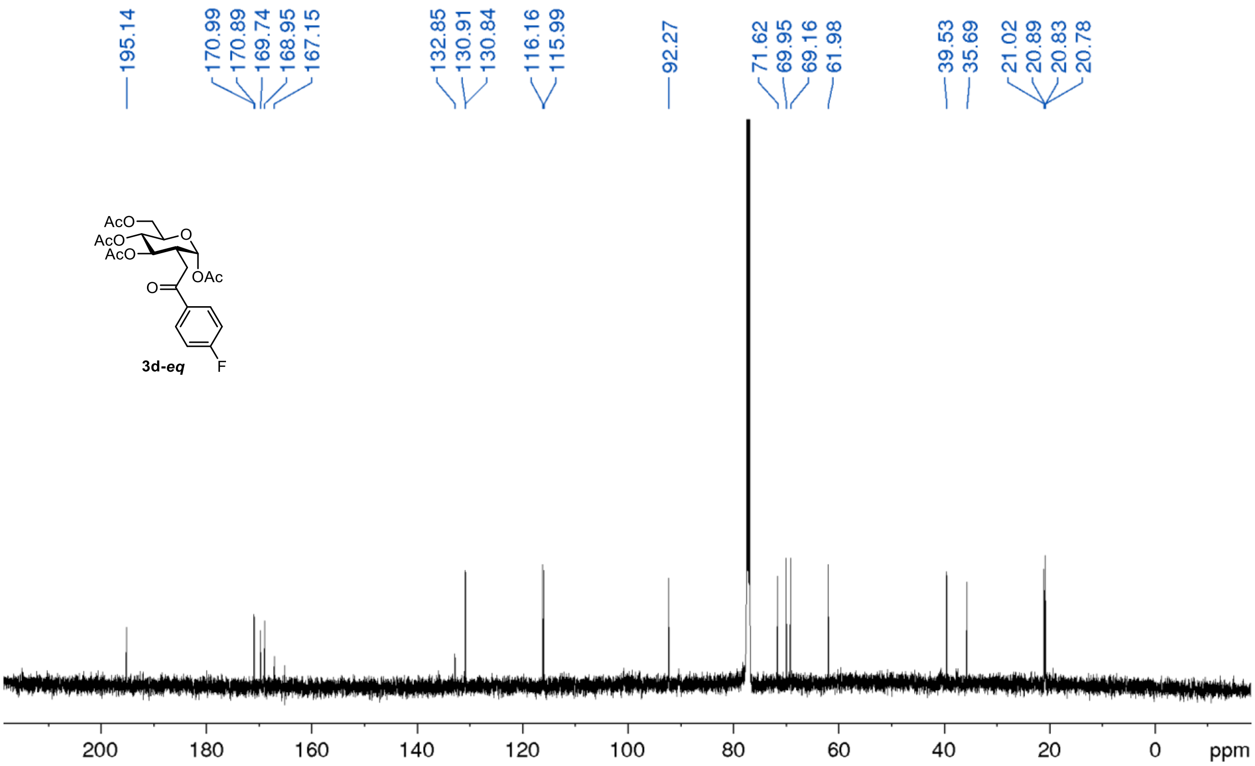

^{19}F NMR (700 MHz, $CDCl_3$, 25 °C) of (3d-*eq*)

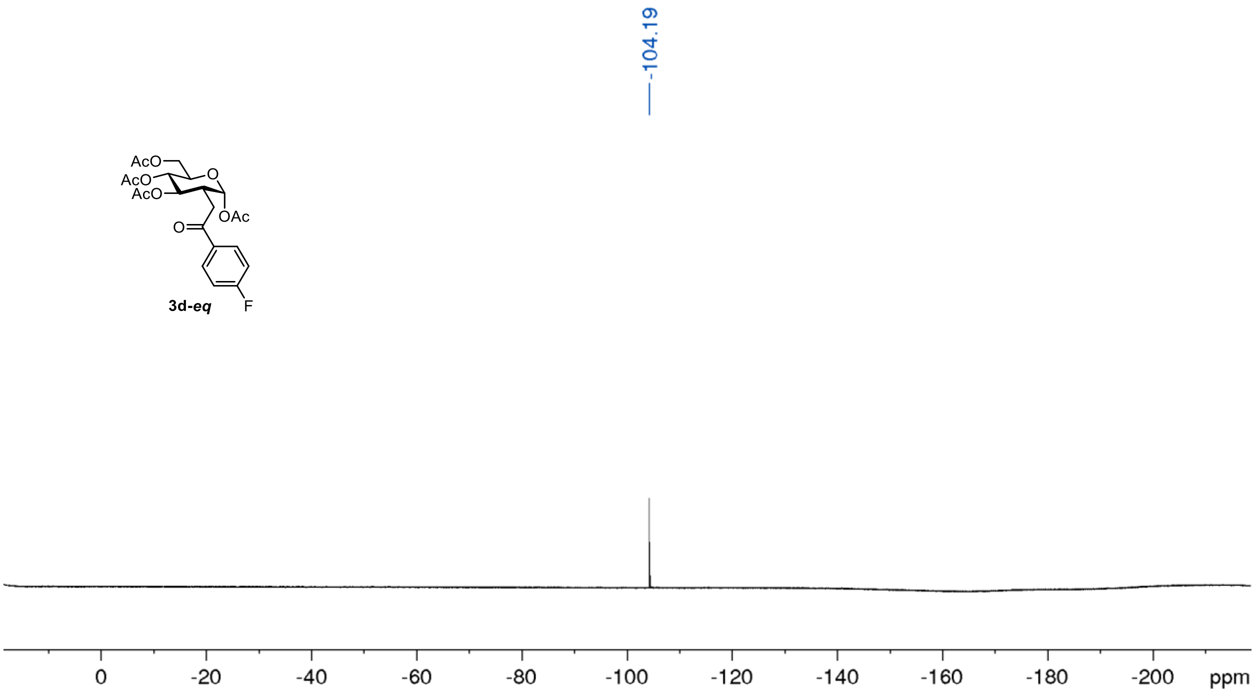

^{1}H NMR (500 MHz, $CDCl_3$, 25 ºC) of (3e-*ax*)

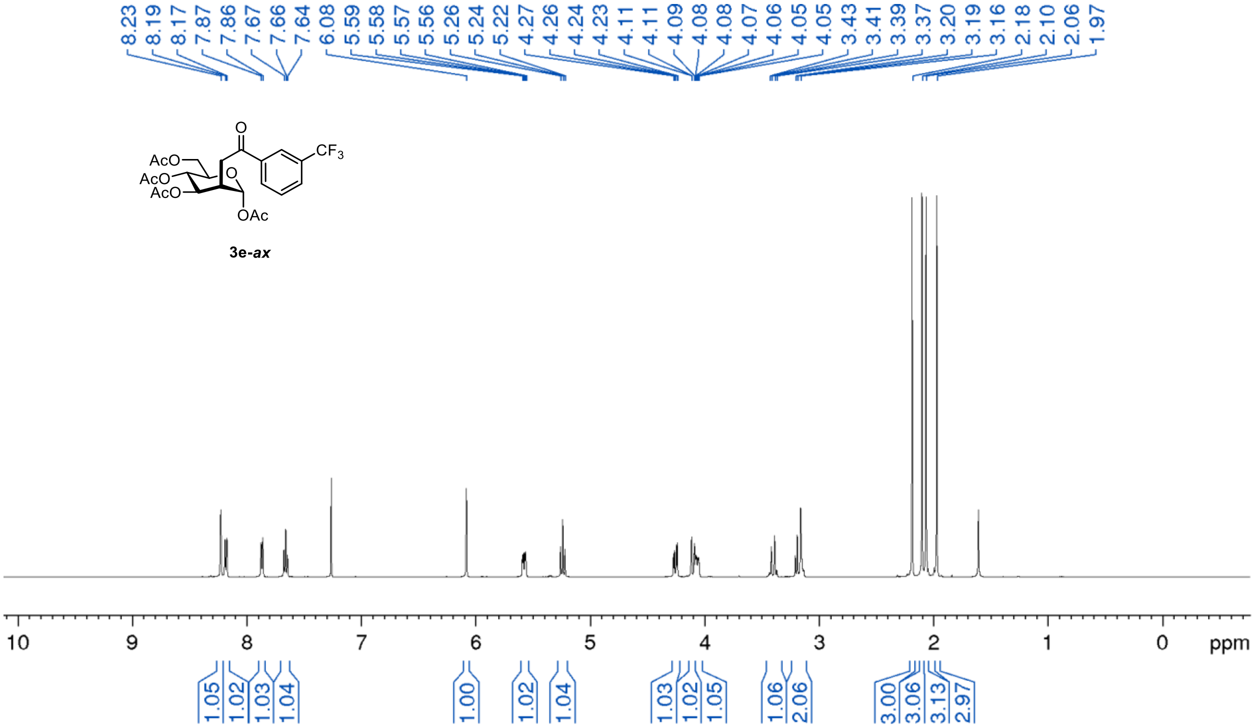

^{13}C NMR (125 MHz, $CDCl_3$, 25 ºC) of (3e-*ax*)

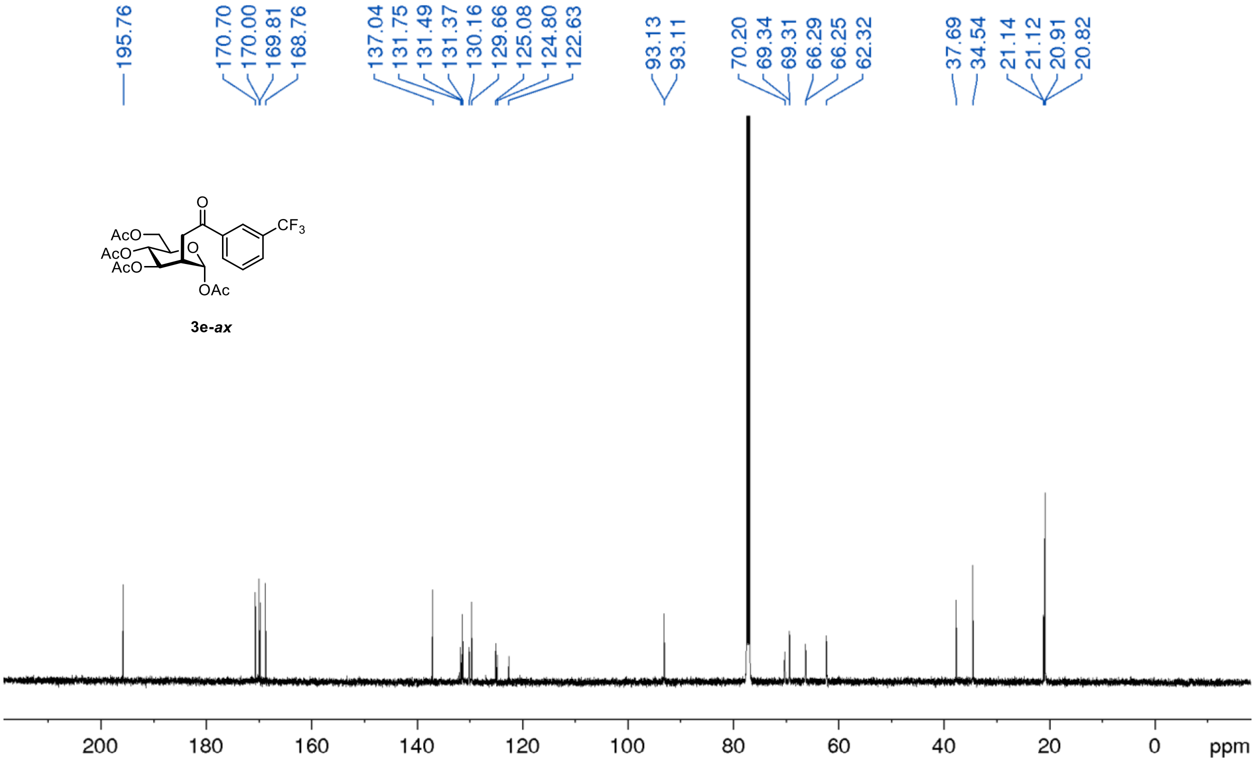

^{19}F NMR (700 MHz, $CDCl_3$, 25 °C) of (3e-*ax*)

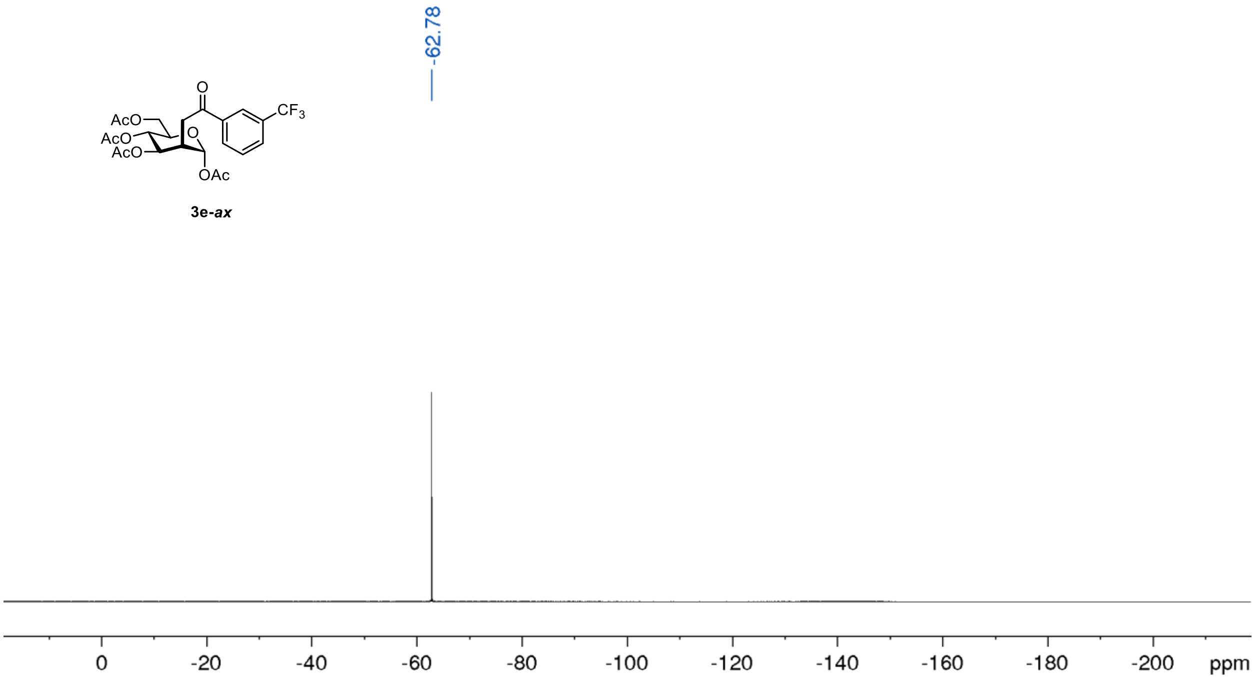

^{1}H NMR (700 MHz, $CDCl_3$, 25 °C) of (3f-*ax*)

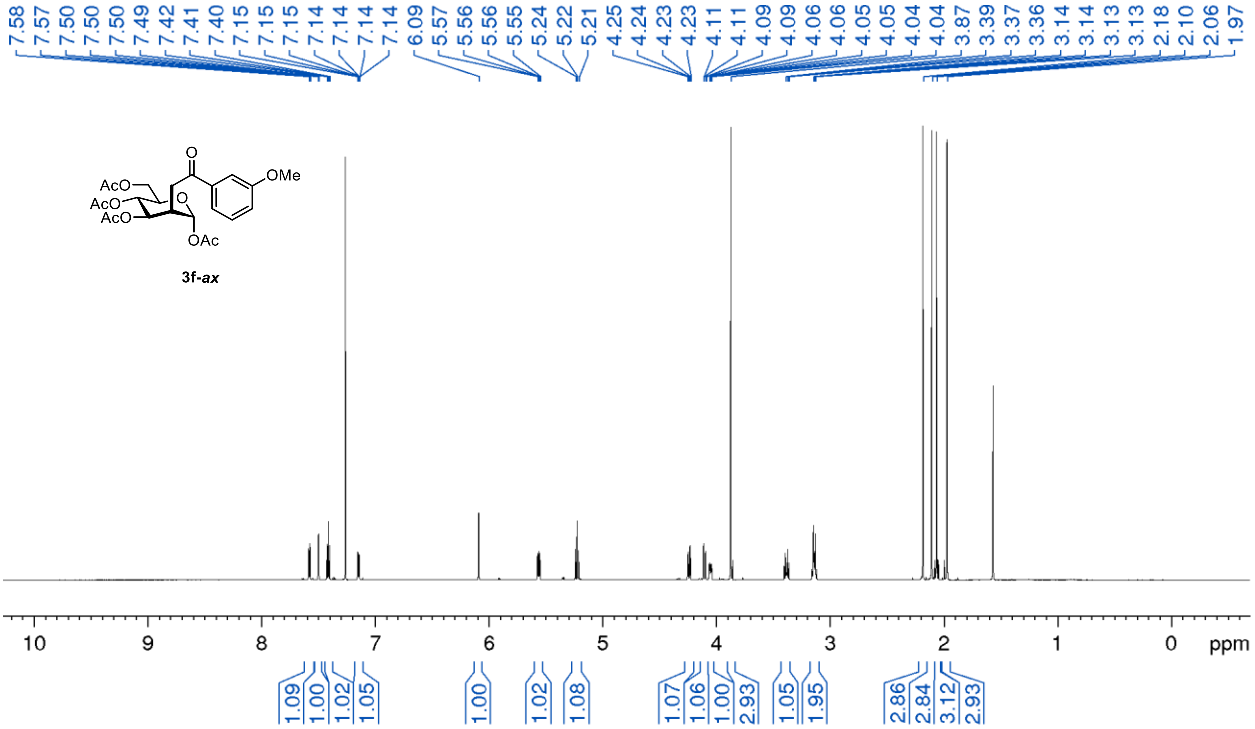

^{13}C NMR (175 MHz, $CDCl_3$, 25 ºC) of (3f-*ax*)

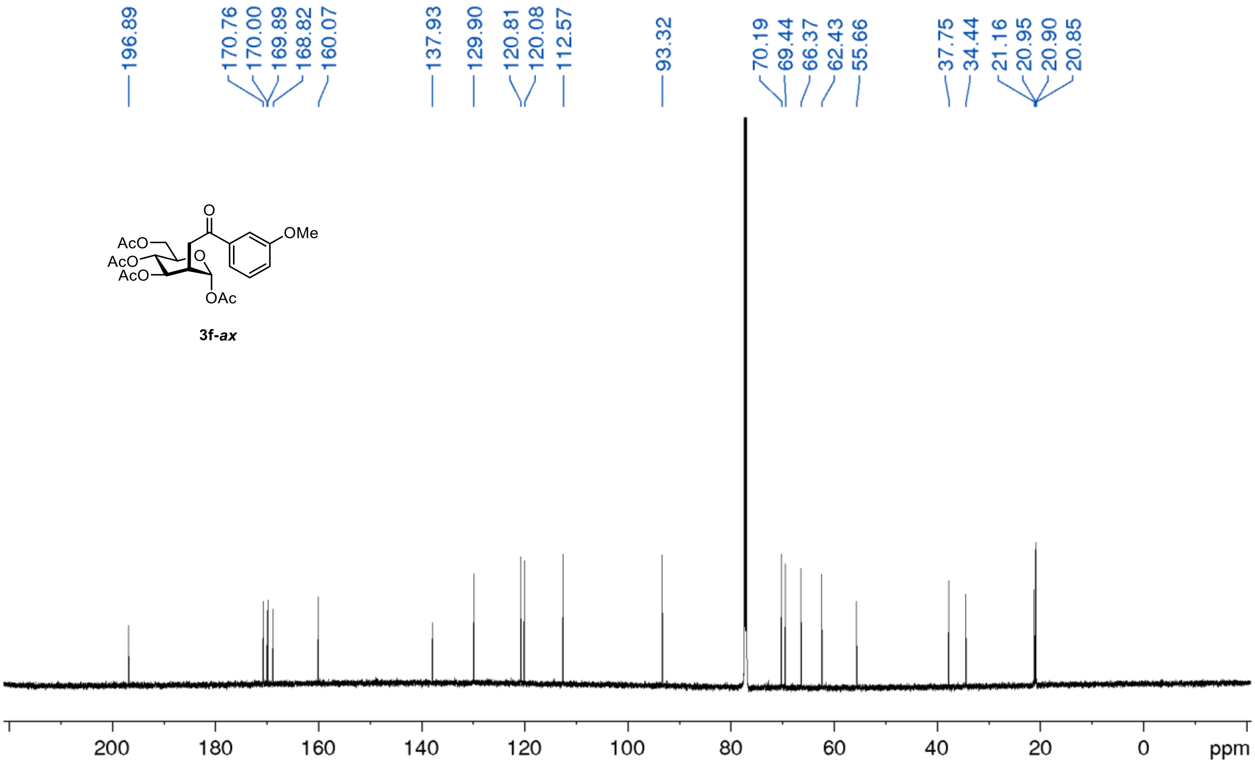

^{1}H NMR (700 MHz, $CDCl_3$, 25 ºC) of (3f-*eq*)

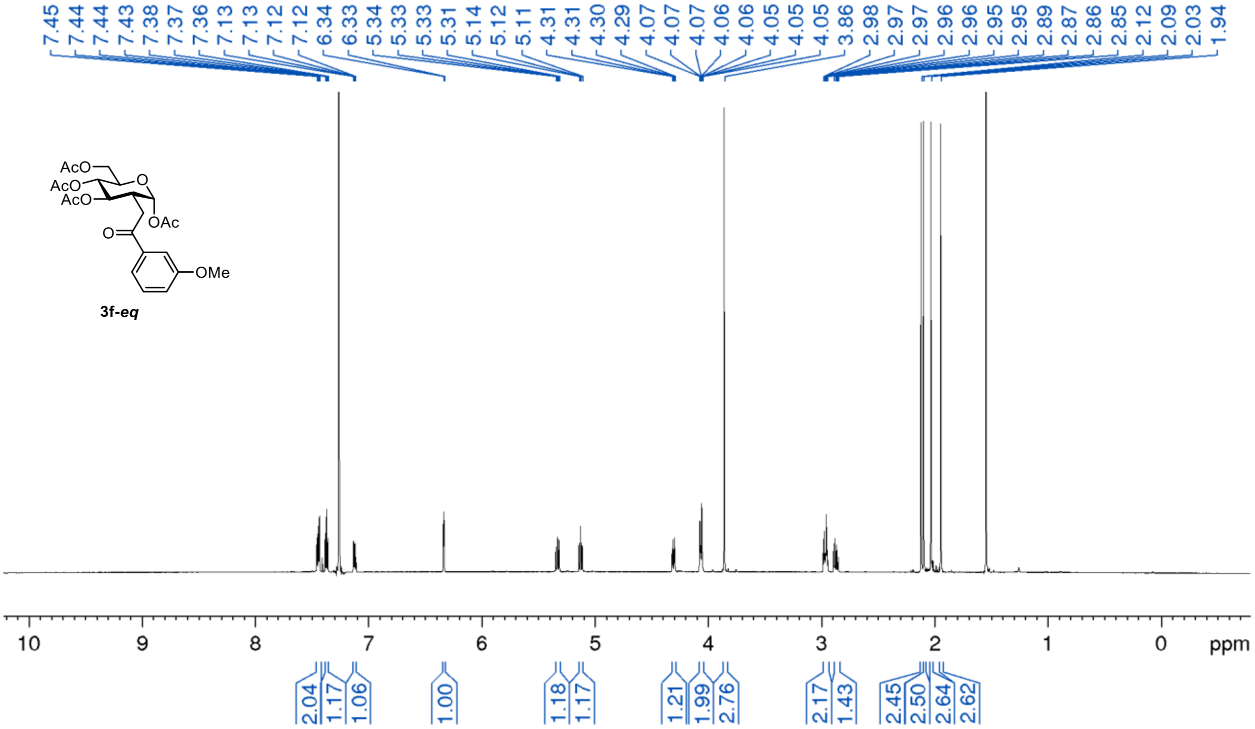

^{13}C NMR (175 MHz, CDCl₃, 25 ºC) of (3f-*eq*)

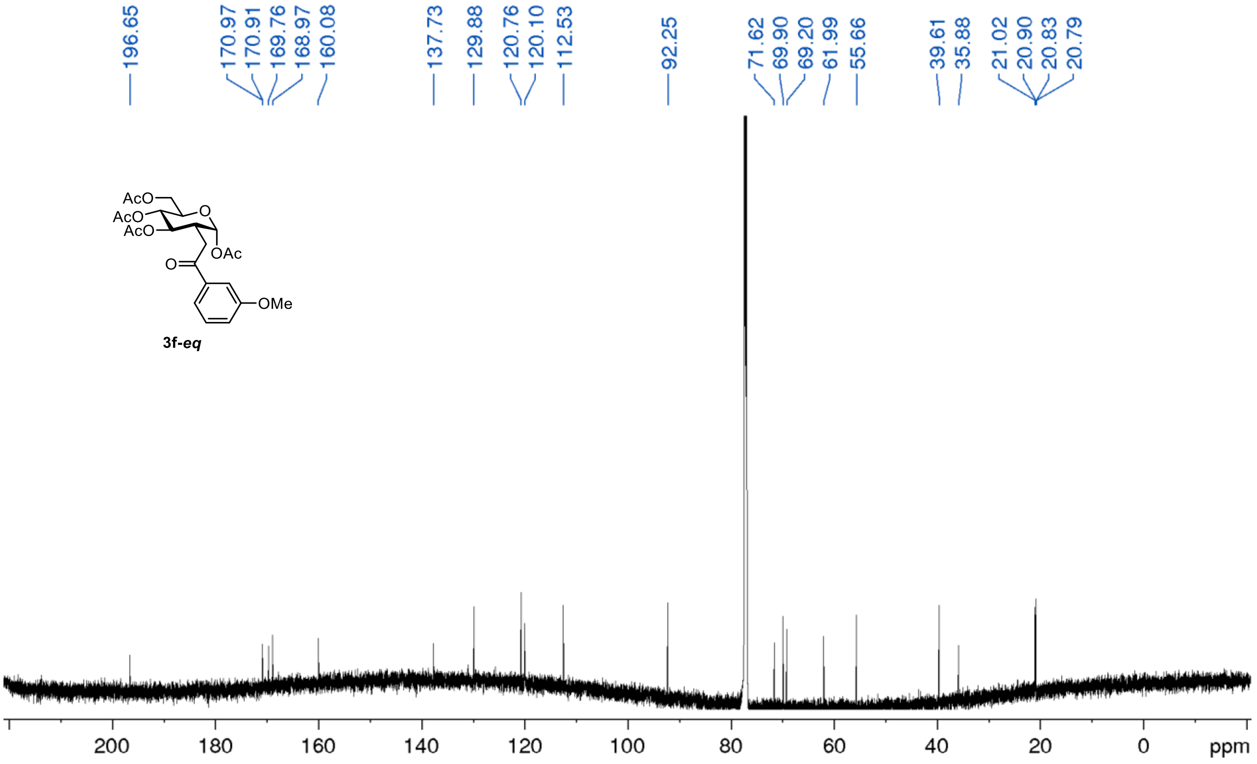

^{1}H NMR (500 MHz, CDCl₃, 25 ºC) of (3g-*ax*)

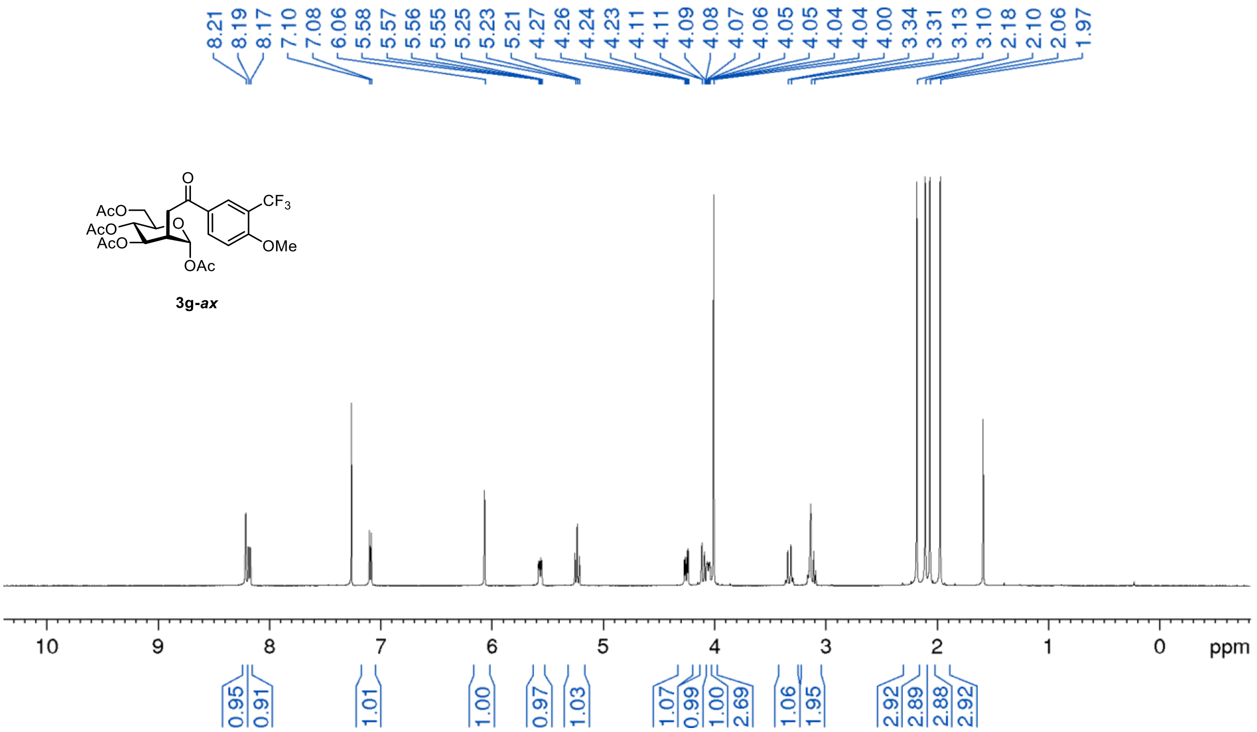

^{13}C NMR (125 MHz, $CDCl_3$, 25 ºC) of (3g-*ax*)

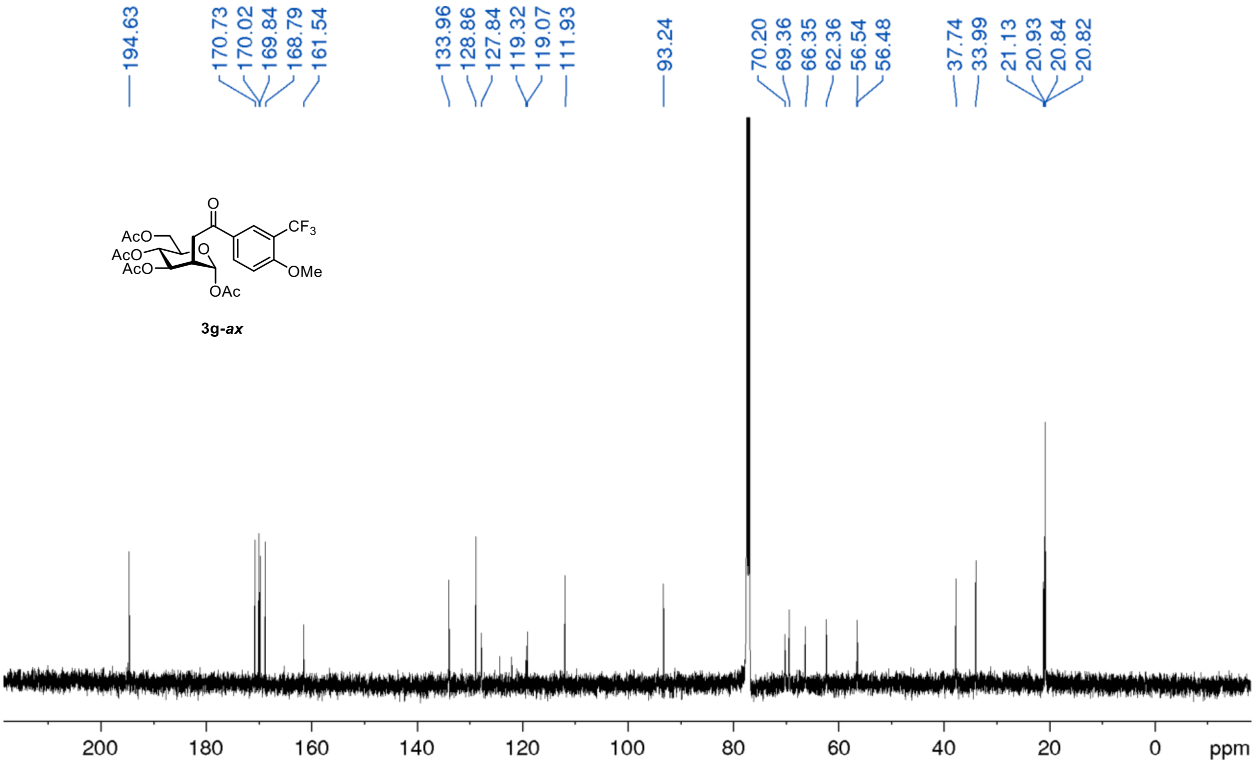

^{19}F NMR (700 MHz, $CDCl_3$, 25 ºC) of (3g-*ax*)

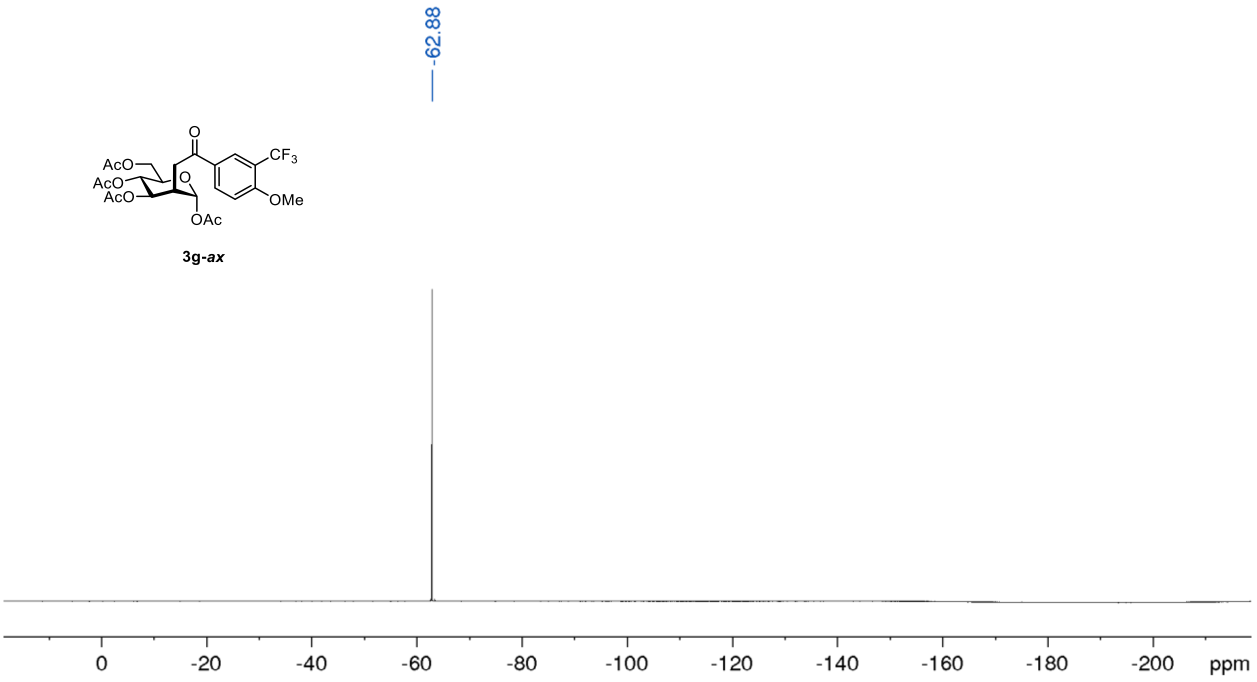

^{1}H NMR (500 MHz, $CDCl_3$, 25 °C) of (3h-*ax*)

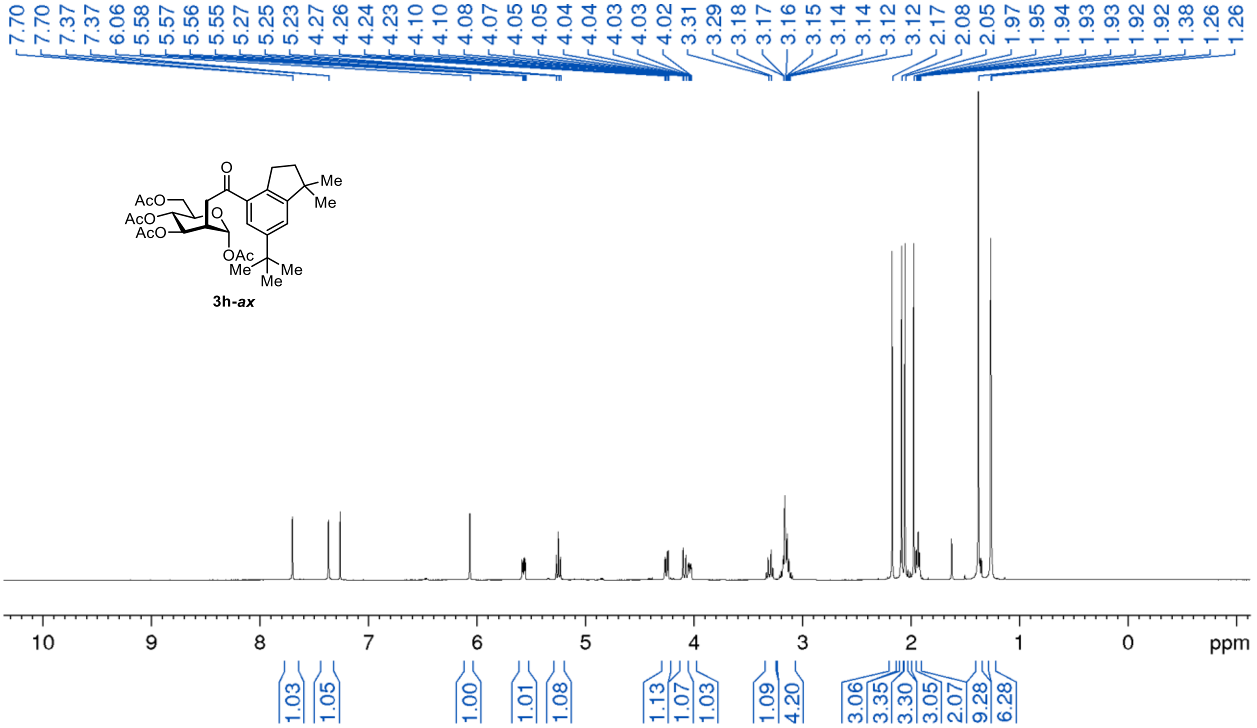

^{13}C NMR (125 MHz, $CDCl_3$, 25 °C) of (3h-*ax*)

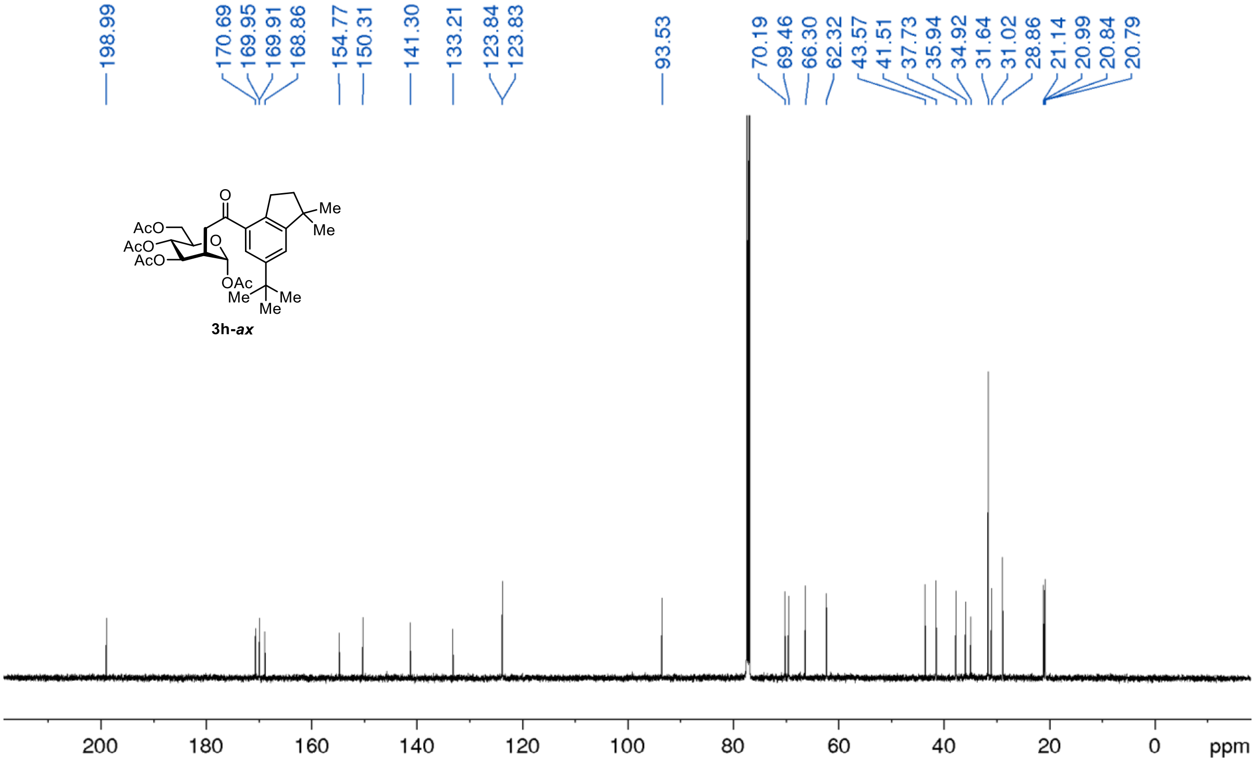

^{1}H NMR (500 MHz, CDCl$_3$, 25 °C) of (3h-*eq*)

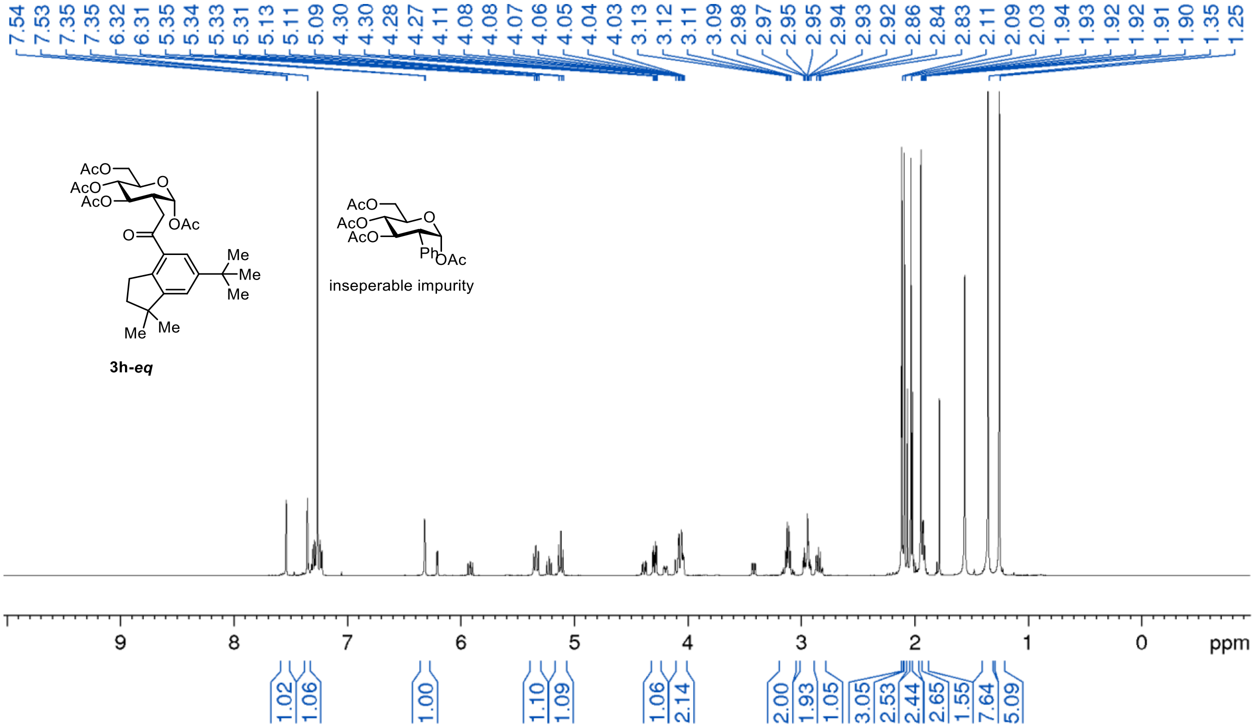

^{13}C NMR (125 MHz, CDCl$_3$, 25 °C) of (3h-*eq*)

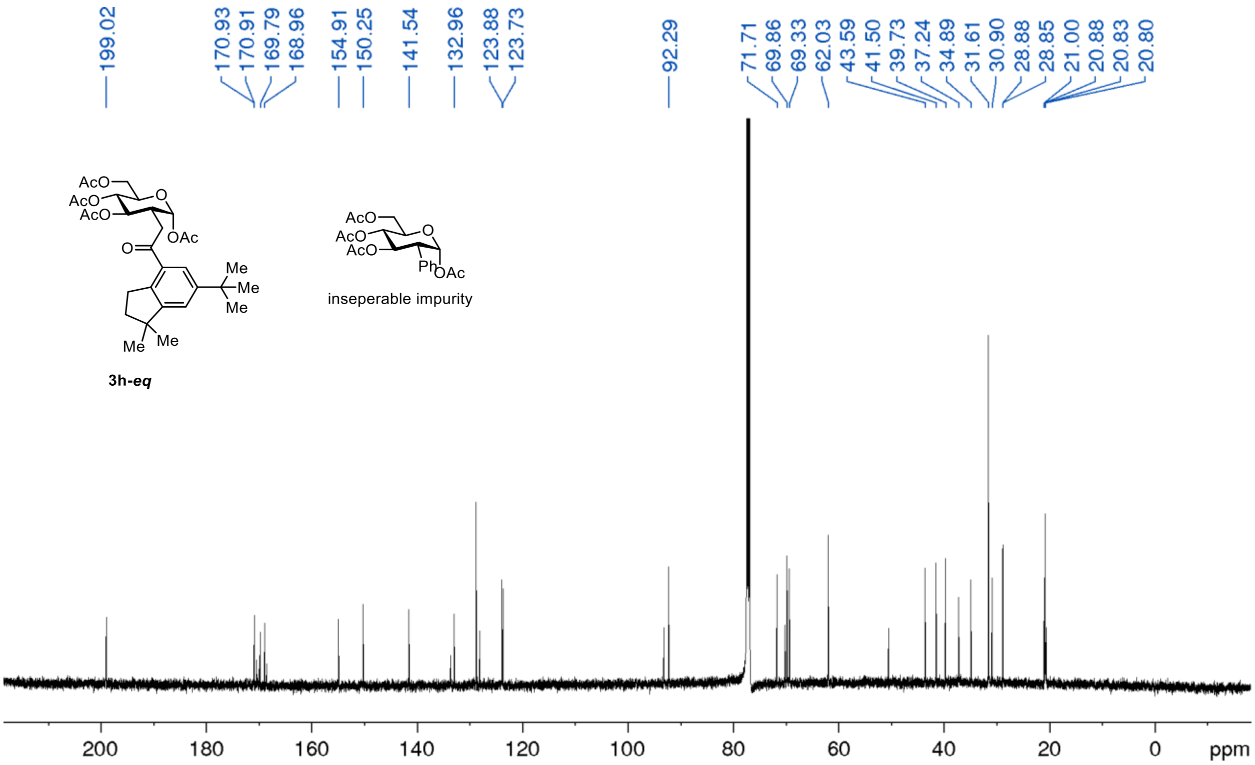

^{1}H NMR (700 MHz, CDCl$_3$, 25 °C) of (3i-*ax*)

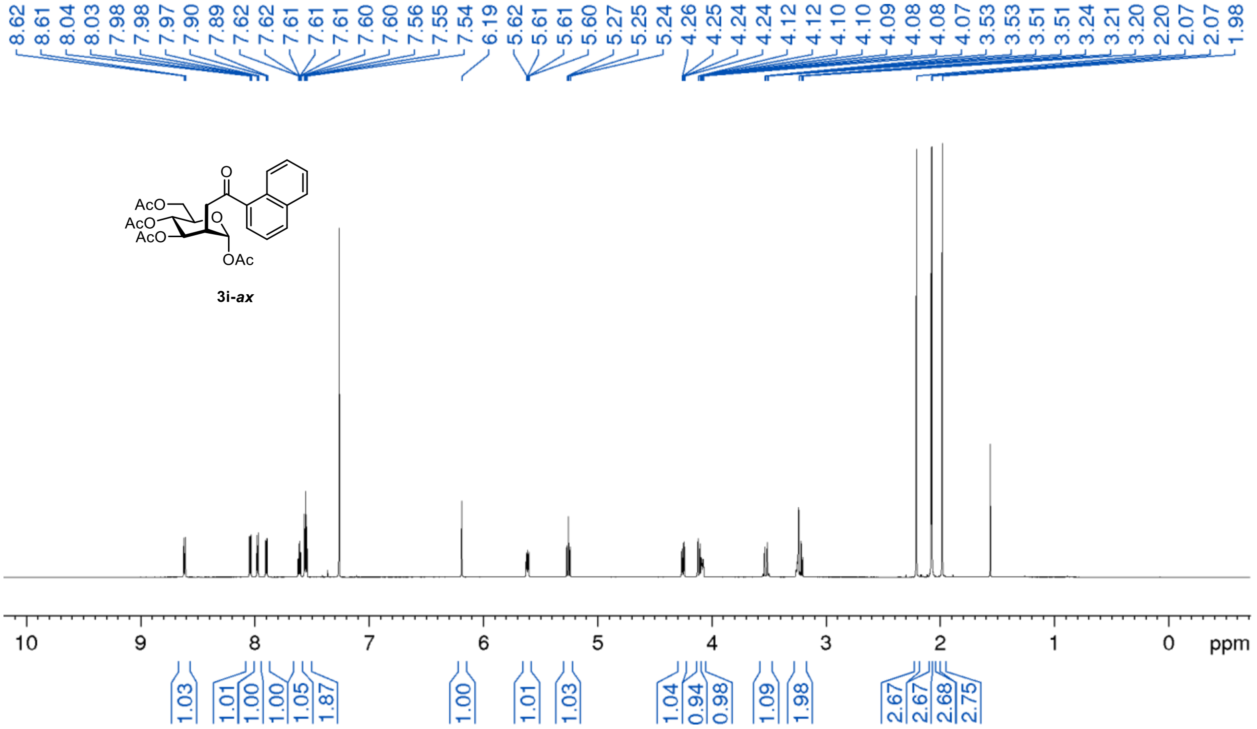

^{13}C NMR (175 MHz, CDCl$_3$, 25 °C) of (3i-*ax*)

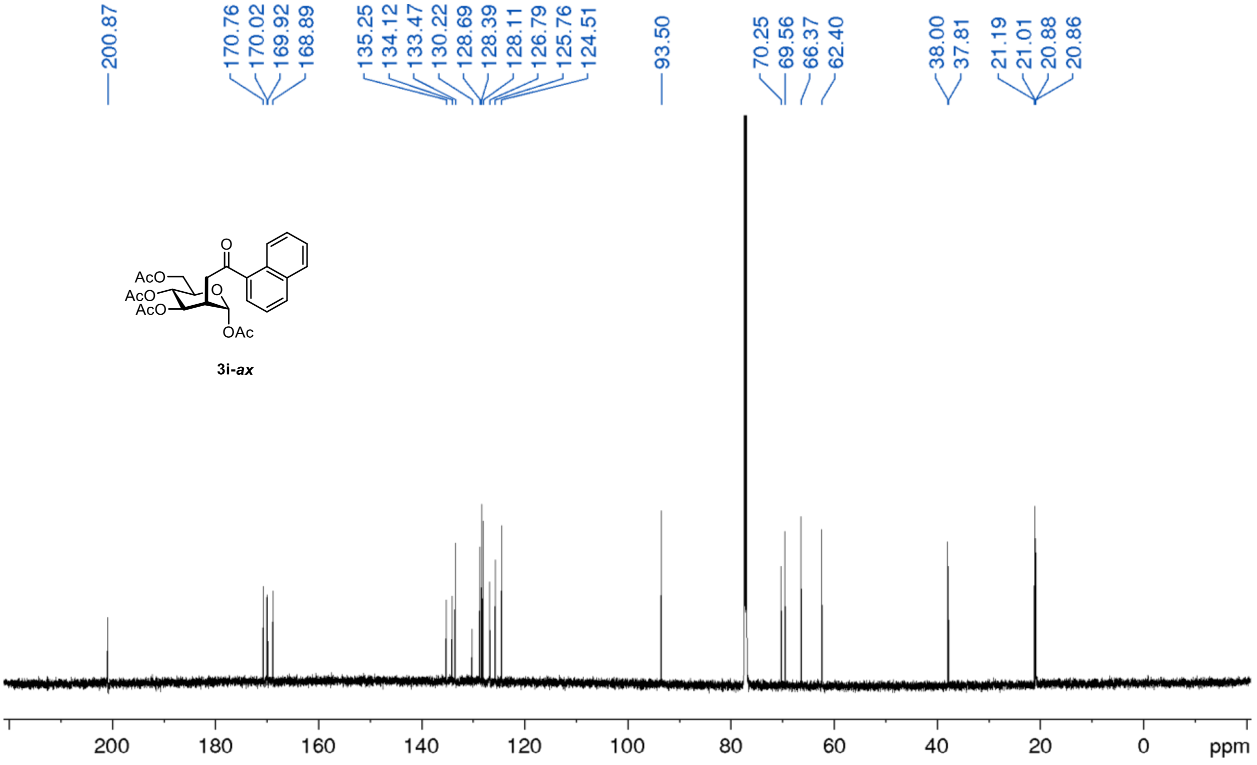

^{1}H NMR (700 MHz, $CDCl_3$, 25 °C) of (3i-*eq*)

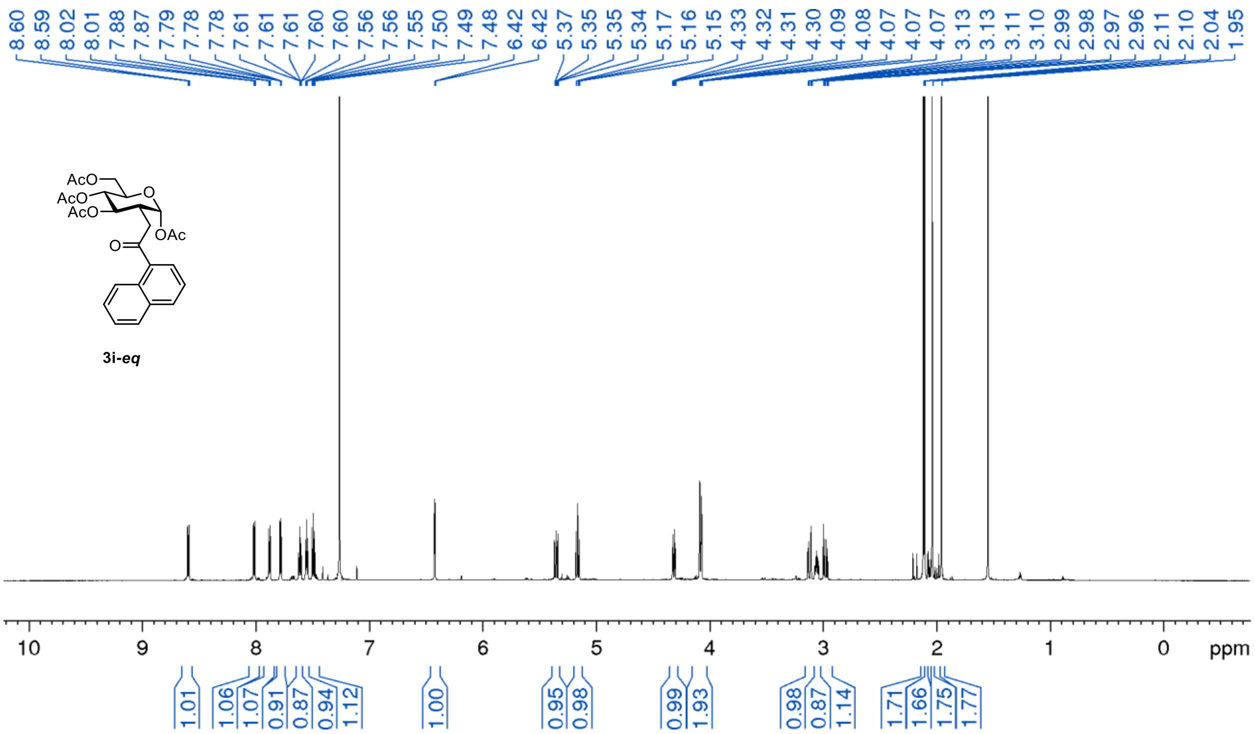

^{13}C NMR (125 MHz, $CDCl_3$, 25 °C) of (3i-*eq*)

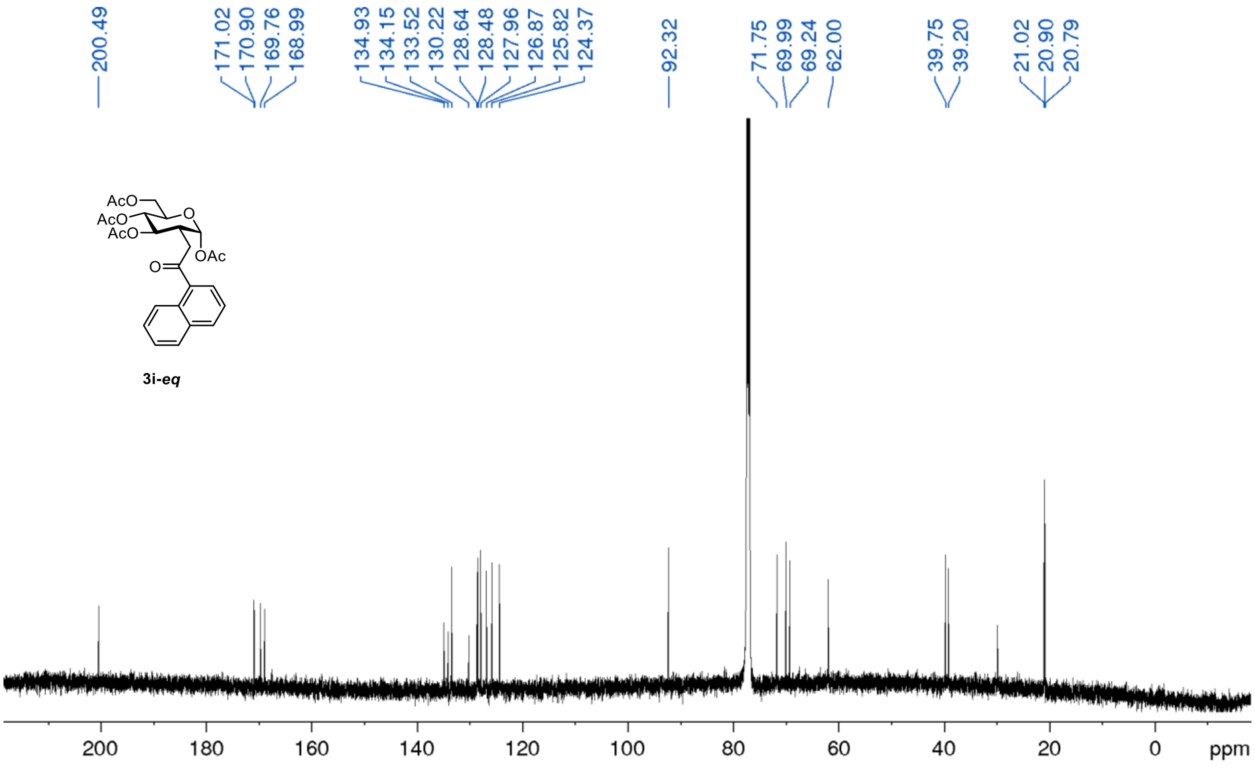

^{1}H NMR (500 MHz, CDCl$_3$, 25 °C) of (3j-*ax*)

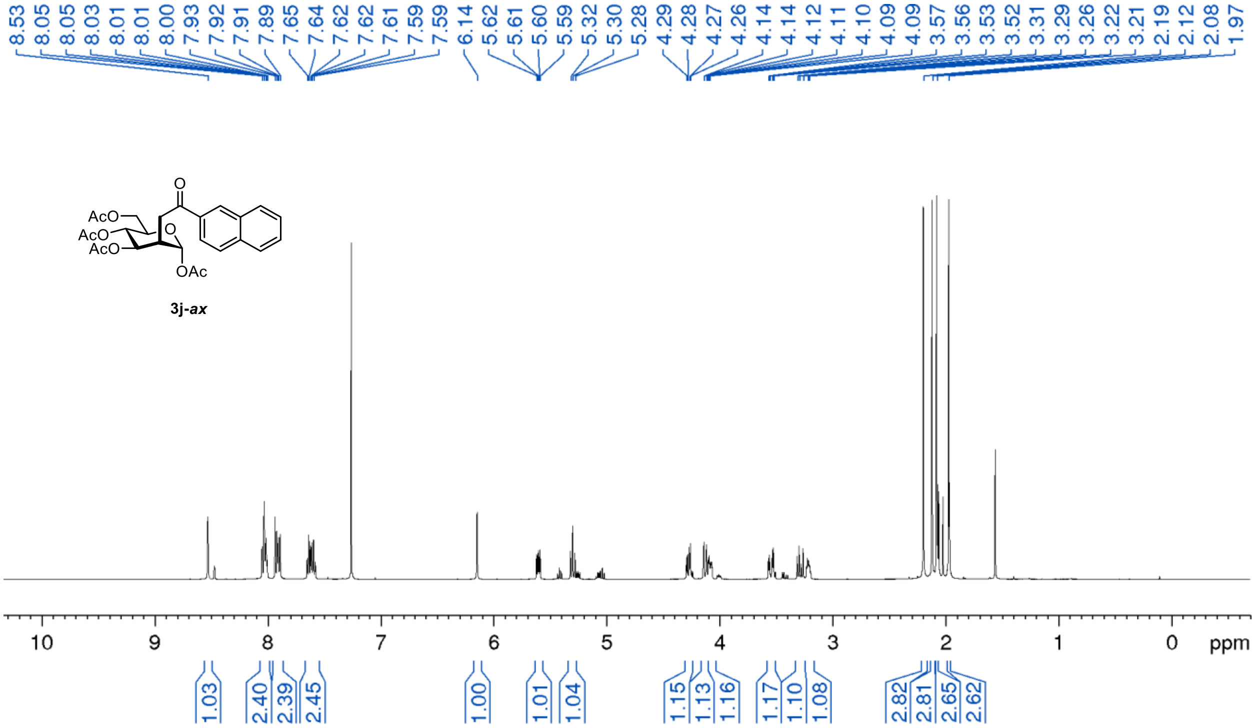

^{13}C NMR (125 MHz, CDCl$_3$, 25 °C) of (3j-*ax*)

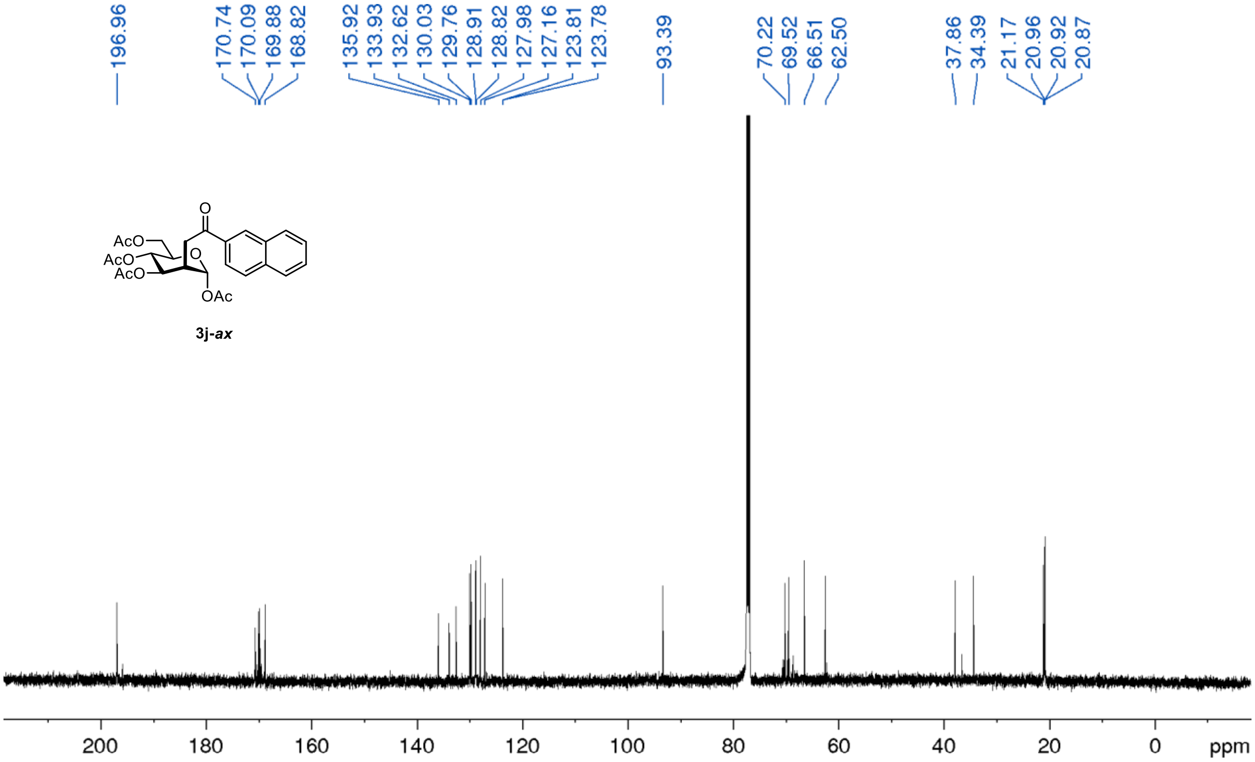

^{1}H NMR (500 MHz, $CDCl_3$, 25 °C) of (3j-*eq*)

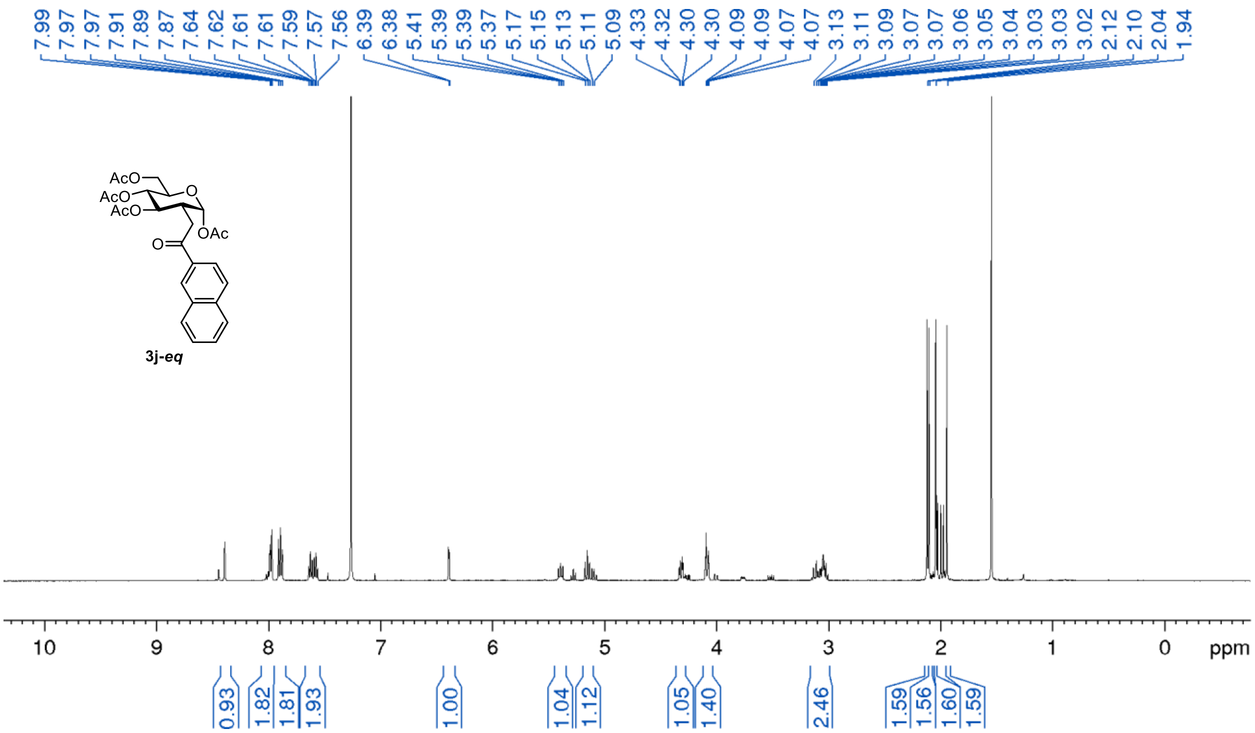

^{13}C NMR (175 MHz, $CDCl_3$, 25 °C) of (3j-*eq*)

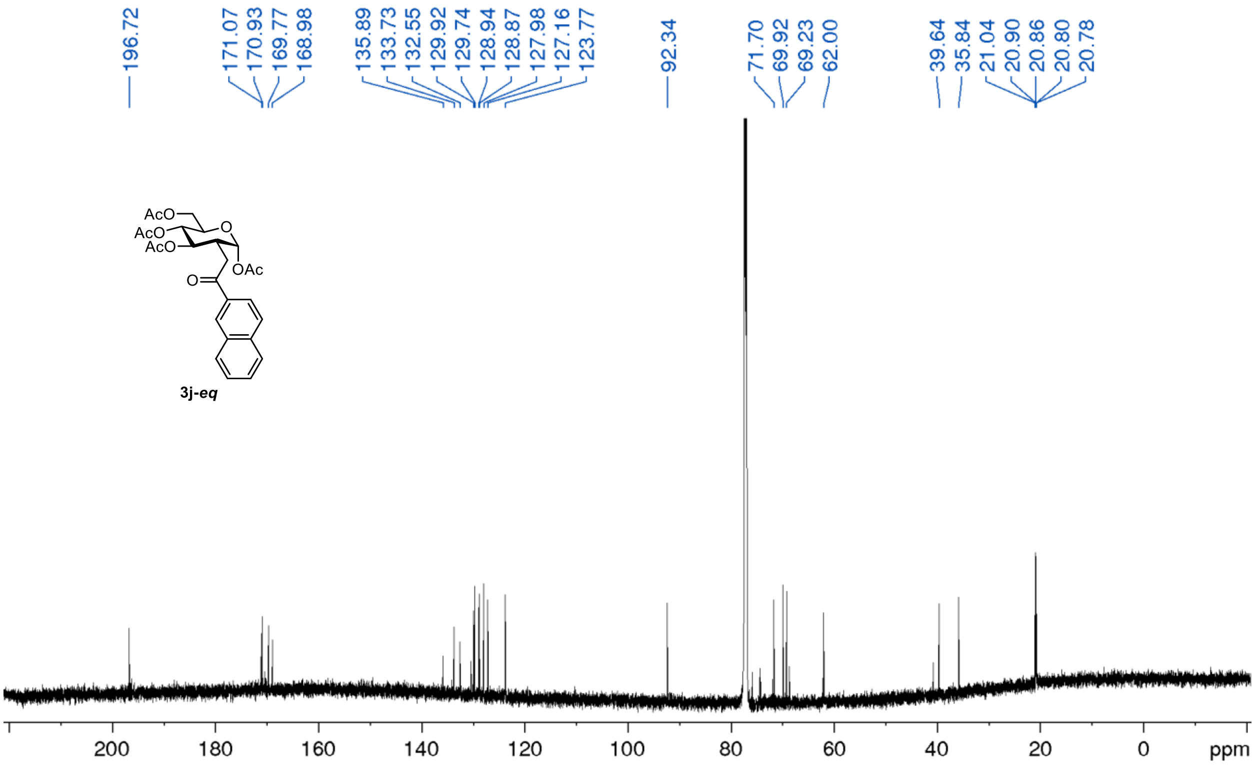

^{1}H NMR (500 MHz, CDCl$_3$, 25 °C) of (3k-*ax*)

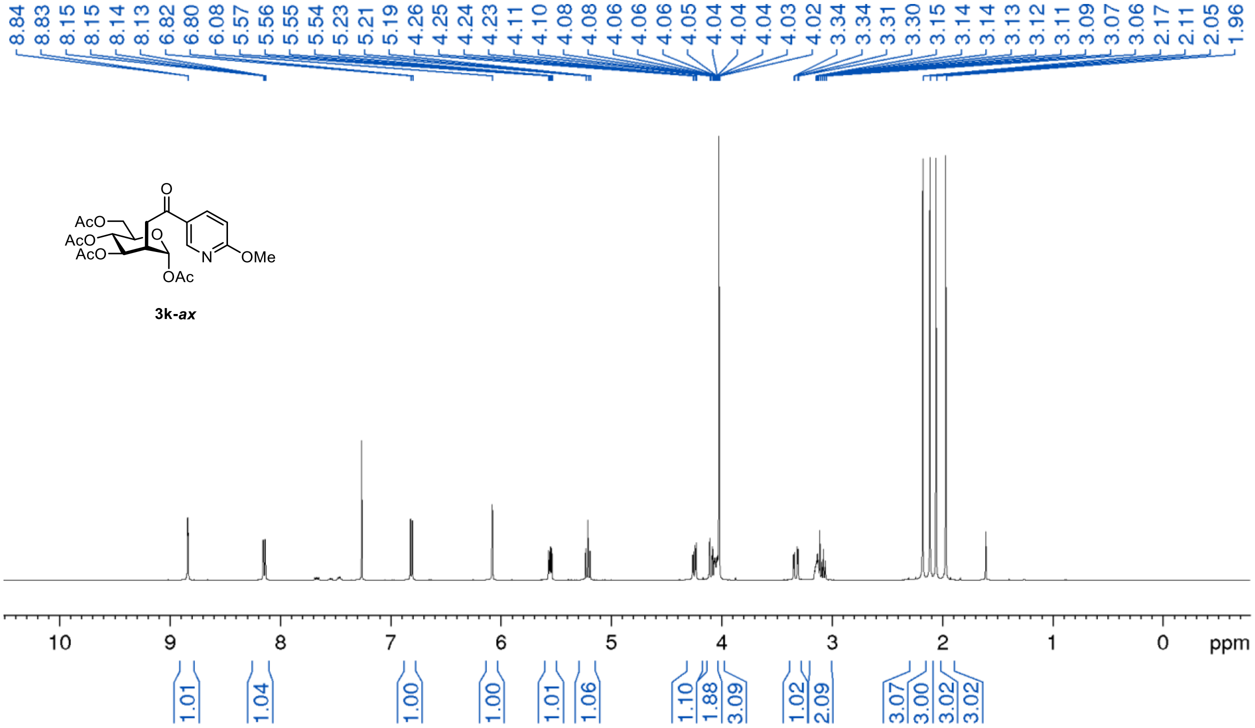

^{13}C NMR (125 MHz, CDCl$_3$, 25 °C) of (3k-*ax*)

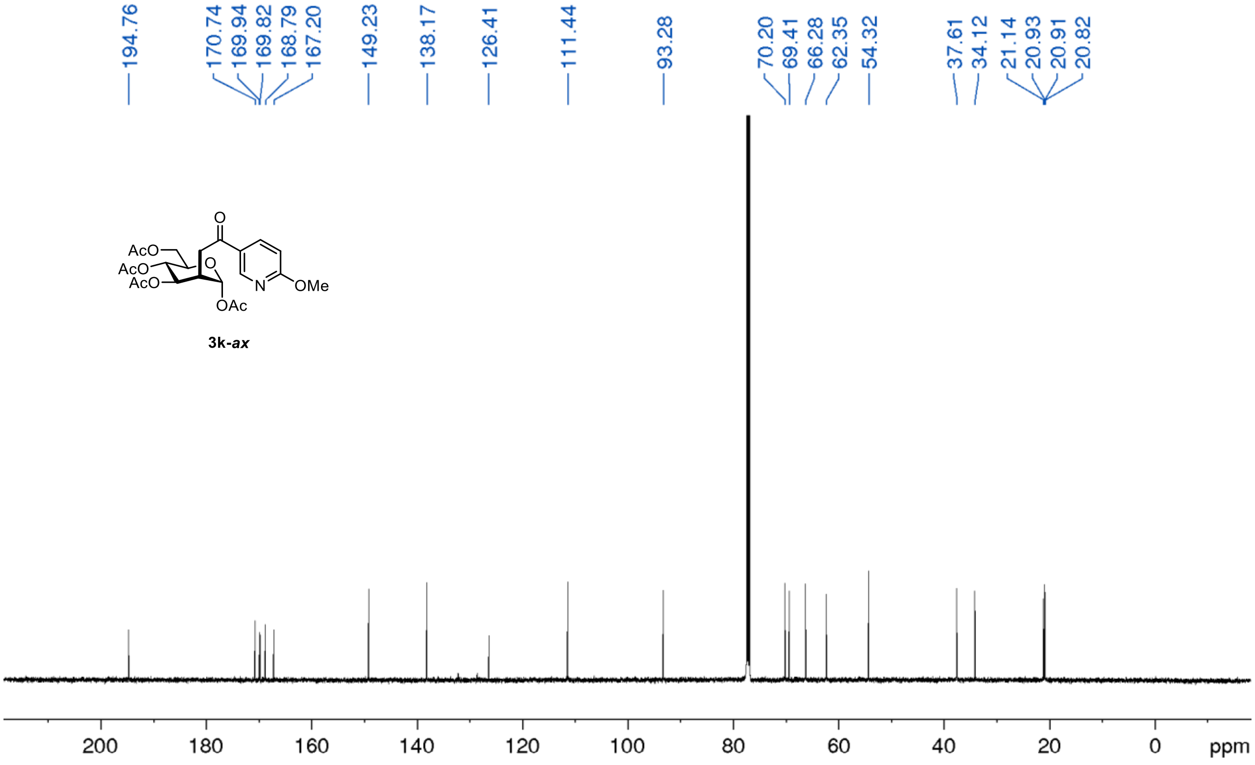

^{1}H NMR (500 MHz, $CDCl_3$, 25 °C) of (3k-*eq*)

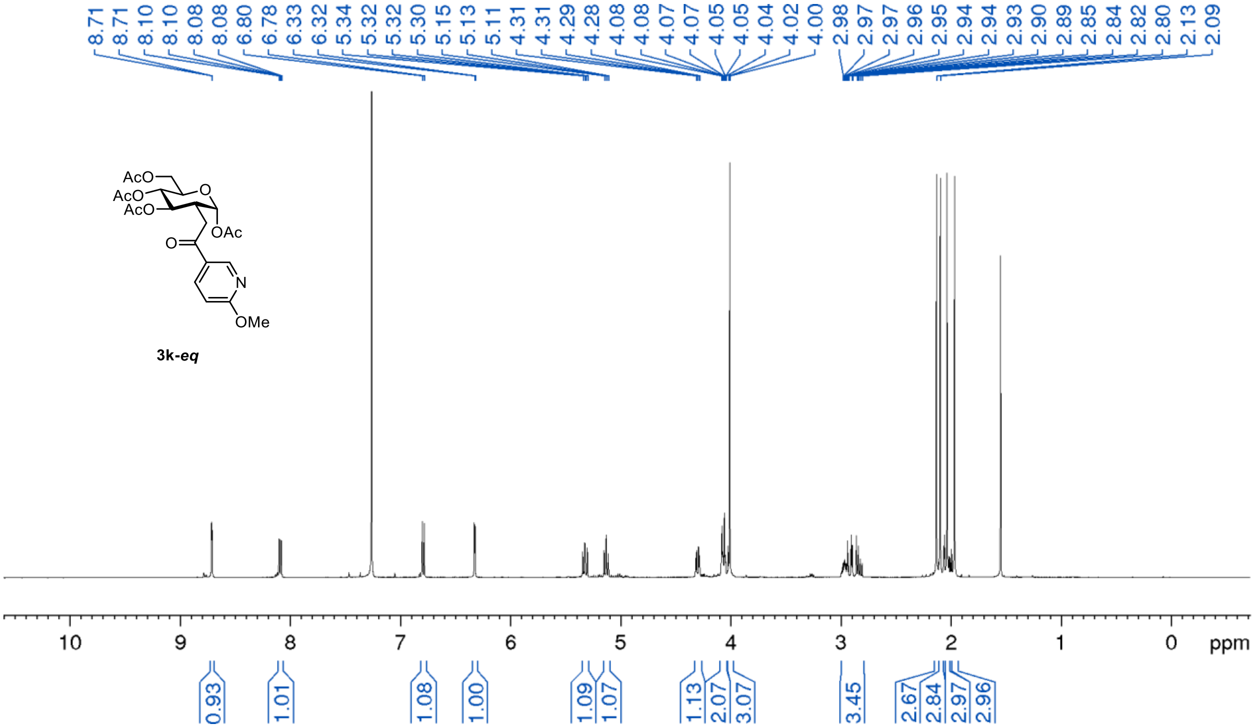

^{13}C NMR (125 MHz, $CDCl_3$, 25 °C) of (3k-*eq*)

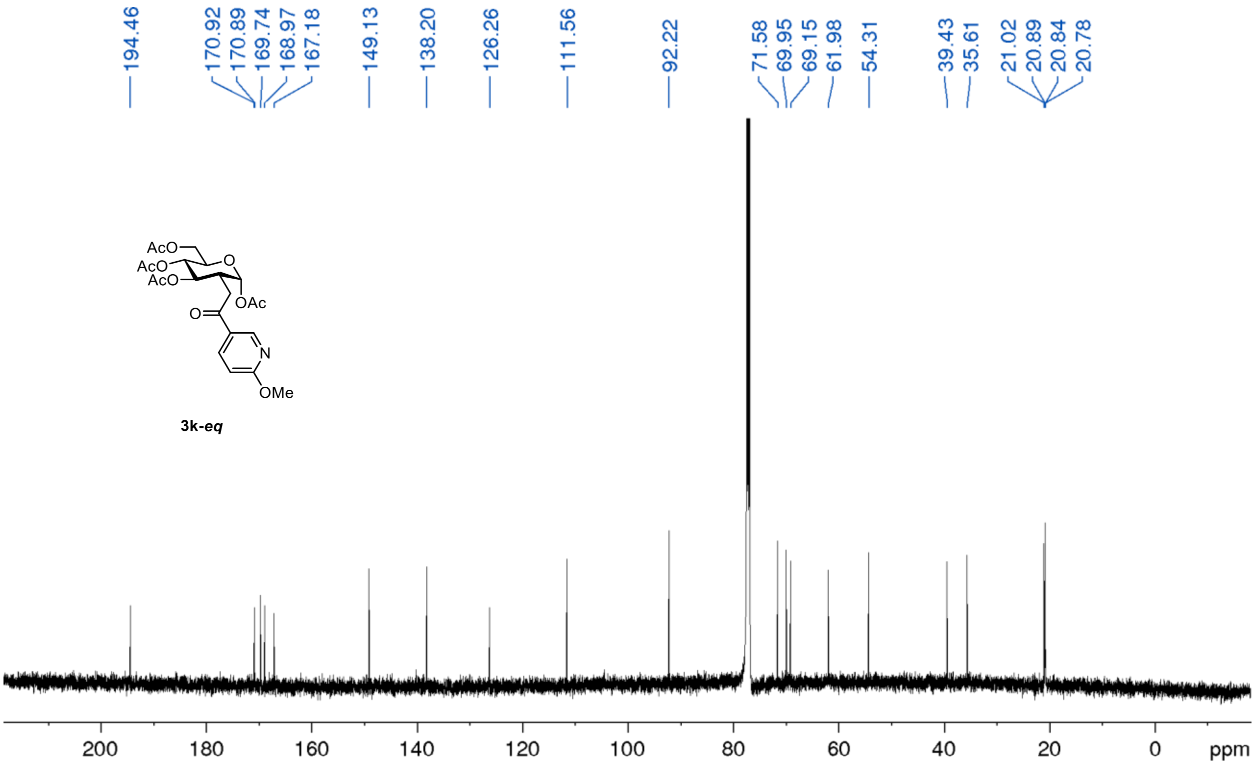

^{1}H NMR (700 MHz, CDCl$_3$, 25 °C) of (3l-*ax*)

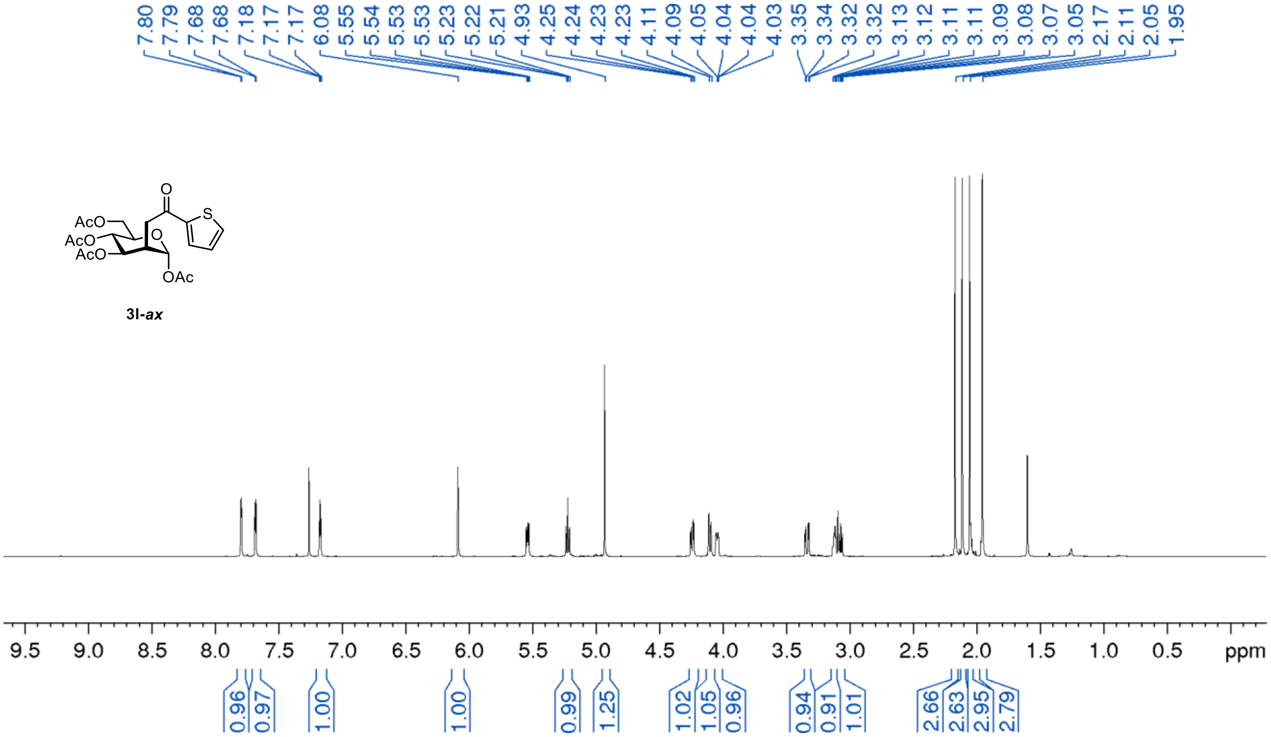

^{13}C NMR (175 MHz, CDCl$_3$, 25 °C) of (3l-*ax*)

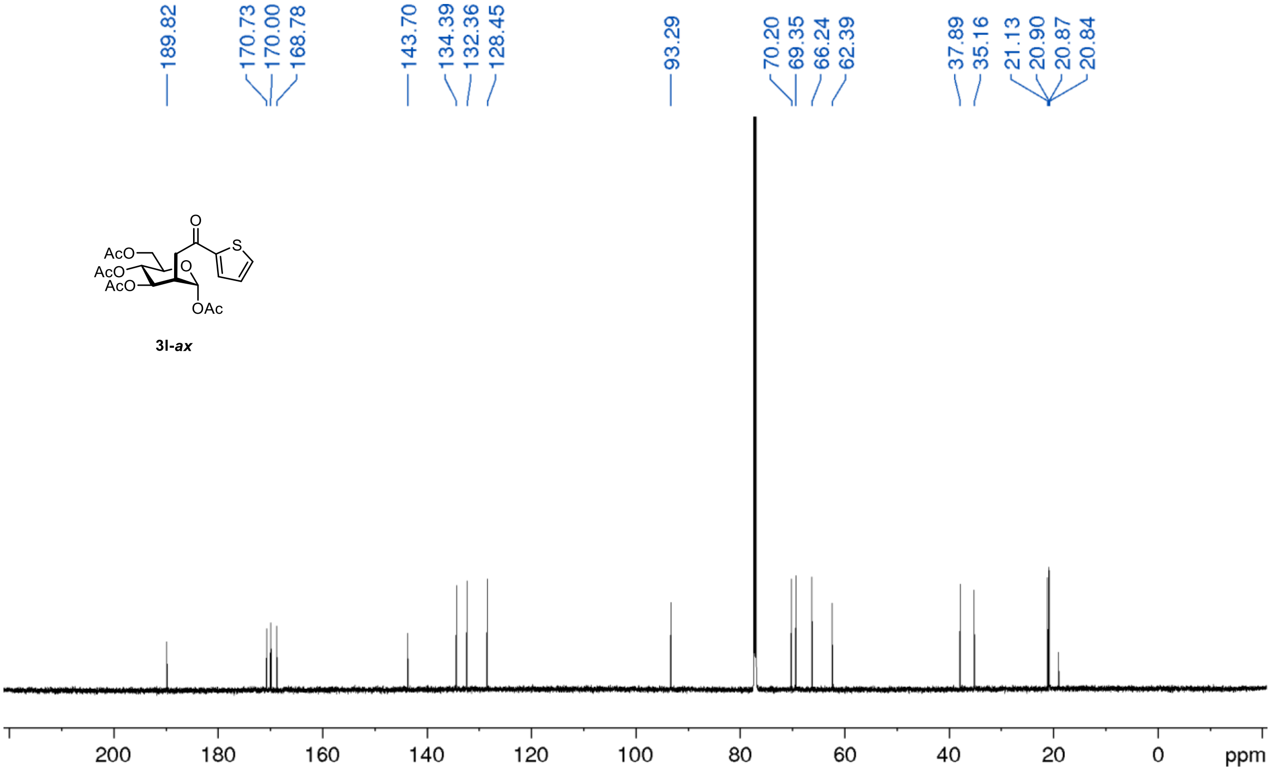

^{1}H NMR (500 MHz, $CDCl_3$, 25 ºC) of (3m-*ax*)

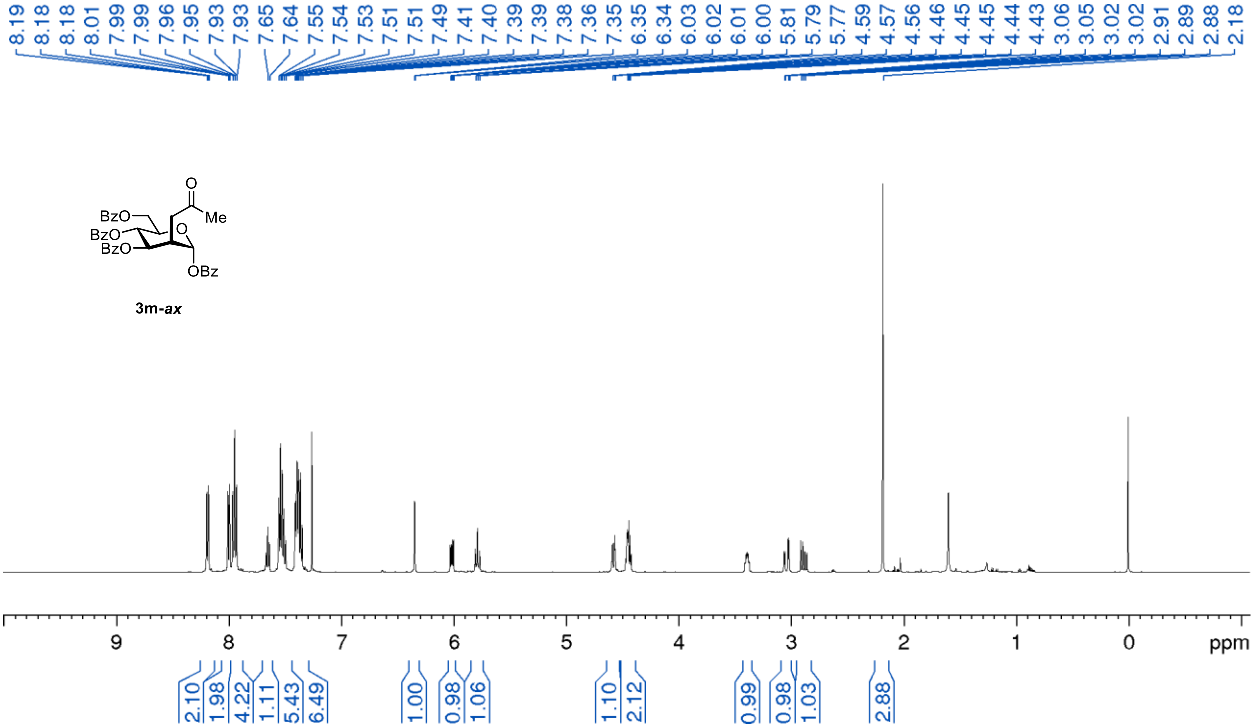

^{13}C NMR (125 MHz, $CDCl_3$, 25 ºC) of (3m-*ax*)

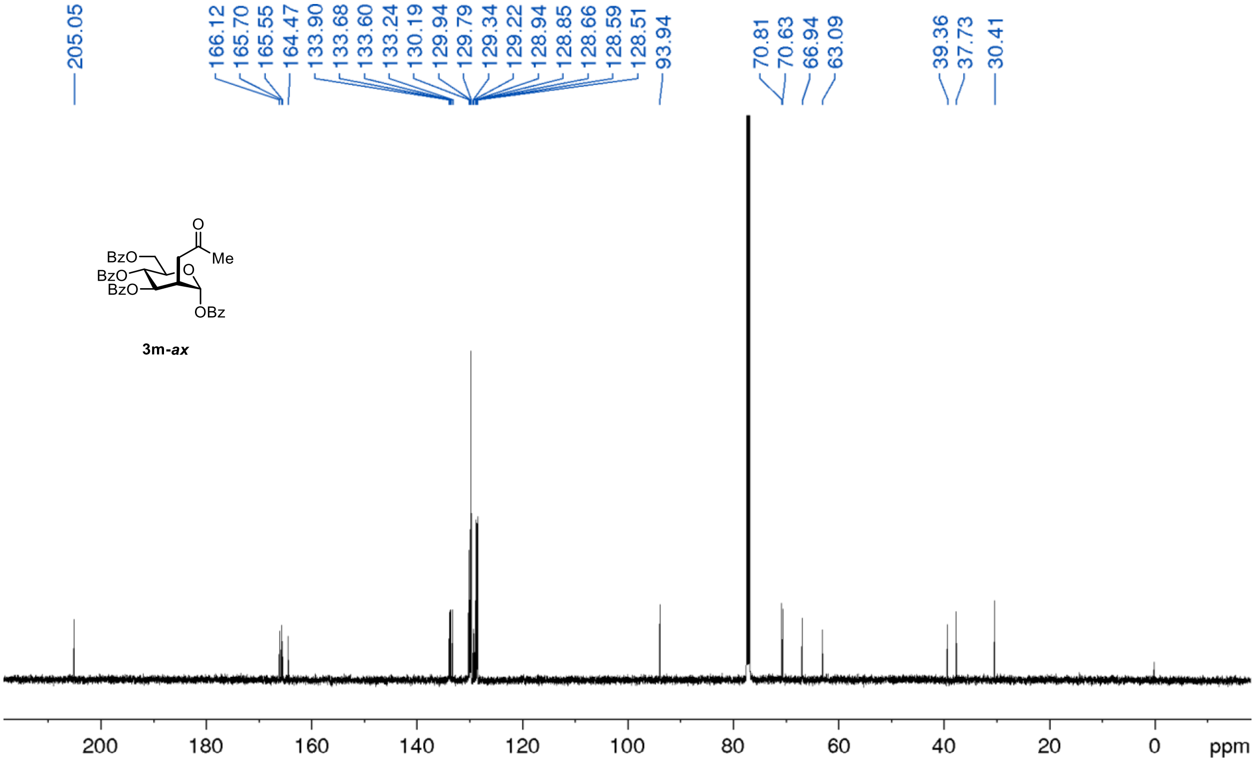

^{1}H NMR (700 MHz, $CDCl_3$, 25 °C) of (3m-*eq*)

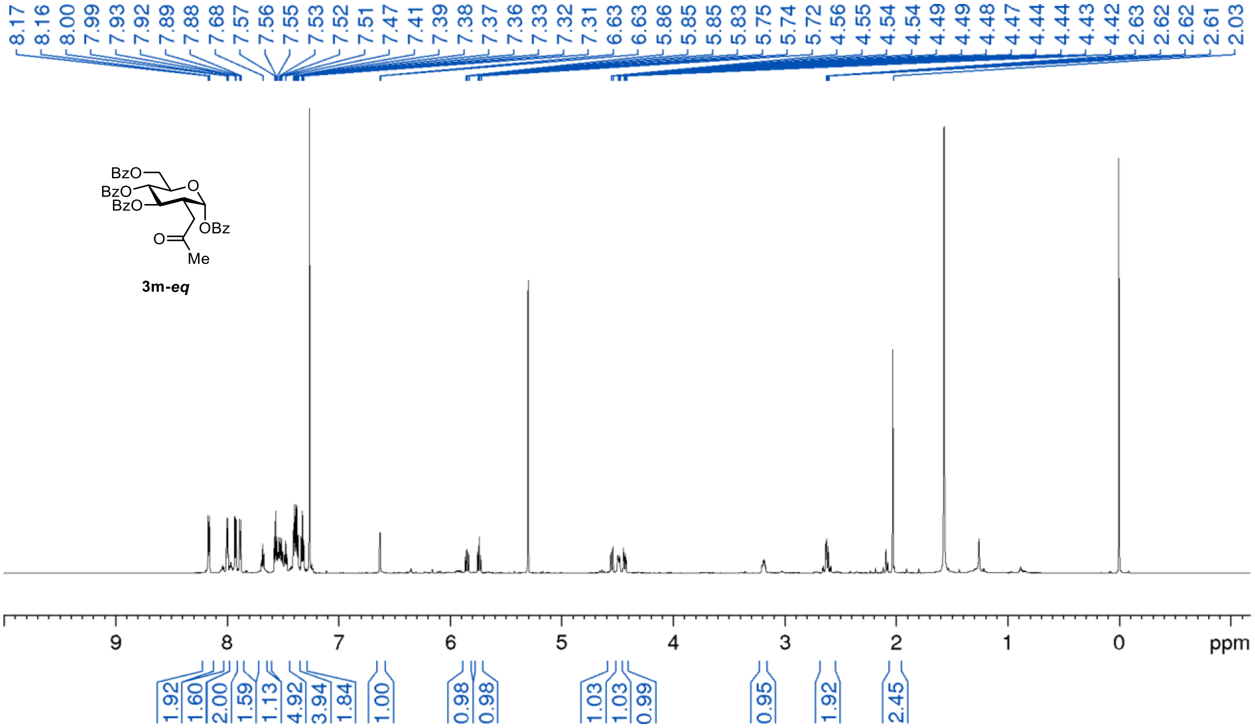

^{13}C NMR (175 MHz, $CDCl_3$, 25 °C) of (3m-*eq*)

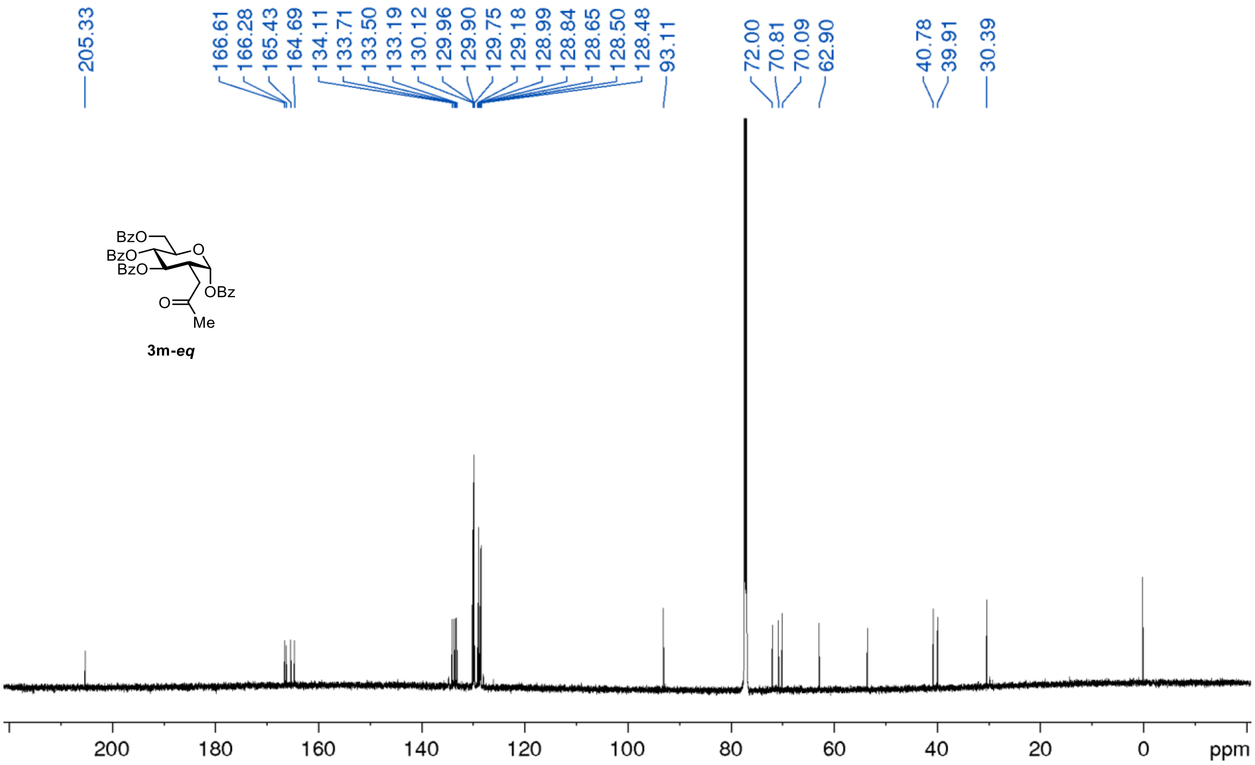

^{1}H NMR (700 MHz, $CDCl_3$, 25 °C) of (3n-*ax*)

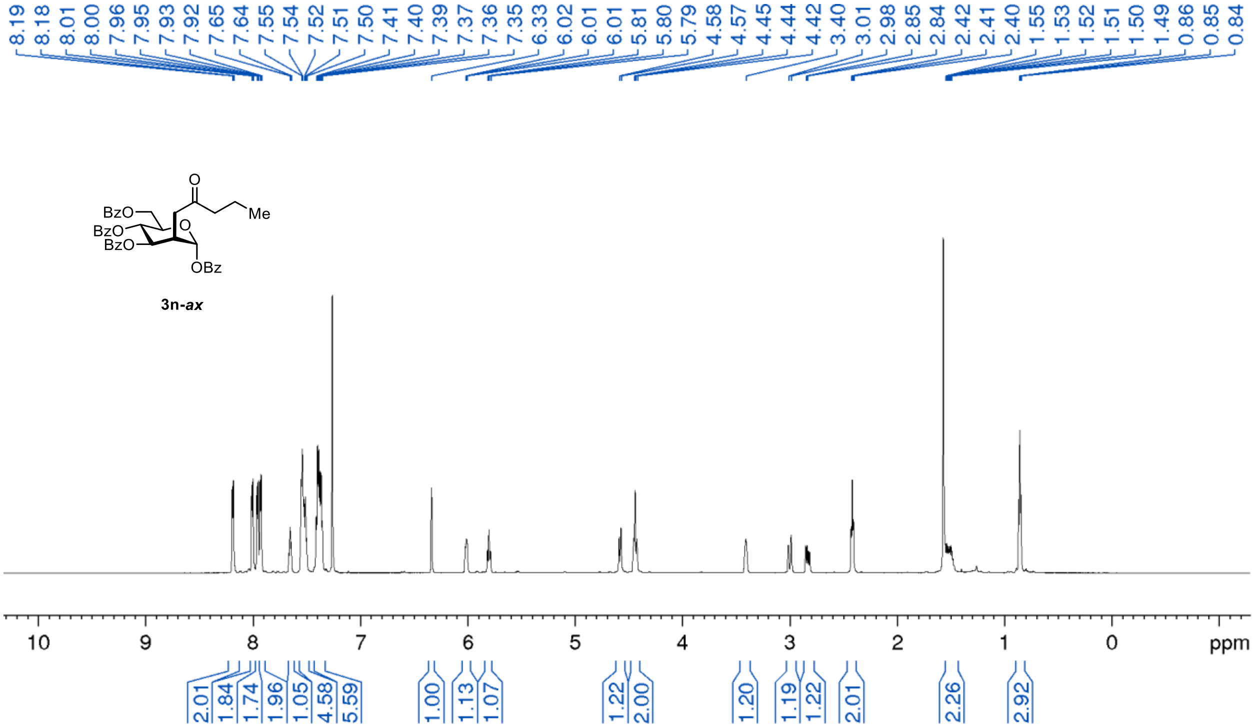

^{13}C NMR (175 MHz, $CDCl_3$, 25 °C) of (3n-*ax*)

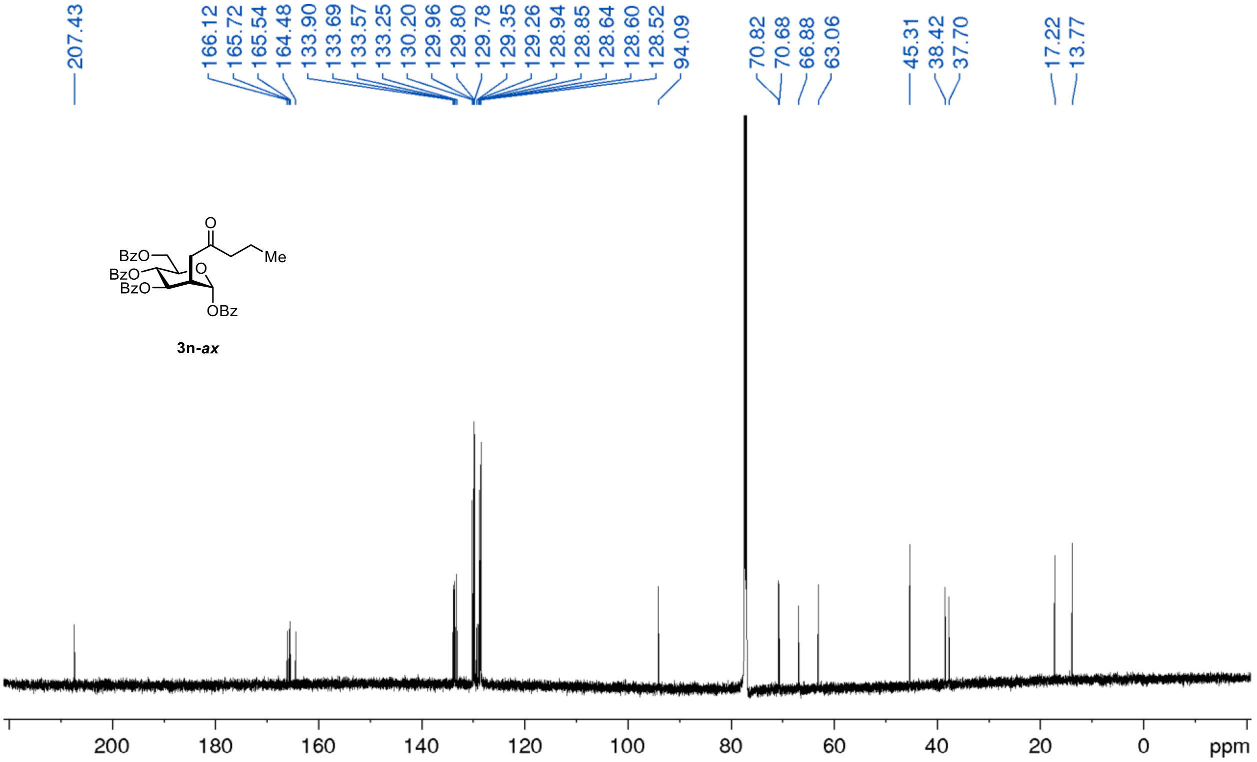

^{1}H NMR (700 MHz, CDCl3, 25 °C) of (3n-*eq*)

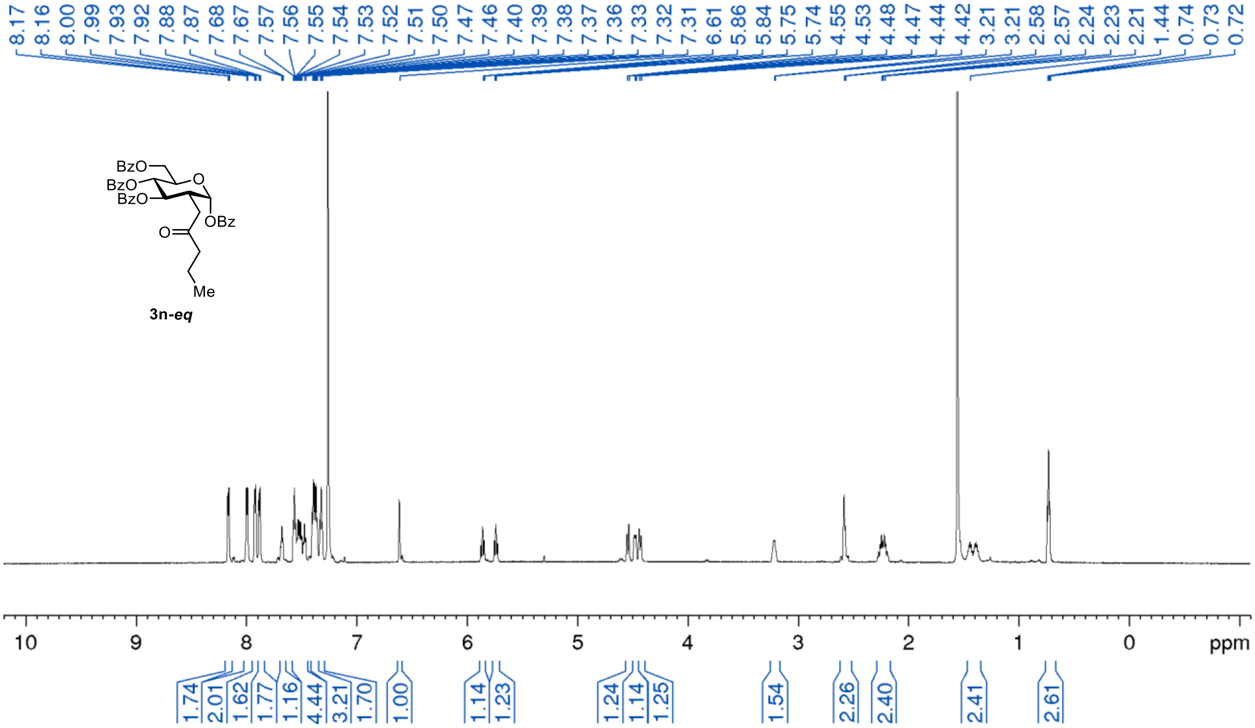

^{13}C NMR (175 MHz, CDCl3, 25 °C) of (3n-*eq*)

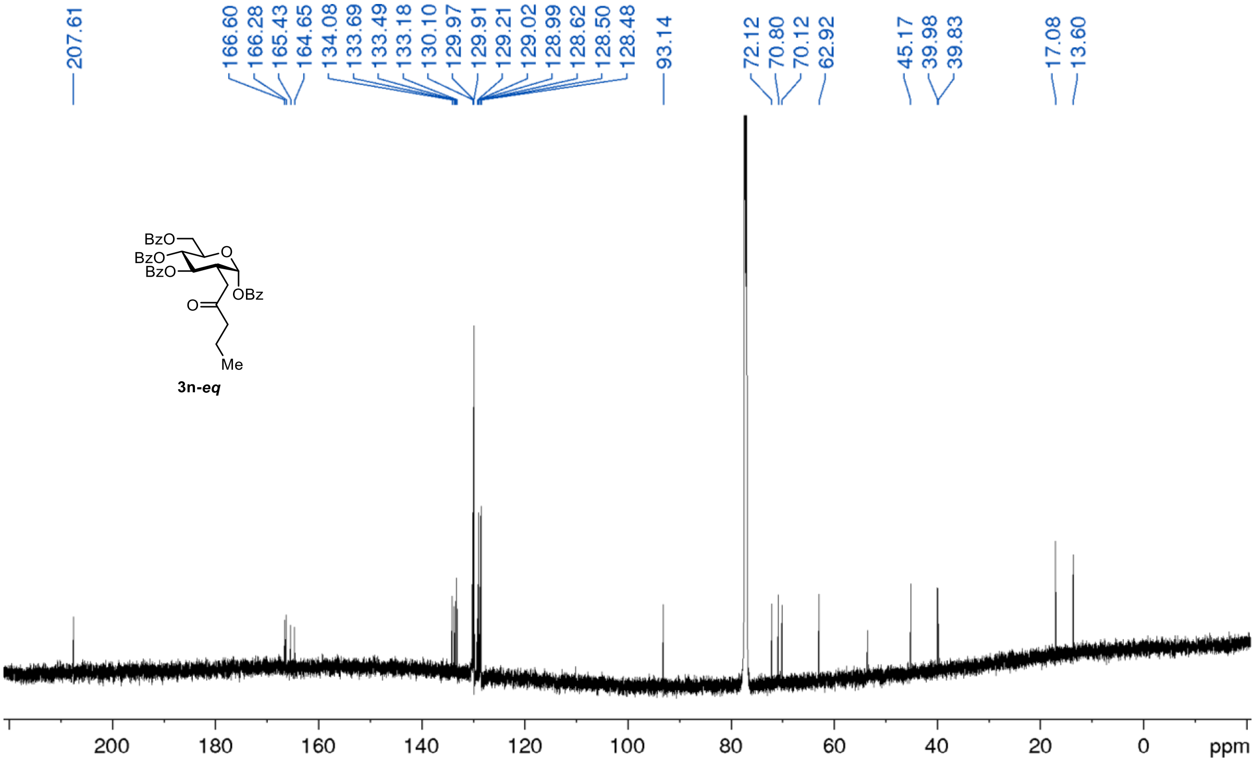

^{1}H NMR (500 MHz, $CDCl_3$, 25 °C) of (3o-*ax*)

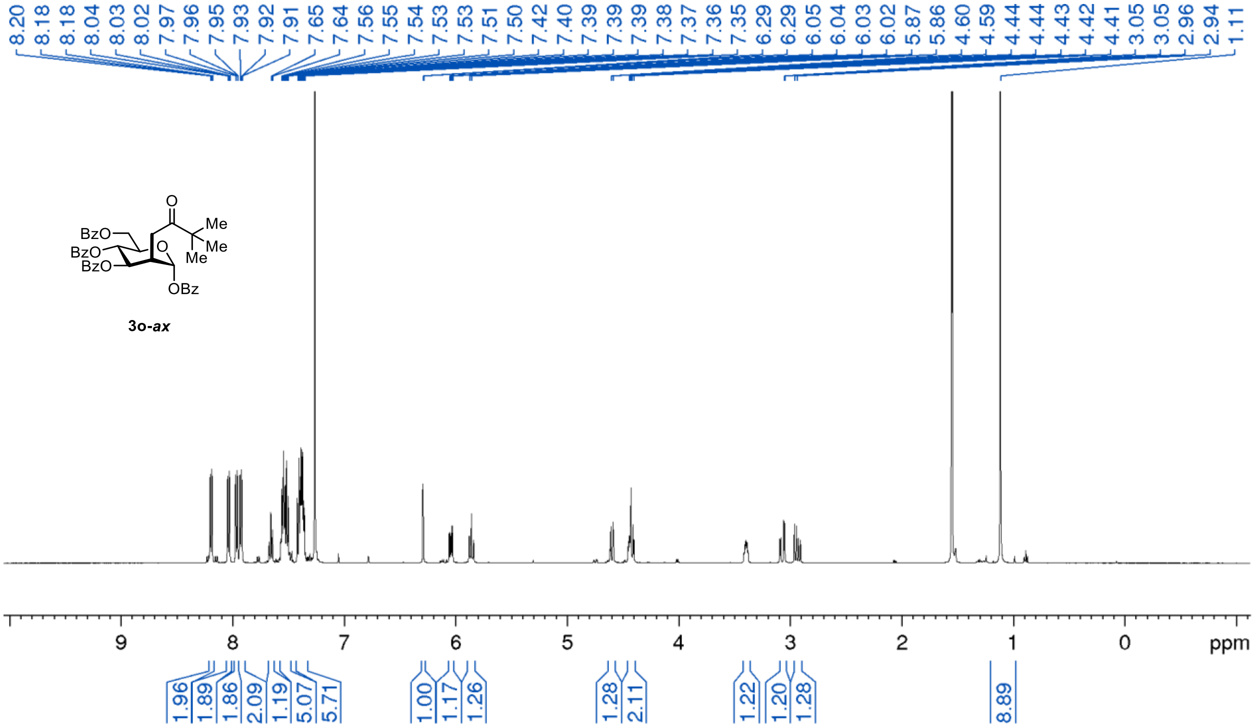

^{13}C NMR (125 MHz, $CDCl_3$, 25 °C) of (3o-*ax*)

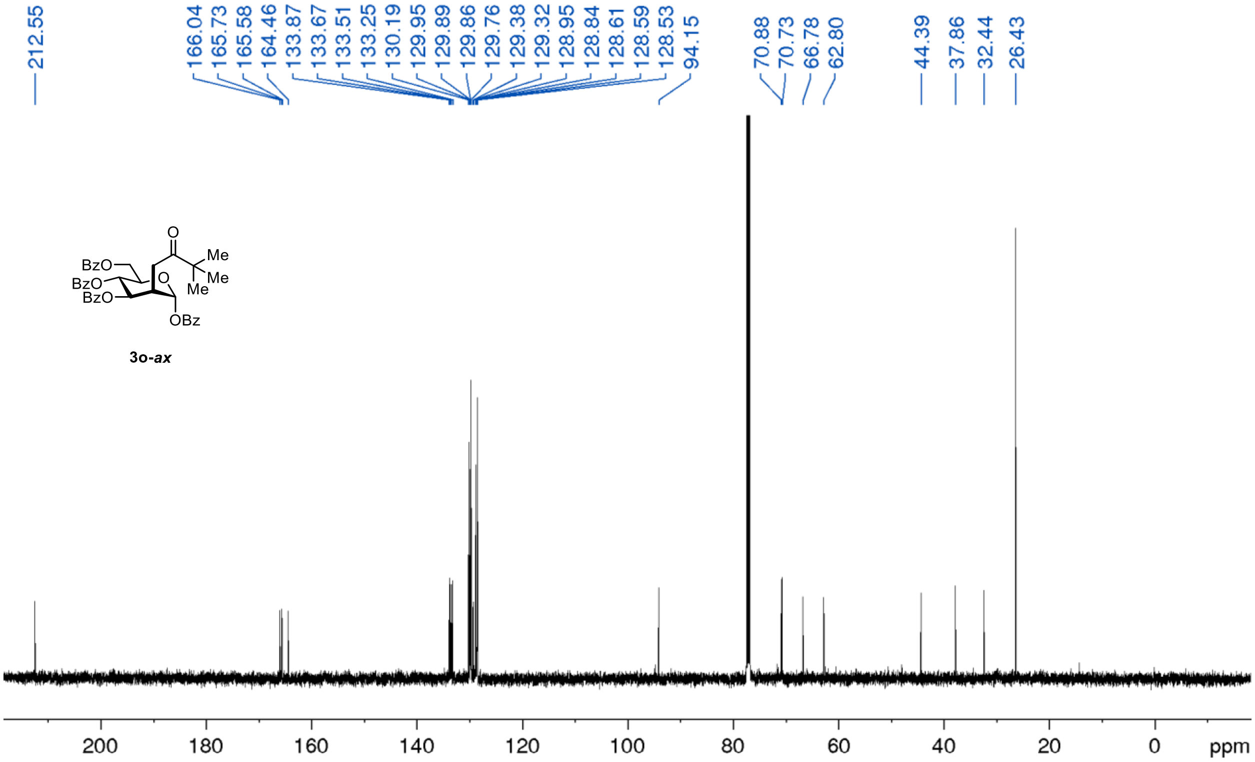

^{1}H NMR (700 MHz, CD_3CN, 25 ºC) of (3p-*ax*)

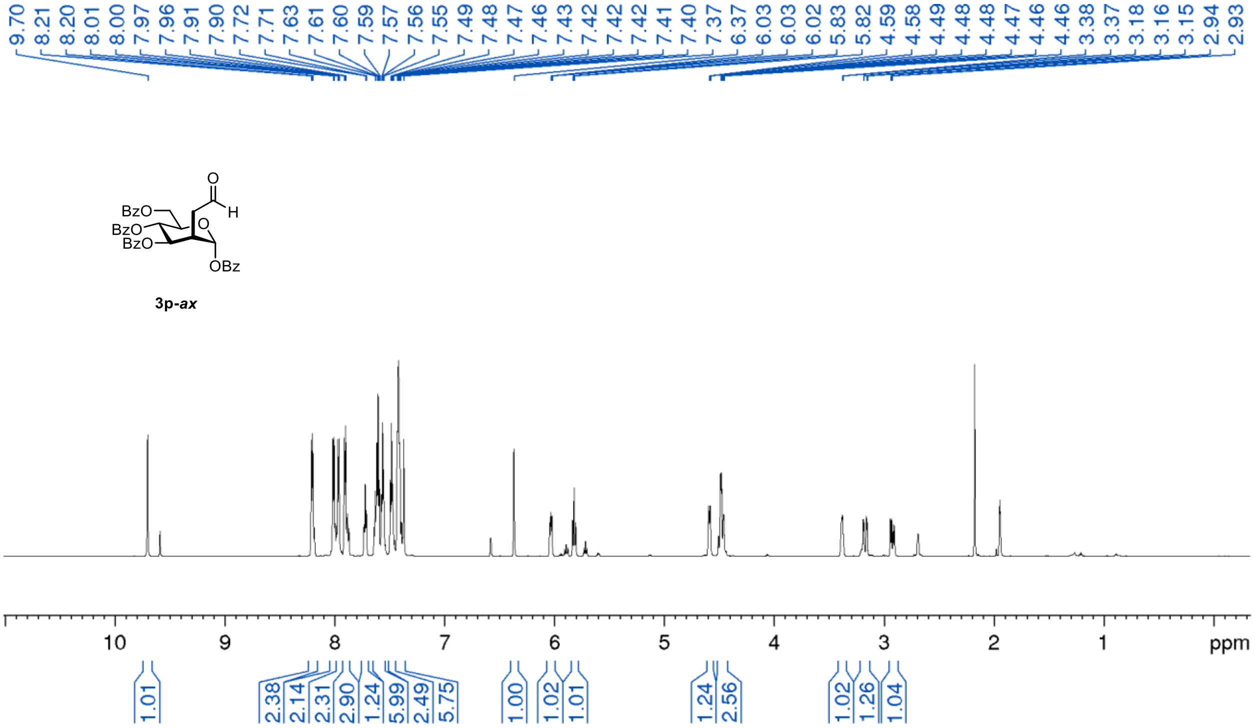

^{13}C NMR (175 MHz, CD_3CN, 25 ºC) of (3p-*ax*)

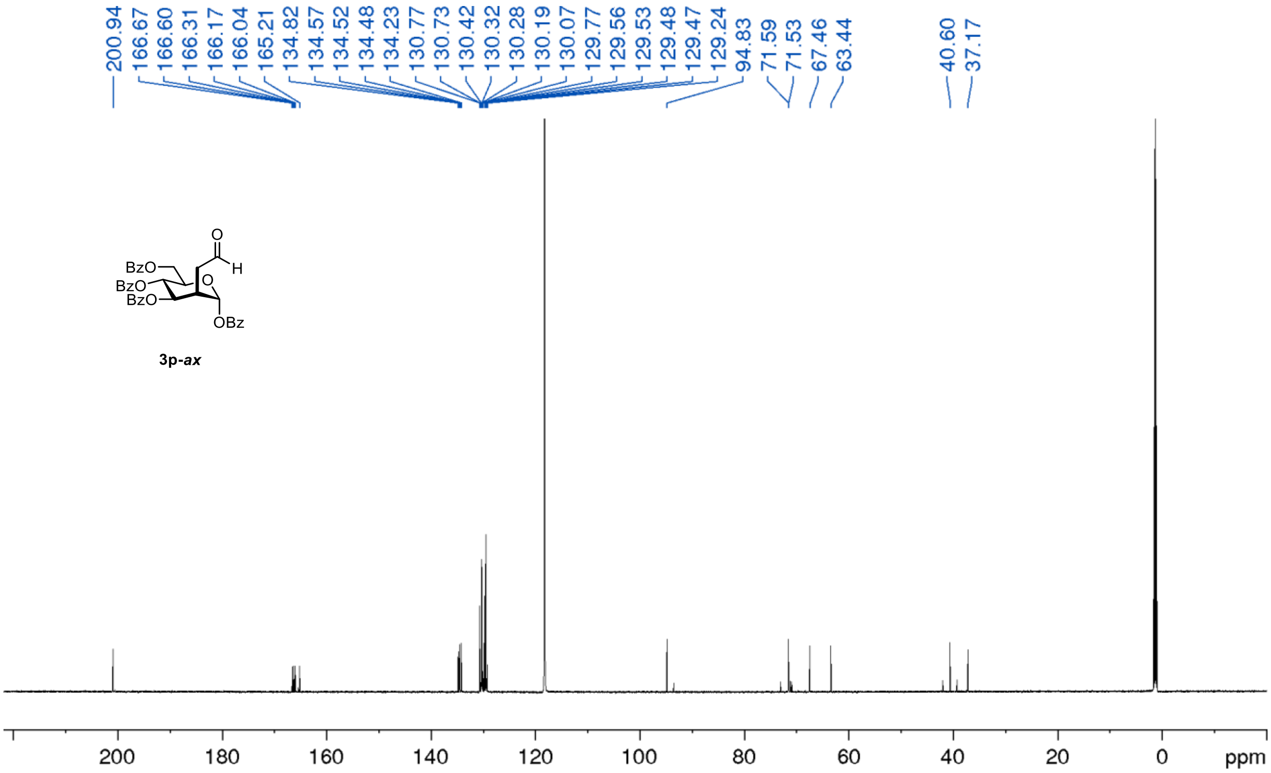

^{1}H NMR (500 MHz, CDCl$_3$, 25 °C) of (3q-*ax*)

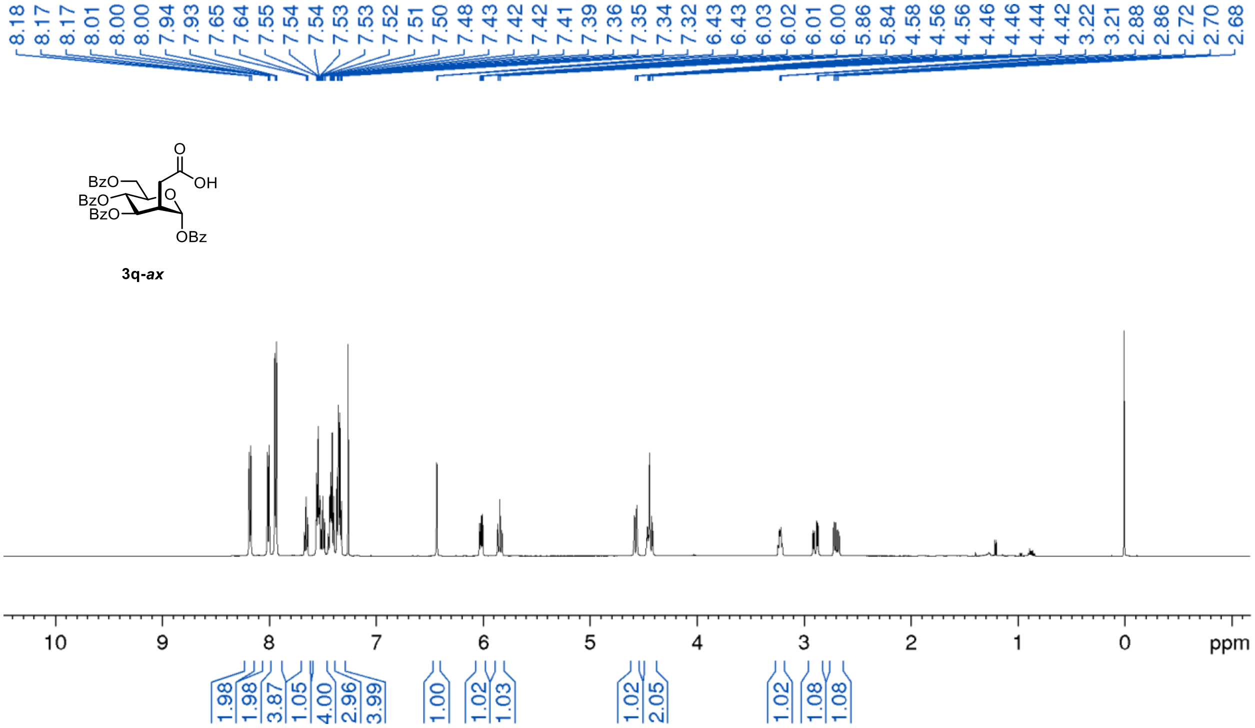

^{13}C NMR (125 MHz, CDCl$_3$, 25 °C) of (3q-*ax*)

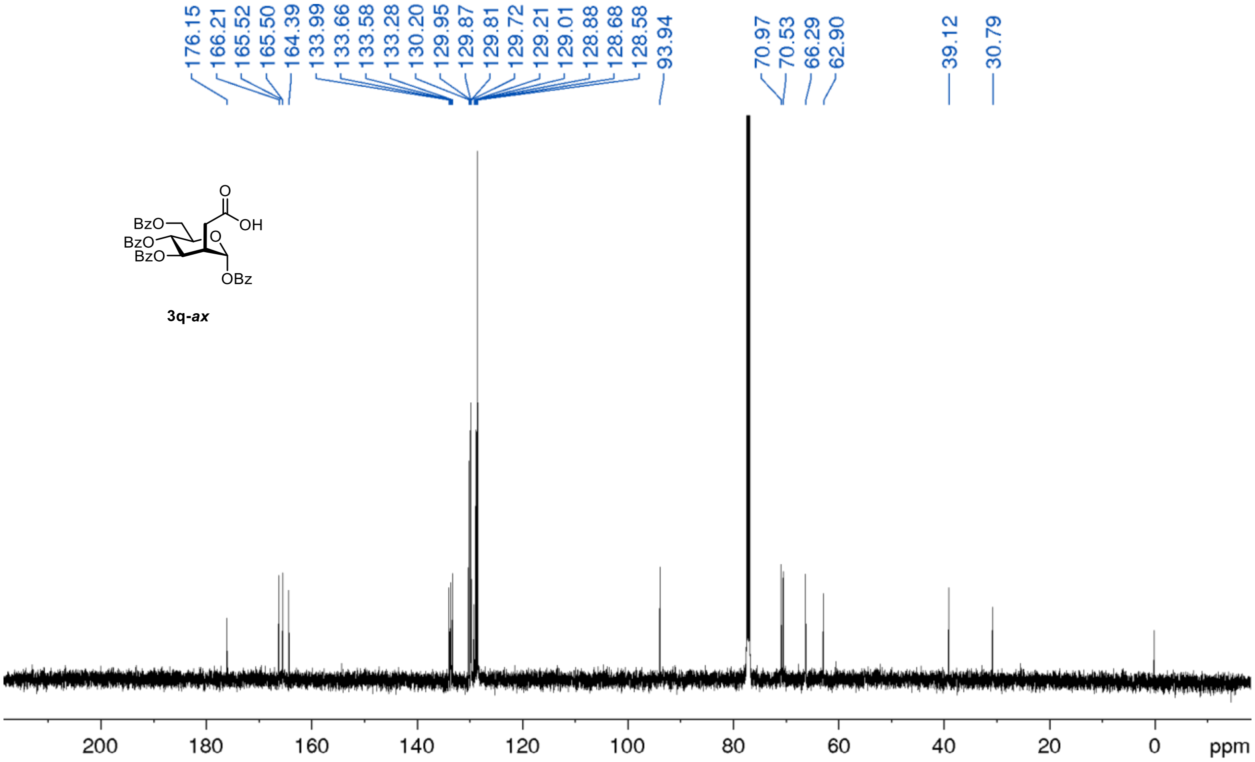

^{1}H NMR (500 MHz, $CDCl_3$, 25 °C) of (3q-*eq*)

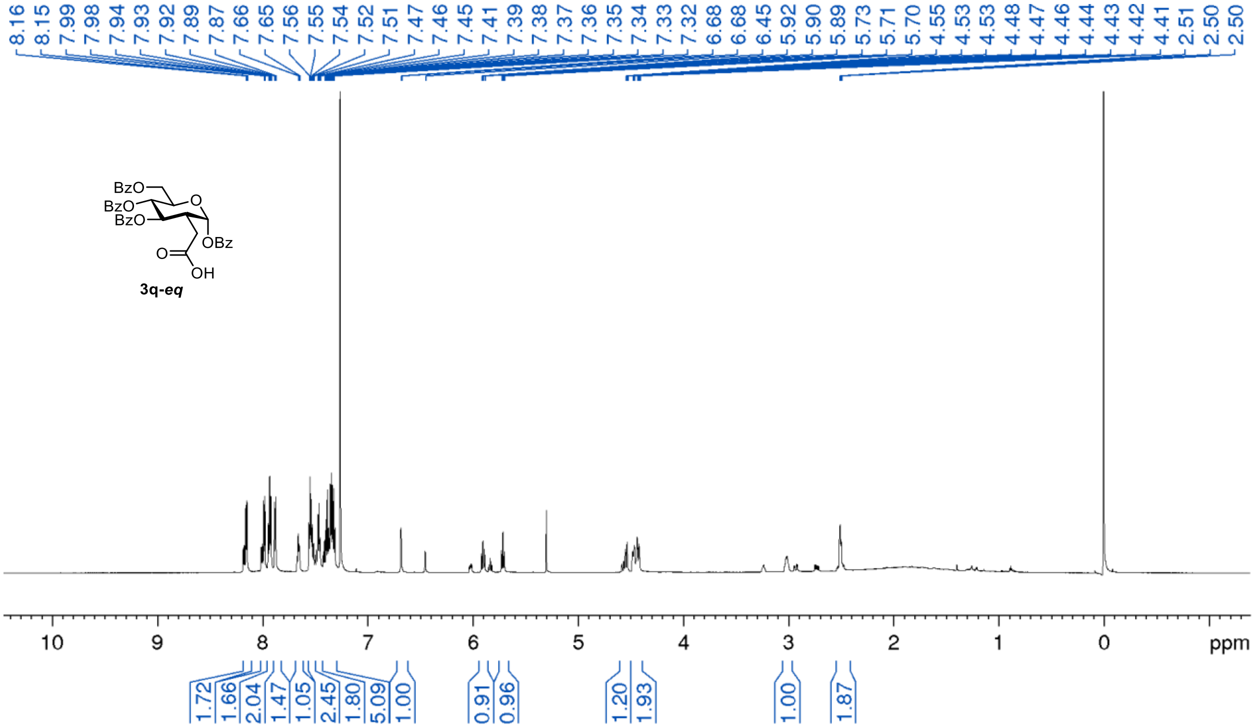

^{13}C NMR (125 MHz, $CDCl_3$, 25 °C) of (3q-*eq*)

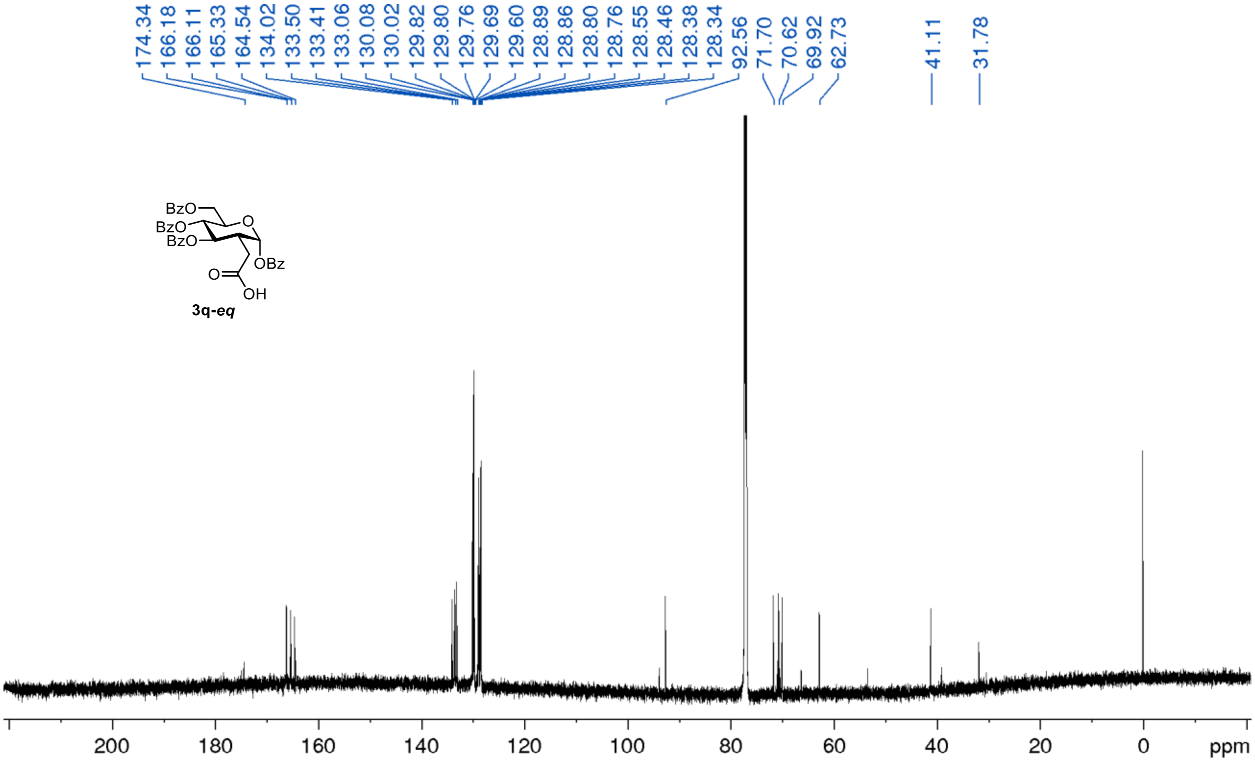

^{1}H NMR (700 MHz, $CDCl_3$, 25 °C) of (3r-*ax*)

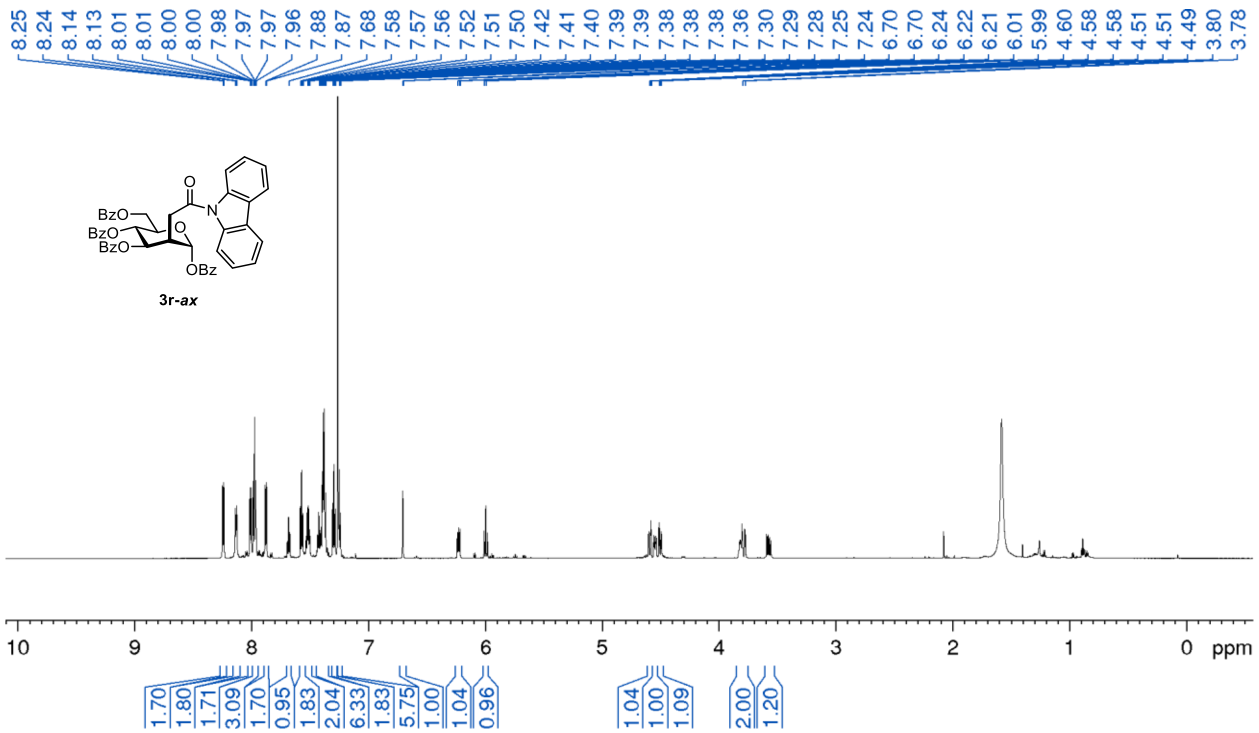

^{13}C NMR (175 MHz, $CDCl_3$, 25 °C) of (3r-*ax*)

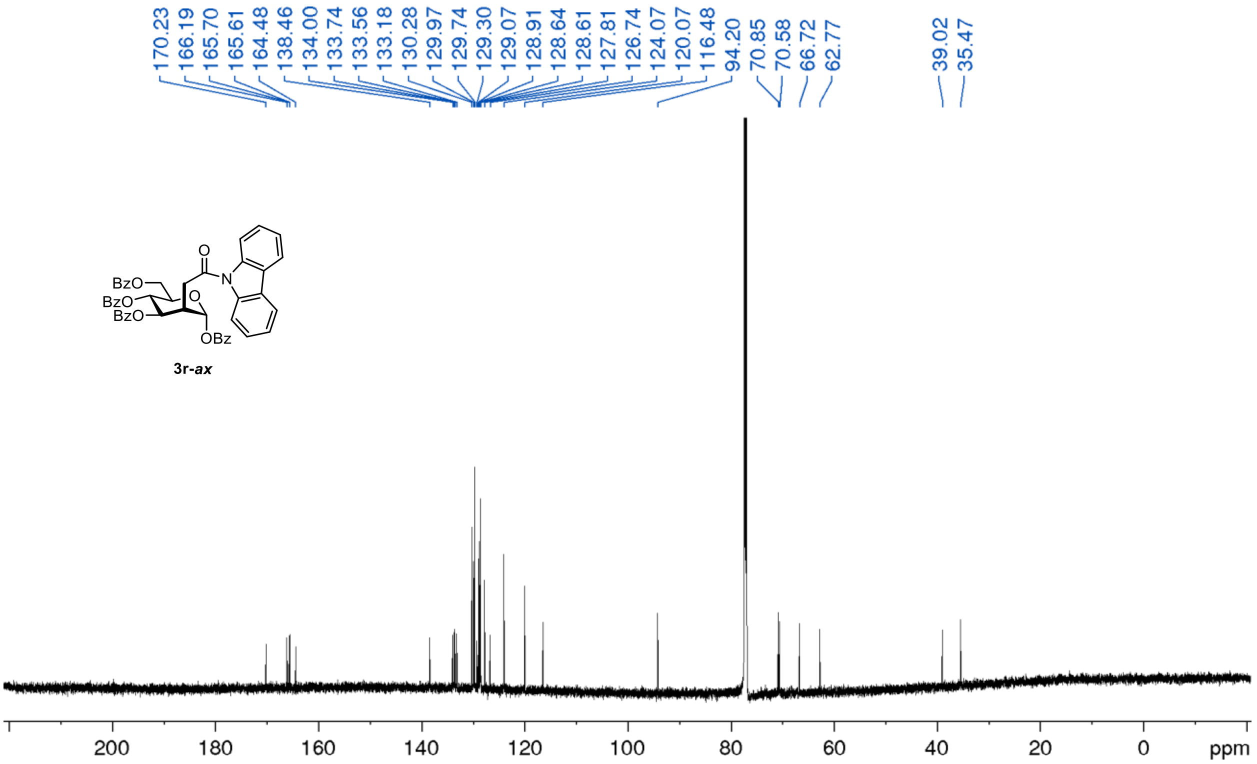

^{1}H NMR (500 MHz, $CDCl_3$, 25 ºC) of (4b-*ax*)

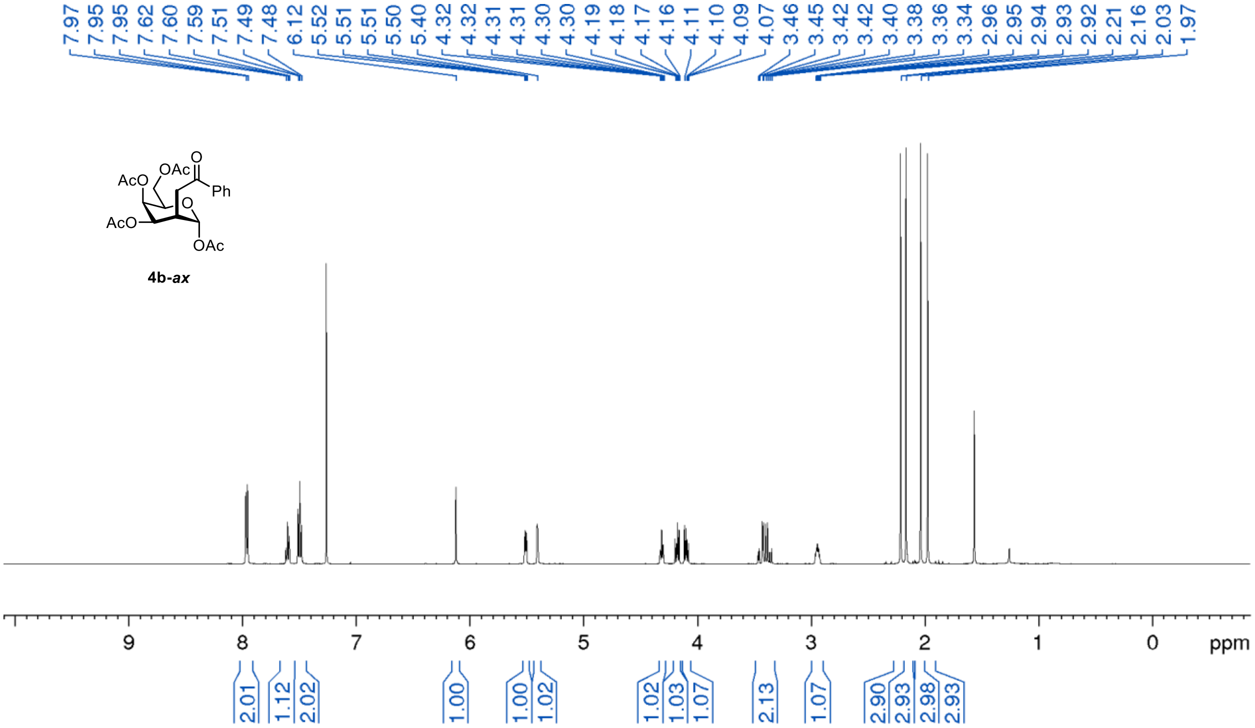

^{13}C NMR (125 MHz, $CDCl_3$, 25 ºC) of (4b-*ax*)

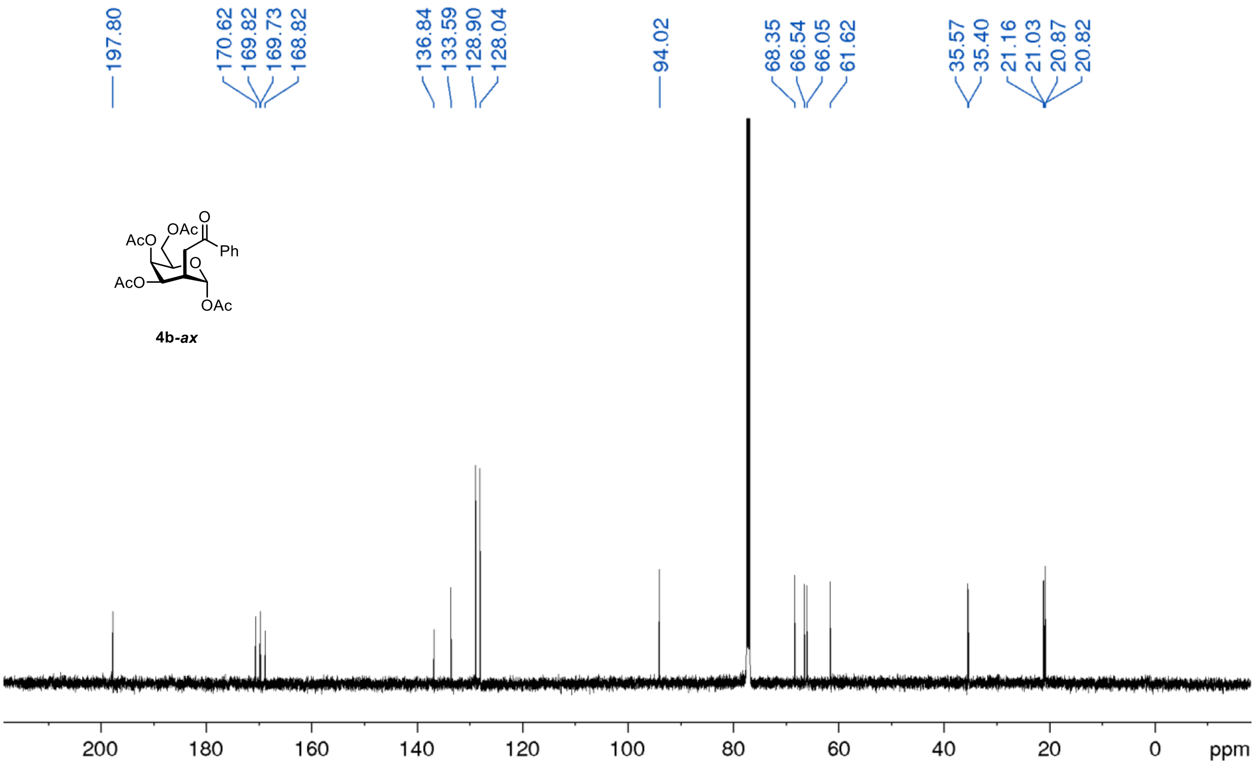

^{1}H NMR (500 MHz, $CDCl_3$, 25 °C) of (4b-*eq*)

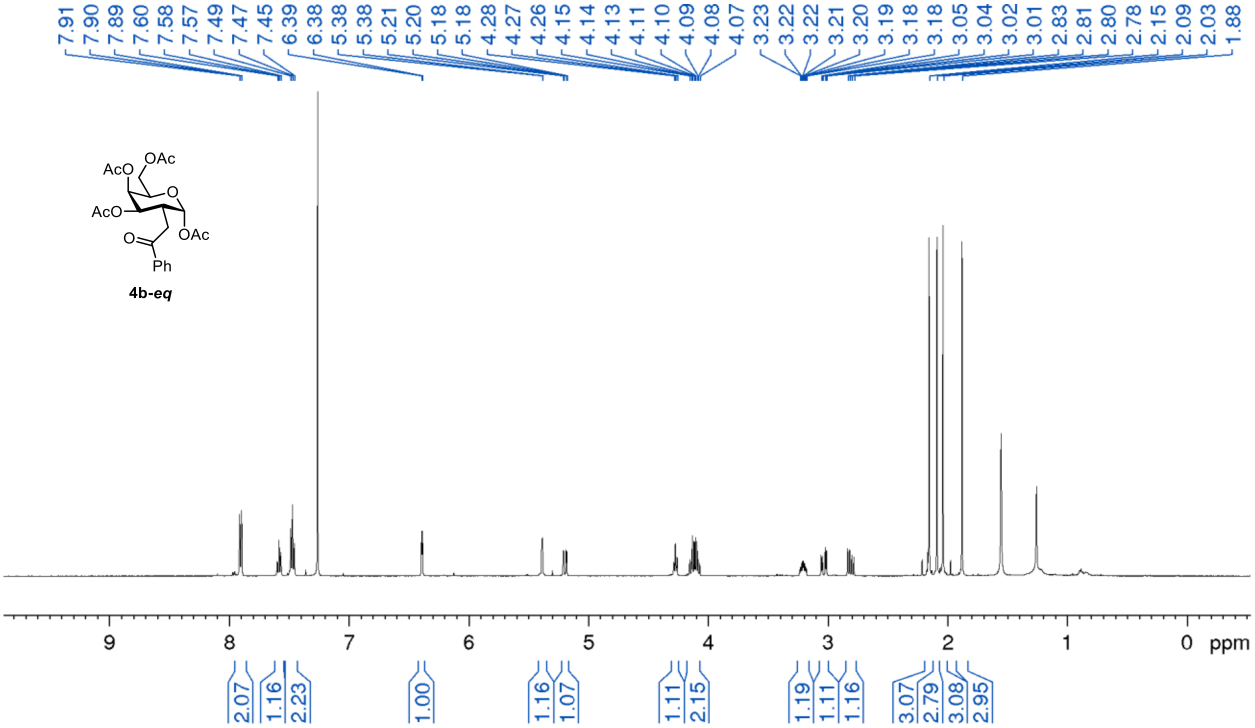

^{13}C NMR (175 MHz, $CDCl_3$, 25 °C) of (4b-*eq*)

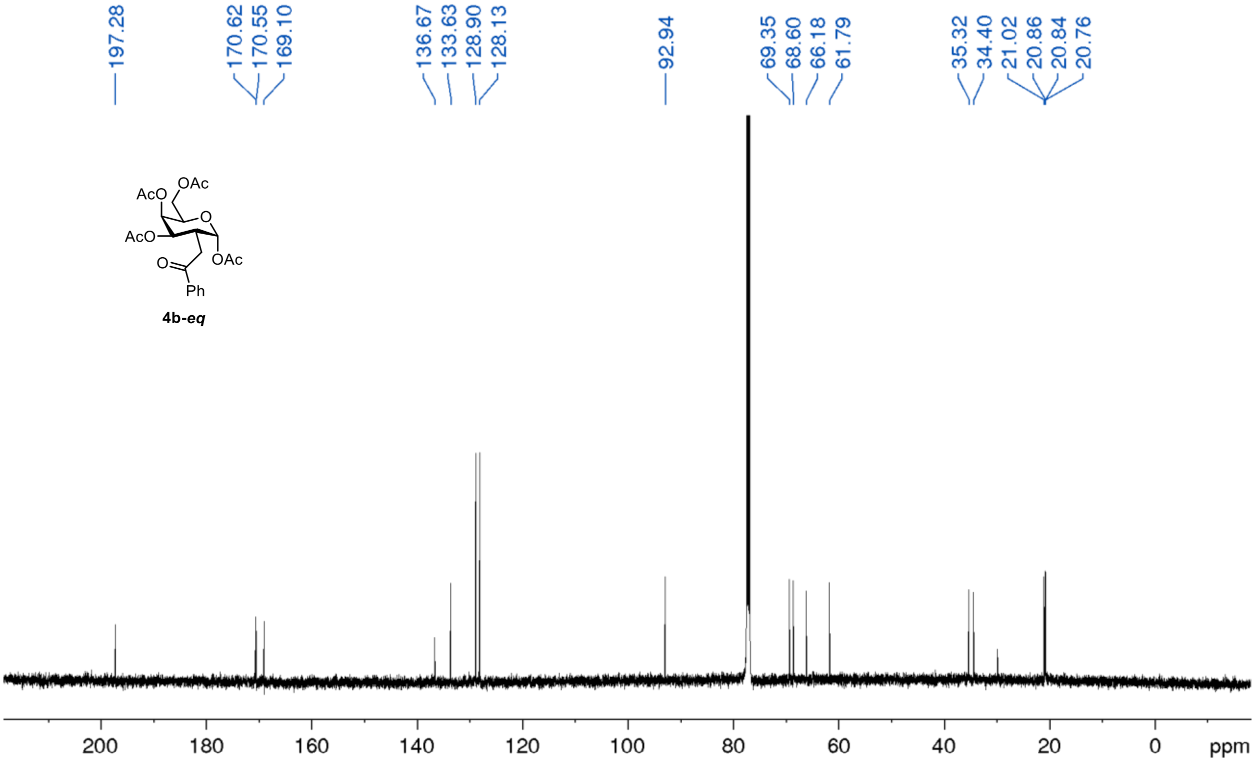

^{1}H NMR (500 MHz, CDCl$_3$, 25 °C) of (4c-*ax*)

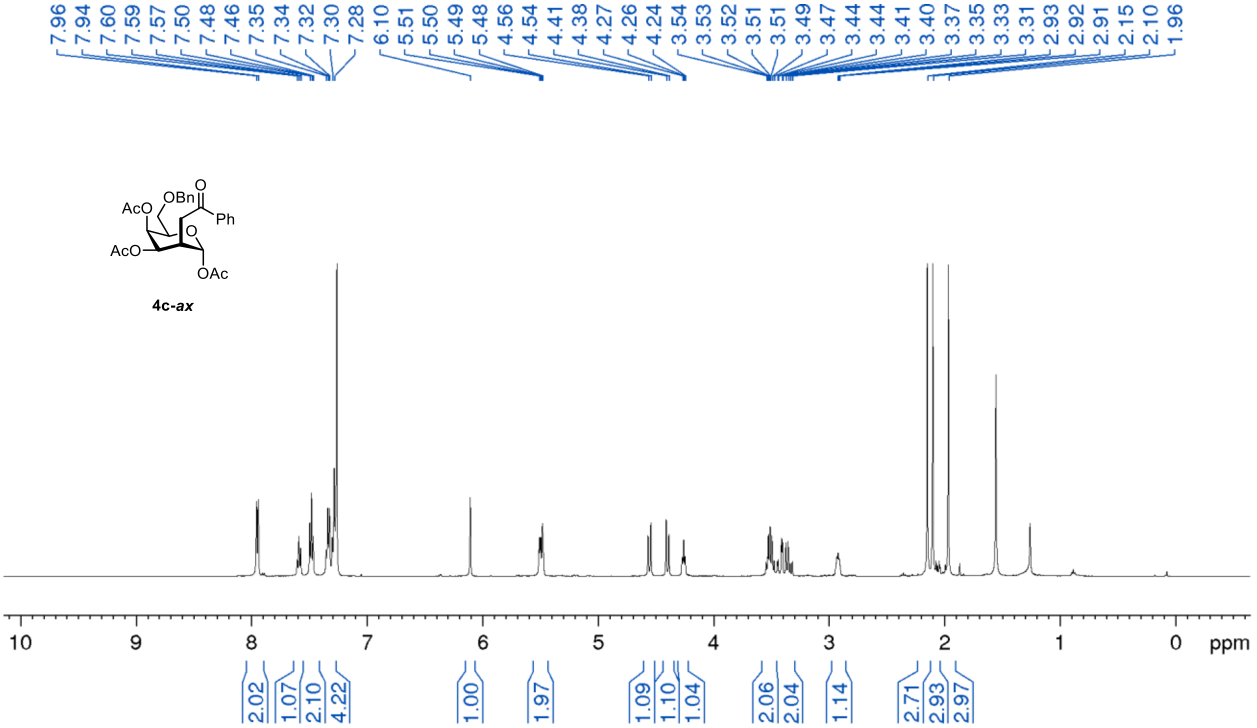

^{13}C NMR (125 MHz, CDCl$_3$, 25 °C) of (4c-*ax*)

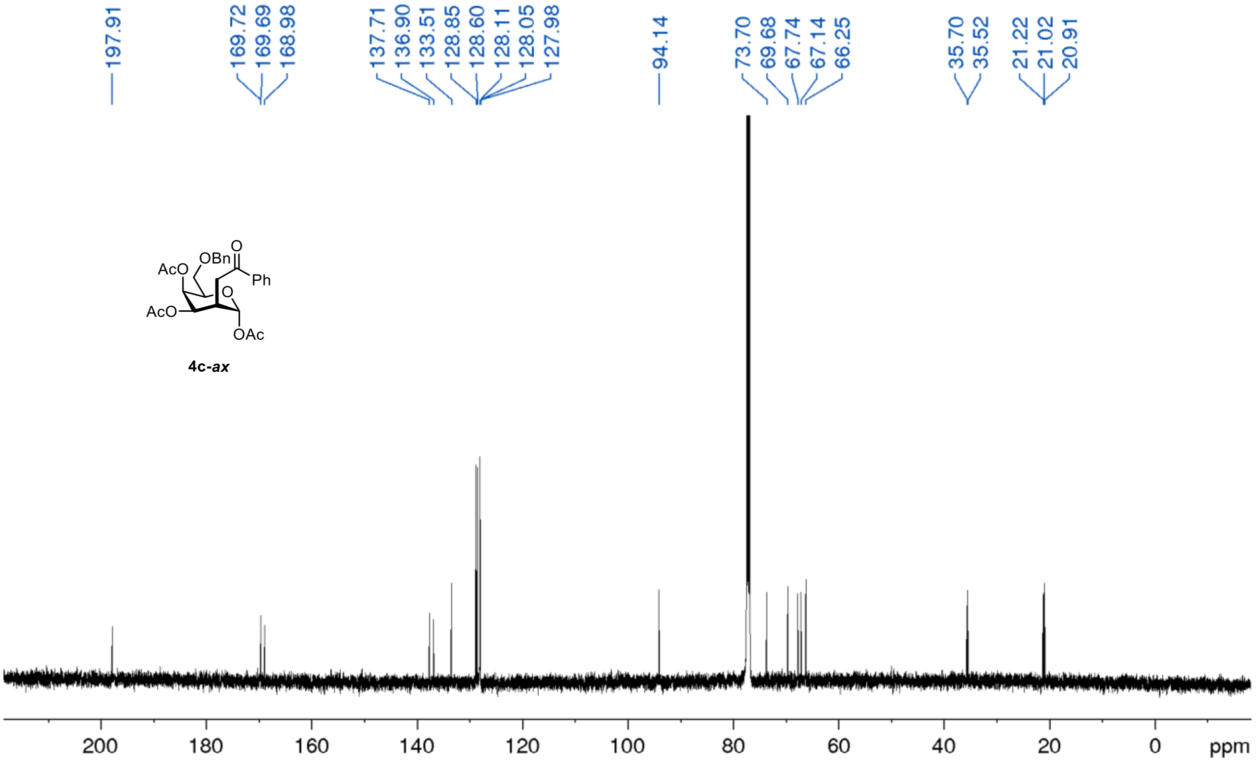

^{1}H NMR (700 MHz, CDCl$_3$, 25 °C) of (4c-*eq*)

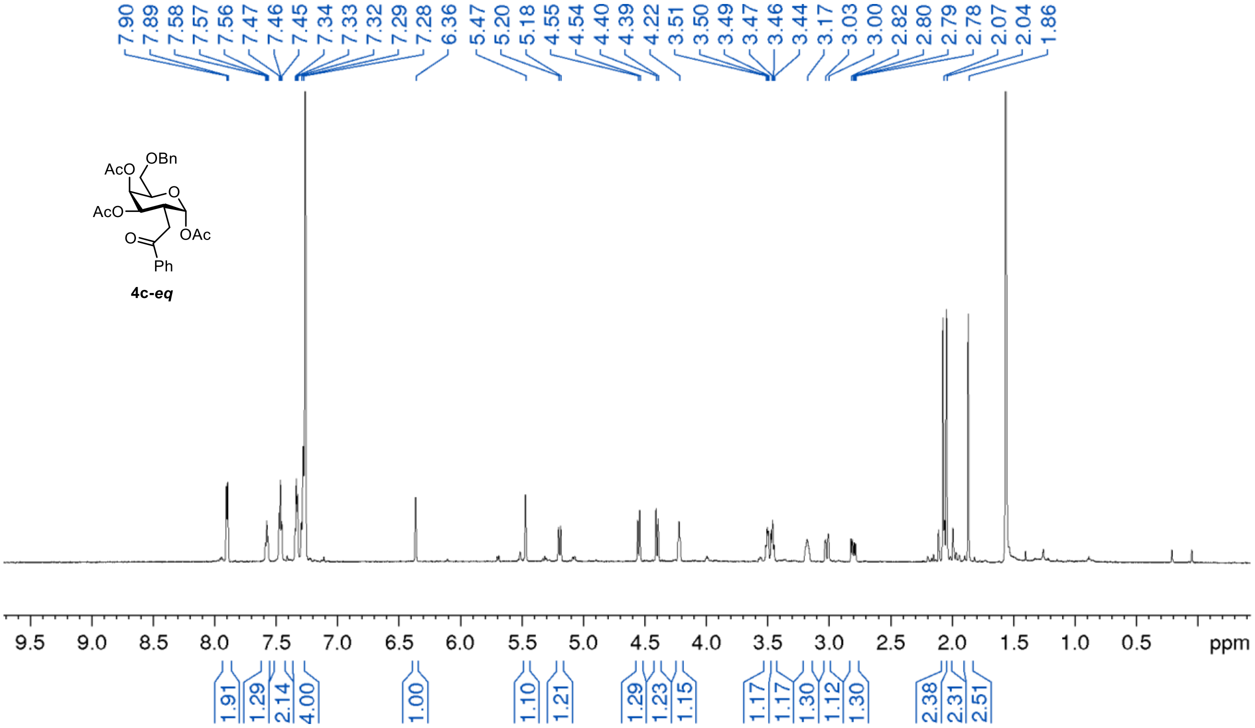

^{13}C NMR (175 MHz, CDCl$_3$, 25 °C) of (4c-*eq*)

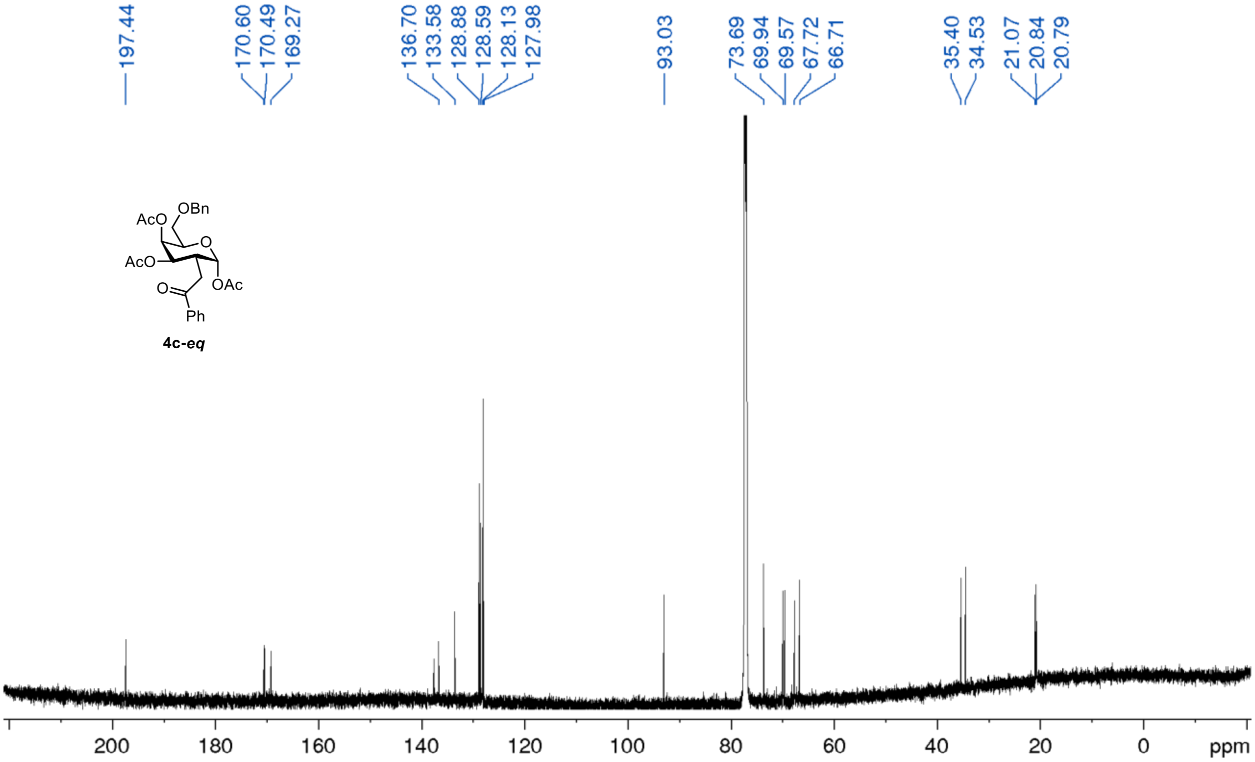

^{1}H NMR (500 MHz, CDCl$_3$, 25 °C) of (4d-*ax*)

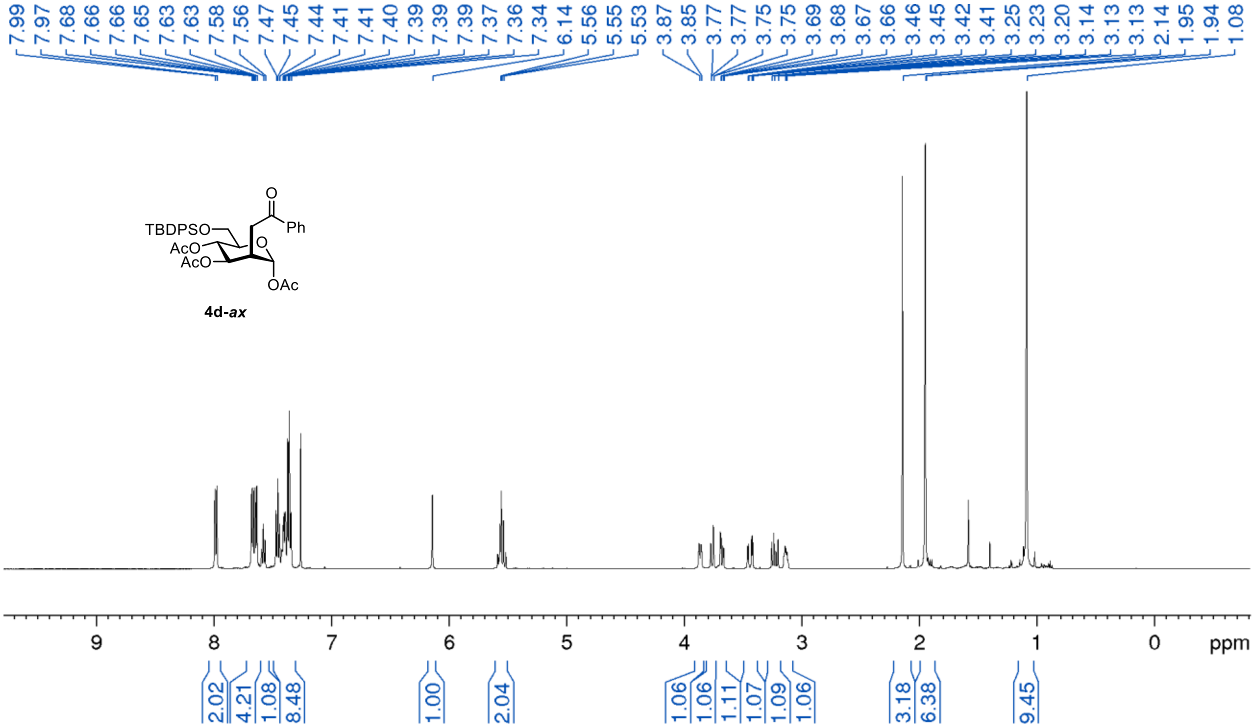

^{13}C NMR (125 MHz, CDCl$_3$, 25 °C) of (4d-*ax*)

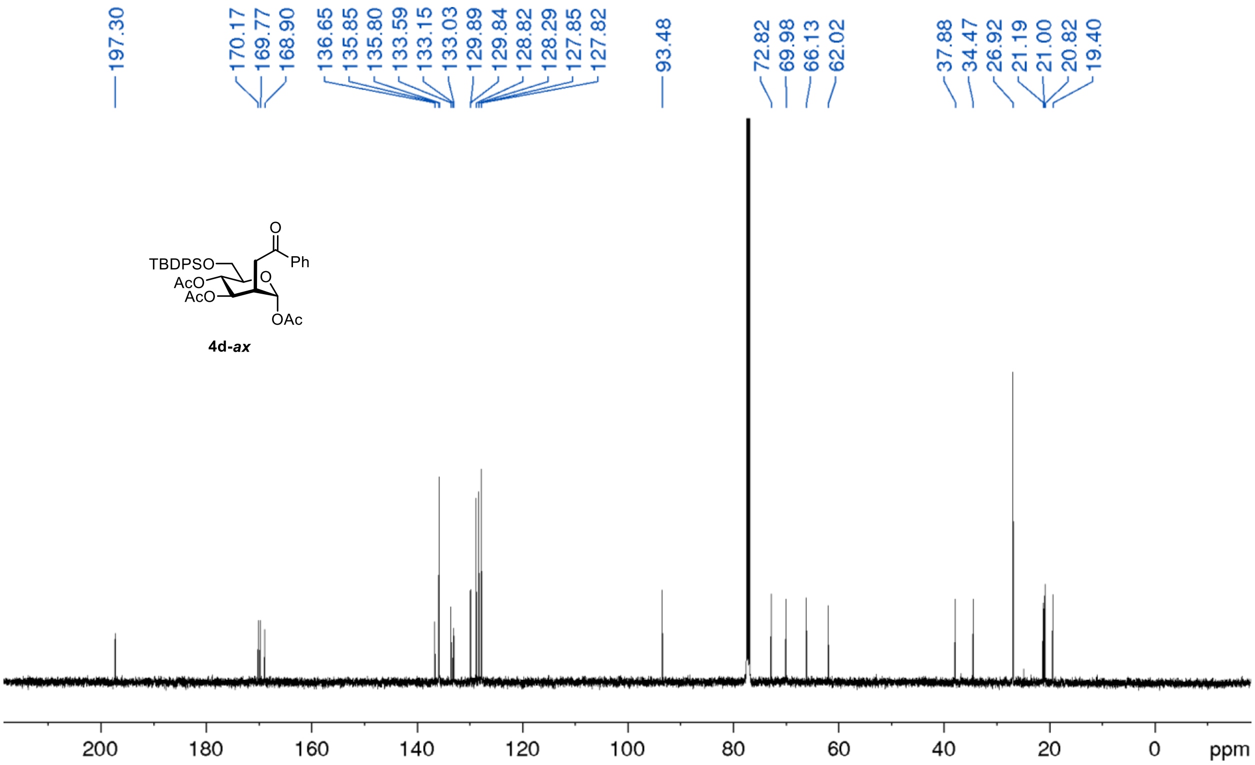

^{1}H NMR (500 MHz, $CDCl_3$, 25 °C) of (4d-*eq*)

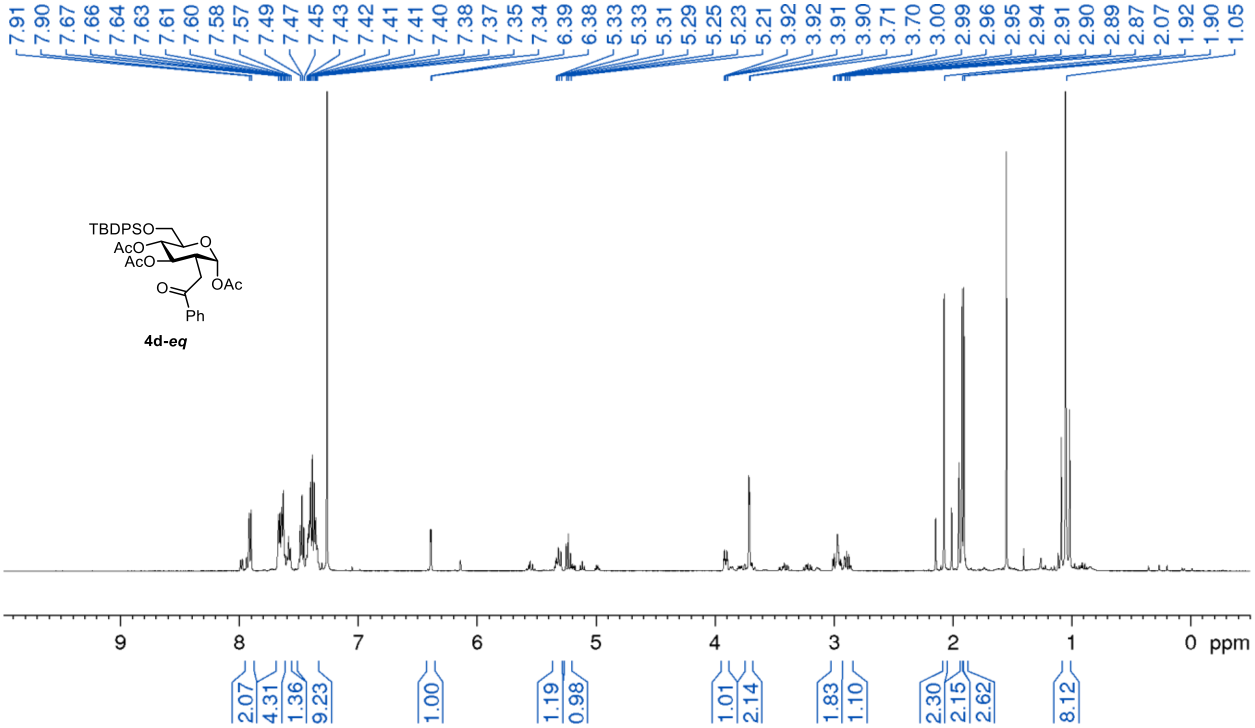

^{13}C NMR (125 MHz, $CDCl_3$, 25 °C) of (4d-*eq*)

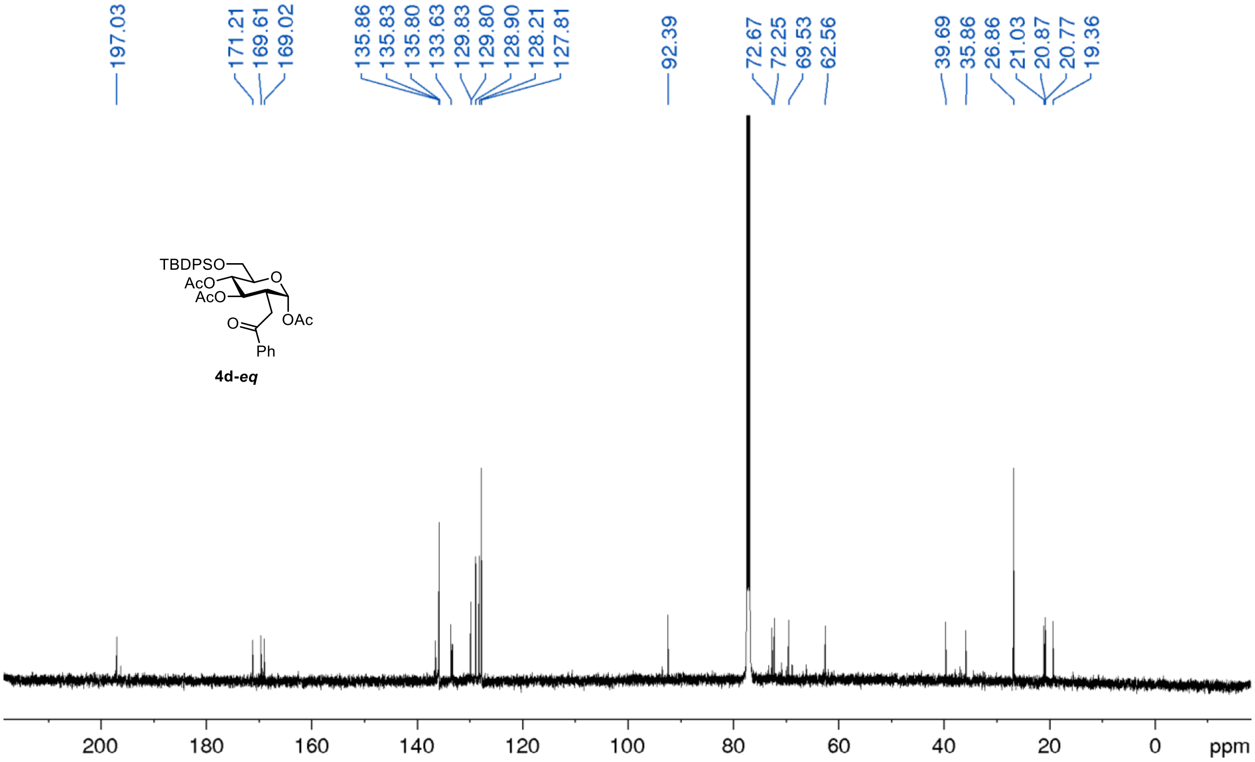

^{1}H NMR (500 MHz, CDCl$_3$, 25 °C) of (4e-*ax*)

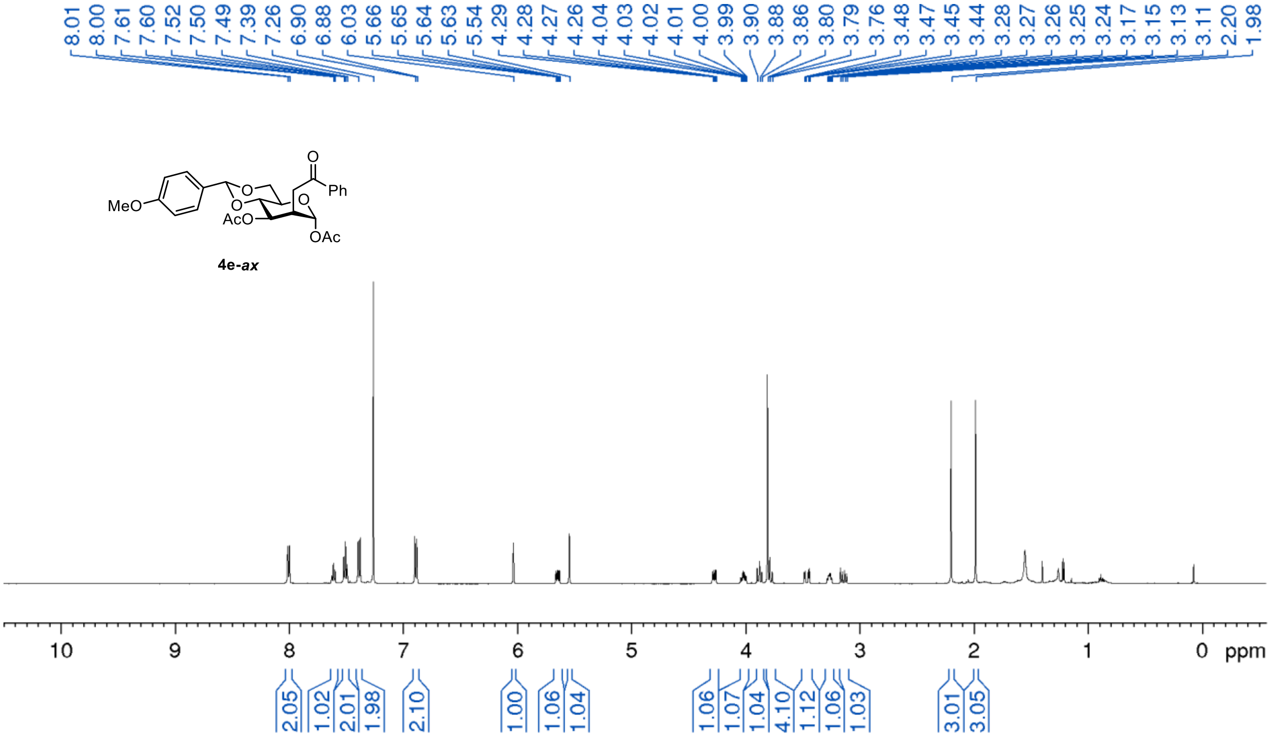

^{13}C NMR (125 MHz, CDCl$_3$, 25 °C) of (4e-*ax*)

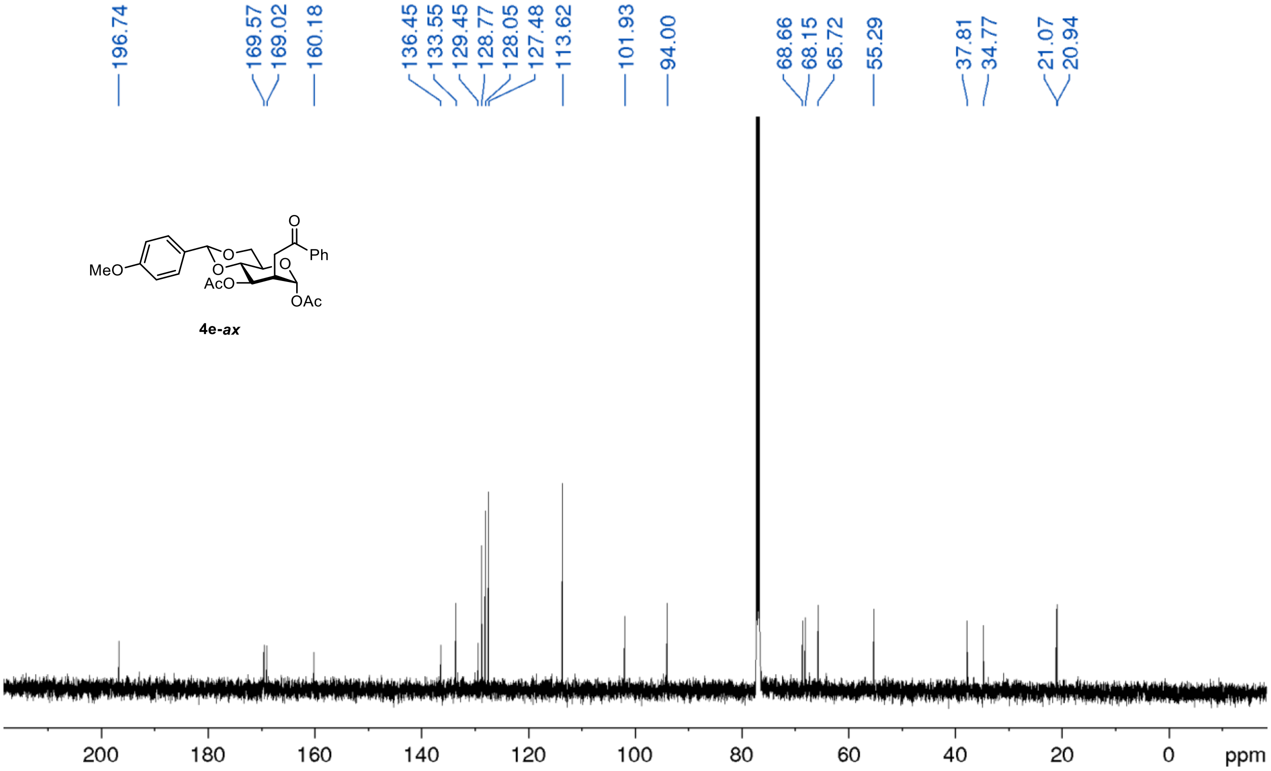

^{1}H NMR (700 MHz, $CDCl_3$, 25 °C) of (4f-*ax*)

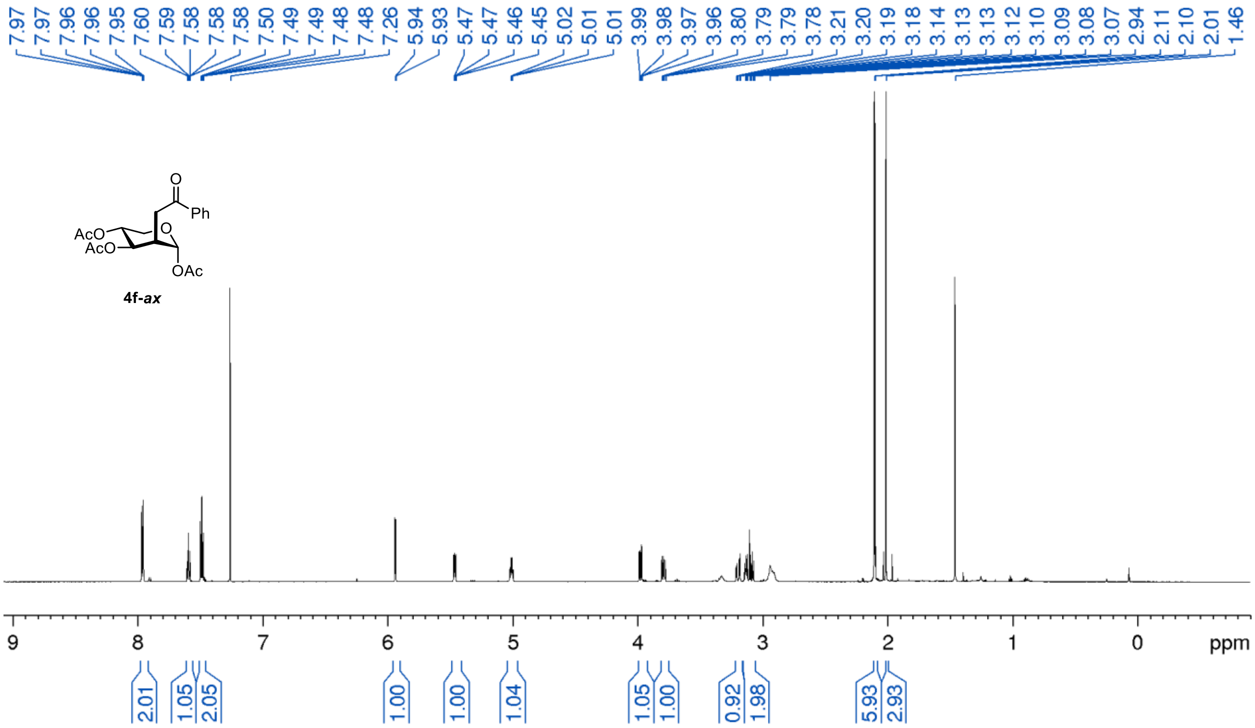

^{13}C NMR (175 MHz, $CDCl_3$, 25 °C) of (4f-*ax*)

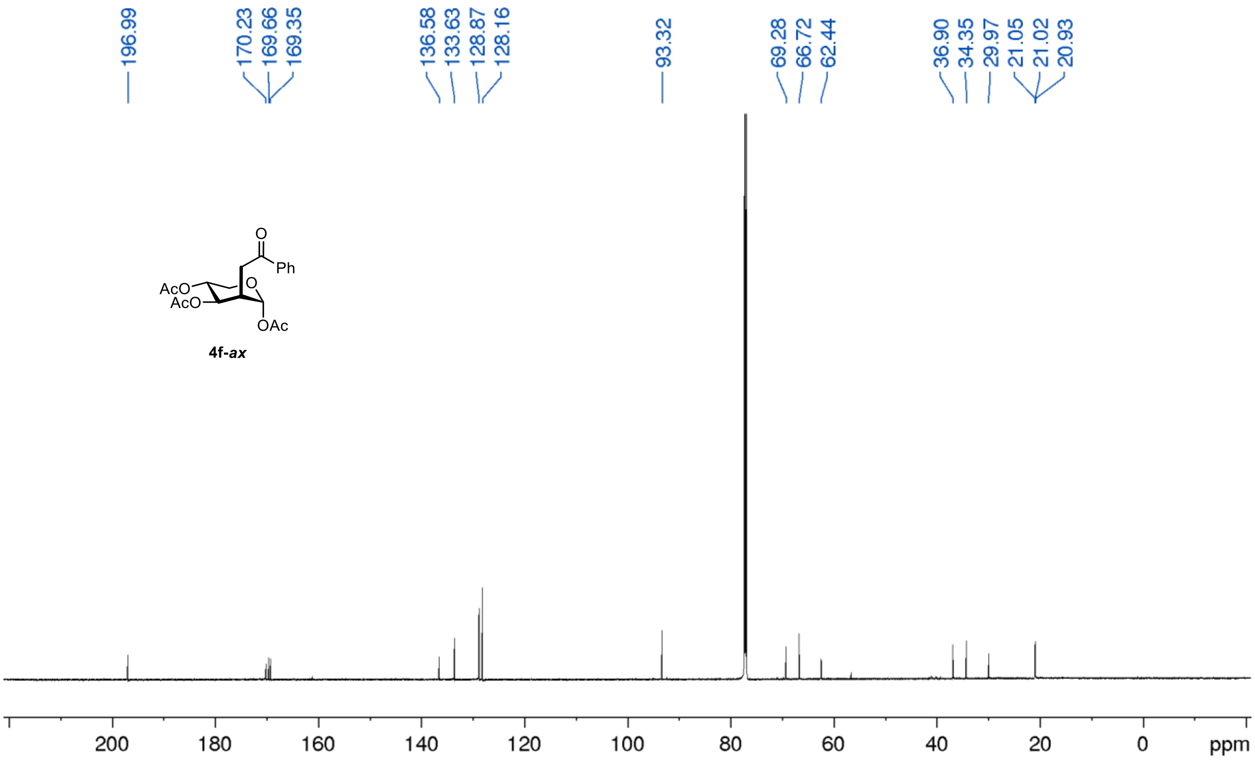

^{1}H NMR (700 MHz, CDCl$_3$, 25 °C) of (4f-*eq*)

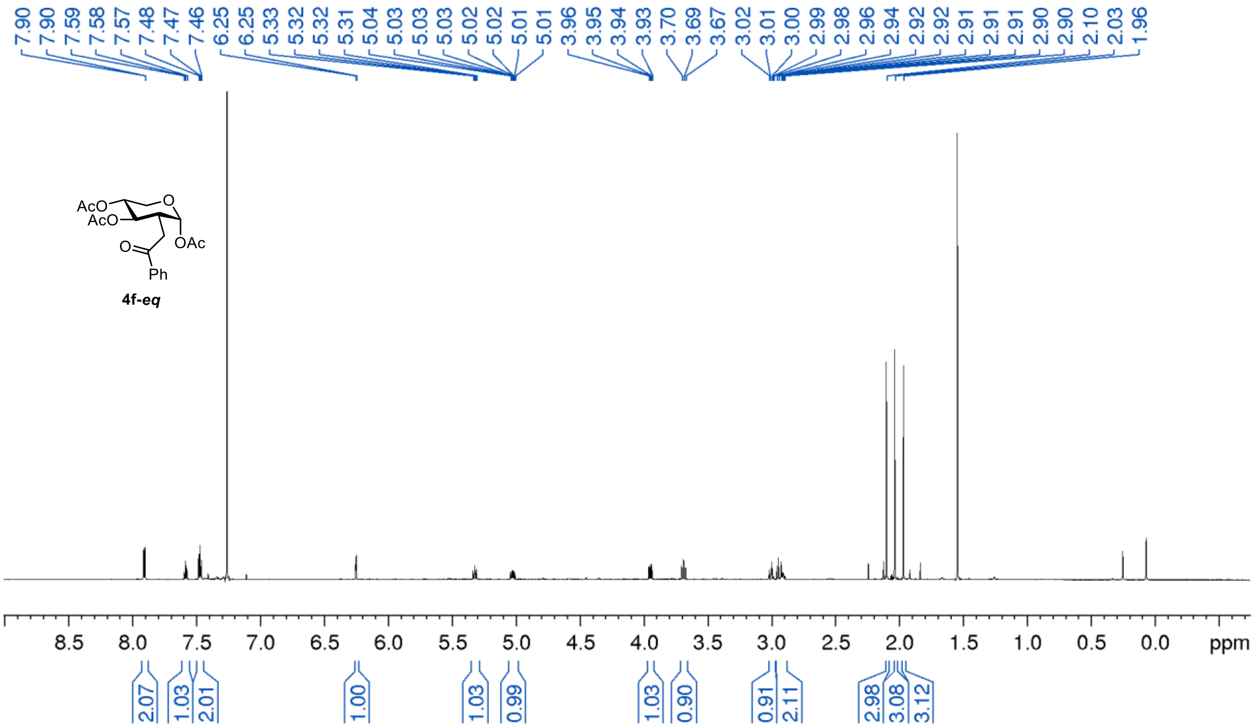

^{13}C NMR (175 MHz, CDCl$_3$, 25 °C) of (4f-*eq*)

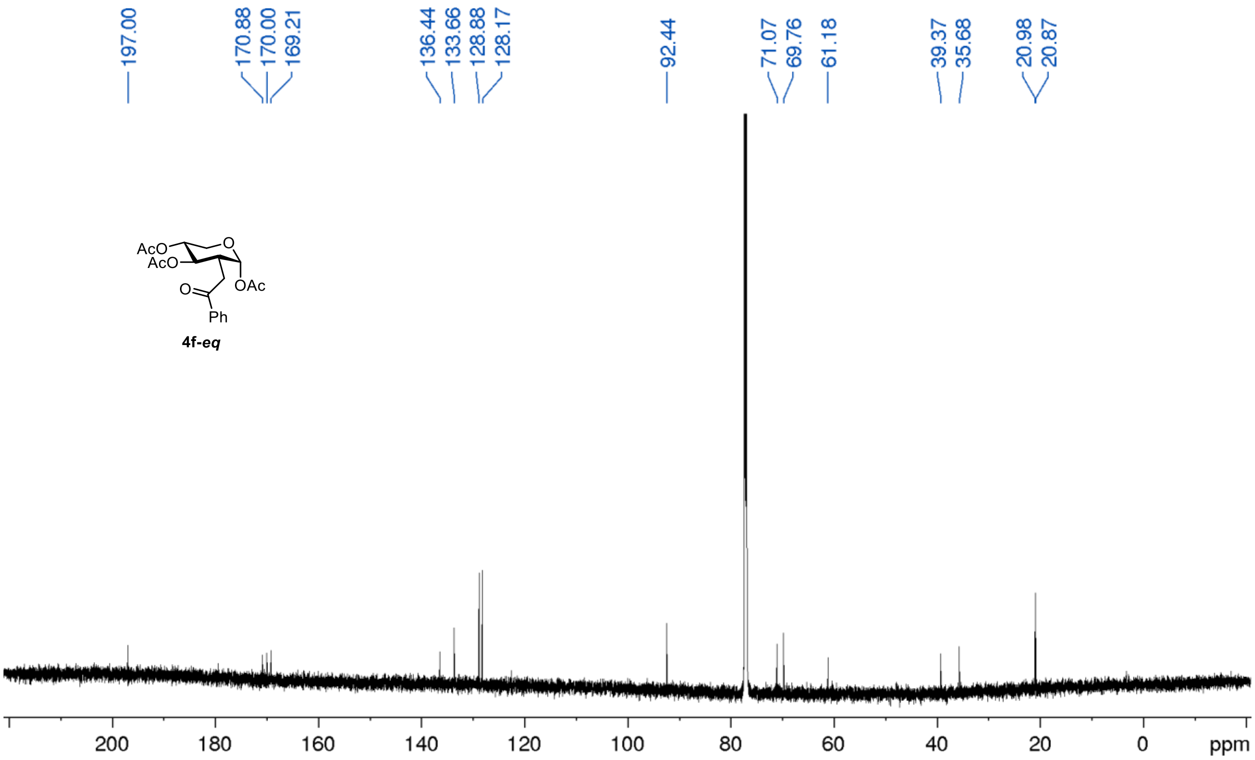

^{1}H NMR (500 MHz, $CDCl_3$, 25 °C) of (4g)

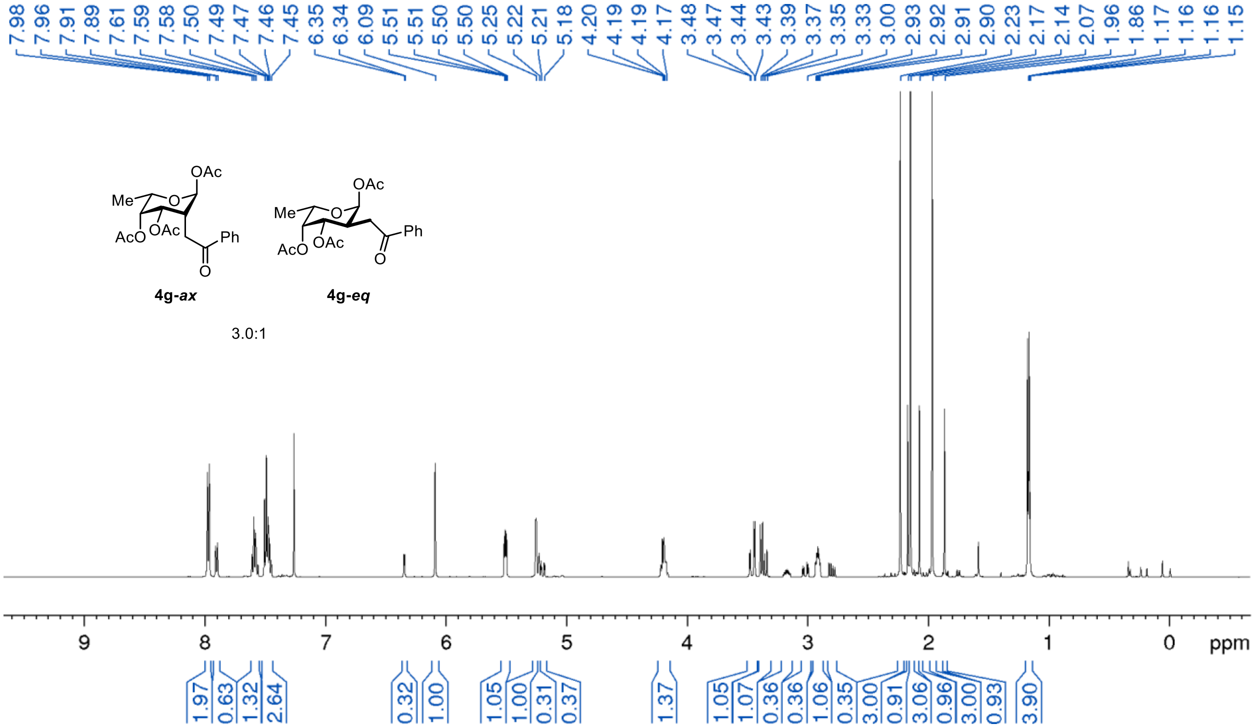

^{13}C NMR (125 MHz, $CDCl_3$, 25 °C) of (4g)

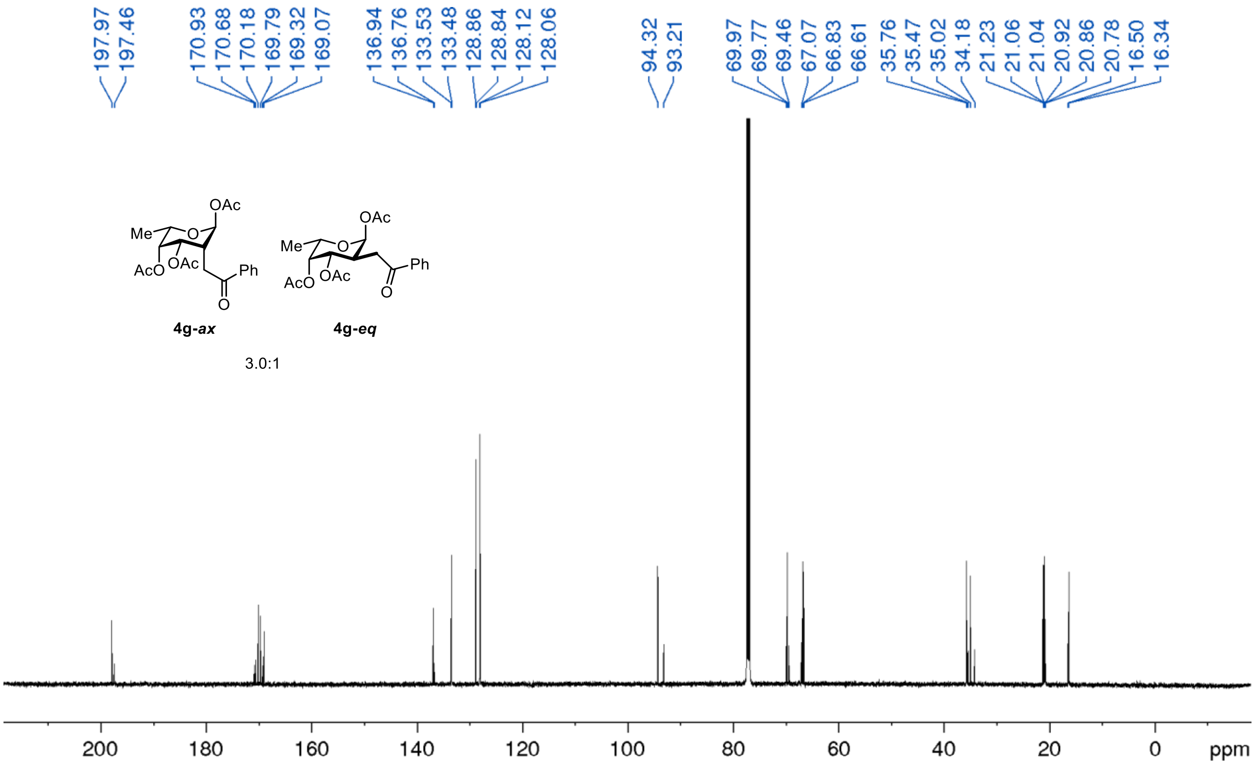

^{1}H NMR (500 MHz, CDCl$_3$, 25 °C) of (4h-*ax*)

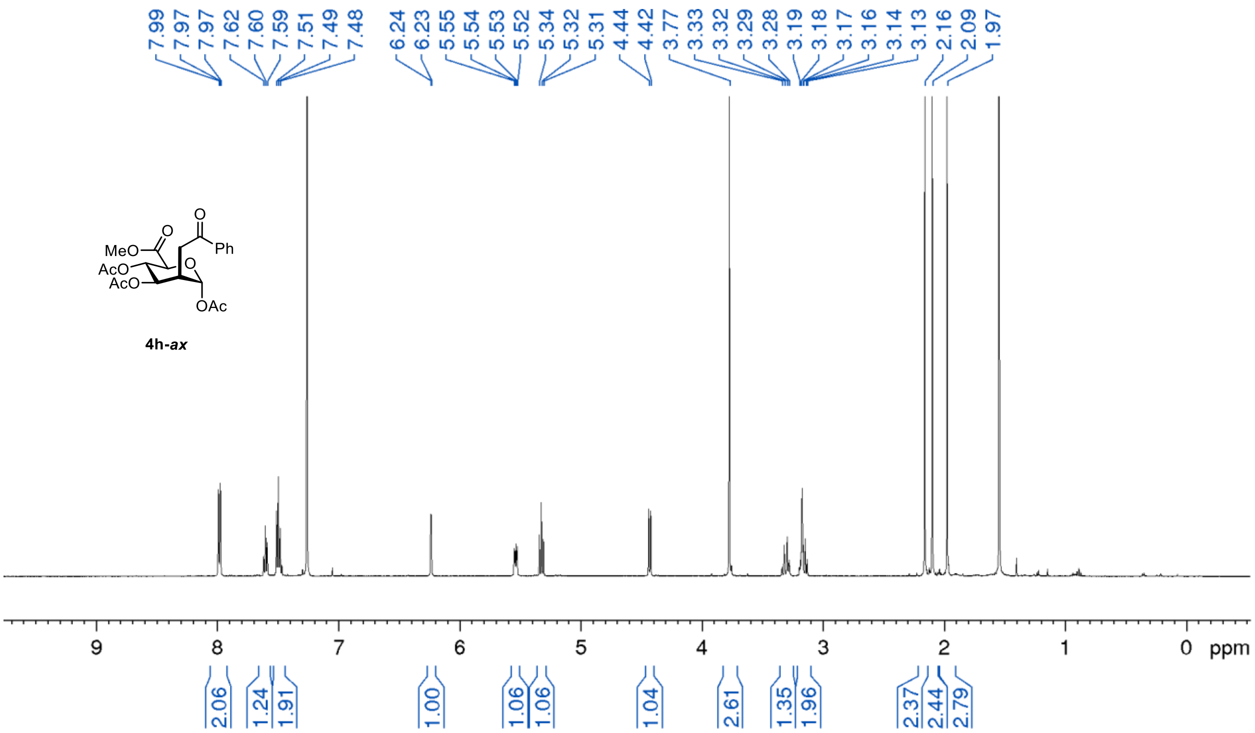

^{13}C NMR (125 MHz, CDCl$_3$, 25 °C) of (4h-*ax*)

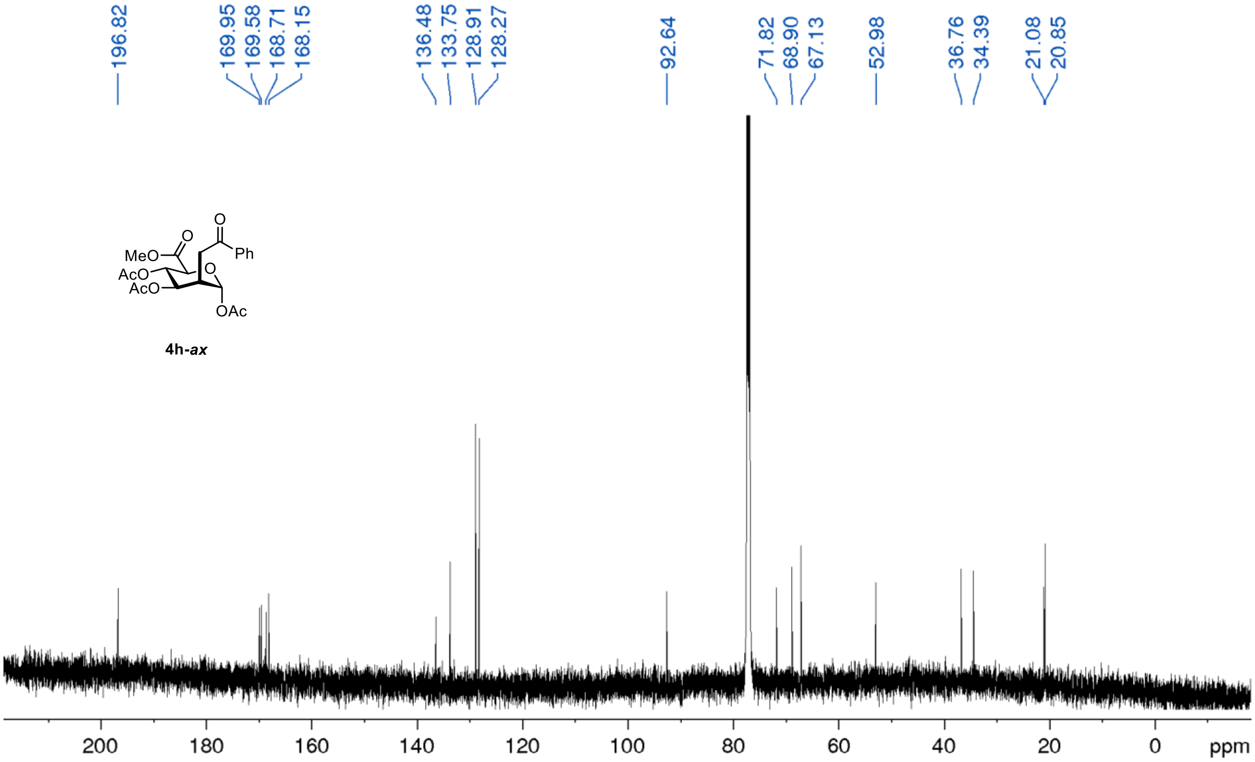

^{1}H NMR (500 MHz, CDCl$_3$, 25 °C) of (4i-*ax*)

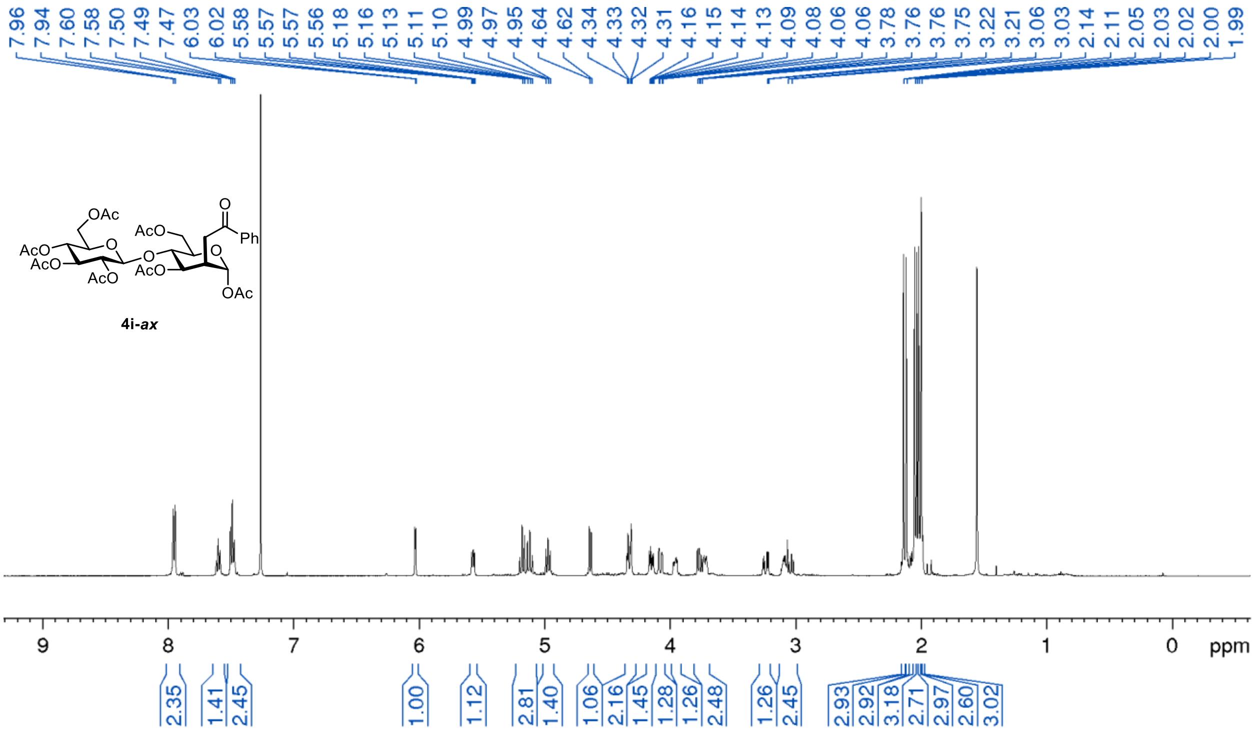

^{13}C NMR (125 MHz, CDCl$_3$, 25 °C) of (4i-*ax*)

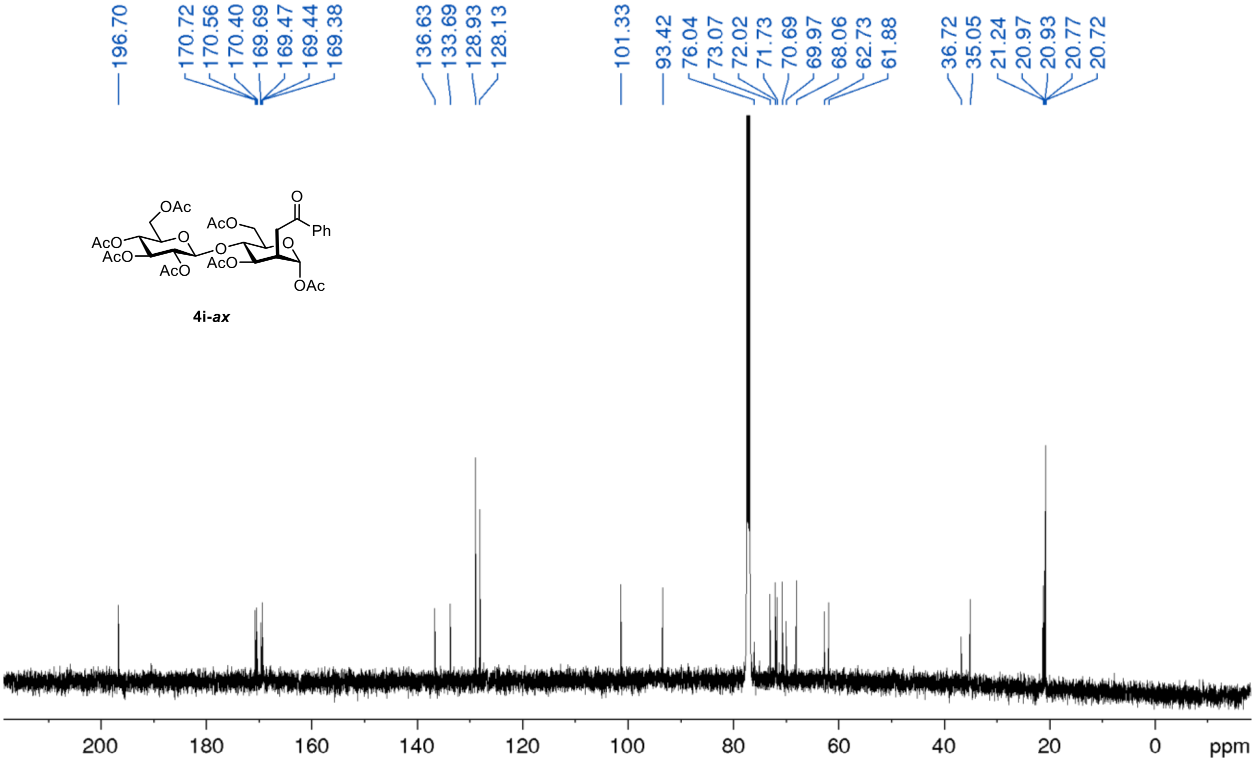

^{1}H NMR (500 MHz, $CDCl_3$, 25 ºC) of (4j-*ax*)

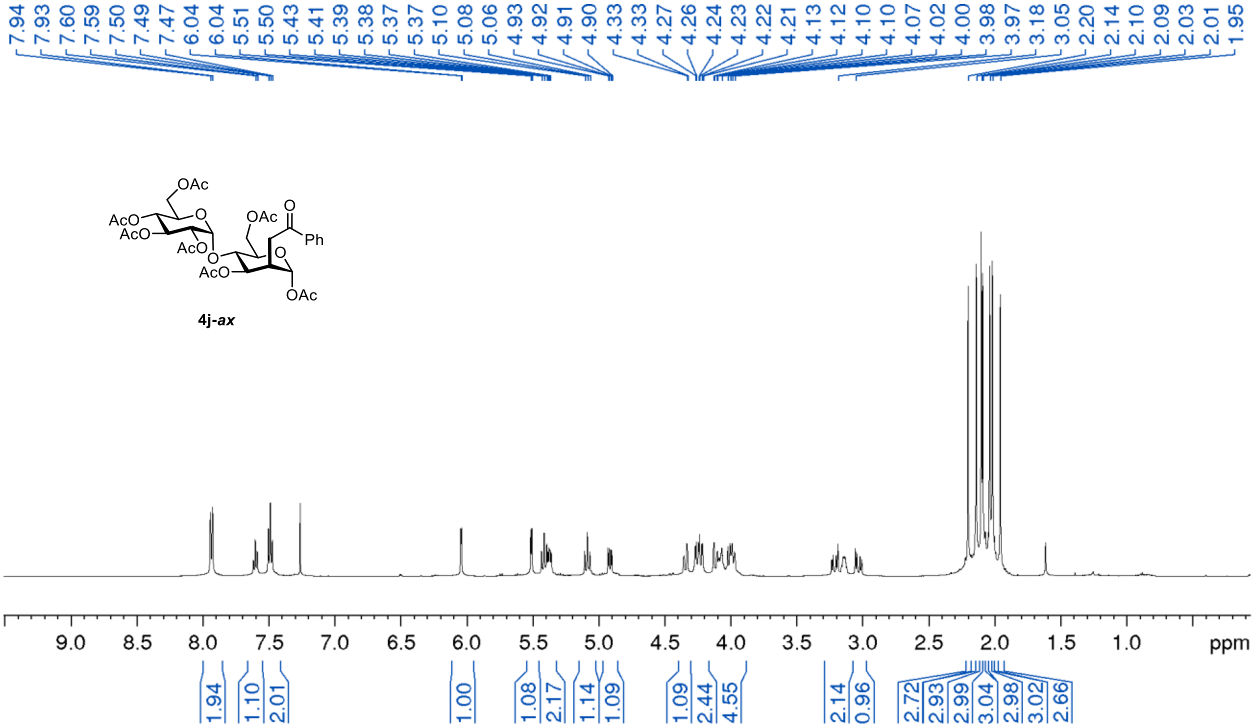

^{13}C NMR (125 MHz, $CDCl_3$, 25 ºC) of (4j-*ax*)

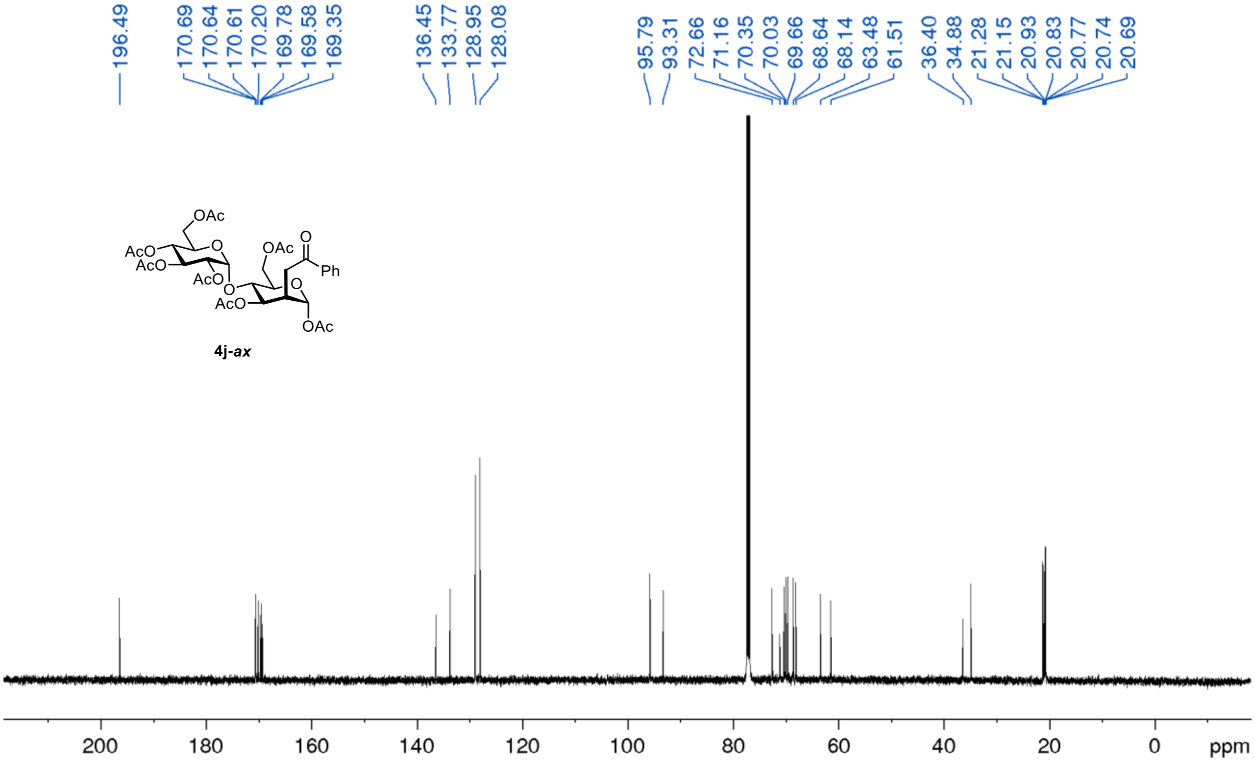

^{1}H NMR (700 MHz, $CDCl_3$, 25 °C) of (4k-*ax*)

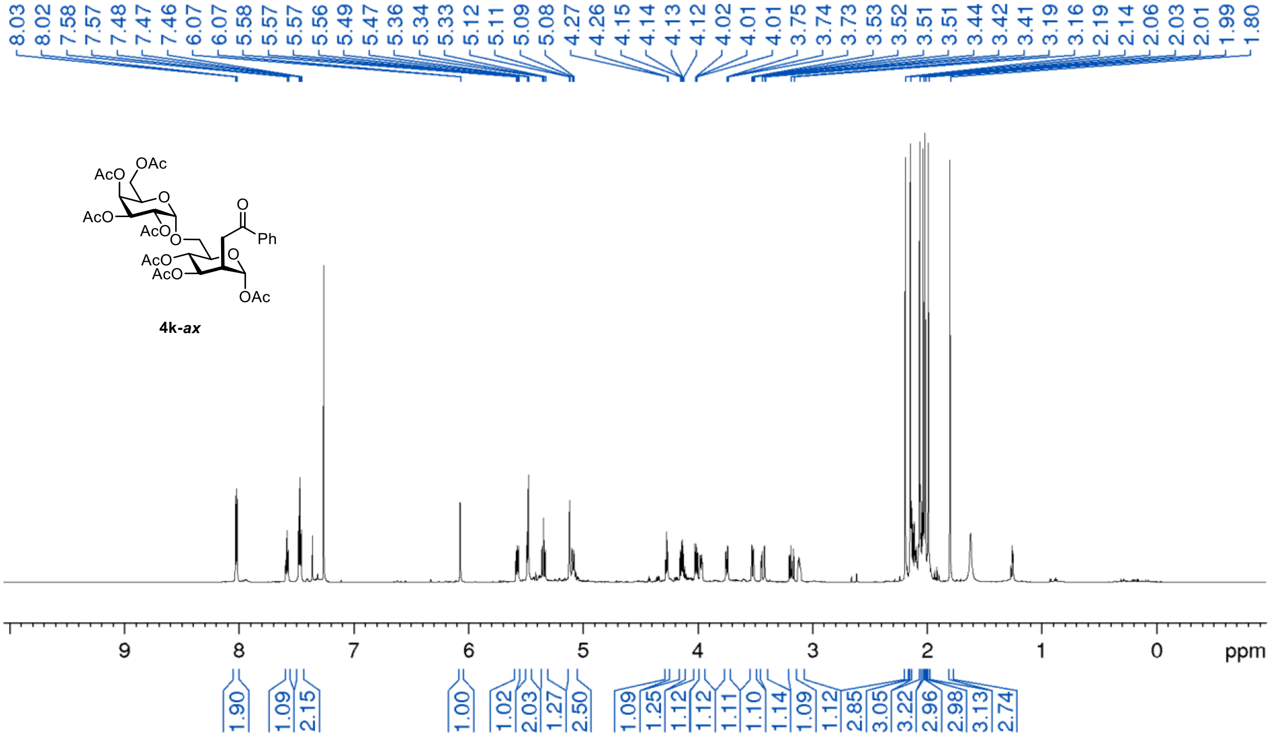

^{13}C NMR (175 MHz, $CDCl_3$, 25 °C) of (4k-*ax*)

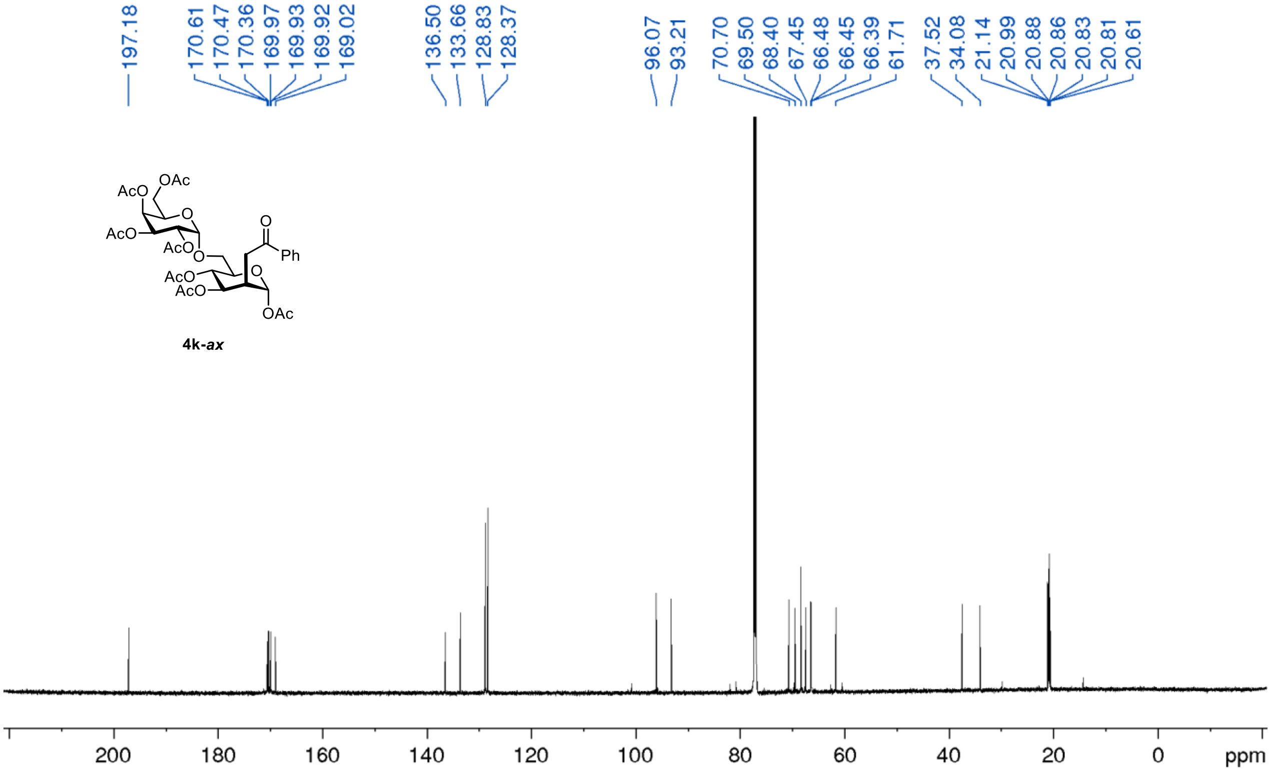

^{1}H NMR (500 MHz, CDCl$_3$, 25 °C) of (4l-*ax*)

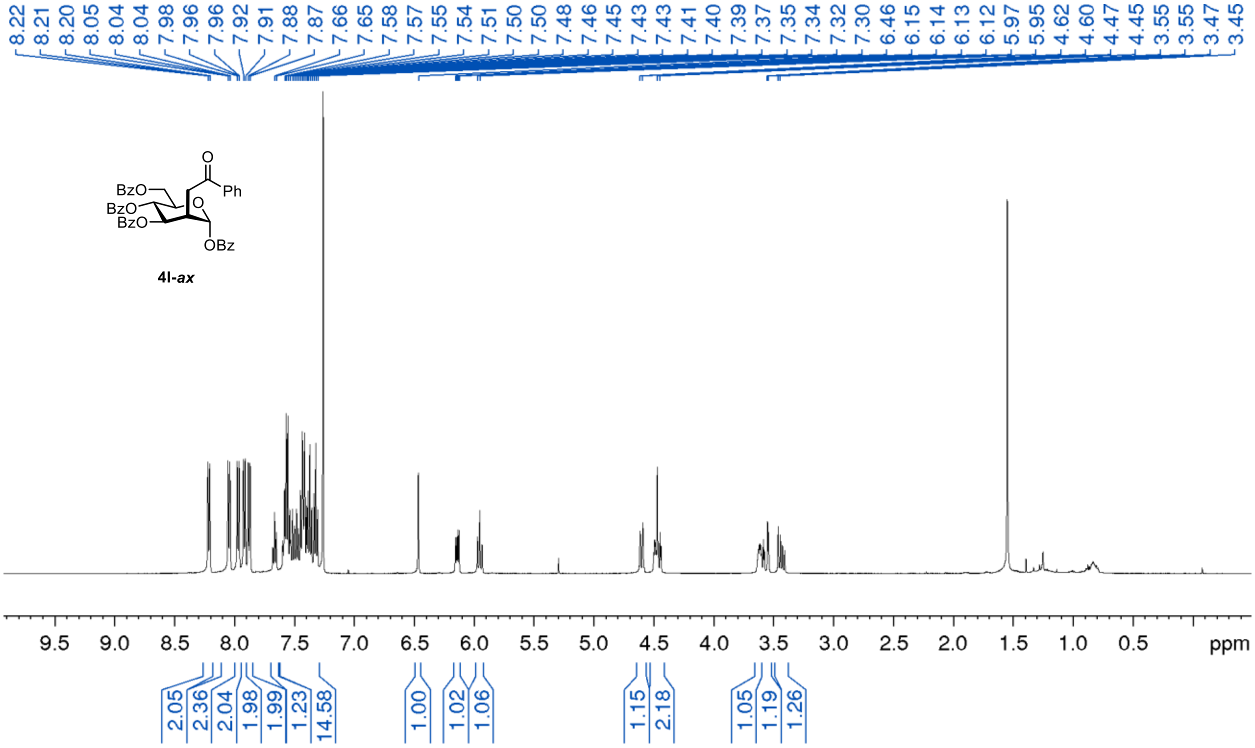

^{13}C NMR (125 MHz, CDCl$_3$, 25 °C) of (4l-*ax*)

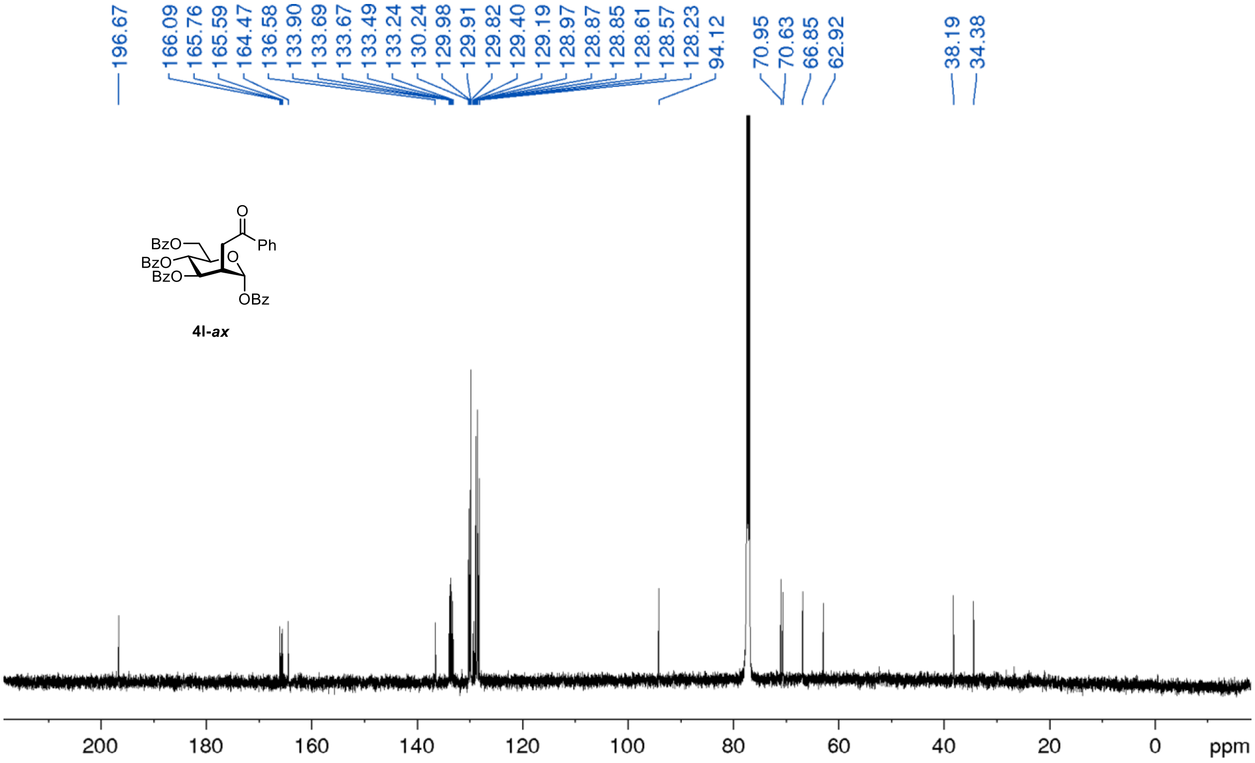

^{1}H NMR (700 MHz, $CDCl_3$, 25 °C) of (4l-*eq*)

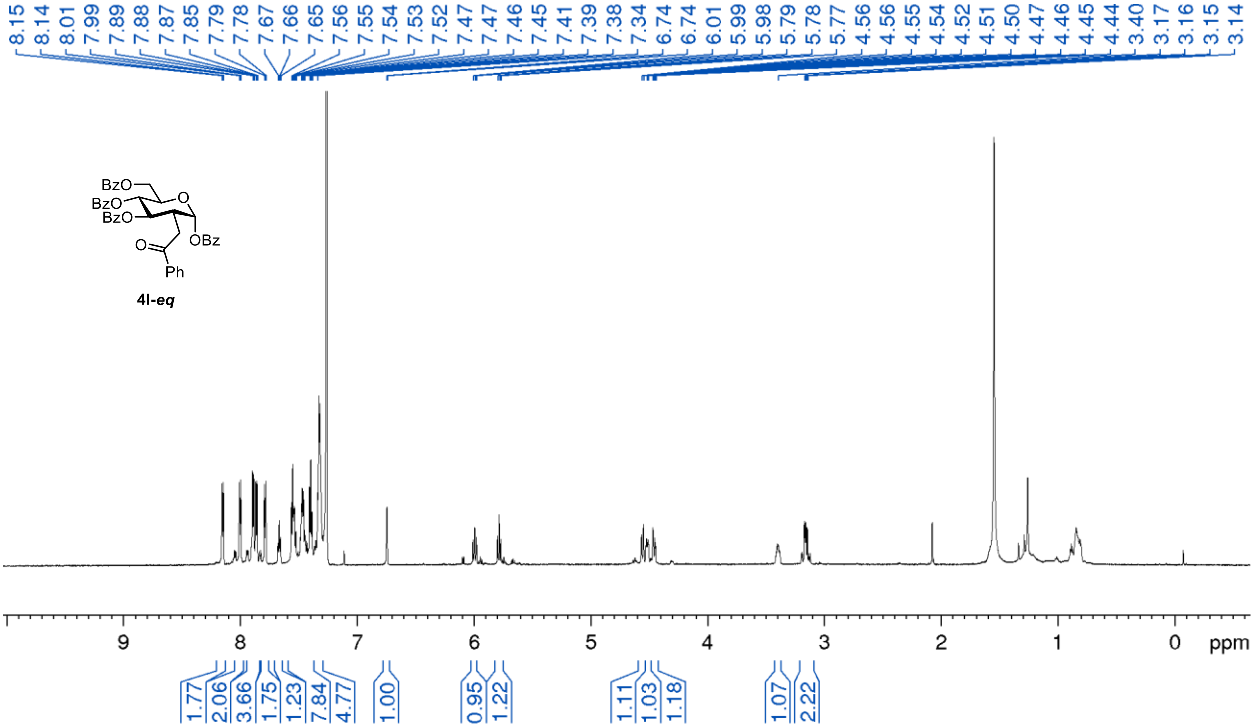

^{13}C NMR (175 MHz, $CDCl_3$, 25 °C) of (4l-*eq*)

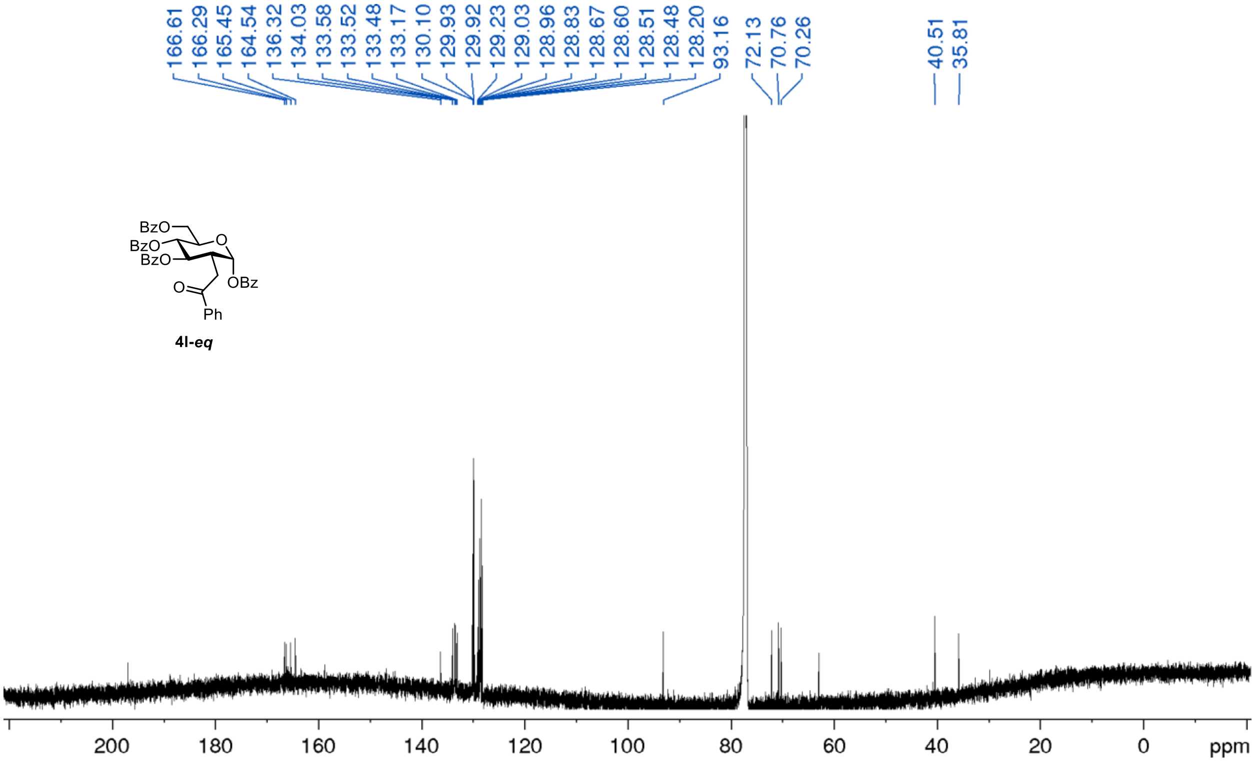

^{1}H NMR (500 MHz, CDCl$_3$, 25 °C) of (4m-*ax*)

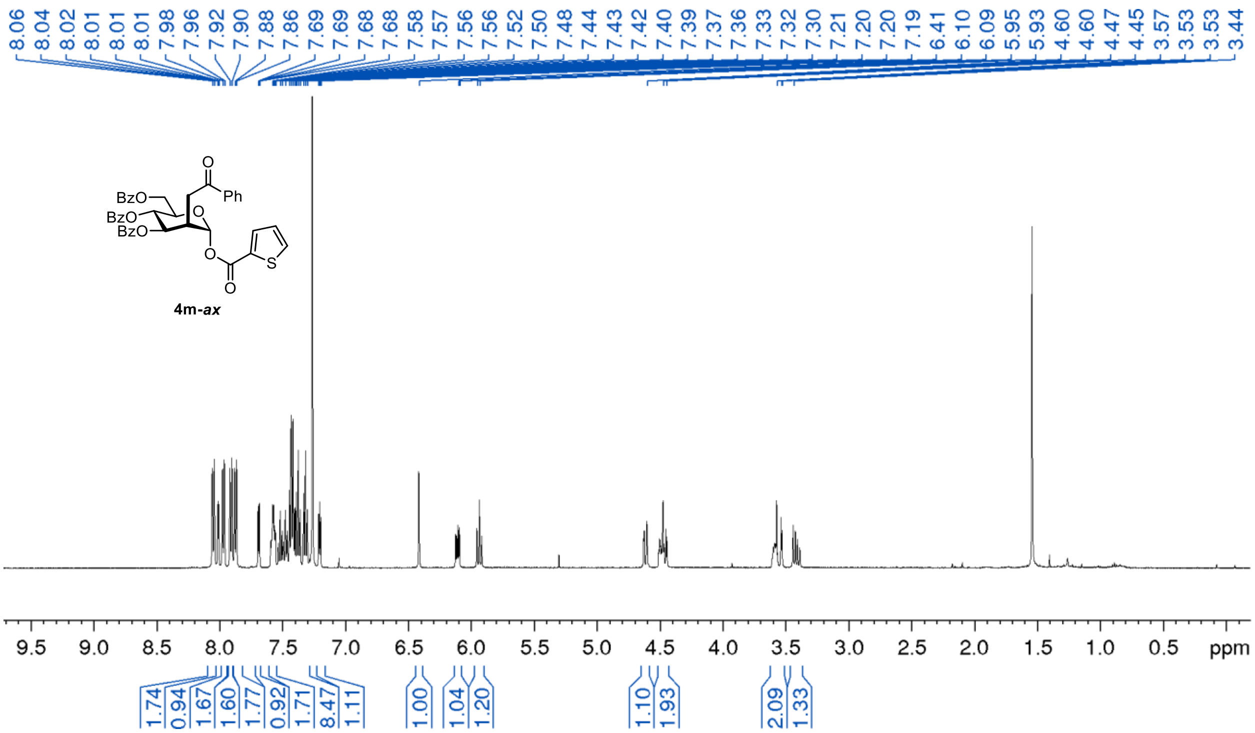

^{13}C NMR (125 MHz, CDCl$_3$, 25 °C) of (4m-*ax*)

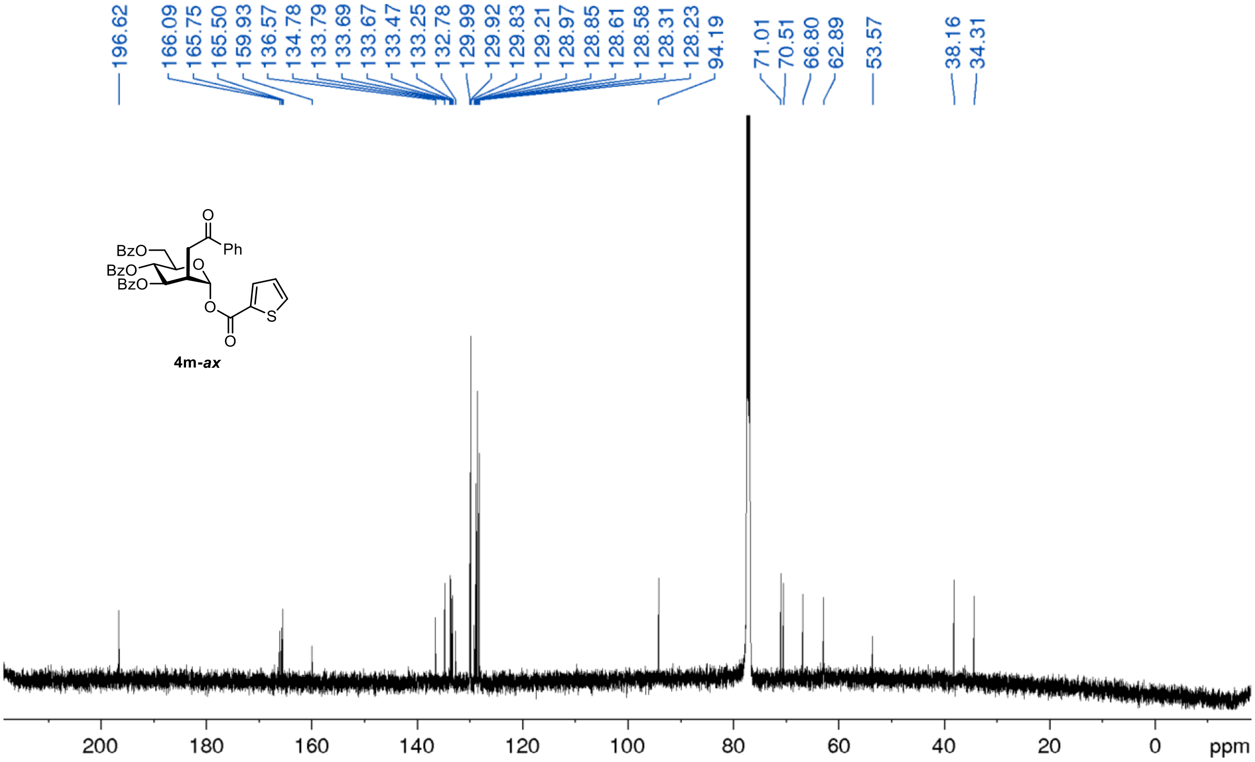

^{1}H NMR (500 MHz, $CDCl_3$, 25 °C) of (4n-*ax*)

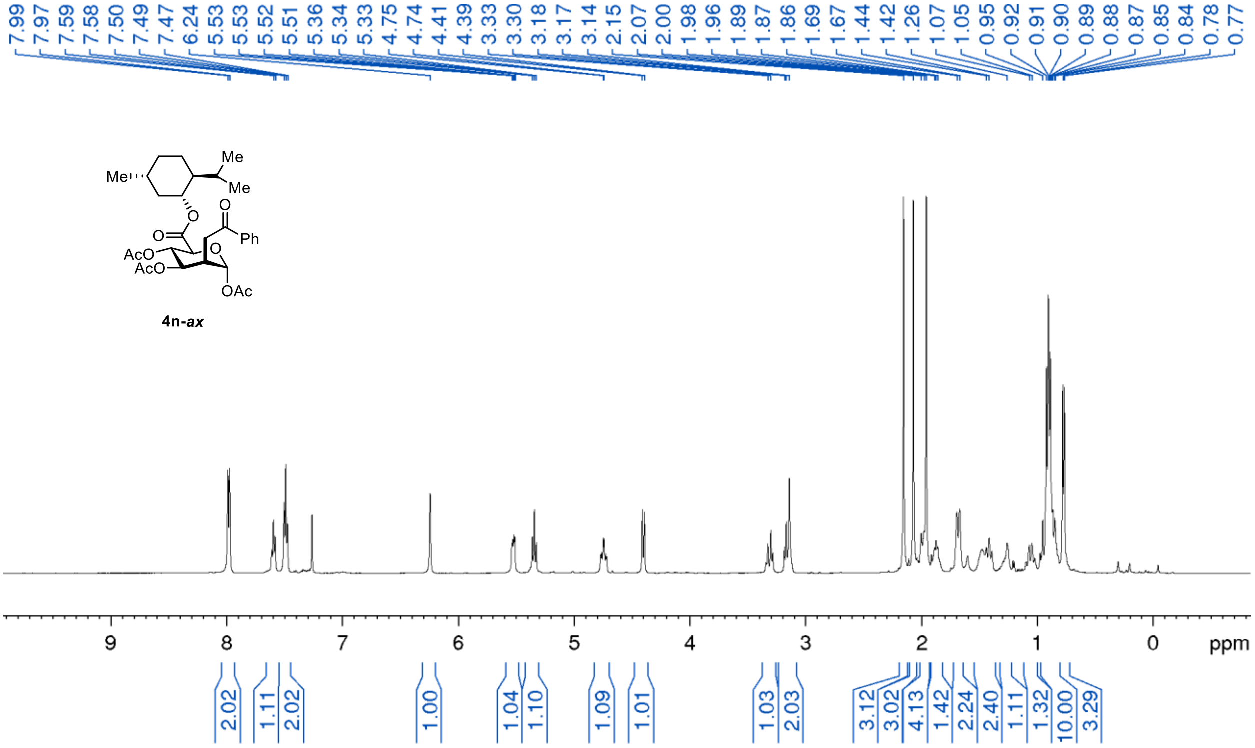

^{13}C NMR (125 MHz, $CDCl_3$, 25 °C) of (4n-*ax*)

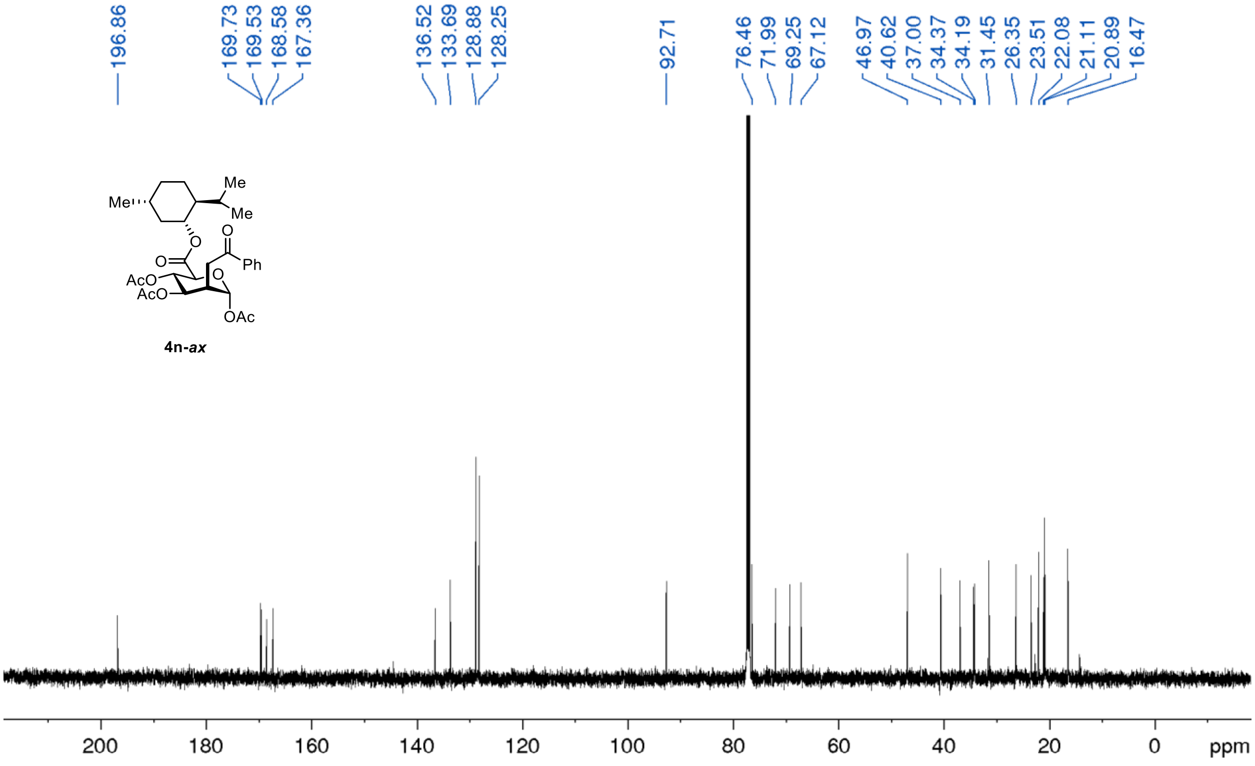

^{1}H NMR (700 MHz, CDCl$_3$, 25 °C) of (4o-*ax*)

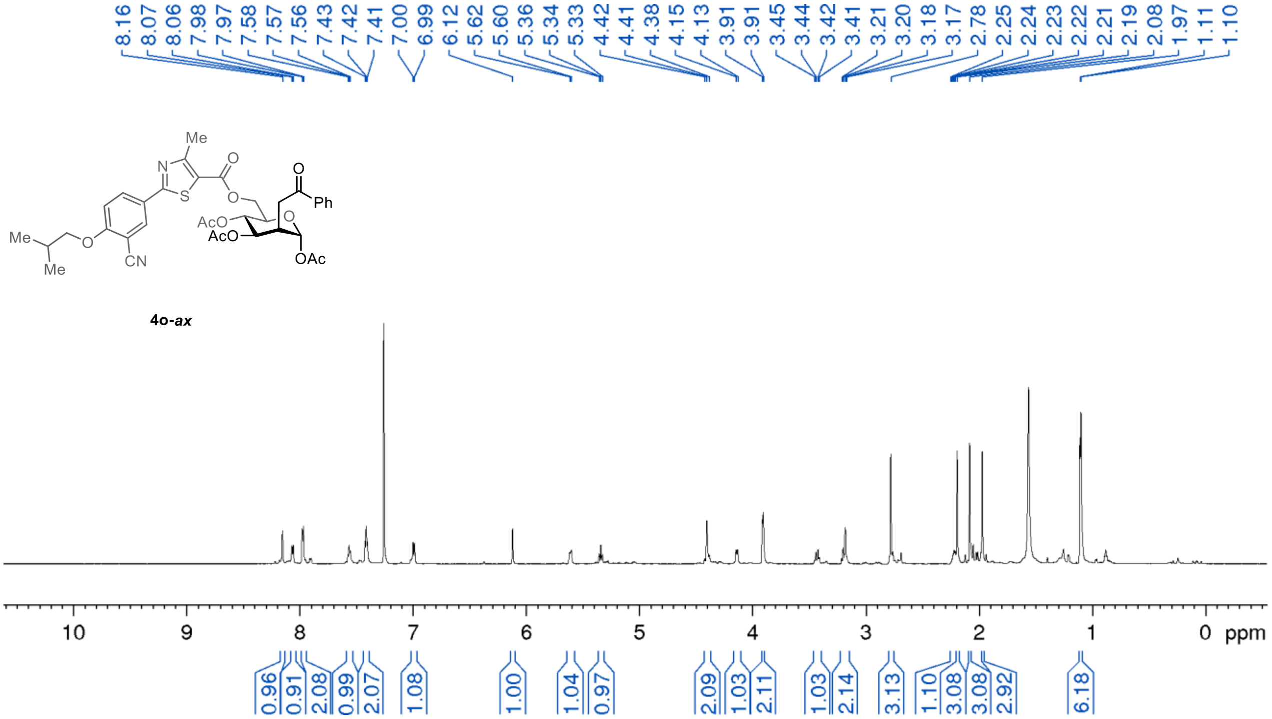

^{13}C NMR (175 MHz, CDCl$_3$, 25 °C) of (4o-*ax*)

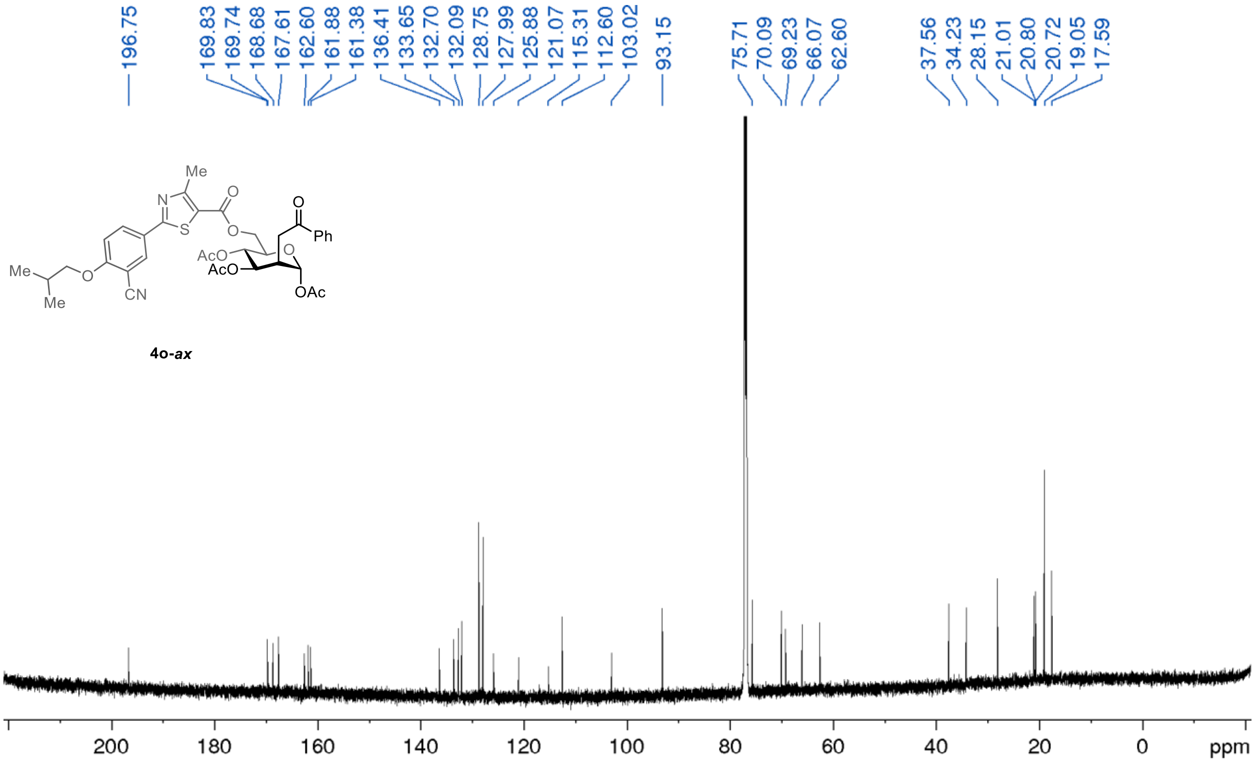

^{1}H NMR (500 MHz, CDCl$_3$, 25 °C) of 4p-*ax*

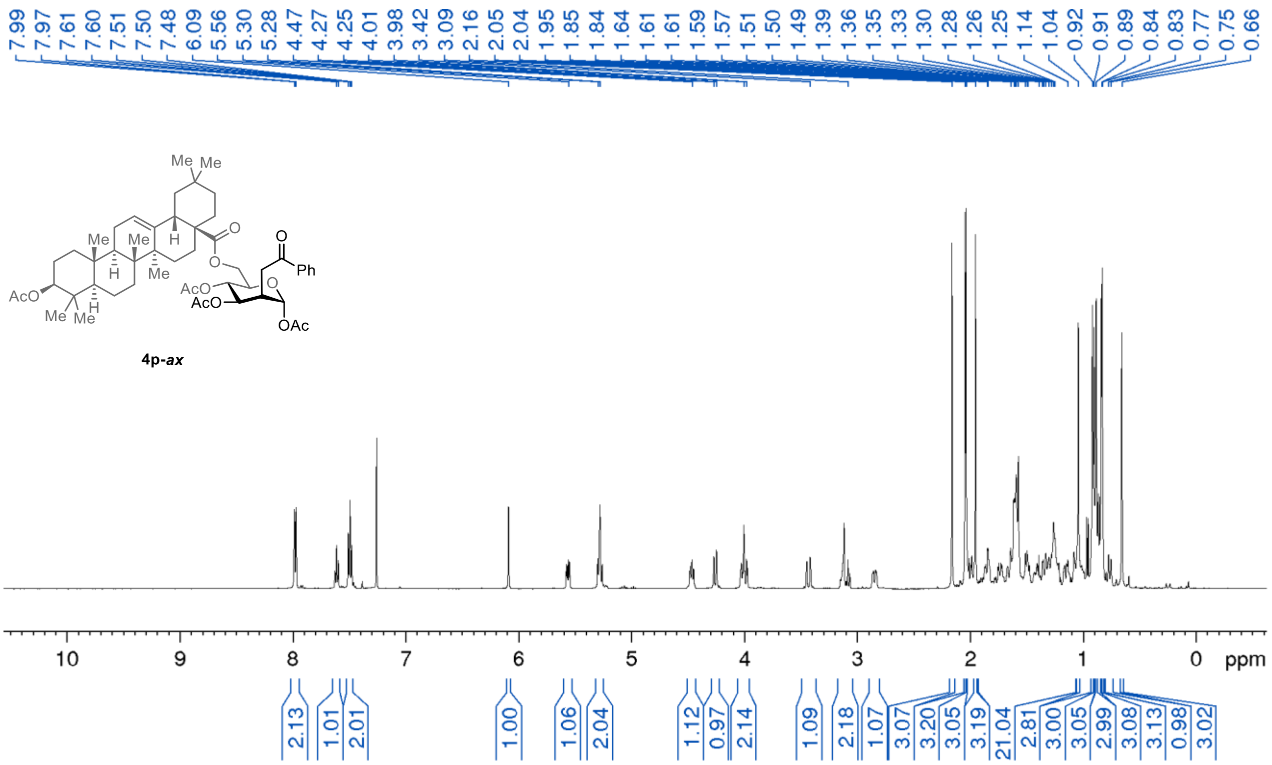

^{13}C NMR (125 MHz, CDCl$_3$, 25 °C) of 4p-*ax*

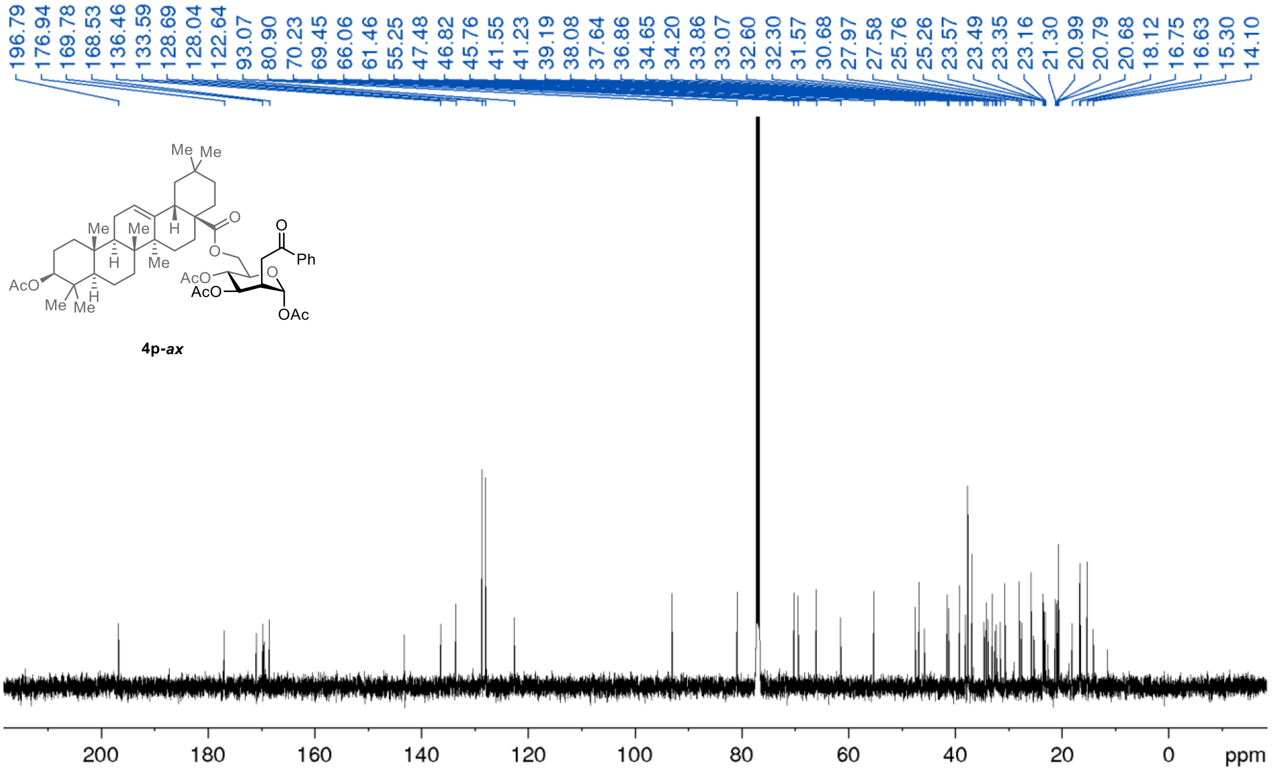

^{1}H NMR (500 MHz, CDCl$_3$, 25 °C) of (4q-*ax*)

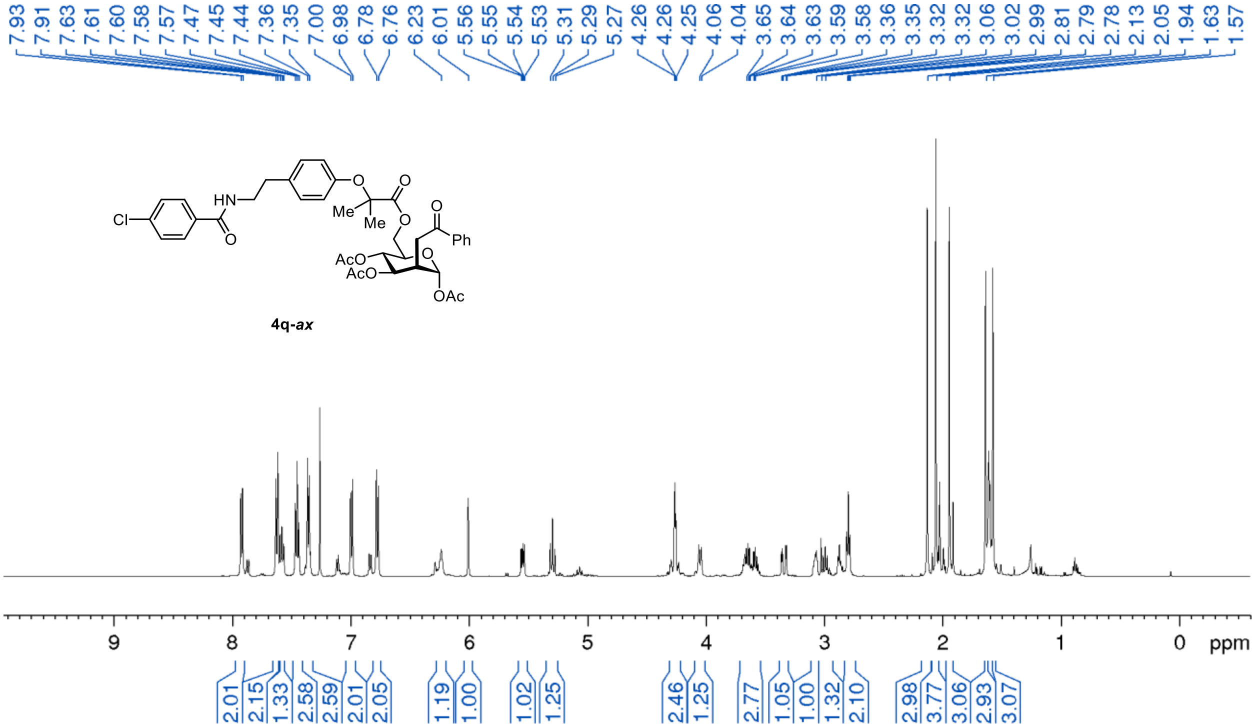

^{13}C NMR (125 MHz, CDCl$_3$, 25 °C) of (4q-*ax*)

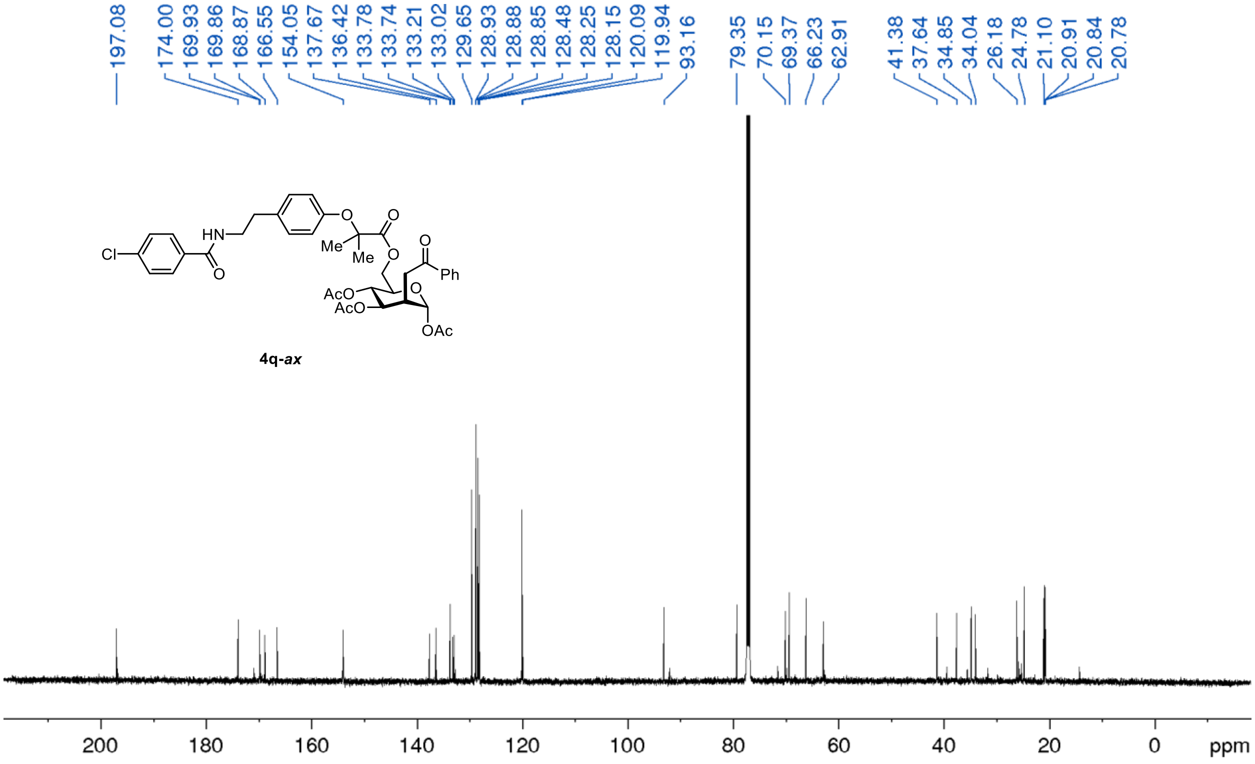

^{1}H NMR (500 MHz, $CDCl_3$, 25 °C) of (4r-*ax*)

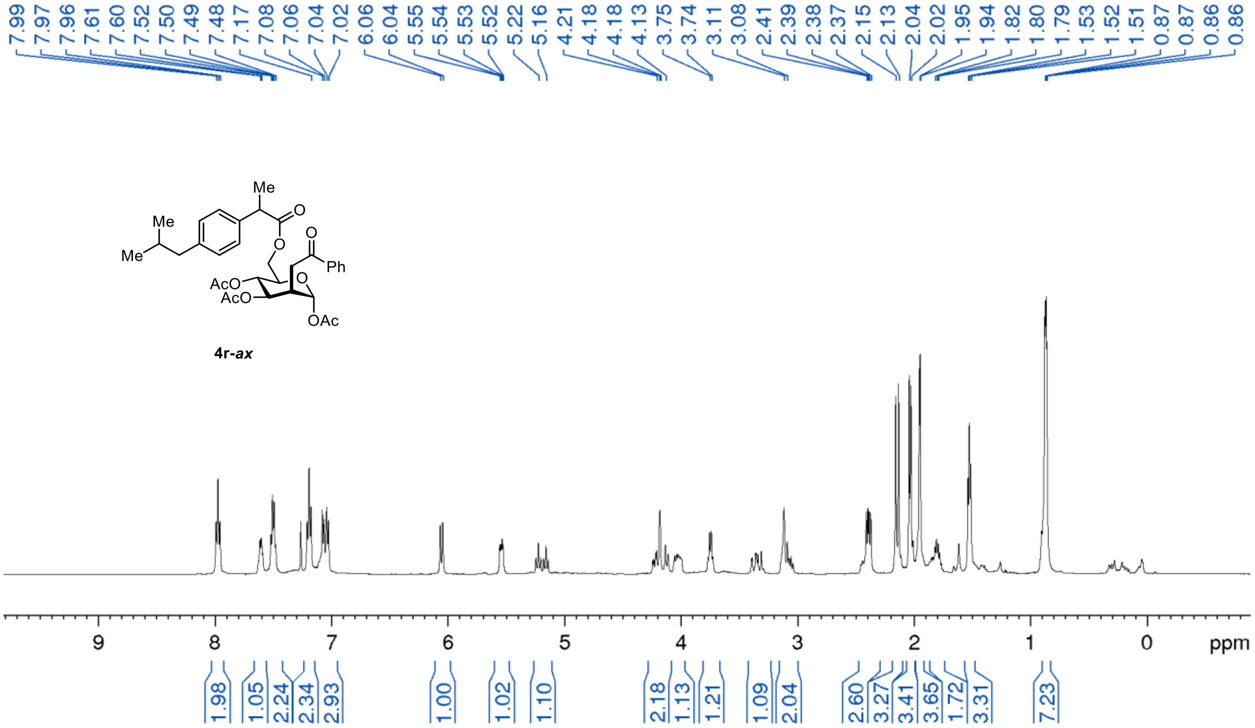

^{13}C NMR (125 MHz, $CDCl_3$, 25 °C) of (4r-*ax*)

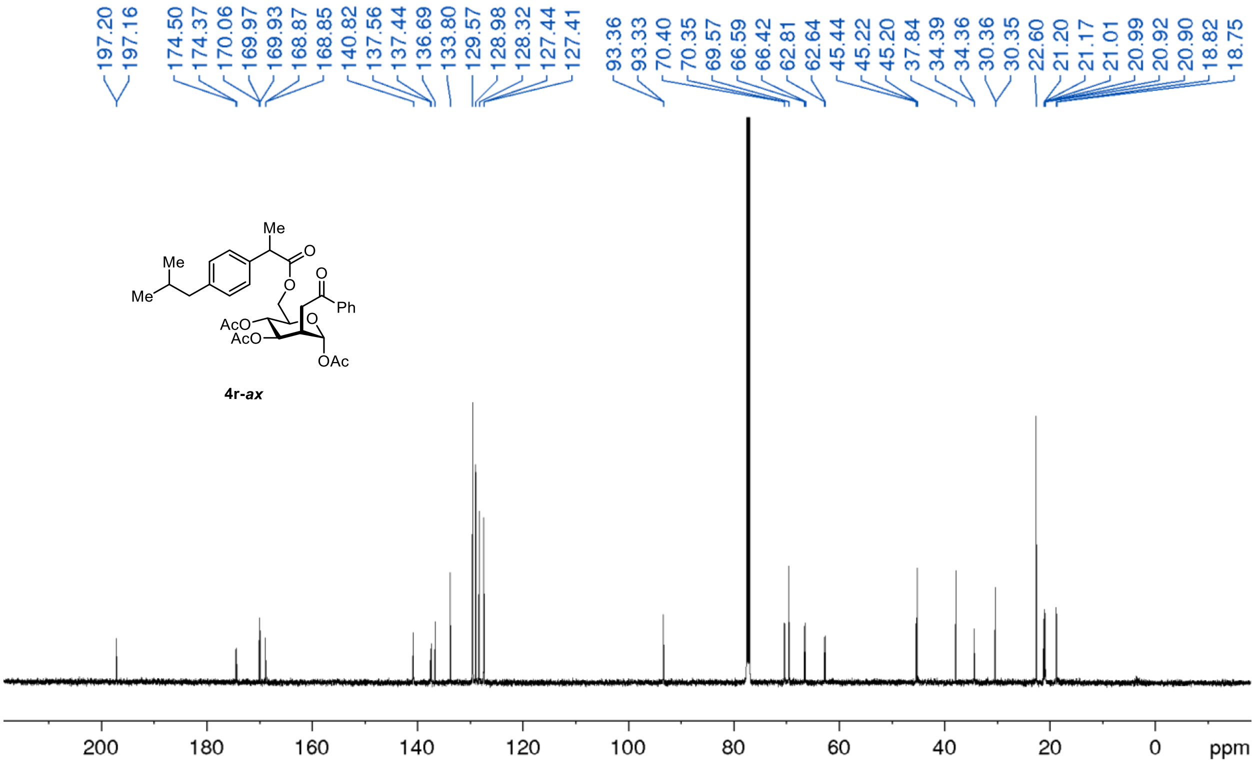

^{1}H NMR (700 MHz, $CDCl_3$, 25 °C) of (4s-*ax*)

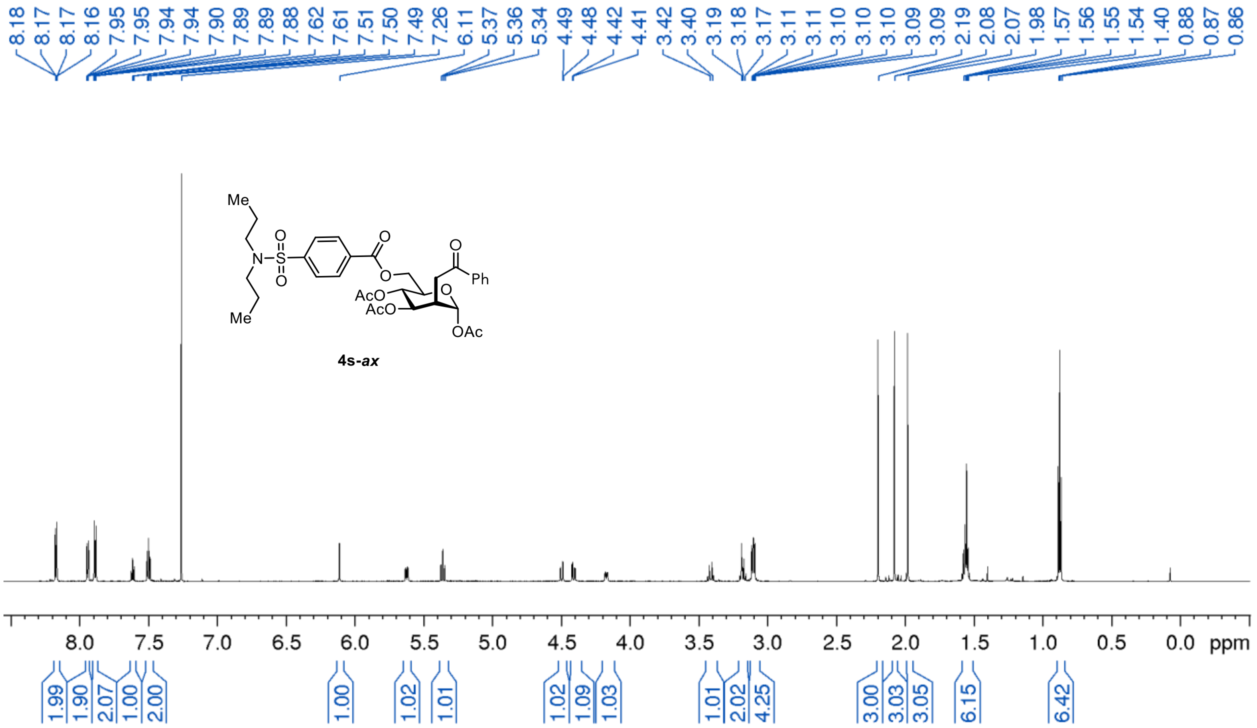

^{13}C NMR (175 MHz, $CDCl_3$, 25 °C) of (4s-*ax*)

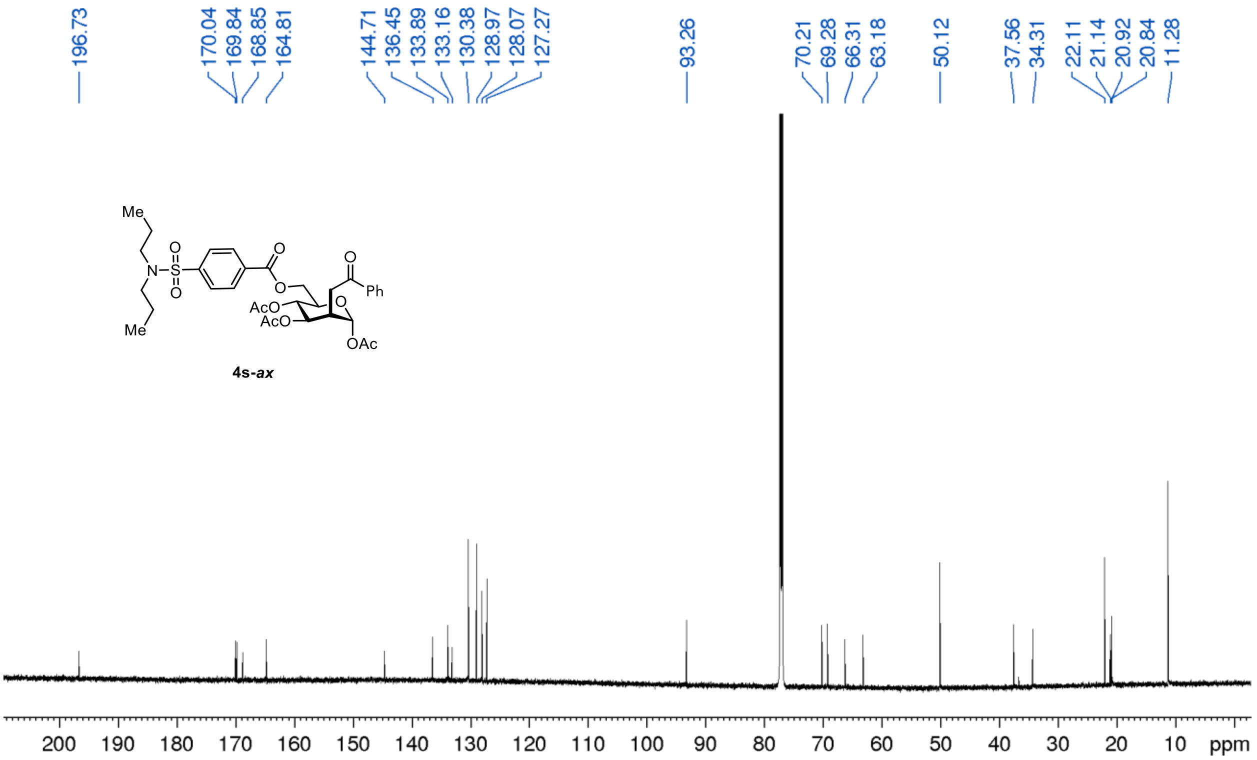

^{1}H NMR (700 MHz, $CDCl_3$, 25 ºC) of (4t-*ax)*

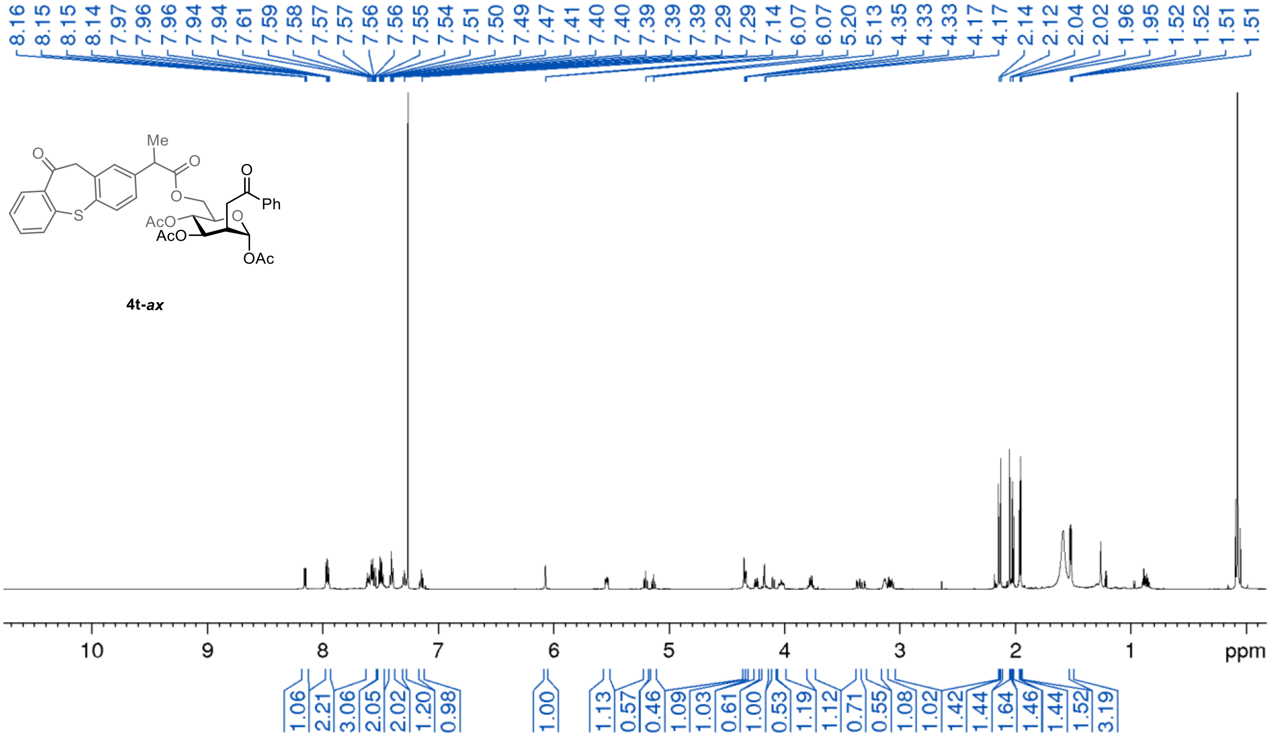

^{13}C NMR (175 MHz, $CDCl_3$, 25 ºC) of (4t-*ax)*

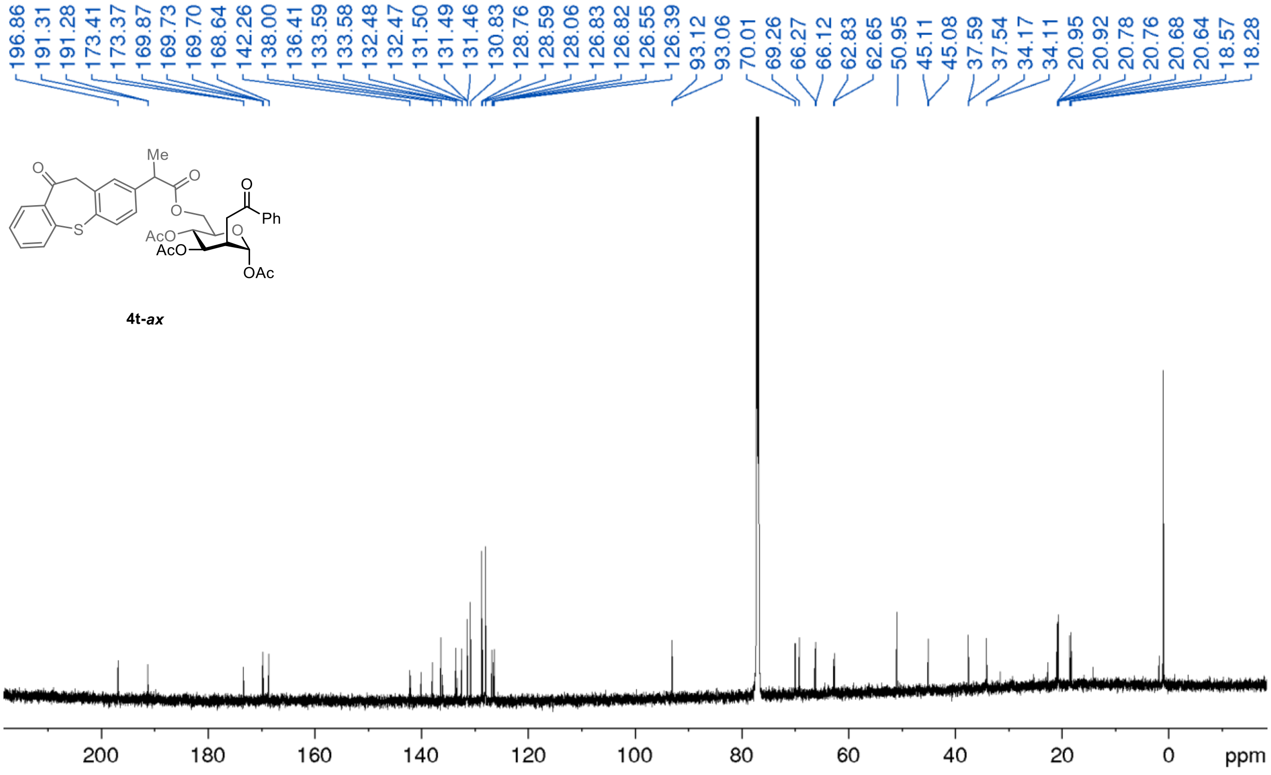

^{1}H NMR (500 MHz, CDCl$_3$, 25 °C) of (4u-*ax*)

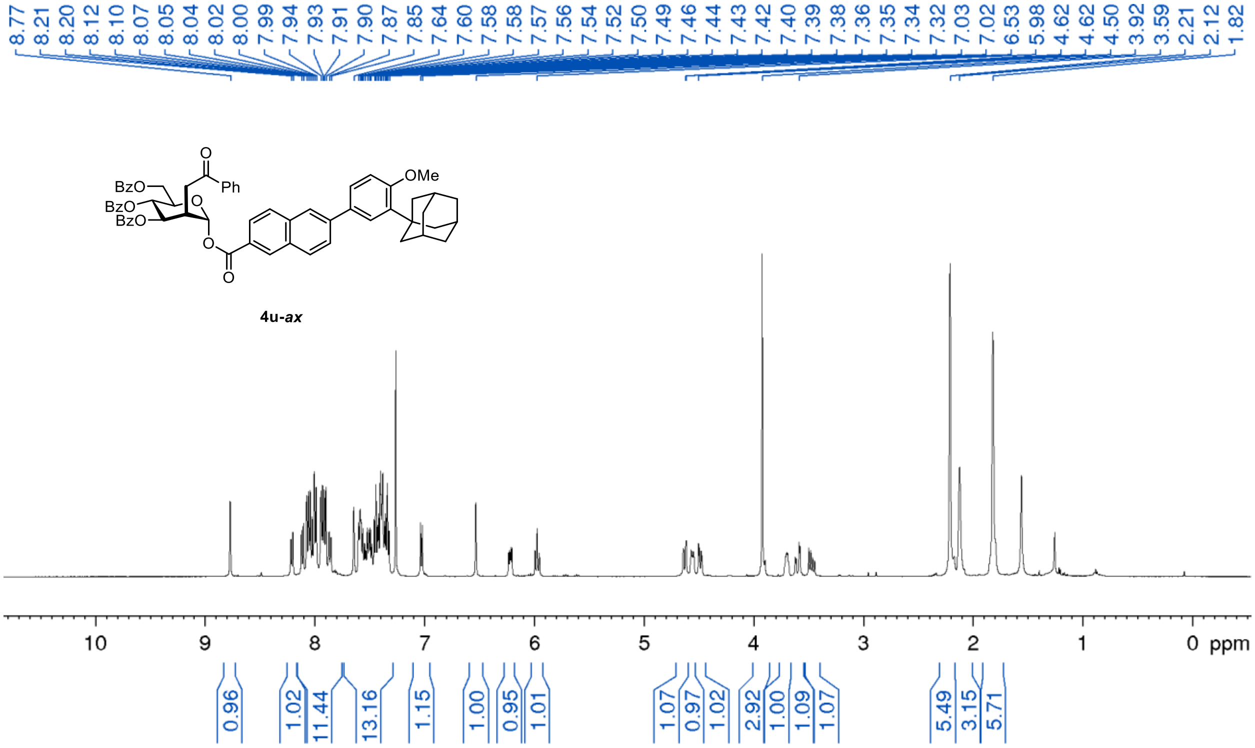

^{13}C NMR (125 MHz, CDCl$_3$, 25 °C) of (4u-*ax*)

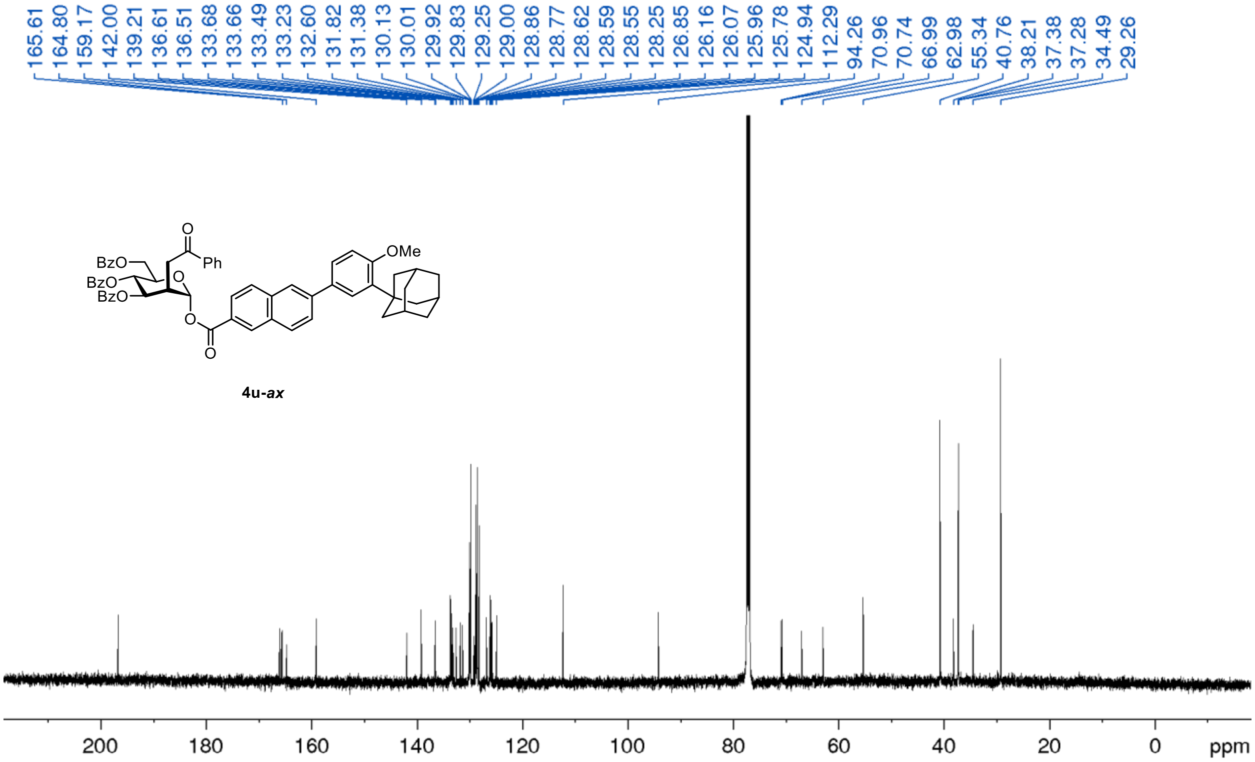

^{1}H NMR (700 MHz, $CDCl_3$, 25 ºC) of (4u-*eq*)

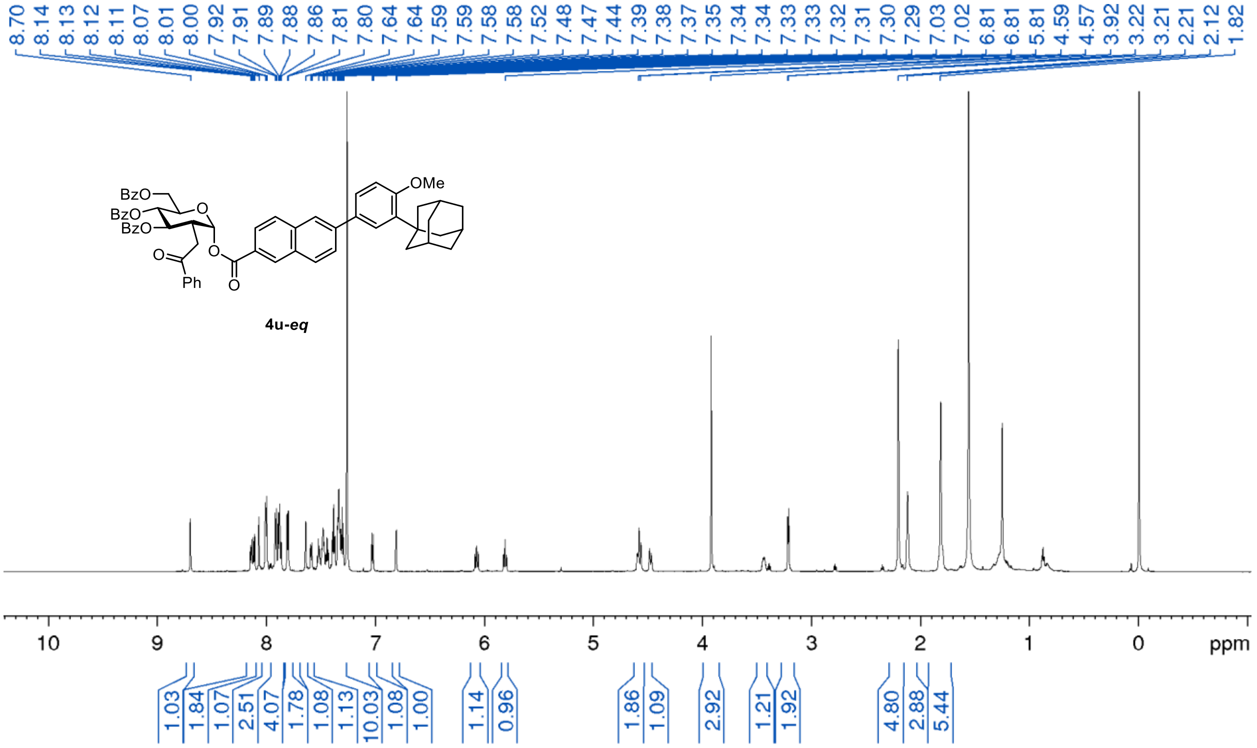

^{13}C NMR (175 MHz, $CDCl_3$, 25 ºC) of (4u-*eq*)

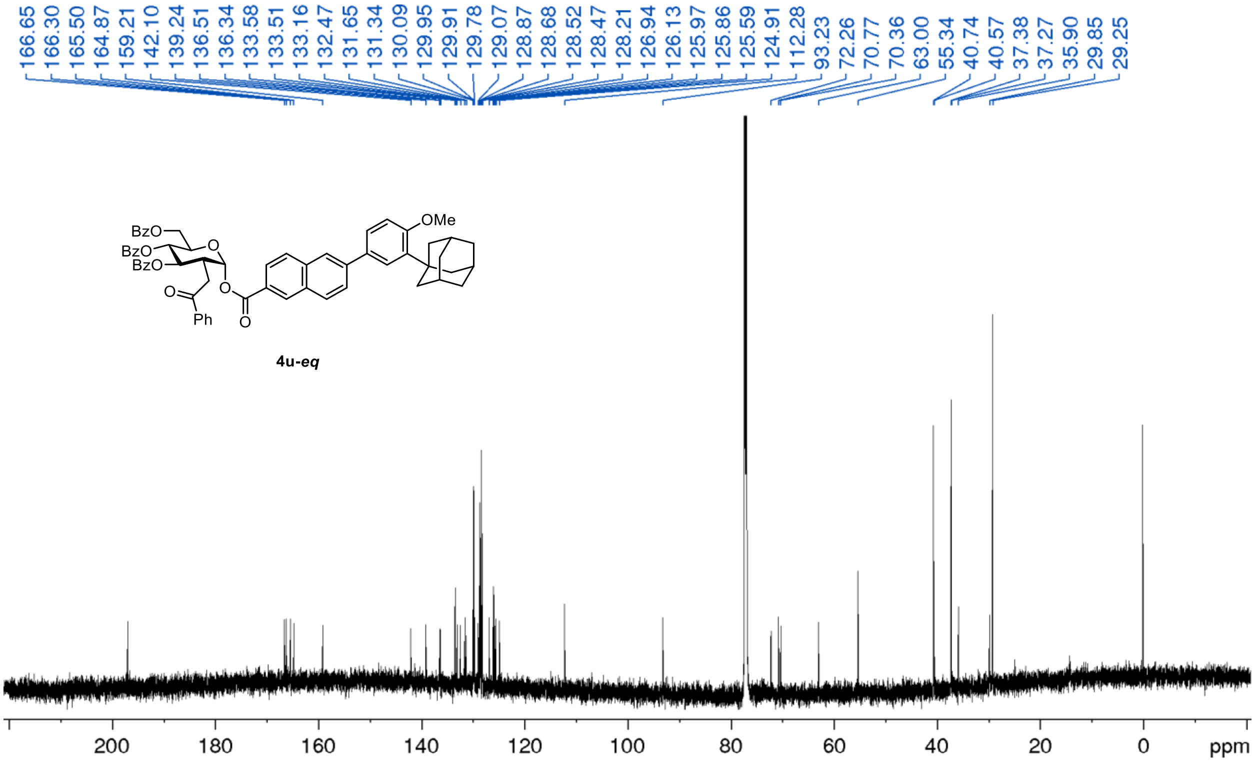

^{1}H NMR (700 MHz, $CDCl_3$, 25 °C) of (5a)

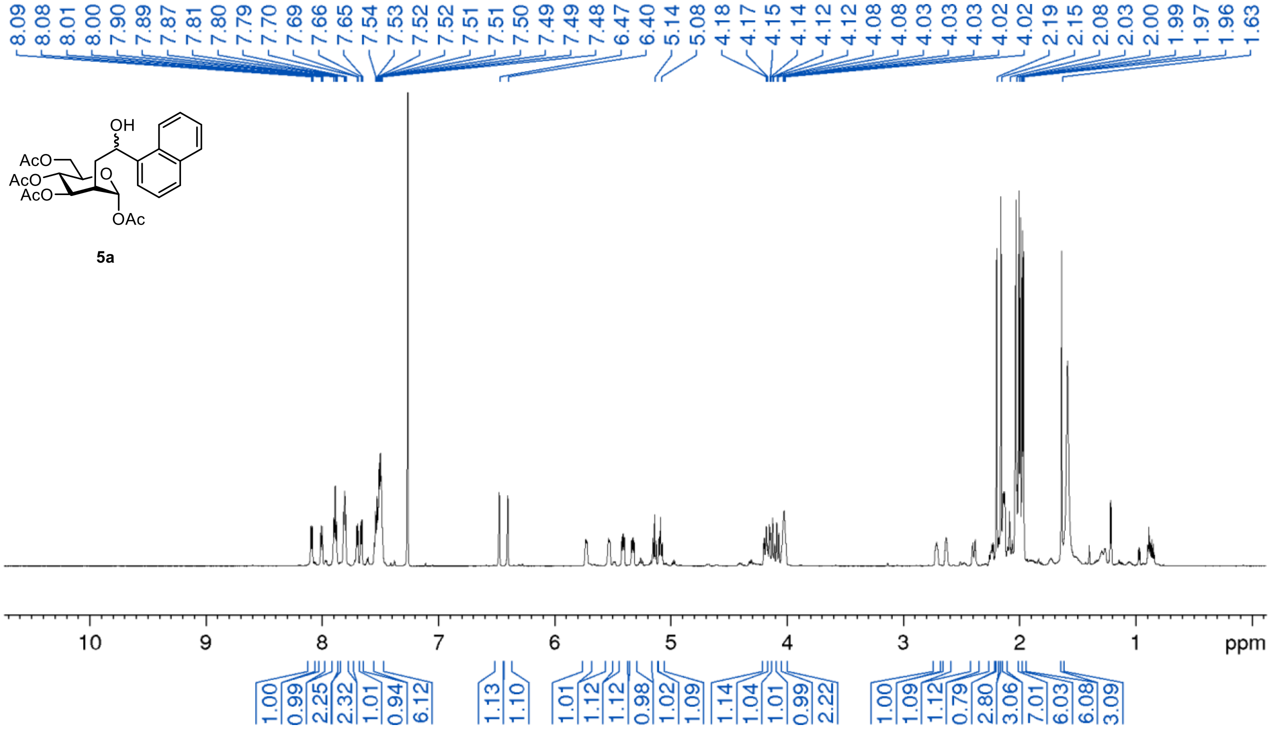

^{13}C NMR (175 MHz, $CDCl_3$, 25 °C) of (5a)

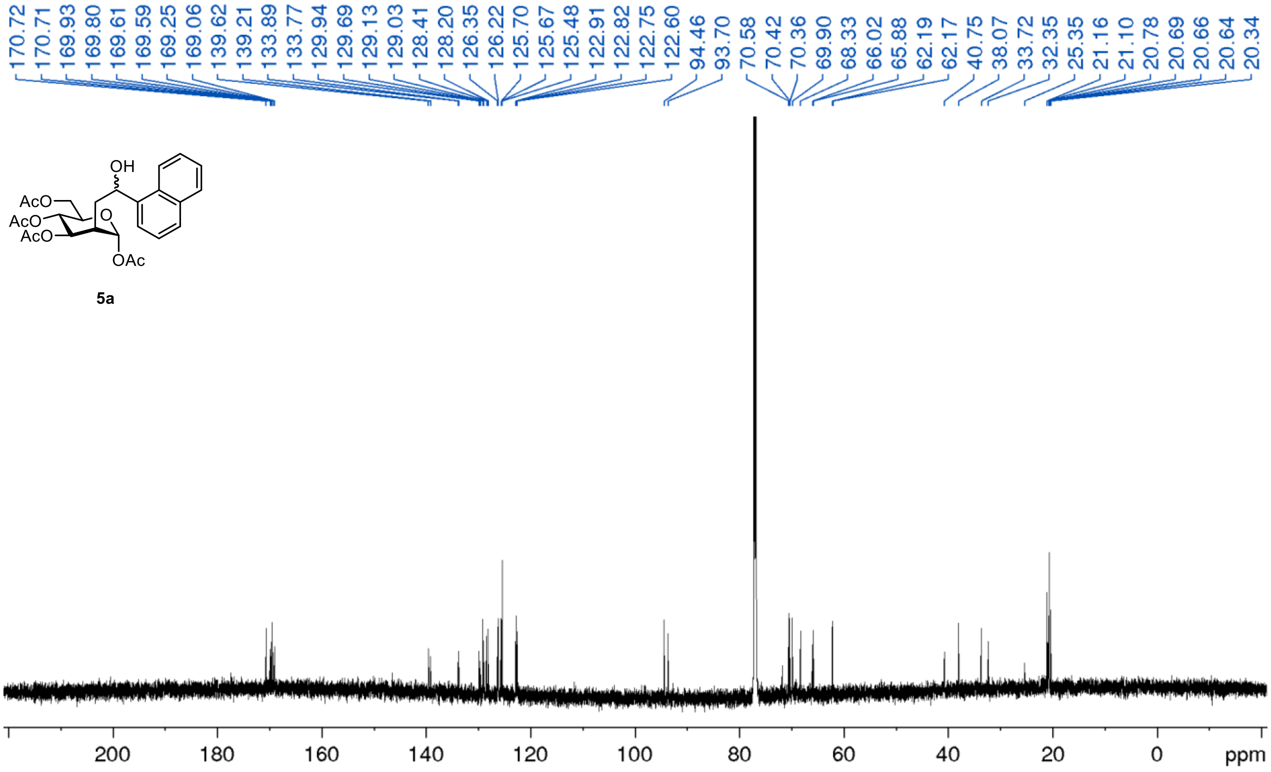

^{1}H NMR (500 MHz, CDCl$_3$, 25 °C) of (6a)

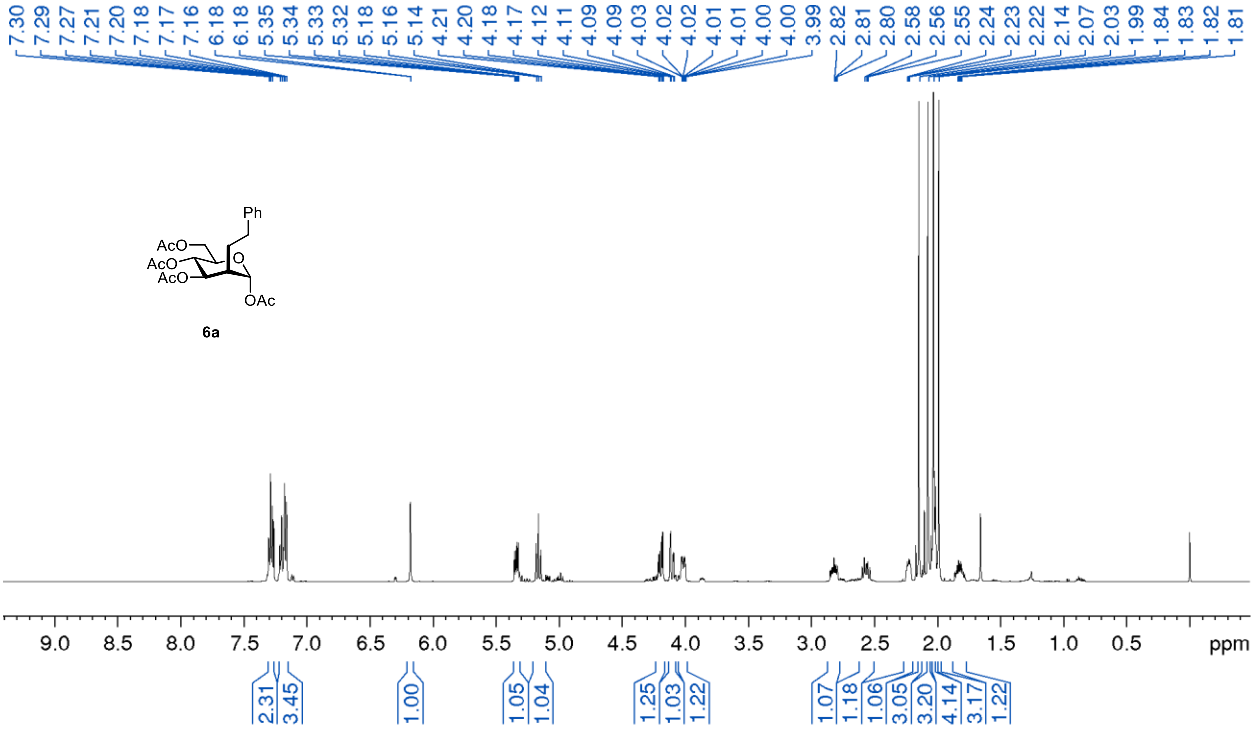

^{13}C NMR (125 MHz, CDCl$_3$, 25 °C) of (6a)

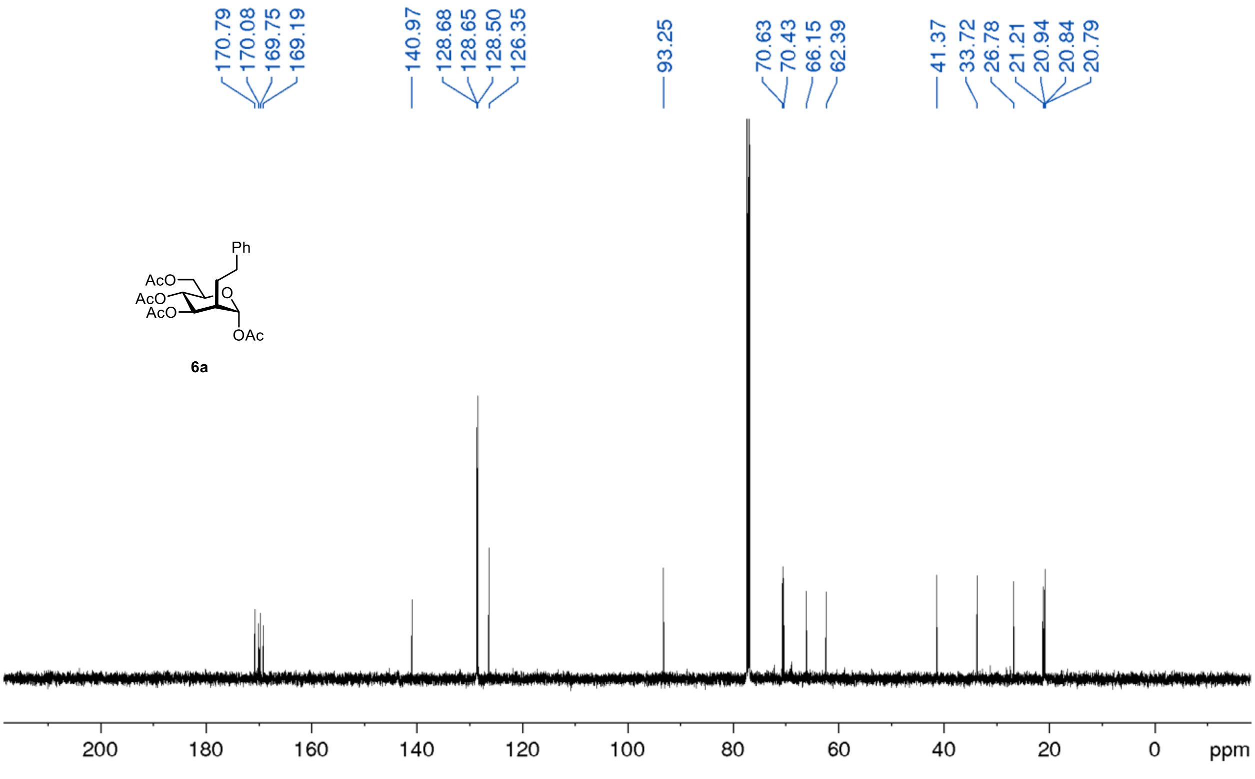

^{1}H NMR (500 MHz, $CDCl_3$, 25 °C) of (7a)

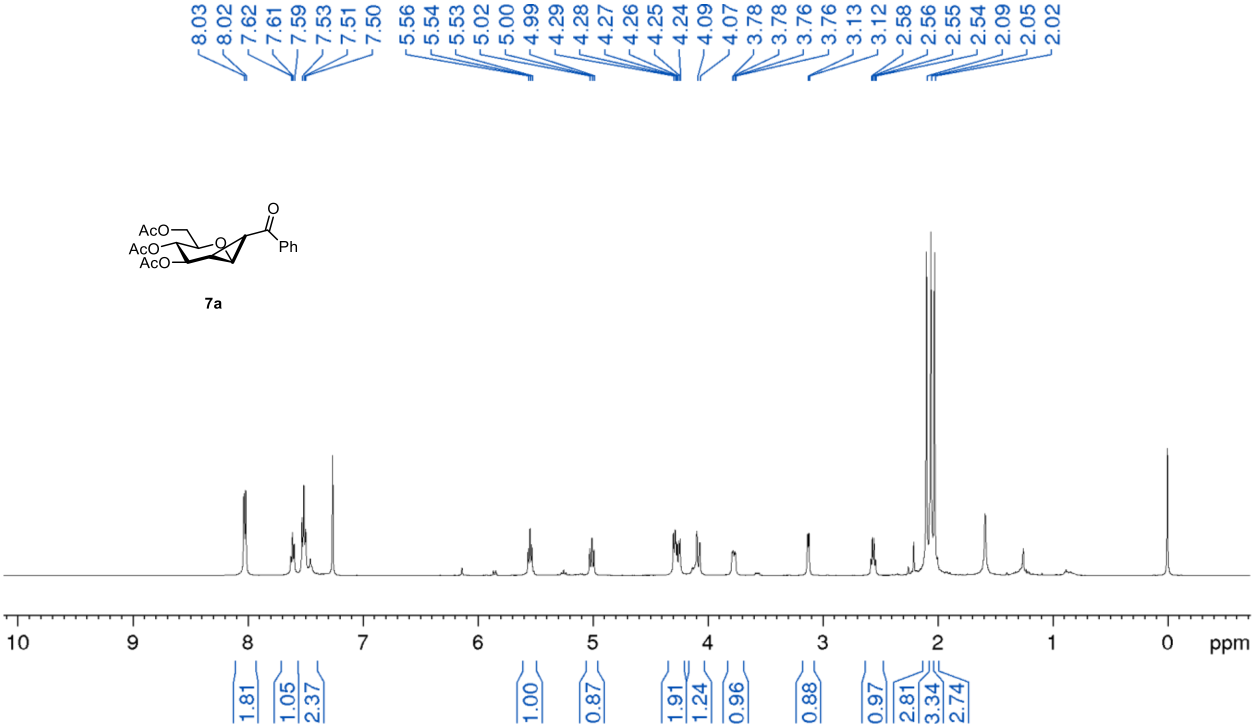

^{13}C NMR (125 MHz, $CDCl_3$, 25 °C) of (7a)

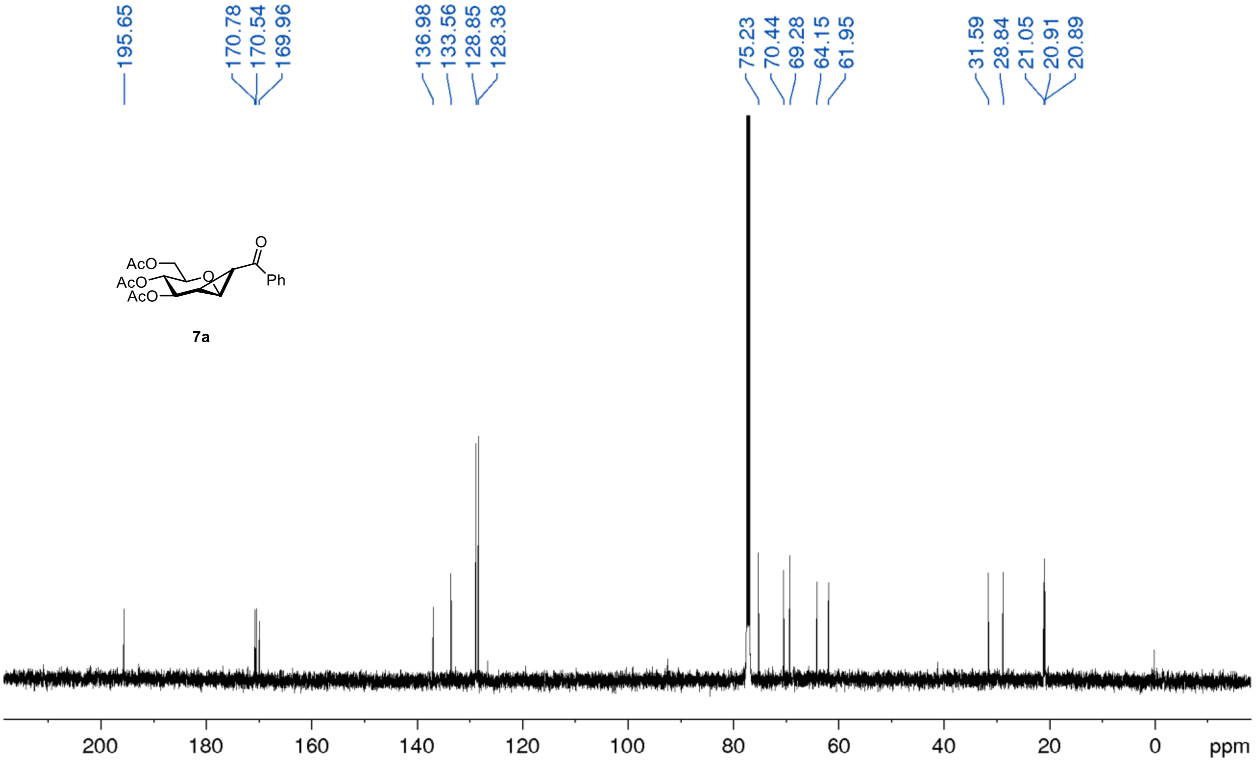

^{1}H NMR (700 MHz, $CDCl_3$, 25 ºC) of (8a)

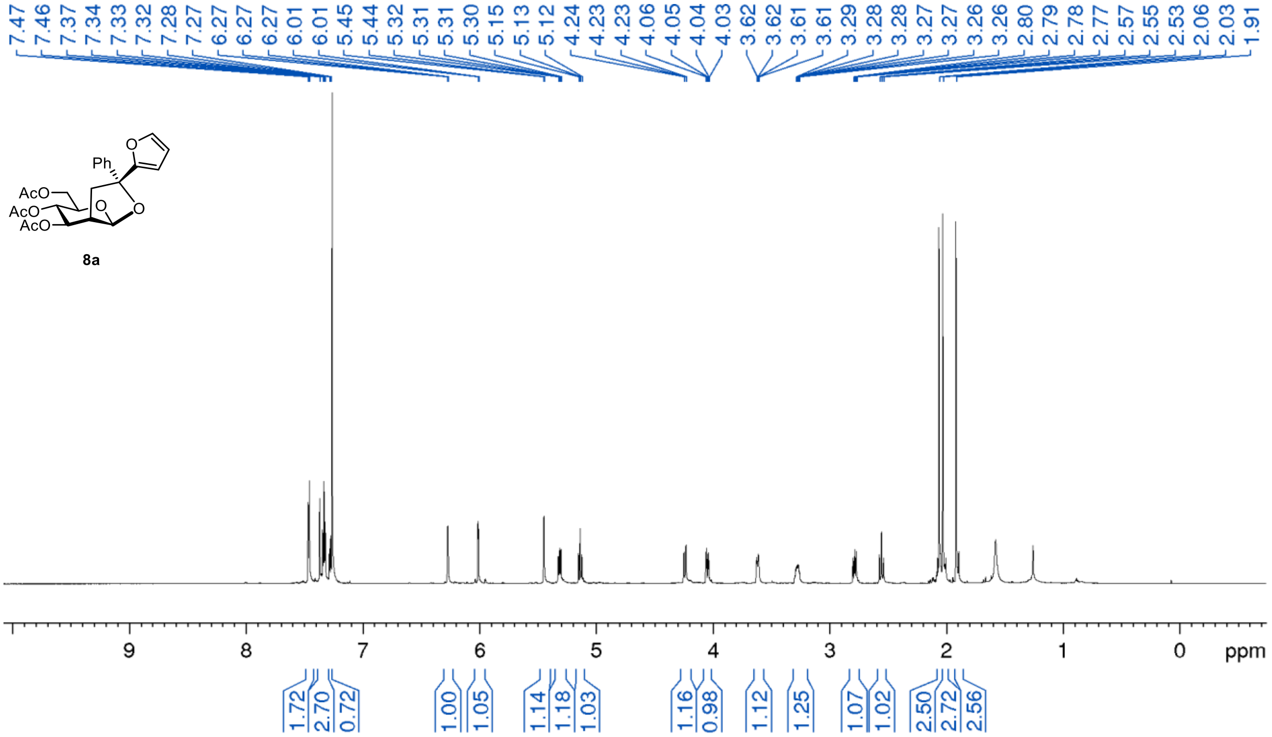

^{13}C NMR (175 MHz, $CDCl_3$, 25 ºC) of (8a)

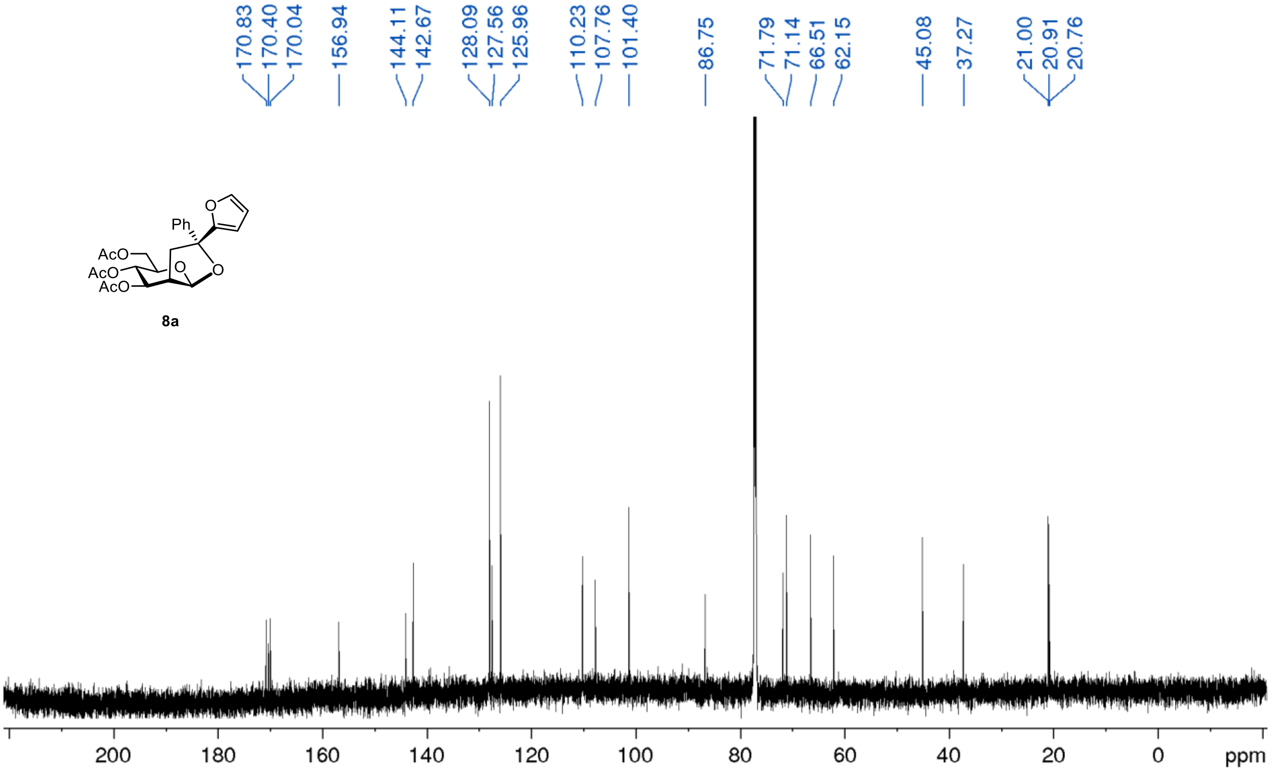

HSQC (700 MHz, $CDCl_3$, 25 °C) of (8a)

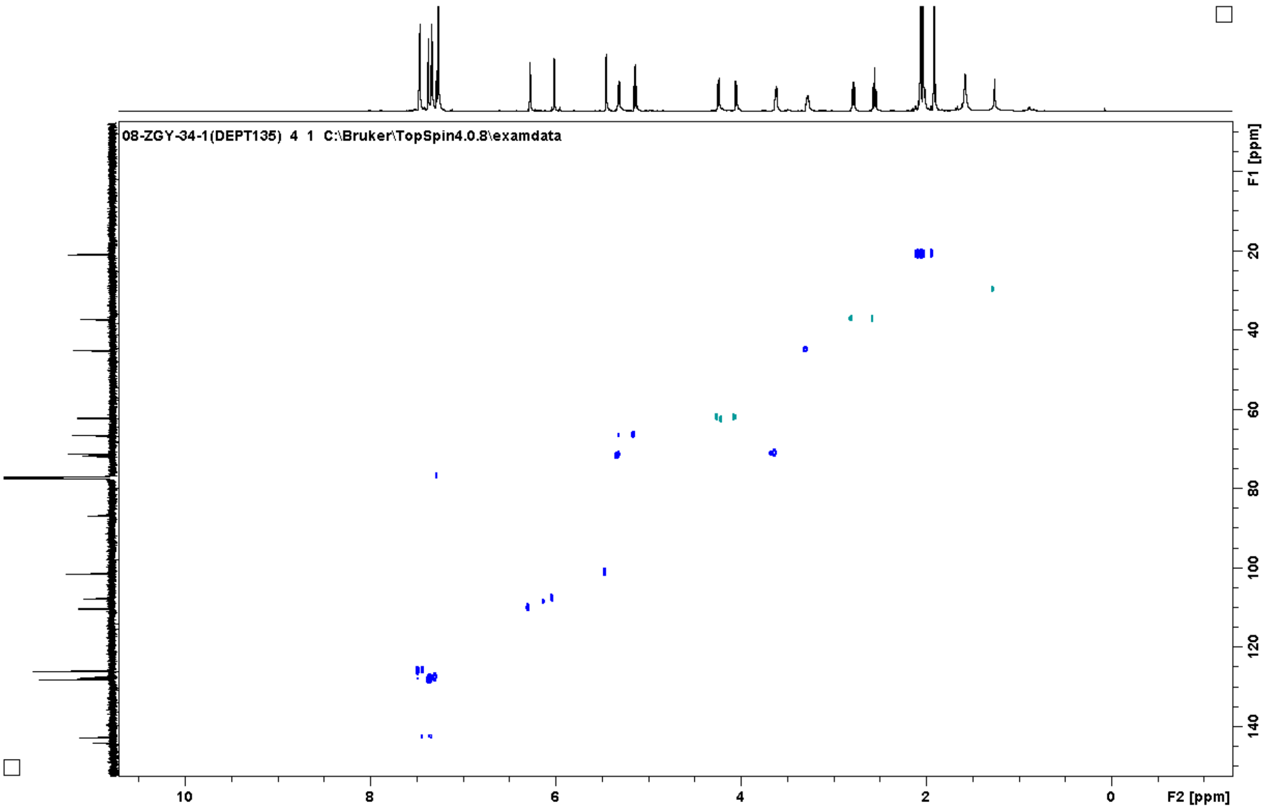

^{1}H NMR (700 MHz, $CDCl_3$, 25 °C) of (*epi*-8a)

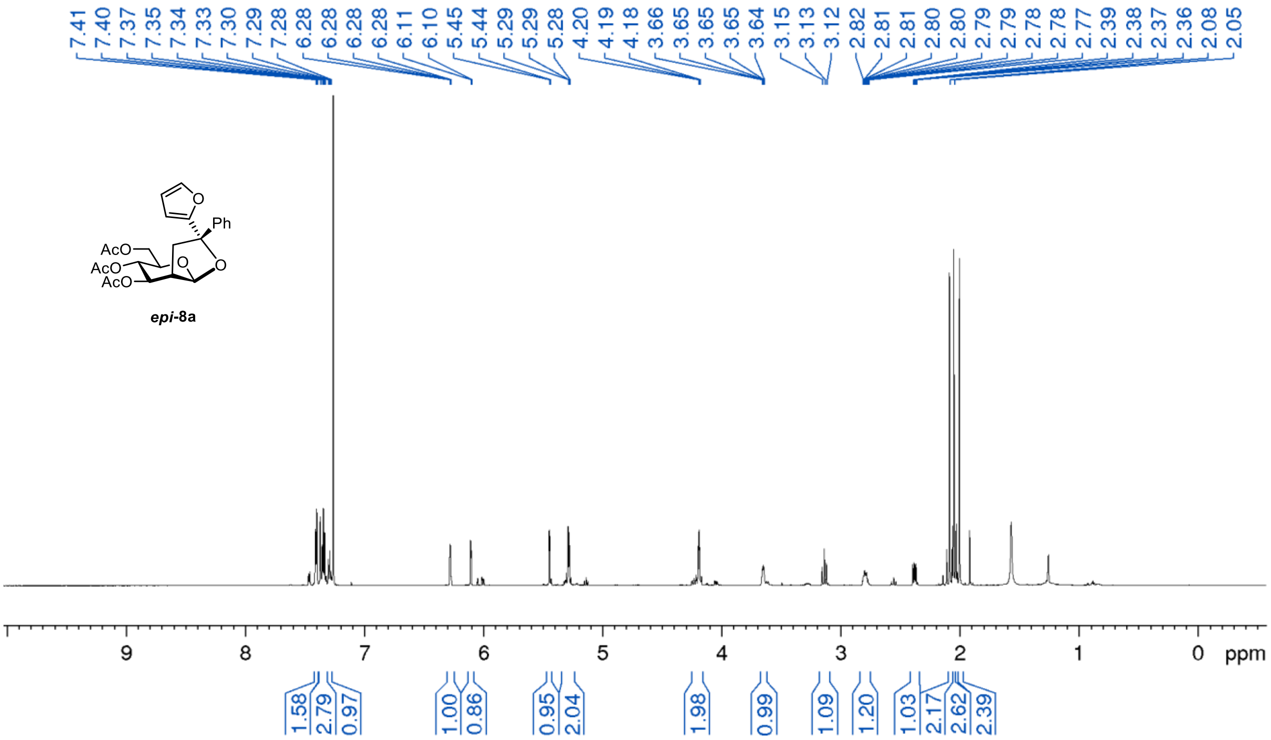

^{13}C NMR (175 MHz, $CDCl_3$, 25 ºC) of (*epi*-8a)

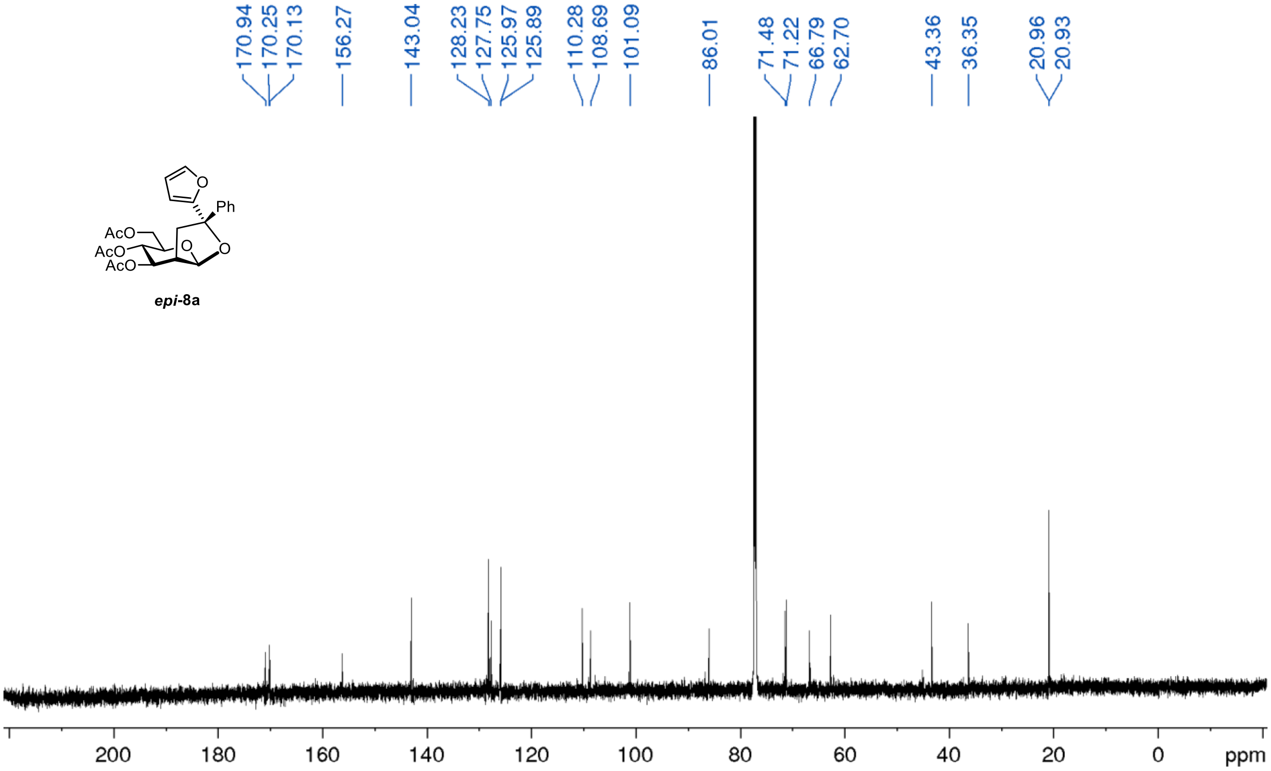

^{1}H NMR (500 MHz, $CDCl_3$, 25 ºC) of (9a)

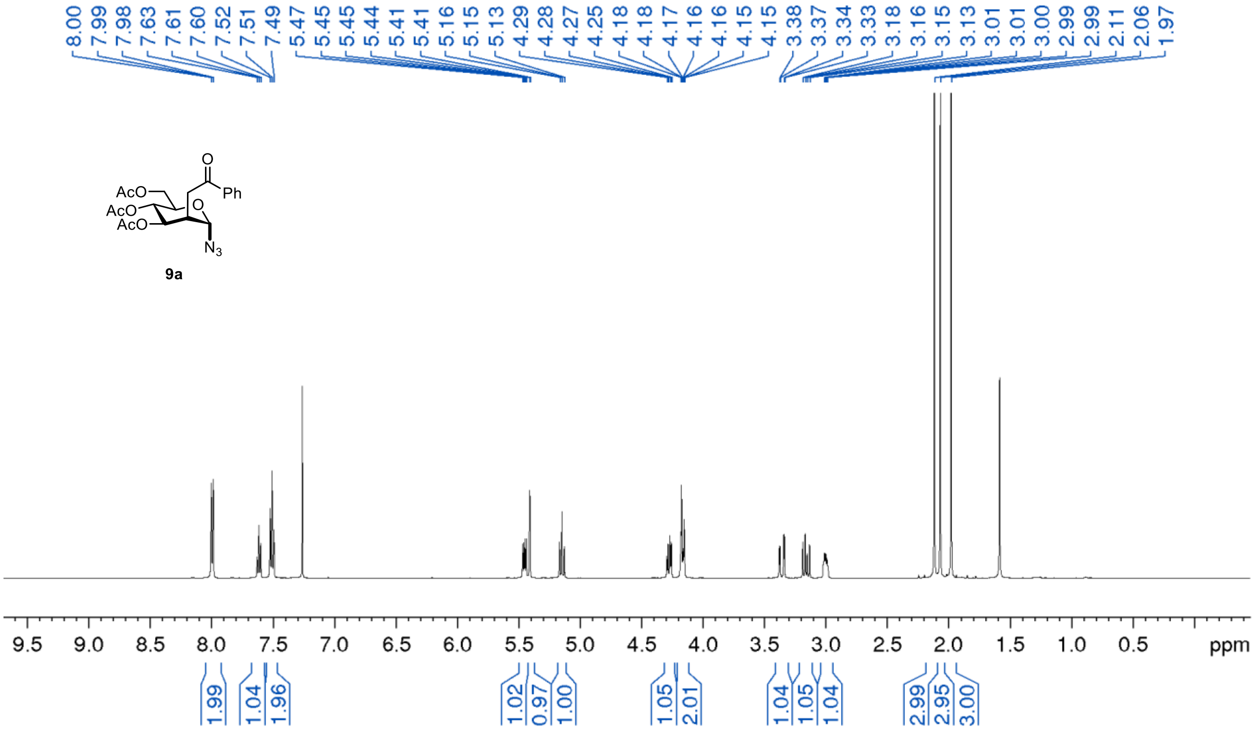

^{13}C NMR (125 MHz, $CDCl_3$, 25 °C) of (9a)

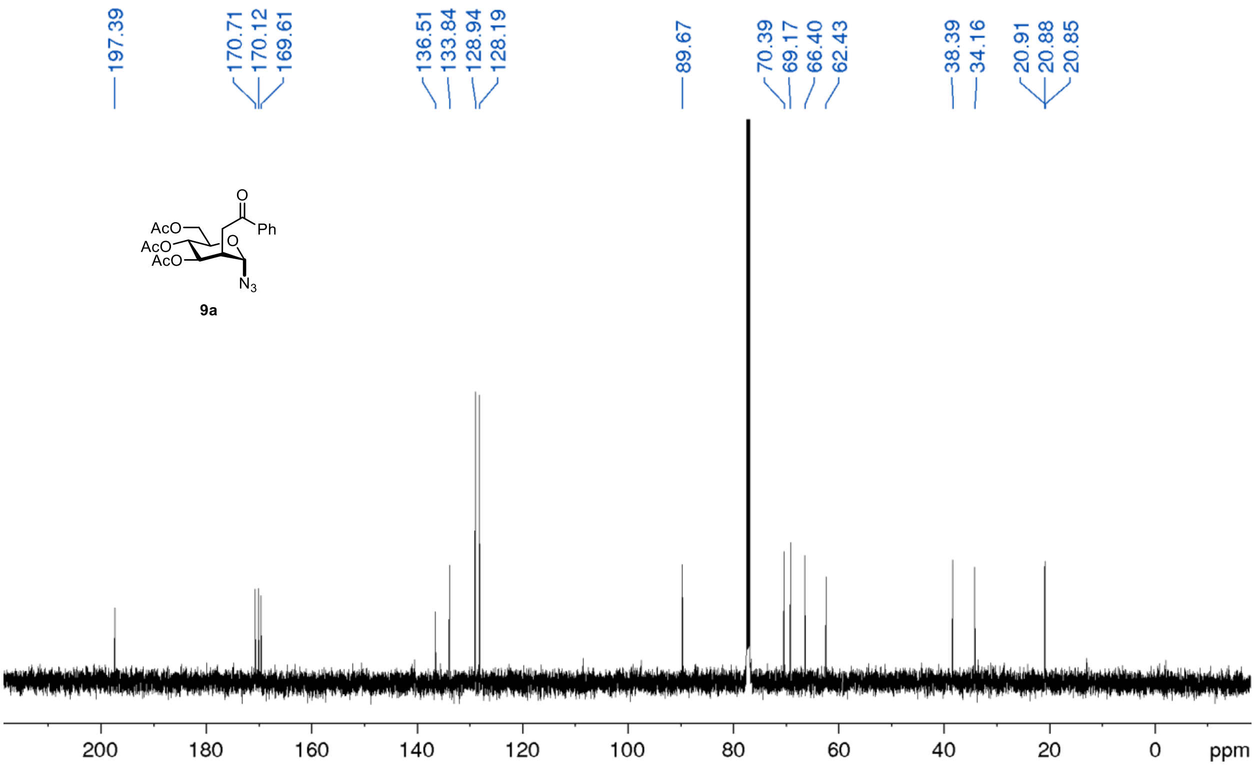

^{1}H NMR (500 MHz, $CDCl_3$, 25 °C) of (10a)

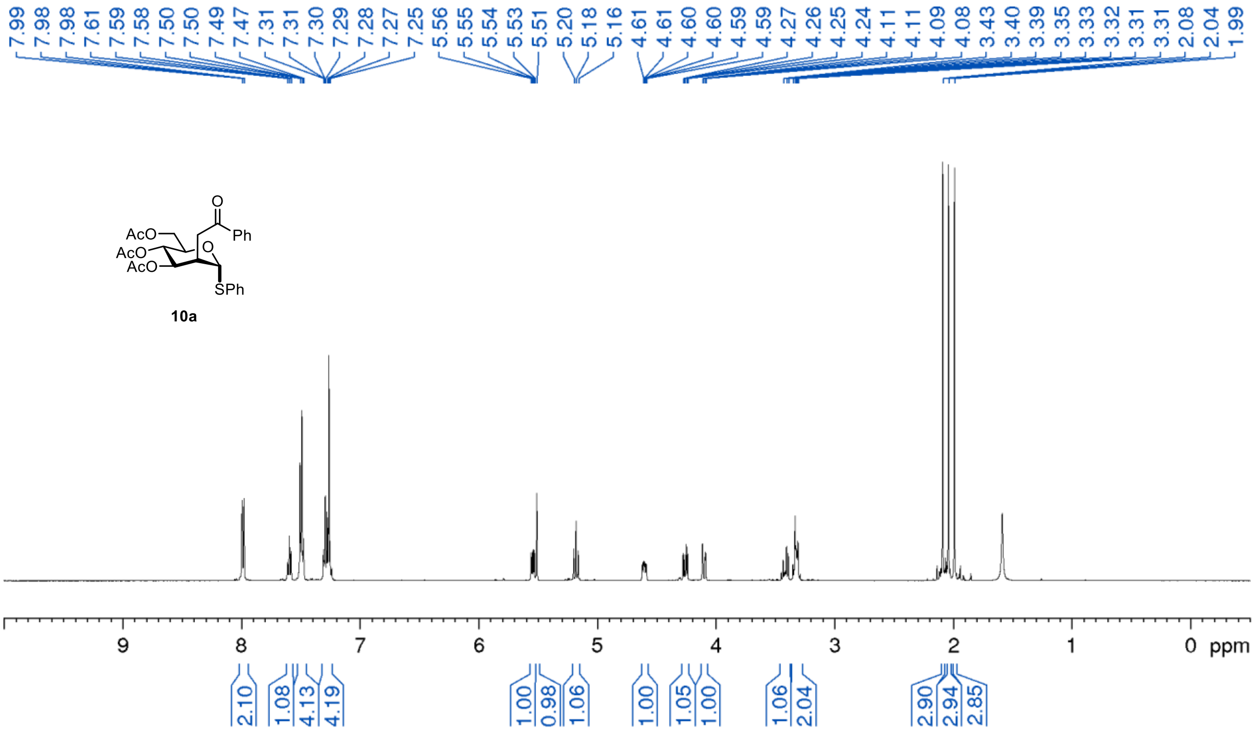

^{13}C NMR (125 MHz, $CDCl_3$, 25 ºC) of (10a)

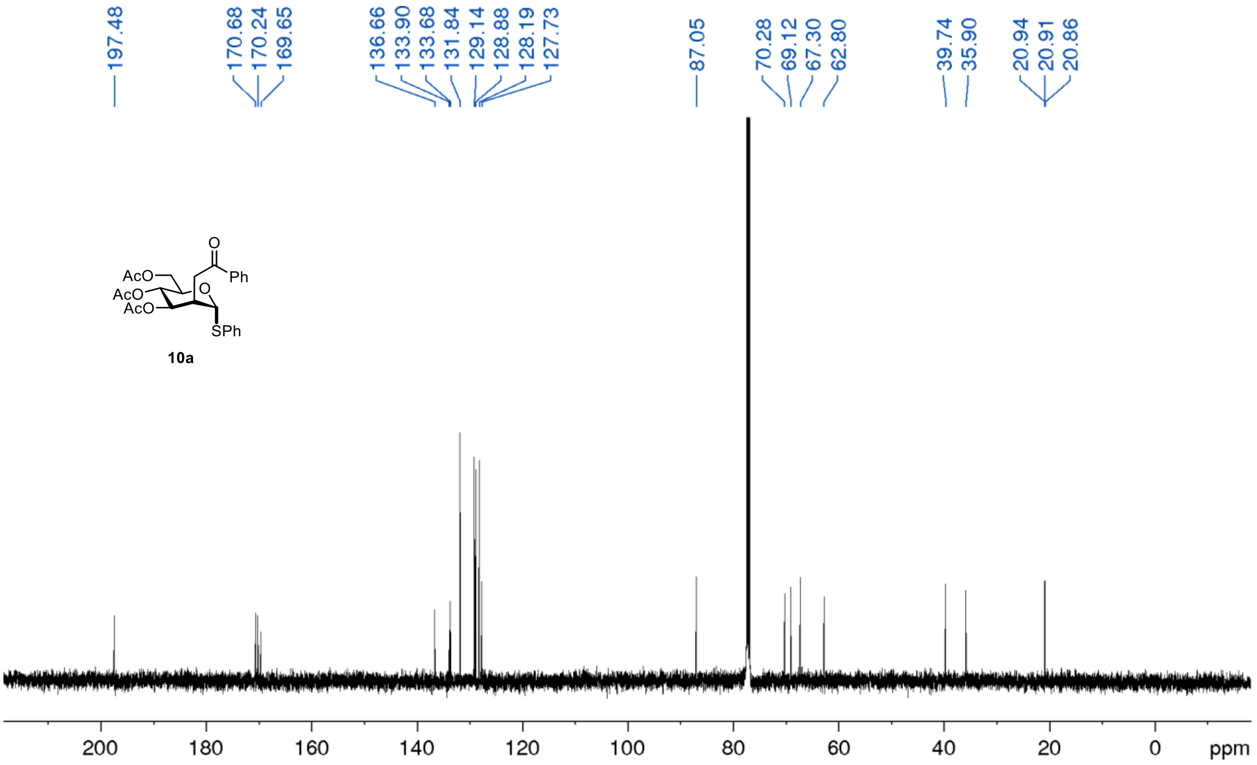

^{1}H NMR (500 MHz, $CDCl_3$, 25 ºC) of (11a)

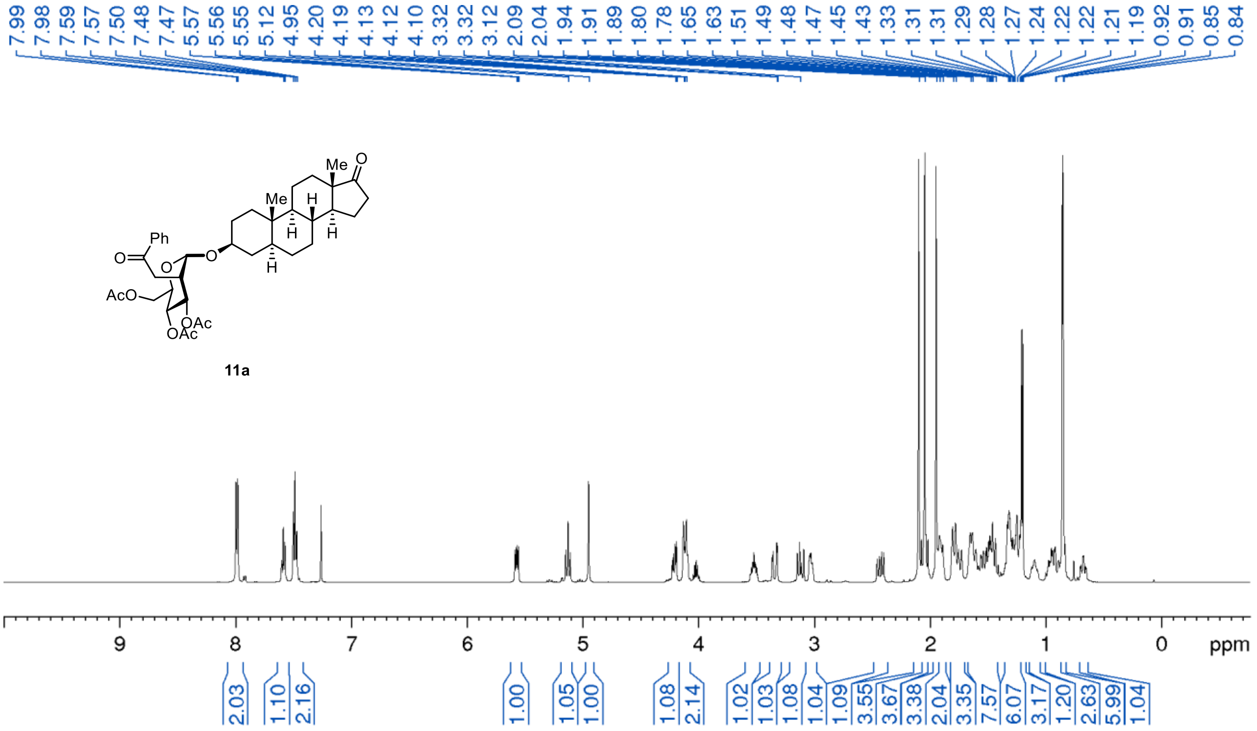

^{13}C NMR (100 MHz, CDCl$_3$, 25 °C) of (11a)

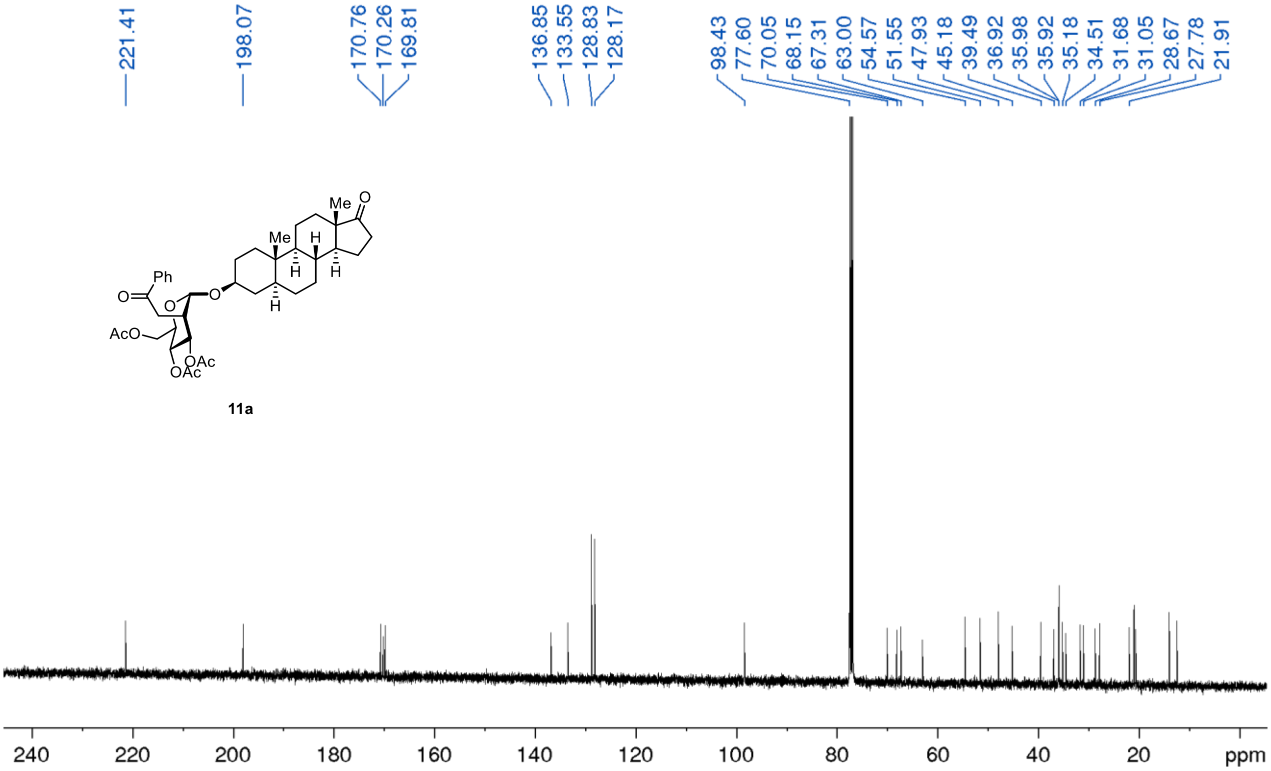

11a

^{1}H NMR (700 MHz, CDCl$_3$, 25 °C) of (12a)

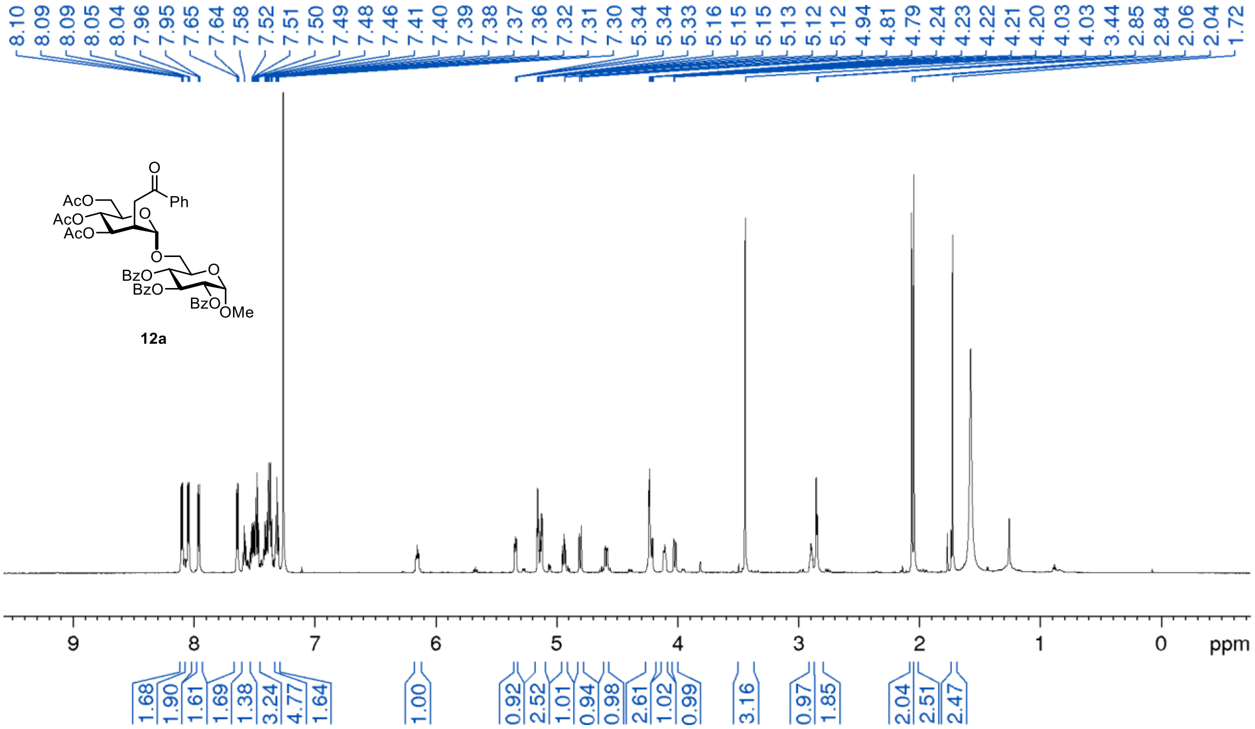

12a

^{13}C NMR (175 MHz, CDCl$_3$, 25 ºC) of (12a)

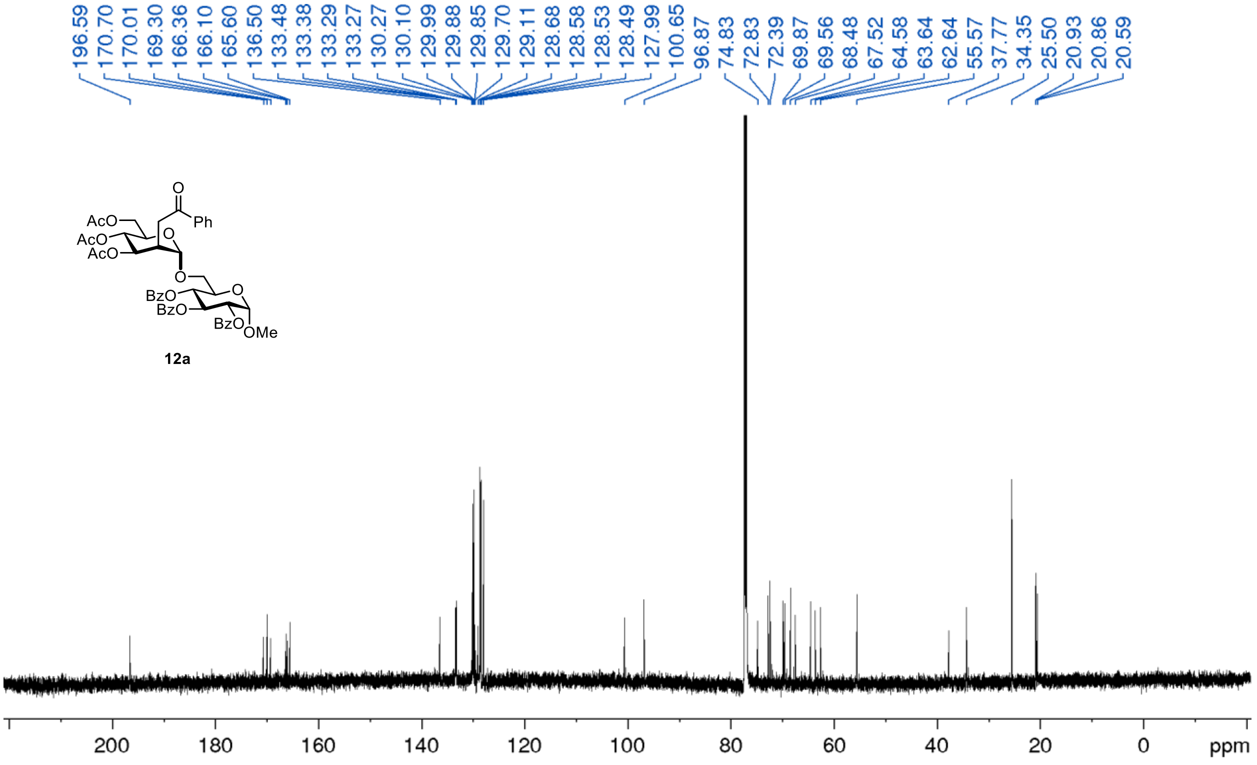

^{1}H NMR (700 MHz, CDCl$_3$, 25 ºC) of (3s)

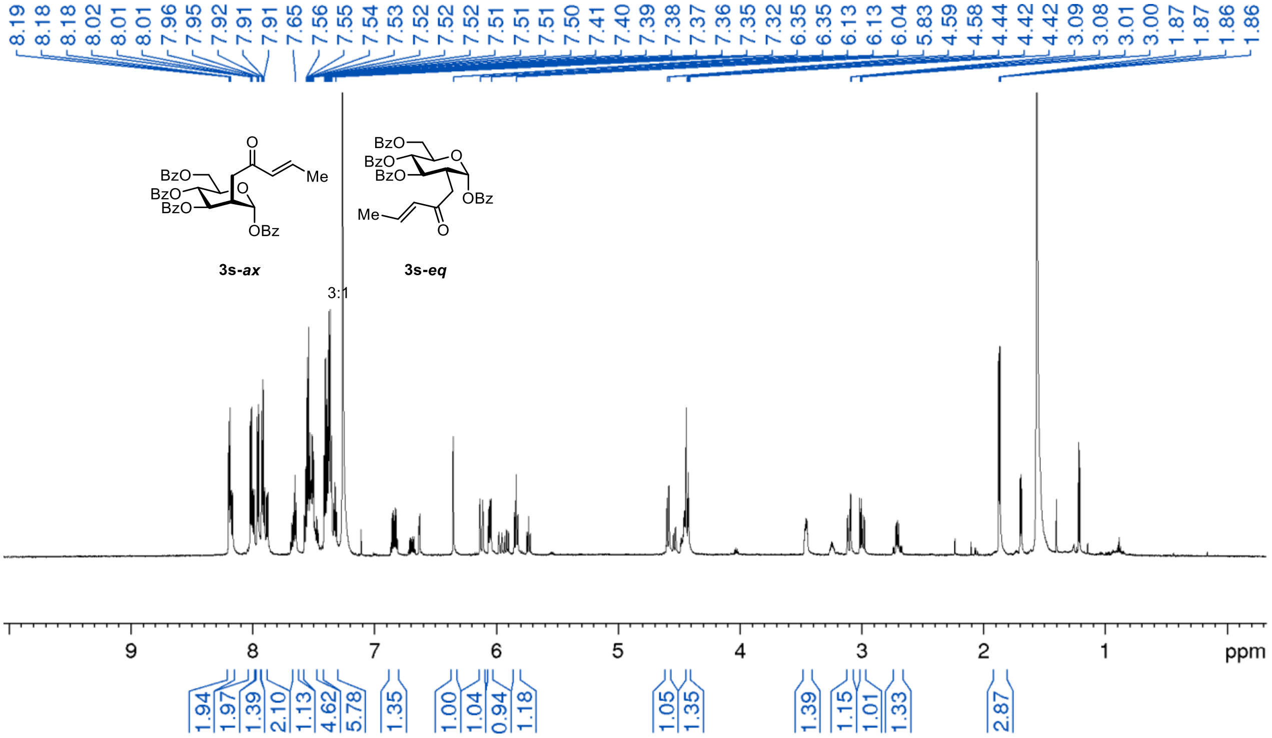

^{13}C NMR (175 MHz, $CDCl_3$, 25 °C) of (3s)

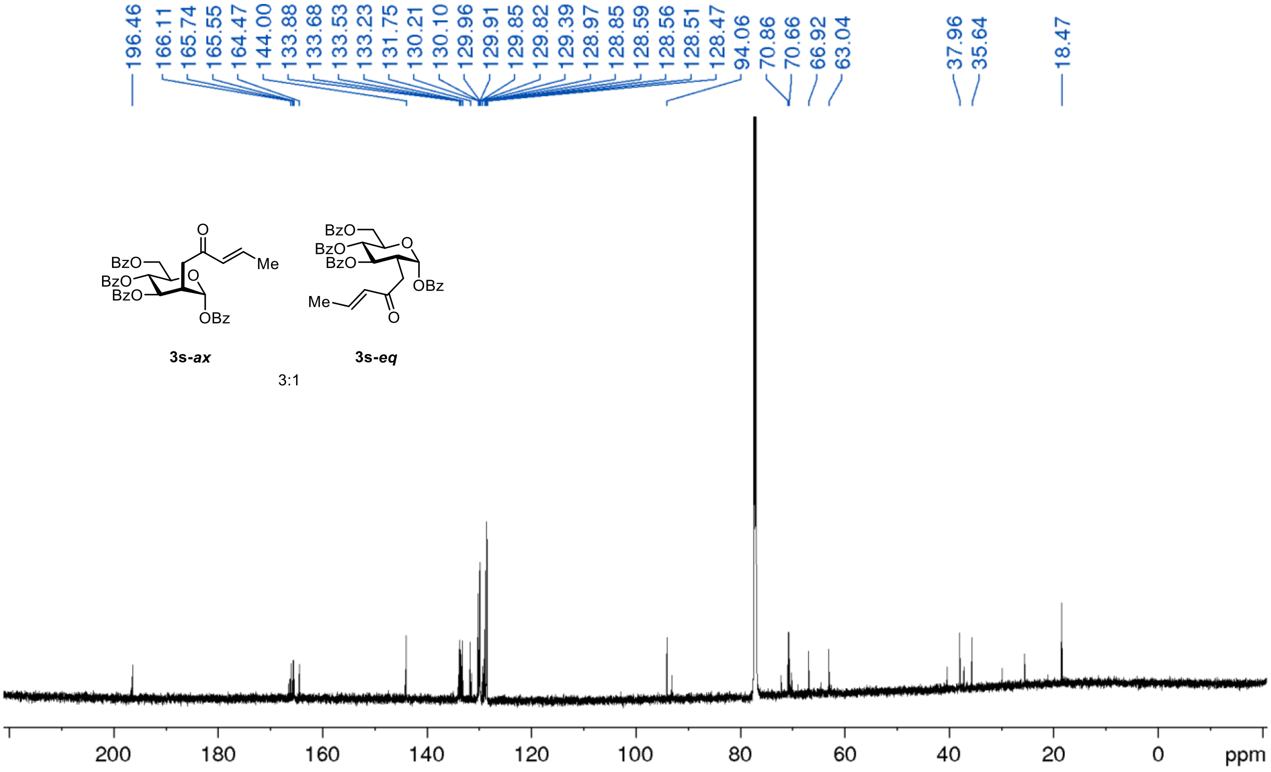

HSQC (700 MHz, $CDCl_3$, 25 °C) of (3a-*ax*)

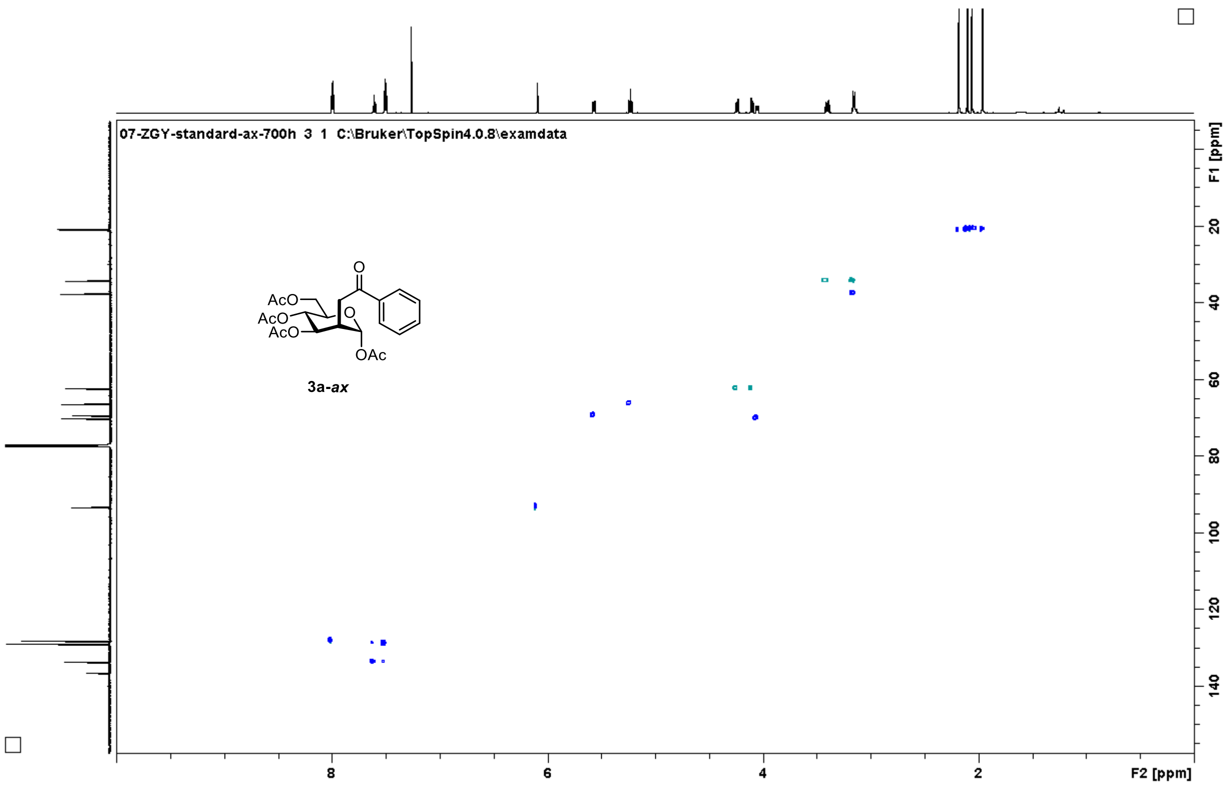

HSQC (700 MHz, CDCl$_3$, 25 °C) of (3a-*eq*)

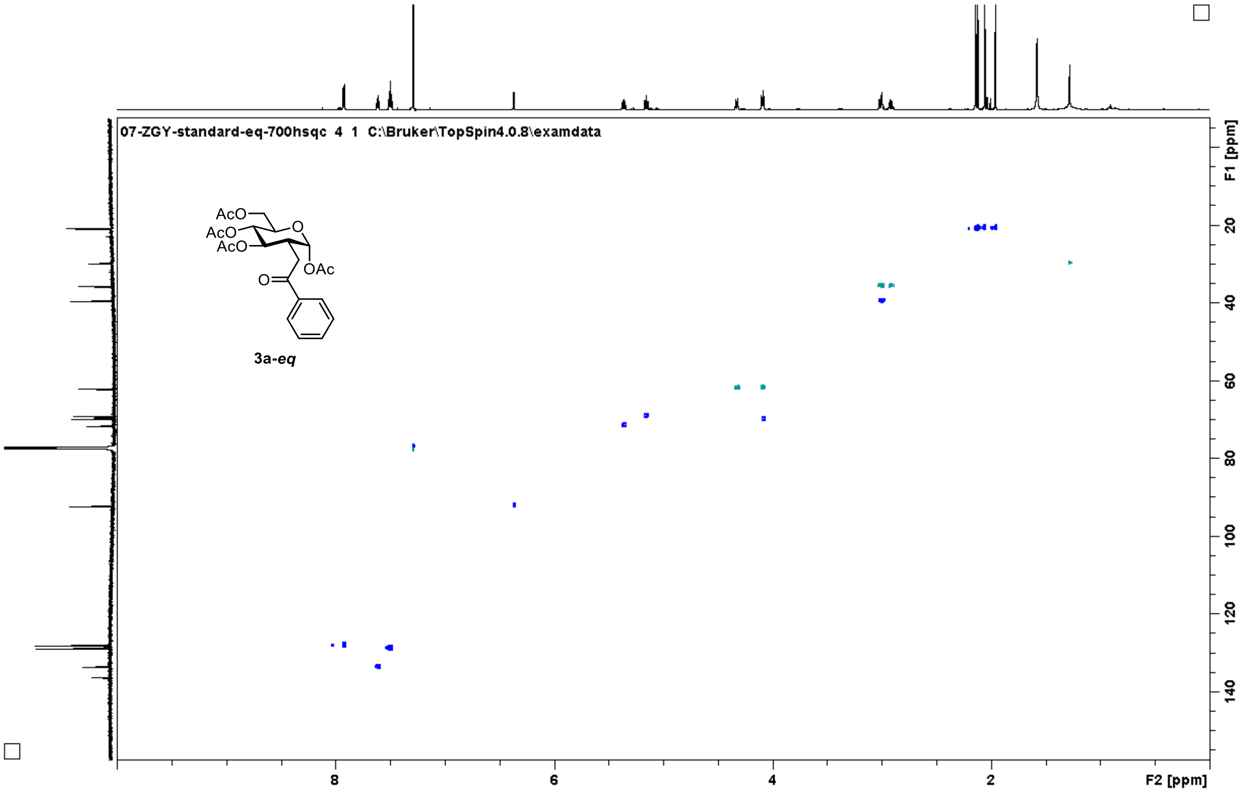

Cartesian Coordinates (Å) and Energies of the Optimized Structures

II

B3LYP-D3 SCF energy:	-881.886481384 a.u.
B3LYP-D3 enthalpy:	-881.563622384 a.u.
B3LYP-D3 free energy:	-881.634300384 a.u.
M06 SCF energy in solution:	-881.25962 a.u.
M06 enthalpy in solution (25°C):	-881.319433 a.u.
M06 free energy in solution (25°C):	-881.261347 a.u.
M06 enthalpy in solution (90°C):	-881.333008 a.u.
M06 free energy in solution (90°C):	-881.886481384 a.u.

O	0.60431800	-1.82414300	-0.51128700
O	3.20600200	-1.11390100	0.58532400
O	1.77172300	1.65459100	-0.54913100
O	-0.85962800	1.93034000	0.60957400
O	-2.64829100	-0.14426800	-0.33138600
O	-3.45760400	-1.64679400	1.16071400
C	-0.74845700	-1.64274200	-0.44100300
H	-1.27299800	-2.57248500	-0.24849400
C	-1.29845300	-0.37536400	0.15063100
H	-1.34325200	-0.43224300	1.24793500
C	-0.48242700	0.85460000	-0.23568400
H	-0.68860000	1.09592200	-1.28860800
C	1.00665700	0.54812700	-0.08518700
H	1.21854100	0.33765800	0.96912200
C	1.38384300	-0.68772000	-0.91370600
H	1.17848600	-0.47467900	-1.97416400
C	-3.63993400	-0.82745800	0.28710700
C	-4.98456300	-0.41817100	-0.26693000
H	-5.15421800	0.64687900	-0.07695700
H	-5.76822100	-1.01034600	0.20663400
H	-5.00408000	-0.56483000	-1.35157100
C	2.85025500	-1.05983300	-0.78362200
H	3.01246200	-2.03494100	-1.26993000
H	3.45057300	-0.30318600	-1.31244700
C	4.53838300	-1.53990900	0.78620800
H	4.71642000	-1.54005300	1.86467900
H	4.70247100	-2.55698000	0.39584800
H	5.26137600	-0.86212400	0.30291400
C	2.59337300	2.24526800	0.45046000
H	3.15153400	3.04921600	-0.03761600
H	1.98692200	2.66832600	1.26323600
H	3.29579300	1.51231900	0.86939100
C	-0.98459300	3.17881600	-0.05399400
H	-1.78828600	3.15239400	-0.80609500
H	-1.24362200	3.91473300	0.71257700
H	-0.04797100	3.47414700	-0.54258300

2

B3LYP-D3 SCF energy:	-793.60546927 a.u.
B3LYP-D3 enthalpy:	-793.34862327 a.u.
B3LYP-D3 free energy:	-793.40933027 a.u.
M06 SCF energy in solution:	-793.079032739 a.u.
M06 enthalpy in solution (25°C):	-793.131173739 a.u.
M06 free energy in solution (25°C):	-793.080160739 a.u.
M06 enthalpy in solution (90°C):	-793.143053739 a.u.
M06 free energy in solution (90°C):	-793.60546927 a.u.

C	-0.17844900	-0.83141200	-0.04692800
C	0.14444300	-2.10154300	0.25215400
H	-0.61329100	-2.81117300	0.55840200
H	1.16503400	-2.46143600	0.20601200
C	-1.56739700	-0.30421200	-0.04606700
C	-2.67724500	-1.15347200	-0.19223300
C	-1.79525800	1.07310200	0.10281100
C	-3.97328100	-0.64439800	-0.16167900
H	-2.52465400	-2.21581800	-0.35582700
C	-3.09314300	1.58182000	0.13222900
H	-0.94515600	1.73957700	0.19603400
C	-4.18829200	0.72619000	0.00431600
H	-4.81776100	-1.31814900	-0.28102800
H	-3.24836400	2.65073900	0.25420800
H	-5.19978700	1.12310600	0.02323900
O	0.74098900	0.12518700	-0.37857400
Si	2.39130400	0.23896000	-0.02068600
C	3.38134500	-0.94878500	-1.09688300
H	3.12281000	-0.81642100	-2.15395500
H	4.45662800	-0.75758300	-0.98992100
H	3.20472700	-1.99890100	-0.84039400
C	2.81204900	2.01480700	-0.46103800
H	2.22961600	2.71895400	0.14400600
H	3.87527700	2.22333900	-0.29078500
H	2.59356600	2.21846800	-1.51545500
C	2.65986800	-0.10139100	1.81026200
H	2.27940000	-1.08776200	2.09482600
H	3.72652200	-0.06235700	2.06302000
H	2.14143200	0.64424500	2.42415100

III

B3LYP-D3 SCF energy:	-881.888056 a.u.
B3LYP-D3 enthalpy:	-881.566074 a.u.
B3LYP-D3 free energy:	-881.636778 a.u.
M06 SCF energy in solution:	-881.263663 a.u.
M06 enthalpy in solution (25°C):	-881.323674 a.u.
M06 free energy in solution (25°C):	-881.265341 a.u.
M06 enthalpy in solution (90°C):	-881.337309 a.u.
M06 free energy in solution (90°C):	-881.888056 a.u.

C	0.47573000	-1.69691600	-0.20824900
C	-0.70282900	-1.45910500	-1.08820500
C	-1.50034400	-0.21634000	-0.92086500
O	-0.71596600	0.91392700	-0.66996100
C	0.25254800	0.74394800	0.37353800
C	0.92971800	2.08537500	0.59319800
H	1.52086400	2.03452500	1.52142600
O	1.75998600	2.38833700	-0.51260900
C	2.33332000	3.67685200	-0.42634000
H	2.95634100	3.81159700	-1.31437100
H	1.56219800	4.46338100	-0.40692800
H	2.96220900	3.78561200	0.47303600
H	0.15085500	2.85407200	0.71876200
C	1.23930300	-0.37757500	0.00304300
H	1.72976300	-0.11417400	-0.93984700
O	2.18604800	-0.53549600	1.05398200
C	3.51526200	-0.17582600	0.69790300
H	4.12026200	-0.27578900	1.60355100
H	3.56617400	0.85715000	0.33161300
H	3.91493600	-0.84717400	-0.07622300
H	-0.25699900	0.47162400	1.30984300
H	-2.11340800	0.02519200	-1.78915100
O	-2.40425000	-0.43455500	0.21919300
C	-3.61383400	0.18986000	0.17790500
O	-4.02186500	0.83131600	-0.76234800
C	-4.36522000	-0.04849000	1.46722300
H	-5.35686900	0.40027600	1.39969100
H	-4.44749000	-1.12255800	1.66160000
H	-3.81242900	0.39354500	2.30309000
H	-1.06196600	-2.26026300	-1.72364200
O	1.30127800	-2.69257200	-0.79826600
C	1.96378300	-3.53680000	0.13198500
H	2.55505300	-4.24332100	-0.45740500
H	2.62354800	-2.97361600	0.80174100
H	1.24376600	-4.10322000	0.74428300
H	0.13672000	-2.03441300	0.79218500

TS1-ax

B3LYP-D3 SCF energy:	-1675.515465 a.u.
B3LYP-D3 enthalpy:	-1674.934386 a.u.
B3LYP-D3 free energy:	-1675.041946 a.u.
M06 SCF energy in solution:	-1674.35079 a.u.
M06 enthalpy in solution (25°C):	-1674.437738 a.u.
M06 free energy in solution (25°C):	-1674.354447 a.u.
M06 enthalpy in solution (90°C):	-1674.458092 a.u.
M06 free energy in solution (90°C):	-1675.515465 a.u.

C	-1.43931300	2.10050200	0.21358600
C	-0.38563900	1.21485500	0.79811300
C	-0.79994900	-0.09424400	1.35941200

O	-1.72499800	-0.76847800	0.56085600
C	-2.83754600	-0.00828900	0.08912200
C	-3.60974800	-0.94137800	-0.82968400
H	-4.53796000	-0.44506100	-1.14939500
O	-2.79223800	-1.25206800	-1.94704800
C	-3.40093700	-2.16480100	-2.83669200
H	-2.69758700	-2.33111600	-3.65696400
H	-3.61378600	-3.12859200	-2.34727400
H	-4.34364800	-1.76719700	-3.24692900
H	-3.86700200	-1.85664600	-0.27432300
C	-2.41659300	1.28333200	-0.63787300
H	-1.92297900	1.01570500	-1.57816100
O	-3.62017200	1.99592200	-0.88873300
C	-3.62528500	2.72785800	-2.10656400
H	-4.60539300	3.20849500	-2.17732700
H	-3.49225700	2.06027000	-2.97231700
H	-2.83792300	3.48965000	-2.12208000
H	-3.48717300	0.27148900	0.93192500
H	0.04188300	-0.77386200	1.48658200
O	-1.39310800	0.13429300	2.68828900
C	-1.36735200	-0.92540300	3.53802600
O	-0.85173400	-1.99300100	3.29026900
C	-2.06396300	-0.57664600	4.83340700
H	-1.99559700	-1.41858100	5.52325100
H	-1.60748000	0.31395300	5.27708000
H	-3.11502100	-0.34063100	4.63554800
H	0.49955900	1.67224500	1.22655700
O	-0.88168600	3.12456300	-0.62141900
C	-0.37045700	4.23513900	0.09496800
H	-0.03431600	4.96252100	-0.64919600
H	-1.14559900	4.69849000	0.72494500
H	0.48473600	3.96489000	0.73168700
H	-2.03908100	2.58396900	1.01009100
C	1.92123500	-0.08305800	-0.60716600
C	0.72719700	0.27721900	-1.15930600
H	0.60337200	1.23373100	-1.64796500
H	-0.05109500	-0.45728400	-1.32389300
C	3.02247200	0.85978400	-0.35077500
C	2.85548600	2.24966800	-0.52238000
C	4.26931100	0.38954200	0.09996700
C	3.90471000	3.12832400	-0.26975700
H	1.89575800	2.64456400	-0.83881500
C	5.31670500	1.27371400	0.35110600
H	4.40273000	-0.67530800	0.25342500
C	5.14284600	2.64680200	0.16621900
H	3.75331500	4.19609600	-0.40713400
H	6.27299700	0.88801400	0.69524100
H	5.95969200	3.33544300	0.36474400
O	2.13714300	-1.33663700	-0.11284600
Si	1.67274100	-2.85371900	-0.73158500
C	1.87888100	-2.80898700	-2.60313600

H	2.91466500	-2.58444600	-2.88310100
H	1.61023600	-3.77527700	-3.04731500
H	1.23770400	-2.04240700	-3.05157800
C	2.89894400	-4.02542700	0.07673800
H	2.81731800	-3.97437100	1.16856400
H	2.71212000	-5.06332700	-0.22456500
H	3.92990900	-3.77332700	-0.19698700
C	-0.08548000	-3.29500500	-0.24929100
H	-0.83204700	-2.70384400	-0.78861900
H	-0.26367900	-4.35681100	-0.46742300
H	-0.26354600	-3.13712800	0.82072000

TS1-eq

B3LYP-D3 SCF energy:	-1675.506965 a.u.
B3LYP-D3 enthalpy:	-1674.925631 a.u.
B3LYP-D3 free energy:	-1675.033489 a.u.
M06 SCF energy in solution:	-1674.345481 a.u.
M06 enthalpy in solution (25°C):	-1674.432285 a.u.
M06 free energy in solution (25°C):	-1674.349179 a.u.
M06 enthalpy in solution (90°C):	-1674.452664 a.u.
M06 free energy in solution (90°C):	-1675.506965 a.u.

C	1.40191400	-1.00180100	0.35214100
C	0.75859300	0.27213200	-0.10503900
C	1.53040800	1.50450200	0.22703600
O	2.85664000	1.43687400	-0.23566200
C	3.58502600	0.28509100	0.21157700
C	4.97565100	0.36369200	-0.39634700
H	5.60741100	-0.39804600	0.08547100
O	4.89463600	0.13967900	-1.79116300
C	6.15475800	0.18933600	-2.42378800
H	5.98980100	-0.00219000	-3.48763400
H	6.63122700	1.17686900	-2.30927800
H	6.84403100	-0.57392900	-2.02508900
H	5.40109000	1.35805100	-0.18460200
C	2.87002100	-1.03200400	-0.15094000
H	2.88817700	-1.14207300	-1.24259500
O	3.62126700	-2.05911000	0.47522500
C	3.60572300	-3.30974000	-0.19599700
H	4.30456600	-3.95923300	0.33929700
H	3.94545900	-3.20556200	-1.23840900
H	2.60731800	-3.76071300	-0.18467600
H	3.69229500	0.32257200	1.30483900
H	1.09742800	2.40675600	-0.20211300
O	1.59900300	1.66934300	1.68263400
C	0.92760200	2.69278200	2.24903500
O	0.18035400	3.44224900	1.65315700
C	1.21435800	2.75725700	3.73030600
H	0.68225400	3.60212300	4.16850700
H	0.89276300	1.82524200	4.20718900

H	2.29090600	2.85748100	3.90048000
H	0.29837800	0.29023700	-1.08675600
O	0.70609800	-2.20417700	0.02019000
C	0.24231100	-2.33061200	-1.31890600
H	-0.01868500	-3.38520100	-1.44778500
H	-0.65260400	-1.72479300	-1.49737400
H	1.01543700	-2.07221800	-2.05587100
H	1.45894000	-1.02031000	1.44763000
C	-2.32298100	0.19994200	0.08682200
C	-1.43185900	0.36129100	1.10096300
H	-1.21380300	1.34633800	1.48609700
H	-1.08635700	-0.48872600	1.67271700
C	-2.85261100	1.29893700	-0.74131900
C	-2.35040100	2.61254200	-0.63728700
C	-3.87428600	1.04618900	-1.67474000
C	-2.87066000	3.63240100	-1.42884900
H	-1.54562900	2.84472000	0.05270800
C	-4.39024800	2.07171800	-2.46467000
H	-4.25544200	0.03649500	-1.77657100
C	-3.89370100	3.37081000	-2.34513600
H	-2.46805400	4.63736800	-1.33308500
H	-5.18190100	1.85397700	-3.17720900
H	-4.29443200	4.17050600	-2.96239100
O	-2.69844000	-1.04862400	-0.33568400
Si	-3.34614000	-2.32204000	0.57805700
C	-4.89802400	-1.67978200	1.42640300
H	-5.64728300	-1.35773200	0.69424900
H	-5.35336000	-2.44536200	2.06626800
H	-4.65674000	-0.81612000	2.05743000
C	-3.72490700	-3.61688900	-0.72880800
H	-2.80458300	-3.94343900	-1.22692400
H	-4.19924000	-4.50126600	-0.28675700
H	-4.39828400	-3.21913500	-1.49639900
C	-2.11181200	-2.99005700	1.83140700
H	-2.07303800	-2.37147200	2.73543900
H	-2.39236500	-4.00556200	2.13886200
H	-1.10544100	-3.02337000	1.39875800

IV-ax

B3LYP-D3 SCF energy:	-1675.568191 a.u.
B3LYP-D3 enthalpy:	-1674.983259 a.u.
B3LYP-D3 free energy:	-1675.090767 a.u.
M06 SCF energy in solution:	-1674.40439 a.u.
M06 enthalpy in solution (25°C):	-1674.491024 a.u.
M06 free energy in solution (25°C):	-1674.407996 a.u.
M06 enthalpy in solution (90°C):	-1674.511409 a.u.
M06 free energy in solution (90°C):	-1675.568191 a.u.

C	-1.43404500	1.91026000	-0.41379300
C	-0.22265300	1.17512300	0.19772000

C	-0.70920200	0.24712300	1.30947400
O	-1.66030100	-0.66724700	0.85038100
C	-2.81794300	-0.11157500	0.21816700
C	-3.60935000	-1.29007100	-0.32285000
H	-4.58210200	-0.92905500	-0.68940200
O	-2.87036800	-1.89430300	-1.37255600
C	-3.48983800	-3.05717400	-1.88275000
H	-2.85102900	-3.43508500	-2.68534600
H	-3.59069300	-3.83530600	-1.10969900
H	-4.48984700	-2.83925400	-2.29166600
H	-3.78078200	-2.00916300	0.49293500
C	-2.48475400	0.90844600	-0.88910300
H	-2.10214900	0.37430300	-1.76643500
O	-3.71860800	1.54611700	-1.19635500
C	-3.88391200	1.88179300	-2.56692700
H	-4.87457200	2.33677300	-2.65808500
H	-3.84456600	0.98438500	-3.20431900
H	-3.11964200	2.59094900	-2.90296900
H	-3.43416700	0.40610500	0.96721800
H	0.09522300	-0.35405000	1.73067500
O	-1.26803300	1.08494900	2.35022100
C	-1.31828200	0.54729100	3.60338300
O	-0.87556300	-0.53795500	3.89497700
C	-1.99469800	1.50819900	4.55176400
H	-2.00087700	1.08492300	5.55665800
H	-1.46915900	2.46861600	4.55018300
H	-3.02086600	1.69694800	4.21882800
H	0.45792300	1.90698500	0.64625500
O	-1.06610700	2.71440000	-1.53228000
C	-0.46983600	3.95259100	-1.18561500
H	-0.29852000	4.49231700	-2.12081700
H	-1.13244300	4.55133300	-0.54173600
H	0.49427200	3.82515600	-0.67212400
H	-1.89913400	2.54342600	0.35634400
C	1.89258800	-0.11177000	-0.36424600
C	0.56008700	0.35684100	-0.86625500
H	0.67144400	0.98125500	-1.75649700
H	-0.04335600	-0.50834000	-1.15857500
C	3.07240300	0.68131900	-0.36383400
C	3.11156300	1.97435100	-0.96362100
C	4.27168700	0.20616100	0.24198200
C	4.27593700	2.72934300	-0.95875600
H	2.22332300	2.37588900	-1.44047100
C	5.42649800	0.97361700	0.23804100
H	4.26067000	-0.76797500	0.71818000
C	5.44515900	2.24146500	-0.36044800
H	4.27610500	3.71096400	-1.42678300
H	6.32653100	0.58542100	0.70908400
H	6.35300600	2.83792800	-0.35966100
O	1.92124800	-1.29513300	0.31520600
Si	1.60998100	-2.87092400	-0.24326400

C	1.77201400	-2.87313900	-2.12014500
H	2.73124000	-2.44403600	-2.43220000
H	1.71477700	-3.89509000	-2.51454100
H	0.97366400	-2.28931100	-2.59230100
C	2.94404000	-3.92040400	0.56422700
H	2.90321600	-3.82360900	1.65541500
H	2.82221700	-4.98217700	0.31739300
H	3.94244600	-3.60823400	0.23663200
C	-0.10091000	-3.40928500	0.30412500
H	-0.88710600	-2.86181500	-0.22491700
H	-0.23733600	-4.48374700	0.12361200
H	-0.24491500	-3.22413400	1.37482700

IV-eq

B3LYP-D3 SCF energy:	-1675.56764 a.u.
B3LYP-D3 enthalpy:	-1674.982699 a.u.
B3LYP-D3 free energy:	-1675.088322 a.u.
M06 SCF energy in solution:	-1674.403005 a.u.
M06 enthalpy in solution (25°C):	-1674.489906 a.u.
M06 free energy in solution (25°C):	-1674.406313 a.u.
M06 enthalpy in solution (90°C):	-1674.51018 a.u.
M06 free energy in solution (90°C):	-1675.56764 a.u.

C	1.73857500	-0.53951800	1.27698700
C	0.53926700	-0.37116900	0.33586900
C	3.07473500	0.83386300	-0.36658800
C	3.03591400	-0.45284600	0.47311600
H	0.56236200	-1.21485800	-0.36504700
H	1.73228400	0.25653100	2.03660000
H	3.10615000	1.69294600	0.31903500
H	3.06938400	-1.31304900	-0.20447600
O	1.91381800	0.93229100	-1.20476400
O	4.15956000	-0.45462500	1.35253200
O	1.62520700	-1.81707000	1.90097300
C	4.29731400	0.91912600	-1.26355700
H	5.18582600	1.08611000	-0.63380500
H	4.17977700	1.78463400	-1.93477300
O	4.43145000	-0.27830900	-2.00687400
C	5.48899900	-0.22404000	-2.94177200
H	5.51546900	-1.18943500	-3.45392500
H	5.33193100	0.57238400	-3.68667500
H	6.46129300	-0.05185400	-2.45049100
C	5.08971400	-1.49810900	1.09456100
H	4.64210300	-2.48557400	1.27866300
H	5.92694300	-1.35111800	1.78303700
H	5.45445600	-1.46219600	0.05948300
C	2.07181900	-1.85764100	3.24835200
H	1.45951800	-1.20686600	3.89287800
H	1.95779400	-2.89224300	3.58445800
H	3.12102500	-1.55439300	3.33710000

C	0.69851300	0.88962400	-0.51756900
H	-0.08688000	0.98222000	-1.26608200
C	-1.97128600	-0.64858800	0.17280800
C	-2.35148800	-1.93690700	-0.29816900
O	-2.50002400	0.45959800	-0.43494700
C	-3.38853400	-2.09132400	-1.26216300
C	-1.71129900	-3.12236400	0.17079600
Si	-3.69720900	1.50429900	0.15131900
C	-3.77680000	-3.34828100	-1.70040500
H	-3.86740900	-1.20400100	-1.65955100
C	-2.11109900	-4.37228900	-0.28082800
H	-0.88553900	-3.05263400	0.87209400
C	-3.86444500	2.81009300	-1.17952200
C	-5.27957200	0.51796300	0.41173900
C	-3.15059100	2.25772800	1.79216400
C	-3.14933000	-4.50271200	-1.21247100
H	-4.57336000	-3.43490000	-2.43562000
H	-1.60389400	-5.25896500	0.09181500
H	-2.90053300	3.30005900	-1.35341700
H	-4.19001500	2.36280200	-2.12563800
H	-4.59973400	3.57329800	-0.89640700
H	-5.68710000	0.15719900	-0.53911200
H	-5.09138800	-0.35899200	1.04227500
H	-6.05069900	1.12602500	0.90080700
H	-3.89684500	2.97758200	2.15098700
H	-3.03019300	1.49240400	2.56768200
H	-2.19779900	2.78806500	1.69095000
H	-3.45680000	-5.48504600	-1.55971700
O	0.61394800	2.02808500	0.38726600
C	0.07629300	3.18040700	-0.09001000
O	-0.43445800	3.29994200	-1.17979200
C	0.21809100	4.28467900	0.93161800
H	-0.39770900	5.13528100	0.63701600
H	-0.06490200	3.93181200	1.92740800
H	1.26786000	4.59584600	0.98007900
C	-0.81067400	-0.40587200	1.09077600
H	-0.94536100	0.54311700	1.61746300
H	-0.74781200	-1.19260700	1.84689000

IV'-ax

B3LYP-D3 SCF energy:	-6638.480175 a.u.
B3LYP-D3 enthalpy:	-6637.250546 a.u.
B3LYP-D3 free energy:	-6637.440982 a.u.
M06 SCF energy in solution:	-6638.383357 a.u.
M06 enthalpy in solution (25°C):	-6638.535665 a.u.
M06 free energy in solution (25°C):	-6638.390804 a.u.
M06 enthalpy in solution (90°C):	-6638.572066 a.u.
M06 free energy in solution (90°C):	-6638.480175 a.u.

C	-5.02560400	-0.33753800	-1.34593900

C	-4.09943100	0.76687200	-0.80096400
C	-5.33980200	-1.08808500	1.07059700
C	-5.07349900	-1.52967700	-0.38306100
H	-6.04464400	0.06582900	-1.43677800
H	-6.39844300	-0.80513100	1.14165100
H	-4.11710900	-2.05583400	-0.43599000
O	-4.52822800	0.02319800	1.47756400
O	-6.14126600	-2.40713500	-0.75124800
O	-4.54449100	-0.73944300	-2.62270700
C	-5.07350600	-2.19776700	2.07424300
H	-5.84008200	-2.98117200	1.96284900
H	-5.15327600	-1.77659900	3.08837500
O	-3.77984000	-2.72961000	1.85348900
C	-3.36456300	-3.62209400	2.86604200
H	-2.36707700	-3.96910200	2.59055400
H	-3.32395700	-3.12471800	3.84835700
H	-4.03719300	-4.49223500	2.94321100
C	-5.75317800	-3.75691900	-0.94603100
H	-5.16547800	-3.87869000	-1.86757800
H	-6.67398500	-4.34134400	-1.03442400
H	-5.15854700	-4.13722200	-0.10458400
C	-5.55109900	-1.09246400	-3.55692200
H	-6.22971800	-0.24676000	-3.75298300
H	-5.03624100	-1.35165100	-4.48617900
H	-6.14912100	-1.94531600	-3.21490400
C	-4.54455600	1.11378800	0.62013900
C	-2.61192300	0.32520900	-0.88963100
H	-2.51869800	-0.70614500	-0.55638600
H	-2.32289000	0.31964100	-1.93825900
C	-1.64543300	1.19519000	-0.09208400
C	-1.50705000	0.95330500	1.34529700
O	-1.62383200	2.51101300	-0.44602900
C	-1.32713200	2.01065200	2.28180800
C	-1.44499500	-0.39023200	1.82205500
Si	-1.49602400	3.42179100	-1.88903800
C	-1.08022300	1.74039300	3.61123300
H	-1.41670300	3.03019800	1.93155700
C	-1.17416600	-0.63807800	3.18520400
H	-1.80161200	-1.21860300	1.22392700
C	-2.28898600	2.60097200	-3.38479100
C	-2.40111400	4.99876300	-1.40869000
C	0.30688800	3.80500100	-2.26490900
C	-0.98121000	0.40714800	4.06937000
H	-0.96529300	2.56224000	4.31318500
H	-1.14730900	-1.66687900	3.53163100
H	-1.69413800	1.73859300	-3.69977200
H	-3.32104200	2.27489300	-3.22423500
H	-2.29433000	3.32901200	-4.20733100
H	-1.90388300	5.47970600	-0.55676600
H	-2.40033900	5.71562500	-2.23966000
H	-3.43594200	4.79552800	-1.11796900

H	0.38584100	4.14594300	-3.30575000
H	0.70714500	4.59372500	-1.62010000
H	0.93016800	2.91256900	-2.16693600
H	-0.78254200	0.20895200	5.11936400
H	-4.23586600	1.66852000	-1.40289200
C	3.76078600	3.07956600	-1.11598200
C	3.40455400	1.83127900	-0.58444300
C	3.86609400	0.69455800	-1.26189600
C	4.68606200	0.74534200	-2.39387200
C	5.00187900	2.00960400	-2.89953400
C	4.53839100	3.16551200	-2.26955500
C	4.17069500	-1.64975900	-2.75848200
C	3.35393400	-1.57217200	-1.62857100
C	2.35644300	-2.50761100	-1.33484900
C	2.20254600	-3.59406400	-2.20313700
C	3.00906200	-3.70703200	-3.33575200
C	3.97496700	-2.73829600	-3.61447600
H	3.41281400	3.98691400	-0.63576800
H	5.61938600	2.10095200	-3.78643800
H	4.79320100	4.14054600	-2.67520600
H	1.43227700	-4.33334500	-2.00914000
H	2.87834300	-4.54812400	-4.01074600
H	4.58072700	-2.83901200	-4.50860200
C	5.24826300	-0.57611900	-2.93159900
O	3.49744800	-0.52979600	-0.73928200
P	1.25758500	-2.14093200	0.08988300
P	2.21897000	1.62964000	0.81604300
C	-0.12265200	-3.34406200	-0.16527400
C	-0.30992700	-4.49305100	0.61482300
C	-1.04844300	-3.05273500	-1.18530000
C	-1.40535700	-5.33262400	0.38334000
H	0.39988700	-4.74036500	1.39836800
C	-2.13582800	-3.89272700	-1.41241200
H	-0.90554300	-2.17046400	-1.80334600
C	-2.32214700	-5.03305800	-0.62443200
H	-1.53850200	-6.22158400	0.99506600
H	-2.83962700	-3.64201400	-2.20095700
H	-3.17485000	-5.68420700	-0.79783000
C	2.18238000	-2.84567100	1.51475800
C	1.94821300	-2.30971600	2.78749000
C	3.08470400	-3.91259700	1.37626400
C	2.58556900	-2.84525900	3.90731400
H	1.28545100	-1.45886200	2.89526000
C	3.72813900	-4.44033600	2.49616200
H	3.28290700	-4.32776500	0.39233900
C	3.47467400	-3.91150900	3.76470000
H	2.40648300	-2.40886800	4.88521400
H	4.42734200	-5.26430900	2.37825800
H	3.97848800	-4.32191600	4.63607700
C	2.09122800	3.37579000	1.42381500
C	3.09913200	3.99940500	2.17893500

C	0.94670300	4.11100500	1.09260100
C	2.95830700	5.32487600	2.59000300
H	3.99425600	3.44570600	2.44597000
C	0.80500800	5.43958000	1.50081500
H	0.16409000	3.63526100	0.51738200
C	1.81041800	6.04902900	2.25229400
H	3.74645800	5.79468400	3.17305200
H	-0.09239400	5.99181700	1.23273800
H	1.70272800	7.08136500	2.57508600
C	3.23041500	0.91966500	2.19270500
C	2.64328200	0.94279200	3.47079300
C	4.50798700	0.36414700	2.05165800
C	3.32383600	0.44356400	4.57810000
H	1.64841000	1.36149700	3.59559700
C	5.17963100	-0.15934600	3.16027800
H	4.99078100	0.34474700	1.08155500
C	4.59583700	-0.11654700	4.42582400
H	2.85689400	0.48232500	5.55950900
H	6.16736800	-0.59415900	3.03052000
H	5.12394000	-0.51940400	5.28600100
Pd	0.22203200	0.05875400	0.03390400
Br	0.45974400	0.15509000	-2.53854000
O	-5.91892500	1.61758300	0.59102800
C	-6.09568500	2.92975400	0.32815000
C	-7.56198600	3.29103900	0.37753100
O	-5.19168900	3.70065100	0.08418600
H	-8.11559200	2.70074100	-0.36037700
H	-7.97345200	3.05111000	1.36332200
H	-7.68166200	4.35483000	0.16953700
H	-3.91722000	1.88371500	1.06598500
C	5.71304000	-0.45567600	-4.39093200
H	6.50401300	0.29447700	-4.48539500
H	6.13674900	-1.40141900	-4.74231800
H	4.88557100	-0.17673500	-5.05123900
C	6.46379800	-0.97024300	-2.04483600
H	6.88294200	-1.92709300	-2.37743600
H	7.24381900	-0.20242200	-2.10517400
H	6.16366300	-1.07483400	-0.99705600

TS3

B3LYP-D3 SCF energy:	-6638.4565 a.u.
B3LYP-D3 enthalpy:	-6637.233658 a.u.
B3LYP-D3 free energy:	-6637.42466 a.u.
M06 SCF energy in solution:	-6638.35817 a.u.
M06 enthalpy in solution (25°C):	-6638.510177 a.u.
M06 free energy in solution (25°C):	-6638.365783 a.u.
M06 enthalpy in solution (90°C):	-6638.546531 a.u.
M06 free energy in solution (90°C):	-6638.4565 a.u.

H	-0.77062500	0.02374800	0.10700400

C	3.67948000	3.65001500	0.04900500
C	3.12087300	2.38559200	0.28357600
C	3.99778800	1.28735000	0.31606300
C	5.37894900	1.41193200	0.12118000
C	5.88922000	2.68720900	-0.13369500
C	5.04779300	3.79905500	-0.16470500
C	5.43365700	-1.01676700	-0.32222200
C	4.05490100	-1.03261000	-0.09318800
C	3.22440400	-2.08907100	-0.49855000
C	3.82137600	-3.15665300	-1.18013500
C	5.19068800	-3.15527900	-1.44490500
C	5.98939200	-2.09468700	-1.01826600
H	3.03504900	4.52094300	0.00880600
H	6.95166900	2.82169400	-0.30475900
H	5.45973000	4.78520000	-0.35937700
H	3.20749200	-3.98263500	-1.52324200
H	5.63672300	-3.98478100	-1.98641000
H	7.05293600	-2.11359400	-1.22983600
C	6.23088400	0.14772300	0.27174600
O	3.47026300	0.03478900	0.56945800
P	1.41051300	-1.99475300	-0.15524600
P	1.29404000	2.13995900	0.43085700
C	0.75566900	-3.45540900	-1.06078200
C	0.64748100	-4.75746900	-0.55007800
C	0.35601900	-3.20188100	-2.38224300
C	0.15136500	-5.78843600	-1.35150100
H	0.94802600	-4.97179700	0.47010000
C	-0.13717800	-4.23373100	-3.18126200
H	0.42246800	-2.19500300	-2.78139700
C	-0.23728900	-5.52985200	-2.66905500
H	0.06550600	-6.79250500	-0.94364900
H	-0.46104200	-4.00668500	-4.19227300
H	-0.62931400	-6.33344600	-3.28730000
C	1.39740800	-2.43098200	1.63989800
C	0.63245800	-1.65330000	2.51838700
C	2.19700900	-3.46043500	2.16571600
C	0.64313900	-1.91278900	3.89062300
H	0.05644500	-0.81942500	2.13437000
C	2.19426300	-3.73166400	3.53406000
H	2.84217300	-4.03405400	1.50582100
C	1.41370400	-2.95901900	4.39921900
H	0.05960800	-1.28106400	4.55291400
H	2.81328000	-4.53447700	3.92662400
H	1.42360300	-3.16118600	5.46739800
C	0.62105500	3.77508900	-0.07024000
C	0.41327000	4.84262800	0.81544700
C	0.34661100	3.94645300	-1.43615700
C	-0.05925600	6.06827100	0.34009600
H	0.62390000	4.71697700	1.87366100
C	-0.12041200	5.17379800	-1.90752100
H	0.50978900	3.11843100	-2.12094500

C	-0.32607800	6.23569100	-1.02200700
H	-0.21205200	6.89319100	1.03186500
H	-0.32736100	5.29724700	-2.96717800
H	-0.69056500	7.19114500	-1.39098900
C	1.00255500	2.05025400	2.24443400
C	-0.32186100	2.08547500	2.71550800
C	2.03854700	1.80329800	3.15712900
C	-0.59842200	1.88386000	4.06663900
H	-1.13791300	2.26740800	2.02115500
C	1.75792300	1.59384100	4.50847800
H	3.06682700	1.76702500	2.81360900
C	0.44091500	1.63360600	4.96748200
H	-1.62642600	1.90175200	4.41164400
H	2.57238300	1.39583900	5.20043200
H	0.22138700	1.46528300	6.01869200
Pd	0.76658600	0.17671300	-0.68166500
Br	2.10038400	0.59779100	-2.90329500
C	7.61295200	0.29805500	-0.38067800
H	8.17364200	1.11539800	0.08317400
H	8.20759800	-0.60935300	-0.23769500
H	7.53047300	0.49588300	-1.45436900
C	6.42110000	-0.12576300	1.79147600
H	6.99889900	-1.04514000	1.94145900
H	6.95550700	0.70747000	2.26274500
H	5.45477000	-0.24358800	2.29155300
C	-4.05001800	-0.04953100	-1.57277700
C	-2.53299700	-0.21547300	-1.35608600
C	-3.53678700	2.46720200	-1.75083300
C	-4.46523200	1.36530700	-1.17416000
H	-4.26475200	-0.19978900	-2.63778400
H	-3.90966400	2.70700500	-2.75478000
H	-4.43786100	1.40230300	-0.08503900
O	-2.14879200	2.11335200	-1.84291500
O	-5.79153000	1.65932600	-1.61686300
O	-4.77859700	-1.00939300	-0.80946900
C	-3.58295100	3.72078900	-0.88789300
H	-4.58872600	4.16706400	-0.95122900
H	-2.84655200	4.44548600	-1.26050400
O	-3.28412400	3.36016900	0.45517400
C	-3.27317200	4.45992300	1.34570300
H	-3.01250100	4.06470500	2.33165800
H	-2.52312300	5.20539300	1.05203400
H	-4.26238700	4.94365200	1.39951000
C	-6.70333000	1.91071900	-0.56022500
H	-6.82352800	1.03208800	0.09003200
H	-7.66583600	2.14979400	-1.02146500
H	-6.38427000	2.76217900	0.06023000
C	-5.93508100	-1.51189600	-1.46733300
H	-5.66575100	-2.05906300	-2.38365900
H	-6.41459200	-2.20670100	-0.77313500
H	-6.63211000	-0.70832300	-1.72873100

C	-1.86064900	0.82290900	-2.27081400
H	-0.76960100	0.72353500	-2.27366100
O	-2.30494100	0.75027500	-3.65682600
C	-1.95440700	-0.32067900	-4.40476200
O	-1.47858100	-1.34109200	-3.95642400
C	-2.22865100	-0.06052200	-5.86624900
H	-2.22190800	-1.00495200	-6.41235900
H	-3.18195800	0.45827900	-6.00049500
H	-1.43626000	0.58693500	-6.25899100
C	-2.13870200	0.06754900	0.10611300
H	-2.28731000	1.10477600	0.38798500
C	-2.47783100	-0.85184100	1.11277500
H	-2.22302300	-1.21735800	-1.65181600
O	-2.21125900	-2.14389000	0.88471600
Si	-3.15492400	-3.56929000	0.86569800
C	-2.17087500	-4.80719500	1.88122100
C	-4.83316300	-3.20963500	1.63934800
C	-3.27365200	-4.08280100	-0.93060700
H	-2.57542100	-5.82036600	1.76511900
H	-1.12761800	-4.82045500	1.55289400
H	-2.18520700	-4.55903200	2.94852700
H	-4.77548200	-3.15785800	2.73132900
H	-5.20837500	-2.24881200	1.27447200
H	-5.55674800	-3.98947700	1.37058800
H	-3.82151200	-5.02608000	-1.04806500
H	-3.78702000	-3.30777800	-1.50621800
H	-2.27505200	-4.21406400	-1.35777900
C	-2.91315300	-0.45863000	2.45268900
C	-2.70364800	-1.31296600	3.55510900
C	-3.61538800	0.74840100	2.66884900
C	-3.17796300	-0.97803100	4.82011300
H	-2.13255900	-2.22231000	3.41346900
C	-4.10069100	1.06990000	3.93291700
H	-3.78792800	1.43378000	1.84732100
C	-3.88891600	0.20958000	5.01621500
H	-2.99138300	-1.64638400	5.65657200
H	-4.65241600	1.99598800	4.07303000
H	-4.27195100	0.46322700	6.00077300

TS2

B3LYP-D3 SCF energy:	-6638.452486 a.u.
B3LYP-D3 enthalpy:	-6637.228035 a.u.
B3LYP-D3 free energy:	-6637.415435 a.u.
M06 SCF energy in solution:	-6638.353997 a.u.
M06 enthalpy in solution (25°C):	-6638.505803 a.u.
M06 free energy in solution (25°C):	-6638.361102 a.u.
M06 enthalpy in solution (90°C):	-6638.541877 a.u.
M06 free energy in solution (90°C):	-6638.452486 a.u.

C	-2.18182300	-0.22202900	-0.12660000

H	-0.87667700	0.09566800	0.32532100
H	-2.39799900	-0.86274500	0.72343800
C	-2.10622800	-0.96264300	-1.32608200
C	-2.18230900	-2.44603000	-1.29007200
C	-3.13907200	-3.08594200	-0.48018900
C	-1.33813200	-3.23343200	-2.09207300
C	-3.28144800	-4.47227600	-0.51666300
H	-3.77549400	-2.50986100	0.18165000
C	-1.48347700	-4.61867200	-2.12210500
H	-0.52174300	-2.75791400	-2.62279000
C	-2.46847000	-5.24315000	-1.35081900
H	-4.03178800	-4.94714900	0.10930800
H	-0.81042700	-5.21042800	-2.73668900
H	-2.58764800	-6.32306900	-1.38456200
O	-2.05871800	-0.33550800	-2.50814300
Si	-2.55056400	-0.81462000	-4.09074500
C	-1.29141700	-1.87813400	-4.99101300
H	-0.27605500	-1.62526400	-4.66269700
H	-1.44756800	-2.94925300	-4.83567300
H	-1.36530500	-1.67386400	-6.06711600
C	-4.23103800	-1.63911300	-3.90142400
H	-4.84389700	-1.08533100	-3.18200500
H	-4.76131500	-1.68144100	-4.86077900
H	-4.12184400	-2.66306600	-3.52837600
C	-2.62361800	0.81548200	-5.02262000
H	-2.93402800	0.62956800	-6.05840900
H	-3.31449200	1.54053300	-4.58811200
H	-1.62470700	1.26506800	-5.05582100
C	3.43624400	2.56393700	-2.47987200
C	3.03564900	1.82563000	-1.35940400
C	3.92872400	0.86007000	-0.87217200
C	5.18425500	0.61623300	-1.43489600
C	5.53967100	1.36590500	-2.55970500
C	4.67462900	2.33093600	-3.07658500
C	5.21384600	-1.52399200	-0.18333000
C	3.95459100	-1.17766100	0.31239000
C	3.07970600	-2.09462500	0.91347000
C	3.51603200	-3.42090100	1.02869500
C	4.76281000	-3.80111400	0.53269000
C	5.60077500	-2.86310400	-0.07264400
H	2.76908200	3.30555100	-2.90455900
H	6.49819000	1.20284200	-3.04029900
H	4.96674700	2.90404100	-3.95202700
H	2.86965200	-4.16086500	1.48845200
H	5.08463900	-4.83517600	0.61916400
H	6.56495100	-3.18264400	-0.45300000
C	6.09540400	-0.39690700	-0.73208800
O	3.52837300	0.13266000	0.23217100
P	1.38119300	-1.52788600	1.34279200
P	1.34548600	1.93014600	-0.62278600
C	0.62194500	-3.04685000	2.06485500

C	0.56546700	-3.30417300	3.44360100
C	0.05813100	-3.97483400	1.17513300
C	-0.04358000	-4.46673200	3.92075600
H	0.99564800	-2.59584000	4.14455500
C	-0.53092400	-5.14602100	1.65345100
H	0.07730800	-3.77531600	0.10797400
C	-0.58843700	-5.39234600	3.02762600
H	-0.08683000	-4.65081700	4.99131900
H	-0.96080800	-5.85156500	0.94987600
H	-1.06074400	-6.29744800	3.40069900
C	1.62727800	-0.47509800	2.83536300
C	0.54510700	0.31249100	3.25645000
C	2.82240800	-0.42895000	3.56525600
C	0.64666700	1.13533400	4.37574900
H	-0.38412800	0.28693100	2.69844000
C	2.92760800	0.39425300	4.68935700
H	3.66995700	-1.03337900	3.25497300
C	1.84438700	1.17669300	5.09448300
H	-0.19711000	1.75955500	4.65302800
H	3.86148500	0.42702500	5.24537900
H	1.93715300	1.82840600	5.95932000
C	0.49627700	3.19415400	-1.66917900
C	0.80392800	4.56407400	-1.59956000
C	-0.50593000	2.76507000	-2.55172800
C	0.11719700	5.48713000	-2.38751700
H	1.57709400	4.90790200	-0.91908400
C	-1.19483500	3.69406700	-3.33830700
H	-0.74550200	1.70673000	-2.61394300
C	-0.88809400	5.05301000	-3.25582700
H	0.36556800	6.54339500	-2.32287800
H	-1.96953700	3.35530200	-4.01803600
H	-1.42847600	5.77175700	-3.86653700
C	1.61012600	2.89125800	0.93896400
C	0.55193600	3.64950400	1.46889200
C	2.82027200	2.85086200	1.64776800
C	0.70613100	4.36187500	2.65764900
H	-0.39268300	3.70745700	0.93465600
C	2.96975800	3.55862500	2.84024700
H	3.65264800	2.26751500	1.27699300
C	1.91760400	4.32031400	3.34789400
H	-0.12757500	4.92987200	3.05651900
H	3.91406800	3.50645600	3.37525300
H	2.03549000	4.86815100	4.27891000
Pd	0.47400700	-0.26938200	-0.48268300
Br	1.67234500	-1.25796500	-2.56674400
C	-2.79987000	1.17218700	0.00954200
C	-4.34050200	1.16918700	-0.13422100
C	-2.49389300	1.65858700	1.43449400
H	-2.35681700	1.86213500	-0.71306600
C	-4.96974000	0.25027900	0.90679300
O	-4.77652200	0.74707900	-1.42057200

H	-4.70767700	2.18634200	0.05401500
O	-3.00289300	0.77190900	2.38455500
H	-1.42448000	1.72445900	1.63330800
O	-3.06197100	2.98159600	1.56776900
C	-4.41067400	0.49160200	2.32798100
H	-4.77140100	-0.78491900	0.61297200
O	-6.37092800	0.46331500	1.01901300
C	-4.88562000	1.81552000	-2.34462800
C	-2.76445600	3.67065100	2.70545100
C	-4.63546300	-0.74270900	3.19951200
H	-4.96281000	1.34258300	2.75160000
C	-7.16453500	-0.40898700	0.22647000
H	-5.21062300	1.38316500	-3.29346100
H	-5.63171400	2.55319100	-2.01221900
H	-3.92472900	2.32735800	-2.49561000
O	-2.06117900	3.24829900	3.59251200
C	-3.41359200	5.03338900	2.67021600
H	-5.71566400	-0.91604100	3.26287700
O	-4.04698300	-1.90284800	2.62934300
H	-4.24751800	-0.55516600	4.20967700
H	-8.20806000	-0.15796100	0.43726000
H	-6.99047300	-1.46244100	0.49653600
H	-6.96191000	-0.28015000	-0.84241800
H	-4.47690600	4.94556400	2.42759500
H	-3.28280200	5.52380800	3.63551900
H	-2.94613500	5.63719600	1.88400000
C	-2.77355000	-2.26071400	3.16334900
H	-2.40429900	-3.09615100	2.56563000
H	-2.05810700	-1.43409100	3.10648900
H	-2.86218600	-2.58266200	4.21143300
C	6.76124100	0.31817900	0.47813800
H	7.40349200	-0.38113000	1.02614300
H	7.37041600	1.16205300	0.13395900
H	6.00367200	0.70029300	1.17015800
C	7.19496300	-0.92661900	-1.66491600
H	7.83166000	-0.10920700	-2.01692300
H	7.84915100	-1.62566600	-1.13509400
H	6.76830100	-1.43716500	-2.53427500

LPd^{I}Br

B3LYP-D3 SCF energy:	-4962.880777 a.u.
B3LYP-D3 enthalpy:	-4962.239963 a.u.
B3LYP-D3 free energy:	-4962.356307 a.u.
M06 SCF energy in solution:	-4963.939977 a.u.
M06 enthalpy in solution (25°C):	-4964.043314 a.u.
M06 free energy in solution (25°C):	-4963.954047 a.u.
M06 enthalpy in solution (90°C):	-4964.065459 a.u.
M06 free energy in solution (90°C):	-4962.880777 a.u.

C	-3.32341600	1.77753200	-1.29265000

C	-2.13937100	1.39170400	-0.64618600
C	-1.04598800	2.25784300	-0.74921600
C	-1.05511700	3.45020000	-1.47888300
C	-2.24933900	3.79228900	-2.11955500
C	-3.37365500	2.96854100	-2.01711600
C	1.40616100	3.29408600	-1.53702800
C	1.28020600	2.11858800	-0.79511300
C	2.26555900	1.13046700	-0.71694100
C	3.46128600	1.35534500	-1.41220400
C	3.62370400	2.52072000	-2.16364600
C	2.60644600	3.47716200	-2.23202500
H	-4.19646000	1.13611700	-1.24947100
H	-2.31084700	4.70337300	-2.70506800
H	-4.29450200	3.25222700	-2.51845800
H	4.25189800	0.61320700	-1.37846700
H	4.54997000	2.68361400	-2.70701000
H	2.75644300	4.36839200	-2.83203400
C	0.23443100	4.28322300	-1.48039400
O	0.11139400	1.87696200	-0.09829400
P	1.83294800	-0.41630100	0.18037200
P	-1.87973000	-0.25335200	0.15396500
C	3.39140200	-1.39382200	0.07868900
C	4.48878300	-1.11030300	0.90955600
C	3.49580000	-2.41604600	-0.87659600
C	5.67292000	-1.83547500	0.78081400
H	4.41651400	-0.32613400	1.65748100
C	4.68569900	-3.13697900	-1.00188200
H	2.64729900	-2.66098800	-1.50928200
C	5.77363300	-2.84937900	-0.17641100
H	6.51553300	-1.61094800	1.42963000
H	4.75346300	-3.93001000	-1.74142500
H	6.69614500	-3.41600900	-0.27259000
C	1.78126500	0.09736100	1.94322900
C	1.27018800	-0.82468000	2.86883400
C	2.25162100	1.33670000	2.39851000
C	1.25114500	-0.52027100	4.22822600
H	0.87611300	-1.77514800	2.51860400
C	2.21997200	1.64367600	3.76039300
H	2.64655800	2.06107800	1.69249300
C	1.72523300	0.71477900	4.67691500
H	0.84351500	-1.23754700	4.93389900
H	2.58557900	2.60814700	4.10343300
H	1.69919400	0.95509500	5.73641100
C	-3.49106400	-1.10234800	-0.13892800
C	-4.64387200	-0.76298000	0.59143400
C	-3.57591800	-2.08324100	-1.13819400
C	-5.85850500	-1.39237700	0.32278300
H	-4.58883700	-0.00951500	1.37175400
C	-4.79589200	-2.71079400	-1.40320200
H	-2.69030600	-2.37173500	-1.69846100
C	-5.93623000	-2.36789200	-0.67614500

H	-6.74314000	-1.12349900	0.89408900
H	-4.84657100	-3.47395700	-2.17473000
H	-6.88285600	-2.86083300	-0.88142100
C	-1.93425700	0.06877400	1.96501800
C	-2.20727200	-1.01839400	2.81337100
C	-1.67398100	1.32370600	2.52991700
C	-2.24741100	-0.84450900	4.19512400
H	-2.40706500	-1.99858000	2.38832300
C	-1.70107600	1.49067400	3.91571200
H	-1.45172600	2.17397300	1.89543500
C	-1.99487300	0.41253800	4.75075700
H	-2.47339500	-1.69146400	4.83765200
H	-1.49115700	2.46826600	4.34082900
H	-2.01989200	0.54809100	5.82864500
Pd	-0.06576300	-1.47693000	-0.70442000
Br	0.02204800	-3.36646400	-2.31510500
C	0.27083100	5.29023400	-2.63841000
H	-0.56003000	5.99826700	-2.56127100
H	1.19179200	5.88053400	-2.60692600
H	0.21198500	4.78868200	-3.60997100
C	0.31784400	5.05600400	-0.13359200
H	1.24590200	5.63714200	-0.08803300
H	-0.53141600	5.74183300	-0.03685500
H	0.30292200	4.36653000	0.71622400

LPd^{II}HBr

B3LYP-D3 SCF energy:	-4963.473285 a.u.
B3LYP-D3 enthalpy:	-4962.824303 a.u.
B3LYP-D3 free energy:	-4962.937482 a.u.
M06 SCF energy in solution:	-4964.532112 a.u.
M06 enthalpy in solution (25°C):	-4964.625134 a.u.
M06 free energy in solution (25°C):	-4964.535714 a.u.
M06 enthalpy in solution (90°C):	-4964.647089 a.u.
M06 free energy in solution (90°C):	-4963.473285 a.u.

C	-3.29383400	1.87328600	-0.48530000
C	-2.10926800	1.29928600	-0.00981000
C	-1.04851700	2.16281100	0.28167600
C	-1.10506400	3.54979200	0.14407300
C	-2.30396600	4.08777800	-0.33600500
C	-3.38376500	3.25713000	-0.64722300
C	1.36364500	3.53996900	0.13194300
C	1.29056200	2.14812800	0.23795600
C	2.32403900	1.28050800	-0.13910600
C	3.51226800	1.86175600	-0.60386200
C	3.62709900	3.24886600	-0.69941800
C	2.56026600	4.07957400	-0.34895400
H	-4.13074300	1.23767600	-0.75258600
H	-2.40219300	5.15913300	-0.47493500
H	-4.30277900	3.69321300	-1.02777200

H	4.33593400	1.23168100	-0.91980600
H	4.55041700	3.68607600	-1.06820800
H	2.66712400	5.15355400	-0.45685500
C	0.13427900	4.34497400	0.57624900
O	0.12932100	1.57169000	0.71369500
P	1.95895200	-0.53067700	-0.22962700
P	-1.75111600	-0.50158400	0.09834400
C	3.53356700	-1.26536400	-0.84977300
C	4.68324400	-1.23783000	-0.03810700
C	3.60977200	-1.86211900	-2.11426500
C	5.88351100	-1.77947100	-0.49276900
H	4.63589200	-0.79349500	0.95194900
C	4.81344500	-2.41145000	-2.56512800
H	2.72668700	-1.89925600	-2.74374800
C	5.95064200	-2.36898900	-1.75925500
H	6.76468400	-1.74654000	0.14230500
H	4.85548900	-2.87453100	-3.54705300
H	6.88554200	-2.79686800	-2.11122500
C	1.98831500	-1.13519500	1.51081000
C	2.14674400	-2.51652500	1.71322600
C	1.88843900	-0.28990600	2.62218100
C	2.23413900	-3.03647100	3.00247000
H	2.22593100	-3.18329200	0.85839300
C	1.96573400	-0.81615000	3.91312300
H	1.75530600	0.77715800	2.48888500
C	2.14797300	-2.18513600	4.10696900
H	2.36960800	-4.10536500	3.14448900
H	1.87952500	-0.15103000	4.76758300
H	2.21228400	-2.58922800	5.11358700
C	-3.36173400	-1.34179200	-0.18215100
C	-4.47380600	-1.05141000	0.62686500
C	-3.46386600	-2.34215900	-1.15747800
C	-5.67524800	-1.73349200	0.44483400
H	-4.39357600	-0.29833500	1.40624100
C	-4.66726200	-3.03102000	-1.32978000
H	-2.61398100	-2.56277000	-1.79298000
C	-5.77306300	-2.72548600	-0.53609300
H	-6.53112200	-1.49818300	1.07201200
H	-4.73793900	-3.80172000	-2.09239100
H	-6.70905000	-3.26006800	-0.67607600
C	-1.56611900	-0.79057900	1.91204300
C	-1.22378200	-2.08716300	2.32318100
C	-1.82439800	0.18450900	2.88439000
C	-1.16408200	-2.40754700	3.67748500
H	-1.00411300	-2.84862400	1.57924300
C	-1.75331200	-0.13552000	4.24209200
H	-2.09253200	1.19328300	2.58680400
C	-1.43058200	-1.43242700	4.64169800
H	-0.89329600	-3.41474700	3.98020100
H	-1.95845800	0.63020700	4.98600800
H	-1.37893800	-1.68199200	5.69817800

Br	-1.49182300	-0.55407000	-3.44580200
Pd	0.07604100	-0.75590900	-1.51526200
H	1.13685900	-0.70046800	-2.65611400
C	0.13966100	4.42516000	2.12919100
H	1.03538000	4.95071000	2.47953000
H	-0.74568500	4.96582100	2.48255900
H	0.13069700	3.42496700	2.57367200
C	0.13748100	5.76823900	0.00134000
H	-0.74134200	6.32303400	0.34395800
H	1.01496500	6.32377600	0.34658400
H	0.13830300	5.75949400	-1.09351800

V-ax

B3LYP-D3 SCF energy:	-1674.981205 a.u.
B3LYP-D3 enthalpy:	-1674.407159 a.u.
B3LYP-D3 free energy:	-1674.511291 a.u.
M06 SCF energy in solution:	-1673.829046 a.u.
M06 enthalpy in solution (25°C):	-1673.914552 a.u.
M06 free energy in solution (25°C):	-1673.83235 a.u.
M06 enthalpy in solution (90°C):	-1673.934489 a.u.
M06 free energy in solution (90°C):	-1674.981205 a.u.

C	-1.37819300	-0.68691100	0.90796100
C	-0.68659800	-0.78995200	-0.47155400
C	-3.16777000	0.69541500	-0.21402200
C	-2.18785400	0.60680400	0.97191400
H	-2.06394800	-1.53875700	1.02693200
H	-3.94528900	-0.06695300	-0.06760000
H	-1.48977300	1.44752000	0.92769400
O	-2.51643900	0.47086600	-1.47516400
O	-2.95886900	0.66559300	2.17263000
O	-0.38209100	-0.73108500	1.92338700
C	-3.84822000	2.05054500	-0.30071300
H	-4.55105700	2.15013400	0.54164900
H	-4.42237700	2.09820000	-1.23925900
O	-2.86954700	3.07451000	-0.25836600
C	-3.41787300	4.36283200	-0.44550300
H	-2.59156800	5.07613800	-0.38581200
H	-3.90332500	4.45888500	-1.42981700
H	-4.16019200	4.60846700	0.33210700
C	-2.69575200	1.81602600	2.96355000
H	-1.66426100	1.81177400	3.34551700
H	-3.38976300	1.78440100	3.80841800
H	-2.85601600	2.74139800	2.39330400
C	-0.79331800	-1.34486900	3.13721400
H	-1.08541400	-2.39440400	2.97501100
H	0.07405700	-1.32276000	3.80281000
H	-1.63221200	-0.81451300	3.59912400
C	-1.77141200	-0.70189800	-1.55587600
H	-1.33166000	-0.73287400	-2.55350300

O	-2.70521600	-1.81651500	-1.42762700
C	-2.33902500	-2.99447400	-1.98744100
O	-1.28492100	-3.17409900	-2.55635000
C	-3.42160900	-4.03050000	-1.79472600
H	-3.11294100	-4.96840900	-2.25746900
H	-3.60492600	-4.18317900	-0.72587800
H	-4.35835700	-3.68086900	-2.24061500
C	0.32590700	0.29853100	-0.70512800
H	-0.05620700	1.28131700	-0.96140500
C	1.65712200	0.12162800	-0.65630600
H	-0.19677000	-1.76354700	-0.54946000
O	2.19718900	-1.12328500	-0.46484600
Si	2.95319500	-1.68560600	0.94798900
C	4.72318000	-2.13026500	0.48544900
C	2.90510300	-0.34087800	2.25996200
C	2.00037300	-3.22187000	1.46085700
H	4.74475100	-2.82637100	-0.36147400
H	5.28879200	-1.23558700	0.20161900
H	5.24445700	-2.60796100	1.32449000
H	3.51559800	0.52006400	1.96600600
H	1.87406100	0.00033800	2.39622000
H	3.28203600	-0.71409200	3.22024000
H	2.41341400	-3.67602900	2.36993800
H	0.95655500	-2.95441800	1.64988100
H	2.02109000	-3.97650400	0.66599200
C	2.63479000	1.21692200	-0.87282500
C	3.87919100	0.93999800	-1.46269700
C	2.35313000	2.53750600	-0.48575600
C	4.80814600	1.95771000	-1.67462600
H	4.10221100	-0.07852500	-1.76277500
C	3.28201400	3.55395800	-0.70032900
H	1.41196200	2.75810400	0.00921200
C	4.51346000	3.26921000	-1.29588300
H	5.76296700	1.72626200	-2.13969200
H	3.05008900	4.56887600	-0.38777000
H	5.23969300	4.06165700	-1.45593700

benzene

B3LYP-D3 SCF energy:	-232.120421 a.u.
B3LYP-D3 enthalpy:	-232.147663 a.u.
B3LYP-D3 free energy:	-232.18046 a.u.
M06 SCF energy in solution:	-232.014332 a.u.
M06 enthalpy in solution (25°C):	-232.044181 a.u.
M06 free energy in solution (25°C):	-232.014332 a.u.
M06 enthalpy in solution (90°C):	-232.050198 a.u.
M06 free energy in solution (90°C):	-232.120421 a.u.

C	-1.33964700	-0.39543500	0.00000500
C	-0.32725200	-1.35787800	0.00006900
C	1.01226500	-0.96244300	-0.00005600

C	1.33961300	0.39554700	0.00000800
C	0.32736500	1.35784700	0.00006200
C	-1.01234400	0.96236300	-0.00005800
H	-2.38186000	-0.70320600	-0.00006500
H	-0.58204400	-2.41430200	0.00008000
H	1.79994300	-1.71109500	-0.00015300
H	2.38189500	0.70305700	0.00000800
H	0.58191500	2.41432300	0.00001800
H	-1.79984600	1.71121200	-0.00006300

References

(1) Still, W. C.; Kahn, M.; Mitra, A., Rapid Chromatographic Technique for Preparative Separations with Moderate Resolution. *J. Org. Chem.* **1978,** *43*, 2923-2925.

(2) Fulmer, G. R.; Miller, A. J. M.; Sherden, N. H.; Gottlieb, H. E.; Nudelman, A.; Stoltz, B. M.; Bercaw, J. E.; Goldberg, K. I., Nmr Chemical Shifts of Trace Impurities: Common Laboratory Solvents, Organics, and Gases in Deuterated Solvents Relevant to the Organometallic Chemist. *Organometallics* **2010,** *29*, 2176-2179.

(3) Khan, I.; Reed ‐ Berendt, B. G.; Melen, R. L.; Morrill, L. C., Flp ‐ Catalyzed Transfer Hydrogenation of Silyl Enol Ethers. *Angew. Chem.* **2018,** *130*, 12536-12539.

(4) Wei, S.; Du, H., A Highly Enantioselective Hydrogenation of Silyl Enol Ethers Catalyzed by Chiral Frustrated Lewis Pairs. *J. Am. Chem. Soc.* **2014,** *136*, 12261-12264.

(5) Cazeau, P.; Duboudin, F.; Moulines, F.; Babot, O.; Dunogues, J., A New Practical Synthesis of Silyl Enol Ethers: Part. I. From Simple Aldehydes and Ketones. *Tetrahedron* **1987,** *43*, 2075-2088.

(6) Lippert, A. R.; Naganawa, A.; Keleshian, V. L.; Bode, J. W., Synthesis of Phototrappable Shape-Shifting Molecules for Adaptive Guest Binding. *J. Am. Chem. Soc.* **2010,** *132*, 15790-15799.

(7) Frick, U.; Simchen, G., Reaktionen Der Trialkylsilyl ‐ Trifluormethansulfonate, Viii. Synthese Von O ‐ (Trimethylsilyl) Keten ‐ O, N ‐ Acetalen, 2, 5 ‐ Bis (Trimethylsiloxy) Pyrrolen, ‐ Furanen Und ‐ Thiophenen. *Liebigs Ann. Chem.* **1987,** *1987*, 839-845.

(8) Grieco, P. A.; Ohfune, Y., 1-Trimethylsilyloxy-1-Cyclopropylethylene: A Useful Reagent for the Preparation Of. Beta.-Cyclopropyl-. Alpha.,. Beta.-Unsaturated Ketones. *J. Org. Chem.* **1978,** *43*, 2720-2721.

(9) Ayers, B. J.; Hollinshead, J.; Saville, A. W.; Nakagawa, S.; Adachi, I.; Kato, A.; Izumori, K.; Bartholomew, B.; Fleet, G. W. J.; Nash, R. J., Iteamine, the First Alkaloid Isolated from Itea Virginica L. Inflorescence. *Phytochemistry* **2014,** *100*, 126-131.

(10) Gouliaras, C.; Lee, D.; Chan, L.; Taylor, M. S., Regioselective Activation of Glycosyl Acceptors by a Diarylborinic Acid-Derived Catalyst. *J. Am. Chem. Soc.* **2011,** *133*, 13926-13929.

(11) Li, L.; Franckowiak, E. A.; Xu, Y.; McClain, E.; Du, W., Efficient Synthesis of Β-(1,6)-Linked Oligosaccharides through Microwave-Assisted Glycosylation. *J. Polym. Sci. A Polym. Chem.* **2013,** *51*, 3693-3699.

(12) Fürstner, A.; Radkowski, K.; Grabowski, J.; Wirtz, C.; Mynott, R., Ring-Closing Alkyne Metathesis. Application to the Total Synthesis of Sophorolipid Lactone. *J. Org. Chem.* **2000,** *65*, 8758-8762.

(13) Camponovo, J. r. m.; Hadad, C.; Ruiz, J.; Cloutet, E.; Gatard, S.; Muzart, J.; Bouquillon, S.; Astruc, D., "Click" Glycodendrimers Containing 27, 81, and 243 Modified Xylopyranoside Termini. *J. Org. Chem.* **2009,** *74*, 5071-5074.

(14) Adelhorst, K.; Whitesides, G. M., Large-Scale Synthesis of Β-L-Fucopyranosyl Phosphate and the Preparation of Gdp-Β-L-Fucose. *Carbohydr. Res.* **1993,** *242*, 69-76.

(15) Zhu, X.-R.; Zheng, Y.; Wang, P.; Yang, S.-B.; Chen, R.; Zhu, Y.-Q., Synthesis of Two Metabolites of Edaravone. *J. Chin. Pharm. Sci.* **2010,** *19*, 307.

(16) (a) Porwanski, S.; Marsura, A., Tandem Staudinger–Aza–Wittig Templated Reaction: De Novo Synthesis of Sugar–Ureido Cryptands. Wiley Online Library: 2009; (b) Zhao, J.; Wei, S.; Ma, X.; Shao, H., A Mild and Environmentally Benign Method for the Synthesis of Glycals in Peg-600/H 2 O. *Green Chem.* **2009,** *11*, 1124-1127.

(17) Yuan, X.; Cheng, S.; Shi, Y.; Xue, W., Photocatalytic Synthesis of Glycosyl Bromides. *Synthesis* **2014,** *46*, 331-335.

(18) Lichtenthaler, F. W.; Köhler, B., An Expedient Route to Acylated Glucosyl Halides with a Free 2-Oh Group. *Carbohydr. Res.* **1994,** *258*, 77-85.

(19) Khatri, V.; Kumar, A.; Singh, B.; Malhotra, S.; Prasad, A. K., Synthesis of Β-C-Glycopyranosyl Aldehydes and 2, 6-Anhydro-Heptitols. *J. Org. Chem.* **2015,** *80*, 11169-11174.

(20) Cheng, W.-M.; Shang, R.; Fu, Y., Irradiation-Induced Palladium-Catalyzed Decarboxylative Desaturation Enabled by a Dual Ligand System. *Nature communications* **2018,** *9*, 1-9.

(21) Cismesia, M. A.; Yoon, T. P., Characterizing Chain Processes in Visible Light Photoredox Catalysis. *Chemical Science* **2015,** *6*, 5426-5434.

(22) (a) Hatchard, C. G.; Parker, C. A.; Bowen Edmund, J., A New Sensitive Chemical Actinometer - Ii. Potassium Ferrioxalate as a Standard Chemical Actinometer. *Proc. R. Soc. Lond. A Math. Phy. Sci.* **1956,** *235*, 518-536; (b) Kuhn, H. J.; Braslavsky, S. E.; Schmidt, R., Chemical Actinometry (Iupac Technical Report). *Pure Appl. Chem.* **2004,** *76*, 2105-2146; (c) Montalti, M.; Credi, A.; Prodi, L.; Gandolfi, M. T., *Handbook of Photochemistry*. CRC Press: 2006.

(23) Legault, C. Y., *Cylview20*, Université De Sherbrooke, **2020** http://www.cylview.org.

(24) Gaussian 16, Revision C.01, Frisch, M. J.; Trucks, G. W.; Schlegel, H. B.; Scuseria, G. E.; Robb, M. A.; Cheeseman, J. R.; Scalmani, G.; Barone, V.; Petersson, G. A.; Nakatsuji, H.; Li, X.; Caricato, M.; Marenich, A. V.; Bloino, J.; Janesko, B. G.; Gomperts, R.; Mennucci, B.; Hratchian, H. P.; Ortiz, J. V.; Izmaylov, A. F.; Sonnenberg, J. L.; Williams-Young, D.; Ding, F.; Lipparini, F.; Egidi, F.; Goings, J.; Peng, B.; Petrone, A.; Henderson, T.; Ranasinghe, D.; Zakrzewski, V. G.; Gao, J.; Rega, N.; Zheng, G.; Liang, W.; Hada, M.; Ehara, M.; Toyota, K.; Fukuda, R.; Hasegawa, J.; Ishida, M.;

Nakajima, T.; Honda, Y.; Kitao, O.; Nakai, H.; Vreven, T.; Throssell, K.; Montgomery, J. A., Jr.; Peralta, J. E.; Ogliaro, F.; Bearpark, M. J.; Heyd, J. J.; Brothers, E. N.; Kudin, K. N.; Staroverov, V. N.; Keith, T. A.; Kobayashi, R.; Normand, J.; Raghavachari, K.; Rendell, A. P.; Burant, J. C.; Iyengar, S. S.; Tomasi, J.; Cossi, M.; Millam, J. M.; Klene, M.; Adamo, C.; Cammi, R.; Ochterski, J. W.; Martin, R. L.; Morokuma, K.; Farkas, O.; Foresman, J. B.; Fox, D. J. Gaussian, Inc., Wallingford CT, **2016**.

(25) (a) Lee, C.; Yang, W.; Parr, R. G., Development of the Colle-Salvetti Correlation-Energy Formula into a Functional of the Electron Density. *Phy. Rev. B* **1988,** *37*, 785; (b) Becke, A. D., Density‐Functional Thermochemistry. Iii. The Role of Exact Exchange. *J. Chem. Phys.* **1993,** *98*, 5648-5652.

(26) Grimme, S.; Antony, J.; Ehrlich, S.; Krieg, H., A Consistent and Accurate Ab Initio Parametrization of Density Functional Dispersion Correction (Dft-D) for the 94 Elements H-Pu. *J. Chem. Phys.* **2010,** *132*, 154104.

(27) Zhao, Y.; Truhlar, D. G., The M06 Suite of Density Functionals for Main Group Thermochemistry, Thermochemical Kinetics, Noncovalent Interactions, Excited States, and Transition Elements: Two New Functionals and Systematic Testing of Four M06-Class Functionals and 12 Other Functionals. *Theor. Chem. Acc.* **2008,** *120*, 215-241.

(28) Marenich, A. V.; Cramer, C. J.; Truhlar, D. G., Universal Solvation Model Based on Solute Electron Density and on a Continuum Model of the Solvent Defined by the Bulk Dielectric Constant and Atomic Surface Tensions. *J. Phys. Chem. B* **2009,** *113*, 6378-6396.

(29) Funes-Ardoiz, I.; Paton, R. S., Goodvibes: Version 2.0. 3. **2018**.

(30) Ribeiro, R. F.; Marenich, A. V.; Cramer, C. J.; Truhlar, D. G., Use of Solution-Phase Vibrational Frequencies in Continuum Models for the Free Energy of Solvation. *J. Phys. Chem. B* **2011,** *115*, 14556-14562.

(31) Mammen, M.; Shakhnovich, E. I.; Deutch, J. M.; Whitesides, G. M., Estimating the Entropic Cost of Self-Assembly of Multiparticle Hydrogen-Bonded Aggregates Based on the Cyanuric Acid⊙Melamine Lattice. *J. Org. Chem.* **1998,** *63*, 3821-3830.

(32) (a) Grimme, S., Exploration of Chemical Compound, Conformer, and Reaction Space with Meta-Dynamics Simulations Based on Tight-Binding Quantum Chemical Calculations. *J. Chem. Theory Comput.* **2019,** *15*, 2847-2862; (b) Pracht, P.; Bohle, F.; Grimme, S., Automated Exploration of the Low-Energy Chemical Space with Fast Quantum Chemical Methods. *Phys. Chem. Chem. Phys.* **2020,** *22*, 7169-7192.

(33) (a) Grimme, S.; Bannwarth, C.; Shushkov, P., A Robust and Accurate Tight-Binding Quantum Chemical Method for Structures, Vibrational Frequencies, and Noncovalent Interactions of Large Molecular Systems Parametrized for All Spd-Block Elements (Z= 1–86). *J. Chem. Theory Comput.* **2017,** *13*, 1989-2009; (b) Bannwarth, C.; Ehlert, S.; Grimme, S., Gfn2-Xtb—an Accurate and Broadly Parametrized Self-Consistent Tight-Binding Quantum Chemical Method with Multipole Electrostatics and Density-Dependent Dispersion Contributions. *J. Chem. Theory Comput.* **2019,** *15*, 1652-1671.
